# What you sample is what you get: ecomorphological variation in *Trithemis* (Odonata, Libellulidae) dragonfly wings reconsidered

**DOI:** 10.1186/s12862-022-01978-y

**Published:** 2022-04-11

**Authors:** Norman MacLeod, Benjamin Price, Zackary Stevens

**Affiliations:** 1 School of Earth Sciences and Engineering, Nanjing University, 163 Xianlin Avenue, Nanjing, 210023 Jiangsu China; 2 Department of Life Sciences, The Natural History Museum, Cromwell Road, London, SW7 5BD UK; 3 School of Earth and Environmental Sciences, Cardiff University, Main Building, Cardiff, CF10 3AT UK

**Keywords:** *Trithemis*, Odonata, Dragonfly, Morphology, Ecology, Geometric morphometrics, Machine learning, Convolution neural network

## Abstract

**Background:**

The phylogenetic ecology of the Afro-Asian dragonfly genus *Trithemis* has been investigated previously by Damm et al. (in Mol Phylogenet Evol 54:870–882, 2010) and wing ecomorphology by Outomuro et al. (in J Evol Biol 26:1866–1874, 2013). However, the latter investigation employed a somewhat coarse sampling of forewing and hindwing outlines and reported results that were at odds in some ways with expectations given the mapping of landscape and water-body preference over the *Trithemis* cladogram produced by Damm et al. (in Mol Phylogenet Evol 54:870–882, 2010). To further explore the link between species-specific wing shape variation and habitat we studied a new sample of 27 *Trithemis* species employing a more robust statistical test for phylogenetic covariation, more comprehensive representations of *Trithemis* wing morphology and a wider range of morphometric data-analysis procedures.

**Results:**

Contrary to the Outomuro et al. (in J Evol Biol 26:1866–1874, 2013) report, our results indicate that no statistically significant pattern of phylogenetic covariation exists in our *Trithemis* forewing and hindwing data and that both male and female wing datasets exhibit substantial shape differences between species that inhabit open and forested landscapes and species that hunt over temporary/standing or running water bodies. Among the morphometric analyses performed, landmark data and geometric morphometric data-analysis methods yielded the worst performance in identifying ecomorphometric shape distinctions between *Trithemis* habitat guilds. Direct analysis of wing images using an embedded convolution (deep learning) neural network delivered the best performance. Bootstrap and jackknife tests of group separations and discriminant-function stability confirm that our results are not artifacts of overtrained discriminant systems or the “curse of dimensionality” despite the modest size of our sample.

**Conclusion:**

Our results suggest that *Trithemis* wing morphology reflects the environment’s “push” to a much greater extent than phylogeny’s “pull”. In addition, they indicate that close attention should be paid to the manner in which morphologies are sampled for morphometric analysis and, if no prior information is available to guide sampling strategy, the sample that most comprehensively represents the morphologies of interest should be obtained. In many cases this will be digital images (2D) or scans (3D) of the entire morphology or morphological feature rather than sparse sets of landmark/semilandmark point locations.

**Supplementary Information:**

The online version contains supplementary material available at 10.1186/s12862-022-01978-y.

## Background

The interplay between the effects of phylogenetic relations among species, and the role of the environment in shaping the range of morphologies we observe in nature, has been a subject of perennial interest for those who seek to understand evolutionary processes. That both factors have effected biodiversity in the distant past, as well as in the present day, is beyond question. But appreciating the extent to which one factor has exerted dominance over the other—whether the forms we observe in nature are the result of phylogeny’s “pull” or the environment’s “push”—is an issue that must be considered on a case-by-case basis.

Prior to the advent of phylogenetic systematics and the revolution wrought by the introduction of molecular data into systematics, the environment was considered to have the upper hand in this contest. Even into the distant reaches of antiquity, when a species that exhibited a novel combination of morphological characteristics was discovered, the first question most naturalists asked was what the structure could be used for; what was its purpose?

In their influential 1979 essay, Stephen Jay Gould and Richard Lewontin referred to this point-of-view as the “adaptationist programme”; the idea that the environment, through the agency of natural selection, optimized all aspects of a species’ morphology, physiology, behavior, etc. for the conditions present in the environments they inhabited. As an alternative to this view of life, Gould and Lewontin [1] offered a model that reinforced the potential role of phylogenetic history in such explanatory narratives. Of course, all species must meet the functional challenges posed by their local environments. But does this really, or always, mean that every aspect of a species’s biology is optimized for some proximate purpose by natural selection at all times throughout its evolutionary history? Or should species morphologies be regarded as integrated Baupläne, constrained by phyletic inheritance, some of whose attributes are shaped by the needs of present environmental interactions but others of which are neutral in the face of selection? Indeed, they might even be slightly disadvantageous to individual survival if they were the by-products of a developmental system that served some larger adaptive purpose. In raising this alternative explanation Gould and Lewontin did not resolve the question of how best to interpret either novel or ordinary morphological structures. They only extended the range of interpretations, and so the range and types of evidence, that might be brought to bear on this question’s resolution.

Today, we have much better conceptual and analytic tools for discovering and describing patterns in the distribution of morphological features, along with much better ways of estimating degrees of phylogenetic relations within groups of species. In particular, Felsenstein and later colleagues’ work on the biological comparative method [2–7] have addressed many long-standing statistical difficulties surrounding attempts to analyze patterns of similarity and difference across groups of taxa that are embedded in a network of ancestry and descent. While work remains to be done in this area (e.g., [8]), many outstanding problems that have complicated, and in many cases compromised, the research of evolutionary biologists for generations have been overcome.

In addition, new tools have become available for undertaking exploratory analyses of morphological structure. Of particular note, the geometric morphometric (GM) approach has done much to encourage the quantitative analysis of morphological data. This approach to morphometric analysis (see [9–14]) is now over 30 years old and can no longer be described as a “new” development (see [15, 16]). While GM constitutes a very valuable set of tools, procedures and standards for testing many types of morphological hypotheses, its core paradigm—that patterns of morphological variation be described via reference to sparse sets of landmark and/or semilandmark locations—requires that the features of greatest morphological interest be known at the outset of an investigation. The geometries of these features must also be capable of being represented adequately by a small number of two-dimensional (2D) or three-dimensional (3D) point coordinates. These point-coordinate locations should, ideally (1) be distributed more-or-less evenly across the morphologies or structures in question, (2) be able to be located unambiguously at topologically homologous positions and (3) be able to be located on every specimen in the sample. So long as morphological comparisons are being made across a set of well-preserved and morphologically similar species, this approach works well. Nevertheless, as the taxonomic scope and/or spatial detail of a morphological investigation increases, the ability of a sample to meet these rather stringent requirements often decreases, resulting in a concomitant decrease in the power, and so the appropriateness, of classic GM-style analyses.

Over the last two decades a completely novel approach to the analysis of morphological variation has been developed in the form of machine learning (ML) algorithms. While this approach has its origins in regression-based procedures that would be familiar to any GM practitioner (e.g., linear regression, principal component analysis (PCA), linear discriminant analysis), its core algorithms have been incorporated into such complex data-analysis system designs that their regression-based origins have become obscure to many casual users. Nonetheless, it is this complexity that gives ML algorithms their extraordinary power; a power that has provoked both amazement and, in certain areas, no small amount of concern. Despite many broad and convincing demonstrations that ML-based approaches can sense and identify patterns of variation in a very wide range of data types (and so in a very wide range of data-analysis contexts), systematic biologists have been slow to avail themselves of these new data-analysis tools and integrate them into their research programs.

Machine-learning algorithms are well suited to deliver the sorts of analyses GM-style approaches struggle to provide. One prominent example is the exploratory search for morphological differences between a priori defined taxonomic groups, especially in those (common) instances where there is little consensus among experts as to which morphological features carry group-diagnostic signal(s). This is precisely the type of problem many “deep learning”, or convolution (artificial) neural networks (CNNs), were designed originally to address [17–22]. The performance of CNN-based systems on standardized, human-validated, image-challenge datasets is what, arguably, was responsible for the current renaissance of interest in ML techniques (e.g., [23, 24]) and, more generally, in artificial intelligence (see [25]).

Insect wing morphology has long been recognized as having great potential for exploring and illustrating the advantages of quantitative morphological analysis in both taxonomic and ecological contexts. Some of the first publications illustrating the use of GM methods took insect-wing morphology as their subject (e.g., [26–28]). Insect wings are complex structures for which an extensive descriptive nomenclature has been developed [29, 30], In particular, the intersections or nodes of insect wing veins form classic Type 3 landmark positions [11], many of which can be relocated across a surprisingly broad range of species. This observation, along with the consistency of form or relative positions of many major structural veins, lends support to the widely-held belief among entomologists that wings constitute biological homologues for insects as a whole [30].

Much recent quantitative research has documented the fact that insect wing morphology can be used to identify species (e.g., [26, 31, 32]), populations (e.g., [33]) and even sexes [34]. But, while much remains to be learned about the relation between wing shape and aerodynamic function [35], mechanistic and observational evidence indicates that insect wings of different shapes and internal structural arrangements are associated with different aerial capabilities [36]. This suggests that, like the wings of birds [37–39] and bats [40], insect wing form might reflect aspects of species’ preferred environment(s), a suggestion for which there is a limited amount of positive evidence (see [35]).

Unfortunately, the morphological complexities of the insect wing also pose a number of challenges for quantitative morphological analysis. The sheer number of vein domains (sensu [41, 42]) comprising many insect wings makes it difficult to decide how to characterize wing morphology and the level of detail required to resolve particular morphology-related questions. It is, of course, always tempting to employ as much data as can be collected in attempts to resolve outstanding issues of controversy and/or develop comprehensive summaries of morphological trends. However, the well-known “curse of dimensionality” often renders datasets, in which the number of variables greatly exceeds the number of samples or specimens available, difficult to analyze (see [43]), especially when the task is to achieve reliable between-groups discrimination ([44], but see [45, 46]). Related to this question are perennial concerns regarding whether it is better to focus analysis on the locations of landmark configurations that represent aspects of the wing’s internal morphology, or the geometry of the wing outline. Then there is the question what to do about the coloration patterns that are an intrinsic part of the morphology of many insect wings and may have significant species characterization/identification and/or behavioral roles, but that resist attempts to characterize them consistently or accurately across even modestly sized samples via reference landmark or semilandmark data (Additional file 1).

The difficulties raised by these considerations are compounded by the fact that, at the outset of an investigation, many researchers have little idea which aspect(s) of the morphology are best suited to resolving particular questions. Yet, the outcome of any morphological hypothesis test wholly depends on decisions regarding which aspect(s) of the morphology to measure. If a poor choice is made, inadvertently, and a negative result obtained, is it appropriate to conclude that the structure or character complex in question does not exhibit the pattern of variation predicted by the hypothesis test? Or could it be that the data used to represent the structure or character complex in question do not exhibit the pattern(s) of variation predicted by the hypothesis test, but that other, as yet unsampled, aspects of the morphology might?

In 2013 Outomuro et al. [47] published a study that compared wing-shape and habitat variation in dragonflies belonging to the Afro-Asian genus *Trithemis*. These species occur in a variety of ecological habitats with some species preferring forests to open country, others preferring to hunt in the vicinity of permanent running streams while others are found typically around temporary or standing pools. This study also compared sexual dimorphism in wing shape across the species included in the sample. These authors found significant phylogenetic covariation among wing shapes, no significant association between wing shape and water body type, a contrast between forewing and hindwing shape in terms of the ability of these structures to reflect landscape type, and a distinct difference among males and females belonging to the same species. Accordingly, Outomuro et al. [47] concluded that “natural and sexual selection are acting partially independently on [*Trithemis*] fore- and hind- wings and with differences between the sexes, despite evidence for phenotypic correlation of wing shape between males and females” (p. 1866).

We find nothing wrong or amiss with any of these conclusions. Nevertheless, we wonder whether this represents the whole of the story embodied by the wing morphologies of *Trithemis* dragonflies. Outomuro et al. [47] employed a decidedly non-standard manner of representing both forewing and hindwing shapes (see Fig. 1 of [47]). Their study focused on quite a sparse set of landmark locations collected from the wing peripheries with complications arising from the fact that these data (apparently) were collected from specimens in which the outlines of the forewings and hindwings overlapped. This may have prevented precise location of landmarks along the trailing, proximal edge of the forewing. In addition, the location of forewing landmarks was quite uneven with the morphology of the trailing edge and wing tip being sampled much more intensively than the leading edge proximal wing margin. These same spatial discrepancies in sampling intensity were also present for the hindwing. The effect of using sets of sparse and unevenly distributed landmarks to represent wing form would be to characterize only portions of each wings’ overall geometry and to differentially weight variation in those parts of the wings sampled more intensively relative to those sampled using fewer landmarks.

**Fig. 1:**
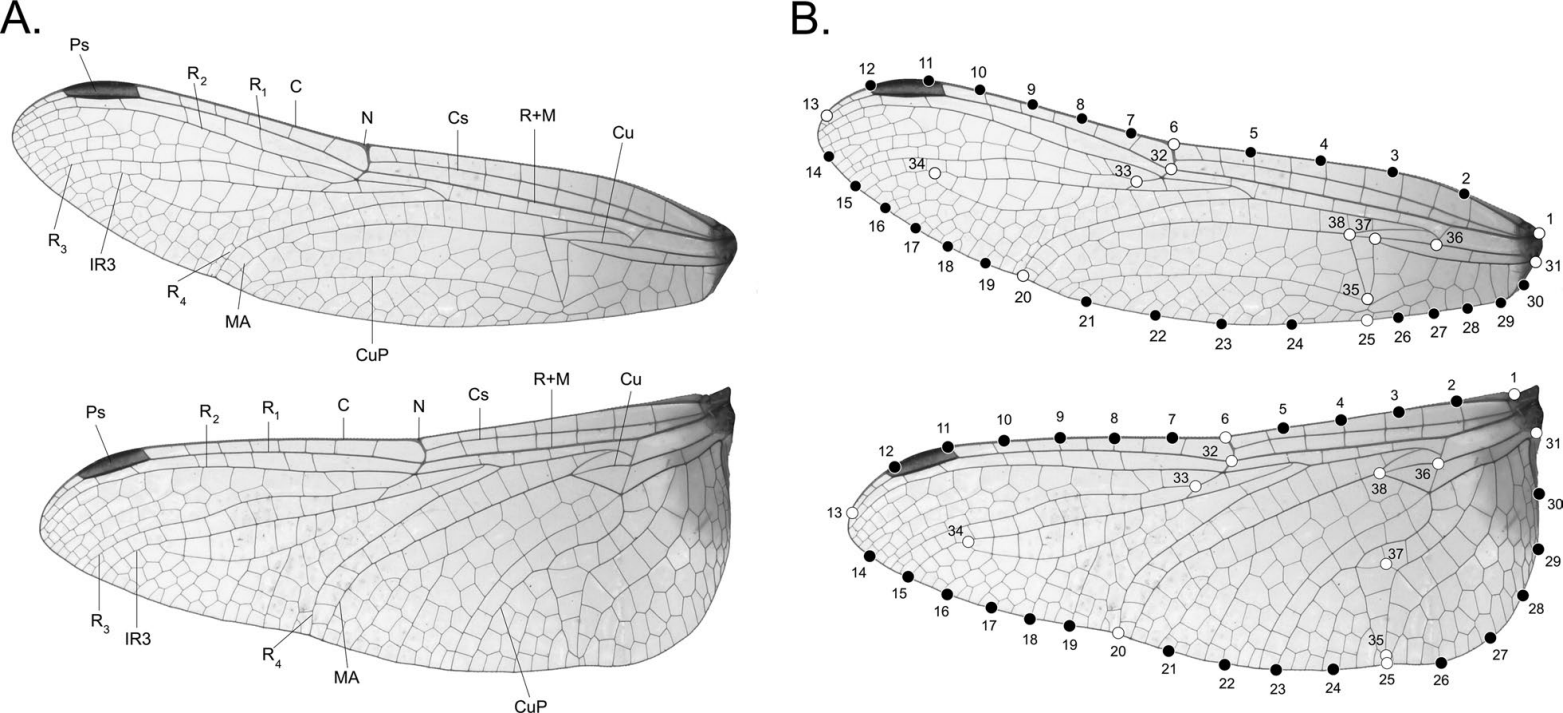
A Major morphological features of the *Trithemis* forewing (top) and hindwing (bottom). C = costa; N = nodus, Cs = subcosta; R + M = radius and media; R_1_ = first radius; R_2_ = second radius; R_3_—third radius; R_4_ = fourth radius; MA = media anterior; IR_3_ = intercalary vein behind R_3_, Cu = cubitus; CuP = cubitus posterior, Ps = pterostigma. Note that, unlike other dragonfly genera, the major structures features of the *Trithemis* forewing and hindwing are quite similar (see also Additional file 1: Plates 1 and 2), facilitating detailed direct comparisons between individual wings and across species within this genus. B Positions of the 38 landmarks (white) and semilandmarks (black) used to quantify *Trithemis* forewing (top) and hindwing (bottom) form. Landmarks are located at the origins, intersections or peripheral termini of major wing veins (see Additional files 2: Landmark-Semilandmark Definitions). Peripheral landmarks 1, 6, 13, 20, 25 and 31 were used to subdivide the wing outlines into five zones with the number of evenly spaced semilandmarks interpolated within these zones being sufficient to represent their zone-based peripheral outline geometries to a minimum accuracy of > 95% across all specimens in the sample. This form-sampling system ensures accurate and even representation of the wing outline characterization and improves inter-semilandmark correspondence across specimens

Outomuro et al. [47] also appear to have used an odd scheme of angles to locate peripheral landmarks in the anal region of the hindwing’s trailing edge, with positions located at 10°, 27°, 54°, 73°, 81° and 90° from a chord drawn between the wing’s anterior thoracic articulation and the intersection between the nodus to the wing periphery (at least, as shown in Fig. 1 of [47]). Other than this figure, no definitions of either the forewing or the hindwing landmarks were provided though, in all cases other than those noted above, the landmarks appear to have been placed at the intersections between major wing veins (e.g., costa, subcosta, first radius), or pterostigma (see Fig. 1A), and the wing periphery. Standard GM practice would be to represent each wing’s outline shape using a set of (initially) equally spaced semilandmark points from a common starting landmark in order to ensure consistent resolution across the dataset and across all forms comprizing the sample.

Given these aspects of the Outomuro et al. [47] analysis, in addition to the fact that no internal landmarks were employed in the characterization of wing morphology, we suspected that potentially important aspects of the relation between *Trithemis* wing form and habitat guild may have been overlooked. Accordingly, we chose to determine (1) whether we could reproduce the findings of Outomuro et al. [47] in terms of the relation between wing shape and both landscape and water body preference using different form-characterization standards and different data-analysis methods and (2) whether there was anything more this dragonfly genus had to tell us about the relation between wing morphology, ecology and natural selection. This comparison was also undertaken to (3) clarify the range of options available to quantitative morphologists interested in confronting similar problems in other taxonomic groups and (4) provide a relative assessment of the power of different data types and data-analysis methods when used in similar ecomorphological contexts.

## Results

### Phylogenetic signal analyses

**Fig. 2:**
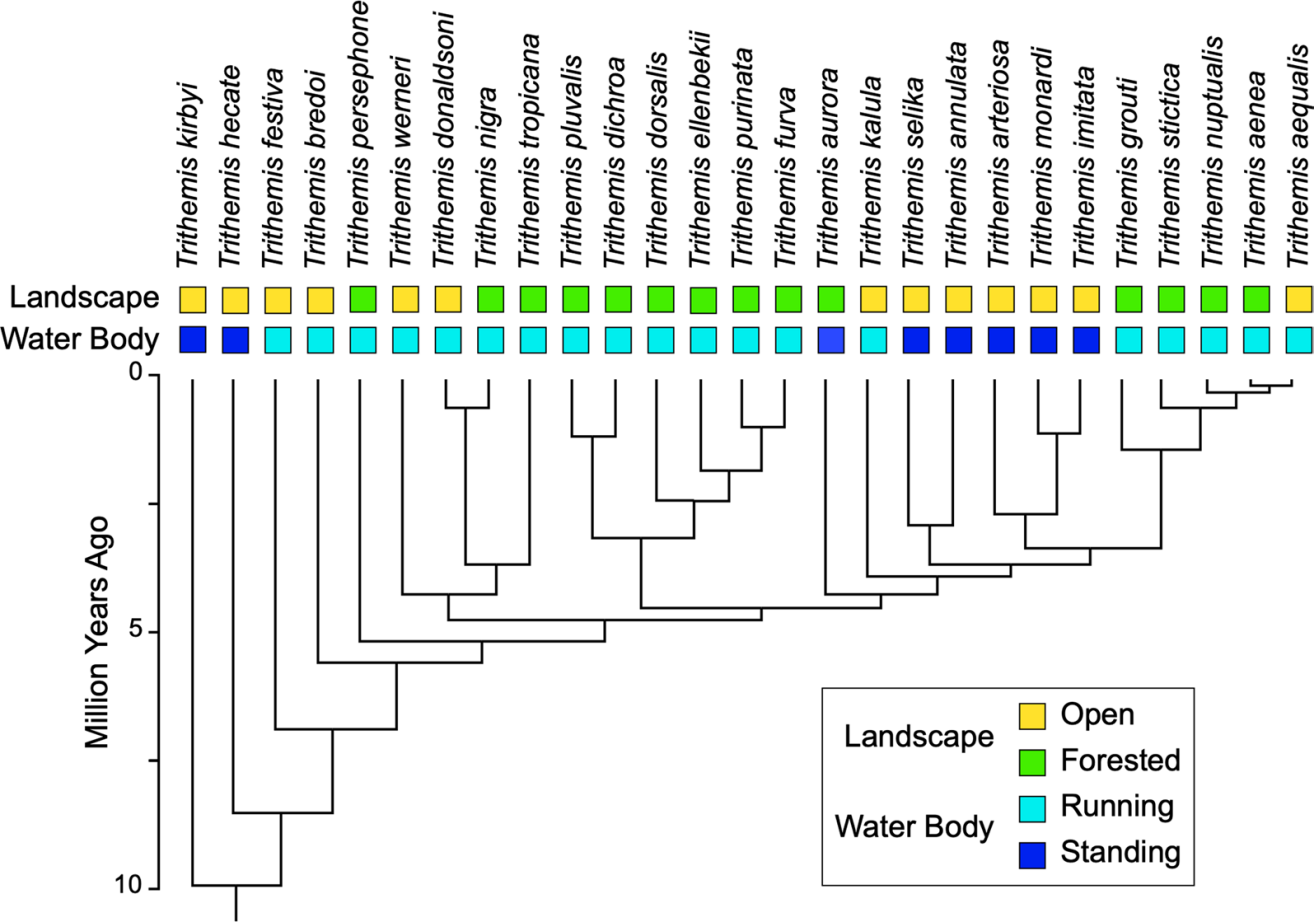
Ultrametric *Trithemis* cladogram (modified from [49]) showing phylogenetic relations for all species included in this investigation. This tree was inferred from ND1 and 16S genetic sequences based on maximum likelihood branch lengths. Time calibration was based on the r8s sequence using a 10 mya estimate for the basal *Trithemis* node which itself was based on data from the fossil record [50]. Landscape characters also from [49]. Note the manner in with the distributions of landscape and water-body preferences are arrange as phylogenetic grades rather than phylogenetic clades

The degree to which patterns of shape variation in the *Trithemis* species considered here covary with the pattern of *Trithemis* ancestry and descent (Fig. 2) was examined for mean forewing and hindwing landmark-semilandmark shape configurations using the *K*_mult_ test described by [48]. This statistic tests the null hypothesis that the degree of morphological shape similarity existing among a set of species reflects the structure of phylogenetic relations existing between species under the assumption that morphological evolution conforms to a Brownian-motion model with an expected value of 0.0 and a variance (*σ*^2^) proportional to the elapsed time since speciation from a common ancestor. As noted by Adams [48], the random, or Brownian, expectation for GM data is derived by calculating the ratio between the observed square of the distance-based deviation of each species’ mean shape-coordinate configuration from the phylogenetic mean (= mean square error observed) and the expected square of the distance-based deviation of each species’ mean shape-coordinate configuration from the phylogenetic mean along the phylogenetic tree (= mean square error expected). This ratio is then scaled by the phylogenetic covariance matrix. Values less than, or greater than, 95 percent of the expected distribution of *K*_mult_ values for our sample of 27 species reflect patterns of shape variation that was either less than, or greater than expected under a random phylogeny model (= no phylogenetic signal) respectively. In order to avoid the need to make unsubstantiated assumptions with regard to interactions among variables, the expected *K*_mult_ distributions conforming to the null hypothesis of no phylogenetic covariation were estimated via a bootstrap strategy, by permuting the tips of the *Trithemis* tree randomly 1000 times and calculating the expected *K*_mult_ value for each permuted tree.

**Fig. 3:**
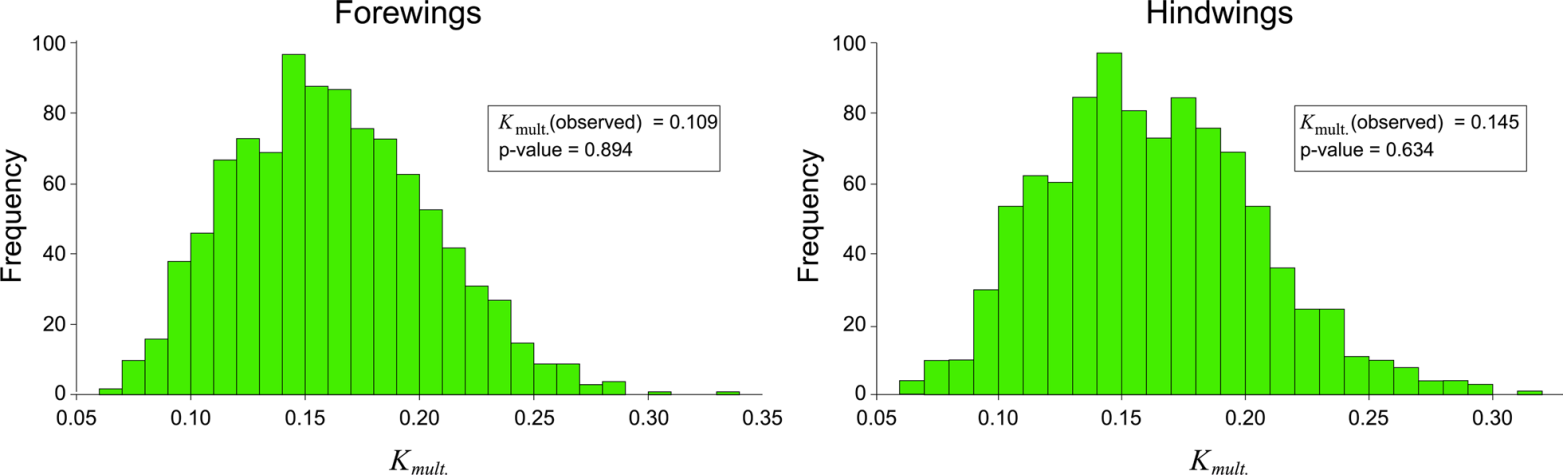
Bootstrapped phylogenetic signal distributions for the *Trithemis* forewing and hindwing landmark-semilandmark shape datasets. Note that, in both cases, the observed values of the K_mult_ statistic fall well into the range expected for randomized phylogenies. Accordingly, there are no grounds for concluding either forewing or hindwing shape distributions reflect phylogenetic covariation in *Trithemis* to a statistically significant extent

Results of the *K*_mult_ tests for forewing and hindwing landmark-semilandmark datasets (Fig. 3) show that, for both wings, the *K*_mult_ values calculated from the *Trithemis* phylomorphospace failed to indicate that these wing shapes contained a statistically significant signal of phylogenetic covariation. Given the number of studies demonstrating significant patterns of phylogenetic covariation in a host of morphological, ecological, and behavioral variables this result might strike some as unexpected. However, most of this extensive literature involves the analysis of single or pairs of metric traits or categorical variables. Owing to the lack of generalized tests for the extent of phylogenetic signals in morphometric data it is presently unknown whether this result would be regarded as common, or unusual, if a substantial body of similar, published reports, were available. What can be said without doubt, however, is that these GM-based characterizations of our *Trithemis* forewing and hindwing morphologies—which are the most comprehensive and detailed collected to date—failed to yield any evidence for patterns of wing-shape variation consistent with their phylogenetic structure.

**Fig. 4:**
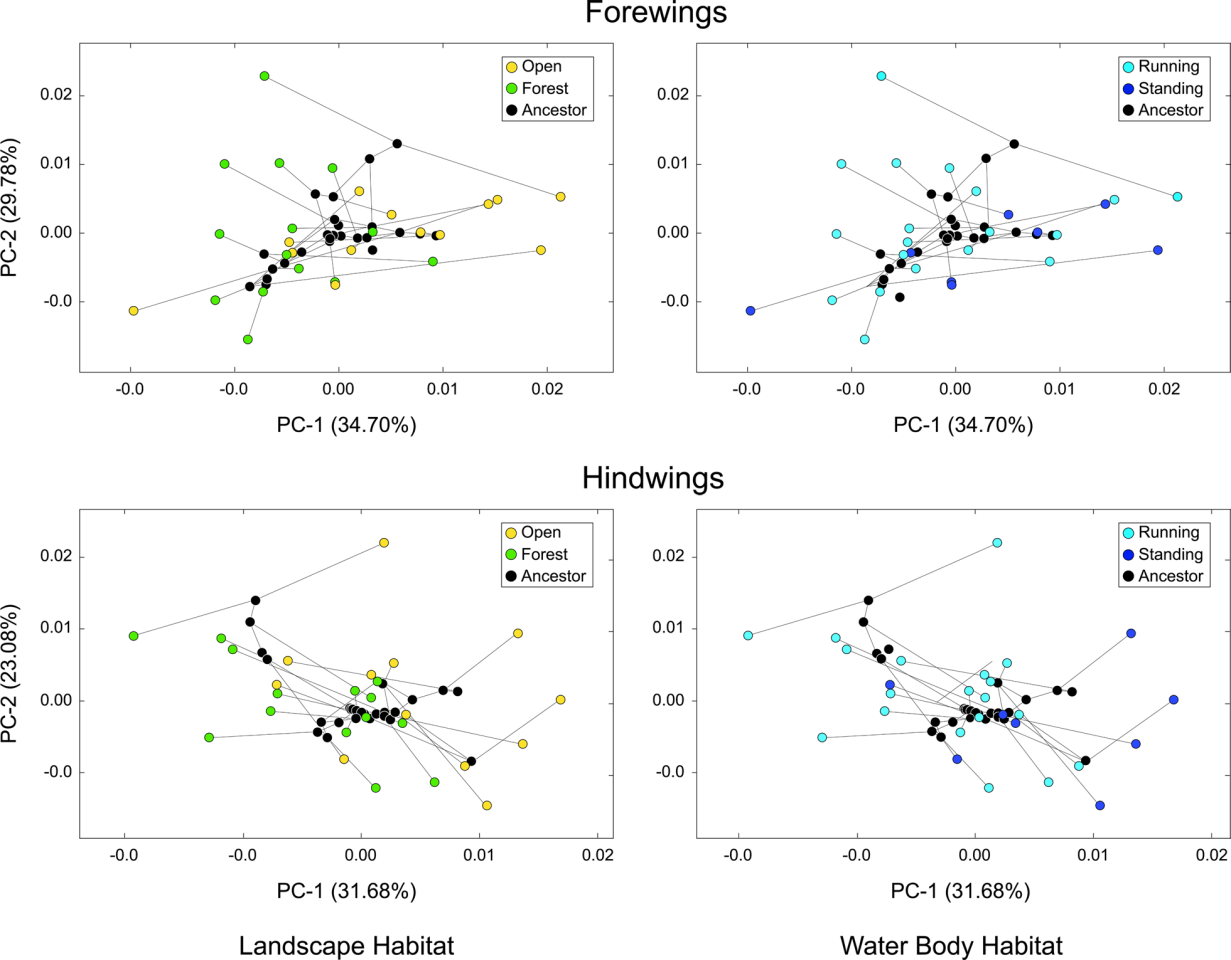
Phylomorphospace plots for the subspaces formed by the first two Procrustes principal components calculated from the *Trithemis* forewing and hindwing landmark-semilandmark datasets. Projected positions of the *Trithemis* (tree tip) species color coded to reflect their landscape and water body habitat preferences. Note the number of tree branches that cross one another in this space, indicating that the distribution of wing shapes is not being controlled, or reflecting phylogenetic structure to any substantial extent. These results support the findings of the statistical phylogenetic signal test (K_mult_) shown in Fig. 3

An analysis of the Procrustes PCA spaces created as a by-product of the phylogenetic signal test (Fig. 4) both supports and clarifies the nature of this phylogenetic covariation test result. These diagrams show that hypothetical ancestral shapes (= internal tree nodes) are clustered in the centers of these spaces; indicating they exhibit closer correspondence to the phylogenetic mean wing shape configuration than is typical of the terminal taxa. Projected positions of the terminal branches (= *Trithemis* species) form a “halo” of relatively more extreme wing shapes surrounding those estimated for the hypothetical *Trithemis* ancestors. However, the tree branches connecting internal nodes with each other, and with the terminal *Trithemis* species, exhibit a high degree of network crossover. This result indicates that species from very different parts of the *Trithemis* phylogeny exhibit similar forewing and hindwing shapes, and that closely related species often exhibit quite different and distinct forewing and hindwing shapes. Thus, there is little evidence in these data for any substantial degree of phylogenetic covariance in the structure of *Trithemis* forewing or hindwing shapes; a visual morphometric result that supports the results obtained from the *K*_mult_ statistical phylogenetic covariation tests.

The lack of obvious habitat-based clustering of *Trithemis* species in this (or any other) Procrustes PCA subspace suggests there is little evidence supporting the idea that the wing shapes of *Trithemis* species reflect either landscape or water body habitat preferences. However, these analytic results constitute a weak test of this ecomorphological hypothesis insofar as the shape ordinations that result from any PCA are optimized to reflect the directions of maximum variance in the pooled sample, without taking account of any differences that may, or may not, exist between subsidiary groups. The correct interpretation of these results, with respect to testing hypotheses of group distinction, is that, if any group-level shape distinctions are present within these data, they are not aligned with the directions of pooled-sample wing-shape variance. Owing to the nature of the optimizations present in PCA ordination spaces, this interpretational constraint applies not only to visual inspections of the ordination-space patterns, but also to any statistical tests based on these PCA score data, no matter how many components are included in such tests. Accordingly, neither inspection of PCA ordination plots such as these, nor analysis of PCA score data, constitutes a sufficient basis on which to conclude that group-level shape distinctions do not exist within morphometric data.

### Geometric morphometric (GM)-style analyses of landmark-semilandmark datasets

**Fig. 5:**
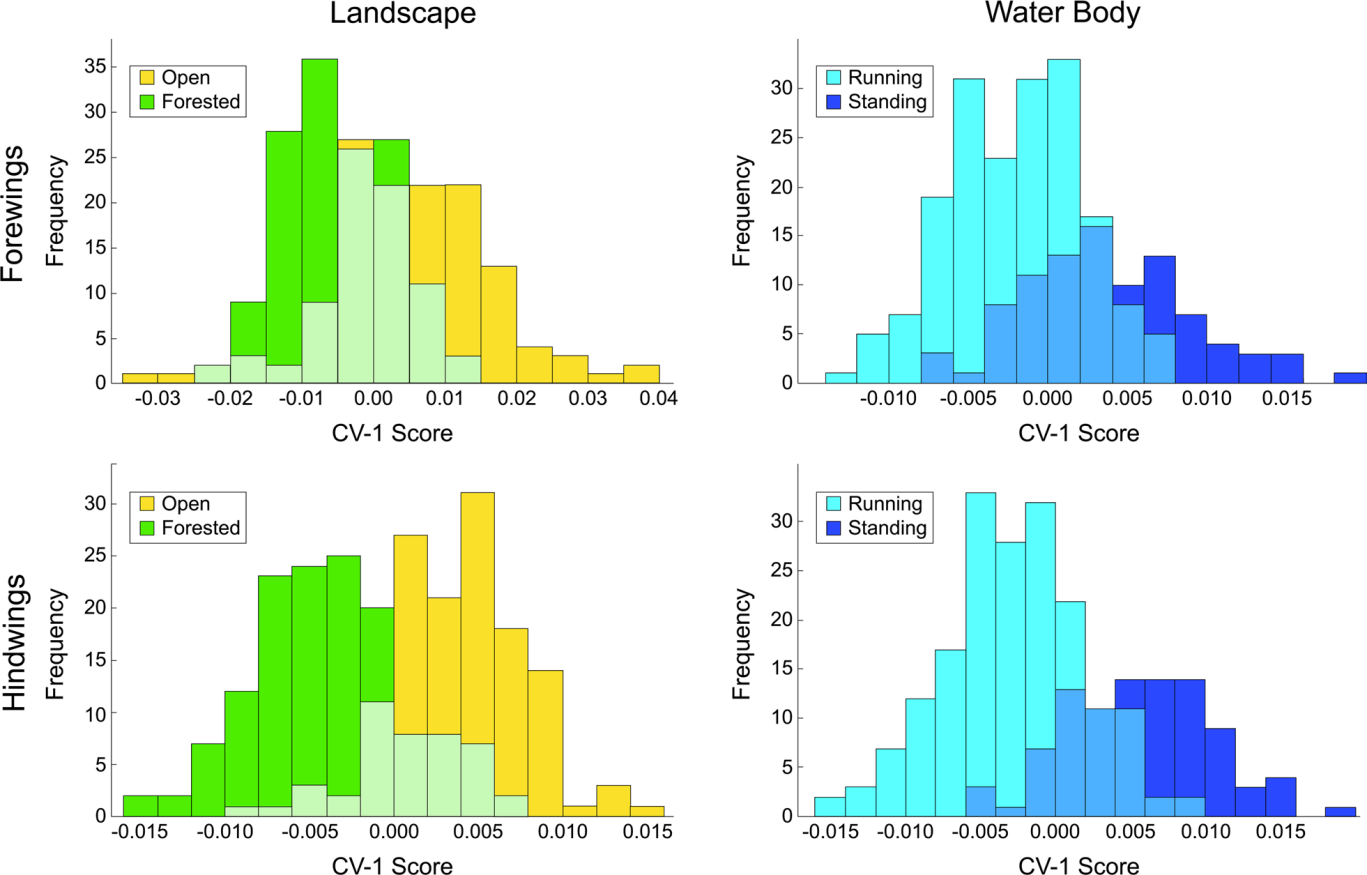
Frequency histograms for *Trithemis* forewing and hindwing shape distinctions for species preferring different landscape and water body habitats as assessed from the landmark-semilandmark coordinate datasets. Note that, despite the broad ranges of shape overlap, a clear distinction exists between the central tendencies of these habitat-based wing shape groups. Statistical comparison of the between-groups separation relative to within-groups dispersion using a bootstrapped version of Hotelling's T^2^ test indicate that all four comparisons are significant at well beyond the p = 0.95 confidence level

The issue of whether habitat-based, wing-shape distinctions do exist within these GM-style data was addressed by performing a CVA on the PCA scores of wing-shape configurations for the set of Procrustes principal components (= covariance eigenvectors) that together accounted for 95 percent of the observed shape variance for both the forewing (13 components) and hindwing (14 components) datasets. It is clear from the histograms of the projected wing-shape positions on the single linear discriminant axis (Fig. 5) that, for this classic GM-style representation of *Trithemis* wing shape, broad zones of overlap exist between species that prefer open and forested habitats and among species that prefer to hunt over running, as opposed to temporary or standing water. This having been said, a clear distinction also exists between the wing morphologies found typically among these two opposing sets of ecological habitats. Tests of this distinction using a bootstrapped version of Hotelling’s multivariate extension of the two-sample *t*-test—to avoid interpretative constraints imposed by any failure of these data to meet the assumptions of the parametric *T*^2^ test—demonstrate that the shape differences shown in Fig. 5 are significant statistically at well beyond the standard p = 0.95 confidence level (see Additional files 2: Geometric Morphometrics (Landmarks) CVA results for forewing and hindwing datasets). Since wing shape variation in these *Trithemis* species cannot be interpreted to be an epiphenomenon of phylogenetic covariation (see above), the most reasonable interpretation is that forewing and hindwing shape differences result from morphological convergence on forms that represent airfoil designs suited for aerial hunting in these different environments. The fact that these habitat-related shape differences do not account for the major directions of wing shape variation present within the sample suggests that a variety of *Trithemis* forewing and hindwing shapes are viable functionally. But based on these results there is a strong, and statistically significant, indication that subtle, but definite, common wing-shape differences exist between forest and open habitat-dwelling, and between running and temporary/standing water-hunting, *Trithemis* species.

**Fig. 6:**
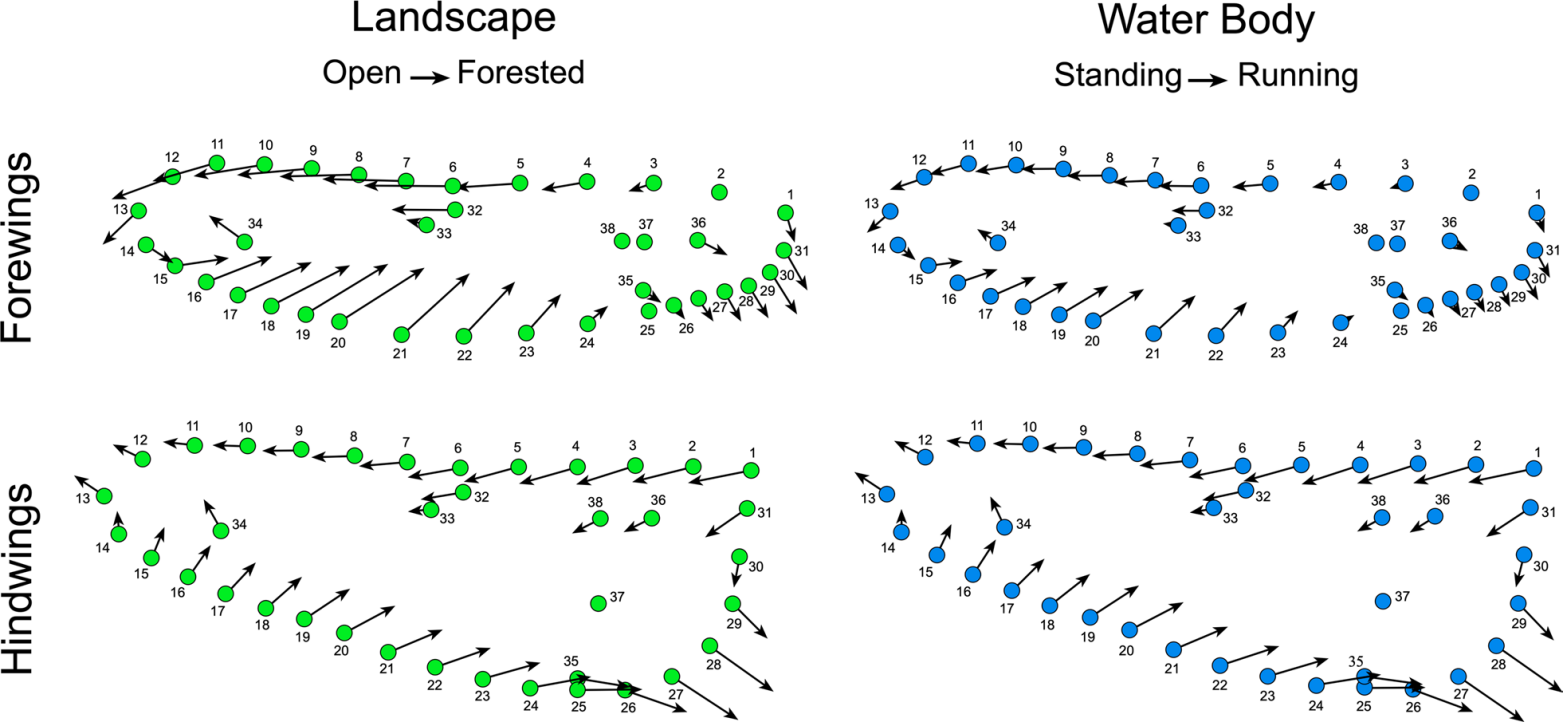
Vector displacement models for *Trithemis* habitat-category contrasts calculated from the linear discriminant analysis of forewing and hindwing datasets. For illustrative purposes displacement-vector lengths have been exaggerated by a factor of 4. Note similarity in the geometric character of habitat-group displacement patterns across the landscape and water body linear discriminant results

Which aspects of *Trithemis* wing morphology are responsible for these habitat-level distinctions? Shape configuration vector-displacement models (Fig. 6), based on the forewing and hindwing linear discriminant results, show that open landscape species, and species that hunt over running water bodies, exhibit slightly narrower forewings relative to forest-dwelling and standing water-hunting species. In both cases this narrowing is achieved via lateral, outboard migration of all costal landmarks and semilandmarks with the displacement magnitude reaching an acme in the middle of the interval between the nodus (semilandmark 6) and the wing apex close to the costal terminus (semilandmark 12) with these displacement vectors incorporating a subordinate posterior orientation in the vicinity of the wing apex. Displacements of the descending nodus vein-radius + media vein vertex landmark (32), R_3_ bifurcation vertex landmark (33) and distal IR_3_ landmark (34) also follow this general displacement pattern with the latter exhibiting a contrary subordinate anterior orientation. In contrast, landmarks and semilandmarks along the posterior wing margin exhibit inboard-anterior displacement vectors whose magnitudes culminate in the vicinity of the R_3_ vein terminus (semilandmark 20). These displacements have the effect not only of making the wings of open landscape and running water species narrower than those of forest-dwelling and temporary/standing water-species, but also more uniform in width. In addition, the proximal posterior wing margins of open landscape and running water species are more gently curved than forest-dwelling and temporary/standing water-species which exhibit a more sharply angled anal area or “proximate posterior corner”.

With regard to hindwing morphology, the landmark-semilandmark displacement trends are similar to those of the forewing in general, but with a few interesting differences. As with the *Trithemis* forewings, hindwings in species that inhabit forested landscapes and hunt over running waters are slightly narrower and have more pronounced anal area “corners” compared to species that inhabit open landscapes and hunt over temporary/standing waters. Also, as with the forewings, this shape transformation is accomplished via lateral outboard migration of the costal landmarks and semilandmarks (1–13), lateral inboard and anterior-ward migration of the distal and medial posterior margin landmarks and semilandmarks (14–24), and a pronounced lateral outboard and posterior migration of the posterior wing-margin landmarks and semilandmarks located in the wing’s anal area (25–29). Internal hindwing landmarks also mimic the displacements seen in their analogous forewing landmarks for the most part.

Nevertheless, differences exist in the orientation of hindwing displacement vectors at the extreme proximal and extreme distal ends of the wing body. Whereas landmarks and semilandmarks documenting the forms of the forewing’s apex (12–14) exhibit a subordinate posterior migration, in the forewing, these same points exhibit a distinct-but-subordinate anterior migration in the hindwing. Similarly, whereas the forewing anterior wing-attachment landmarks (1 and 31) and closely associated semilandmark (30) display a dominant posterior-inboard migration in concert with the anal-area semilandmarks, in the hindwing these landmarks display a pronounced anterior migration in opposition to the anal-area semilandmarks. This transformation further accentuates the proximal width of *Trithemis* hindwings in those species that inhabit forested landscapes and hunt over running waters.

**Fig. 7:**
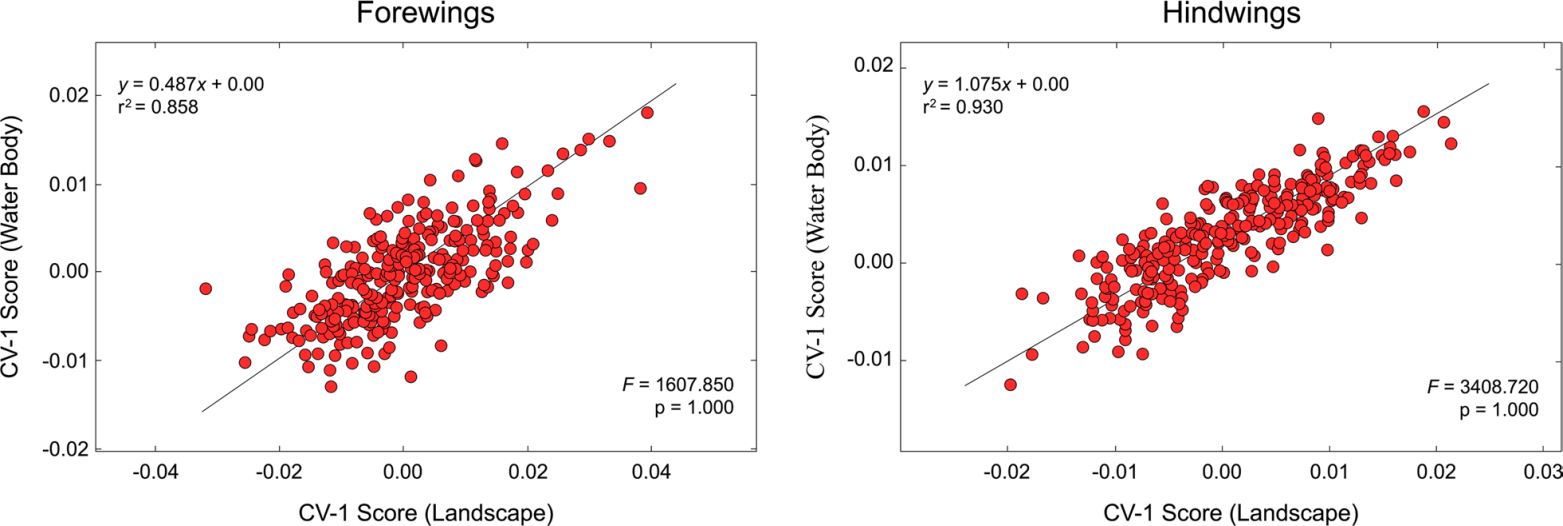
Standardized major axis linear regression analyses of linear discriminant scores calculated for landscape and water body habitat differences for *Trithemis* forewing and hindwing landmark-semilandmark datasets. Although there is a broad range of scatter in these data and the species assigned to these different habitat categories differ somewhat (see Fig. 3), a statistically significant linear trend does exist for both datasets. Accordingly, the null hypothesis that selection for wing shape differences operated in a different manner for landscape and water body aspects of the environment can be rejected

The consistency of the CVA results displayed in Figs. 5 and 6 across habitat categories raises the question of whether these habitat-based distinctions in wing shape represent separate, or conjugate, aspects of selection on these dragonfly species. This determination cannot be made from the frequency histograms, but instead requires a linear regression analysis (Fig. 7) to determine the level of similarity between landscape and water-body linear discriminant analysis scores for both forewing and hindwing analyses.

The null hypothesis for these regression tests is that no linear relation exists between the projections of wing shape in discriminant spaces optimized for landscape and water-body aspects of the *Trithemis* habitat. In evolutionary terms, this null hypothesis amounts to a statement that selection on *Trithemis* wing shape operated differently in its attempt to modify forewing and hindwing shapes for optimal function in forested, as opposed to open, landscapes and above running, as opposed to temporary/standing, waters. The scatterplots shown in Fig. 7 document an abundant degree of variation in the comparison of wing-shape scores in landscape and water body-optimized linear discriminant vectors. But these data also document a well-established and convincing linear trend in both *Trithemis* forewings and hindwings. Accordingly, it would seem that, despite the fact that differences are present in the species assigned to different landscape and water body character states (see Fig. 2), these differences are insufficient to overturn the hypothesis that, for this dataset, a pronounced, and statistically significant, correspondence exists between species inhabiting forested landscapes and running water bodies and between species inhabiting open landscapes and temporary/standing water bodies, in terms of their patterns of forewing and hindwing shape variation.

Failure to identify a clear distinction between these two habitat categories is, perhaps not surprising in that it may be a by-product of the *Trithemis* samples included in this investigation. All samples came from museum collections where, it can be assumed, priority was given to collecting specimens that represent the species in question, but not necessarily the range of habitats in which members of that species might occur. Species-specific sample sizes are also modest relative to most ecomorphological investigations (see Methods). Be this as it may, it is undeniably the case that just under 75 percent of our *Trithemis* species which occur typically in “forested” habitats have also been assigned by [49] to the “running” water-body category and those occurring typically in “open” habitats to the “temporary/standing” water-body category. To the extent that these assignments are correct and accurate, our finding that no wing shape distinction exists between these habitat-pair combinations may be a fully justified overall reflection of *Trithemis* biology. Of course, this result is also tied to the taxonomic composition of our *Trithemis* sample and the manner in which our landmark-semilandmark data represent *Trithemis* wing morphology. As such, we a regard this result as an accurate description of our sample, but an interpretation of *Trithemis* ecomorphology that is provisional, pending more focused ecomorphological investigation with larger intra-specific samples collected with this issue in mind.

Regarding the overall quality of the discriminant partition obtained, Table 1 summarizes the performance of these datasets for the purpose of discriminating between landscape and water body habitat groups. While previous results demonstrated that a GM-style approach to *Trithemis* wing-shape characterization was sufficient to document the existence of subtle, but consistent habitat-based shape differences, these data were, on the whole, unable to separate different species based on their landscape or water body habitat preferences with high degrees of accuracy. The degrees of apparent shape overlap between forest and open landscape species, and between running and standing water species, are simply too great to rely on either forewing or hindwing shape alone to be an accurate diagnostic indicator of these environmental preferences. The fact that identification accuracies as high as 66 to 80 percent were delivered by our leave-one-out jackknife analyses is, itself, quite noteworthy. We doubt accuracies this high could be delivered routinely by taxonomic experts for species identifications who had access only to whole-wing images, much less configuration plots of 38 mathematical point locations. Matthews correlation coefficient [51–53] values of less than 0.75, plus the time and technology required to collect even a single set of landmark-semilandmark coordinates, however, suggest that GM-style ecomorphological analyses may represent a suboptimal approach to the problem of ecomorphological group-discrimination problem in *Trithemis*, and perhaps in many other cases as well.

**Table 1:**
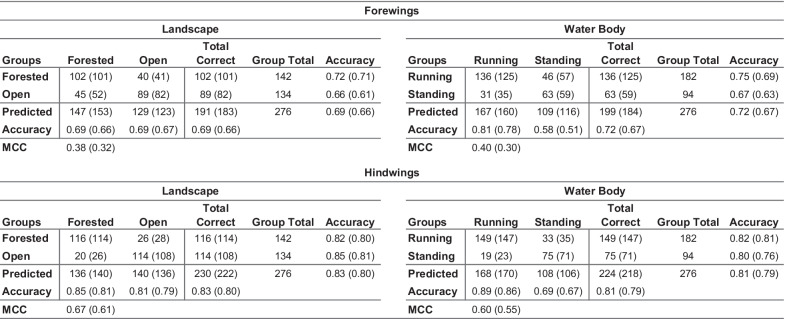
Confusion matrices for the *Trithemis* linear discriminant results obtained from the landmark-semilandmark dataset

Both raw, post hoc and leave-one-out jackknife (in parentheses) are shown. MCC: Matthews Correlation Coefficient for the confusion matrix as a whole

### Geometric morphometric (GM)-style analyses of image datasets

One of the inherent limitations of classic GM approaches to form analysis is that it is based on the collection of—and representation of morphologies by—relatively sparse sets of topologically homologous landmark and semilandmark point locations. In the Outomuro et al. analysis [47], this approach required wing-form estimates be based on intersections between major wing veins, and of these veins with the wing periphery. But *Trithemis* wings are obviously composed of far more—and considerably more complex—structural elements than those represented either by our GM-style form-representation system or by the system employed in the Outomuro et al. investigation [47]. In order to determine whether a more comprehensive representation of wing morphology might improve the ability to detect, document, and model differences in *Trithemis* habitat guilds, we conducted a parallel GM-style data analysis in which wing images—as represented by grey-scale brightness values for all pixels in standardized 200 × 56-pixel (forewings) and 200 × 81-pixel (hindwings) image frames—were substituted for the shape-coordinate datafiles (Additional file 1: *Trithemis* Wing Images Archive). The fact that the same data-analysis procedures were employed in both the landmark-semilandmark and image-based sets renders the results obtained comparable despite slightly reduced datasets being employed for the wing-image analyses. Perhaps even more importantly, use of segmented whole-wing images avoided the need to select any aspect of the wing morphology for investigation at the outset of the analysis as well as ensuring that those aspects of the morphology which could not be represented by single-coordinate, or single-pixel, locations (e.g., color or shading patterns) were included along with those aspects that could.

**Fig. 8:**
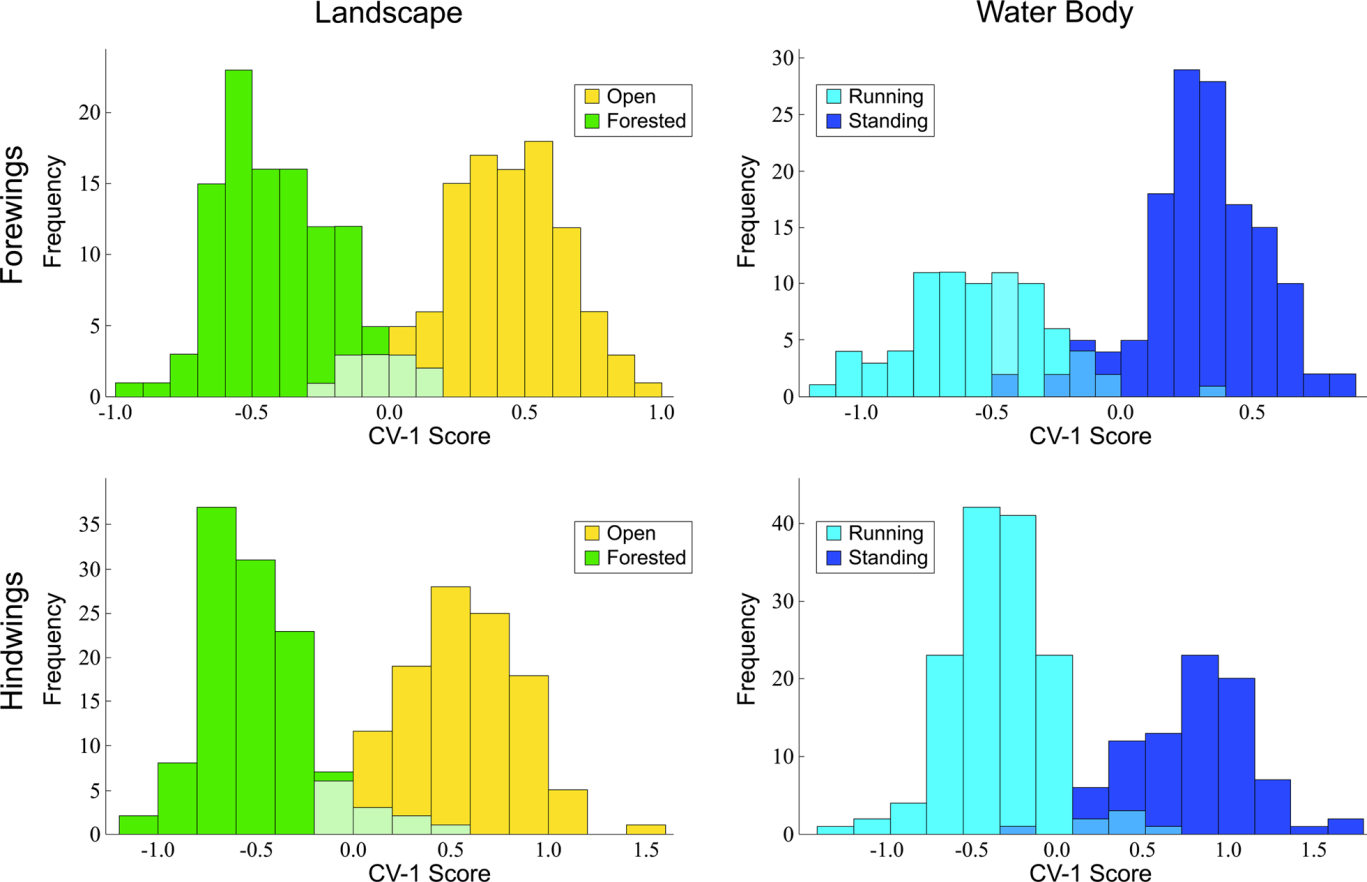
Frequency histograms for *Trithemis* forewing and hindwing shape distinctions for species preferring different landscape and water body habitats based on the direct assessment of wing-image datasets. Note the minimum extent of shape-range overlap and clear distinction exists between the central tendencies of these habitat-based wing shape differences. Statistical comparisons of the between-groups separation relative to within-groups dispersion using a bootstrapped version of Hotelling's T^2^ test indicate that all four comparisons are significant at well beyond the p = 0.95 confidence level

Results obtained from the PCA-CVA analysis of wing-image pixel values for those *Trithemis* images that did not include representations of the specimen labels (Fig. 8), see also Additional file 2: Geometric Morphometrics (Images) results for forewing and hindwing datasets show that, despite the minor differences in the sample composition between the two analyses (a difference that would favor lower discrimination power), use of images instead of a landmark-semilandmark-based representations of wing form resulted in a marked improvement in between-group separations for each wing morphology and for each habitat contrast. Some might be tempted to interpret this improvement to reflect the well-know tendency for high-dimensional datasets to yield artificially large apparent group distinctions when subjected to linear-discriminant analysis owing to the sparse distribution of data points in high-dimensional mathematical spaces (see [44, 54]). If this was the correct interpretation of our results this should be revealed by a bootstrap analysis of between-group separation relative to within-group dispersion via any of a number of statistical test indices (see [45, 46]). However, when this experiment was carried out using the well-known Hotelling’s T^2^ test, for each of the four comparisons shown in Fig. 8, observed values of the *T*^2^ statistic fell well beyond the ranges of *T*^2^-value distributions obtained from 1000 random permutations of the data (see Additional file 2: Geometric Morphometrics (Images) CVA results for forewing and hindwing datasets). Based on these results it seems clear that the between-group separations shown in Fig. 8 cannot be interpreted as mere artifacts of variable number-sample size interactions, but rather reflect clear and consistent differences in the shapes of *Trithemis* wings for species that preferentially inhabit different landscape and water-body habitats. These results support interpretations offered for the previous GM-style landmark-semilandmark datasets and imply (1) that distinctions present between the wing morphologies of these different habitat groups include aspects not represented in the landmark-semilandmark dataset and perhaps aspects that cannot be represented under GM-style morphology sampling conventions and (2) reliance on GM-style morphology sampling conventions resulted in a substantial underrepresentation of the actual degree of difference between a priori-defined *Trithemis* habitat groups in terms of wing-morphology differences.

**Fig. 9:**
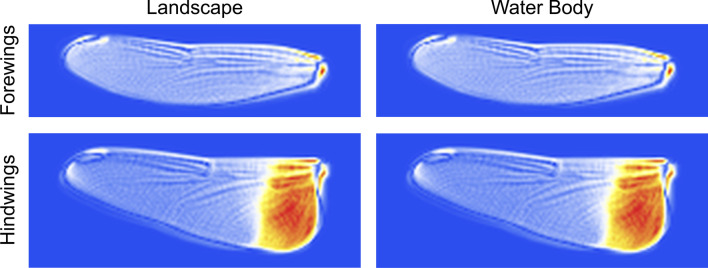
Color-coded comparisons of *Trithemis* forewing and hindwing mean shapes illustrating which wing pixels changed their brightness values least (blue) and most (red) along the linear discriminant vectors separating landscape (left) and water-body (right) species. As with the previous landmark-semilandmark results, the transitions from forested to open landscape habitats and temporary-standing and running water-body habitats involve essentially identical shape transitions, despite different species cohorts being placed into these habitat groups. Note that the forewing and hindwing areas exhibiting the greatest changes in pixel brightness values constitute either very small well specified regions (forewings) or large areas (hindwings) that were either not included in the landmark-semilandmark sampling scheme (forewings) or could not be so included owing to the fact that the wing character most consistent with between-group difference was a pigmentation region (hindwings)

What aspects of *Trithemis* wing morphology might be responsible for the greater between habitat-groups separations shown in Fig. 8? Fig. 9 illustrates color-coded comparisons of shape models for the end-member coordinate positions along the different habitat-group linear discriminant axes for both wing complexes. These shape models are consistent with the landmark-semilandmark displacement patterns (Fig. 6) for both *Trithemis* forewings and hindwings. But owing to their more complete coverage of wing morphology, contain much additional information about habitat-structured wing-shape differences.

With regard to the forewings, the dark blue line most evident in the lower right and upper left of the forewing shape models (but present around the entire wing) marks the periphery of the typical “open landscape” wing form. The solid white areas in the regions adjacent to this periphery signal that, in typical “forested landscape” *Trithemis* forewings, the distal portion of the wing has adopted a slightly more anteriority angled orientation and the proximal posterior margin a more posteriorly expanded orientation with a more prominently expressed anal-area “corner”. The forewing tip also appears less rounded (more asymmetrical) in typical forest-dwelling species. The arrangement of both the internal major wing veins and the polygonal cells formed by the minor wing veins, also appears to exhibit consistently well-structured displacements that reflect the wing-margin alterations.

In other words, these results suggest no major reorganization of the internal *Trithemis* forewing structure is part of the distinction either between open or forested landscape-dwelling species or between running or temporary/standing water body-dwelling species. This too is consistent with results of the forewing landmark-semilandmark displacement analysis results (Fig. 6). But whereas that previous analysis only sampled seven discrete internal wing landmark locations, these image-based data facilitate a remarkably detailed examination of internal structures across the entire forewing at a level of morphological resolution that simply could not be matched by landmark-semilandmark sampling schemes without engaging a very laborious and time-consuming data-collection programs.

These differences having been noted, we hasten to point out that variations in the forewing peripheral outline, along with the apparently passive general displacement of major wing textural elements, are *not* the most important morphological distinctions that characterize these different *Trithemis* habitat guilds and serve to distinguish them from one another. As can be seen clearly in both forewing model comparisons, the largest distinctions between these habitat-guild pairs occurs in the extreme proximal portion of the forewings, close to the attachment between the wing and the body. The most prominent of these differences involves the posterior wing attachment which is composed of a complex array of morphological structures (e.g., distal plate, proximal plate, axillary plates I–III).

The reason this region plays such a distinctive role in between-habitat group distinction appears to be linked to pronounced changes in the form of the extreme proximal posterior forewing outline which forced this complex of elements to migrate inboard and posteriorly to accommodate and support the proximally wider forewing typical of forest-dwelling and running water-hunting *Trithemis* species. Resolution in this area of the wing morphology was lost in the case of the landmark-semilandmark dataset because the posterior wing attachment was represented by only a single landmark (31, see Fig. 1B). This landmark did exhibit a large displacement relative to surrounding landmarks in the extreme proximal region of the wing (see Fig. 6), but the overall morphological complexity of this attachment was underestimated by the decision to represent it with only a single landmark location. The desire to spread landmarks as evenly as possible over the morphological in question often results in complex, and perhaps disadvantageous, decisions having to be made regarding how to represent intricate  morphologies under classic GM-style landmark-based sampling systems; decisions whose implications are difficult to appreciate when no alternative strategy for wing-shape sampling available. The higher, more comprehensive, and much easier-to-collect morphological data available via the use of image pixels as morphological variables facilitates the complete and comprehensive analysis of all available morphological information encoded in these 2D wing structures. In addition, and as these results demonstrate, patterns of highly localized (and so taxonomically informative) differences in comparative morphology can result from image-based analyses.

Along with the posterior wing-body attachment complex, our linear discriminant model-based comparison of between open and forested landscape-dwelling species, and between running and temporary/standing water body-dwelling species, also identified the proximate limbs of the costa, subcosta and radius + media veins as important sites of distinction between these habitat groups. In the effort to ensure even landmark-semilandmark placement across the entire forewing form, and because of difficulties in defining a landmark point to represent the form of a laterally extensive wing vein, these sites were also not represented by any landmarks in our classic GM-style analysis or in those of [47].

Much the same interpretation can be offered for the hindwing image dataset (Fig. 9), but with a rather obvious addition in hindsight. Relative to the typical hindwings of open landscape and temporary/standing water body-dwelling species, typical forested landscape and running water body-dwelling species possess hindwings that are more distally elongate, with greater asymmetry at the wing apex as well as being proximally wider. The angle of the peripheral hindwing is slightly greater in the latter groups, but not as much so as in the forewings. Overall, the hindwing textural elements appear to have responded more-or-less passively to changes in peripheral outline shape, though there is some suggestion that the pterostigma has shifted to a position slightly more proximal along the wing’s anterior margin than would be expected as a result solely of the increase in distal hindwing angularity. Nevertheless, these groups possess proximally narrower hindwings. Also, similar to the forewings, both the anterior and posterior wing attachments, as well as the proximal costa, subcosta and radius + media veins along with (in the hindwings), the proximal cubitus vein exhibit pronounced variations along the transition from forested landscape and running water-dwelling species to open landscape and temporary/standing water-dwelling species.

Once again, as no landmarks were placed in these areas of the hindwing morphology, these aspects of hindwing variation were invisible to the previous landmark-semilandmark-based analysis. However, by far the most prominent aspect of hindwing form difference is the presence of the medium-to dark pigmented region proximal to the body characteristic of such temporary/standing water body-dwelling species and such open landscape-dwelling species as *T. annulata*, *T. arteriosa*, *T. aurora*, *T. kalula*, *T. kirbyi* and *T. monardi*. While it is true that *T. tropicana* exhibits the darkest proximal color bands of all species examined here (see Additional file 1: Trithemis Wing Images Archive), this is an atypical condition for the majority of forest and running water-dwelling species in our study sample (e.g., *T. dichroa*, *T. dorsalis*, *T. ellenbekii*, *T. nuptalis*). As a result, prominent proximal hindwing pigmentation was not considered by the linear analysis to be the typical state for these groups as a whole, it is clearly, but non-exclusively, common among temporary/standing water body-dwelling species and such open landscape-dwelling species.

The degree to which these image-based, linear discriminant analyses of *Trithemis* forewing and hindwing images were successful in identifying landscape and water-body habitat groups is summarized in the confusion matrices presented in Table 2. Comparing these with analogous results obtained from the GM-style landmark-semilandmark datasets (Table 1) shows how dramatic the difference can be between the amount of ecomorphologically important information represented by these different data types. For a post-hoc analysis of the training datasets, group-identification accuracies for the forewing landmark-semilandmark datasets ranged from 0.69 and 0.72. These accuracy values rose to between 0.93 and 0.94 for the image-based dataset. Similarly, the hindwing analysis group-identification accuracies ranged from 0.81 and 0.83 for the landmark-semilandmark datasets, but rose to values between 0.94 and 0.97 for the image-based dataset.

**Table 2:**
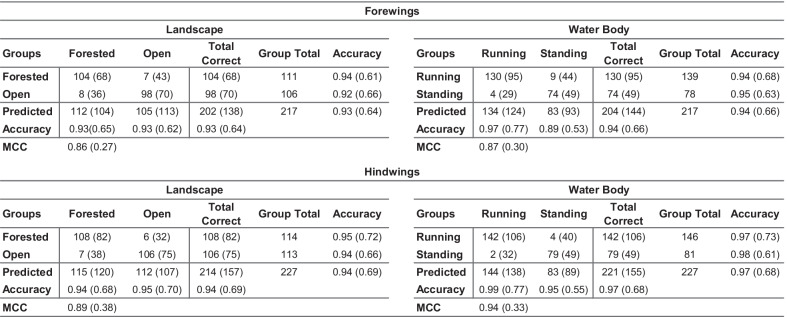
Confusion matrices for the *Trithemis* linear discriminant results obtained from the image datasets

Both raw, post hoc and leave-one-out jackknife (in parentheses) are shown. MCC: Matthews Correlation Coefficient for the confusion matrix as a whole

The fact that such high accuracy values can be achieved based on wing morphology alone was unexpected and seems remarkable. However, our enthusiasm for these results is tempered by the results revealed in the (leave-one-out) jackknife analysis of discriminant-function stability. While unexpectedly high and quite respectable accuracies were achieved by the image datasets under this constraint (ranging from 0.64 to 0.66 for forewings and 0.68 to 0.69 for hindwings), these accuracy values are comparable those achieved for the landmark-semilandmark dataset for the *Trithemis* forewings and inferior to those achieved by the landmark-semilandmark dataset for the hindwings. This result in no way compromises the statistical validity of the results achieved by the image-based linear discriminant analysis for the individuals analyzed in this investigation since the bootstrap version of Hotelling's T^2^ test takes the possibility of overtraining into consideration explicitly. Nevertheless, we would not advocate use of these image-based forewing or hindwing discriminant functions as a basis for making any critical habitat-based identifications. This fall-off in performance stability was most likely caused by dramatically higher dimensionality of the image datasets and consequent need (ideally) to increase the image-dataset size dramatically in order to offset problems arising from sample size-dimensionality interactions. In addition, it may be the case that the somewhat poor stability results reported for both the landmark-semilandmark and image-based datasets arose not only, and not exclusively, from sample-size issues but, indeed, from the wisdom of assuming that geometric distinctions between these different habitat groups can be modeled accurately via the use of linear approaches to morphological data analysis.

### Machine-learning (ML) analyses of image datasets

In order to address the issue of suboptimality induced by the linear modelling of ecomorphological group discrimination, one further set of analyses were undertaken for the *Trithemis* datasets based on use of the deep learning LetNet-5 CNN architecture employing the embedded image-contrast sampling protocol. In the case of the forewing analysis a total of 37,671 image contrasts, included both within and between habitat-group comparisons, were used to train the system to discriminate between the wing morphologies of landscape and water body species groups. The complete training sequence consisted of 1767 iterations of 64-image contrasts each (= a batch) with each set of iterations being regarded as a single training round (= an epoch). Training proceeded over three rounds with each round consisting of a total of 113,088 randomly drawn training-image contrasts. The order of these image contrasts was shuffled randomly between rounds and training allowed to proceed across the entire three-round (or epoch) cycle. Thus, training was based on the consideration of 339,264 pairwise image comparisons despite the fact that only 37,671 unique image contrasts, based on a total sample of 217 images, were employed. Similarly, the hindwing analysis employed a total of 41,223 image contrasts organized into three training rounds (or epochs) of 1935 iterations of 64-image batches. The hindwing training sequence, then, employed a total of 371,520 unique image contrasts based on a total sample of 227 images. In both cases convergence was achieved with post-training error-loss values of 2.38 × 10^–5^ and 1.00 × 10^–4^ for the landscape and water-body group analyses respectively.

**Fig. 10:**
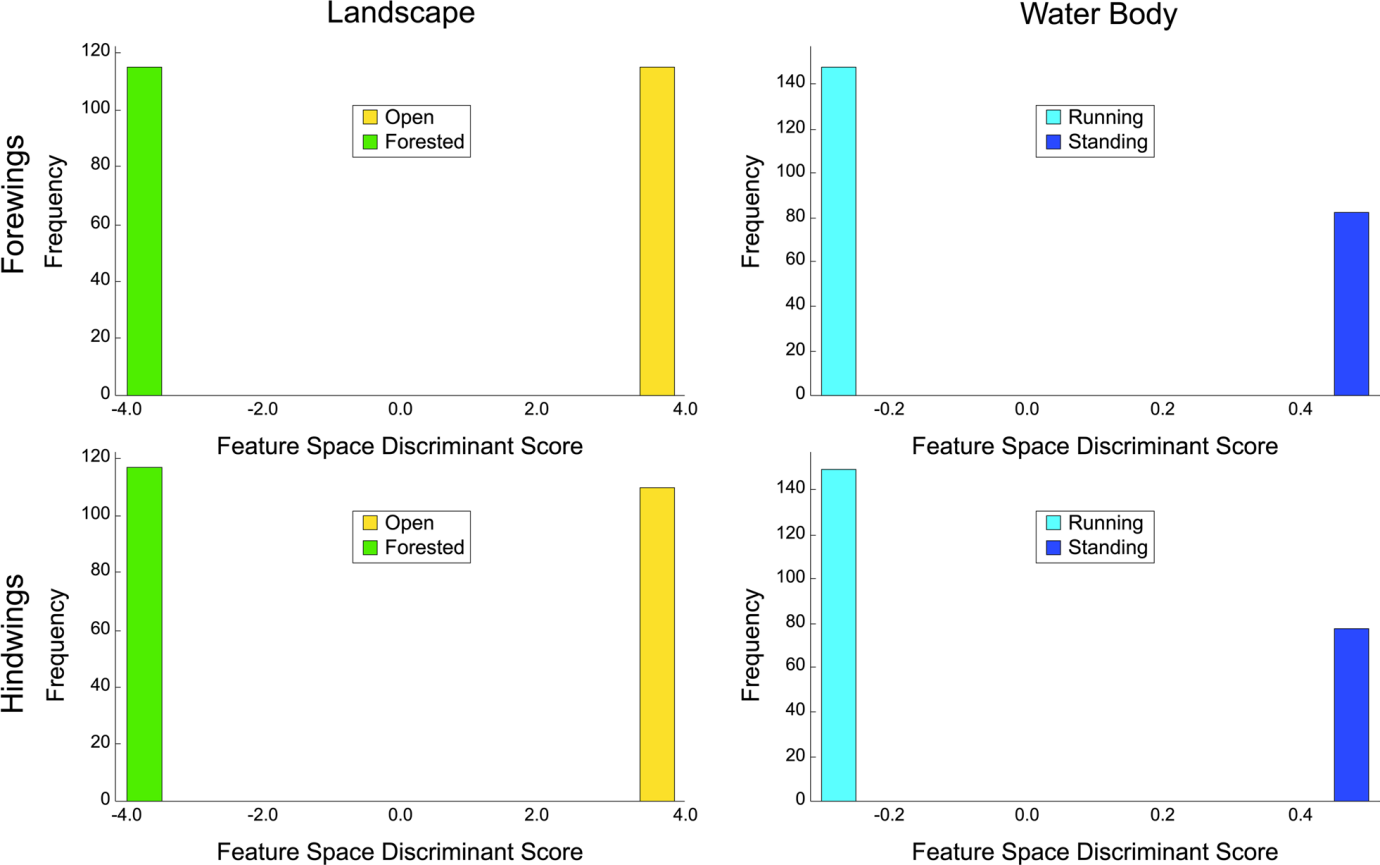
Frequency histograms for *Trithemis* forewing and hindwing shape distinctions for species preferring different landscape and water body habitats as assessed from the wing-image datasets using the embedded LeNet-5 deep-learning algorithm. Note the complete, and quite marked, separation that was achieved for these habitat-based wing morphology differences in the case of both forewings and hindwings. Comparison of these results with those of the linear-model analyses (Figs. 7 and 10) suggests that the geometries of morphological differences between these habitat groups exhibit a strongly non-linear character

Results of the LeNet-5 “deep-learning” analysis of wing-shape differences between landscape and water-body guilds for *Trithemis* forewing and hindwing images (Fig. 10) show that, in all four cases, the LeNet-based ML architecture was able to identify morphological features characteristic of these different ecological groups with 100 percent accuracy. Indeed, the degree of between-groups separation achieved—which is indicative of the statistical confidence the algorithm has in its results—was such that within-group discriminant-score variation (of which there is a bit), has been obscured totally by selecting the same number of histogram bins used to illustrate the discriminant results of previous analyses.

Note that this exercise is not quite the same as asking the trained system to identify the images on which it was trained insofar as the system was not trained on the raw wing images, but rather on distance-based estimates of contrasts between pairs of wing images both within and across *Trithemis* habitat-guild groups. This embedded training design assisted the CNN system in its task of focusing training on those aspects of morphological variation most closely associated either with group membership (= contrasts between images belonging to the same genetic species) or group distinction (= contrasts between images belonging to different genetic species). Once trained though, the system can be employed to identify raw wing images. Consequently, it is informative to use the set of raw training images to evaluate trained-system performance despite it being true that these same images participated in system training in the sense that they provided the basis for the contrasts on which system was trained.

As is always the case with the analysis of high-dimensional data, the results shown in Fig. 10 might be regarded as suspect; the result of an overtrained learning system. Two arguments can be cited against this interpretation. The first involves the actually dimensionality of the data submitted to the LeNet-5 system for analysis. The first step of the LeNet-5 procedure is to process the raw images into 40 × 40-pixel thumbnail images. This operation reduced the original image sizes (200 × 56 pixels for the forewings and 200 × 81 pixels for the hindwings) to a standard of 1600 pixel values. Naturally, some morphological detail was lost during this interpolation process. Nevertheless, the dimensionality of the input data was reduced by 86 percent and 90 percent for the forewings and hindwings respectively. While this dimensionality remains quite high by GM standards, its effect was offset by employing an embedded, group-contrast ML approach, which focuses not on the number of raw images in the datasets, but on the number of image contrasts inherent in the raw image sets. Thus, the effective sample sizes used to train the LetNet-5 system (37,671 and 41,223 pairwise comparisons for the forewing and hindwing datasets respectively) were c. 25 times greater than the dimensionality of the data being discriminated. Therefore, there is little reason to suspect to suspect that system overtraining might arise in these analyses as a consequence of the curse of dimensionality.

In a more practical sense, however, empirical evidence of the stability, and appropriateness, of the results shown in Fig. 10 can be generated using a standard leave-one-out jackknife strategy. Owing to the amount of time required to train the LeNet-5 system on a GPU-enabled platform (c. 1 h per total training cycle), it was considered impractical to submit the entire forewing and hindwing image datasets to the jackknife procedure. Instead, a series of 25 randomly chosen images were selected from each dataset and the jackknife procedure carried out on these 25 target images in order to estimate of the stability of each discriminant result. This selection was carried out independently for each of the habitat-guild group analyses and for each of the forewing and hindwing datasets to ensure each estimated stability result was independent of the subsample selected for jackknife sequestration.

Table 3 summarizes results of these four jackknife analyses for the *Trithemis* habitat-guild datasets. In each case the LeNet-5 systems, trained with the entire forewing and hindwing datasets minus the sequestered specimen images successively, were able to identify the sequestered specimens with 100 percent accuracy overall. These results indicate that discriminant analyses of each system calculated from the *Trithemis* hindwing and forewing datasets, partitioned either by landscape or water-body habitat groupings, exhibit compelling stability with no evidence of any identification inaccuracies that could indicate substantial—or indeed any—overtraining. Further, these results not only agree with our previous morphometric results in suggesting consistent and stable morphological differences exist in the wing morphologies of *Trithemis* species that inhabit open and forested landscapes, and that hunt above temporary/standing or running waters, they imply that these differences are more substantial, more consistent and more stable than was indicated by either the standard GM-style linear analyses of landmark-semilandmark data or the linear analysis of image pixel data. This result also suggests that these differences may be distributed in a non-linear manner in the wing shape space this making them even more difficult to identify via simple visual inspection. Finally, since the species cohorts assigned to the landscape and water-body groups show some, but not total, identity (see Fig. 2), these results also appear to indicate that different sets of morphological features are responsible for these landscape and water-body ecomorphological distinctions.

**Table 3:**
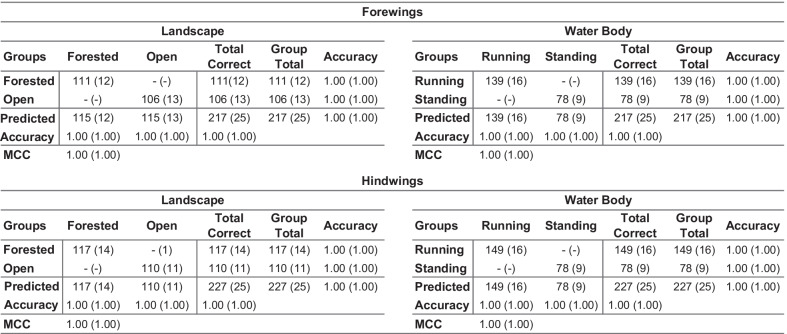
Confusion matrices for the *Trithemis* LeNet-5 deep-learning discriminant results obtained from the image datasets

Both raw, post hoc and leave-one-out jackknife (in parentheses) are shown. MCC: Matthews Correlation Coefficient for the confusion matrix as a whole

## Discussion

### Phylogenetic covariance

Damm et al. [49] reconstructed ancestral character states for *Trithemis* landscape and water body habitat characters using the stochastic analysis method of Bollback [55]. While it was not possible to infer all ancestral character states with complete certainty, these results confirm that the transition from inhabiting open landscapes to forested landscapes, and from hunting above temporary/standing waters to flowing waters, arose multiple times in *Trithemis*. Our results, coupled with those of Damm et al. [49] suggest that, on each of those occasions, morphological changes in the phenotype were much more extensive than had been realized previously. These appear to have involved not only those well-studied characteristics known to be important for species identification, but also features—such as wing morphology—whose innate complexity had prevented their detailed analysis to date. In addition, our results suggest that common sets of morphological modifications characterize species that occupy both ancestral and derived ecological zones. Of course, it is also the case that each of these derived ecological radiations incorporates morphological variations unique to that species and/or to that radiation. Nonetheless, any species-specific or radiation-specific morphological trends in wing morphology were not of sufficient scope to obscure the subtle, but consistent, wing-form differences that characterize these ecological guilds.

Outomuro et al. [47] employed the method described by Klingenberg and Gidaszewski [56] to assess the strength of the phylogenetic signal of their wing landmark-semilandmark data. We employed the multivariate extension of the *K*-statistic method described by Adams ([48], see also [8]). The Klingenberg and Gidaszewski method [56] fits phenotypic data to the cladogram using squared change parsimony and then obtains an estimate of the phylogenetic signal by summing the squared trait changes across all branches. Under this scenario the smaller the sum the greater the conformance with phylogeny. Nonetheless, as Adams [48] points out, this method relies on ancestral-state reconstruction which usually involves high levels of uncertainty (see also [57]) and is unsuitable for use in evaluating phenotypic traits owing the fact that geometric scaling is not taken into consideration and changes systematically as trait variation among species and/or with the number of traits increases. The Klingenberg and Gidaszewski method [56] also incorporates a matrix inversion that limits its utility with datasets composed of large numbers of phenotypical characteristics and comparatively small numbers of species. Adams’ multivariate *K*-statistic approach [48] circumvents these limitations and is designed specifically for use with high-dimensional phenotypic data.

Our phylogenetic covariation results are further supported by our calculation of the PC-based phylomorphospaces for both *Trithemis* species and their reconstructed ancestors based on the pruned Damm et al. ultrametric tree ([49], Fig. 2). As illustrated by [48], phylomorphospaces calculated from morphometric data that exhibit a strong covariance with phylogeny exhibit an organization in which closely-related species, along with their hypothetical ancestors, are grouped together in different regions of the Procrustes PC space with few tree branches crossing one another. Our *Trithemis* phylomorphospace results (Fig. 4) exhibit a pattern of morphospace distribution that is the opposite of this expectation. Based on classic-GM analysis results, closely related *Trithemis* species projected to positions in vastly different parts of the shape space and many inferred tree branches cross. Moreover, given the expectation that estimated ancestral wing morphologies will tend to occupy regions closer to the origin of the Procrustes PC space than the terminal taxa, the range of shape variation displayed our GM data is such that it is difficult for us to imagine any configuration of inferred hypothetical ancestral wing shapes that would be consistent with the expectations of a strongly supported phylogenetic covariation pattern. This ordination geometry indicates that our results, and our interpretations of those results, are robust to any reasonable level of imprecision in ancestral node morphology inferences.

The finding that many biological datasets do not exhibit significant patterns of phylogenetic covariation is well established—including for morphometric datasets—and can arise for different reasons (see [57] and references therein). Notwithstanding the results reported by Outomuro et al. [47], *Trithemis* forewing and hindwing morphology appears to fall into this broad category. Our phylogenetic-signal results are also consistent with our finding of substantial ecomorphological covariation in *Trithemis* forewing and hindwing morphology given the fact that mappings of both landscape and water body characteristics of these species are distributed across the *Trithemis* cladogram. [Note: Outomuro et al. [47] report using their own estimate of *Trithemis* phylogenetic relations, but note that theirs was “similar to that previously published by Damm et al. (2010)” (p. 1868).]

### Morphology sampling strategy

The discrepancies evident between the morphometric results reported by Outomuro et al. [47] and ours are likely down to multiple differences between the two investigations. Note that, in a sense, this means both sets of results are correct representations of patterns inherent in the data that were collected during each investigation. Nonetheless, we regard these differences as being down, mostly, to differences in way the *Trithemis* wings were sampled.

Owing to our failure to document a significant phylogenetic signal in either our *Trithemis* forewing or hindwing data, we did not follow Outomuro et al. [47] and subject any of our datasets to phylogenetic least-squares “correction”. This operation removes substantial amounts of information from the data and can only be justified when there is a clear data analysis-based concern with overall data independence.

Outomuro et al. [47] found significant differences among forest and open landscape-dwelling species for males, but not females. Sexual dimorphism was not a target of our study, but our dataset was, on the whole, balanced in terms of the representation of male and female morphologies (44% females, 38% males, 14% uncertain) with all species being represented by individuals from both sexes. Accordingly, our findings of significant wing-morphology differences between landscape and water body groups imply that they pertain equally to both males and females, which is the more usual and expected pattern.

For our GM-style dataset, the difference between our habitat group findings and those of Outomuro et al. [47] likely result the level of completeness, evenness and specificity in the sampling of wing morphology. The Outomuro et al. [47] sampling scheme focused in wing apex region and the anterior margin. Our sampling scheme achieved a much more even coverage of all parts of the peripheral outline. More importantly, the extended eigenshape sampling protocol we used to determine how the wing periphery should be sampled automatically places more semilandmark points in those regions that exhibit the greatest shape variation across the dataset as a whole [58]. This had the effect of weighting our morphometric analysis toward those regions that exhibit the greatest amount of shape variation, thus ensuring appropriate advantage was taken of the information contained in those regions. No equivalent effort to focus the landmark-semilandmark data collected from *Trithemis* wings was employed in the Outomuro et al. study [47].

Our summary of the distribution of shape differences among the different habitat groups (Fig. 6) indicated that the wing periphery regions with the largest landmark/semilandmark displacements differed for the forewings and hindwings. In the case of the former the largest landmark/semilandmark point displacements occurred mid-wing along the posterior, or trailing, margin, along the distal anterior margin, and along the proximal posterior margin, especially very close to the posterior wing attachment. These regions were weakly and unevenly sampled in the Outomuro et al. investigation [47]. Similarly, in the case of the hindwings, the largest displacements occurred along the proximal posterior margin, especially close to the point of maximum wing-periphery curvature (= the prominent proximate posterior “corner”), followed by the proximate anterior wing margin and the posterior mid-wing margin. Again, these are areas where the Outomuro et al. scheme [47] obtained few, and unevenly distributed, samples of morphological variation.

Aside from the implications our results have for understanding the ecomorphology of *Trithemis*, the discrepancy between the results we and Outomuro et al. [47] obtained with our landmark-semilandmark GM analyses make a larger and more general point. The results of any morphometric investigation are determined completely by the sample that is obtained. What you sample determines what results you get. Morphometric representations of biological forms, especially those sampled by sparse sets of landmark-semilandmark points, cannot, should not, and must not be mistaken for the morphologies of the individuals or species themselves and the results generated therefrom pertain only to those aspects of the morphology that were sampled, not to the overall morphology itself (see also [59] where this issue was problematic). This distinction should be kept in mind, especially if negative results are obtained from any morphometric hypothesis test.

There are, of course, many ways to represent any complex morphological structure. All systematists, and all morphometricians, strive to inspect or obtain adequate and accurate representations of the morphologies or structures they investigate. In some cases, and for some structures, this is straightforward. In others it is exceedingly difficult, especially at the outset of a new investigation when little is known about patterns of variation anywhere in the forms or structures of interest. If the question under examination is specific and tied intrinsically to an explicit aspect of the morphology in question (e.g., Are the forewings of male *Trithemis annulata* longer than those of females of the same species?) the data relevant to the hypothesis test can be obvious. But if the question under examination is non-specific and not tied intrinsically to any particular aspect of the morphology in question (e.g., Do the forewings of *Trithemis* species that inhabit forested landscapes differ in some way from those that inhabit open landscapes?) it often is difficult to know what to compare, what data to collect from those aspects of the morphology judged suitable for comparison, and/or how to interpret the results of data-analysis procedures *in terms of the original question(s) of interest*.

In attempting to address this more difficult question, Outomuro et al. [47] chose a wing morphology sampling scheme that achieved a representation of *Trithemis* forewing and hindwing morphology, but did so in quite an uneven and approximate manner. Their approach reflected conventions that have grown up around GM which prioritizes the representation of complex structures thorough the digitization of small sets of independently defined landmark points. Originally, some GM practitioners even objected to the collection and use of boundary outline semilandmarks (e.g., [12, 60]) though these data are now collected and analysed routinely by GM practitioners, largely for practical reasons (e.g., [61]). But even given the (belated) acceptance of semilandmark points as useful means of sampling complex morphologies, we suspect few morphometricians would have been entirely comfortable with the sampling scheme devised by Outomuro et al. [47] as either a comprehensive, or geometrically accurate, representation of a *Trithemis* dragonfly wing. Their scheme quantified certain aspects of the wing morphology, but ignored the vast majority of the information available.

### Data analysis and statistical testing

Of equal importance to the issue of sampling adequacy is the understanding that different data-analysis procedures differ in the assumptions they make about the data that have been collected, the mathematical models they apply to those data, and the power those models have to reveal patterns of similarities and differences either within pooled datasets or (especially). This is especially important when the point of the analysis is to compare groups defined a priori.

The GM revolution was actually a synthesis between three aspects of morphometric practice that had been pursued more-or-less separately until the mid-1980s: (1) the representation of form through the use of sparse sets of topologically corresponding landmark-points (that served as the end-definitions of linear distances originally), (2) the alignment of these geometric point-locations through use of a least-squares Procrustes fitting algorithm, and (3) the representation of patterns of morphological variation via linear multivariate data analysis. While advances in addition to these did figure in the development of geometric morphometrics (e.g., centroid size, bending energy-based shape decomposition, graphic representation of shape deformation via use of thin-plate splines), and acknowledging that the GM synthesis has grown since its original formulation (e.g., admission of semilandmarks as a useful morphology-sampling convention), these three core aspects are those most often used and referred to in GM investigations. This synthesis was powerful, enabling morphological analysis to be pursued quantitatively and at levels of detail, coherence and interpretability unprecedented by the formerly separate schools of morphometric practice. Owing to that power, the geometric morphometric synthesis has proven to be highly effective in addressing a wide range of problems in systematic and comparative morphology, as well as being quite popular among the communities of biological, paleontological, systematics and evolutionary researchers. However, the GM approach, like all data-analysis approaches, has its weaknesses as well as its strengths. Perhaps even more importantly, the field of data analysis rarely remains static for long.

Over the last 20–25 years an alternative—some might say a rival—to GM has appeared in the form of ML. Unlike GM, ML approaches were not developed by researchers whose primary interest was in the analysis of biological morphology. Nevertheless, one of the primary, and most popular, uses of ML approaches, as well as one that spurs much ongoing ML research, has been the ability of these algorithms to find previously unsuspected patterns in all sorts of data, but especially in morphological data.

In many ways, ML represents a natural complement to GM. Whereas GM was designed to operate on a specific type of morphological data (= configurations of landmark point locations), ML can be used to analyze any sort of data. Thus, whereas the application of GM is limited to those situations in which forms can, reasonably, be represented by sparse configurations of point coordinates, ML opens the door to the consideration of a much wider range of morphological data and morphological problems. To date the overwhelming majority of GM analyses published in the biological, paleontological, systematic and evolutionary literature have been based on linear data-analysis models. However, ML approaches can be applied readily to situations in which the optimal models are non-linear, even if that is not known to be the case at the outset of an investigation.

At present, ML models are inferior to their GM counterparts in their ability to be queried and so used to identify which aspects of a set of morphologies are contributing disproportionately to the structure of sample variance. To be sure, attempts have been made to improve the interpretability of ML models (see [62] for a review). But owing to the level of specificity required of diagnostic morphological features that are useful in biological, paleontological, ecological, evolutionary and taxonomic contexts, coupled with spatial uncertainties as to where the boundaries of such features might lie, the relative sizes of the regions identified by currently available ML-interpretation algorithms are too large to be of much use in these contexts (see [63, 64] for examples). Machine-learning interpretation approaches can be useful in ensuring group-diagnostic image features belong to parts of the image that pertain directly to the specimens being imaged (e.g., as opposed to some aspect of the background). Beyond this, however, it appears we must await further developments in the field of ML interpretation before such algorithms can make substantial contributions to revealing the morphological features they are sensing in order deliver their superior group-characterization and group-identification capabilities. This having been said, the tools available for interpreting the results of GM analyses are also, by no means, straight-forward, easy to use nor exploited to their full potential by most practitioners.

Inevitably some will claim that GM is their preferred option for generalized morphological data analysis, either because, despite its limitations, they regard their study group(s) and research questions as being well-served by this approach and/or because they wish to retain a “geometric focus” in their analysis. In response we can only point out that all analyses of morphological data are “geometric” in character. From the results we have presented above it is unquestionably clear that the classic GM approach, when applied to the analysis of *Trithemis* wing-shape data, was the one that performed least well in finding, summarizing, and testing sets of characteristics that could be used to answer the question of whether shape variance was distributed among *Trithemis* landscape and water-body ecological guilds in a continuous or disjunct manner. What is also clear is that this comparative finding is neither an unusual, nor an exceptional, result (e.g., [45, 46, 59, 65–74]).

In terms of statistical testing, Outomuro et al. [47] relied on standard parametric statistical tests whose accuracies rely on assumptions regarding the form of data distributions and equivalence of variances among variables, all of which are rarely met by morphometric data. In contrast, our study employed bootstrapping and jackknife variants of standard statistical and data-analysis tests to ensure the results of our hypothesis tests were robust to violations of distributional assumptions.

## Conclusions

In seeking to understand the structure of the natural world it is important to appreciate the roles played by phylogenetic inertia—that an absence of divergent selection pressure can fail to produce substantial morphological divergence (hence “cryptic” species) even after speciation has taken place—and adaptive environmental diversification—that can lead to substantial morphological diversification even among sister species—in creating and maintaining that structure. Prior to the publication of Felsenstein [2], phylogeny was thought to contribute little more than anecdotal historical information to comparative biology and less still to the quantitative analysis of organismal morphology. However, following what can only be described as Felsenstein’s seminal description of the problems inherent in taking a non-phylogenetic approach to comparative biology, the systematic, biological and evolutionary research communities were converted, more-or-less rapidly, to the idea that "nothing in biology makes sense except in the light of phylogeny” ([75], p. 237; see also [57]). Today, the pendulum has swung decidedly in favor of a phylogenetically informed approach to comparative biology, to such an extent that “many comparative biologists [seem to] believe that phylogeny is not only necessary but also sufficient to answer any evolutionary question” ([57], p. 711). Has comparative biology’s late twentieth century course correction gone too far? Certainly, patterns of ancestry and descent are fundamental to the analysis of all biological data. But are there cases in which the structure of phylogenetic relations might provide little insight into understanding the morphological superstructure of the natural world; in which the demands of the environment may, indeed, have played the dominant role? Just as importantly, which are the best tools to use in determining whether the ranges of variation in complex biological structures are the same as, or different from, those same structures as manifested by another group; whether there is any pattern of distinction that requires explanation?

In our investigation of *Trithemis* forewing and hindwing morphology we set out to address these twin concerns of comparative morphology in an ecomorphological context using some of the most up-to-date, sophisticated and powerful tools available currently. With respect to the issue whether a statistically significant pattern of phylogenetic covariation exists in our sample of 276 mixed male and female individuals representing 27 *Trithemis* species (including species from across Africa, Madagascar, and China), analysis of GM-style landmark-semilandmark data using the multivariate extension of the *K*_mult_ statistic failed to detect any significant pattern of covariation between species-specific forewing or hindwing shapes. This result contrasted with the result obtained previously by Outomuro et al. [47] who used a different phylogenetic signal test, but is supported by the analytic superiority of the *K*_mult_ test and by PCA-based phylomorphospace results derived from the same dataset.

With respect to tests for ecomorphological differences in *Trithemis* forewing and hindwing morphology between landscape and water body ecological guild, linear discriminant analyses of classic GM-style landmark-semilandmark data, direct linear discriminant analyses of wing images, and an embedded image contrast-trained, “deep-learning” CNN analyses of wing images all detected statistically significant shape differences between both habitat-guild partitions for both wing complexes. The best between-groups separations were achieved by the embedded image contrast-trained CNN analyses of image data, the worst by linear discriminant analyses of classic GM-style data. These results suggest that shape differences between both habitat groups are not focused solely on the wing outline and are distributed geometrically in a non-linear manner. Our ecomorphological results also contrast, to some extent, with those reported previously by Outomuro et al. [47] who used a different, though GM-based, morphometric data collection and data-analysis strategy. Regardless, we believe our results are more representative of the actual situation in *Trithemis* because (1) a greater amount of wing-morphology information was included in our analyses and (2) we obtained consistent results from the analysis of radically different wing data sets and radically different data-analysis strategies. Our ecomorphological results are also consistent (3) with expectations of the mapping of these ecological habitat guilds onto the Damm et al. [49] *Trithemis* phylogeny.

In *Trithemis*, radiation from the ancestral ecological conditions of open landscapes and temporary/standing water bodies, into forested landscapes and running water bodies, occurred frequently and at multiple times in this genus’ evolutionary history. Traits that evolve frequently and substantially within taxa are usually responding to the differing needs of life under different selective regimes [57]. This principle is not sex-specific, and would be expected to apply equally to males and females, as our results indicate was the case of *Trithemis*. Moreover, highly functional aspects of the phenotype, such as wings, are subject to mechanistic, physical principles of optimization that are similar within similar environments, but differ across different environments. This is a very well-established principle in the comparative morphology of bird wings, bat wings and insect wings, including members of the Odonata (see above). This principle is also consistent with our finding of little phylogenetic covariation in *Trithemis* wing-shape data.

In raising criticisms regarding the adaptationist paradigm, Gould and Lewontin [1] viewed phylogenetic ancestry as exerting a constraint on morphological change and proposed that this hypothesis be considered a possible alternative to the direct, adaptive modification of each aspect of a species’ morphology, physiology, behavior, etc. to meet some environmentally mandated challenge. But as had been noted repeatedly by a number of researchers (e.g., [57, 76, 77]) phylogeny is not a constraint; rather, it is a pattern. The fact that two closely related species might exhibit similar morphologies cannot necessarily be attributed to the closeness of their phylogenetic relation any more than the fact that morphological differences between distantly related species can be attributed necessarily to their phylogenetic distance. In both cases it is a trivial exercise to cite numerous counter examples. Species remain close to, or diverge widely from, one another because of the manner in which they have responded to the challenges selection pressures have exerted upon them. These pressures need not cause every aspect of their morphologies to change and genetic, as well as mechanistic, linkages constrain the range of realizable options that exist for every species. Phylogenies are indispensable for understanding the structure of the living world. But phylogenetic patterns of ancestry and descent, by themselves, cannot provide an adequate process-level explanation for any aspect of that structure. Neither can associations between morphology and phylogeny be blithely regressed out of morphological datasets and dispensed with as though they were some sort of uninteresting nuisance factor. Instead, such associations should be regarded as constituting a category, or mode, of variation existing within morphological datasets that demands its own set of process-level explanations; separate from, but perhaps linked to, those explanations proposed for features not closely associated with phylogenetic patterns.

In addition to these considerations, it is important to note that the quality of any morphological analysis will depend critically on the data collected from particular sets of morphologies (e.g., structures, characters, species) and the tools used to discover patterns in those data; patterns that can be compared to the pattern of phylogenetic ancestry and descent as well as to aspects of variation in the natural environment. If mathematics can be regarded, as it is by many mathematicians, as the study of patterns in numbers [78], biology can be thought of as the search for patterns in the natural world [79]. Indeed, it is the existence of these patterns that provides not only the subject matter for biological study, but the evidence that deterministic processes or factors responsible for these patterns exist. If such patterns did not exist—if everything in nature simply graded continuously and insensibly into everything else—it would not only be impossible to conduct a truly scientific biological investigation, it would be pointless.

Mathematical data analysis and statistics (the two are *not* synonymous) are tools that, when used properly, can be employed to discover patterns in data that can aid biologists in their attempts to understand the living world. They are not, substitutions for, or means through which careful reasoning by researchers with specialist knowledge and experience can, or should, be overruled. Rather they can, are, and should, be used to aid and support biological reasoning by extending the powers of human senses and perception; by making patterns invisible to the unaided eye visible so they can be identified, discussed and interpreted.

In the same way as new and progressively more powerful statistical tools are being made available to find and compare patterns within morphological datasets and between morphological data and other sources of information, new tools have recently been made available for discovering patterns in biological data; ML being perhaps the latest and most intriguing example. As we hope we have demonstrated, the development of new and much more sophisticated ways of applying quantitative data-analysis procedures to the task of identifying patterns of variation in morphological data promises, at the very least, to invigorate the study of morphology; and perhaps also to revolutionize our appreciation for the amount of useful information that has been encoded in organismal morphologies of which, to this point, we have scarcely been aware.

## Methods

### Materials

*Trithemis* is a large genus (c. 50 species) of mainly African dragonflies referred to commonly as “dropwings” owing to their habit of holding their wings at a negative angle to their bodies, rather than horizontally, when at rest. In this investigation 27 *Trithemis* species (Table 4) were assessed, all of which were sourced from the insect collections of The Natural History Museum (London). Images of both forewings and hindwings from males and females of each species were collected using a low-magnification, digital SLR-based photo-microscopy system supplied by the museum. All images were taken from mounted specimens with a universal stage being employed to correct the orientation of each specimen prior to imaging so that the wing surface was normal to the microscope’s optic axis.

**Table 4:** *Trithemis* species, sample sizes and habitat assignments used in this investigation

	Sample Size (*n*)					
	Forewings		Hindwings			
Species	Landmarks	Images	Landmarks	Images	Landscape	Water Body
*Trithemis aenea*	4	4	4	4	Forested	Running
*Trithemis aequalis*	3	–	3	1	Open	Running
*Trithemis annulata*	20	12	20	13	Open	Standing
*Trithemis arteriosa*	10	10	10	10	Open	Standing
*Trithemis aurora*	10	10	10	10	Open	Standing
*Trithemis bredoi*	10	10	10	10	Forested	Running
*Trithemis dichroa*	16	12	16	8	Forested	Running
*Trithemis donaldsoni*	10	9	10	9	Open	Running
*Trithemis dorsalis*	10	1	10	6	Open	Running
*Trithemis ellenbekii*	11	4	11	6	Open	Running
*Trithemis festiva*	10	2	10	5	Open	Running
*Trithemis furva*	18	12	18	15	Open	Running
*Trithemis grouti*	10	9	10	10	Forested	Running
*Trithemis hecate*	8	7	8	8	Open	Standing
*Trithemis imitata*	10	7	10	3	Open	Standing
*Trithemis kalula*	10	10	10	10	Open	Standing
*Trithemis kirbyi*	19	15	19	17	Open	Standing
*Trithemis monardi*	9	9	9	9	Open	Standing
*Trithemis nigra*	10	10	10	10	Forested	Running
*Trithemis nuptualis*	10	6	10	5	Forested	Running
*Trithemis persephone*	5	5	5	5	Forested	Standing
*Trithemis pluvalis*	8	8	8	8	Open	Running
*Trithemis purinata*	10	10	10	10	Forested	Running
*Trithemis selika*	8	8	8	8	Open	Standing
*Trithemis stictica*	10	10	10	10	Open	Running
*Trithemis tropicana*	10	10	10	10	Forested	Running
*Trithemis weneri*	7	7	7	7	Open	Running
Total	276	217	276	227		

During photography every effort was made to block out the specimen label, which was impaled on the mounting pin beneath each specimen, by placing a white card over the label prior to image capture. In the case of some specimens, however, this operation could not be performed without risking damage to the specimen. Photomicrographs of these specimens were taken and used to assemble GM datasets where imperfections in the image were not relevant to the collection of landmark or semilandmark data. However, specimen images that included aspects of the label were excluded from the image dataset. Preferred landscape and water body assignments for these species were made according to the ecological information provided by [49] and [47], with the addition of data for *T. nigra* based on [80].

### Image processing

All forewing and hindwing images were segmented from dorsal view, whole-specimen images, mounted in a variably sized image frame against a flat white background, converted from 8-bit RGB color to an 8-bit greyscale format and adjusted for consistent average brightness and contrast. In all cases the two pairs of wings present on each individual were inspected and the best preserved/imaged forewing and hindwing set selected to represent the specimen. In those cases where the best preserved/imaged wing was collected from the body’s right side the wing image was mirrored to the left-side orientation to render the wing pose comparable across all species. Once these image-processing and pose-standardization procedures had been carried out, the processed wing images were written to separate image files in the non-compressed TIFF file format to form an archive of *Trithemis* forewing and hindwing images. Additional file 1: plates 1 and 2 were assembled from these archive images.

### Classic GM-style analysis

In order to compare our *Trithemis* ecomorphological wing shape results to those of Outomuro et al. [47] a GM-style morphometric analysis was carried out on a combined landmark-semilandmark dataset that included a set of internal landmarks as well as peripheral outline landmarks and semilandmarks. Figure 1B illustrates the positions of these landmark and semilandmark point locations on a representative set of *Trithemis annulata* wings (see Additional file 2: formal definitions of each landmark/semilandmark). One advantage of working with a group whose species exhibit such similar forewing and hindwing morphologies is that the same landmark and semilandmark points can be located on both the forewings and hindwings of every specimen in the dataset. This ensured comparable geometric coverage, thus facilitating comparisons across wing types as well as across habitat groups. Use of internal landmarks, peripheral outline landmarks and peripheral outline semilandmark point locations to characterize wing morphologies also ensured that a better representation of localized morphological similarities and differences across species was obtained than would have been the case if only peripheral outline landmarks or semilandmarks had been employed. The inclusion of internal landmarks added information to the analysis and assisted in making the collection of geometric information across the wing forms as even as possible.

In all, 13 landmarks located at the origins, intersections or peripheral termini of major wing veins , and 25 semilandmarks located in five different peripheral outline zones defined by landmarks 1, 6, 13, 20, 25 and 31, were used to represent wing form in the classic GM-style analysis. The number and location of peripheral semilandmarks were specified using the extended eigenshape protocol of MacLeod ([58, 81]; see also [82]) which allows the sample to determine how many equally-spaced semilandmarks are required to represent the geometry of outline zone peripheries to a consistent level of geometric accuracy across all specimens. Under the sampling scheme employed for this investigation all outline periphery zones were represented to an accuracy of greater than 95 percent.

Following collection of these data forewing and hindwing landmark-semilandmark configurations were aligned and scaled using the generalized least-squares Procrustes procedure [83]. The aligned shape coordinates were then used to produce the species-specific mean shape configurations that were employed in the test for phylogenetic covariation in wing shapes, against the *Trithemis* ultrametric tree provided by Damm et al. ([49], Fig. 2). The multivariate generalization (*K*_mult_) of the *K* statistic [84] described by Adams [48] was employed to facilitate the test of phylogenetic covariation in the wing-shape datasets.

In order to determine whether the shapes of *Trithemis* forewings and/or hindwings, as represented by these landmark-semilandmark shape configurations, exhibited consistent and statistically significant shape differences between species found in contrasting landscape and water body habitats, the dimensionality of the landmark-semilandmark shape-coordinate data was first reduced by subjecting it to a covariance-based PCA (see Additional files 3 for listings of the computer codes used for all data analyses). Component scores on the set of eigenvectors sufficient to account for 95 percent of the pooled-sample shape variation were retained and submitted to secondary CVA using the landscape and water body habitat assignments as the grouping variable. Projections of the PCA configuration-shape scores onto the single linear discriminant vector enabled visualization of the degree to which shape distinctions existed among our *Trithemis* forewing and hindwing shape configurations. A number of recent authors in various natural history, ML, and archeological fields have employed a combined PCA-CVA approach similar to the one used in this investigation to facilitate the analysis of group separations in a linear multivariate context (e.g., [45, 46, 85–87]. Recently Rohlf [54] has reviewed data-analysis strategies for coping with high-dimensional data in group-discrimination contexts and identified this PCA-CVA technique as one that can possibly circumvent the “curse of dimensionality” issue.

Geometric interpretation of the between-habitat guild wing-shape distinctions was facilitated by back-projecting CV scores into the PCA space and then back-projecting those coordinate positions into the space of the original shape variables (see [88] for a description of this technique). The last step of this procedure involved testing the statistical significance of the observed difference in mean vector orientations for the landscape and water body groups using a bootstrapped version of the standard Hotelling’s T^2^ test [88, 89].

### Direct GM-style analysis of images

In order to compare the GM-style analysis of wing morphology as represented by a sparse set of landmarks and semilandmarks with a mathematically equivalent direct analysis of wing images, subsets of these same forewing and hindwing images that did not include labels were processed to standardize their frame sizes, image sizes, orientations, and pixel color scales in order to render their images geometrically comparable. This processing operation also involved a reduction in the overall sizes of the image fames in order to reduce pixel redundancies, boost each image’s geometric information content, and perform an initial reduction in the image datasets’ dimensionality. After processing, forewing images all occupied the central region of a 200 × 56 pixel, white image frame and the hindwing images a 200 × 81 pixel, white image frame. Despite the high level of resolution reduction entailed by this procedure, all taxonomically critical aspects of wing morphology remained clearly visible on the processed images including the forms of the wing outline, all major wing veins and the size, location and intensity of the of colored areas (e.g., distal pterostigma, see Fig. 1A; proximal darkly pigmented hindwing regions of *T. tropicana* and *T. kirbyi*, the lightly pigmented regions of *T. annulata* and *T. bredoi*, see Additional file 1: Plate 2). Once the greyscale pixel brightness values had been exported and reformatted into a data matrix these were submitted to the same PCA-CVA-based data-analysis procedure employed in the GM-style analysis to facilitate direct comparison with the landmark-semilandmark morphology-characterization results.

### Embedded, image-contrast deep learning analysis of images

In order to determine whether morphological distinctions between habitat categories could be improved and/or clarified by a non-linear discriminant analysis procedure, a “deep learning” convolution neural network (CNN) was employed to analyze the image datasets directly. Our CNN architecture was based on the LeNet-5 system [19–21], which is arguably, the CNN that sparked initial interest in “deep learning” using convolution-based, multi-layer artificial neural networks. The LeNet-5 architecture achieved 98.5% accuracy when tested on the 10,000 test images included in the 70,000-image Modified Nation Institute of Standards and Technology (MNIST) image database (see http://yann.lecun.com/exdb/mnist/) after being trained on the remaining 60,000 28 × 28 pixel digital images.

All CNNs consist of an input layer that receives the information to be processed (in our case images) and an output layer that makes the final allocation of the processed data into one of a number discriminant vectors. Between these a variable number of connected or “hidden” layers exist that process the data by (1) accepting the information from the input or previous layers, (2) evaluating this information for patterns consistent with those established by a previously identified training set of images that have been allocated to their appropriate categories (in our investigation landscape and water-body habitat) and (3) passing the processed data on to the next layer. This layered design is used to overcome the problem of full connectivity which is impractical to apply to large images, but can be applied successfully to small images. For our analysis we adopted the standard LeNet default of autoencoding, or “stepping down” the input image resolutions to, in our case, a set of 40 × 40, 8-bit grayscale pixel values as an initial processing step.

Although LeNet-5 is but one of several advanced, gradient-descent CNN architectures for image-based automated identification applications (see https://resources.wolframcloud.com/NeuralNetRepository), it remains one of the most efficient, best understood, and most flexible of the CNN architectures available currently. The LeNet architecture also has advantages over more elaborate CNN designs in that their complexity requires (optimally) that they be trained with vast numbers of example images in order to avoid the ‘curse of dimensionality’ problem [43]. The LeNet architecture is relative simple—containing only eight processing layers—and so better suited to the analysis of small training sets, especially if only a limited number of group differences are of interest. The overall structure of the LeNet-5 architecture employed in this investigation is listed in Table 5.

**Table 5:** Layer structure of the LeNet-5 CNN employed in this investigation

Layers	Type	Parameters
		Image
1	Input	3-tensor (size: 1 × 28 × 28)
2	Convolution	3-tensor (size: 10 × 25 × 25)
3	Ramp	3-tensor (size: 10 × 25 × 25)
4	Pooling	3-tensor (size: 10 × 12 × 12)
5	Convolution	3-tensor (size: 20 × 9 × 9)
6	Ramp	3-tensor (size: 20 × 9 × 9)
7	Pooling	3-tensor (size: 20 × 4 × 4)
8	Flatten	Vector (size: 320)
9	Linear	Vector (size: 2)
10	Output	Vector (size: 2)

Sizes refer to pixels for layers 1–7, variables for layers 8–10

Even after selecting a relatively simple CNN design, obtaining a sufficient number of example images can be challenging. While our image dataset is relatively large by biometric and morphometric standards, it is quite small in the context of ML analysis. Such datasets typically result in overtrained systems that are unreliable when asked to identify genuine unknown specimens.

**Fig. 11:**
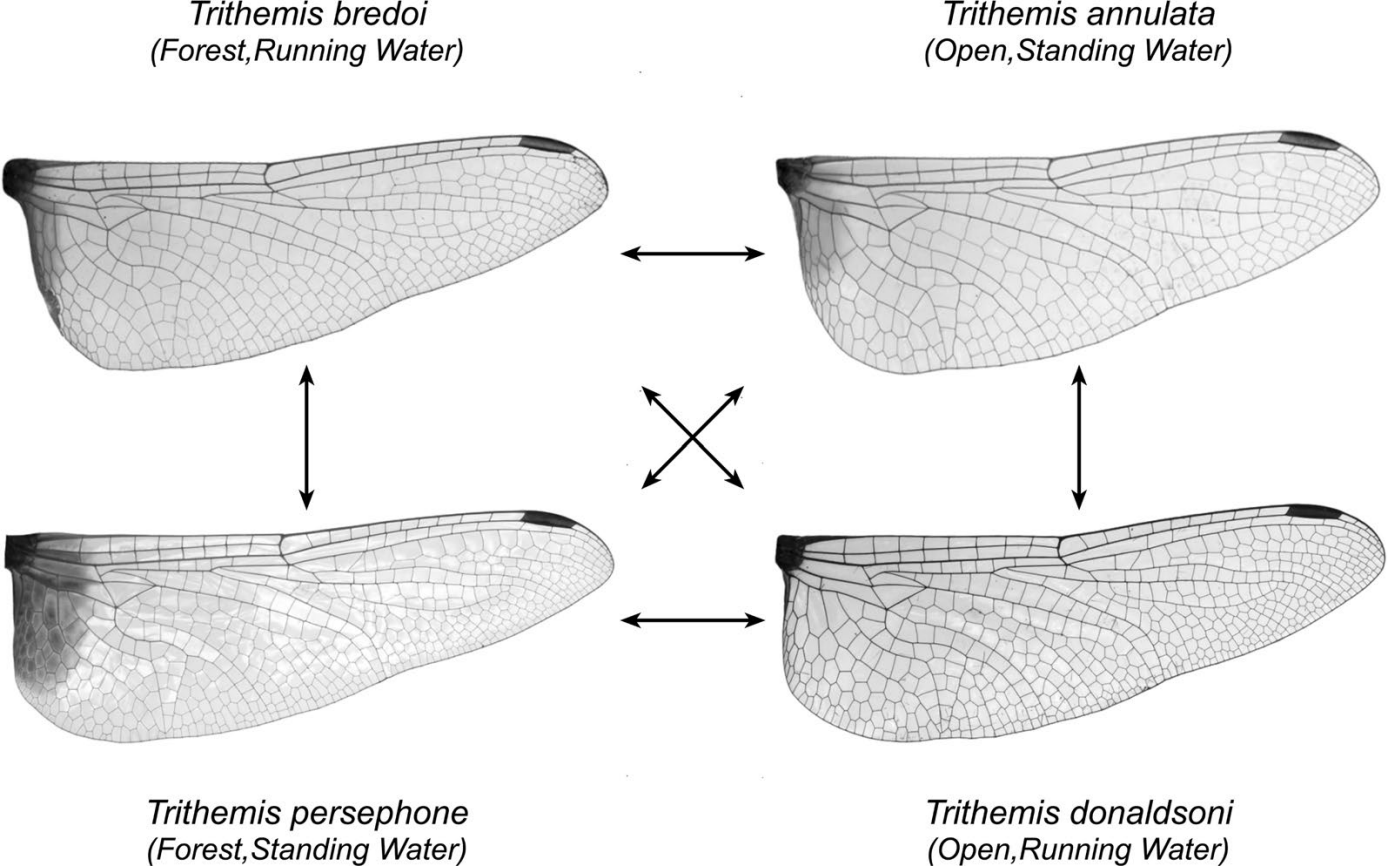
Example of embedded, paired comparison within *Trithemis* species’ hindwings, including within landscape group contrasts (vertical arrows), within water-body group contrasts (horizontal arrows) and between landscape and water-body group contrasts (diagonal arrows). The existence of a multitude of paired comparisons such as these, if they are used as the basis for a morphological assessment of within-group similarity and between-group difference can, in many instances, counteract the effect of inherently small sample sizes. However, in order for this strategy to produce results that can be used with confidence care must be taken either to obtain a representative sample of morphological variation and to be circumspect in interpreting the results of data analyses

Potentially, this problem can be circumvented by opting for training as an embedded learning system, in which the object is not to learn the characteristics of a priori-defined groups themselves but, rather, patterns of explicit similarities and differences between pairs of images that either do, or do not, belong to the same training group (Fig. 11). Recent published applications of this strategy have focused on systems for describing differences between image pairs drawn from large datasets using text-based descriptors [90, 91] as well as image-based analyses [33, 45, 46, 59, 71, 92]. In terms of the analysis of small-to-modestly sized samples, there are many advantages to this approach, including relaxation of the use of single assessments of individual forms insofar as all, or most, pairwise comparisons between images in a dataset can be employed for CNN training. Despite the fact that our image sample contains only 217 and 227 individuals (Table 4), a total of 46,872 and 51,302 pairwise comparisons can be drawn from them: including large numbers of within-group and between-group pairs. By focusing CNN training on differences among images of the same group, and between images of different groups, training can proceed more efficiently than would be possible otherwise.

## Supplementary Information

**Additional file 1:** Trithemis wing images archive.

**Additional file 2:** Datasets and results archive.

**Additional file 3:** Software Archive.

## Data Availability

All data, software code listings, images and intermediate results files are available at https://doi.org/10.5061/dryad.41ns1rnf3 and/or from the corresponding author upon request.

## References

[1] Gould SJ, Lewontin RC (1979). The spandrels of San Marco and the Panglossian paradigm: a critique of the adaptationist programme. Proc R Soc London Ser B.

[2] Felsenstein J (1985). Phylogenies and the comparative method. Am Nat.

[3] Harvey PH, Pagel MD (1991). The comparative method in evolutionary biology.

[4] Garland T, Harvey PH, Ives AR (1992). Procedures for the analysis of comparative data using phylogenetically independent contrasts. Syst Biol.

[5] Garland T, Midford PE, Ives AR (1999). An introduction to phylogenetically based statistical methods, with a new method for confidence intervals on ancestral values. Am Zool.

[6] Ives AR, Midford PE, Garland T (2007). Within-species variation and measurement error in phylogenetic comparative methods. Syst Biol.

[7] Harmon LJ (2018). Phylogenetic comparative methods: learning from trees.

[8] Adams DC, Collyer ML (2018). Multivariate phylogenetic comparative methods: evaluations, comparisons, and recommendations. Syst Biol.

[9] Kendall DG (1984). Shape manifolds, procrustean metrics and complex projective spaces. Bull London Math Soc.

[10] Kendall DG (1986). Comment on "size and shape spaces for landmark data in two dimensions by Fred L. Bookstein Stat Sci.

[11] Bookstein FL (1986). Size and shape spaces for landmark data in two dimensions. Stat Sci.

[12] Bookstein FL (1991). Morphometric tools for landmark data: geometry and biology.

[13] Bookstein FL, Rohlf FJ. Proceedings of the Michigan morphometrics workshop. Ann Arbor, Michigan: The University of Michigan Museum of Zoology, 1990.

[14] Goodall CR (1991). Procrustes methods in the statistical analysis of shape. J R Stat Soc Ser B.

[15] Adams DC, Rohlf FJ, Slice DE (2004). Geometric morphometrics: ten years of progress following the ‘revolution’. Ital J Zool.

[16] Adams DC, Rohlf FJ, Slice DE (2021). A field comes of age: geometric morphometrics in the 21^st^ century. Hystrix.

[17] Fukushima K (1979). Neural network model for a mechanism of pattern recognition unaffected by shift in position—neocognitron. Trans Inst Electron Commun Eng..

[18] Fukushima K (2013). Artificial vision by multi-layered neural networks: neocognitron and its advances. Neural Netw.

[19] LeCun Y, Simard P, Pearlmutter B, Hanson S, Cowan J, Giles L (1993). Automatic learning rate maximization by on-line estimation of the Hessian’s eigenvectors. Advances in neural information processing systems.

[20] LeCun Y, Bottou L, Bengio Y, Haffner P (1998). Gradient-based learning applied to document recognition. Proc IEEE.

[21] LeCun Y, Bengio Y, Hinton G (2015). Deep learning. Nature.

[22] Schmidhuber J (2015). Deep learning in neural networks: an overview. Neural Netw.

[23] Bishop CM (2006). Pattern recognition and machine learning.

[24] Marsland S (2015). Machine learning: an algorithmic perspective.

[25] MacLeod N, Sagar BSD, Cheng Q, McKinley J, Agterberg F (2021). Artificial intelligence in the earth sciences. Encyclopedia of Mathematical Geosciences.

[26] Rohlf FJ, Marcus LF, Bello E, García-Valdecasas A (1993). Relative warp analysis and an example of its application to mosquito wings. Contributions to Morphometrics.

[27] Klingenberg CP, McIntyre GS (1998). Geometric morphometrics of developmental instability: analyzing patterns of fluctuating asymmetry with Procrustes methods. Evolution.

[28] Klingenberg CP, McIntyre GS, Zaklan SD (1998). Left-right asymmetry of fly wings and the evolution of body axes. Proc R Soc B Biol Sci.

[29] Comstock JH, Needham JG (1898). The wings of insects. Am Nat.

[30] Wootton RJ (1979). Function, homology and terminology in insect wings. Syst Entomol.

[31] Yang P, Ma CS, Wen H, Zhan QB, Wang XL (2015). A tool for developing an automatic insect identification system based on wing outlines. Nat Sci Rep.

[32] Sontigun N, Sukontason KL, Zajac BK, Zehner R, Sukontason K, Wannasan A, Amendt J (2017). Wing morphometrics as a tool in species identification of forensically important blow flies of Thailand. Parasit Vectors.

[33] Hall MJR, MacLeod N, Wardhana AH (2014). Use of wing morphometrics to identify populations of the Old World screwworm fly, *Chrysomya bezziana* (Diptera: Calliphoridae): a preliminary study of the utility of museum specimens. Acta Trop.

[34] MacLeod N, Hall MJR, Wardhana AH (2018). Towards the automated identification of *Chrysomya* blow flies from wing images. Med Vet Entomol.

[35] Wootton RJ (1992). Functional morphology of insect wings. Annu Rev Entomol.

[36] Blanke A (2018). Analysis of modularity and integration suggests evolution of dragonfly wing venation mainly in response to functional demands. J R Soc Interface.

[37] Sievwright H, MacLeod N (2012). Eigensurface analysis, ecology, and modelling of morphological adaptation in the falconiform humerus (Falconiformes: Aves). Zool J Linn Soc.

[38] Altshuler DL, Bahlman JW, Dakin R, Gaede AH, Goller B, Lentink D, Segre PS, Skandalis DA (2014). The biophysics of bird flight: functional relationships integrate aerodynamics, morphology, kinematics, muscles, and sensors. Can J Zool.

[39] Baliga B, Szabo I, Altshuler DL (2019). Range of motion in the avian wing is strongly associated with flight behavior and body mass. Sci Adv.

[40] Norberg UM, Rayner JMV (2015). Ecological morphology and flight in bats (Mammalia; Chiroptera): wing adaptations, flight performance, foraging strategy and echolocation. Philos Trans R Soc London Ser B..

[41] Mengesha TE, Vallance RR, Barraja M, Mittal R (2009). Parametric structural modeling of insect wings. Bioinspir Biomim.

[42] Salcedo MK, Hoffmann J, Donoughe S, Mahadevan L (2019). Computational analysis of size, shape and structure of insect wings. Biol Open..

[43] Altman N, Krzywinski M (2018). The curse(s) of dimensionality. Nat Methods.

[44] Mitteröcker P, Bookstein FL (2011). Linear discrimination, ordination, and the visualization of selection gradients in modern morphometrics. Evol Biol.

[45] MacLeod N. The direct analysis of digital images (eigenimage) with a comment on the use of discriminant analysis in morphometrics. In: Lestrel, PE, editor, Proceedings of the Third International Symposium on Biological Shape Analysis. Singapore: World Scientific, 2015, p. 156–182.

[46] MacLeod N (2018). The quantitative assessment of archaeological artifact groups: beyond geometric morphometrics. Quat Sci Rev.

[47] Outomuro D, Dijkstra KD, Johansson F (2013). Habitat variation and wing coloration affect wing shape evolution in dragonflies. J Evol Biol.

[48] Adams DC (2014). A generalized K statistic for estimating phylogenetic signal from shape and other high-dimensional multivariate data. Syst Biol.

[49] Damm S, Dijkstra KDB, Hadrys H (2010). Red drifters and dark residents: the phylogeny and ecology of a Plio-Pleistocene dragonfly radiation reflects Africa’s changing environment (Odonata, Libellulidae, *Trithemis*). Mol Phylogenet Evol.

[50] Nel A (1991). Un nouvel Odonate fossile du Miocène de Bellver de Cerdana (Espagne) (Odonata, Libellulidae). Entomolologica Gall.

[51] Matthews BW (1975). Comparison of the predicted and observed secondary structure of T4 phage lysozyme. Biochim Biophys Acta Protein Struct.

[52] Jurman G, Riccadonna S, Furlanello C (2012). A comparison of MCC and CEN error measures in multi-class prediction. PLoS ONE.

[53] Chicco D, Jurman G (2020). The advantages of the Matthews correlation coefficient (MCC) over F1 score and accuracy in binary classification evaluation. BMC Genomics.

[54] Rohlf, FJ. Why clusters and other patterns can seem to be found in analyses of high-dimensional data. Evol Biol. 2020; 1–16.

[55] Bollback JP (2006). SIMMAP: stochastic character mapping of discrete traits on phylogenies. BMC Bioinform.

[56] Klingenberg CP, Gidaszewski NA (2010). Testing and quantifying phylogenetic signals and homoplasy in morphometric data. Syst Biol.

[57] Losos JB (2011). Seeing the forest for the trees: the limitations of phylogenies in comparative biology. Am Nat.

[58] MacLeod N (1999). Generalizing and extending the eigenshape method of shape visualization and analysis. Paleobiology.

[59] MacLeod N, Kolska HL (2020). Machine-learning strategies for testing patterns of morphological variation in small samples: sexual dimorphism in gray wolf (*Canis lupus*) crania. BMC Biol.

[60] Zelditch ML, Fink WL, Swiderski DL (1995). Morphometrics, homology, and phylogenetics: quantified characters as synapomorphies. Syst Biol.

[61] Hanot P, Bayarsaikhan J, Guintard C, Haruda A, Mijiddorj E, Schafberg R, Taylor W (2021). Cranial shape diversification in horses: variation and covariation patterns under the impact of artificial selection. BMC Ecol Evol.

[62] Molnar C. Interpretable machine learning: a guide for making black box models explainable; 2020. https://christophm.github.io/interpretable-ml-book/.

[63] Arteaga C (2019). Interpretable machine learning for image classification with LIME. Toward Data Sci..

[64] Stewart M. Guide to interpretable machine learning. Toward Data Sci. 2020: 1–40.

[65] MacLeod N (2007). Automated taxon identification in systematics: theory, approaches, and applications.

[66] MacLeod N. On the use of machine learning methods in morphometric analysis. In Lestrel, PE, editor. Proceedings of the Third International Symposium on Biological Shape Analysis. Singapore: World Scientific, 2017, p. 134–171.

[67] Van Bocxlaer B, Schultheiß R (2010). Comparison of morphometric techniques for shapes with few homologous landmarks based on machine-learning approaches to biological discrimination. Paleobiology.

[68] Criminisi A (2016). Machine learning for medical images analysis. Med Image Anal.

[69] Favret C, Sieracki JM (2016). Machine vision automated species identification scaled towards production levels. Syst Entomol.

[70] Monson TA, Armitage DW, Hlusko LJ (2018). Using machine learning to classify extant apes and interpret the dental morphology of the chimpanzee-human last common ancestor. PaleoBios.

[71] Hoyal Cuthill JF, Guttenberg N, Ledger S, Crowther R, Huertas B (2019). Deep learning on butterfly phenotypes tests evolution’s oldest mathematical model. Sci Adv.

[72] Courtenay LA, Huguet R, González-Aguilera D, Yravedra JA (2020). hybrid geometric morphometric deep learning approach for cut and trampling mark classification. Appl Sci.

[73] Courtenay LA, Yravedra J, Huguet R, Aramendi J, Maté-González MÁ, González-Aguilera D, Arriaza MC (2019). Combining machine learning algorithms and geometric morphometrics: a study of carnivore tooth marks. Palaeogeogr Palaeoclimatol Palaeoecol.

[74] van de Lande LS, Papaioannou A, Dunaway DJ, Geometric morphometrics aided by machine learning in craniofacial surgery. J. Orthod. 2019; 1–3. 10.1177/1465312519840030 31056036

[75] MacLeod N (2001). The role of phylogeny in quantitative paleobiological analysis. Paleobiology.

[76] Derrickson EM, Ricklefs RE (1988). Taxon-dependent diversification of life-history traits and the perception of phylogenetic constraints. Funct Ecol.

[77] Blomberg SP, Garland T (2002). Tempo and mode in evolution: phylogenetic inertia, adaptation and comparative methods. J Evol Biol.

[78] Hardy GH (1940). A mathematician’s apology.

[79] MacArthur RH (1972). Geographical ecology: patterns in the distribution of species.

[80] de Loureiro NS, Pontes L (2012). The *Trithemis nigra* (Odonata: Libellulidae) of Príncipe Island, Gulf of Guinea. Afr J Ecol.

[81] MacLeod N (2012). Going round the bend II: extended eigenshape analysis. Palaeontol Assoc Newsl.

[82] Mayall P, Pilbrow V, Bitadze L (2017). Migrating huns and modified heads: eigenshape analysis comparing intentionally modified crania from Hungary and Georgia in the migration period of Europe. PLoS ONE.

[83] Rohlf FJ, Slice D (1990). Extensions of the Procrustes method for optimal superposition of landmarks. Syst Zool.

[84] Christenson AL, Read DW (1977). Numerical taxonomy, r-mode factor analysis and archeological classification. Am Antiq.

[85] Anderson MJ, Willis TJ (2003). Canonical analysis of principal coordinates: a useful method of constrained ordination for ecology. Ecology.

[86] Marrama G, Kriwet J (2017). Principal component and discriminant analyses as powerful tools to support taxonomic identification and their use for functional and phylogenetic signal detection of isolated fossil shark teeth. PLoS ONE.

[87] MacLeod N (2009). Form & shape models. Palaeontol Assoc Newsl.

[88] Hotelling H (1931). The generalization of Student’s ratio. Ann Math Stat.

[89] Manly BFJ, Alberto JAN (2017). Multivariate statistical methods: a primer.

[90] Forbes M, Kaeser-Chen C, Sharma P, Belongie S (2019). Neural naturalist: generating fine-grained image comparisons. ArXiv.

[91] Jhamtani H, Berg-Kirkpatrick T (2018). Learning to describe differences between pairs of similar images. ArXiv.

[92] MacLeod N, Canty RJ, Polazek A (2022). Morphology-based identification of *Bemisia tabaci* cryptic species puparia via embedded group-contrast convolution neural network analysis. Syst Biol.

[93] Blomberg SP, Garland T, Ives AR (2003). Testing for phylogenetic signal in comparative data: behavioral traits are more labile. Evolution.

