# Supplementary figures and images for: What you sample is what you get: ecomorphological variation in Trithemis (Odonata, Libellulidae) dragonfly wings reconsidered (part 2 of 3)
Source: BMC Ecol Evol. 2022 Apr 11;22:43. doi: 10.1186/s12862-022-01978-y (PMC8996507; doi:10.1186/s12862-022-01978-y)

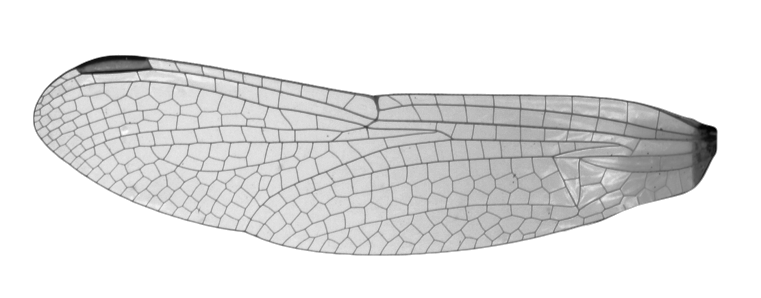

Supplement: Supplementary file 1 — Additional file 1: Trithemis wing images archive. [file 12862_2022_1978_MOESM1_ESM.zip › Additional Files 1/Trithemis Wing Images Archive/Trithemis Wing Images/Forewings/Images (w: Numbers)/193.tif]

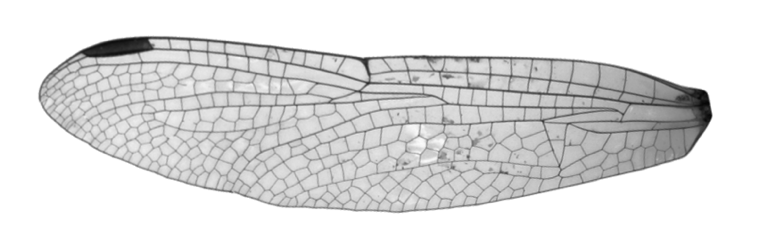

Supplement: Supplementary file 1 — Additional file 1: Trithemis wing images archive. [file 12862_2022_1978_MOESM1_ESM.zip › Additional Files 1/Trithemis Wing Images Archive/Trithemis Wing Images/Forewings/Images (w: Numbers)/030.tif]

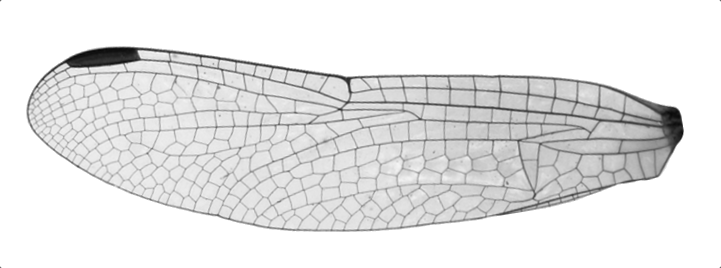

Supplement: Supplementary file 1 — Additional file 1: Trithemis wing images archive. [file 12862_2022_1978_MOESM1_ESM.zip › Additional Files 1/Trithemis Wing Images Archive/Trithemis Wing Images/Forewings/Images (w: Numbers)/024.tif]

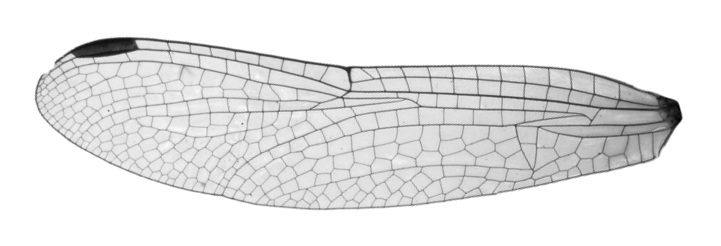

Supplement: Supplementary file 1 — Additional file 1: Trithemis wing images archive. [file 12862_2022_1978_MOESM1_ESM.zip › Additional Files 1/Trithemis Wing Images Archive/Trithemis Wing Images/Forewings/Images (w: Numbers)/018.tif]

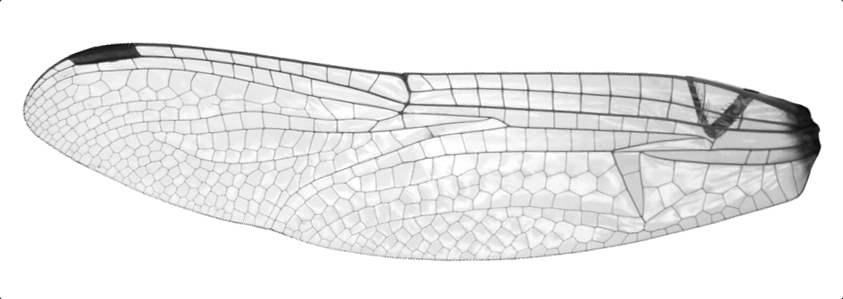

Supplement: Supplementary file 1 — Additional file 1: Trithemis wing images archive. [file 12862_2022_1978_MOESM1_ESM.zip › Additional Files 1/Trithemis Wing Images Archive/Trithemis Wing Images/Forewings/Images (w: Numbers)/232.tif]

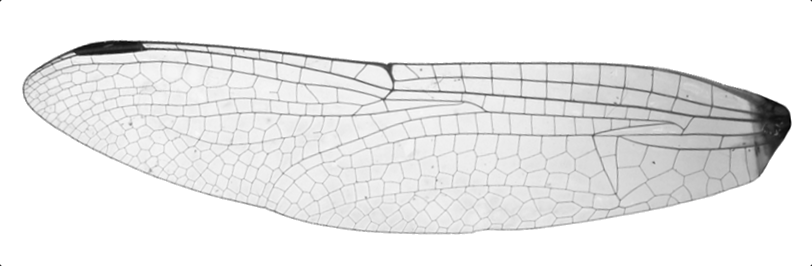

Supplement: Supplementary file 1 — Additional file 1: Trithemis wing images archive. [file 12862_2022_1978_MOESM1_ESM.zip › Additional Files 1/Trithemis Wing Images Archive/Trithemis Wing Images/Forewings/Images (w: Numbers)/233.tif]

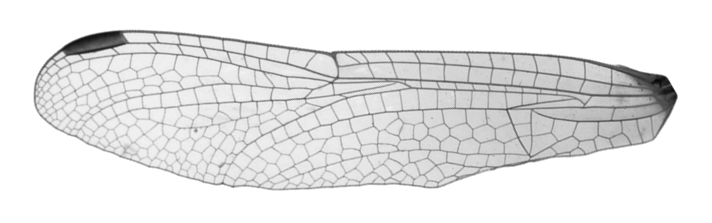

Supplement: Supplementary file 1 — Additional file 1: Trithemis wing images archive. [file 12862_2022_1978_MOESM1_ESM.zip › Additional Files 1/Trithemis Wing Images Archive/Trithemis Wing Images/Forewings/Images (w: Numbers)/019.tif]

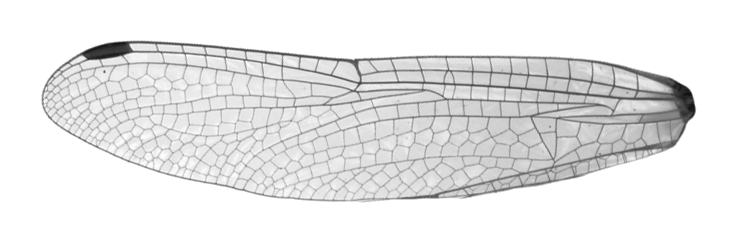

Supplement: Supplementary file 1 — Additional file 1: Trithemis wing images archive. [file 12862_2022_1978_MOESM1_ESM.zip › Additional Files 1/Trithemis Wing Images Archive/Trithemis Wing Images/Forewings/Images (w: Numbers)/025.tif]

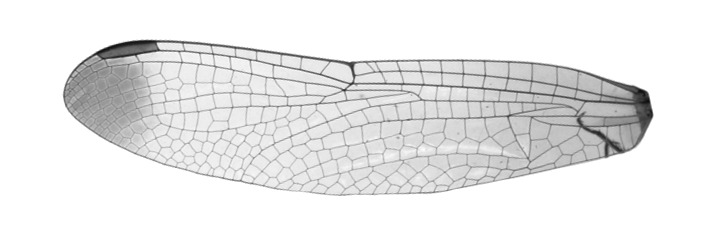

Supplement: Supplementary file 1 — Additional file 1: Trithemis wing images archive. [file 12862_2022_1978_MOESM1_ESM.zip › Additional Files 1/Trithemis Wing Images Archive/Trithemis Wing Images/Forewings/Images (w: Numbers)/031.tif]

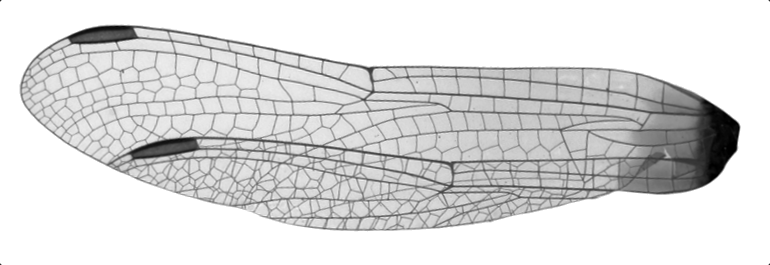

Supplement: Supplementary file 1 — Additional file 1: Trithemis wing images archive. [file 12862_2022_1978_MOESM1_ESM.zip › Additional Files 1/Trithemis Wing Images Archive/Trithemis Wing Images/Forewings/Images (w: Numbers)/192.tif]

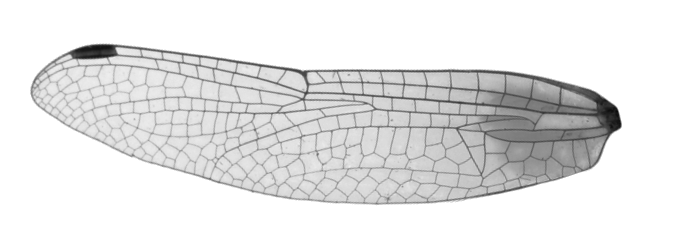

Supplement: Supplementary file 1 — Additional file 1: Trithemis wing images archive. [file 12862_2022_1978_MOESM1_ESM.zip › Additional Files 1/Trithemis Wing Images Archive/Trithemis Wing Images/Forewings/Images (w: Numbers)/186.tif]

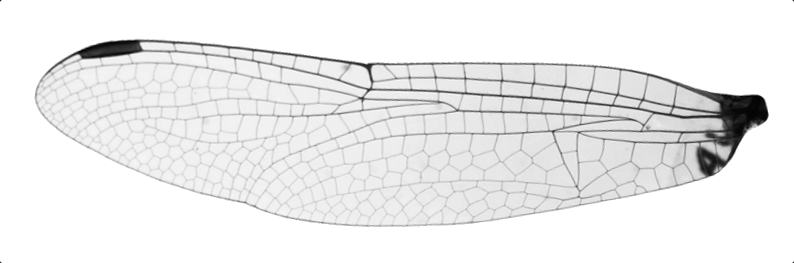

Supplement: Supplementary file 1 — Additional file 1: Trithemis wing images archive. [file 12862_2022_1978_MOESM1_ESM.zip › Additional Files 1/Trithemis Wing Images Archive/Trithemis Wing Images/Forewings/Images (w: Numbers)/147.tif]

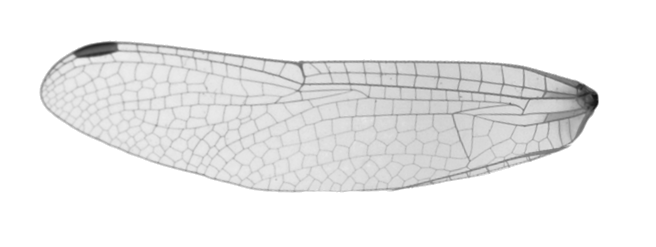

Supplement: Supplementary file 1 — Additional file 1: Trithemis wing images archive. [file 12862_2022_1978_MOESM1_ESM.zip › Additional Files 1/Trithemis Wing Images Archive/Trithemis Wing Images/Forewings/Images (w: Numbers)/190.tif]

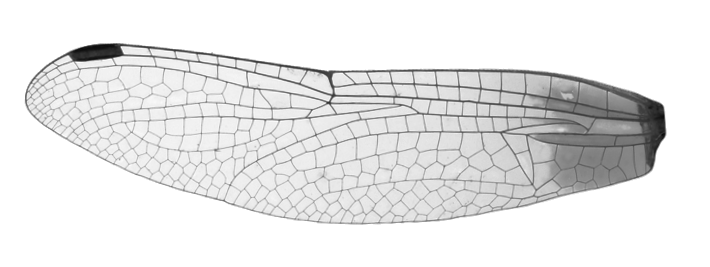

Supplement: Supplementary file 1 — Additional file 1: Trithemis wing images archive. [file 12862_2022_1978_MOESM1_ESM.zip › Additional Files 1/Trithemis Wing Images Archive/Trithemis Wing Images/Forewings/Images (w: Numbers)/184.tif]

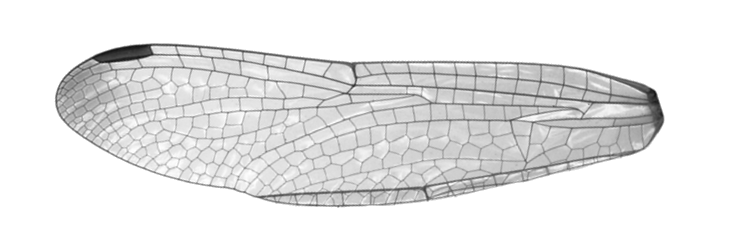

Supplement: Supplementary file 1 — Additional file 1: Trithemis wing images archive. [file 12862_2022_1978_MOESM1_ESM.zip › Additional Files 1/Trithemis Wing Images Archive/Trithemis Wing Images/Forewings/Images (w: Numbers)/027.tif]

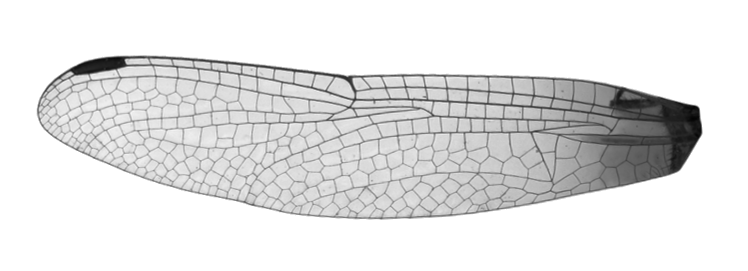

Supplement: Supplementary file 1 — Additional file 1: Trithemis wing images archive. [file 12862_2022_1978_MOESM1_ESM.zip › Additional Files 1/Trithemis Wing Images Archive/Trithemis Wing Images/Forewings/Images (w: Numbers)/033.tif]

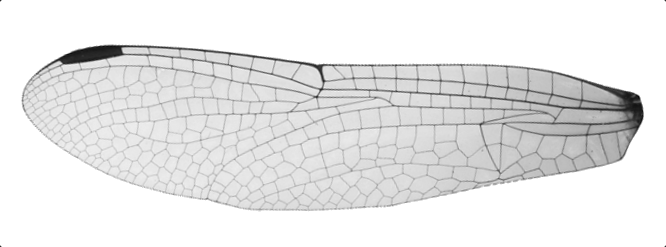

Supplement: Supplementary file 1 — Additional file 1: Trithemis wing images archive. [file 12862_2022_1978_MOESM1_ESM.zip › Additional Files 1/Trithemis Wing Images Archive/Trithemis Wing Images/Forewings/Images (w: Numbers)/231.tif]

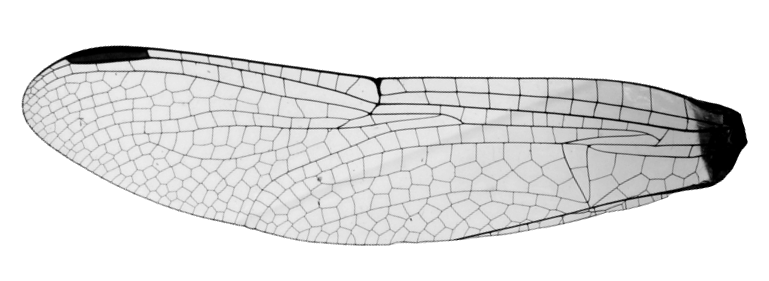

Supplement: Supplementary file 1 — Additional file 1: Trithemis wing images archive. [file 12862_2022_1978_MOESM1_ESM.zip › Additional Files 1/Trithemis Wing Images Archive/Trithemis Wing Images/Forewings/Images (w: Numbers)/219.tif]

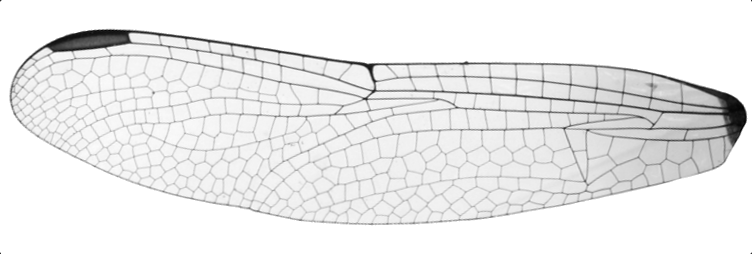

Supplement: Supplementary file 1 — Additional file 1: Trithemis wing images archive. [file 12862_2022_1978_MOESM1_ESM.zip › Additional Files 1/Trithemis Wing Images Archive/Trithemis Wing Images/Forewings/Images (w: Numbers)/218.tif]

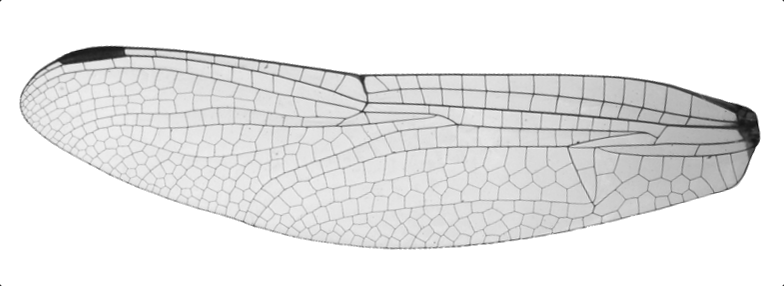

Supplement: Supplementary file 1 — Additional file 1: Trithemis wing images archive. [file 12862_2022_1978_MOESM1_ESM.zip › Additional Files 1/Trithemis Wing Images Archive/Trithemis Wing Images/Forewings/Images (w: Numbers)/230.tif]

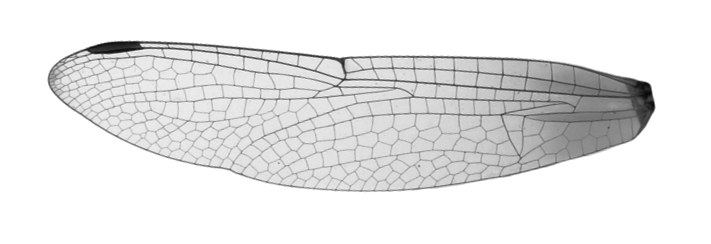

Supplement: Supplementary file 1 — Additional file 1: Trithemis wing images archive. [file 12862_2022_1978_MOESM1_ESM.zip › Additional Files 1/Trithemis Wing Images Archive/Trithemis Wing Images/Forewings/Images (w: Numbers)/032.tif]

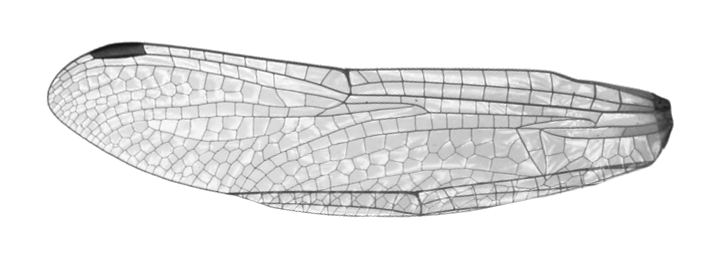

Supplement: Supplementary file 1 — Additional file 1: Trithemis wing images archive. [file 12862_2022_1978_MOESM1_ESM.zip › Additional Files 1/Trithemis Wing Images Archive/Trithemis Wing Images/Forewings/Images (w: Numbers)/026.tif]

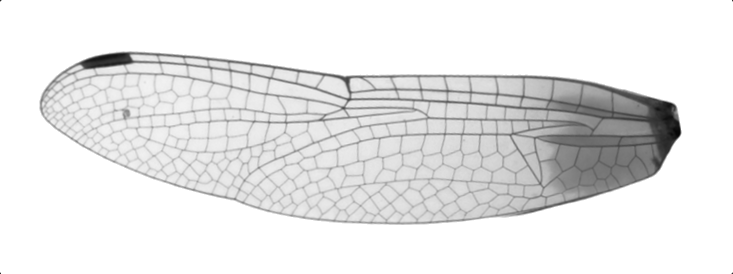

Supplement: Supplementary file 1 — Additional file 1: Trithemis wing images archive. [file 12862_2022_1978_MOESM1_ESM.zip › Additional Files 1/Trithemis Wing Images Archive/Trithemis Wing Images/Forewings/Images (w: Numbers)/185.tif]

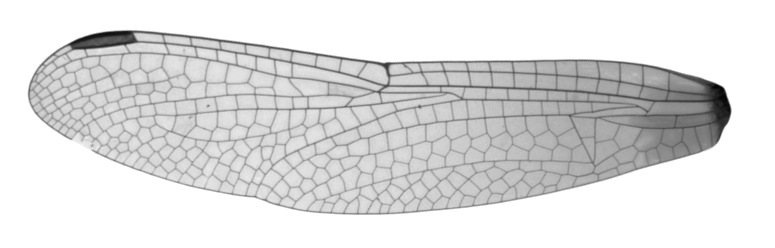

Supplement: Supplementary file 1 — Additional file 1: Trithemis wing images archive. [file 12862_2022_1978_MOESM1_ESM.zip › Additional Files 1/Trithemis Wing Images Archive/Trithemis Wing Images/Forewings/Images (w: Numbers)/191.tif]

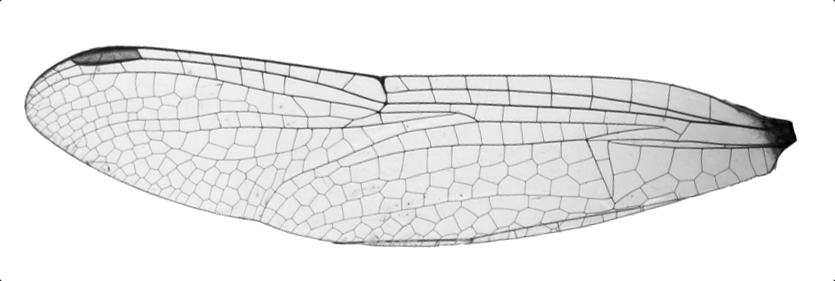

Supplement: Supplementary file 1 — Additional file 1: Trithemis wing images archive. [file 12862_2022_1978_MOESM1_ESM.zip › Additional Files 1/Trithemis Wing Images Archive/Trithemis Wing Images/Forewings/Images (w: Numbers)/156.tif]

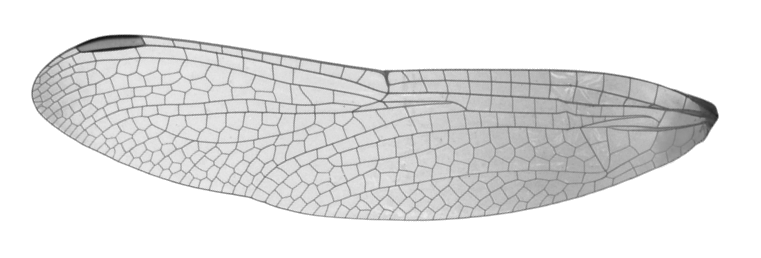

Supplement: Supplementary file 1 — Additional file 1: Trithemis wing images archive. [file 12862_2022_1978_MOESM1_ESM.zip › Additional Files 1/Trithemis Wing Images Archive/Trithemis Wing Images/Forewings/Images (w: Numbers)/195.tif]

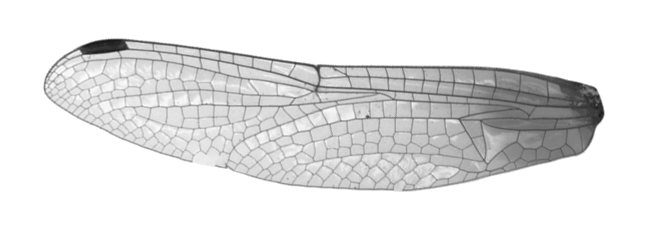

Supplement: Supplementary file 1 — Additional file 1: Trithemis wing images archive. [file 12862_2022_1978_MOESM1_ESM.zip › Additional Files 1/Trithemis Wing Images Archive/Trithemis Wing Images/Forewings/Images (w: Numbers)/181.tif]

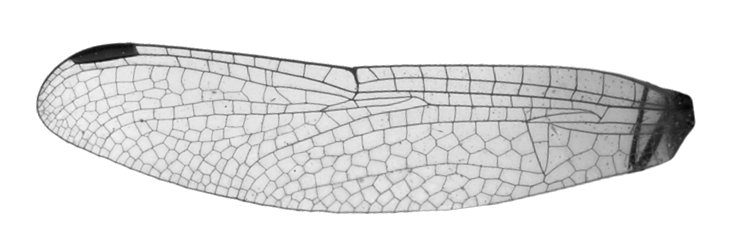

Supplement: Supplementary file 1 — Additional file 1: Trithemis wing images archive. [file 12862_2022_1978_MOESM1_ESM.zip › Additional Files 1/Trithemis Wing Images Archive/Trithemis Wing Images/Forewings/Images (w: Numbers)/022.tif]

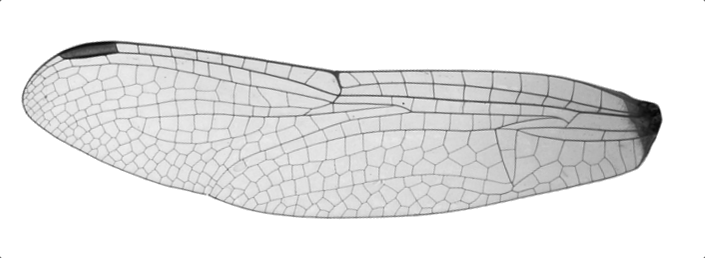

Supplement: Supplementary file 1 — Additional file 1: Trithemis wing images archive. [file 12862_2022_1978_MOESM1_ESM.zip › Additional Files 1/Trithemis Wing Images Archive/Trithemis Wing Images/Forewings/Images (w: Numbers)/036.tif]

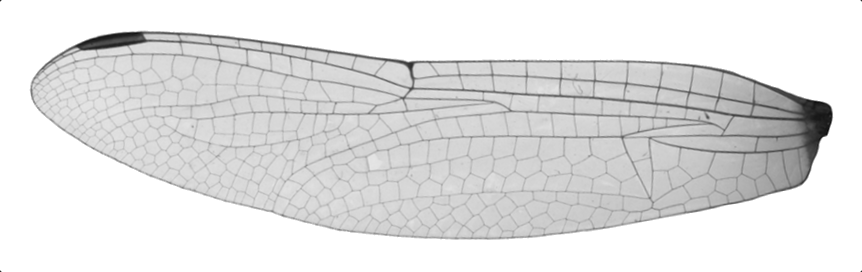

Supplement: Supplementary file 1 — Additional file 1: Trithemis wing images archive. [file 12862_2022_1978_MOESM1_ESM.zip › Additional Files 1/Trithemis Wing Images Archive/Trithemis Wing Images/Forewings/Images (w: Numbers)/234.tif]

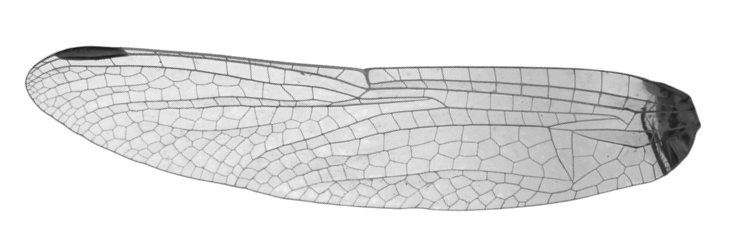

Supplement: Supplementary file 1 — Additional file 1: Trithemis wing images archive. [file 12862_2022_1978_MOESM1_ESM.zip › Additional Files 1/Trithemis Wing Images Archive/Trithemis Wing Images/Forewings/Images (w: Numbers)/235.tif]

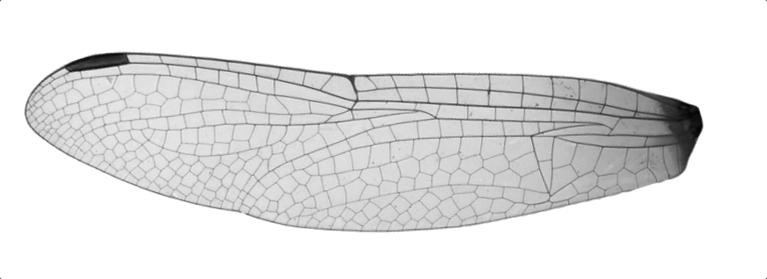

Supplement: Supplementary file 1 — Additional file 1: Trithemis wing images archive. [file 12862_2022_1978_MOESM1_ESM.zip › Additional Files 1/Trithemis Wing Images Archive/Trithemis Wing Images/Forewings/Images (w: Numbers)/037.tif]

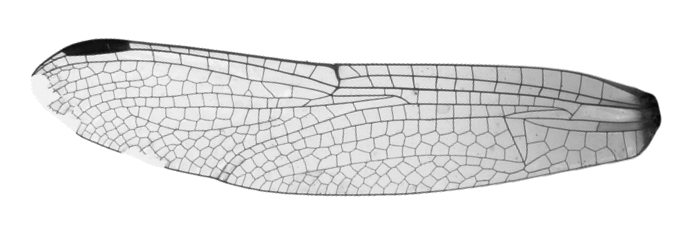

Supplement: Supplementary file 1 — Additional file 1: Trithemis wing images archive. [file 12862_2022_1978_MOESM1_ESM.zip › Additional Files 1/Trithemis Wing Images Archive/Trithemis Wing Images/Forewings/Images (w: Numbers)/023.tif]

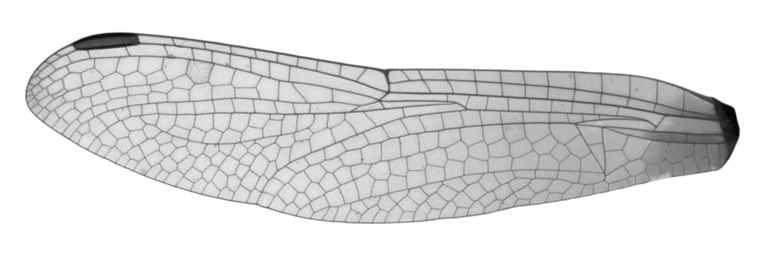

Supplement: Supplementary file 1 — Additional file 1: Trithemis wing images archive. [file 12862_2022_1978_MOESM1_ESM.zip › Additional Files 1/Trithemis Wing Images Archive/Trithemis Wing Images/Forewings/Images (w: Numbers)/194.tif]

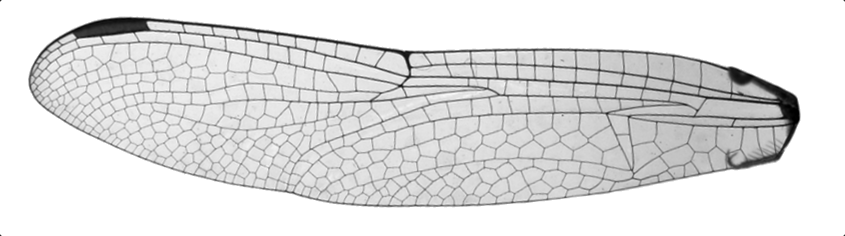

Supplement: Supplementary file 1 — Additional file 1: Trithemis wing images archive. [file 12862_2022_1978_MOESM1_ESM.zip › Additional Files 1/Trithemis Wing Images Archive/Trithemis Wing Images/Forewings/Images (w: Numbers)/143.tif]

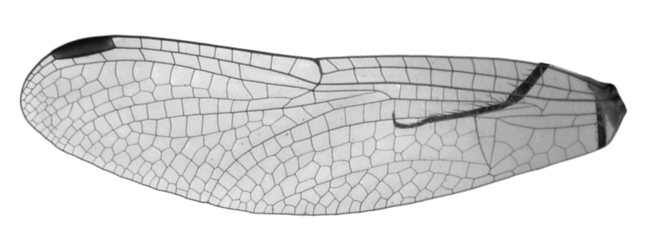

Supplement: Supplementary file 1 — Additional file 1: Trithemis wing images archive. [file 12862_2022_1978_MOESM1_ESM.zip › Additional Files 1/Trithemis Wing Images Archive/Trithemis Wing Images/Forewings/Images (w: Numbers)/169.tif]

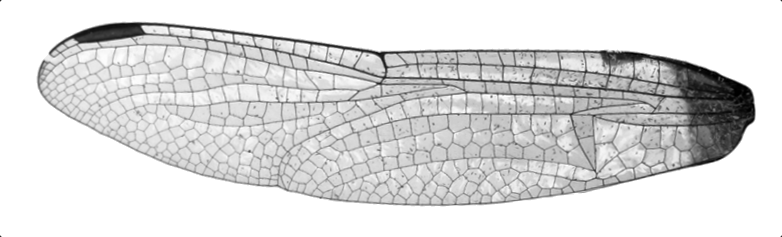

Supplement: Supplementary file 1 — Additional file 1: Trithemis wing images archive. [file 12862_2022_1978_MOESM1_ESM.zip › Additional Files 1/Trithemis Wing Images Archive/Trithemis Wing Images/Forewings/Images (w: Numbers)/141.tif]

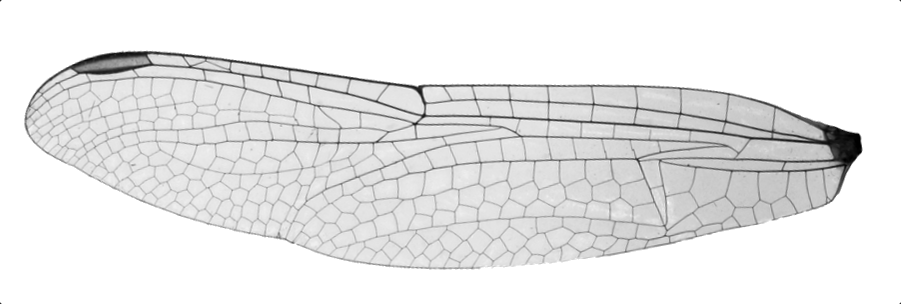

Supplement: Supplementary file 1 — Additional file 1: Trithemis wing images archive. [file 12862_2022_1978_MOESM1_ESM.zip › Additional Files 1/Trithemis Wing Images Archive/Trithemis Wing Images/Forewings/Images (w: Numbers)/155.tif]

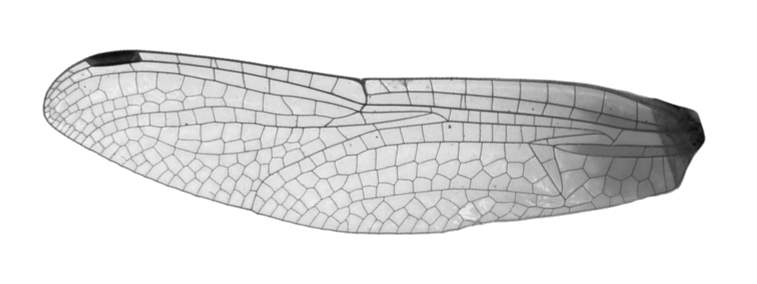

Supplement: Supplementary file 1 — Additional file 1: Trithemis wing images archive. [file 12862_2022_1978_MOESM1_ESM.zip › Additional Files 1/Trithemis Wing Images Archive/Trithemis Wing Images/Forewings/Images (w: Numbers)/182.tif]

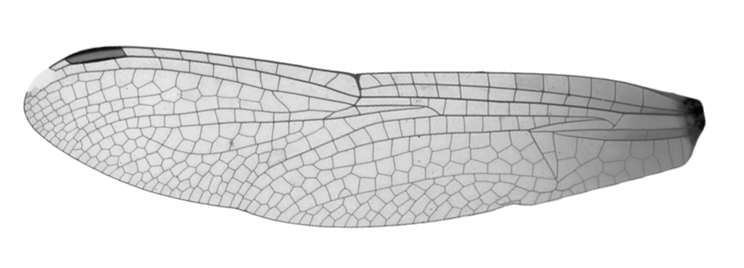

Supplement: Supplementary file 1 — Additional file 1: Trithemis wing images archive. [file 12862_2022_1978_MOESM1_ESM.zip › Additional Files 1/Trithemis Wing Images Archive/Trithemis Wing Images/Forewings/Images (w: Numbers)/196.tif]

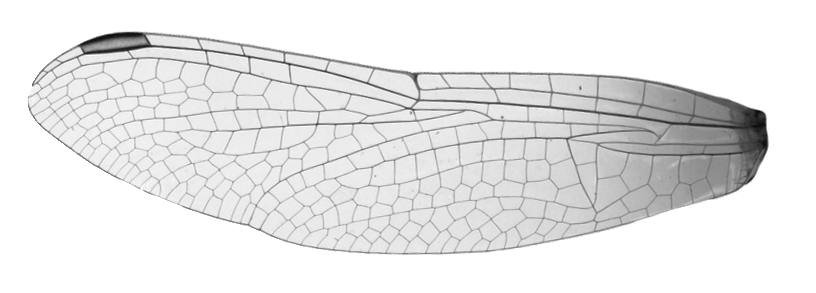

Supplement: Supplementary file 1 — Additional file 1: Trithemis wing images archive. [file 12862_2022_1978_MOESM1_ESM.zip › Additional Files 1/Trithemis Wing Images Archive/Trithemis Wing Images/Forewings/Images (w: Numbers)/009.tif]

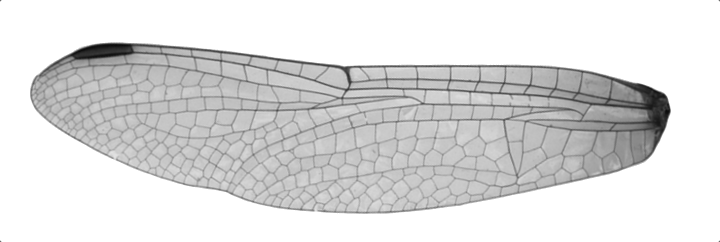

Supplement: Supplementary file 1 — Additional file 1: Trithemis wing images archive. [file 12862_2022_1978_MOESM1_ESM.zip › Additional Files 1/Trithemis Wing Images Archive/Trithemis Wing Images/Forewings/Images (w: Numbers)/035.tif]

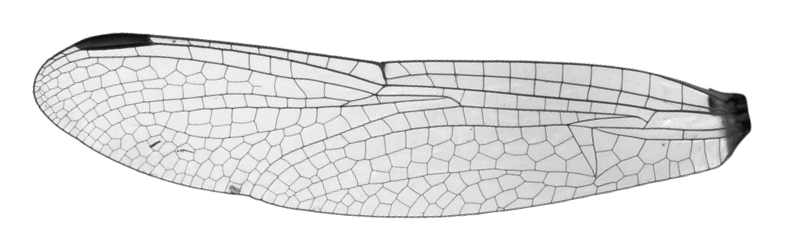

Supplement: Supplementary file 1 — Additional file 1: Trithemis wing images archive. [file 12862_2022_1978_MOESM1_ESM.zip › Additional Files 1/Trithemis Wing Images Archive/Trithemis Wing Images/Forewings/Images (w: Numbers)/021.tif]

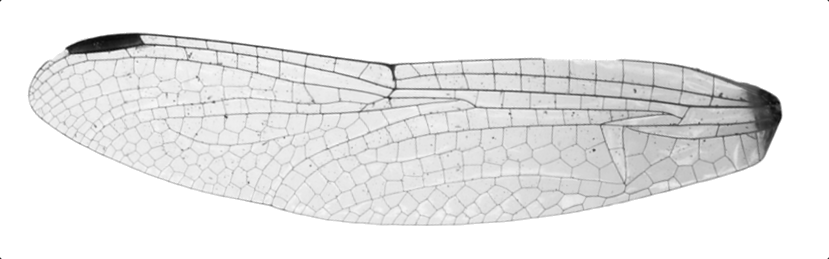

Supplement: Supplementary file 1 — Additional file 1: Trithemis wing images archive. [file 12862_2022_1978_MOESM1_ESM.zip › Additional Files 1/Trithemis Wing Images Archive/Trithemis Wing Images/Forewings/Images (w: Numbers)/237.tif]

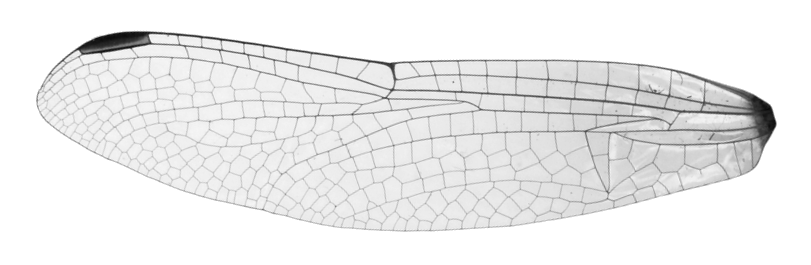

Supplement: Supplementary file 1 — Additional file 1: Trithemis wing images archive. [file 12862_2022_1978_MOESM1_ESM.zip › Additional Files 1/Trithemis Wing Images Archive/Trithemis Wing Images/Forewings/Images (w: Numbers)/236.tif]

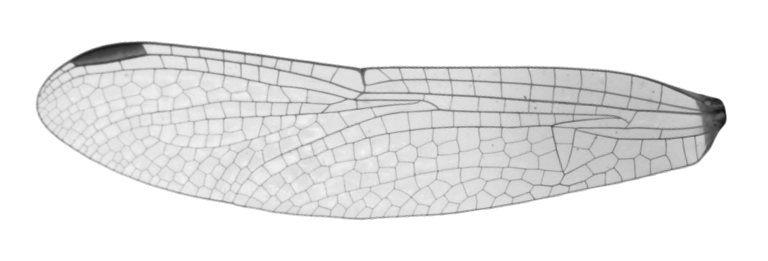

Supplement: Supplementary file 1 — Additional file 1: Trithemis wing images archive. [file 12862_2022_1978_MOESM1_ESM.zip › Additional Files 1/Trithemis Wing Images Archive/Trithemis Wing Images/Forewings/Images (w: Numbers)/020.tif]

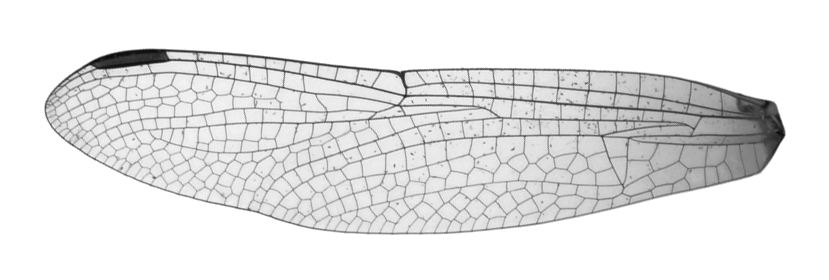

Supplement: Supplementary file 1 — Additional file 1: Trithemis wing images archive. [file 12862_2022_1978_MOESM1_ESM.zip › Additional Files 1/Trithemis Wing Images Archive/Trithemis Wing Images/Forewings/Images (w: Numbers)/034.tif]

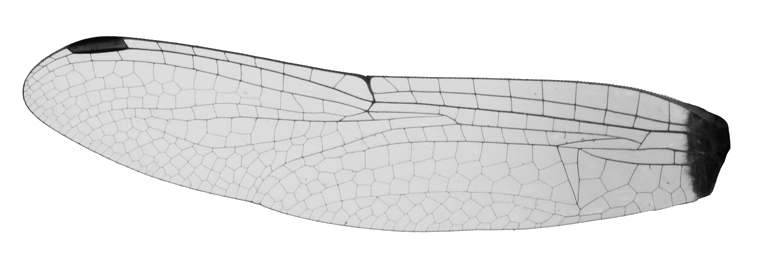

Supplement: Supplementary file 1 — Additional file 1: Trithemis wing images archive. [file 12862_2022_1978_MOESM1_ESM.zip › Additional Files 1/Trithemis Wing Images Archive/Trithemis Wing Images/Forewings/Images (w: Numbers)/008.tif]

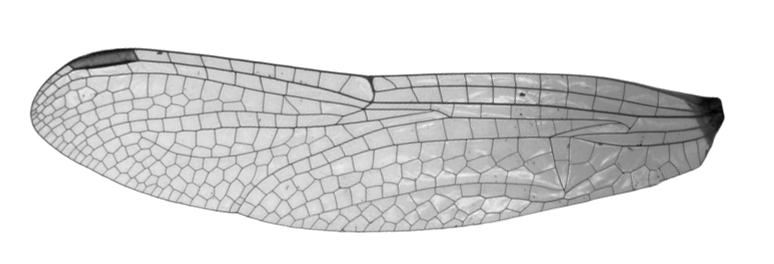

Supplement: Supplementary file 1 — Additional file 1: Trithemis wing images archive. [file 12862_2022_1978_MOESM1_ESM.zip › Additional Files 1/Trithemis Wing Images Archive/Trithemis Wing Images/Forewings/Images (w: Numbers)/197.tif]

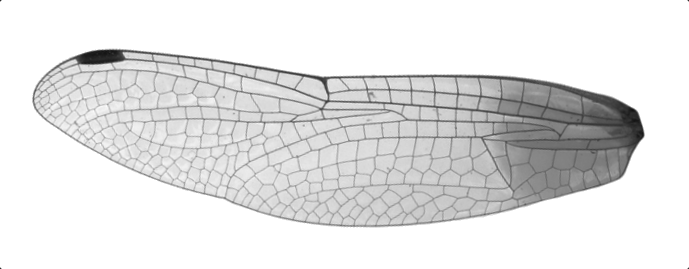

Supplement: Supplementary file 1 — Additional file 1: Trithemis wing images archive. [file 12862_2022_1978_MOESM1_ESM.zip › Additional Files 1/Trithemis Wing Images Archive/Trithemis Wing Images/Forewings/Images (w: Numbers)/183.tif]

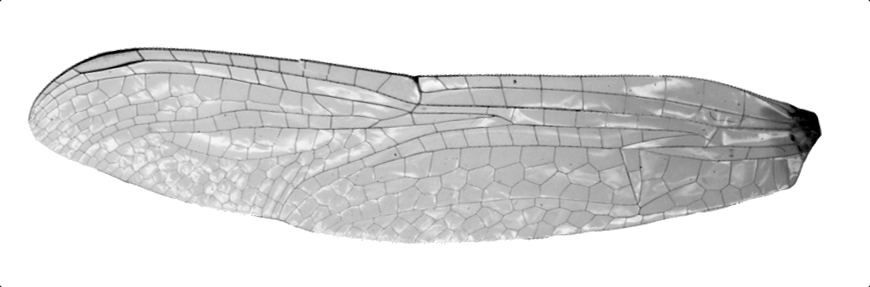

Supplement: Supplementary file 1 — Additional file 1: Trithemis wing images archive. [file 12862_2022_1978_MOESM1_ESM.zip › Additional Files 1/Trithemis Wing Images Archive/Trithemis Wing Images/Forewings/Images (w: Numbers)/154.tif]

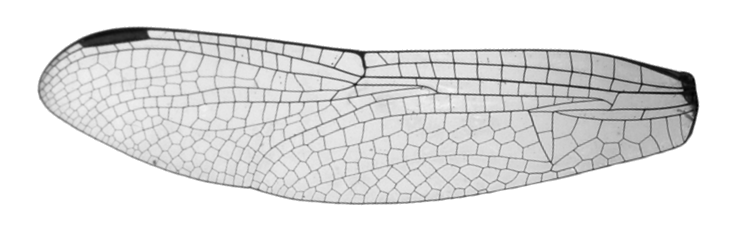

Supplement: Supplementary file 1 — Additional file 1: Trithemis wing images archive. [file 12862_2022_1978_MOESM1_ESM.zip › Additional Files 1/Trithemis Wing Images Archive/Trithemis Wing Images/Forewings/Images (w: Numbers)/140.tif]

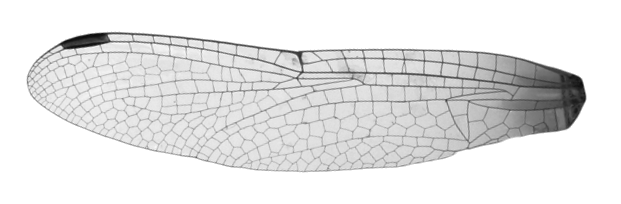

Supplement: Supplementary file 1 — Additional file 1: Trithemis wing images archive. [file 12862_2022_1978_MOESM1_ESM.zip › Additional Files 1/Trithemis Wing Images Archive/Trithemis Wing Images/Forewings/Images (w: Numbers)/168.tif]

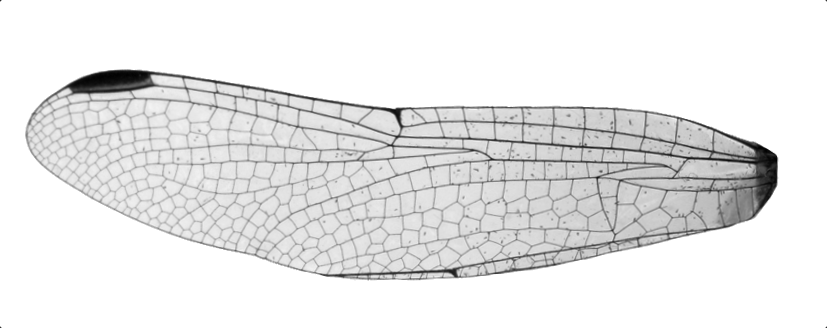

Supplement: Supplementary file 1 — Additional file 1: Trithemis wing images archive. [file 12862_2022_1978_MOESM1_ESM.zip › Additional Files 1/Trithemis Wing Images Archive/Trithemis Wing Images/Forewings/Images (w: Numbers)/127.tif]

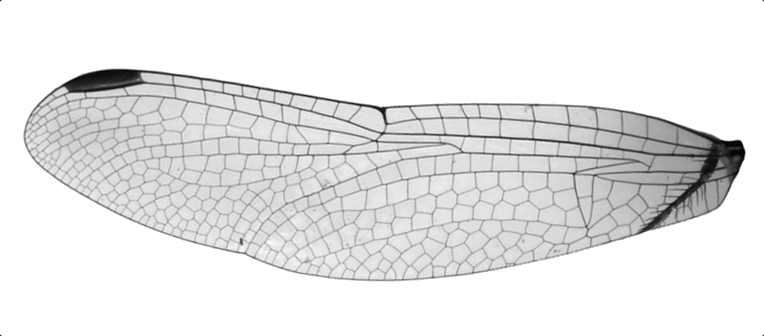

Supplement: Supplementary file 1 — Additional file 1: Trithemis wing images archive. [file 12862_2022_1978_MOESM1_ESM.zip › Additional Files 1/Trithemis Wing Images Archive/Trithemis Wing Images/Forewings/Images (w: Numbers)/133.tif]

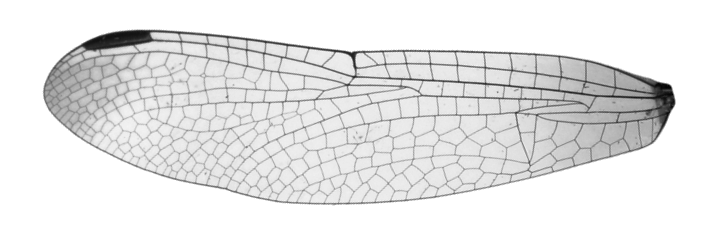

Supplement: Supplementary file 1 — Additional file 1: Trithemis wing images archive. [file 12862_2022_1978_MOESM1_ESM.zip › Additional Files 1/Trithemis Wing Images Archive/Trithemis Wing Images/Forewings/Images (w: Numbers)/053.tif]

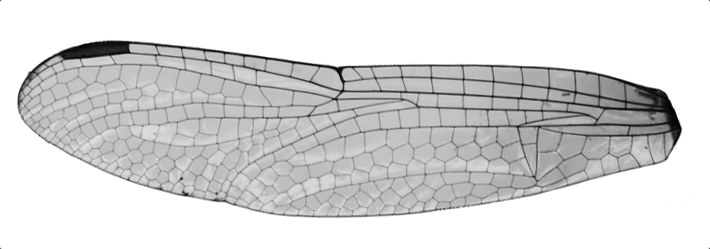

Supplement: Supplementary file 1 — Additional file 1: Trithemis wing images archive. [file 12862_2022_1978_MOESM1_ESM.zip › Additional Files 1/Trithemis Wing Images Archive/Trithemis Wing Images/Forewings/Images (w: Numbers)/047.tif]

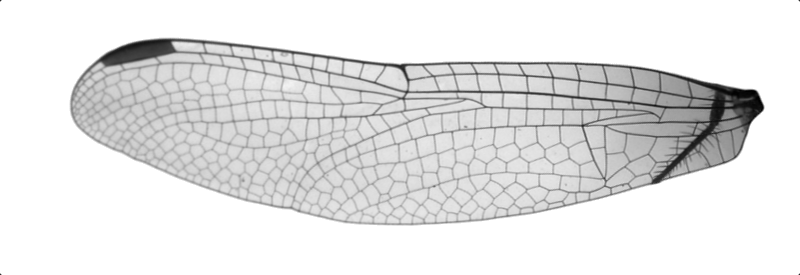

Supplement: Supplementary file 1 — Additional file 1: Trithemis wing images archive. [file 12862_2022_1978_MOESM1_ESM.zip › Additional Files 1/Trithemis Wing Images Archive/Trithemis Wing Images/Forewings/Images (w: Numbers)/251.tif]

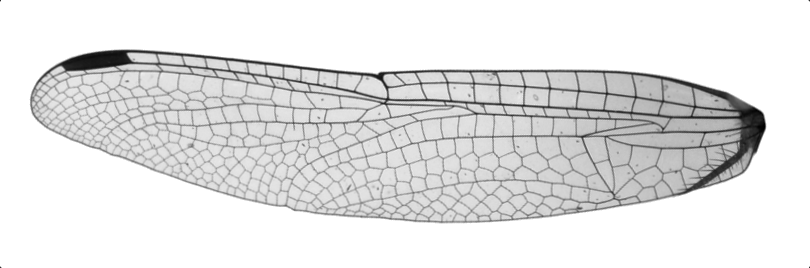

Supplement: Supplementary file 1 — Additional file 1: Trithemis wing images archive. [file 12862_2022_1978_MOESM1_ESM.zip › Additional Files 1/Trithemis Wing Images Archive/Trithemis Wing Images/Forewings/Images (w: Numbers)/245.tif]

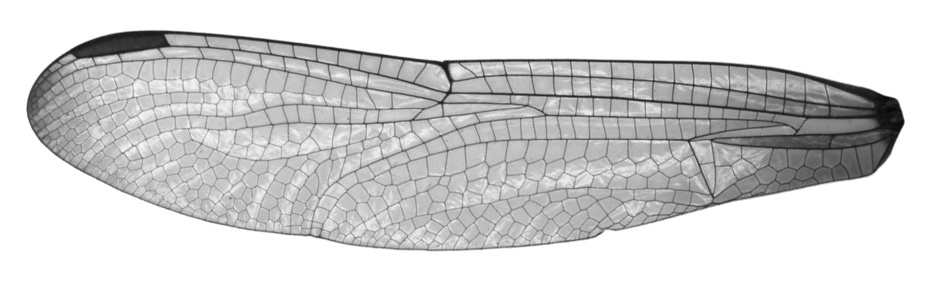

Supplement: Supplementary file 1 — Additional file 1: Trithemis wing images archive. [file 12862_2022_1978_MOESM1_ESM.zip › Additional Files 1/Trithemis Wing Images Archive/Trithemis Wing Images/Forewings/Images (w: Numbers)/279.tif]

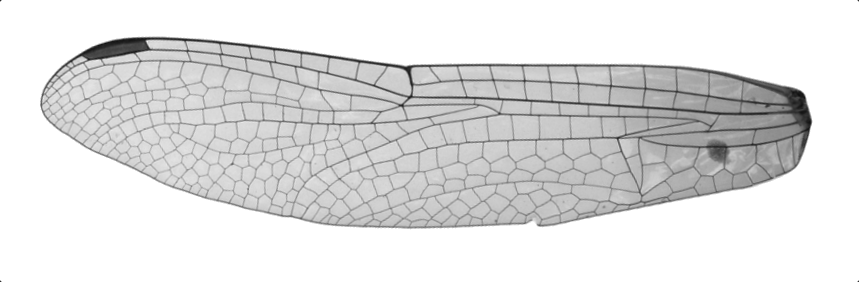

Supplement: Supplementary file 1 — Additional file 1: Trithemis wing images archive. [file 12862_2022_1978_MOESM1_ESM.zip › Additional Files 1/Trithemis Wing Images Archive/Trithemis Wing Images/Forewings/Images (w: Numbers)/286.tif]

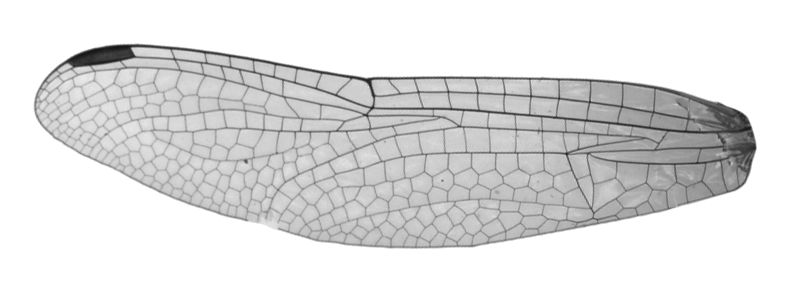

Supplement: Supplementary file 1 — Additional file 1: Trithemis wing images archive. [file 12862_2022_1978_MOESM1_ESM.zip › Additional Files 1/Trithemis Wing Images Archive/Trithemis Wing Images/Forewings/Images (w: Numbers)/287.tif]

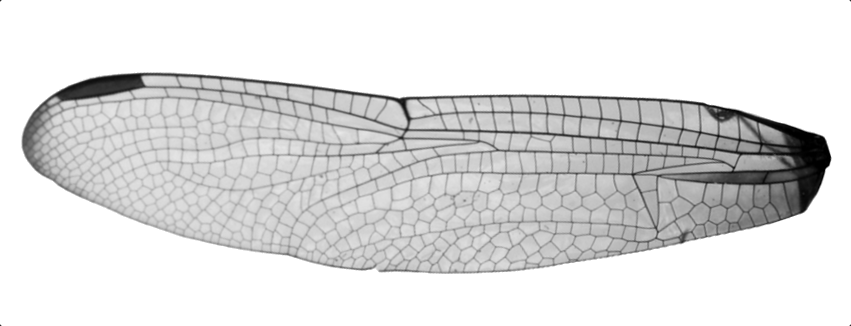

Supplement: Supplementary file 1 — Additional file 1: Trithemis wing images archive. [file 12862_2022_1978_MOESM1_ESM.zip › Additional Files 1/Trithemis Wing Images Archive/Trithemis Wing Images/Forewings/Images (w: Numbers)/278.tif]

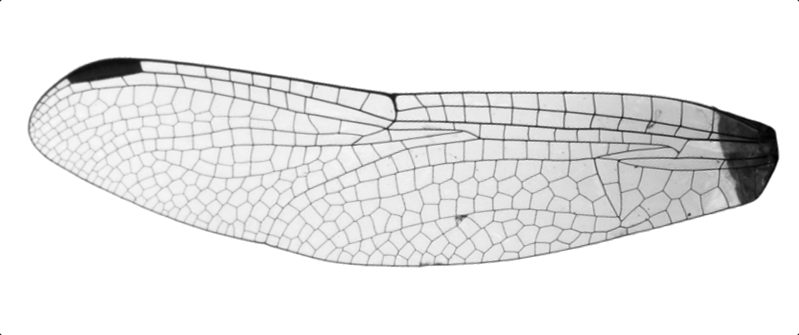

Supplement: Supplementary file 1 — Additional file 1: Trithemis wing images archive. [file 12862_2022_1978_MOESM1_ESM.zip › Additional Files 1/Trithemis Wing Images Archive/Trithemis Wing Images/Forewings/Images (w: Numbers)/244.tif]

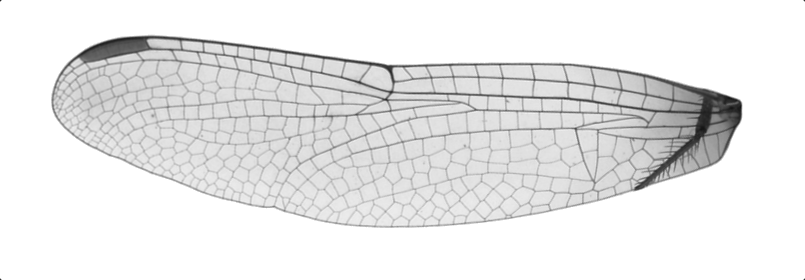

Supplement: Supplementary file 1 — Additional file 1: Trithemis wing images archive. [file 12862_2022_1978_MOESM1_ESM.zip › Additional Files 1/Trithemis Wing Images Archive/Trithemis Wing Images/Forewings/Images (w: Numbers)/250.tif]

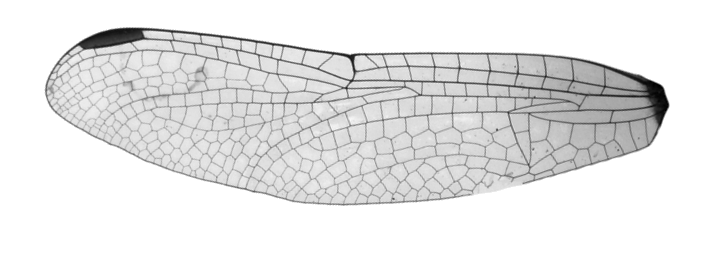

Supplement: Supplementary file 1 — Additional file 1: Trithemis wing images archive. [file 12862_2022_1978_MOESM1_ESM.zip › Additional Files 1/Trithemis Wing Images Archive/Trithemis Wing Images/Forewings/Images (w: Numbers)/052.tif]

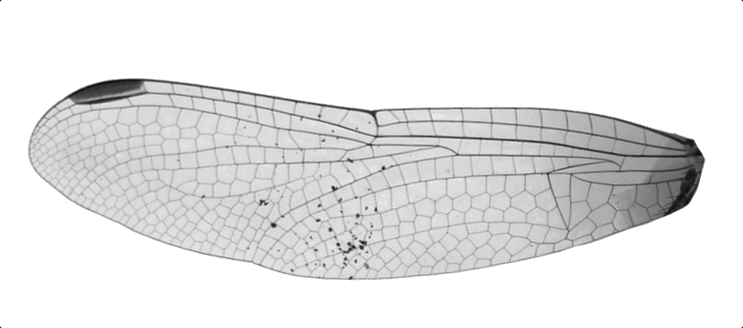

Supplement: Supplementary file 1 — Additional file 1: Trithemis wing images archive. [file 12862_2022_1978_MOESM1_ESM.zip › Additional Files 1/Trithemis Wing Images Archive/Trithemis Wing Images/Forewings/Images (w: Numbers)/132.tif]

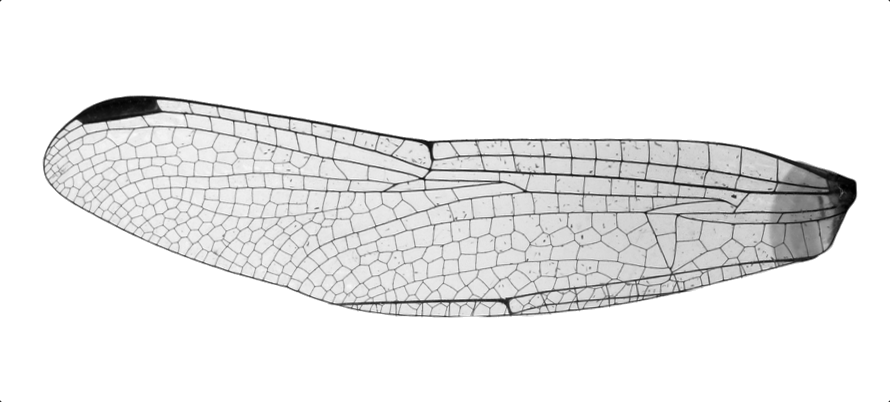

Supplement: Supplementary file 1 — Additional file 1: Trithemis wing images archive. [file 12862_2022_1978_MOESM1_ESM.zip › Additional Files 1/Trithemis Wing Images Archive/Trithemis Wing Images/Forewings/Images (w: Numbers)/126.tif]

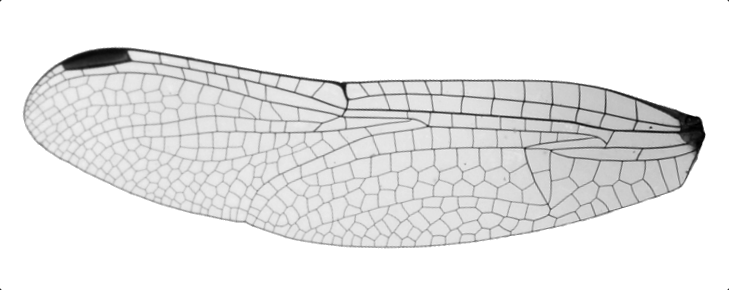

Supplement: Supplementary file 1 — Additional file 1: Trithemis wing images archive. [file 12862_2022_1978_MOESM1_ESM.zip › Additional Files 1/Trithemis Wing Images Archive/Trithemis Wing Images/Forewings/Images (w: Numbers)/130.tif]

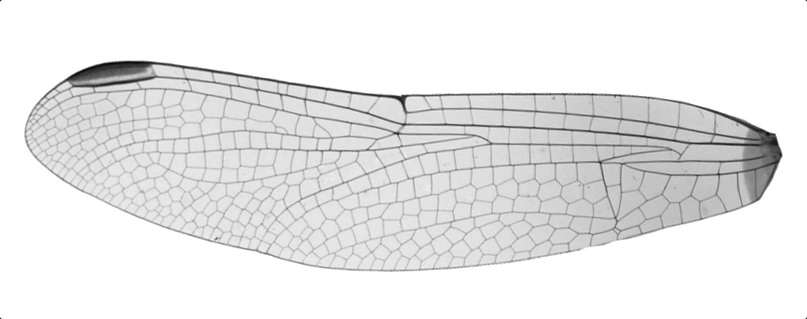

Supplement: Supplementary file 1 — Additional file 1: Trithemis wing images archive. [file 12862_2022_1978_MOESM1_ESM.zip › Additional Files 1/Trithemis Wing Images Archive/Trithemis Wing Images/Forewings/Images (w: Numbers)/124.tif]

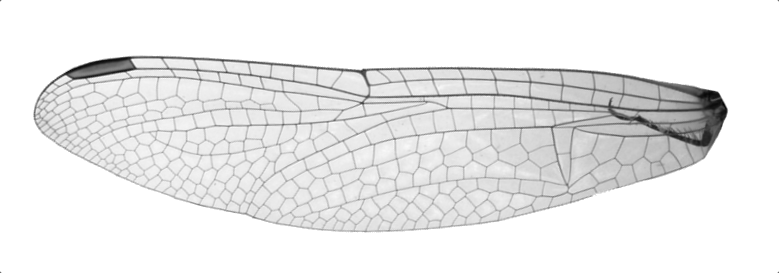

Supplement: Supplementary file 1 — Additional file 1: Trithemis wing images archive. [file 12862_2022_1978_MOESM1_ESM.zip › Additional Files 1/Trithemis Wing Images Archive/Trithemis Wing Images/Forewings/Images (w: Numbers)/044.tif]

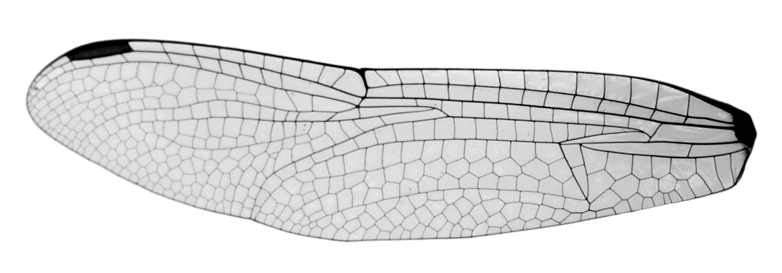

Supplement: Supplementary file 1 — Additional file 1: Trithemis wing images archive. [file 12862_2022_1978_MOESM1_ESM.zip › Additional Files 1/Trithemis Wing Images Archive/Trithemis Wing Images/Forewings/Images (w: Numbers)/078.tif]

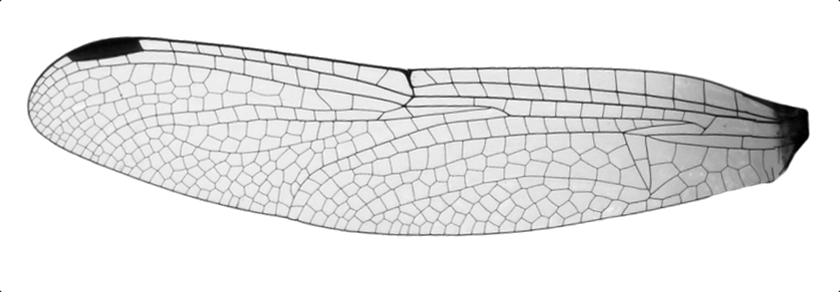

Supplement: Supplementary file 1 — Additional file 1: Trithemis wing images archive. [file 12862_2022_1978_MOESM1_ESM.zip › Additional Files 1/Trithemis Wing Images Archive/Trithemis Wing Images/Forewings/Images (w: Numbers)/246.tif]

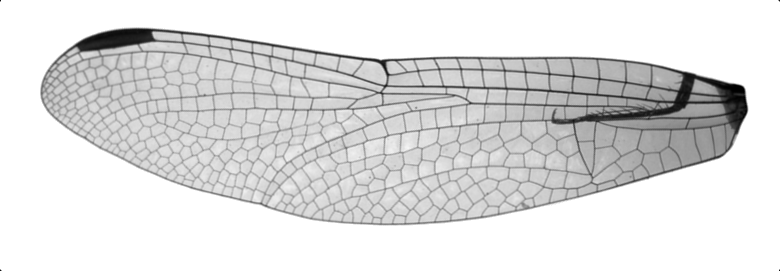

Supplement: Supplementary file 1 — Additional file 1: Trithemis wing images archive. [file 12862_2022_1978_MOESM1_ESM.zip › Additional Files 1/Trithemis Wing Images Archive/Trithemis Wing Images/Forewings/Images (w: Numbers)/252.tif]

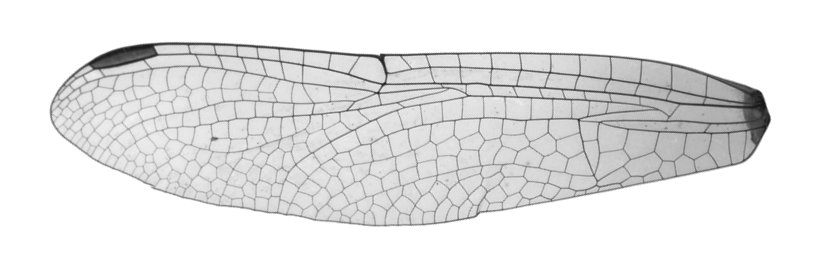

Supplement: Supplementary file 1 — Additional file 1: Trithemis wing images archive. [file 12862_2022_1978_MOESM1_ESM.zip › Additional Files 1/Trithemis Wing Images Archive/Trithemis Wing Images/Forewings/Images (w: Numbers)/285.tif]

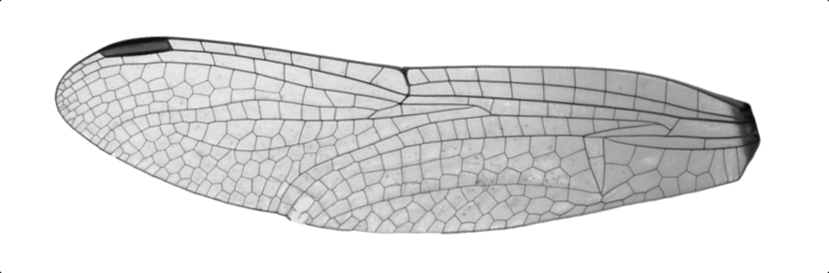

Supplement: Supplementary file 1 — Additional file 1: Trithemis wing images archive. [file 12862_2022_1978_MOESM1_ESM.zip › Additional Files 1/Trithemis Wing Images Archive/Trithemis Wing Images/Forewings/Images (w: Numbers)/284.tif]

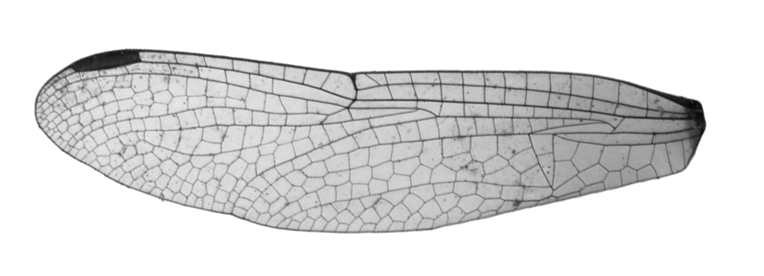

Supplement: Supplementary file 1 — Additional file 1: Trithemis wing images archive. [file 12862_2022_1978_MOESM1_ESM.zip › Additional Files 1/Trithemis Wing Images Archive/Trithemis Wing Images/Forewings/Images (w: Numbers)/253.tif]

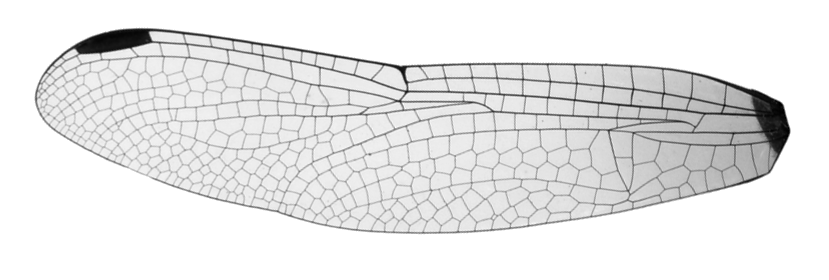

Supplement: Supplementary file 1 — Additional file 1: Trithemis wing images archive. [file 12862_2022_1978_MOESM1_ESM.zip › Additional Files 1/Trithemis Wing Images Archive/Trithemis Wing Images/Forewings/Images (w: Numbers)/247.tif]

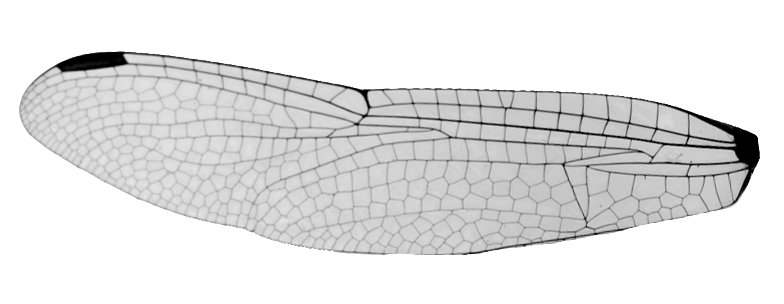

Supplement: Supplementary file 1 — Additional file 1: Trithemis wing images archive. [file 12862_2022_1978_MOESM1_ESM.zip › Additional Files 1/Trithemis Wing Images Archive/Trithemis Wing Images/Forewings/Images (w: Numbers)/079.tif]

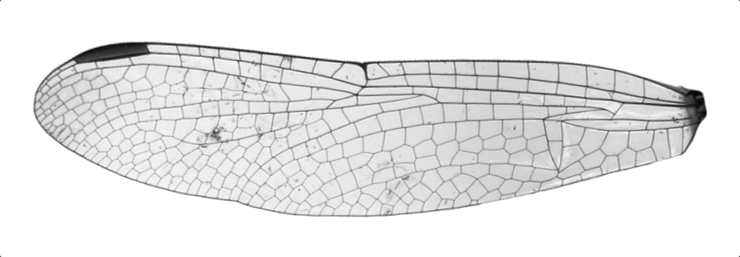

Supplement: Supplementary file 1 — Additional file 1: Trithemis wing images archive. [file 12862_2022_1978_MOESM1_ESM.zip › Additional Files 1/Trithemis Wing Images Archive/Trithemis Wing Images/Forewings/Images (w: Numbers)/051.tif]

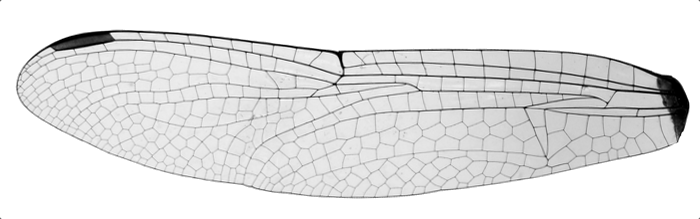

Supplement: Supplementary file 1 — Additional file 1: Trithemis wing images archive. [file 12862_2022_1978_MOESM1_ESM.zip › Additional Files 1/Trithemis Wing Images Archive/Trithemis Wing Images/Forewings/Images (w: Numbers)/045.tif]

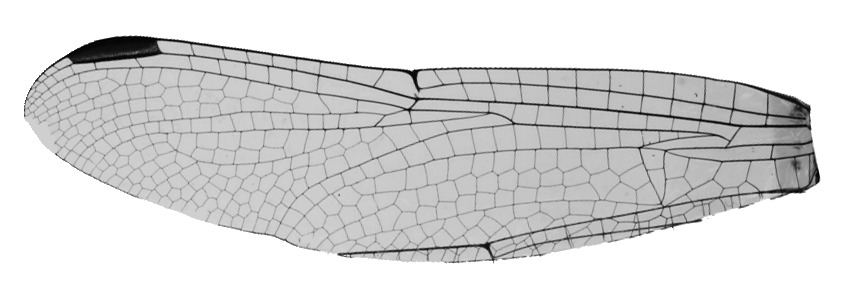

Supplement: Supplementary file 1 — Additional file 1: Trithemis wing images archive. [file 12862_2022_1978_MOESM1_ESM.zip › Additional Files 1/Trithemis Wing Images Archive/Trithemis Wing Images/Forewings/Images (w: Numbers)/119.tif]

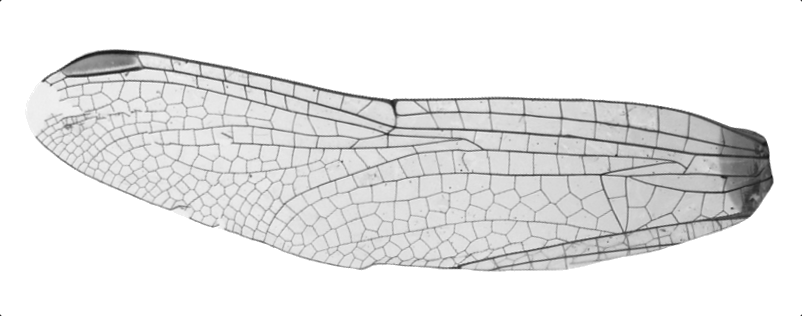

Supplement: Supplementary file 1 — Additional file 1: Trithemis wing images archive. [file 12862_2022_1978_MOESM1_ESM.zip › Additional Files 1/Trithemis Wing Images Archive/Trithemis Wing Images/Forewings/Images (w: Numbers)/125.tif]

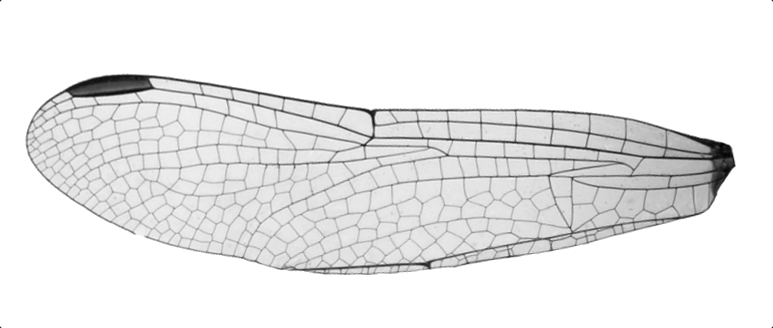

Supplement: Supplementary file 1 — Additional file 1: Trithemis wing images archive. [file 12862_2022_1978_MOESM1_ESM.zip › Additional Files 1/Trithemis Wing Images Archive/Trithemis Wing Images/Forewings/Images (w: Numbers)/131.tif]

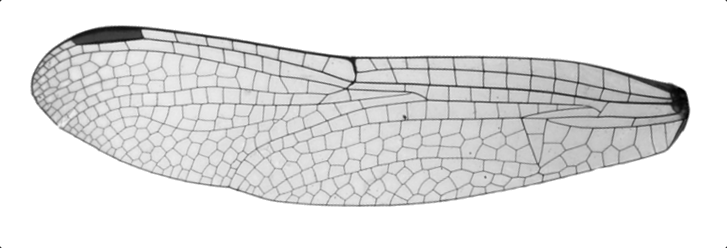

Supplement: Supplementary file 1 — Additional file 1: Trithemis wing images archive. [file 12862_2022_1978_MOESM1_ESM.zip › Additional Files 1/Trithemis Wing Images Archive/Trithemis Wing Images/Forewings/Images (w: Numbers)/135.tif]

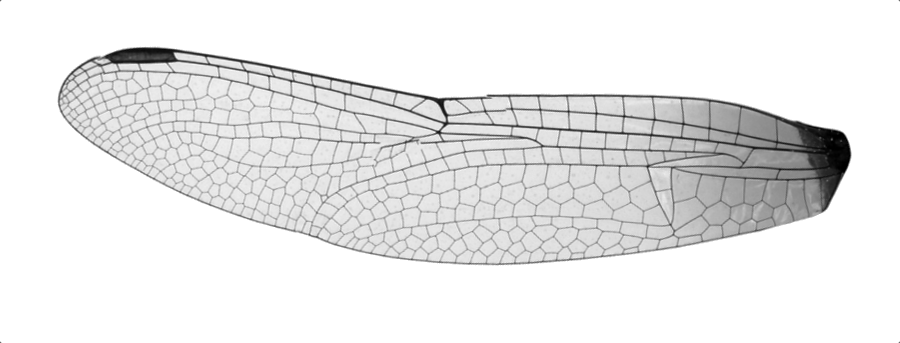

Supplement: Supplementary file 1 — Additional file 1: Trithemis wing images archive. [file 12862_2022_1978_MOESM1_ESM.zip › Additional Files 1/Trithemis Wing Images Archive/Trithemis Wing Images/Forewings/Images (w: Numbers)/069.tif]

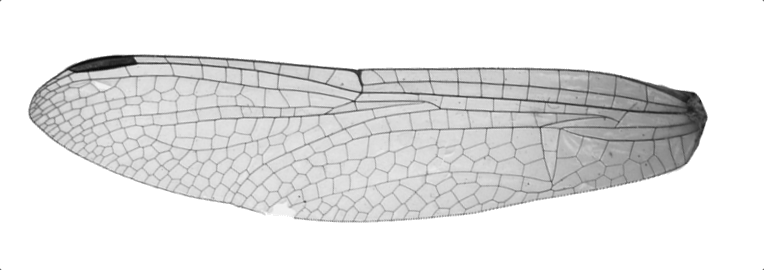

Supplement: Supplementary file 1 — Additional file 1: Trithemis wing images archive. [file 12862_2022_1978_MOESM1_ESM.zip › Additional Files 1/Trithemis Wing Images Archive/Trithemis Wing Images/Forewings/Images (w: Numbers)/041.tif]

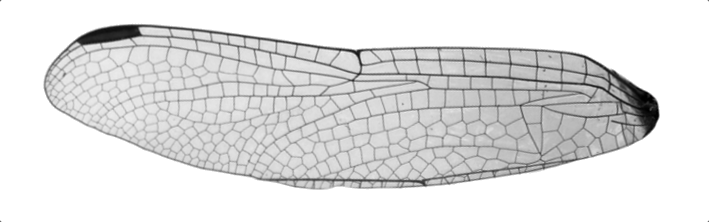

Supplement: Supplementary file 1 — Additional file 1: Trithemis wing images archive. [file 12862_2022_1978_MOESM1_ESM.zip › Additional Files 1/Trithemis Wing Images Archive/Trithemis Wing Images/Forewings/Images (w: Numbers)/055.tif]

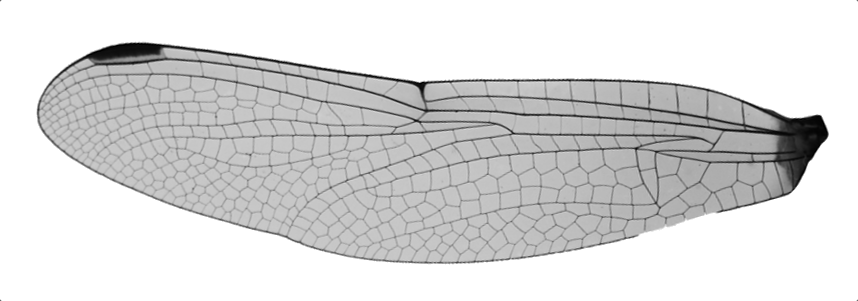

Supplement: Supplementary file 1 — Additional file 1: Trithemis wing images archive. [file 12862_2022_1978_MOESM1_ESM.zip › Additional Files 1/Trithemis Wing Images Archive/Trithemis Wing Images/Forewings/Images (w: Numbers)/096.tif]

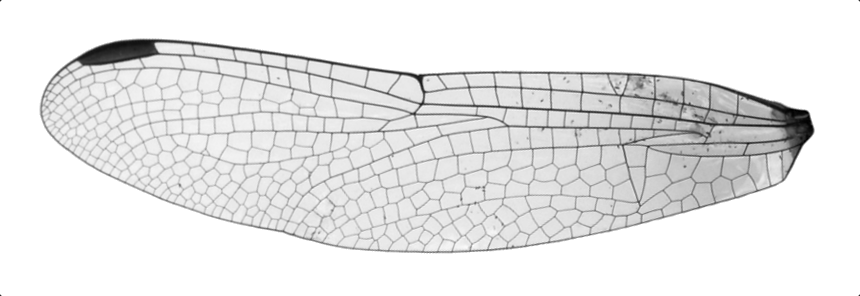

Supplement: Supplementary file 1 — Additional file 1: Trithemis wing images archive. [file 12862_2022_1978_MOESM1_ESM.zip › Additional Files 1/Trithemis Wing Images Archive/Trithemis Wing Images/Forewings/Images (w: Numbers)/243.tif]

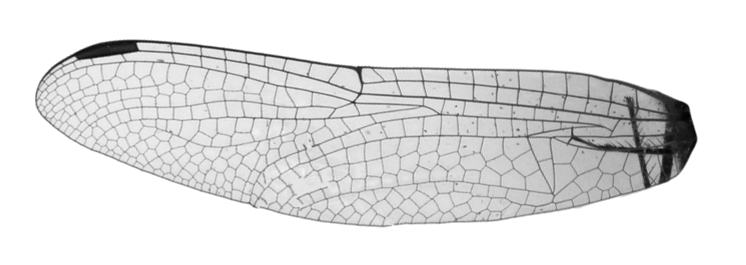

Supplement: Supplementary file 1 — Additional file 1: Trithemis wing images archive. [file 12862_2022_1978_MOESM1_ESM.zip › Additional Files 1/Trithemis Wing Images Archive/Trithemis Wing Images/Forewings/Images (w: Numbers)/257.tif]

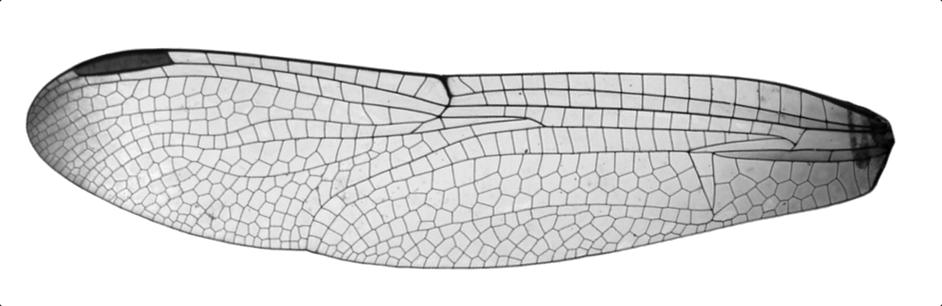

Supplement: Supplementary file 1 — Additional file 1: Trithemis wing images archive. [file 12862_2022_1978_MOESM1_ESM.zip › Additional Files 1/Trithemis Wing Images Archive/Trithemis Wing Images/Forewings/Images (w: Numbers)/280.tif]

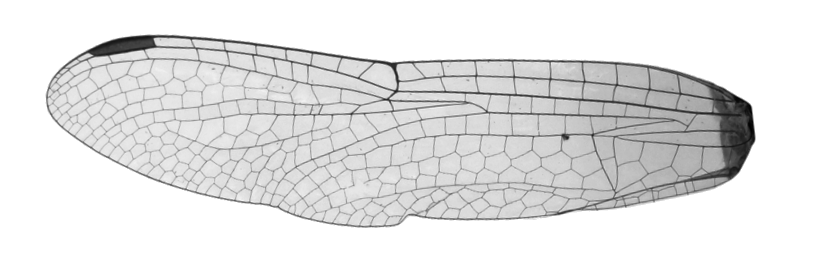

Supplement: Supplementary file 1 — Additional file 1: Trithemis wing images archive. [file 12862_2022_1978_MOESM1_ESM.zip › Additional Files 1/Trithemis Wing Images Archive/Trithemis Wing Images/Forewings/Images (w: Numbers)/281.tif]

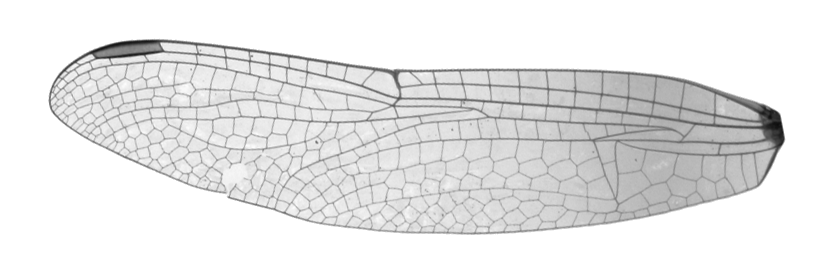

Supplement: Supplementary file 1 — Additional file 1: Trithemis wing images archive. [file 12862_2022_1978_MOESM1_ESM.zip › Additional Files 1/Trithemis Wing Images Archive/Trithemis Wing Images/Forewings/Images (w: Numbers)/256.tif]

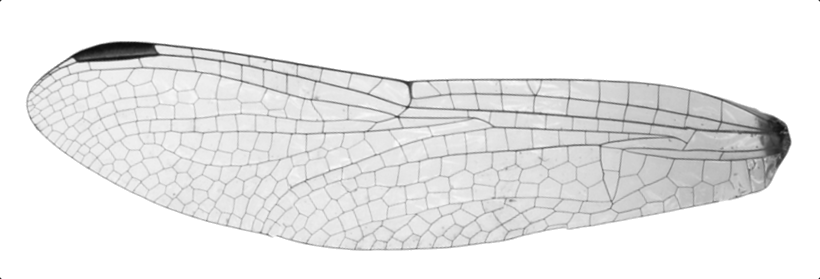

Supplement: Supplementary file 1 — Additional file 1: Trithemis wing images archive. [file 12862_2022_1978_MOESM1_ESM.zip › Additional Files 1/Trithemis Wing Images Archive/Trithemis Wing Images/Forewings/Images (w: Numbers)/242.tif]

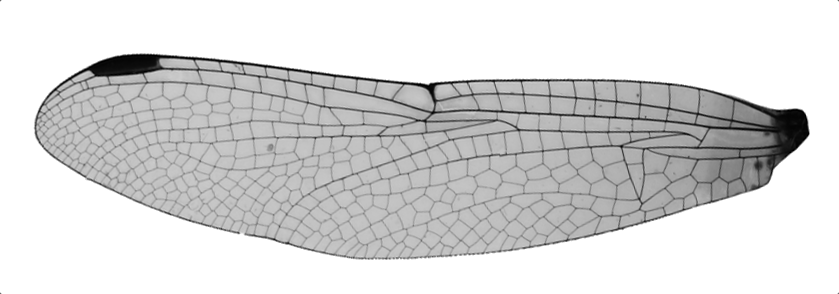

Supplement: Supplementary file 1 — Additional file 1: Trithemis wing images archive. [file 12862_2022_1978_MOESM1_ESM.zip › Additional Files 1/Trithemis Wing Images Archive/Trithemis Wing Images/Forewings/Images (w: Numbers)/097.tif]

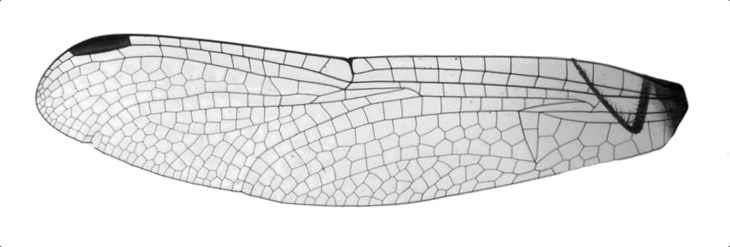

Supplement: Supplementary file 1 — Additional file 1: Trithemis wing images archive. [file 12862_2022_1978_MOESM1_ESM.zip › Additional Files 1/Trithemis Wing Images Archive/Trithemis Wing Images/Forewings/Images (w: Numbers)/054.tif]

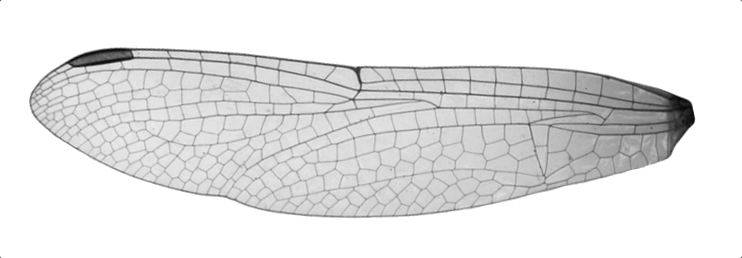

Supplement: Supplementary file 1 — Additional file 1: Trithemis wing images archive. [file 12862_2022_1978_MOESM1_ESM.zip › Additional Files 1/Trithemis Wing Images Archive/Trithemis Wing Images/Forewings/Images (w: Numbers)/040.tif]

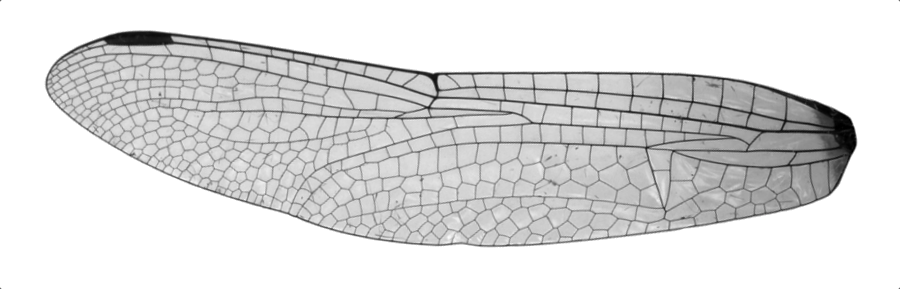

Supplement: Supplementary file 1 — Additional file 1: Trithemis wing images archive. [file 12862_2022_1978_MOESM1_ESM.zip › Additional Files 1/Trithemis Wing Images Archive/Trithemis Wing Images/Forewings/Images (w: Numbers)/068.tif]

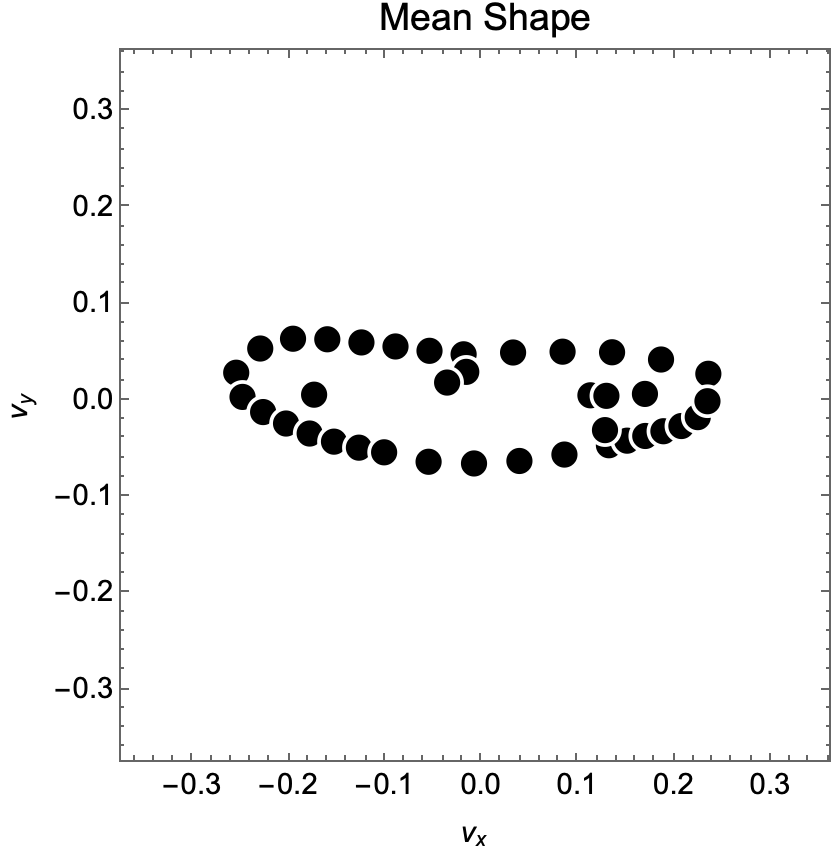

Supplement: Supplementary file 2 — Additional file 2: Datasets and results archive. [file 12862_2022_1978_MOESM2_ESM.zip › Additional Files 2/Datasets & Results Archive/Geometric Morphometrics (Landmarks) Analyses/Forewings/Mean Shape Plot.tif]
