# Supplementary figures and images for: What you sample is what you get: ecomorphological variation in Trithemis (Odonata, Libellulidae) dragonfly wings reconsidered (part 1 of 3)
Source: BMC Ecol Evol. 2022 Apr 11;22:43. doi: 10.1186/s12862-022-01978-y (PMC8996507; doi:10.1186/s12862-022-01978-y)

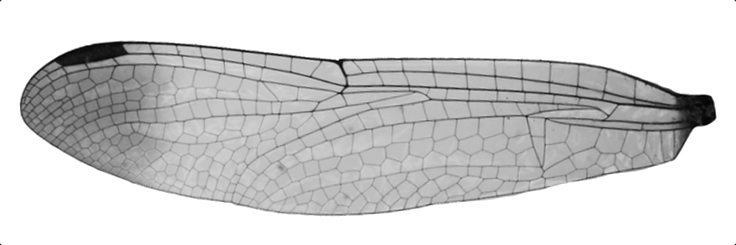

Supplement: Supplementary file 1 — Additional file 1: Trithemis wing images archive. [file 12862_2022_1978_MOESM1_ESM.zip › Additional Files 1/Trithemis Wing Images Archive/Trithemis Wing Images/Forewings/Images (w: Numbers)/106.tif]

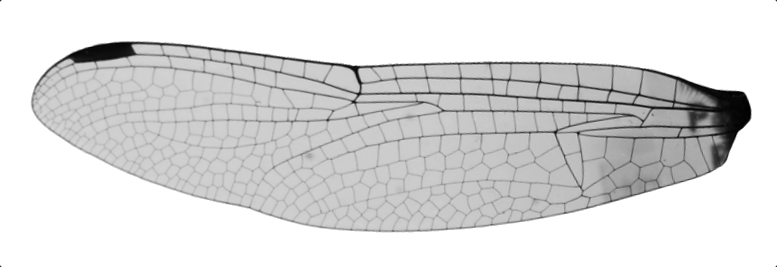

Supplement: Supplementary file 1 — Additional file 1: Trithemis wing images archive. [file 12862_2022_1978_MOESM1_ESM.zip › Additional Files 1/Trithemis Wing Images Archive/Trithemis Wing Images/Forewings/Images (w: Numbers)/112.tif]

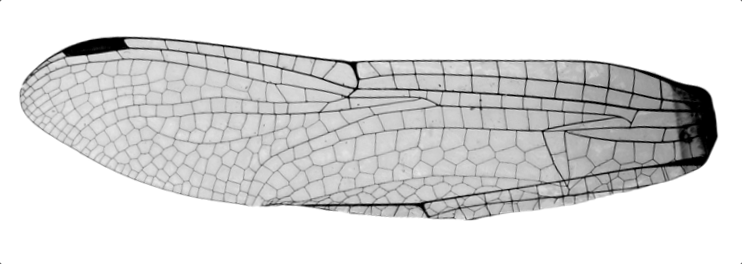

Supplement: Supplementary file 1 — Additional file 1: Trithemis wing images archive. [file 12862_2022_1978_MOESM1_ESM.zip › Additional Files 1/Trithemis Wing Images Archive/Trithemis Wing Images/Forewings/Images (w: Numbers)/072.tif]

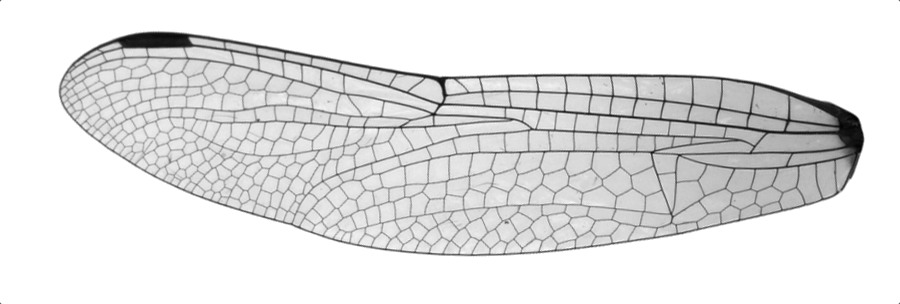

Supplement: Supplementary file 1 — Additional file 1: Trithemis wing images archive. [file 12862_2022_1978_MOESM1_ESM.zip › Additional Files 1/Trithemis Wing Images Archive/Trithemis Wing Images/Forewings/Images (w: Numbers)/066.tif]

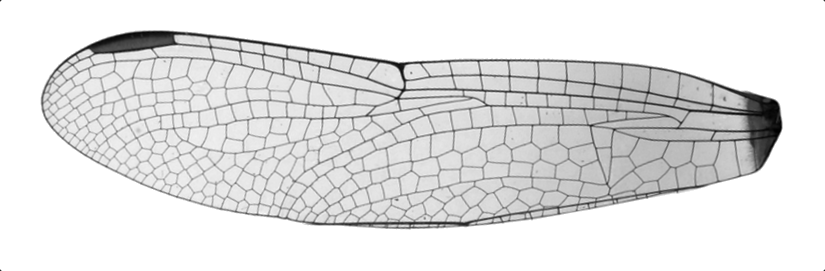

Supplement: Supplementary file 1 — Additional file 1: Trithemis wing images archive. [file 12862_2022_1978_MOESM1_ESM.zip › Additional Files 1/Trithemis Wing Images Archive/Trithemis Wing Images/Forewings/Images (w: Numbers)/270.tif]

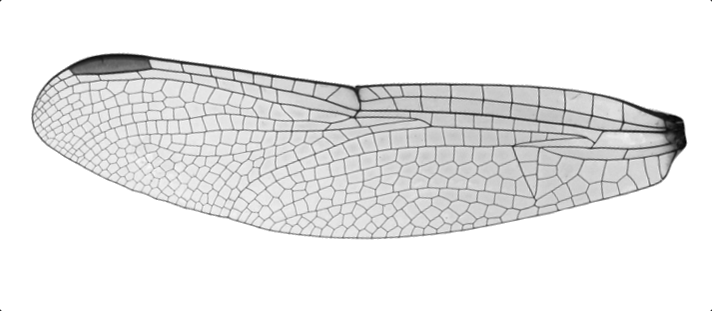

Supplement: Supplementary file 1 — Additional file 1: Trithemis wing images archive. [file 12862_2022_1978_MOESM1_ESM.zip › Additional Files 1/Trithemis Wing Images Archive/Trithemis Wing Images/Forewings/Images (w: Numbers)/264.tif]

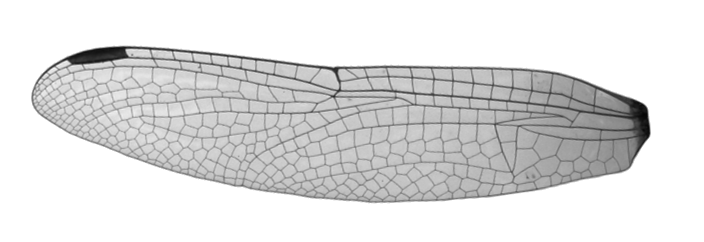

Supplement: Supplementary file 1 — Additional file 1: Trithemis wing images archive. [file 12862_2022_1978_MOESM1_ESM.zip › Additional Files 1/Trithemis Wing Images Archive/Trithemis Wing Images/Forewings/Images (w: Numbers)/258.tif]

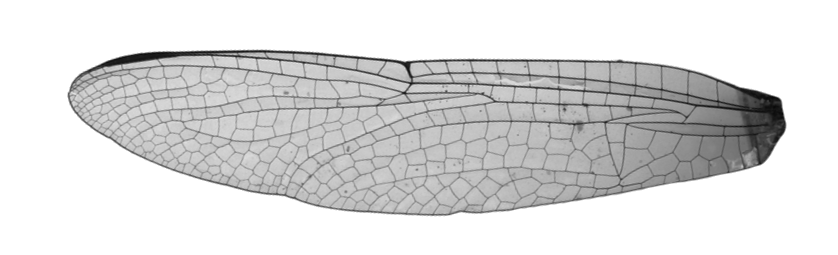

Supplement: Supplementary file 1 — Additional file 1: Trithemis wing images archive. [file 12862_2022_1978_MOESM1_ESM.zip › Additional Files 1/Trithemis Wing Images Archive/Trithemis Wing Images/Forewings/Images (w: Numbers)/259.tif]

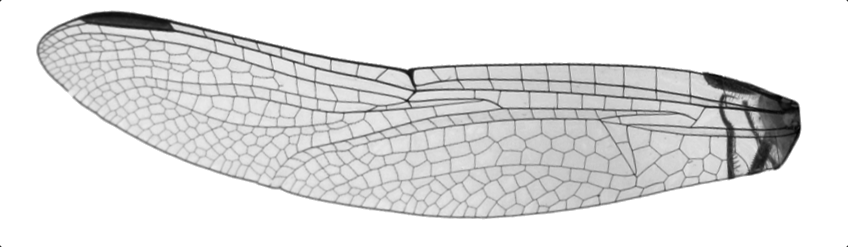

Supplement: Supplementary file 1 — Additional file 1: Trithemis wing images archive. [file 12862_2022_1978_MOESM1_ESM.zip › Additional Files 1/Trithemis Wing Images Archive/Trithemis Wing Images/Forewings/Images (w: Numbers)/265.tif]

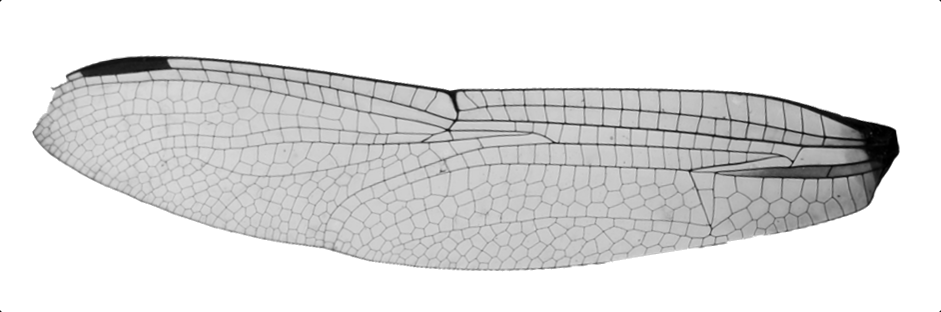

Supplement: Supplementary file 1 — Additional file 1: Trithemis wing images archive. [file 12862_2022_1978_MOESM1_ESM.zip › Additional Files 1/Trithemis Wing Images Archive/Trithemis Wing Images/Forewings/Images (w: Numbers)/271.tif]

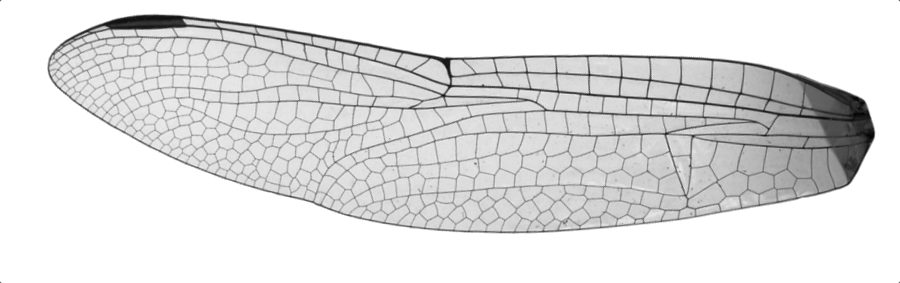

Supplement: Supplementary file 1 — Additional file 1: Trithemis wing images archive. [file 12862_2022_1978_MOESM1_ESM.zip › Additional Files 1/Trithemis Wing Images Archive/Trithemis Wing Images/Forewings/Images (w: Numbers)/067.tif]

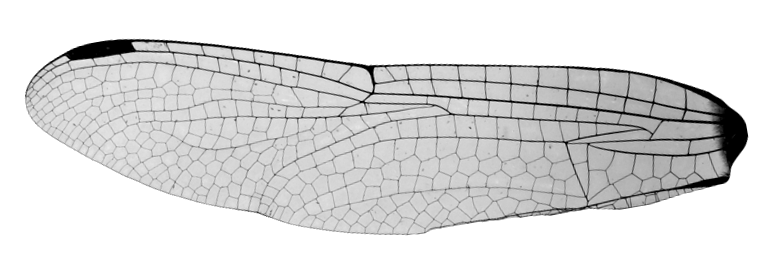

Supplement: Supplementary file 1 — Additional file 1: Trithemis wing images archive. [file 12862_2022_1978_MOESM1_ESM.zip › Additional Files 1/Trithemis Wing Images Archive/Trithemis Wing Images/Forewings/Images (w: Numbers)/073.tif]

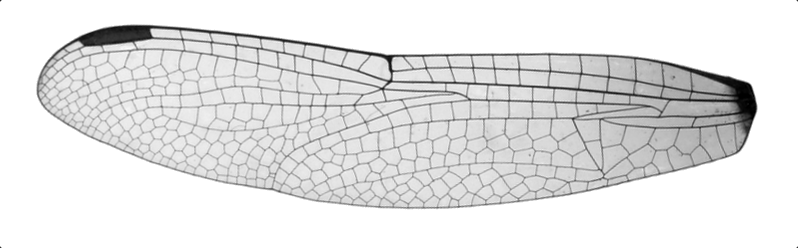

Supplement: Supplementary file 1 — Additional file 1: Trithemis wing images archive. [file 12862_2022_1978_MOESM1_ESM.zip › Additional Files 1/Trithemis Wing Images Archive/Trithemis Wing Images/Forewings/Images (w: Numbers)/139.tif]

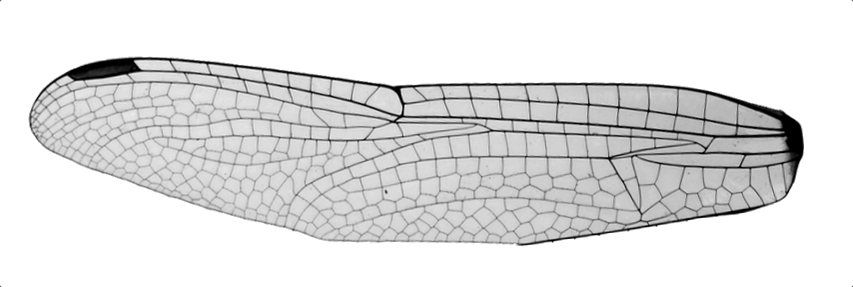

Supplement: Supplementary file 1 — Additional file 1: Trithemis wing images archive. [file 12862_2022_1978_MOESM1_ESM.zip › Additional Files 1/Trithemis Wing Images Archive/Trithemis Wing Images/Forewings/Images (w: Numbers)/071.tif]

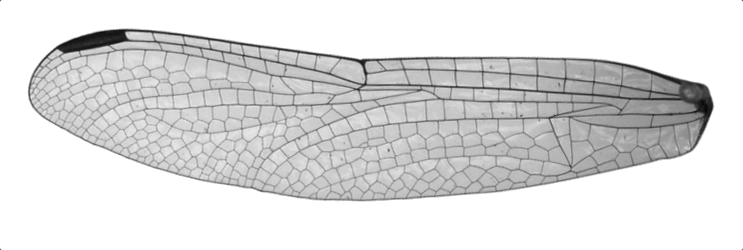

Supplement: Supplementary file 1 — Additional file 1: Trithemis wing images archive. [file 12862_2022_1978_MOESM1_ESM.zip › Additional Files 1/Trithemis Wing Images Archive/Trithemis Wing Images/Forewings/Images (w: Numbers)/059.tif]

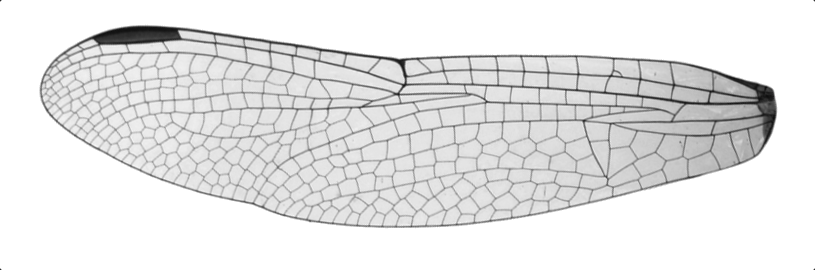

Supplement: Supplementary file 1 — Additional file 1: Trithemis wing images archive. [file 12862_2022_1978_MOESM1_ESM.zip › Additional Files 1/Trithemis Wing Images Archive/Trithemis Wing Images/Forewings/Images (w: Numbers)/267.tif]

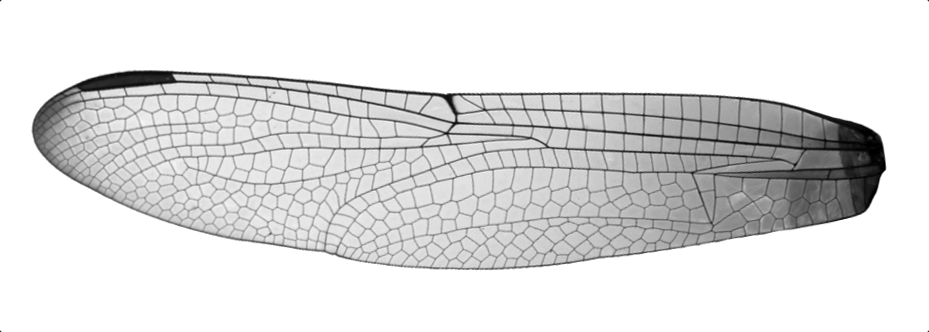

Supplement: Supplementary file 1 — Additional file 1: Trithemis wing images archive. [file 12862_2022_1978_MOESM1_ESM.zip › Additional Files 1/Trithemis Wing Images Archive/Trithemis Wing Images/Forewings/Images (w: Numbers)/273.tif]

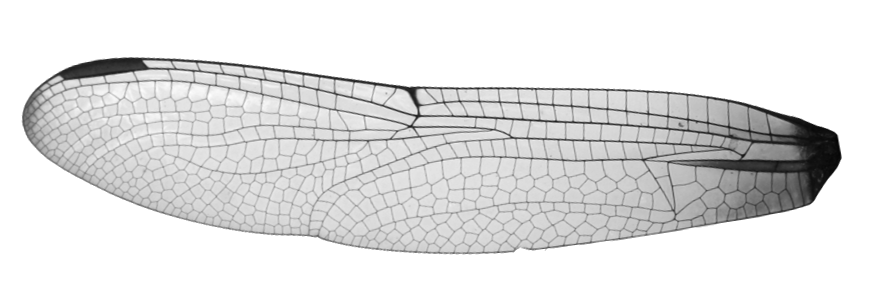

Supplement: Supplementary file 1 — Additional file 1: Trithemis wing images archive. [file 12862_2022_1978_MOESM1_ESM.zip › Additional Files 1/Trithemis Wing Images Archive/Trithemis Wing Images/Forewings/Images (w: Numbers)/272.tif]

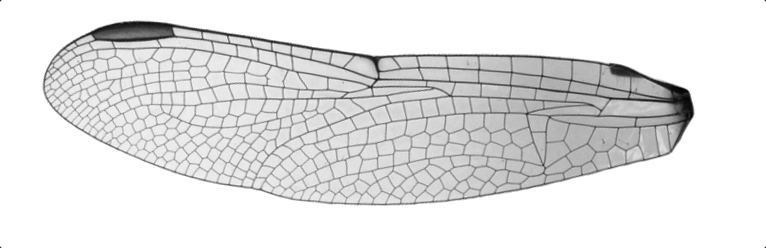

Supplement: Supplementary file 1 — Additional file 1: Trithemis wing images archive. [file 12862_2022_1978_MOESM1_ESM.zip › Additional Files 1/Trithemis Wing Images Archive/Trithemis Wing Images/Forewings/Images (w: Numbers)/266.tif]

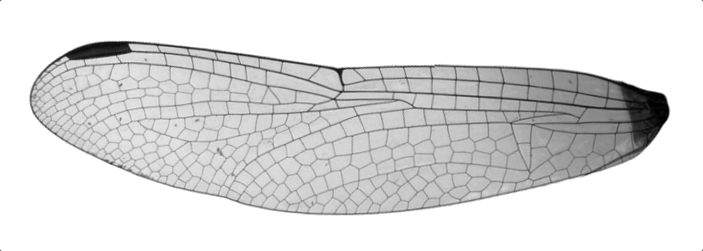

Supplement: Supplementary file 1 — Additional file 1: Trithemis wing images archive. [file 12862_2022_1978_MOESM1_ESM.zip › Additional Files 1/Trithemis Wing Images Archive/Trithemis Wing Images/Forewings/Images (w: Numbers)/058.tif]

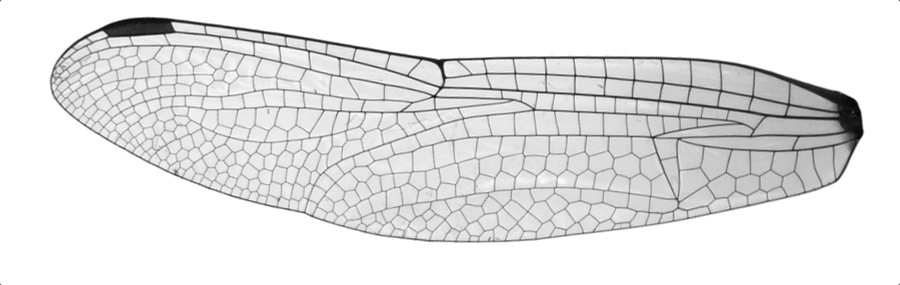

Supplement: Supplementary file 1 — Additional file 1: Trithemis wing images archive. [file 12862_2022_1978_MOESM1_ESM.zip › Additional Files 1/Trithemis Wing Images Archive/Trithemis Wing Images/Forewings/Images (w: Numbers)/070.tif]

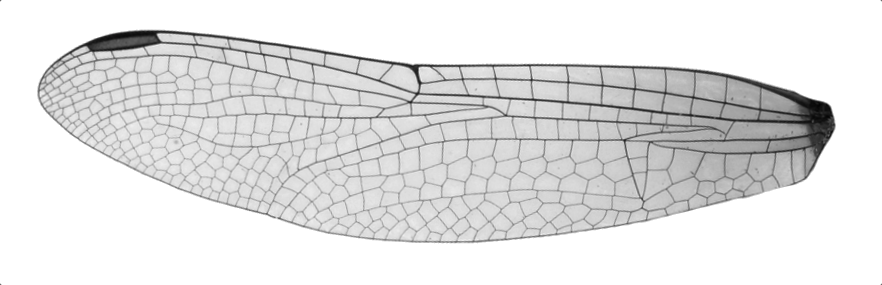

Supplement: Supplementary file 1 — Additional file 1: Trithemis wing images archive. [file 12862_2022_1978_MOESM1_ESM.zip › Additional Files 1/Trithemis Wing Images Archive/Trithemis Wing Images/Forewings/Images (w: Numbers)/064.tif]

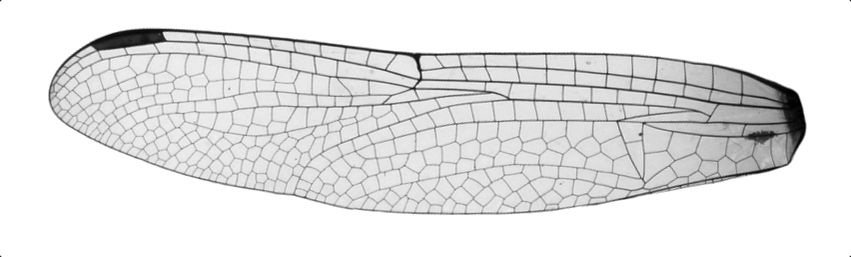

Supplement: Supplementary file 1 — Additional file 1: Trithemis wing images archive. [file 12862_2022_1978_MOESM1_ESM.zip › Additional Files 1/Trithemis Wing Images Archive/Trithemis Wing Images/Forewings/Images (w: Numbers)/138.tif]

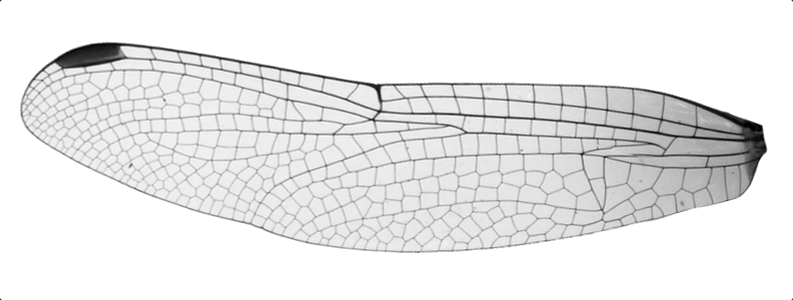

Supplement: Supplementary file 1 — Additional file 1: Trithemis wing images archive. [file 12862_2022_1978_MOESM1_ESM.zip › Additional Files 1/Trithemis Wing Images Archive/Trithemis Wing Images/Forewings/Images (w: Numbers)/128.tif]

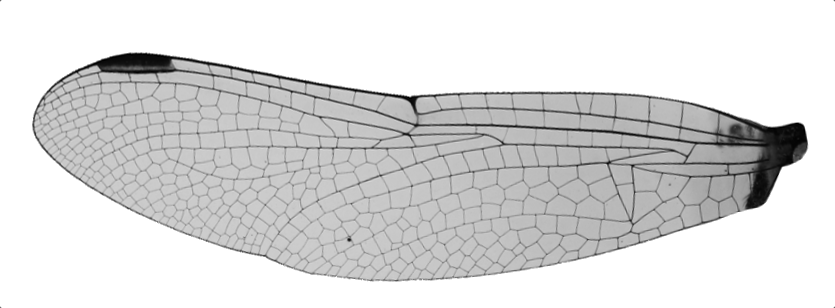

Supplement: Supplementary file 1 — Additional file 1: Trithemis wing images archive. [file 12862_2022_1978_MOESM1_ESM.zip › Additional Files 1/Trithemis Wing Images Archive/Trithemis Wing Images/Forewings/Images (w: Numbers)/100.tif]

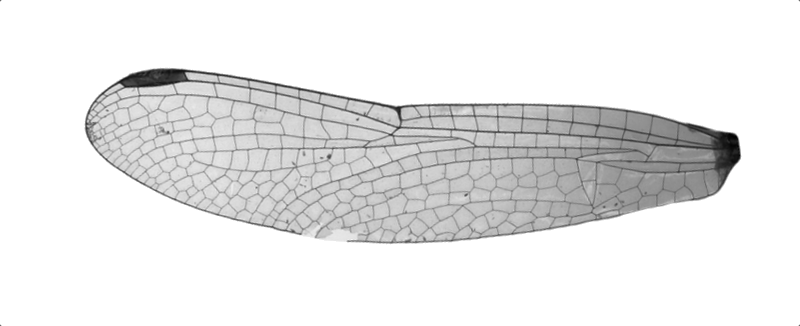

Supplement: Supplementary file 1 — Additional file 1: Trithemis wing images archive. [file 12862_2022_1978_MOESM1_ESM.zip › Additional Files 1/Trithemis Wing Images Archive/Trithemis Wing Images/Forewings/Images (w: Numbers)/060.tif]

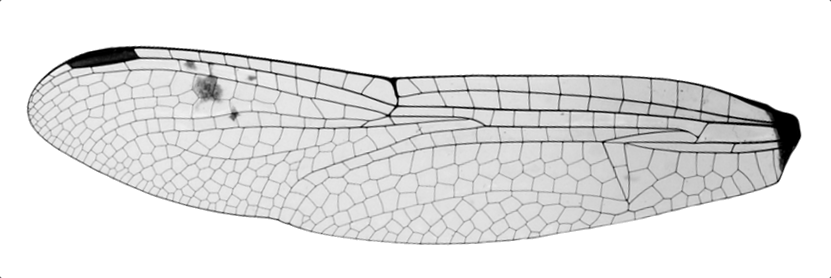

Supplement: Supplementary file 1 — Additional file 1: Trithemis wing images archive. [file 12862_2022_1978_MOESM1_ESM.zip › Additional Files 1/Trithemis Wing Images Archive/Trithemis Wing Images/Forewings/Images (w: Numbers)/074.tif]

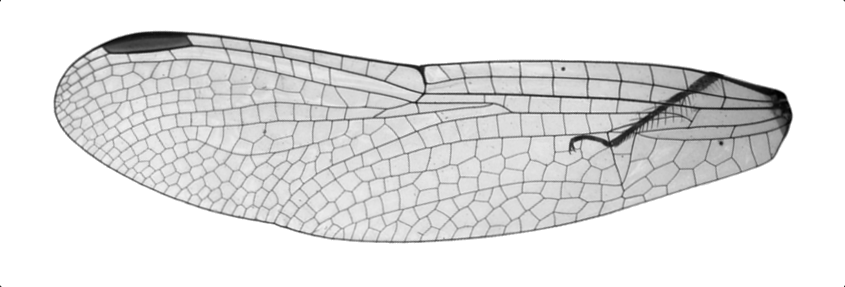

Supplement: Supplementary file 1 — Additional file 1: Trithemis wing images archive. [file 12862_2022_1978_MOESM1_ESM.zip › Additional Files 1/Trithemis Wing Images Archive/Trithemis Wing Images/Forewings/Images (w: Numbers)/262.tif]

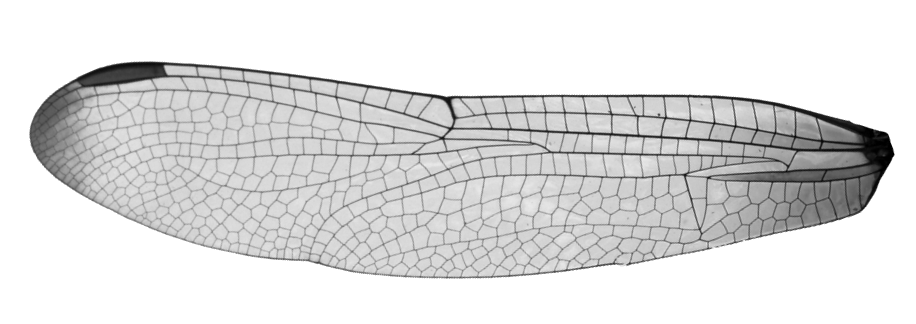

Supplement: Supplementary file 1 — Additional file 1: Trithemis wing images archive. [file 12862_2022_1978_MOESM1_ESM.zip › Additional Files 1/Trithemis Wing Images Archive/Trithemis Wing Images/Forewings/Images (w: Numbers)/276.tif]

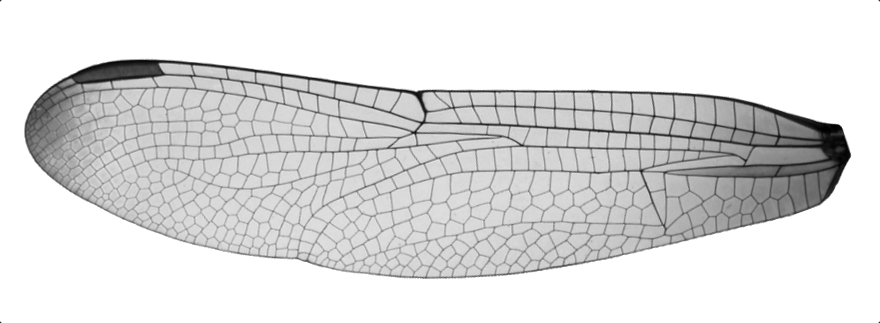

Supplement: Supplementary file 1 — Additional file 1: Trithemis wing images archive. [file 12862_2022_1978_MOESM1_ESM.zip › Additional Files 1/Trithemis Wing Images Archive/Trithemis Wing Images/Forewings/Images (w: Numbers)/277.tif]

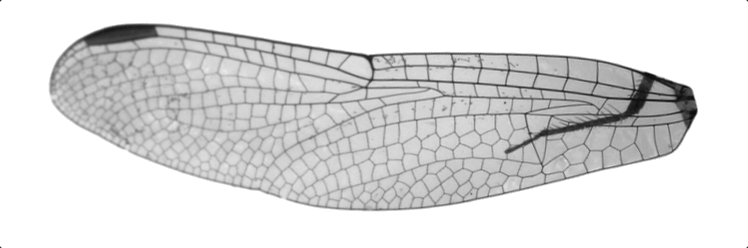

Supplement: Supplementary file 1 — Additional file 1: Trithemis wing images archive. [file 12862_2022_1978_MOESM1_ESM.zip › Additional Files 1/Trithemis Wing Images Archive/Trithemis Wing Images/Forewings/Images (w: Numbers)/263.tif]

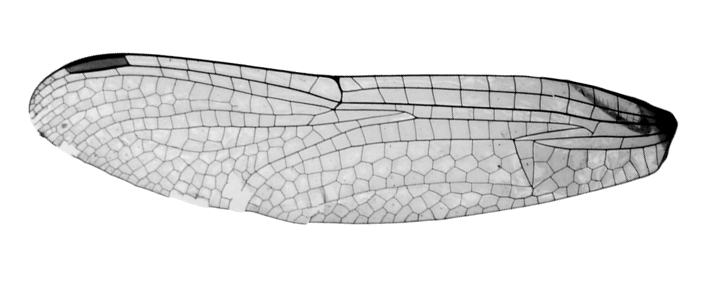

Supplement: Supplementary file 1 — Additional file 1: Trithemis wing images archive. [file 12862_2022_1978_MOESM1_ESM.zip › Additional Files 1/Trithemis Wing Images Archive/Trithemis Wing Images/Forewings/Images (w: Numbers)/075.tif]

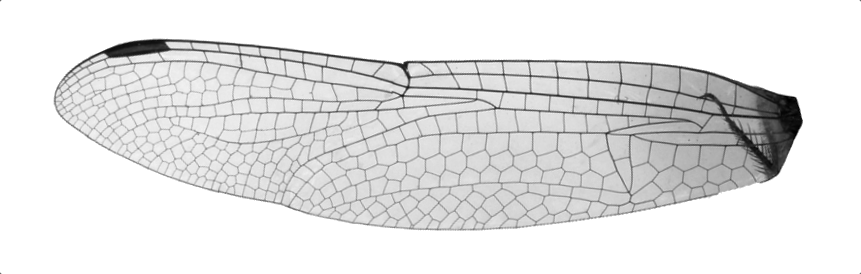

Supplement: Supplementary file 1 — Additional file 1: Trithemis wing images archive. [file 12862_2022_1978_MOESM1_ESM.zip › Additional Files 1/Trithemis Wing Images Archive/Trithemis Wing Images/Forewings/Images (w: Numbers)/061.tif]

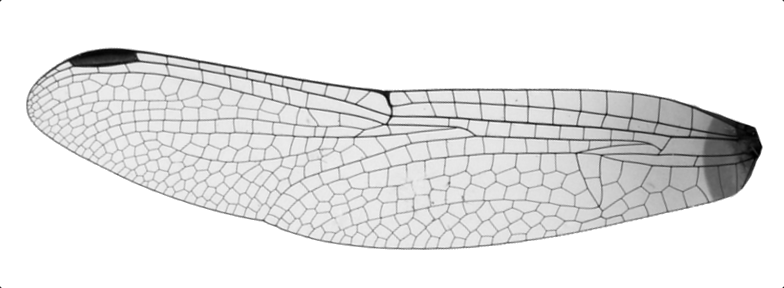

Supplement: Supplementary file 1 — Additional file 1: Trithemis wing images archive. [file 12862_2022_1978_MOESM1_ESM.zip › Additional Files 1/Trithemis Wing Images Archive/Trithemis Wing Images/Forewings/Images (w: Numbers)/129.tif]

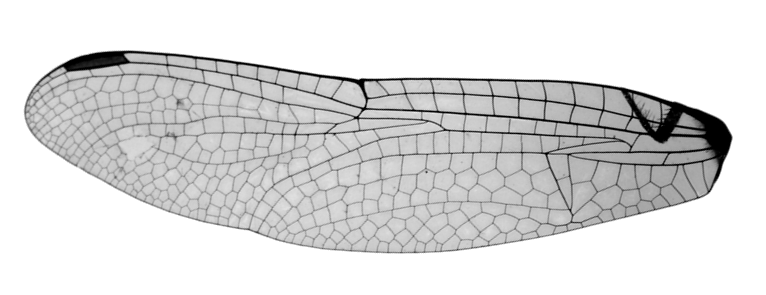

Supplement: Supplementary file 1 — Additional file 1: Trithemis wing images archive. [file 12862_2022_1978_MOESM1_ESM.zip › Additional Files 1/Trithemis Wing Images Archive/Trithemis Wing Images/Forewings/Images (w: Numbers)/077.tif]

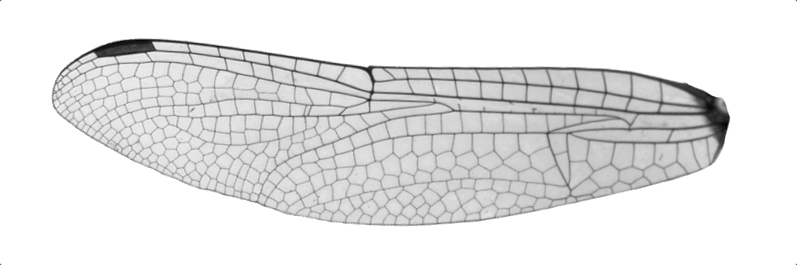

Supplement: Supplementary file 1 — Additional file 1: Trithemis wing images archive. [file 12862_2022_1978_MOESM1_ESM.zip › Additional Files 1/Trithemis Wing Images Archive/Trithemis Wing Images/Forewings/Images (w: Numbers)/063.tif]

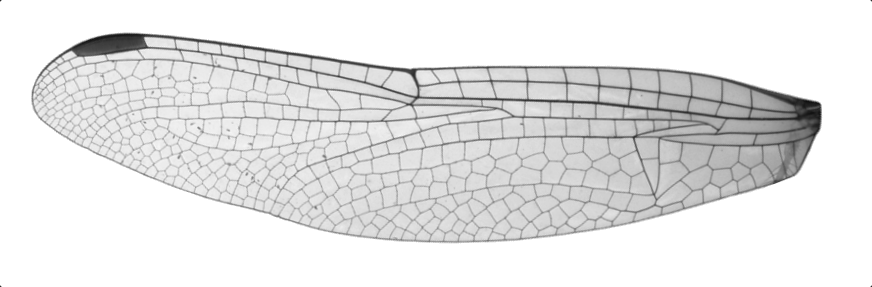

Supplement: Supplementary file 1 — Additional file 1: Trithemis wing images archive. [file 12862_2022_1978_MOESM1_ESM.zip › Additional Files 1/Trithemis Wing Images Archive/Trithemis Wing Images/Forewings/Images (w: Numbers)/249.tif]

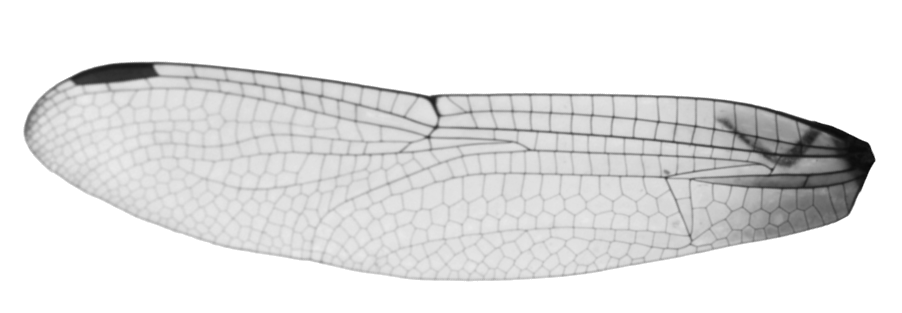

Supplement: Supplementary file 1 — Additional file 1: Trithemis wing images archive. [file 12862_2022_1978_MOESM1_ESM.zip › Additional Files 1/Trithemis Wing Images Archive/Trithemis Wing Images/Forewings/Images (w: Numbers)/275.tif]

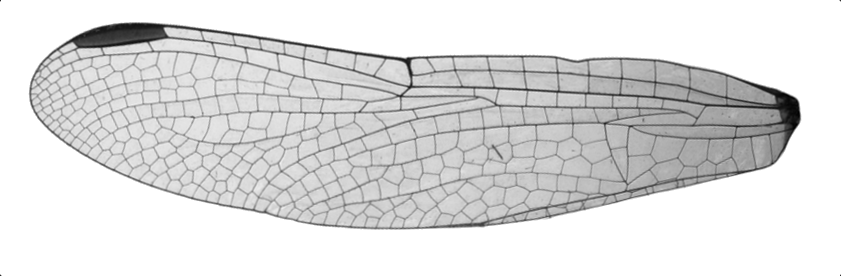

Supplement: Supplementary file 1 — Additional file 1: Trithemis wing images archive. [file 12862_2022_1978_MOESM1_ESM.zip › Additional Files 1/Trithemis Wing Images Archive/Trithemis Wing Images/Forewings/Images (w: Numbers)/261.tif]

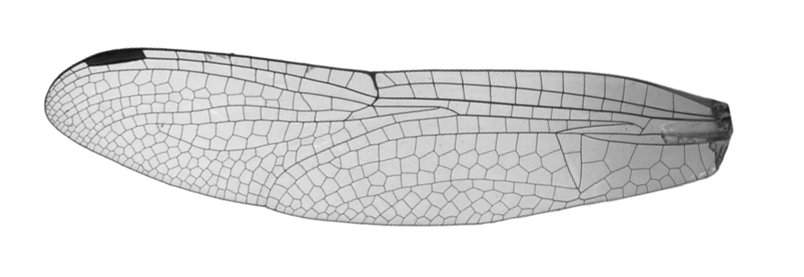

Supplement: Supplementary file 1 — Additional file 1: Trithemis wing images archive. [file 12862_2022_1978_MOESM1_ESM.zip › Additional Files 1/Trithemis Wing Images Archive/Trithemis Wing Images/Forewings/Images (w: Numbers)/260.tif]

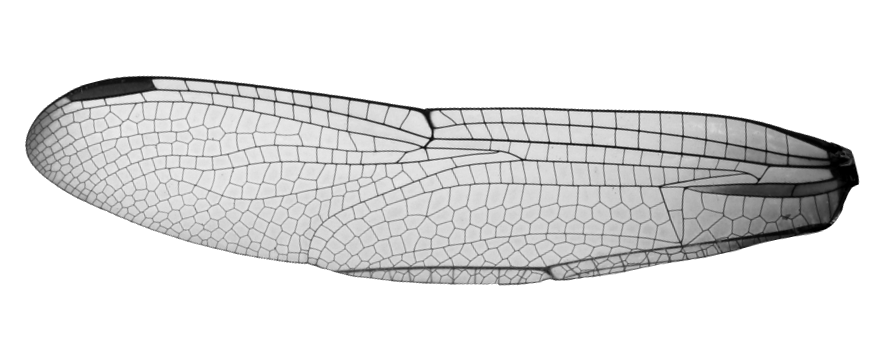

Supplement: Supplementary file 1 — Additional file 1: Trithemis wing images archive. [file 12862_2022_1978_MOESM1_ESM.zip › Additional Files 1/Trithemis Wing Images Archive/Trithemis Wing Images/Forewings/Images (w: Numbers)/274.tif]

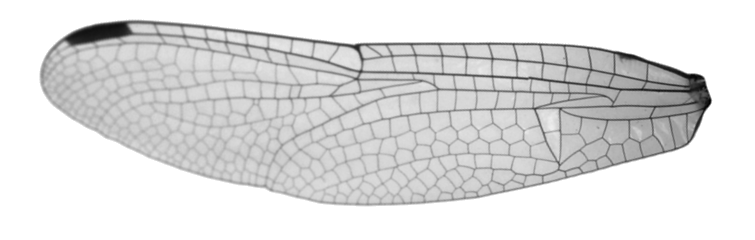

Supplement: Supplementary file 1 — Additional file 1: Trithemis wing images archive. [file 12862_2022_1978_MOESM1_ESM.zip › Additional Files 1/Trithemis Wing Images Archive/Trithemis Wing Images/Forewings/Images (w: Numbers)/248.tif]

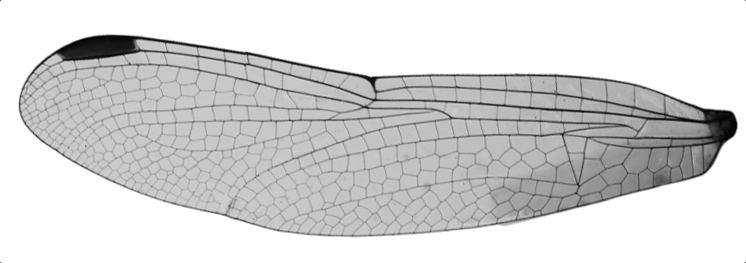

Supplement: Supplementary file 1 — Additional file 1: Trithemis wing images archive. [file 12862_2022_1978_MOESM1_ESM.zip › Additional Files 1/Trithemis Wing Images Archive/Trithemis Wing Images/Forewings/Images (w: Numbers)/089.tif]

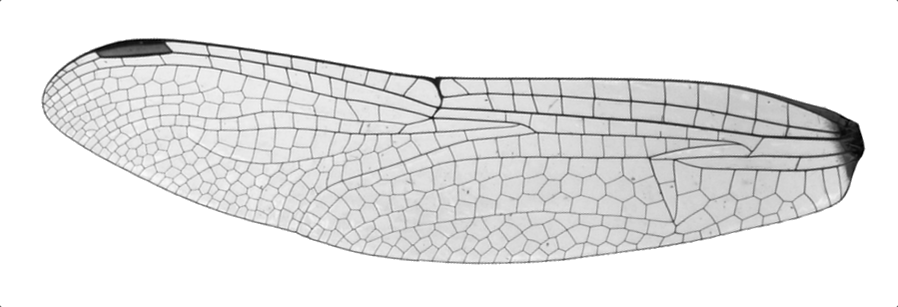

Supplement: Supplementary file 1 — Additional file 1: Trithemis wing images archive. [file 12862_2022_1978_MOESM1_ESM.zip › Additional Files 1/Trithemis Wing Images Archive/Trithemis Wing Images/Forewings/Images (w: Numbers)/062.tif]

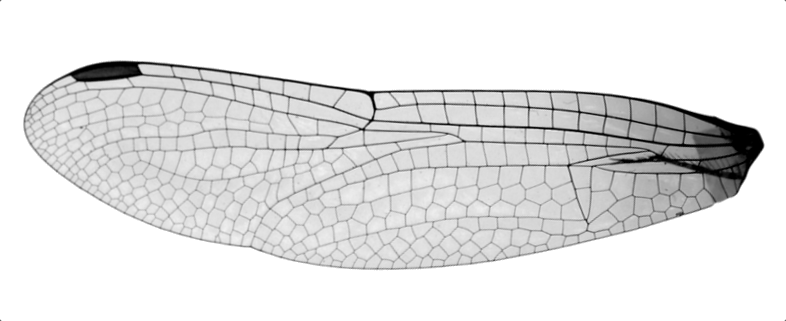

Supplement: Supplementary file 1 — Additional file 1: Trithemis wing images archive. [file 12862_2022_1978_MOESM1_ESM.zip › Additional Files 1/Trithemis Wing Images Archive/Trithemis Wing Images/Forewings/Images (w: Numbers)/076.tif]

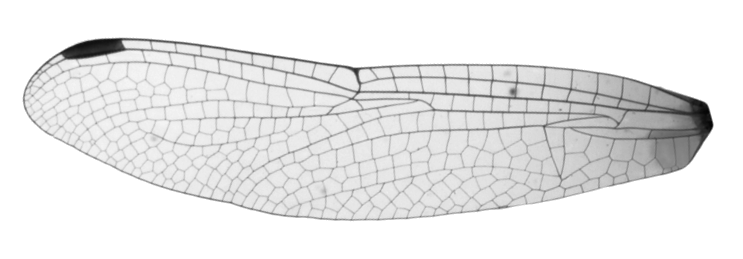

Supplement: Supplementary file 1 — Additional file 1: Trithemis wing images archive. [file 12862_2022_1978_MOESM1_ESM.zip › Additional Files 1/Trithemis Wing Images Archive/Trithemis Wing Images/Forewings/Images (w: Numbers)/165.tif]

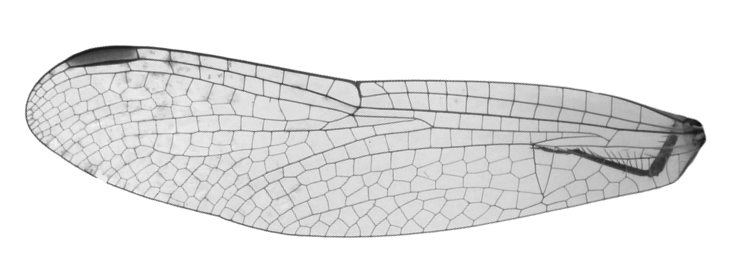

Supplement: Supplementary file 1 — Additional file 1: Trithemis wing images archive. [file 12862_2022_1978_MOESM1_ESM.zip › Additional Files 1/Trithemis Wing Images Archive/Trithemis Wing Images/Forewings/Images (w: Numbers)/171.tif]

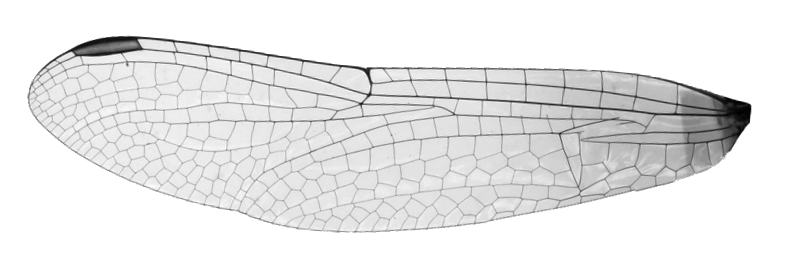

Supplement: Supplementary file 1 — Additional file 1: Trithemis wing images archive. [file 12862_2022_1978_MOESM1_ESM.zip › Additional Files 1/Trithemis Wing Images Archive/Trithemis Wing Images/Forewings/Images (w: Numbers)/159.tif]

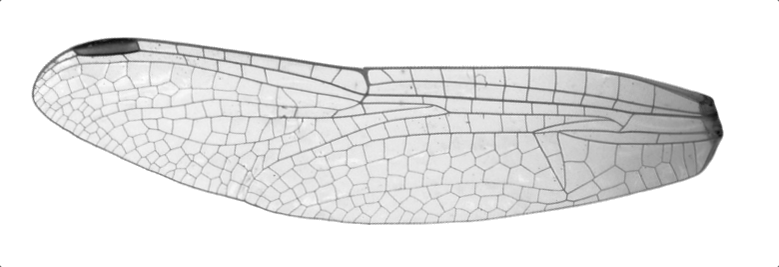

Supplement: Supplementary file 1 — Additional file 1: Trithemis wing images archive. [file 12862_2022_1978_MOESM1_ESM.zip › Additional Files 1/Trithemis Wing Images Archive/Trithemis Wing Images/Forewings/Images (w: Numbers)/011.tif]

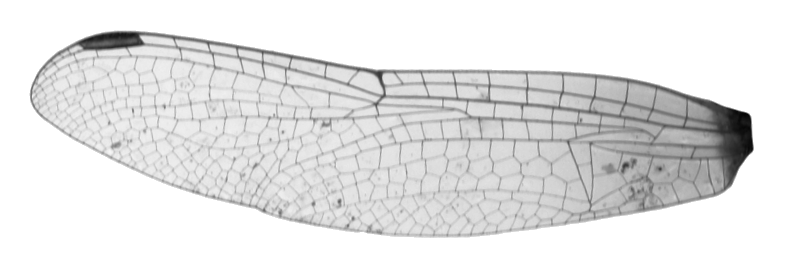

Supplement: Supplementary file 1 — Additional file 1: Trithemis wing images archive. [file 12862_2022_1978_MOESM1_ESM.zip › Additional Files 1/Trithemis Wing Images Archive/Trithemis Wing Images/Forewings/Images (w: Numbers)/005.tif]

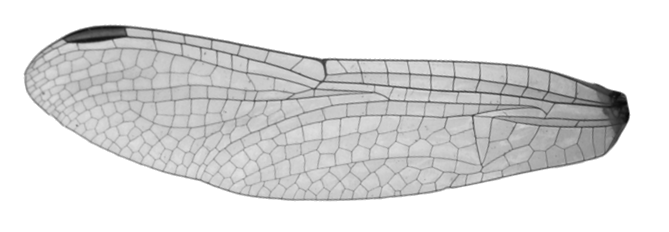

Supplement: Supplementary file 1 — Additional file 1: Trithemis wing images archive. [file 12862_2022_1978_MOESM1_ESM.zip › Additional Files 1/Trithemis Wing Images Archive/Trithemis Wing Images/Forewings/Images (w: Numbers)/039.tif]

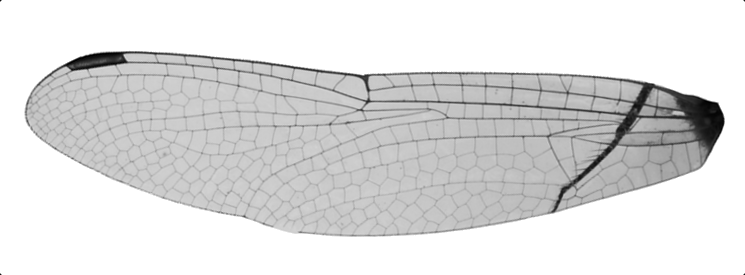

Supplement: Supplementary file 1 — Additional file 1: Trithemis wing images archive. [file 12862_2022_1978_MOESM1_ESM.zip › Additional Files 1/Trithemis Wing Images Archive/Trithemis Wing Images/Forewings/Images (w: Numbers)/207.tif]

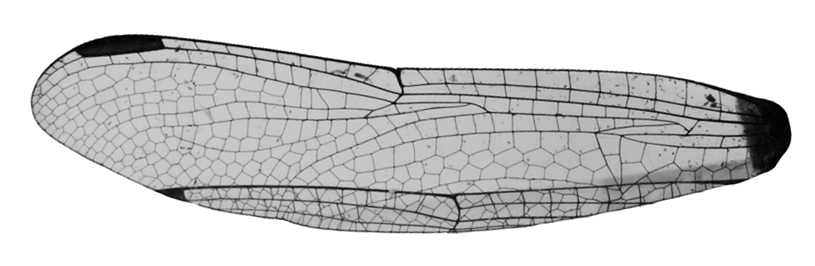

Supplement: Supplementary file 1 — Additional file 1: Trithemis wing images archive. [file 12862_2022_1978_MOESM1_ESM.zip › Additional Files 1/Trithemis Wing Images Archive/Trithemis Wing Images/Forewings/Images (w: Numbers)/212.tif]

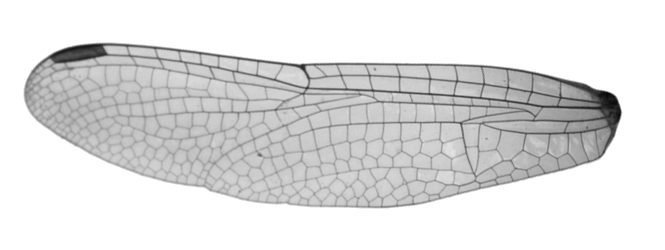

Supplement: Supplementary file 1 — Additional file 1: Trithemis wing images archive. [file 12862_2022_1978_MOESM1_ESM.zip › Additional Files 1/Trithemis Wing Images Archive/Trithemis Wing Images/Forewings/Images (w: Numbers)/038.tif]

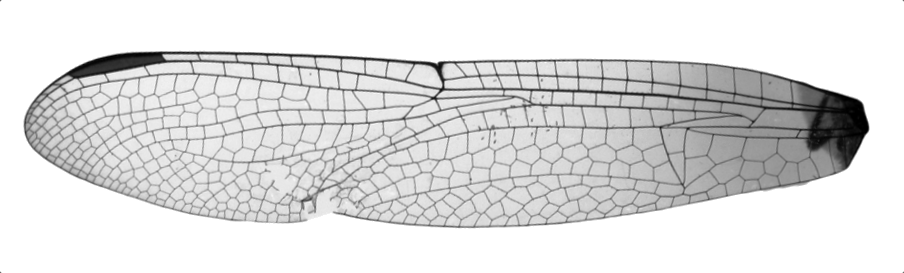

Supplement: Supplementary file 1 — Additional file 1: Trithemis wing images archive. [file 12862_2022_1978_MOESM1_ESM.zip › Additional Files 1/Trithemis Wing Images Archive/Trithemis Wing Images/Forewings/Images (w: Numbers)/004.tif]

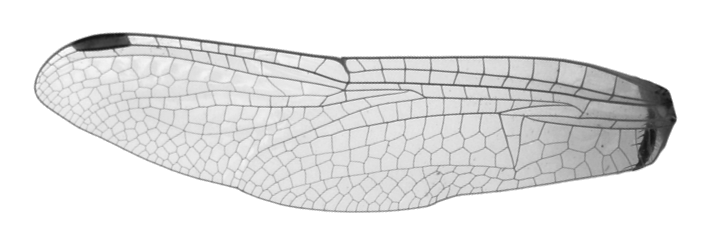

Supplement: Supplementary file 1 — Additional file 1: Trithemis wing images archive. [file 12862_2022_1978_MOESM1_ESM.zip › Additional Files 1/Trithemis Wing Images Archive/Trithemis Wing Images/Forewings/Images (w: Numbers)/010.tif]

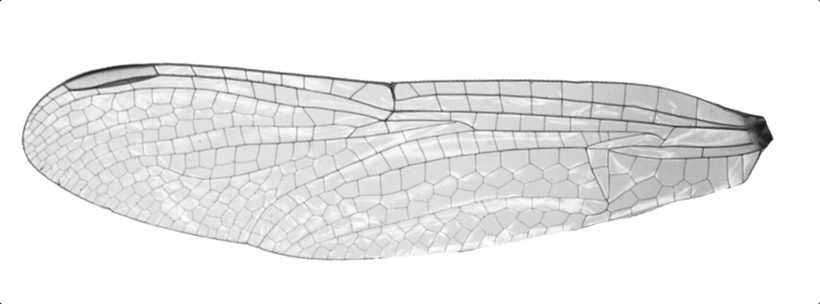

Supplement: Supplementary file 1 — Additional file 1: Trithemis wing images archive. [file 12862_2022_1978_MOESM1_ESM.zip › Additional Files 1/Trithemis Wing Images Archive/Trithemis Wing Images/Forewings/Images (w: Numbers)/158.tif]

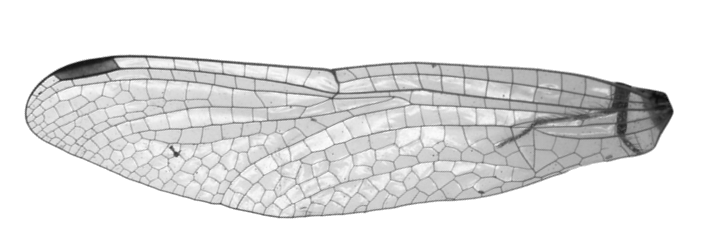

Supplement: Supplementary file 1 — Additional file 1: Trithemis wing images archive. [file 12862_2022_1978_MOESM1_ESM.zip › Additional Files 1/Trithemis Wing Images Archive/Trithemis Wing Images/Forewings/Images (w: Numbers)/170.tif]

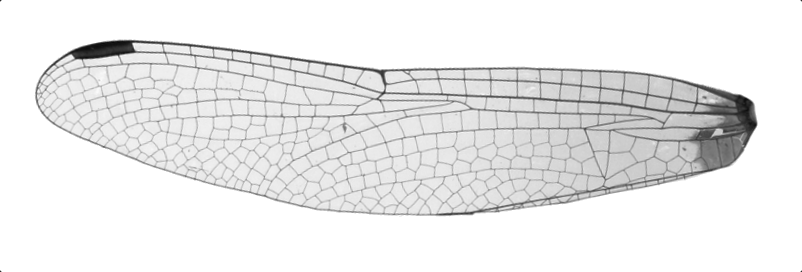

Supplement: Supplementary file 1 — Additional file 1: Trithemis wing images archive. [file 12862_2022_1978_MOESM1_ESM.zip › Additional Files 1/Trithemis Wing Images Archive/Trithemis Wing Images/Forewings/Images (w: Numbers)/164.tif]

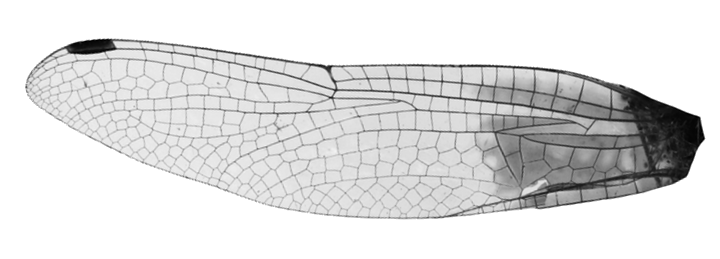

Supplement: Supplementary file 1 — Additional file 1: Trithemis wing images archive. [file 12862_2022_1978_MOESM1_ESM.zip › Additional Files 1/Trithemis Wing Images Archive/Trithemis Wing Images/Forewings/Images (w: Numbers)/172.tif]

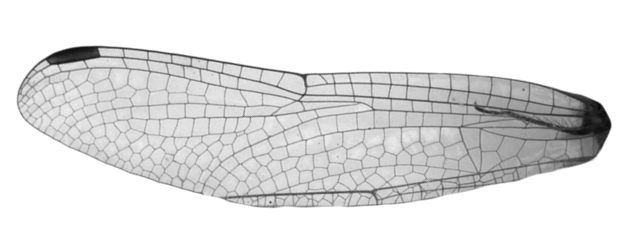

Supplement: Supplementary file 1 — Additional file 1: Trithemis wing images archive. [file 12862_2022_1978_MOESM1_ESM.zip › Additional Files 1/Trithemis Wing Images Archive/Trithemis Wing Images/Forewings/Images (w: Numbers)/166.tif]

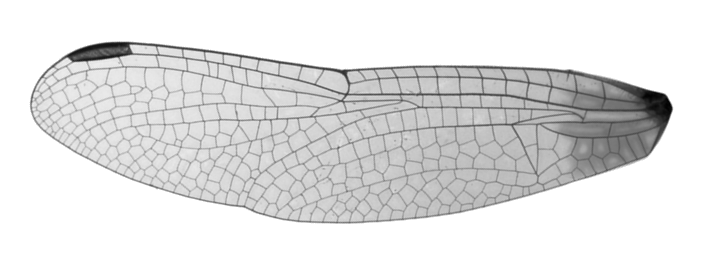

Supplement: Supplementary file 1 — Additional file 1: Trithemis wing images archive. [file 12862_2022_1978_MOESM1_ESM.zip › Additional Files 1/Trithemis Wing Images Archive/Trithemis Wing Images/Forewings/Images (w: Numbers)/199.tif]

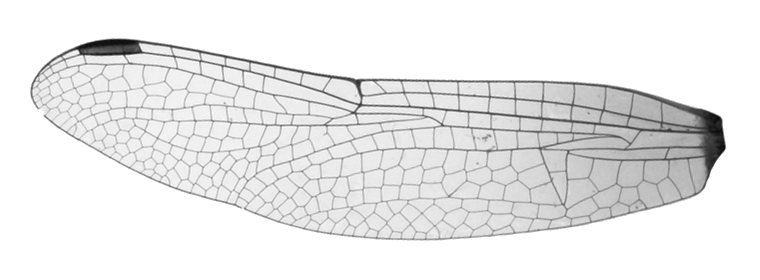

Supplement: Supplementary file 1 — Additional file 1: Trithemis wing images archive. [file 12862_2022_1978_MOESM1_ESM.zip › Additional Files 1/Trithemis Wing Images Archive/Trithemis Wing Images/Forewings/Images (w: Numbers)/006.tif]

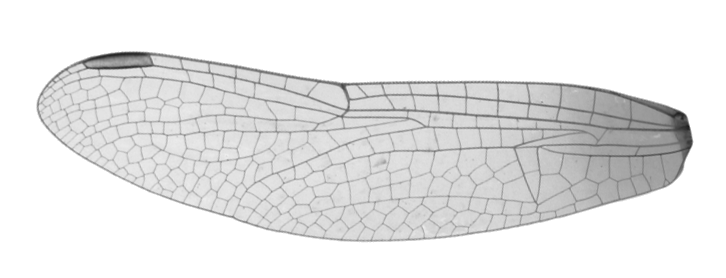

Supplement: Supplementary file 1 — Additional file 1: Trithemis wing images archive. [file 12862_2022_1978_MOESM1_ESM.zip › Additional Files 1/Trithemis Wing Images Archive/Trithemis Wing Images/Forewings/Images (w: Numbers)/012.tif]

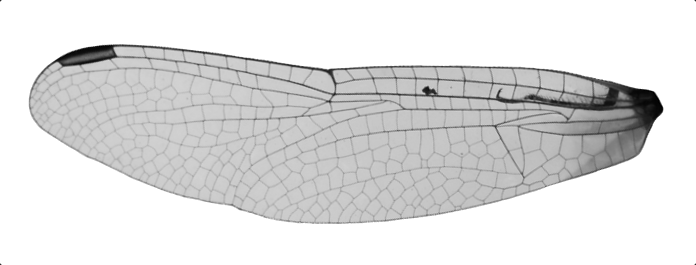

Supplement: Supplementary file 1 — Additional file 1: Trithemis wing images archive. [file 12862_2022_1978_MOESM1_ESM.zip › Additional Files 1/Trithemis Wing Images Archive/Trithemis Wing Images/Forewings/Images (w: Numbers)/204.tif]

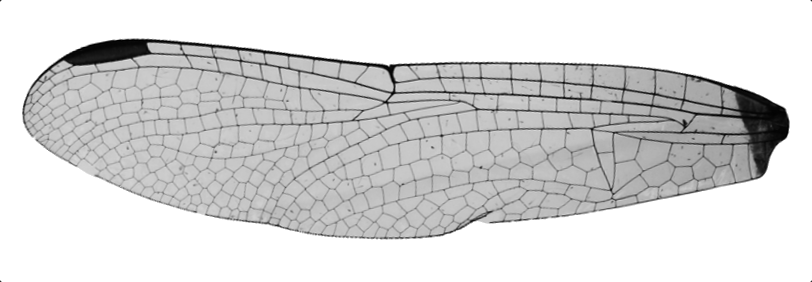

Supplement: Supplementary file 1 — Additional file 1: Trithemis wing images archive. [file 12862_2022_1978_MOESM1_ESM.zip › Additional Files 1/Trithemis Wing Images Archive/Trithemis Wing Images/Forewings/Images (w: Numbers)/210.tif]

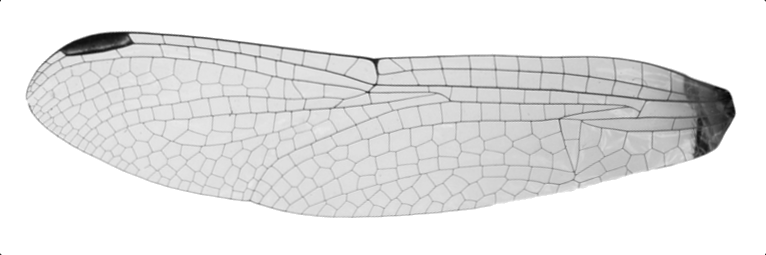

Supplement: Supplementary file 1 — Additional file 1: Trithemis wing images archive. [file 12862_2022_1978_MOESM1_ESM.zip › Additional Files 1/Trithemis Wing Images Archive/Trithemis Wing Images/Forewings/Images (w: Numbers)/238.tif]

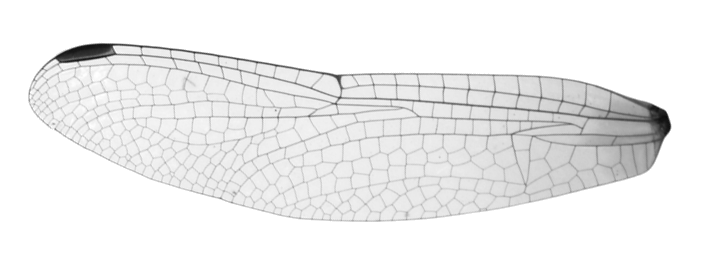

Supplement: Supplementary file 1 — Additional file 1: Trithemis wing images archive. [file 12862_2022_1978_MOESM1_ESM.zip › Additional Files 1/Trithemis Wing Images Archive/Trithemis Wing Images/Forewings/Images (w: Numbers)/239.tif]

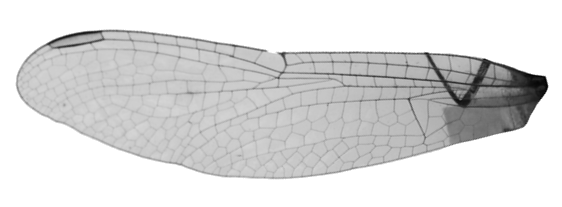

Supplement: Supplementary file 1 — Additional file 1: Trithemis wing images archive. [file 12862_2022_1978_MOESM1_ESM.zip › Additional Files 1/Trithemis Wing Images Archive/Trithemis Wing Images/Forewings/Images (w: Numbers)/205.tif]

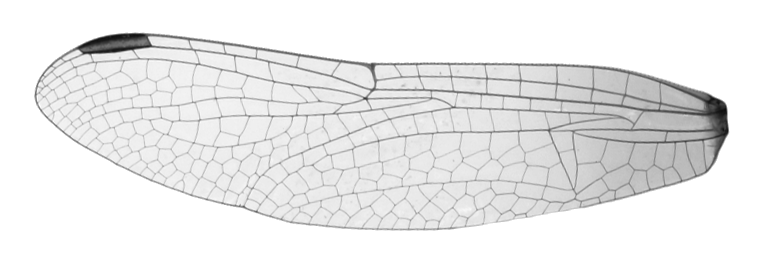

Supplement: Supplementary file 1 — Additional file 1: Trithemis wing images archive. [file 12862_2022_1978_MOESM1_ESM.zip › Additional Files 1/Trithemis Wing Images Archive/Trithemis Wing Images/Forewings/Images (w: Numbers)/013.tif]

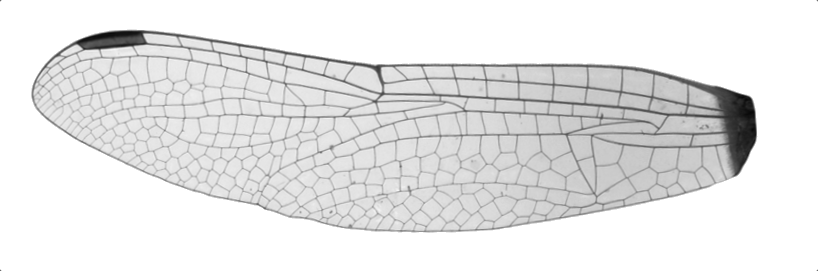

Supplement: Supplementary file 1 — Additional file 1: Trithemis wing images archive. [file 12862_2022_1978_MOESM1_ESM.zip › Additional Files 1/Trithemis Wing Images Archive/Trithemis Wing Images/Forewings/Images (w: Numbers)/007.tif]

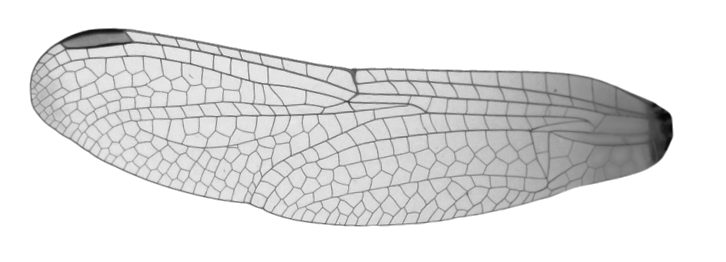

Supplement: Supplementary file 1 — Additional file 1: Trithemis wing images archive. [file 12862_2022_1978_MOESM1_ESM.zip › Additional Files 1/Trithemis Wing Images Archive/Trithemis Wing Images/Forewings/Images (w: Numbers)/198.tif]

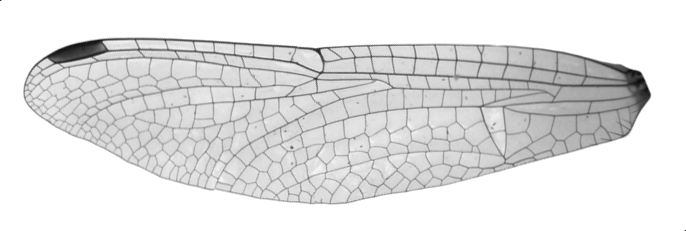

Supplement: Supplementary file 1 — Additional file 1: Trithemis wing images archive. [file 12862_2022_1978_MOESM1_ESM.zip › Additional Files 1/Trithemis Wing Images Archive/Trithemis Wing Images/Forewings/Images (w: Numbers)/167.tif]

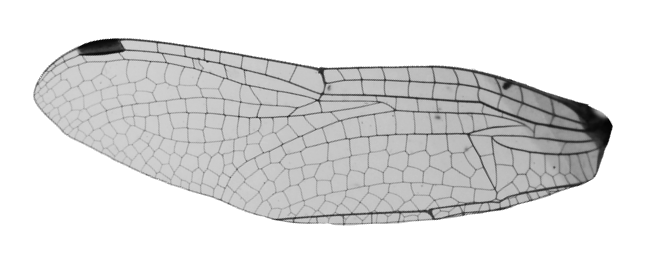

Supplement: Supplementary file 1 — Additional file 1: Trithemis wing images archive. [file 12862_2022_1978_MOESM1_ESM.zip › Additional Files 1/Trithemis Wing Images Archive/Trithemis Wing Images/Forewings/Images (w: Numbers)/173.tif]

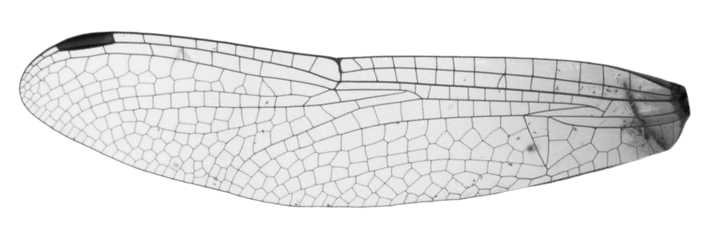

Supplement: Supplementary file 1 — Additional file 1: Trithemis wing images archive. [file 12862_2022_1978_MOESM1_ESM.zip › Additional Files 1/Trithemis Wing Images Archive/Trithemis Wing Images/Forewings/Images (w: Numbers)/163.tif]

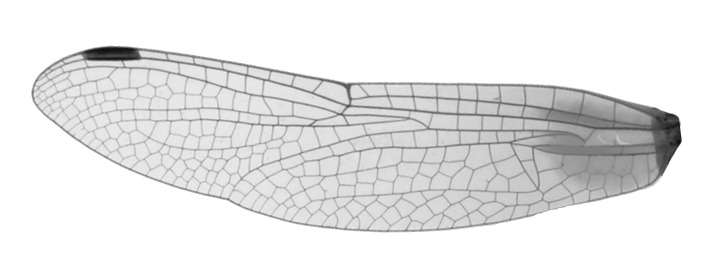

Supplement: Supplementary file 1 — Additional file 1: Trithemis wing images archive. [file 12862_2022_1978_MOESM1_ESM.zip › Additional Files 1/Trithemis Wing Images Archive/Trithemis Wing Images/Forewings/Images (w: Numbers)/188.tif]

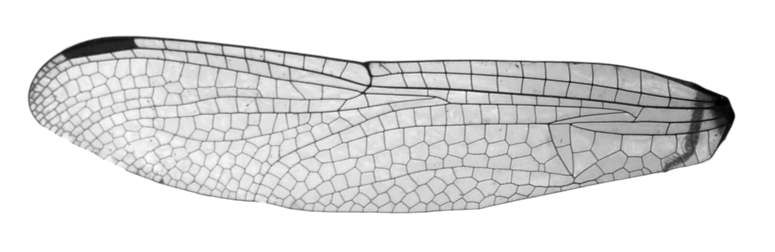

Supplement: Supplementary file 1 — Additional file 1: Trithemis wing images archive. [file 12862_2022_1978_MOESM1_ESM.zip › Additional Files 1/Trithemis Wing Images Archive/Trithemis Wing Images/Forewings/Images (w: Numbers)/003.tif]

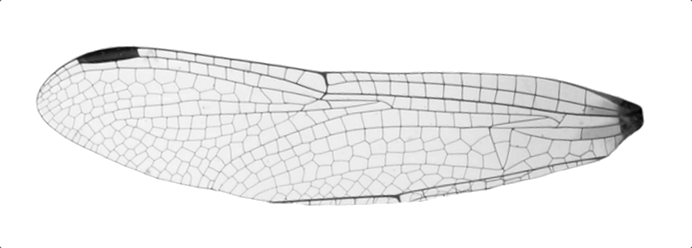

Supplement: Supplementary file 1 — Additional file 1: Trithemis wing images archive. [file 12862_2022_1978_MOESM1_ESM.zip › Additional Files 1/Trithemis Wing Images Archive/Trithemis Wing Images/Forewings/Images (w: Numbers)/017.tif]

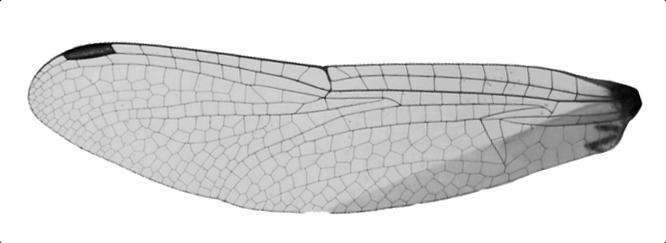

Supplement: Supplementary file 1 — Additional file 1: Trithemis wing images archive. [file 12862_2022_1978_MOESM1_ESM.zip › Additional Files 1/Trithemis Wing Images Archive/Trithemis Wing Images/Forewings/Images (w: Numbers)/201.tif]

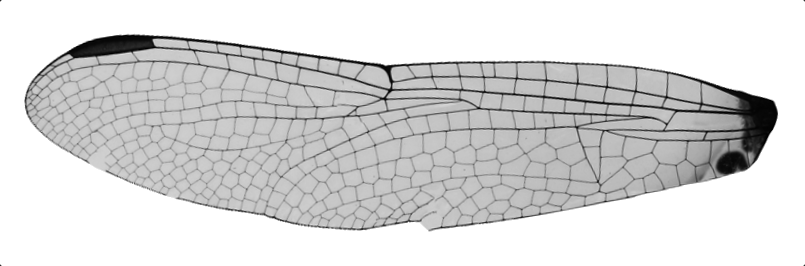

Supplement: Supplementary file 1 — Additional file 1: Trithemis wing images archive. [file 12862_2022_1978_MOESM1_ESM.zip › Additional Files 1/Trithemis Wing Images Archive/Trithemis Wing Images/Forewings/Images (w: Numbers)/215.tif]

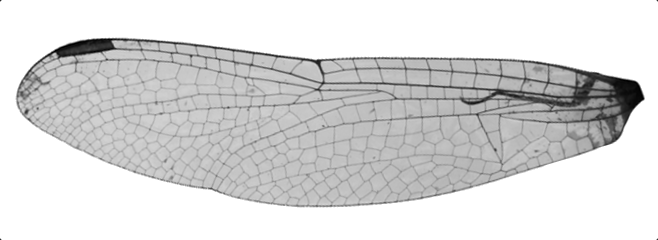

Supplement: Supplementary file 1 — Additional file 1: Trithemis wing images archive. [file 12862_2022_1978_MOESM1_ESM.zip › Additional Files 1/Trithemis Wing Images Archive/Trithemis Wing Images/Forewings/Images (w: Numbers)/200.tif]

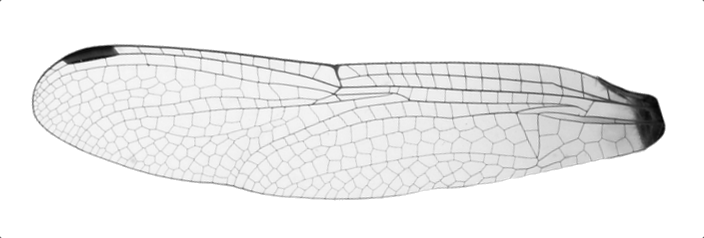

Supplement: Supplementary file 1 — Additional file 1: Trithemis wing images archive. [file 12862_2022_1978_MOESM1_ESM.zip › Additional Files 1/Trithemis Wing Images Archive/Trithemis Wing Images/Forewings/Images (w: Numbers)/016.tif]

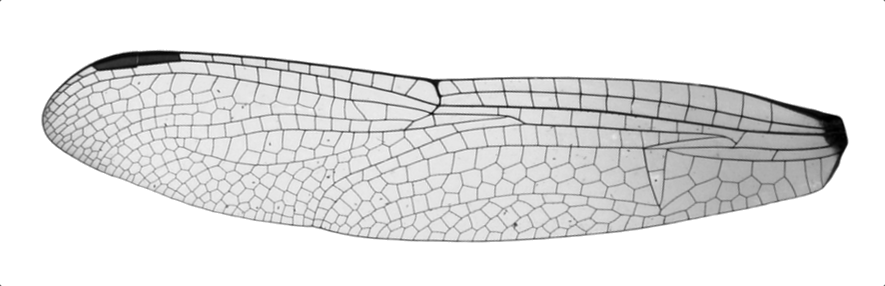

Supplement: Supplementary file 1 — Additional file 1: Trithemis wing images archive. [file 12862_2022_1978_MOESM1_ESM.zip › Additional Files 1/Trithemis Wing Images Archive/Trithemis Wing Images/Forewings/Images (w: Numbers)/002.tif]

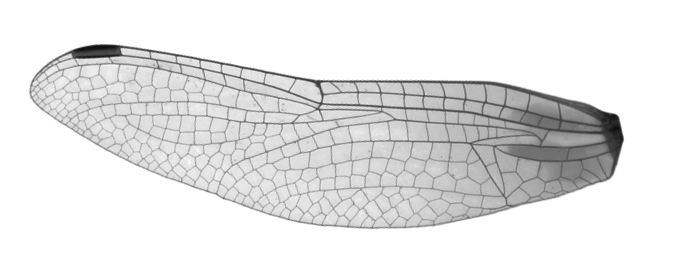

Supplement: Supplementary file 1 — Additional file 1: Trithemis wing images archive. [file 12862_2022_1978_MOESM1_ESM.zip › Additional Files 1/Trithemis Wing Images Archive/Trithemis Wing Images/Forewings/Images (w: Numbers)/189.tif]

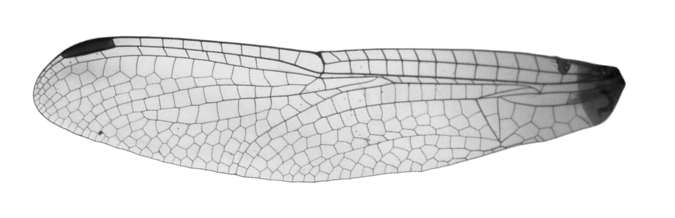

Supplement: Supplementary file 1 — Additional file 1: Trithemis wing images archive. [file 12862_2022_1978_MOESM1_ESM.zip › Additional Files 1/Trithemis Wing Images Archive/Trithemis Wing Images/Forewings/Images (w: Numbers)/162.tif]

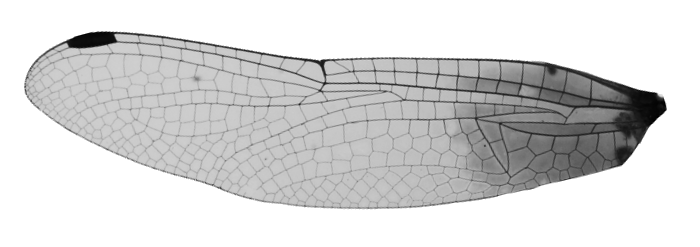

Supplement: Supplementary file 1 — Additional file 1: Trithemis wing images archive. [file 12862_2022_1978_MOESM1_ESM.zip › Additional Files 1/Trithemis Wing Images Archive/Trithemis Wing Images/Forewings/Images (w: Numbers)/176.tif]

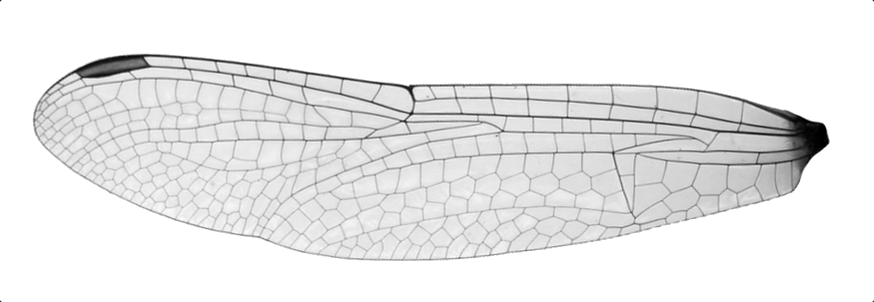

Supplement: Supplementary file 1 — Additional file 1: Trithemis wing images archive. [file 12862_2022_1978_MOESM1_ESM.zip › Additional Files 1/Trithemis Wing Images Archive/Trithemis Wing Images/Forewings/Images (w: Numbers)/160.tif]

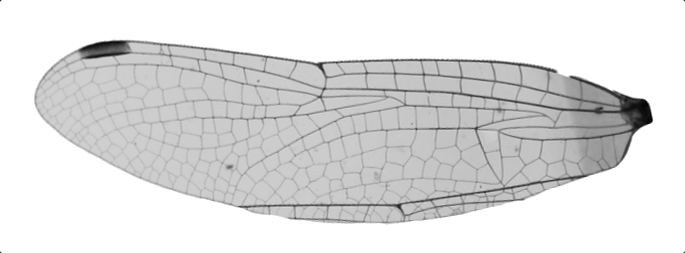

Supplement: Supplementary file 1 — Additional file 1: Trithemis wing images archive. [file 12862_2022_1978_MOESM1_ESM.zip › Additional Files 1/Trithemis Wing Images Archive/Trithemis Wing Images/Forewings/Images (w: Numbers)/174.tif]

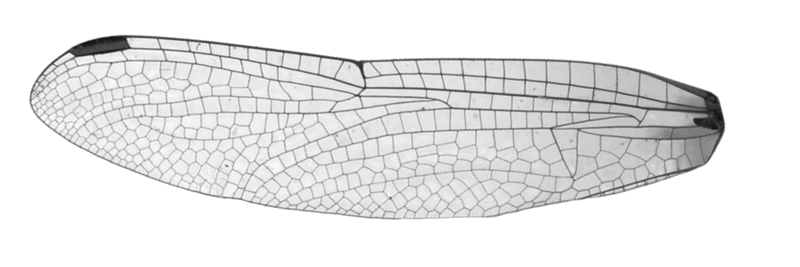

Supplement: Supplementary file 1 — Additional file 1: Trithemis wing images archive. [file 12862_2022_1978_MOESM1_ESM.zip › Additional Files 1/Trithemis Wing Images Archive/Trithemis Wing Images/Forewings/Images (w: Numbers)/028.tif]

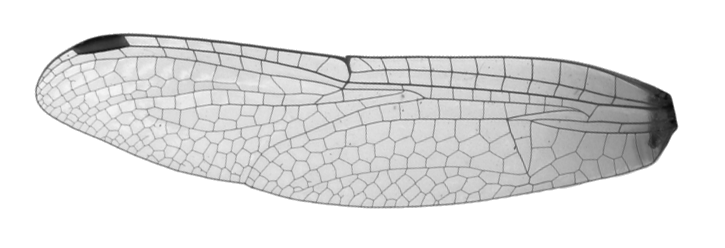

Supplement: Supplementary file 1 — Additional file 1: Trithemis wing images archive. [file 12862_2022_1978_MOESM1_ESM.zip › Additional Files 1/Trithemis Wing Images Archive/Trithemis Wing Images/Forewings/Images (w: Numbers)/014.tif]

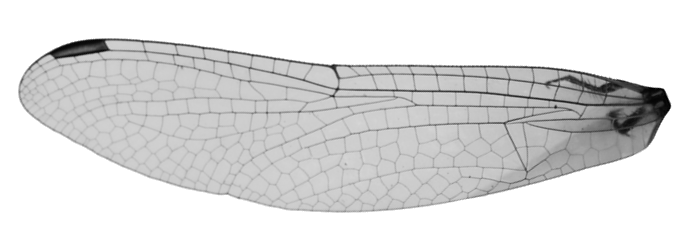

Supplement: Supplementary file 1 — Additional file 1: Trithemis wing images archive. [file 12862_2022_1978_MOESM1_ESM.zip › Additional Files 1/Trithemis Wing Images Archive/Trithemis Wing Images/Forewings/Images (w: Numbers)/202.tif]

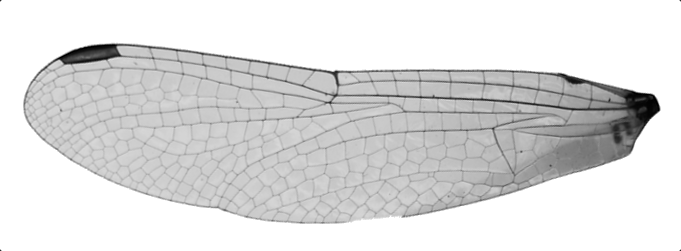

Supplement: Supplementary file 1 — Additional file 1: Trithemis wing images archive. [file 12862_2022_1978_MOESM1_ESM.zip › Additional Files 1/Trithemis Wing Images Archive/Trithemis Wing Images/Forewings/Images (w: Numbers)/203.tif]

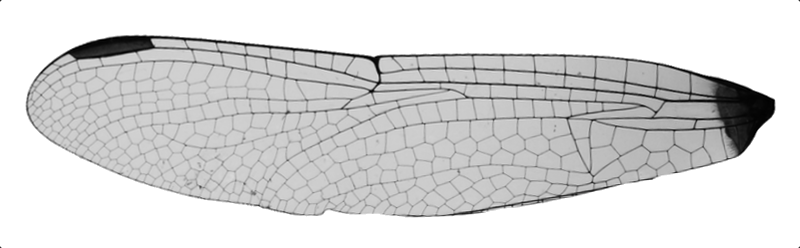

Supplement: Supplementary file 1 — Additional file 1: Trithemis wing images archive. [file 12862_2022_1978_MOESM1_ESM.zip › Additional Files 1/Trithemis Wing Images Archive/Trithemis Wing Images/Forewings/Images (w: Numbers)/217.tif]

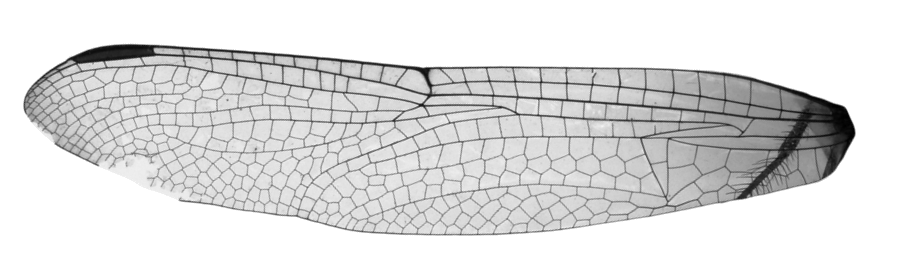

Supplement: Supplementary file 1 — Additional file 1: Trithemis wing images archive. [file 12862_2022_1978_MOESM1_ESM.zip › Additional Files 1/Trithemis Wing Images Archive/Trithemis Wing Images/Forewings/Images (w: Numbers)/001.tif]

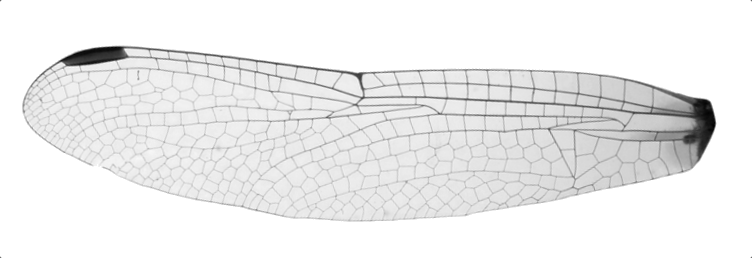

Supplement: Supplementary file 1 — Additional file 1: Trithemis wing images archive. [file 12862_2022_1978_MOESM1_ESM.zip › Additional Files 1/Trithemis Wing Images Archive/Trithemis Wing Images/Forewings/Images (w: Numbers)/015.tif]

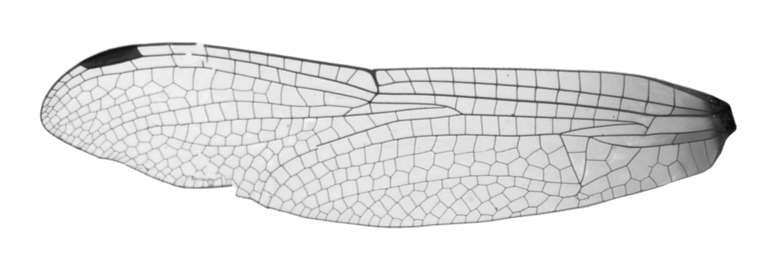

Supplement: Supplementary file 1 — Additional file 1: Trithemis wing images archive. [file 12862_2022_1978_MOESM1_ESM.zip › Additional Files 1/Trithemis Wing Images Archive/Trithemis Wing Images/Forewings/Images (w: Numbers)/029.tif]

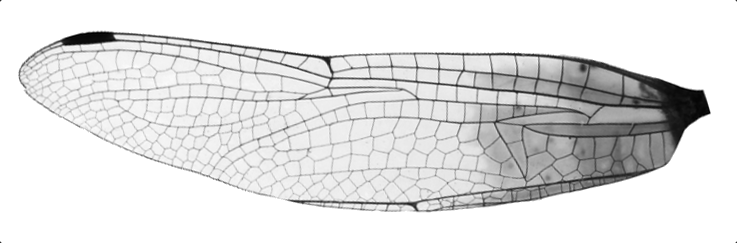

Supplement: Supplementary file 1 — Additional file 1: Trithemis wing images archive. [file 12862_2022_1978_MOESM1_ESM.zip › Additional Files 1/Trithemis Wing Images Archive/Trithemis Wing Images/Forewings/Images (w: Numbers)/175.tif]

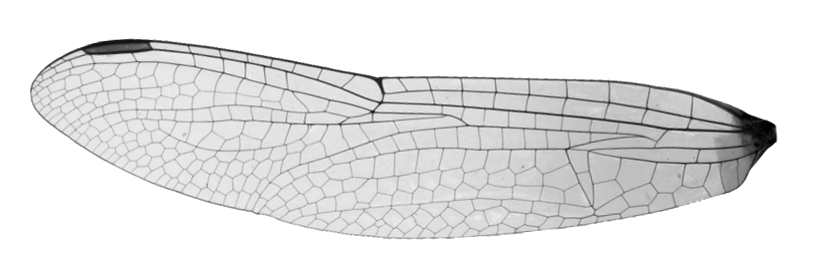

Supplement: Supplementary file 1 — Additional file 1: Trithemis wing images archive. [file 12862_2022_1978_MOESM1_ESM.zip › Additional Files 1/Trithemis Wing Images Archive/Trithemis Wing Images/Forewings/Images (w: Numbers)/161.tif]

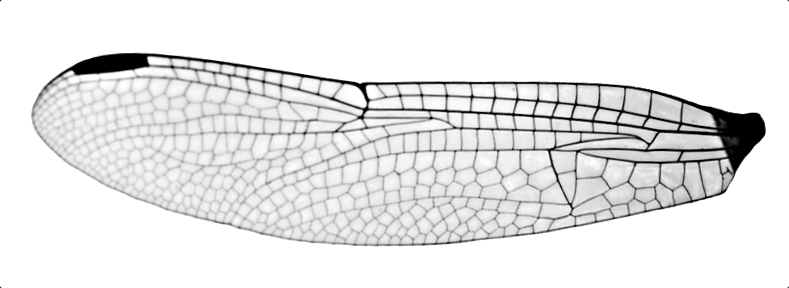

Supplement: Supplementary file 1 — Additional file 1: Trithemis wing images archive. [file 12862_2022_1978_MOESM1_ESM.zip › Additional Files 1/Trithemis Wing Images Archive/Trithemis Wing Images/Forewings/Images (w: Numbers)/149.tif]

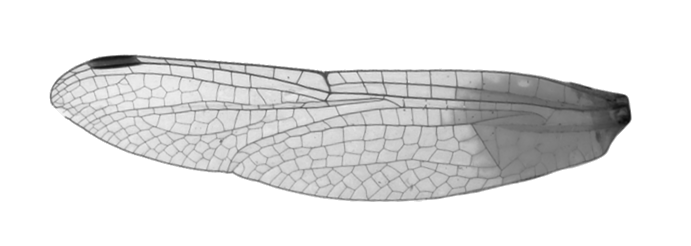

Supplement: Supplementary file 1 — Additional file 1: Trithemis wing images archive. [file 12862_2022_1978_MOESM1_ESM.zip › Additional Files 1/Trithemis Wing Images Archive/Trithemis Wing Images/Forewings/Images (w: Numbers)/187.tif]
