# Supplementary material for: What you sample is what you get: ecomorphological variation in Trithemis (Odonata, Libellulidae) dragonfly wings reconsidered
Source: BMC Ecol Evol. 2022 Apr 11;22:43. doi: 10.1186/s12862-022-01978-y (PMC8996507; doi:10.1186/s12862-022-01978-y)
Supplement: Supplementary file 2 — Additional file 2: Datasets and results archive. [file 12862_2022_1978_MOESM2_ESM.zip › Additional Files 2/Datasets & Results Archive/Geometric Morphometrics (Landmarks) Analyses/Landmark-Semilandmark Definitions.pdf]

Trithemis wing landmark-semilandmark definitions.

Landmark	Definition	Landmark	Definition
1	Anterior wing attachment landmark	20	R_3 vein terminus landmark
2	Proximal costal semilandmark I	21	Medial posterior margin semilandmark I
3	Proximal costal semilandmark II	22	Medial posterior margin semilandmark II
4	Proximal costal semilandmark III	23	Medial posterior margin semilandmark III
5	Proximal costal semilandmark IV	24	Medial posterior margin semilandmark IV
6	Nodus landmark	25	Triangle apex (forewings), anal loop terminus (hindwings), projection landmark
7	Distal costal semilandmark I	26	Proximal posterior margin semilandmark I
8	Distal costal semilandmark II	27	Proximal posterior margin semilandmark II
9	Distal costal semilandmark III	28	Proximal posterior margin semilandmark III
10	Distal costal semilandmark IV	29	Proximal posterior margin semilandmark IV
11	Distal costal semilandmark V	30	Proximal posterior margin semilandmark V
12	Distal costal semilandmark VI	31	Posterior wing attachment landmark
13	Costal vein terminus landmark	32	Descending nodus vein - radius + media vein vertex landmark
14	Distal posterior margin semilandmark I	33	R_3 bifurcation vertex landmark
15	Distal posterior margin semilandmark II	34	Distal IR_3 landmark
16	Distal posterior margin semilandmark III	35	Triangle apex (forewings), posterior anal loop terminus (hindwings)
17	Distal posterior margin semilandmark IV	36	Proximal cubitus first intersection landmark
18	Distal posterior margin semilandmark V	37	Anal area triangle base landmark
19	Distal posterior margin semilandmark VI	38	Proximal cubitus second intersection landmark
