# Supplementary figures and images for: What you sample is what you get: ecomorphological variation in Trithemis (Odonata, Libellulidae) dragonfly wings reconsidered (part 3 of 3)
Source: BMC Ecol Evol. 2022 Apr 11;22:43. doi: 10.1186/s12862-022-01978-y (PMC8996507; doi:10.1186/s12862-022-01978-y)

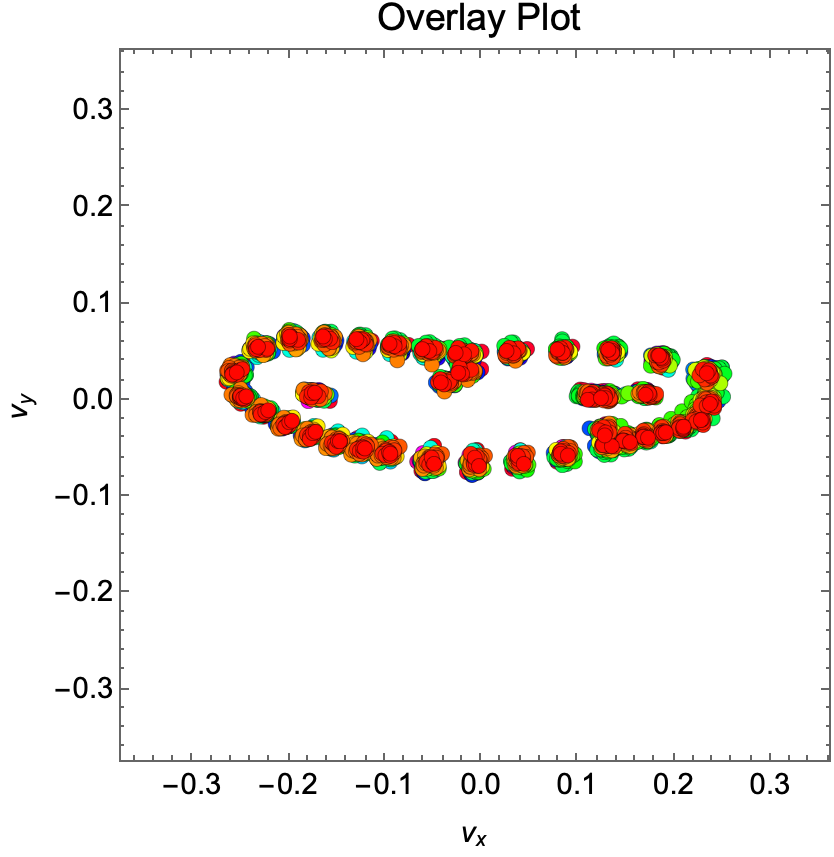

Supplement: Supplementary file 2 — Additional file 2: Datasets and results archive. [file 12862_2022_1978_MOESM2_ESM.zip › Additional Files 2/Datasets & Results Archive/Geometric Morphometrics (Landmarks) Analyses/Forewings/Overlay Plot.tif]

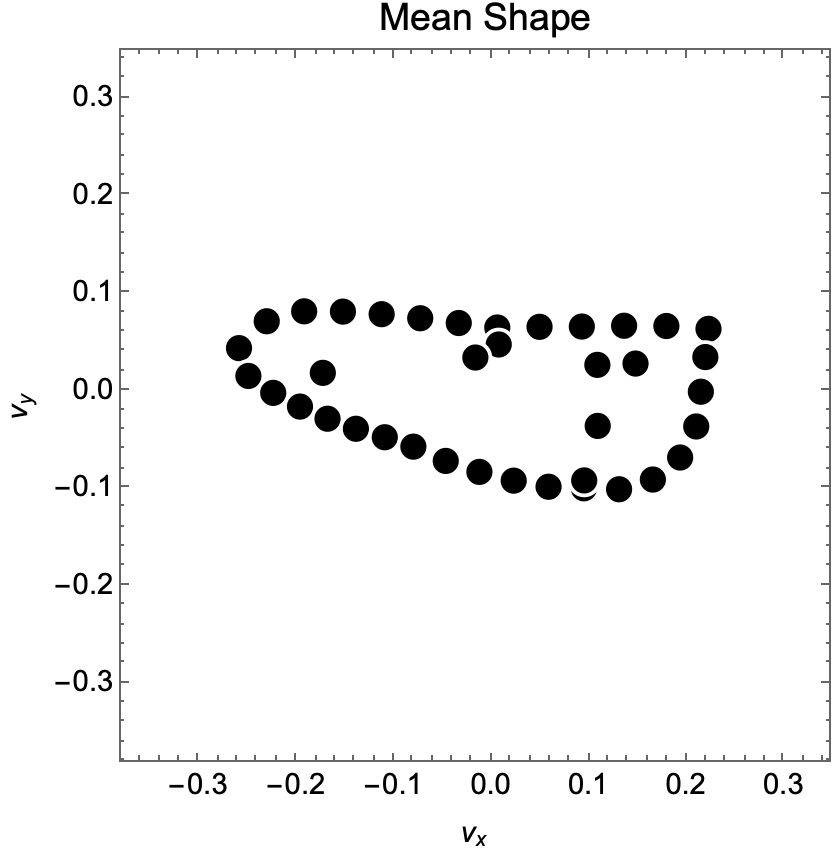

Supplement: Supplementary file 2 — Additional file 2: Datasets and results archive. [file 12862_2022_1978_MOESM2_ESM.zip › Additional Files 2/Datasets & Results Archive/Geometric Morphometrics (Landmarks) Analyses/Hindwings/Mean Shape Plot.tif]

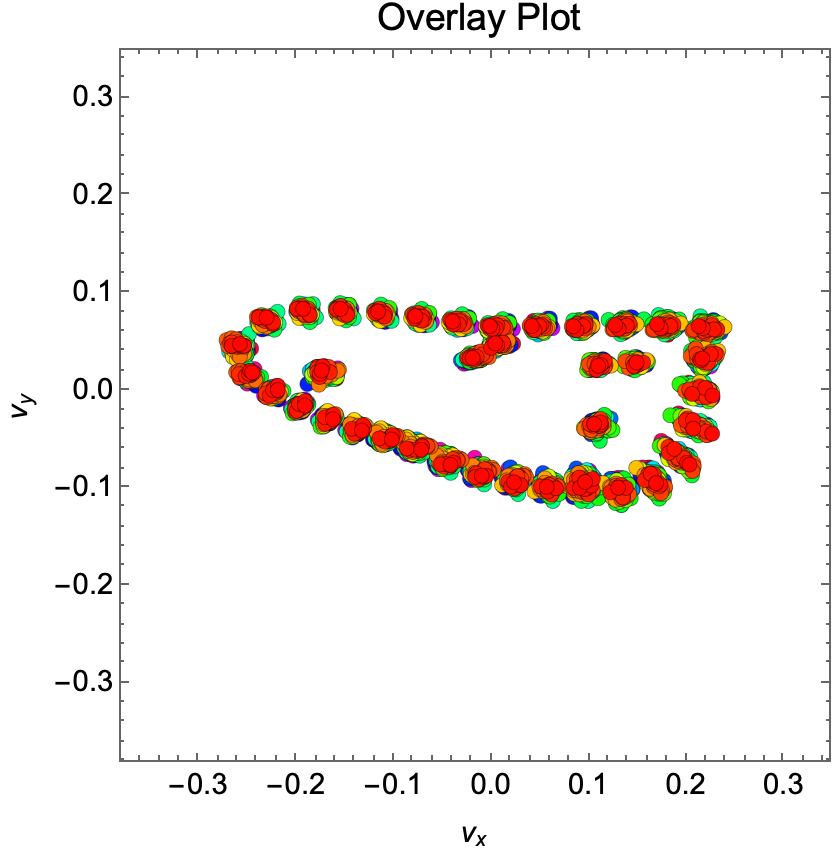

Supplement: Supplementary file 2 — Additional file 2: Datasets and results archive. [file 12862_2022_1978_MOESM2_ESM.zip › Additional Files 2/Datasets & Results Archive/Geometric Morphometrics (Landmarks) Analyses/Hindwings/Overlay Plot.tif]

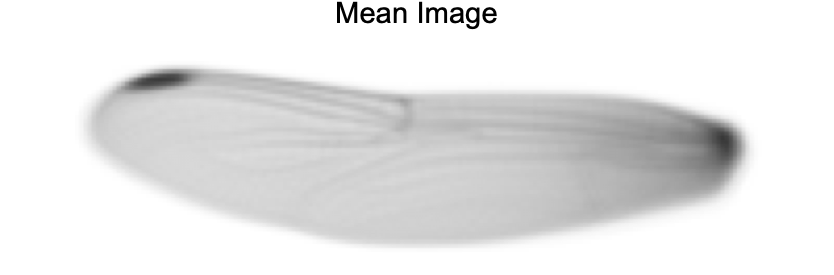

Supplement: Supplementary file 2 — Additional file 2: Datasets and results archive. [file 12862_2022_1978_MOESM2_ESM.zip › Additional Files 2/Datasets & Results Archive/Geometric Morphometric (Images) Analyses/Forewings/Forewing Mean Image.tif]

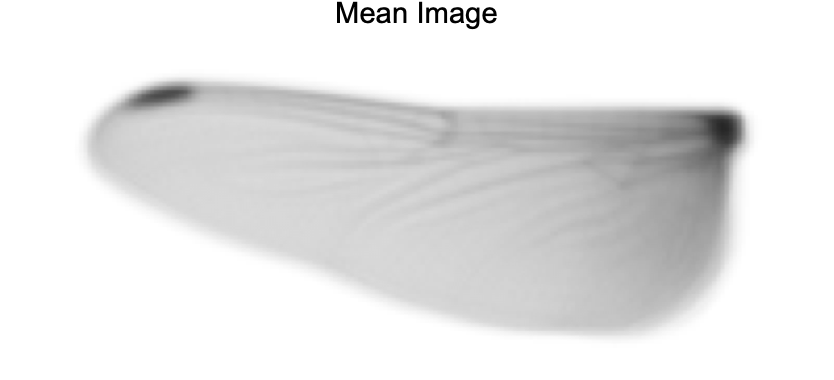

Supplement: Supplementary file 2 — Additional file 2: Datasets and results archive. [file 12862_2022_1978_MOESM2_ESM.zip › Additional Files 2/Datasets & Results Archive/Geometric Morphometric (Images) Analyses/Hindwings/Hindwings Mean Image.tif]

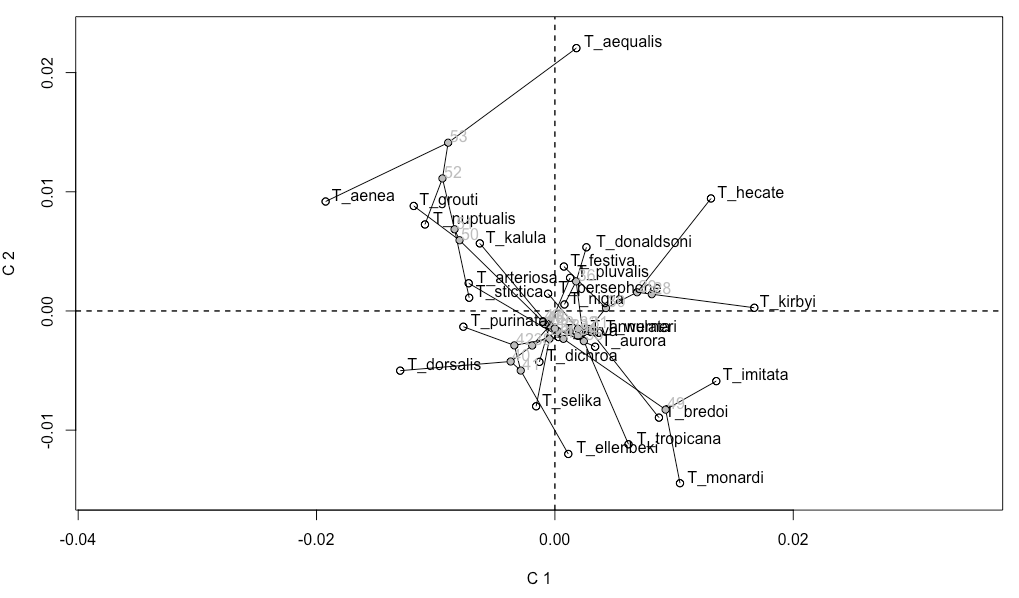

Supplement: Supplementary file 2 — Additional file 2: Datasets and results archive. [file 12862_2022_1978_MOESM2_ESM.zip › Additional Files 2/Datasets & Results Archive/Geometric Morphometrics (Landmarks) Analyses/Phylogenetic Covariation/Hindwings Phylogenetic Signal Tests/C-1 vs C-2 (w: Tree).tiff]

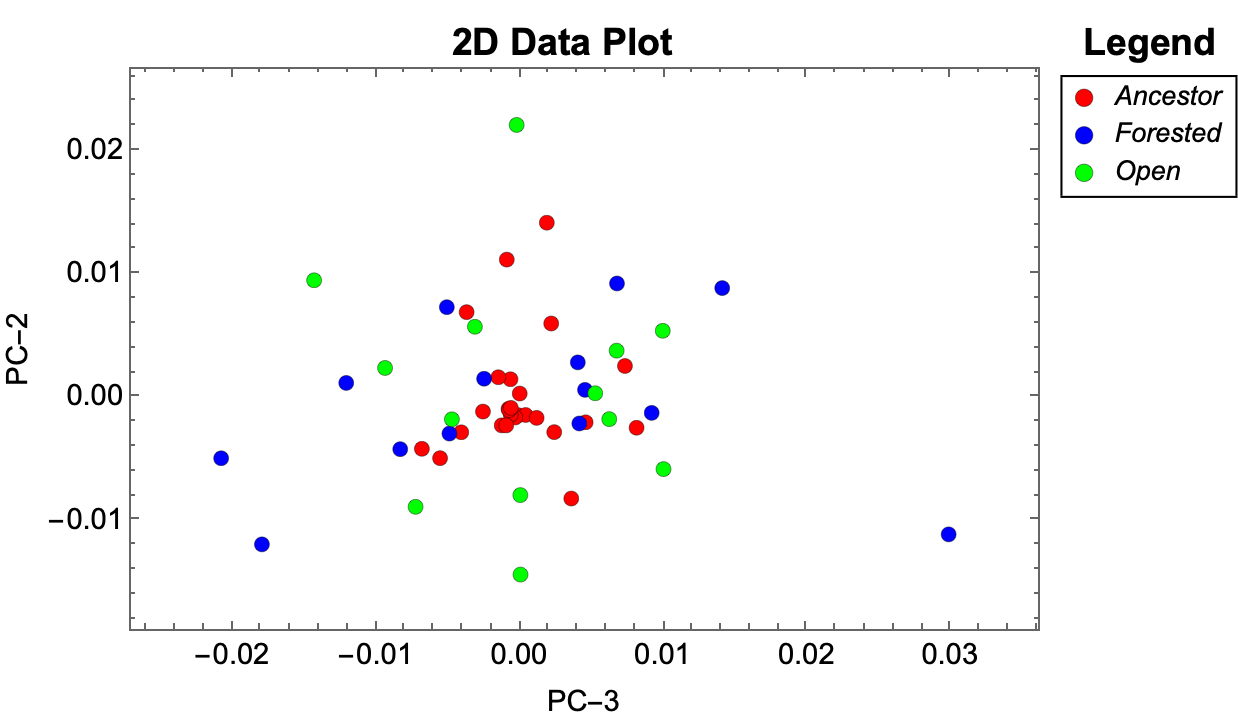

Supplement: Supplementary file 2 — Additional file 2: Datasets and results archive. [file 12862_2022_1978_MOESM2_ESM.zip › Additional Files 2/Datasets & Results Archive/Geometric Morphometrics (Landmarks) Analyses/Phylogenetic Covariation/Hindwings Phylogenetic Signal Tests/PC-3 vs PC-2 (Landscape Groups).tif]

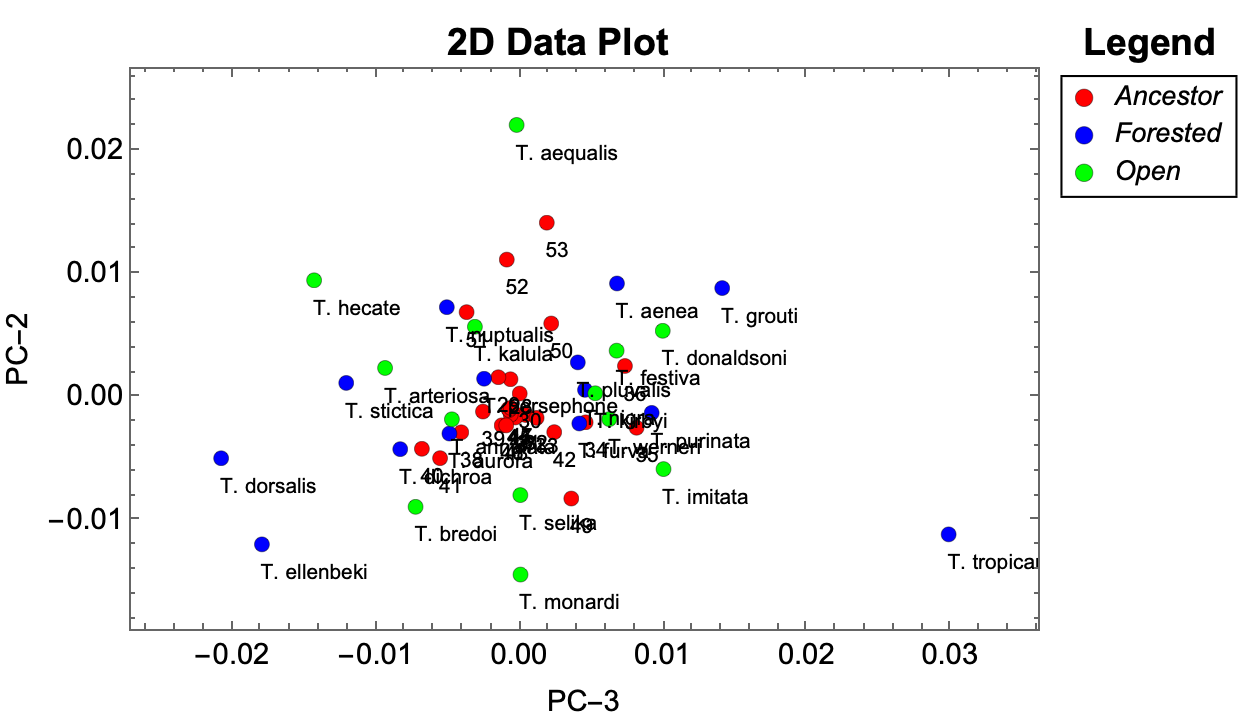

Supplement: Supplementary file 2 — Additional file 2: Datasets and results archive. [file 12862_2022_1978_MOESM2_ESM.zip › Additional Files 2/Datasets & Results Archive/Geometric Morphometrics (Landmarks) Analyses/Phylogenetic Covariation/Hindwings Phylogenetic Signal Tests/PC-3 vs PC-2 (Landscape Groups w: Lables).tif]

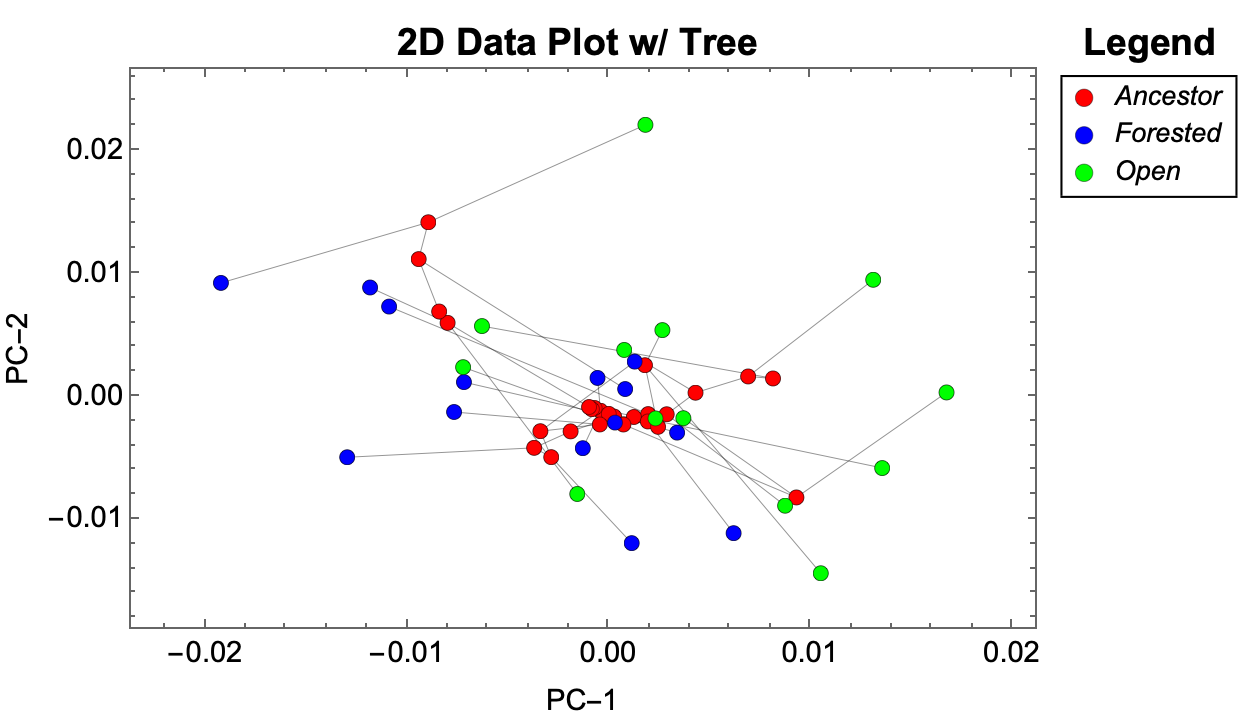

Supplement: Supplementary file 2 — Additional file 2: Datasets and results archive. [file 12862_2022_1978_MOESM2_ESM.zip › Additional Files 2/Datasets & Results Archive/Geometric Morphometrics (Landmarks) Analyses/Phylogenetic Covariation/Hindwings Phylogenetic Signal Tests/PC-1 vs PC-2 (Landscape Groups w: Tree).tif]

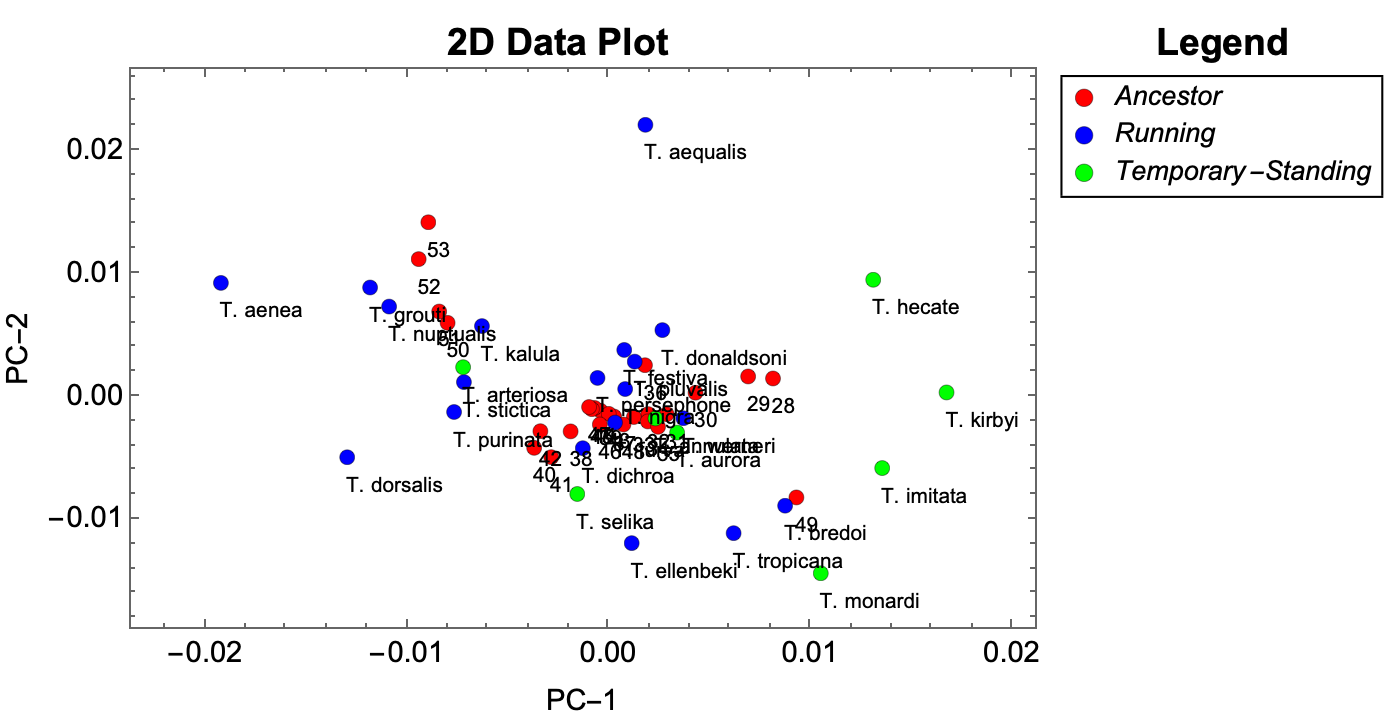

Supplement: Supplementary file 2 — Additional file 2: Datasets and results archive. [file 12862_2022_1978_MOESM2_ESM.zip › Additional Files 2/Datasets & Results Archive/Geometric Morphometrics (Landmarks) Analyses/Phylogenetic Covariation/Hindwings Phylogenetic Signal Tests/PC-1 vs PC-2 (Water Body Groups w: Lables).tif]

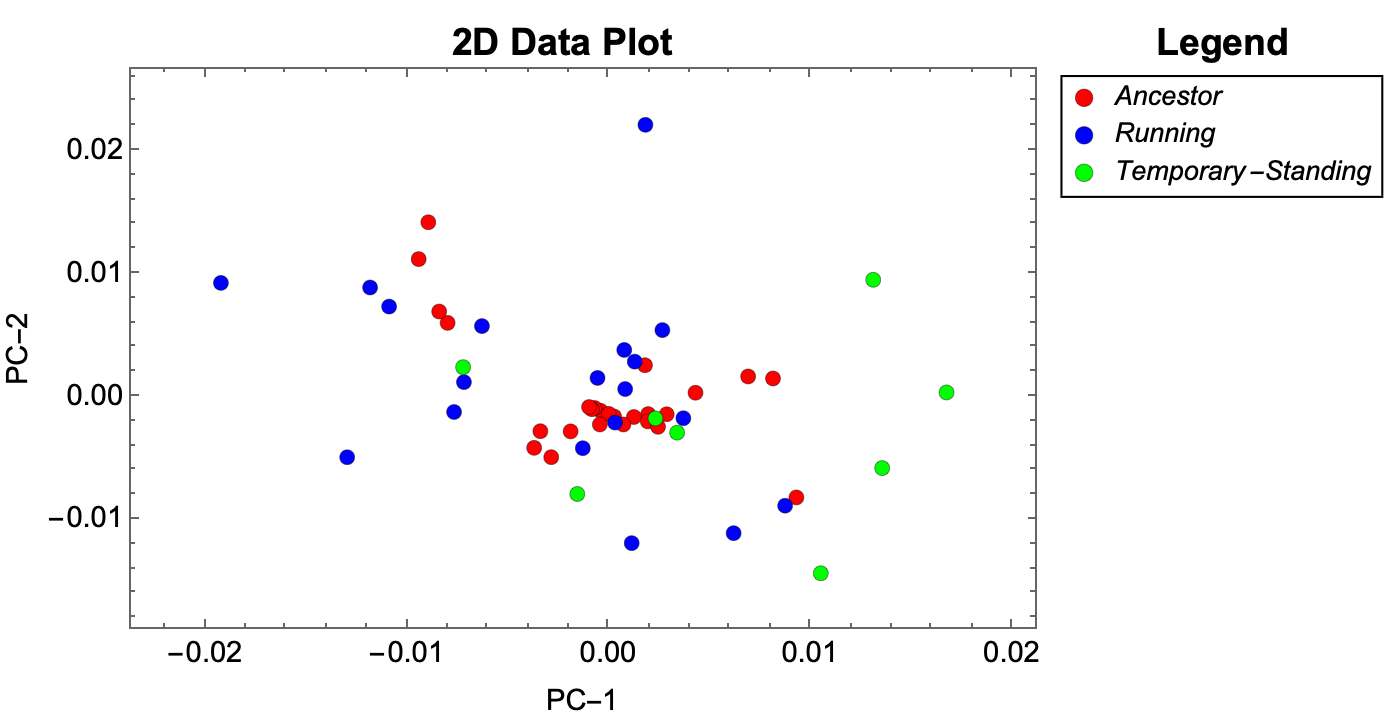

Supplement: Supplementary file 2 — Additional file 2: Datasets and results archive. [file 12862_2022_1978_MOESM2_ESM.zip › Additional Files 2/Datasets & Results Archive/Geometric Morphometrics (Landmarks) Analyses/Phylogenetic Covariation/Hindwings Phylogenetic Signal Tests/PC-1 vs PC-2 (Water Body Groups).tif]

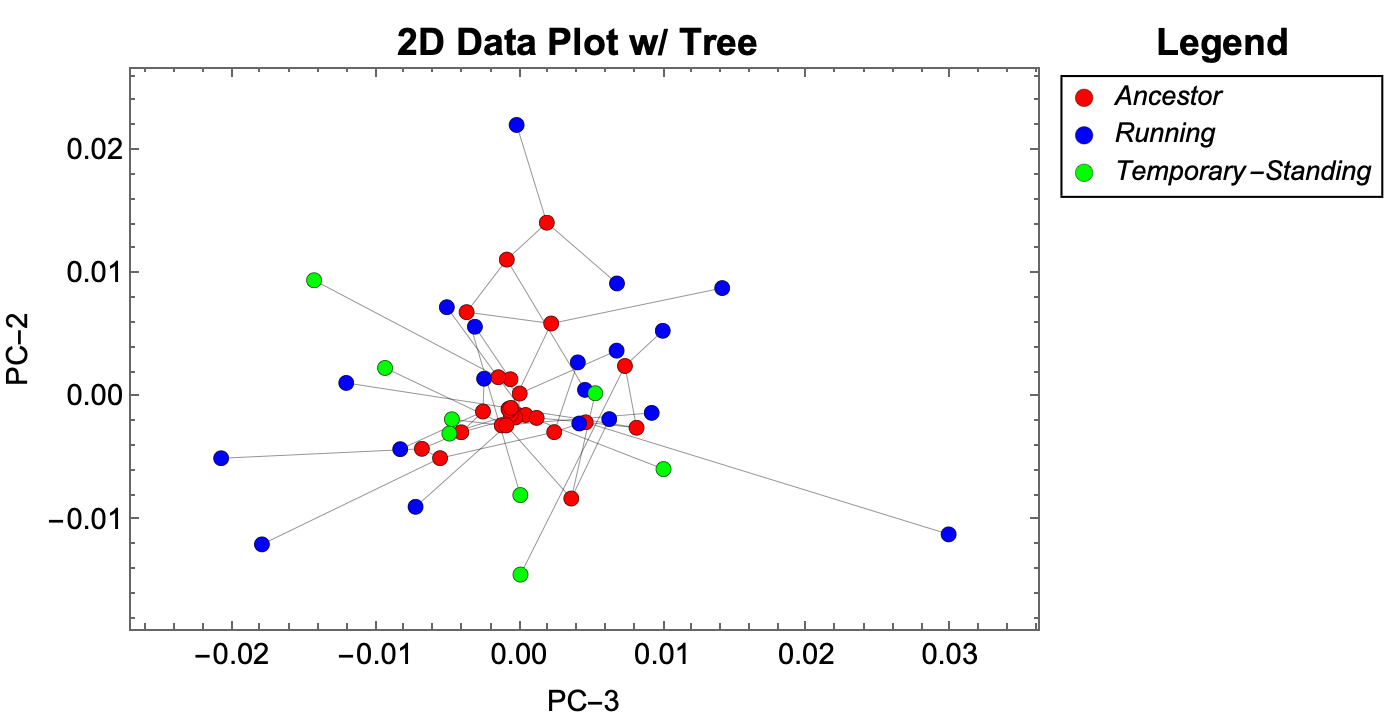

Supplement: Supplementary file 2 — Additional file 2: Datasets and results archive. [file 12862_2022_1978_MOESM2_ESM.zip › Additional Files 2/Datasets & Results Archive/Geometric Morphometrics (Landmarks) Analyses/Phylogenetic Covariation/Hindwings Phylogenetic Signal Tests/PC-3 vs PC-2 (Water Body Groups w: Tree).tif]

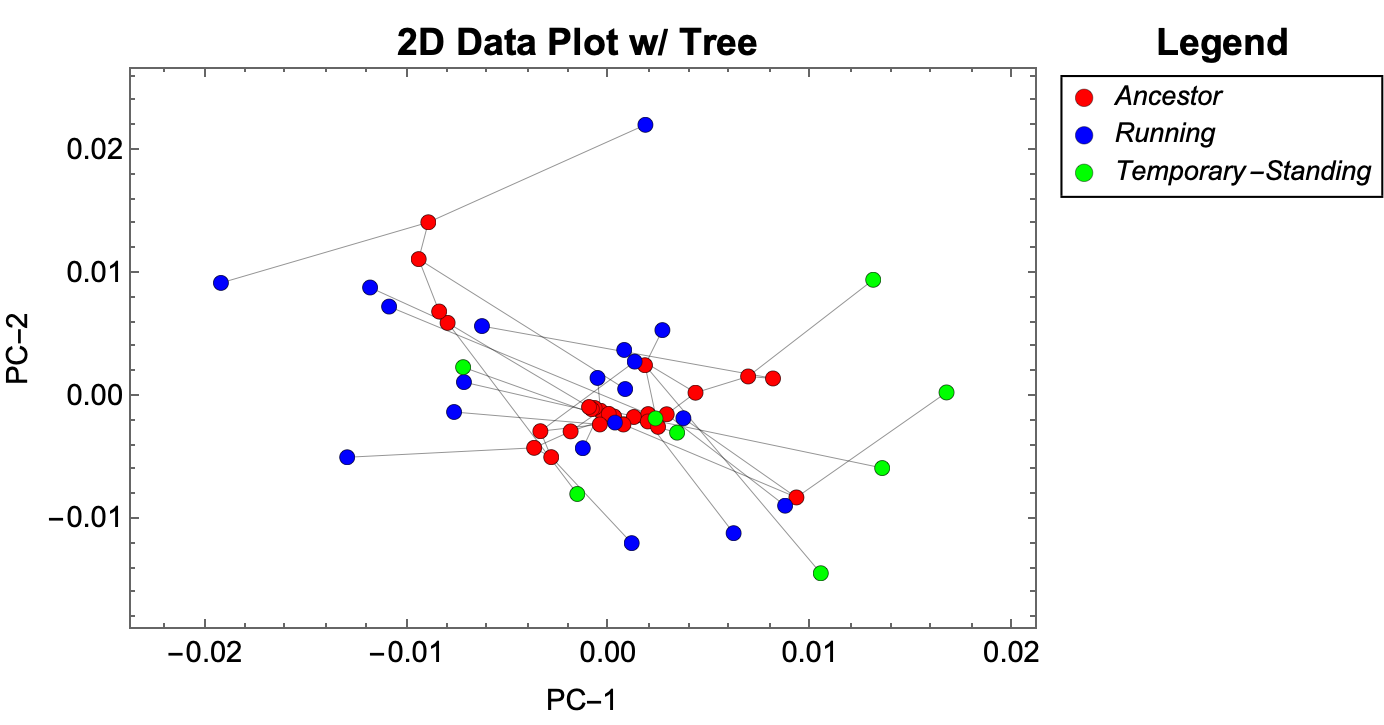

Supplement: Supplementary file 2 — Additional file 2: Datasets and results archive. [file 12862_2022_1978_MOESM2_ESM.zip › Additional Files 2/Datasets & Results Archive/Geometric Morphometrics (Landmarks) Analyses/Phylogenetic Covariation/Hindwings Phylogenetic Signal Tests/PC-1 vs PC-2 (Water Body Groups w: Tree).tif]

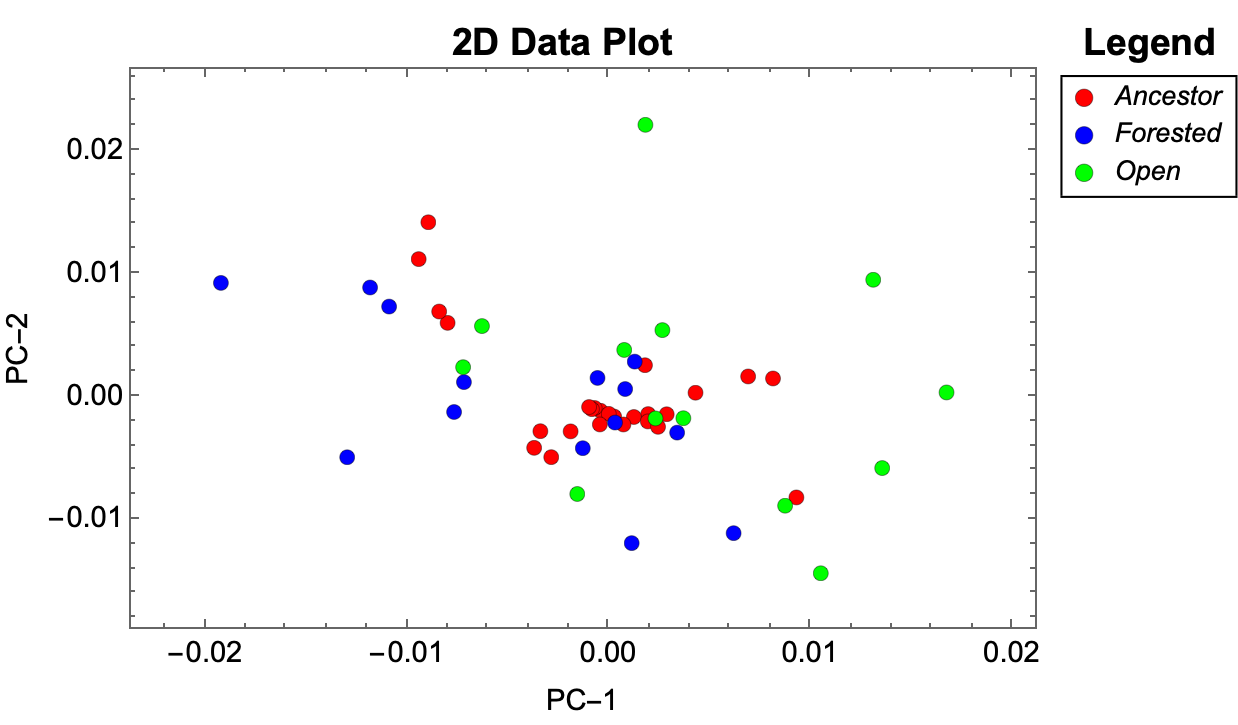

Supplement: Supplementary file 2 — Additional file 2: Datasets and results archive. [file 12862_2022_1978_MOESM2_ESM.zip › Additional Files 2/Datasets & Results Archive/Geometric Morphometrics (Landmarks) Analyses/Phylogenetic Covariation/Hindwings Phylogenetic Signal Tests/PC-1 vs PC-2 (Landscape Groups).tif]

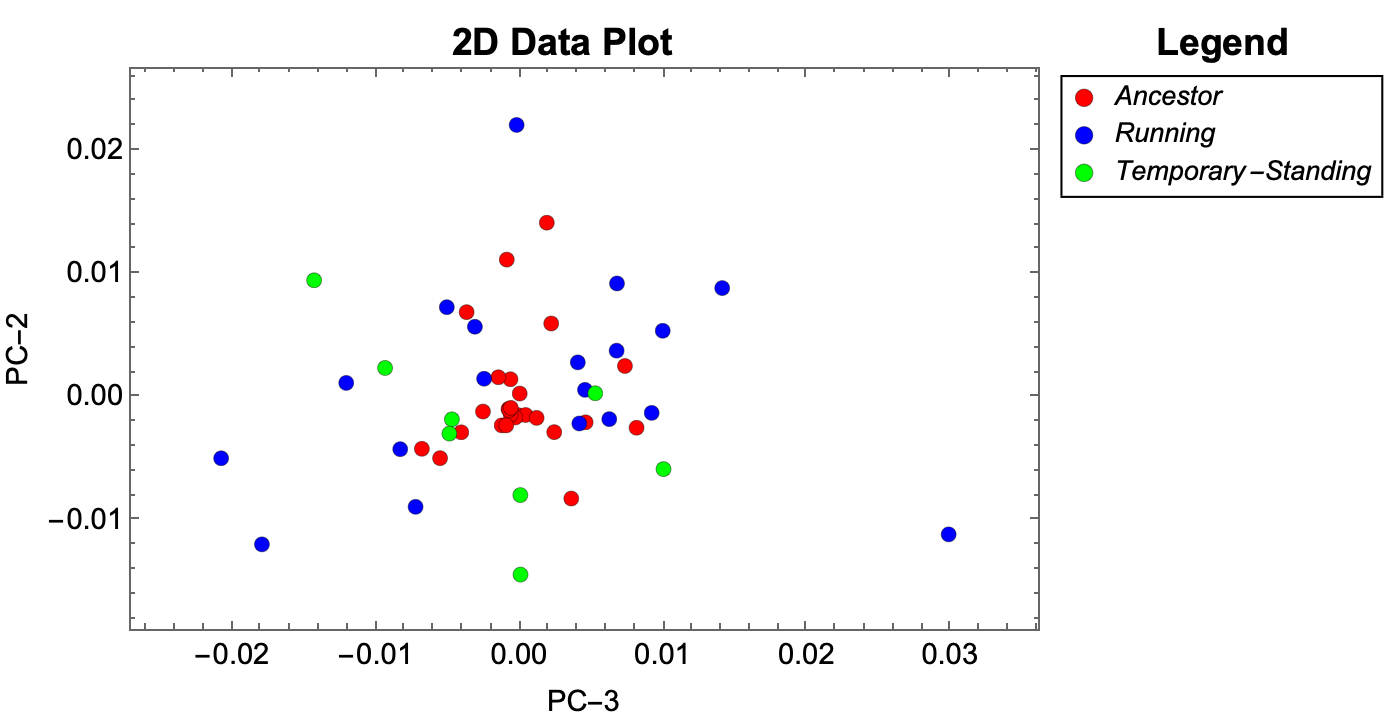

Supplement: Supplementary file 2 — Additional file 2: Datasets and results archive. [file 12862_2022_1978_MOESM2_ESM.zip › Additional Files 2/Datasets & Results Archive/Geometric Morphometrics (Landmarks) Analyses/Phylogenetic Covariation/Hindwings Phylogenetic Signal Tests/PC-3 vs PC-2 (Water Body Groups).tif]

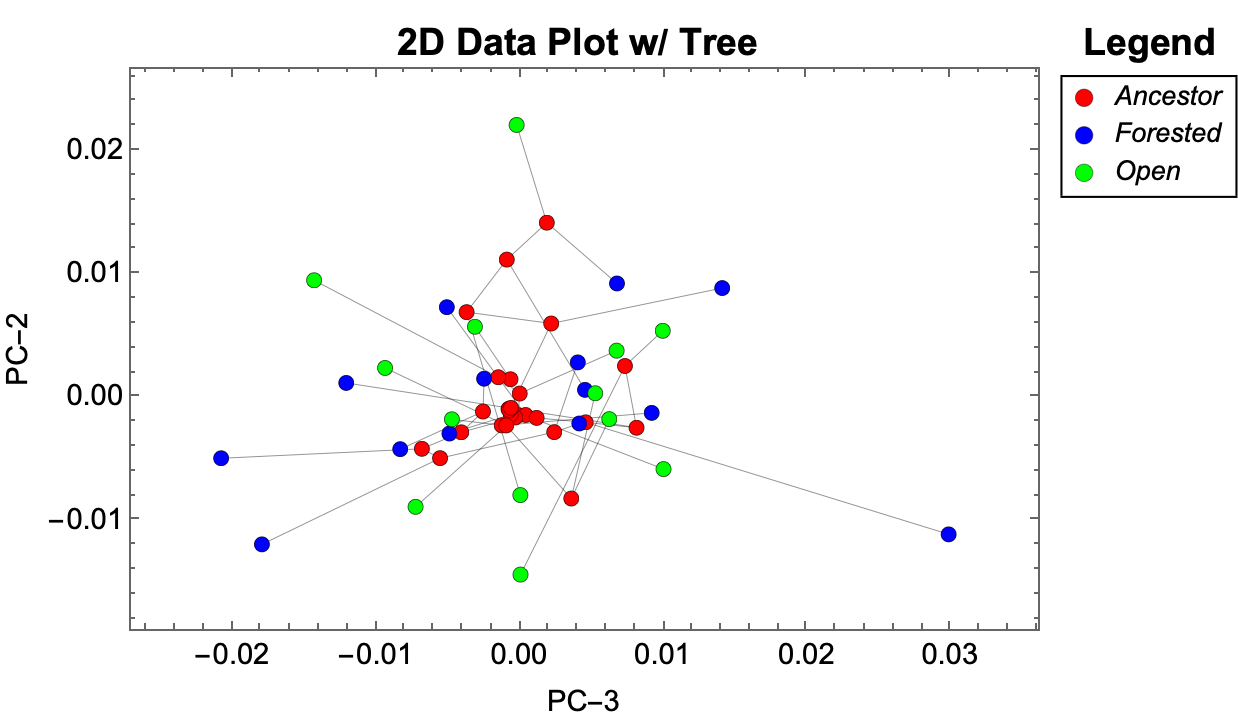

Supplement: Supplementary file 2 — Additional file 2: Datasets and results archive. [file 12862_2022_1978_MOESM2_ESM.zip › Additional Files 2/Datasets & Results Archive/Geometric Morphometrics (Landmarks) Analyses/Phylogenetic Covariation/Hindwings Phylogenetic Signal Tests/PC-3 vs PC-2 (Landscape Groups w: Tree).tif]

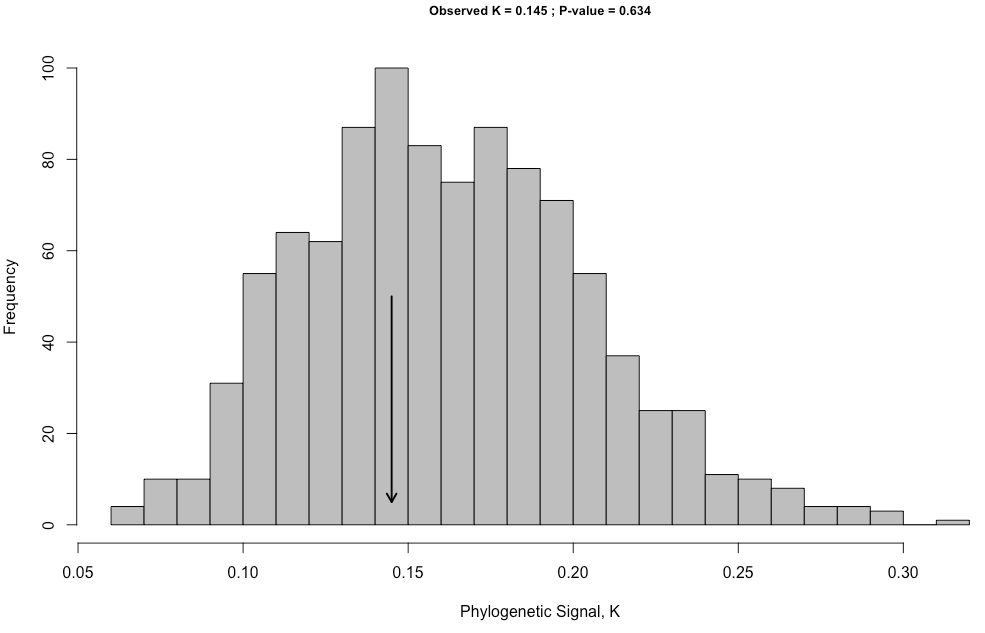

Supplement: Supplementary file 2 — Additional file 2: Datasets and results archive. [file 12862_2022_1978_MOESM2_ESM.zip › Additional Files 2/Datasets & Results Archive/Geometric Morphometrics (Landmarks) Analyses/Phylogenetic Covariation/Hindwings Phylogenetic Signal Tests/K-Statistic Plot (BS).tiff]

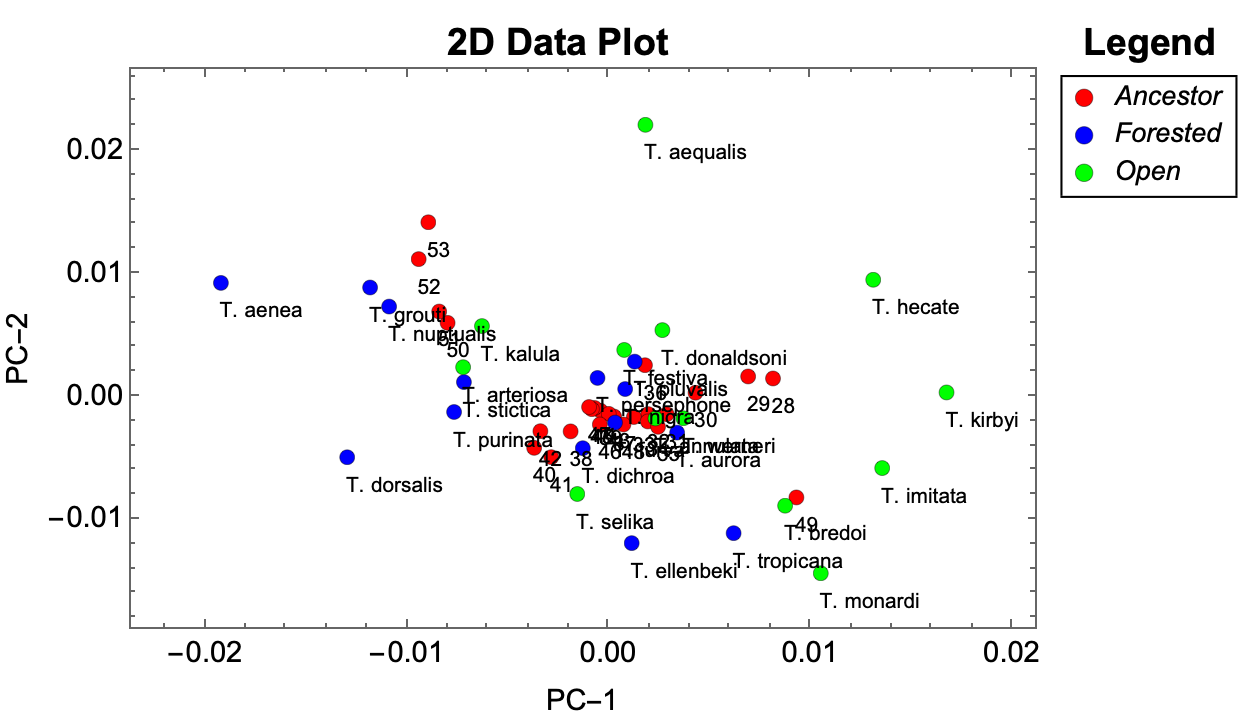

Supplement: Supplementary file 2 — Additional file 2: Datasets and results archive. [file 12862_2022_1978_MOESM2_ESM.zip › Additional Files 2/Datasets & Results Archive/Geometric Morphometrics (Landmarks) Analyses/Phylogenetic Covariation/Hindwings Phylogenetic Signal Tests/PC-1 vs PC-2 (Landscape Groups w: Lables).tif]

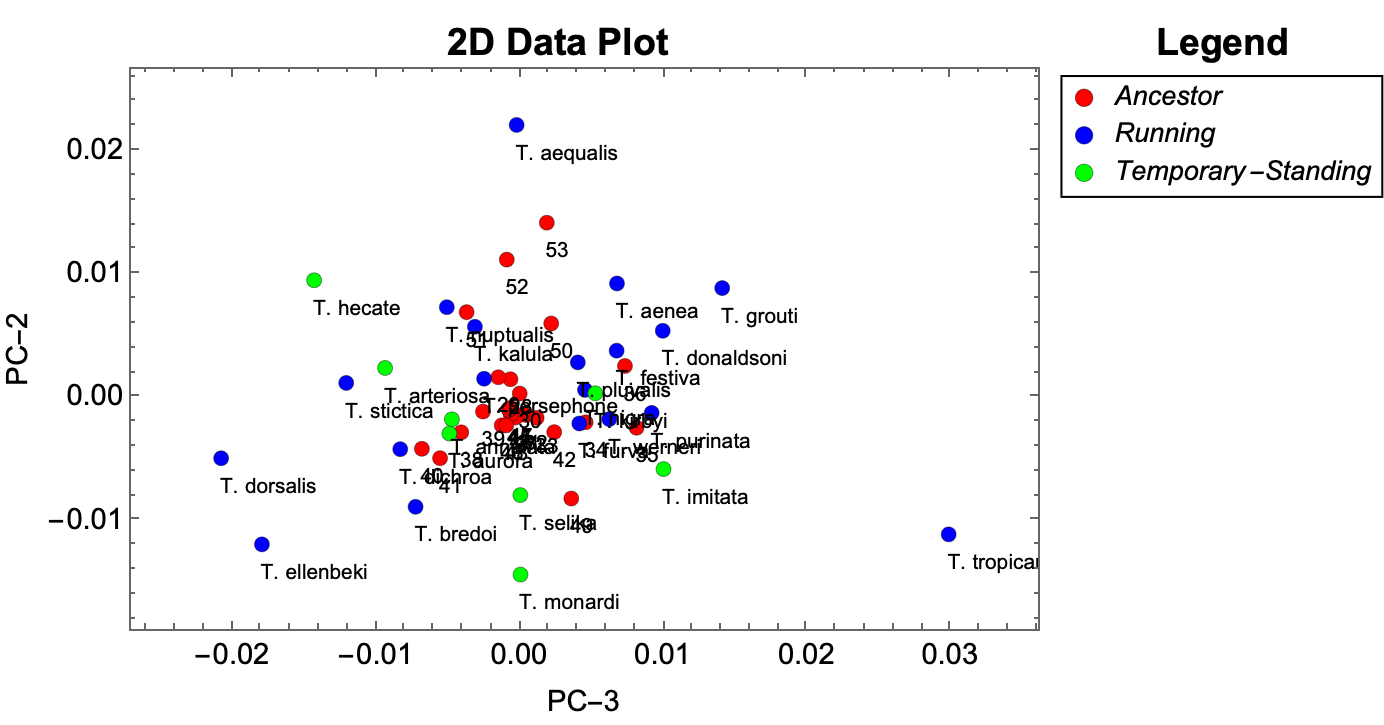

Supplement: Supplementary file 2 — Additional file 2: Datasets and results archive. [file 12862_2022_1978_MOESM2_ESM.zip › Additional Files 2/Datasets & Results Archive/Geometric Morphometrics (Landmarks) Analyses/Phylogenetic Covariation/Hindwings Phylogenetic Signal Tests/PC-3 vs PC-2 (Water Body Groups w: Lables).tif]

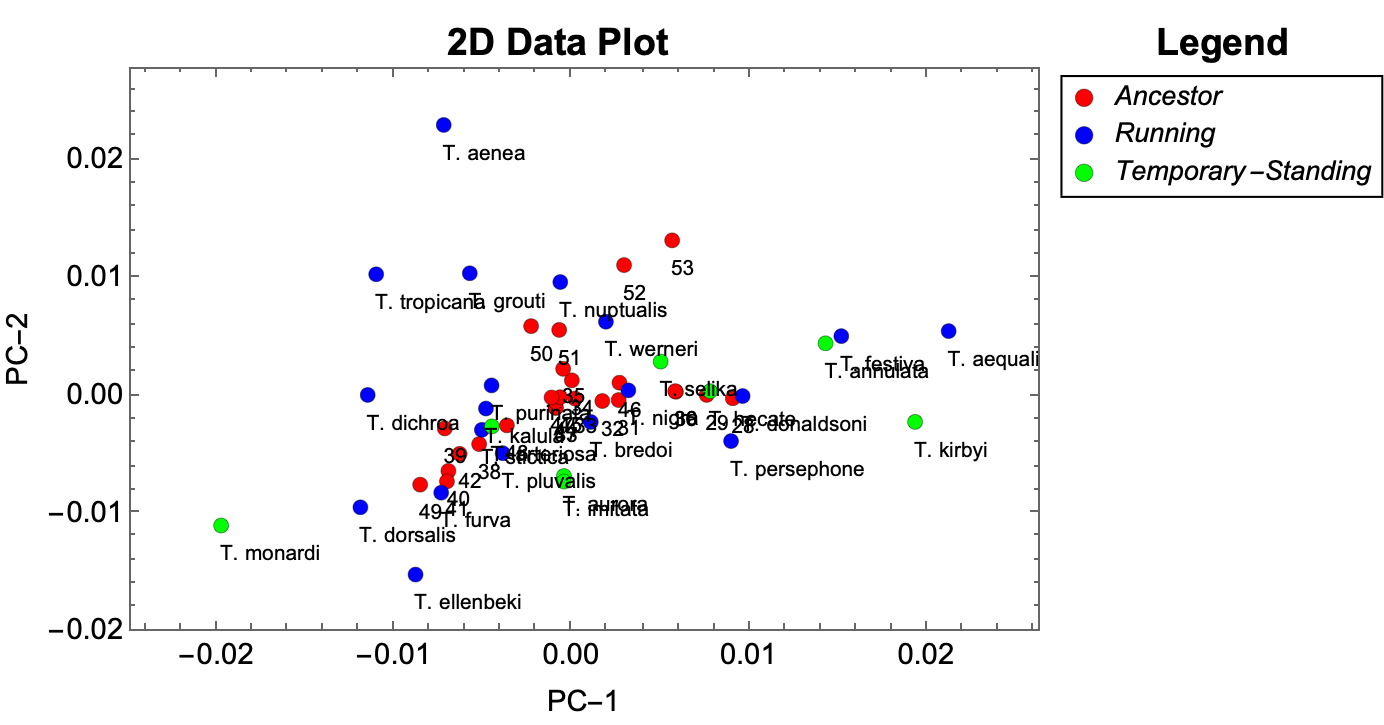

Supplement: Supplementary file 2 — Additional file 2: Datasets and results archive. [file 12862_2022_1978_MOESM2_ESM.zip › Additional Files 2/Datasets & Results Archive/Geometric Morphometrics (Landmarks) Analyses/Phylogenetic Covariation/Forewings Phylogenetic Signal Tests/PC-1 vs PC-2 (Water Body Groups w: Labels).tif]

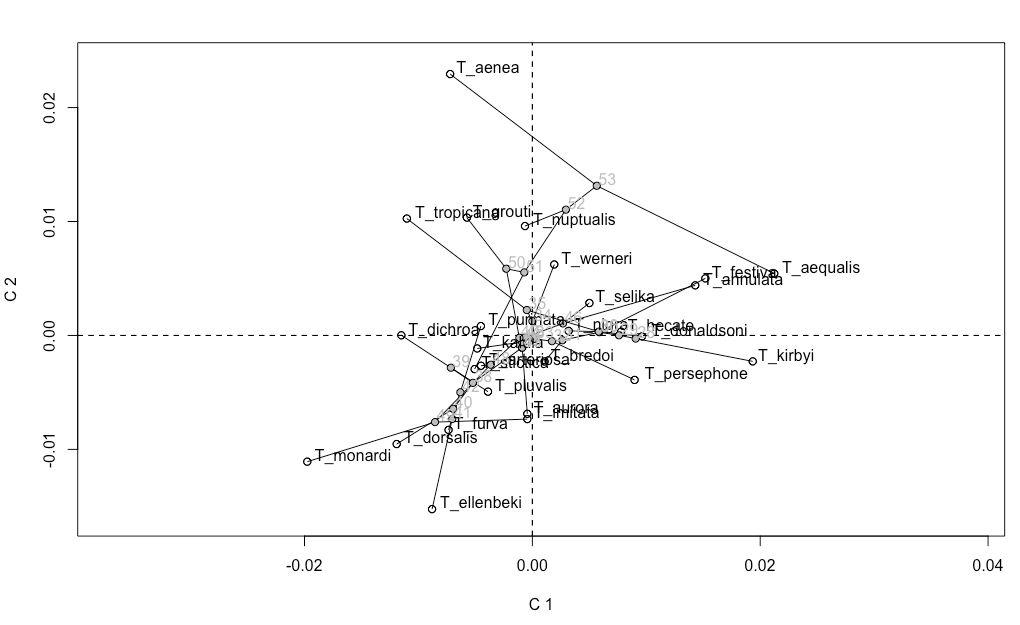

Supplement: Supplementary file 2 — Additional file 2: Datasets and results archive. [file 12862_2022_1978_MOESM2_ESM.zip › Additional Files 2/Datasets & Results Archive/Geometric Morphometrics (Landmarks) Analyses/Phylogenetic Covariation/Forewings Phylogenetic Signal Tests/C-1 vs C-2 (w: Tree).tiff]

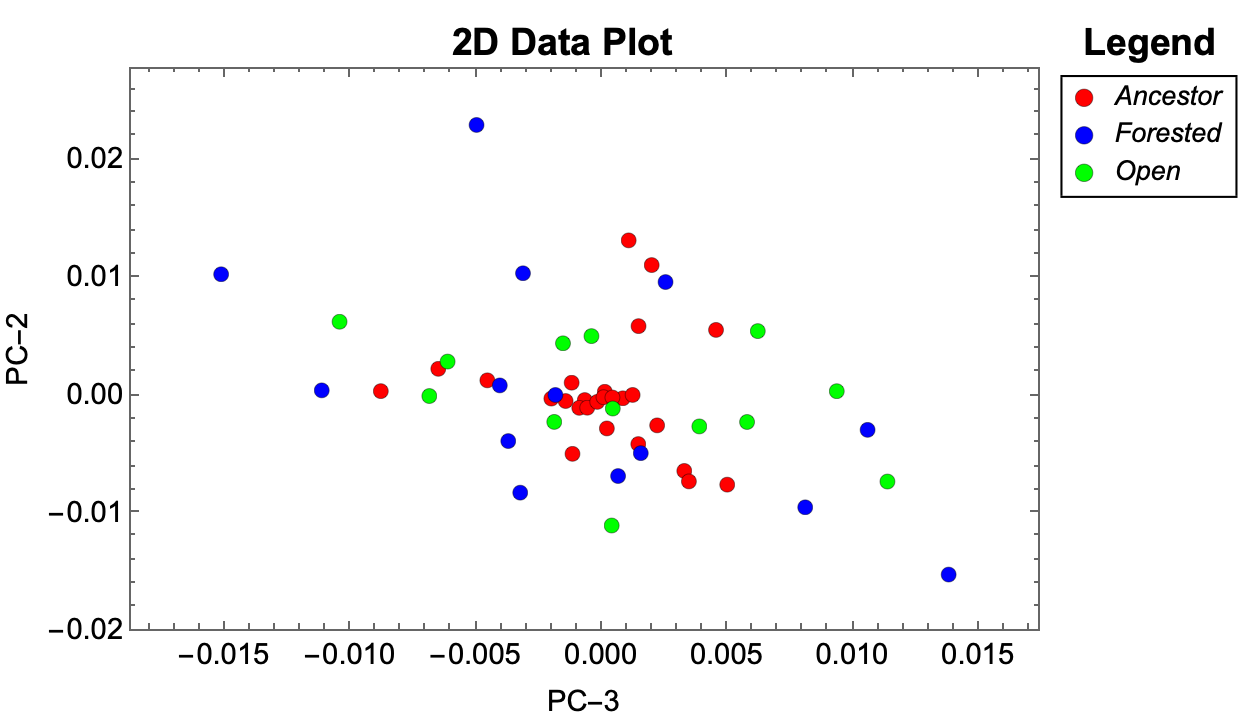

Supplement: Supplementary file 2 — Additional file 2: Datasets and results archive. [file 12862_2022_1978_MOESM2_ESM.zip › Additional Files 2/Datasets & Results Archive/Geometric Morphometrics (Landmarks) Analyses/Phylogenetic Covariation/Forewings Phylogenetic Signal Tests/PC-3 vs PC-2 (Landscape Groups).tif]

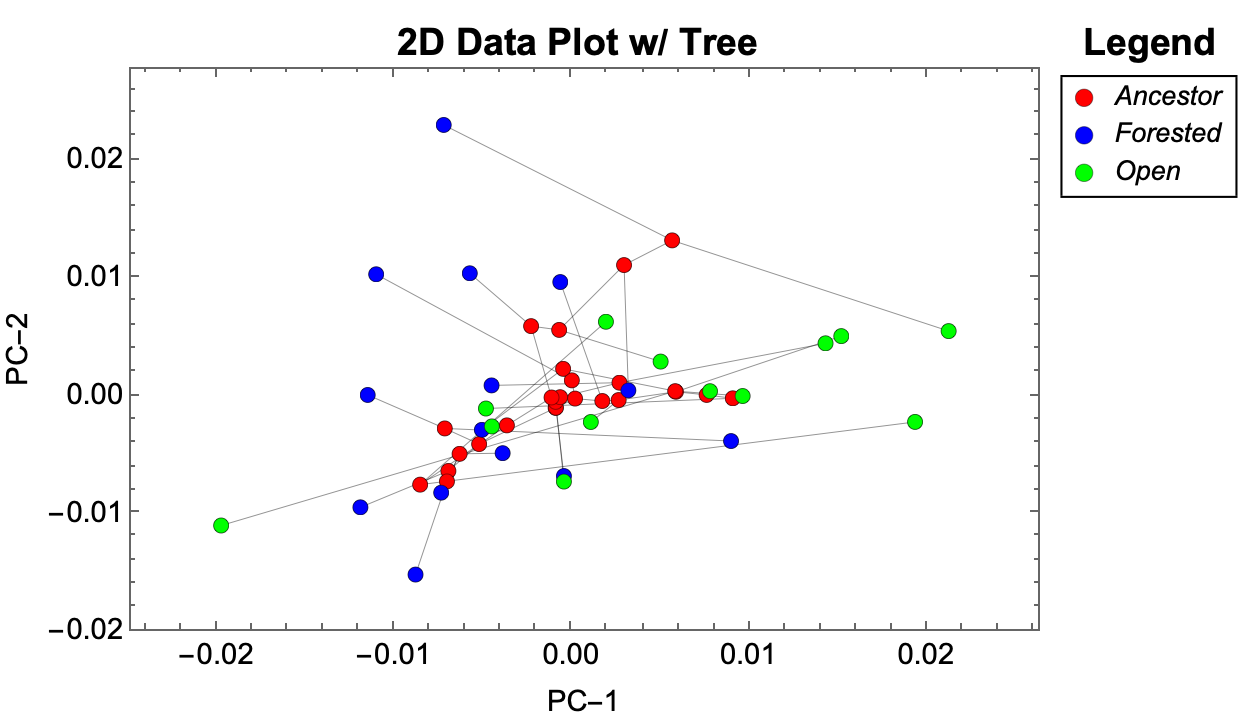

Supplement: Supplementary file 2 — Additional file 2: Datasets and results archive. [file 12862_2022_1978_MOESM2_ESM.zip › Additional Files 2/Datasets & Results Archive/Geometric Morphometrics (Landmarks) Analyses/Phylogenetic Covariation/Forewings Phylogenetic Signal Tests/PC-1 vs PC-2 (Landscape Groups w: Tree).tif]

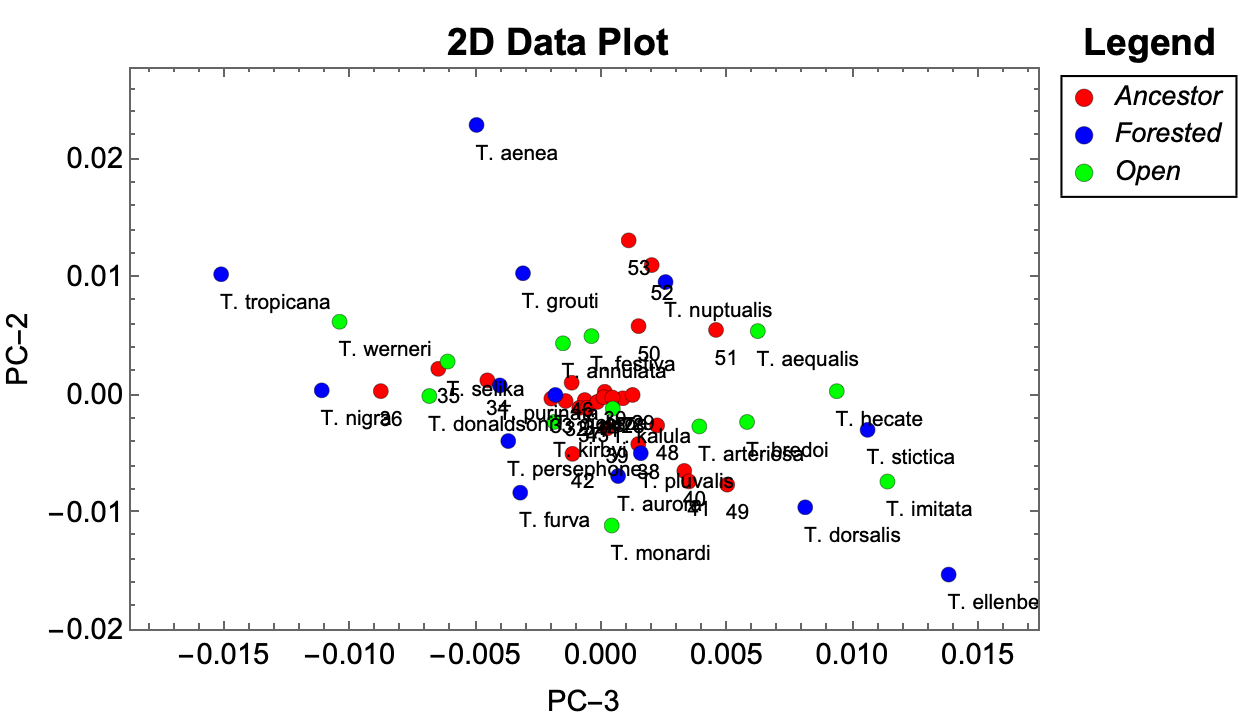

Supplement: Supplementary file 2 — Additional file 2: Datasets and results archive. [file 12862_2022_1978_MOESM2_ESM.zip › Additional Files 2/Datasets & Results Archive/Geometric Morphometrics (Landmarks) Analyses/Phylogenetic Covariation/Forewings Phylogenetic Signal Tests/PC-3 vs PC-2 (Landscape Groups w: Labels).tif]

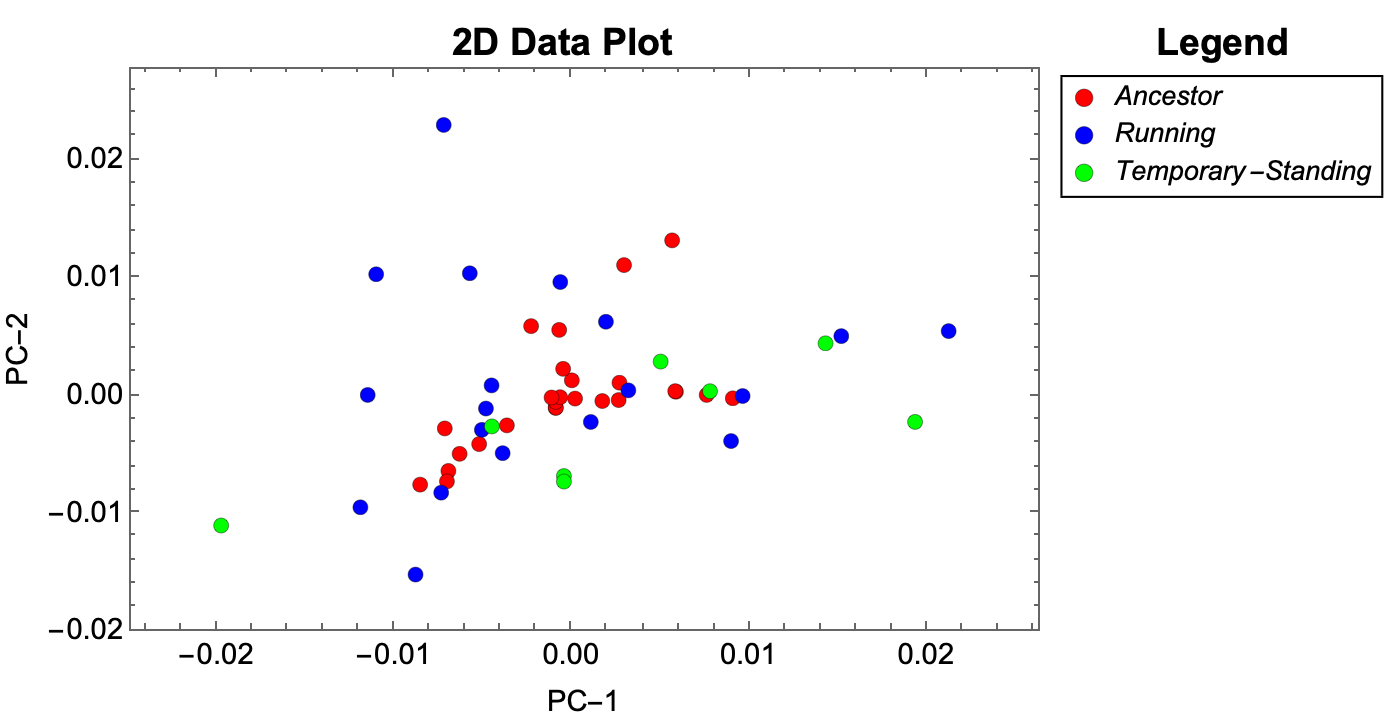

Supplement: Supplementary file 2 — Additional file 2: Datasets and results archive. [file 12862_2022_1978_MOESM2_ESM.zip › Additional Files 2/Datasets & Results Archive/Geometric Morphometrics (Landmarks) Analyses/Phylogenetic Covariation/Forewings Phylogenetic Signal Tests/PC-1 vs PC-2 (Water Body Groups).tif]

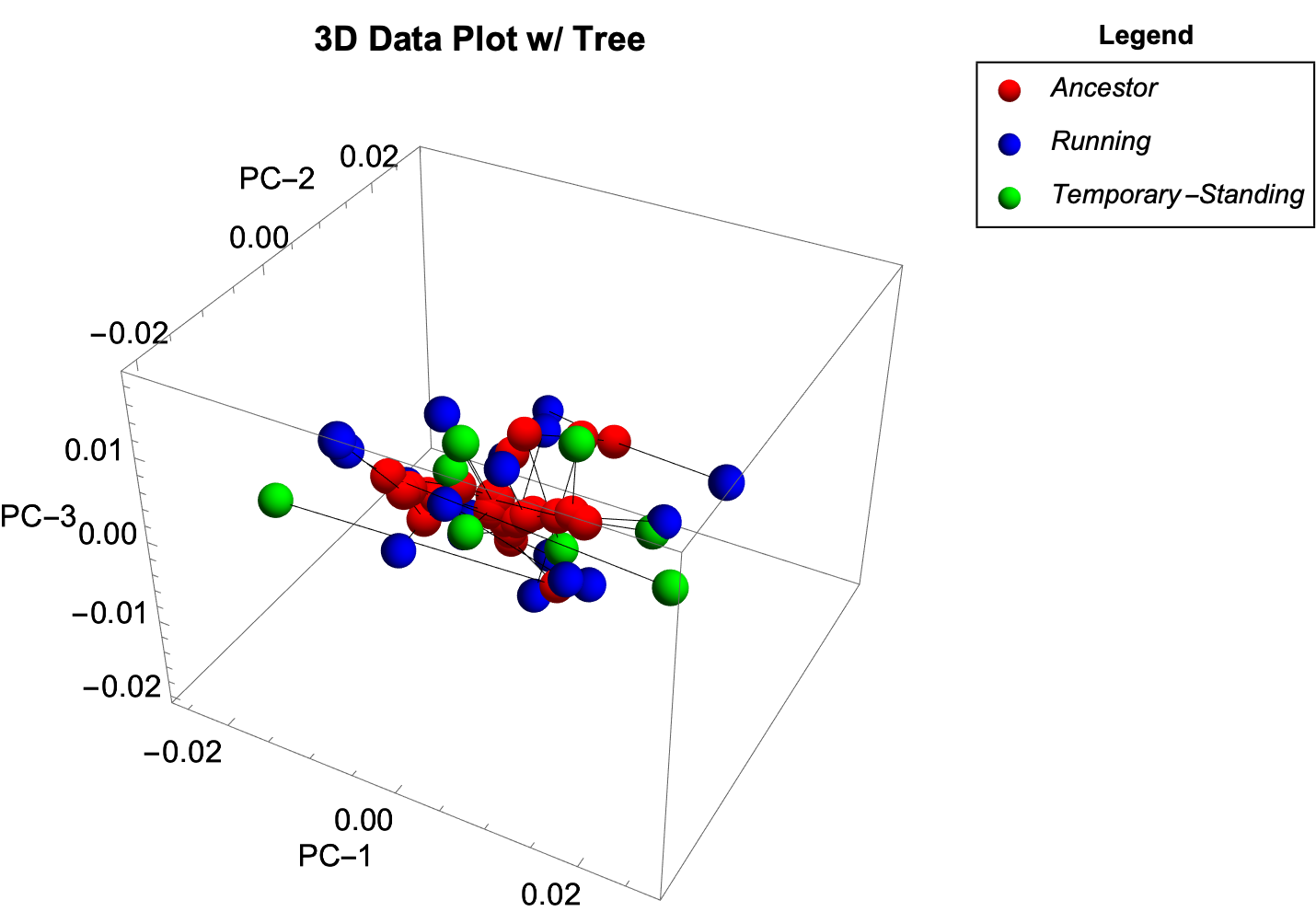

Supplement: Supplementary file 2 — Additional file 2: Datasets and results archive. [file 12862_2022_1978_MOESM2_ESM.zip › Additional Files 2/Datasets & Results Archive/Geometric Morphometrics (Landmarks) Analyses/Phylogenetic Covariation/Forewings Phylogenetic Signal Tests/PC-3 vs PC-2 vs PC-3 (Water Body Groups w: Tree).tif]

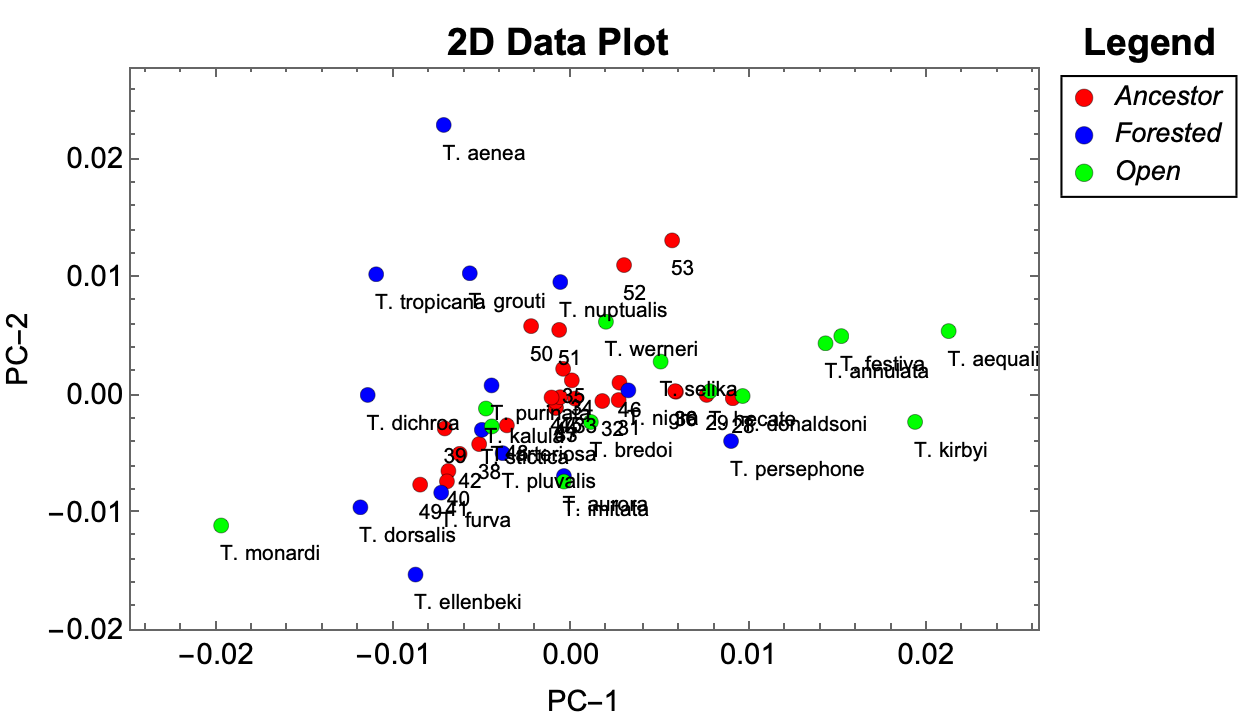

Supplement: Supplementary file 2 — Additional file 2: Datasets and results archive. [file 12862_2022_1978_MOESM2_ESM.zip › Additional Files 2/Datasets & Results Archive/Geometric Morphometrics (Landmarks) Analyses/Phylogenetic Covariation/Forewings Phylogenetic Signal Tests/PC-1 vs PC-2 (Landscape Groups w: Labels).tif]

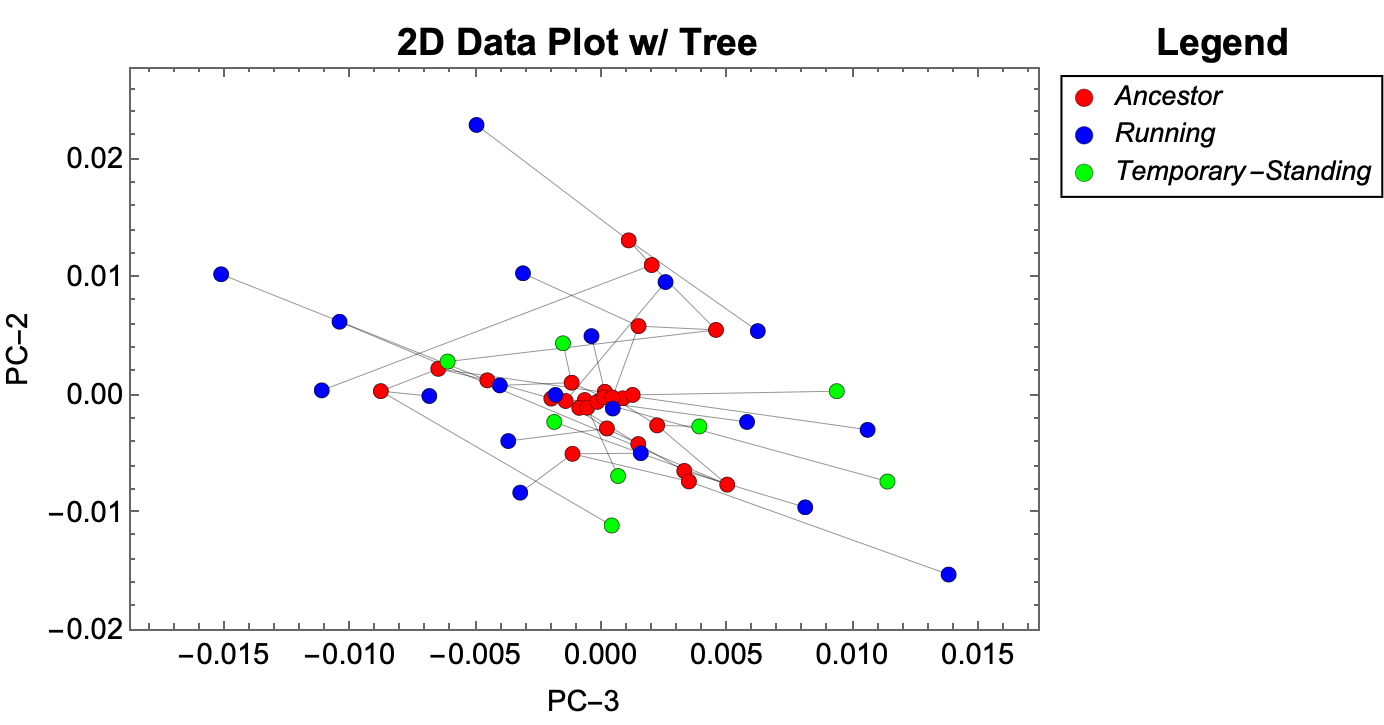

Supplement: Supplementary file 2 — Additional file 2: Datasets and results archive. [file 12862_2022_1978_MOESM2_ESM.zip › Additional Files 2/Datasets & Results Archive/Geometric Morphometrics (Landmarks) Analyses/Phylogenetic Covariation/Forewings Phylogenetic Signal Tests/PC-3 vs PC-2 (Water Body Groups w: Tree).tif]

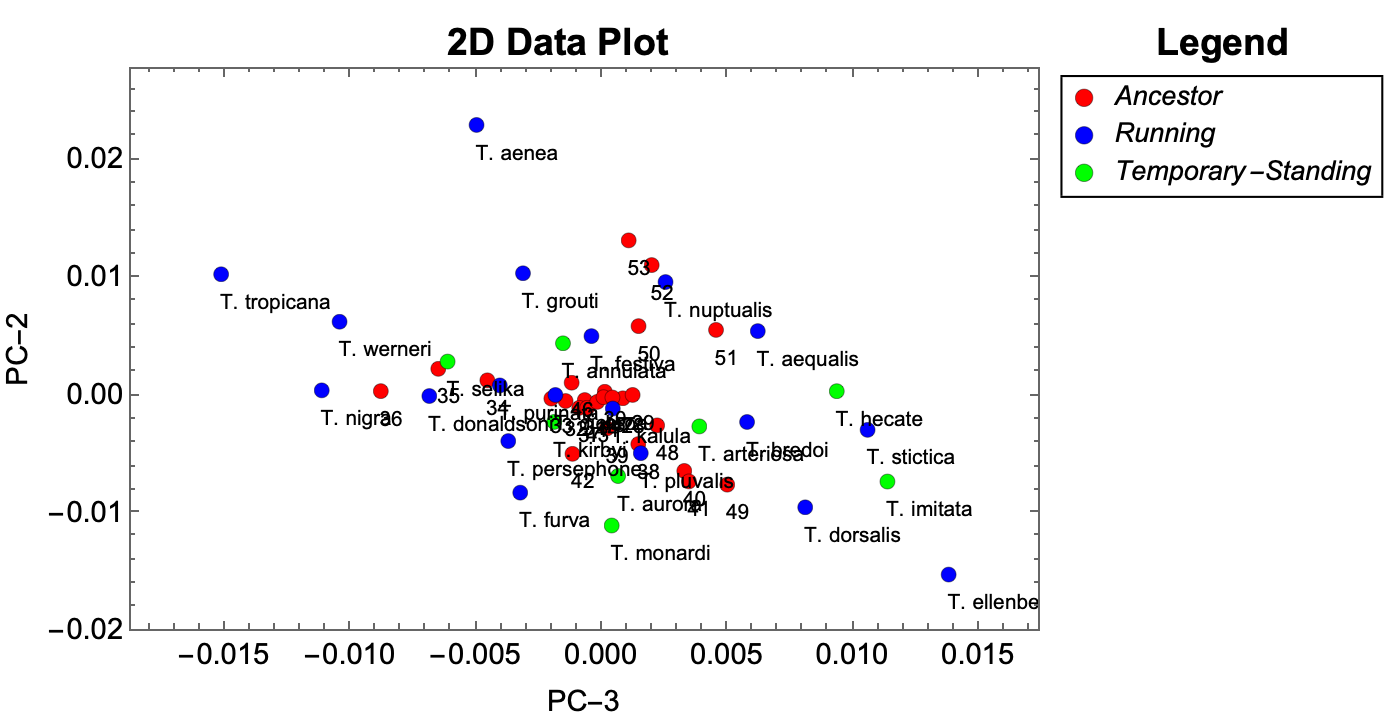

Supplement: Supplementary file 2 — Additional file 2: Datasets and results archive. [file 12862_2022_1978_MOESM2_ESM.zip › Additional Files 2/Datasets & Results Archive/Geometric Morphometrics (Landmarks) Analyses/Phylogenetic Covariation/Forewings Phylogenetic Signal Tests/PC-3 vs PC-2 (Water Body Groups w: Labels).tif]

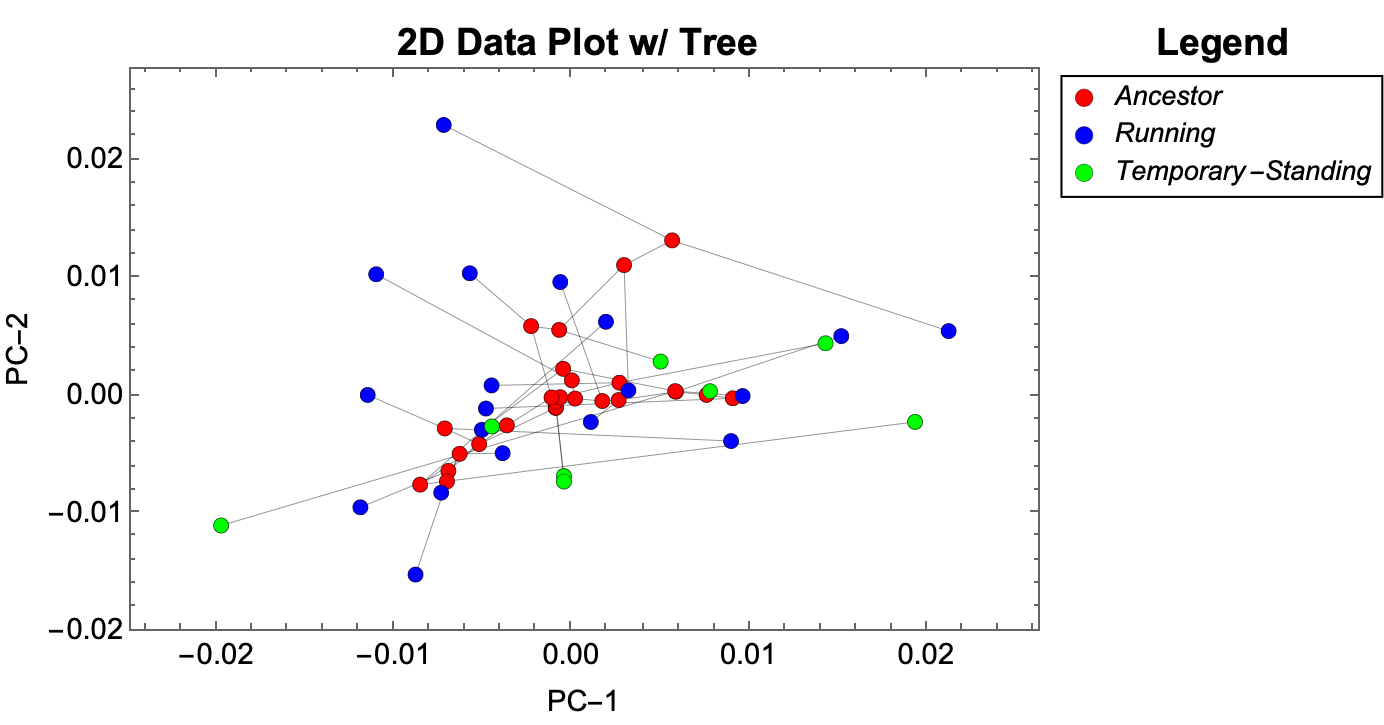

Supplement: Supplementary file 2 — Additional file 2: Datasets and results archive. [file 12862_2022_1978_MOESM2_ESM.zip › Additional Files 2/Datasets & Results Archive/Geometric Morphometrics (Landmarks) Analyses/Phylogenetic Covariation/Forewings Phylogenetic Signal Tests/PC-1 vs PC-2 (Water Body Groups w: Tree).tif]

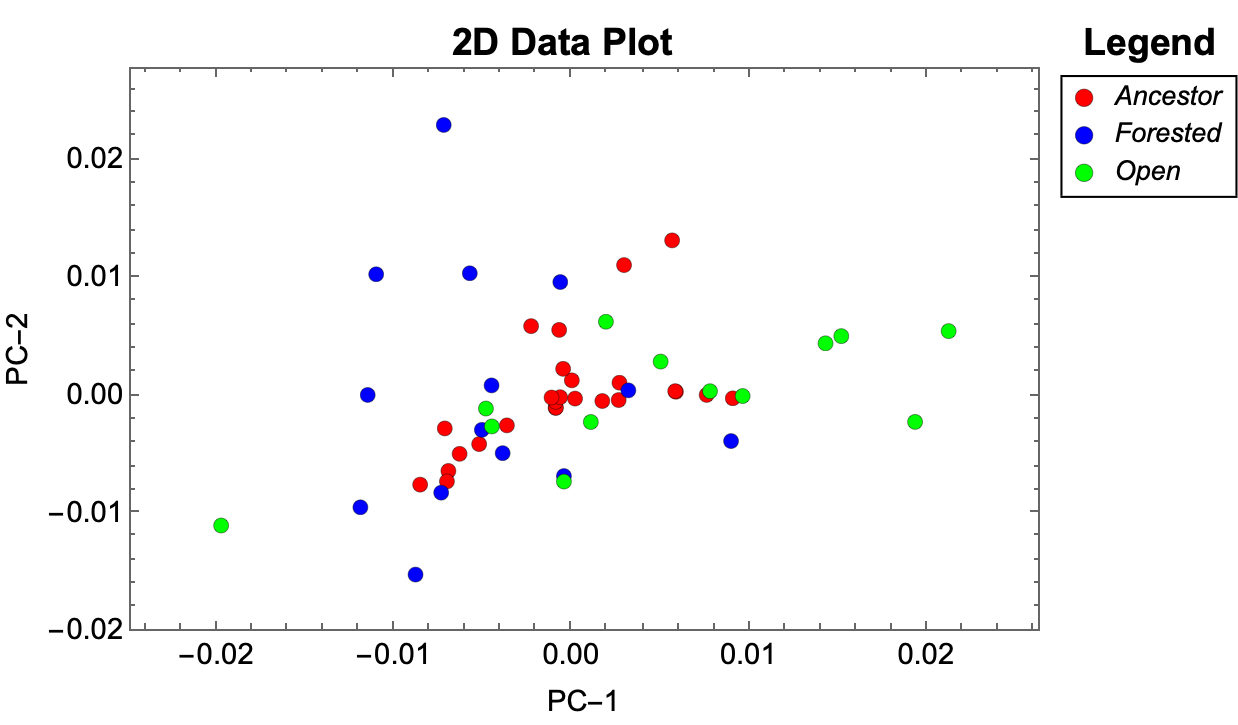

Supplement: Supplementary file 2 — Additional file 2: Datasets and results archive. [file 12862_2022_1978_MOESM2_ESM.zip › Additional Files 2/Datasets & Results Archive/Geometric Morphometrics (Landmarks) Analyses/Phylogenetic Covariation/Forewings Phylogenetic Signal Tests/PC-1 vs PC-2 (Landscape Groups).tif]

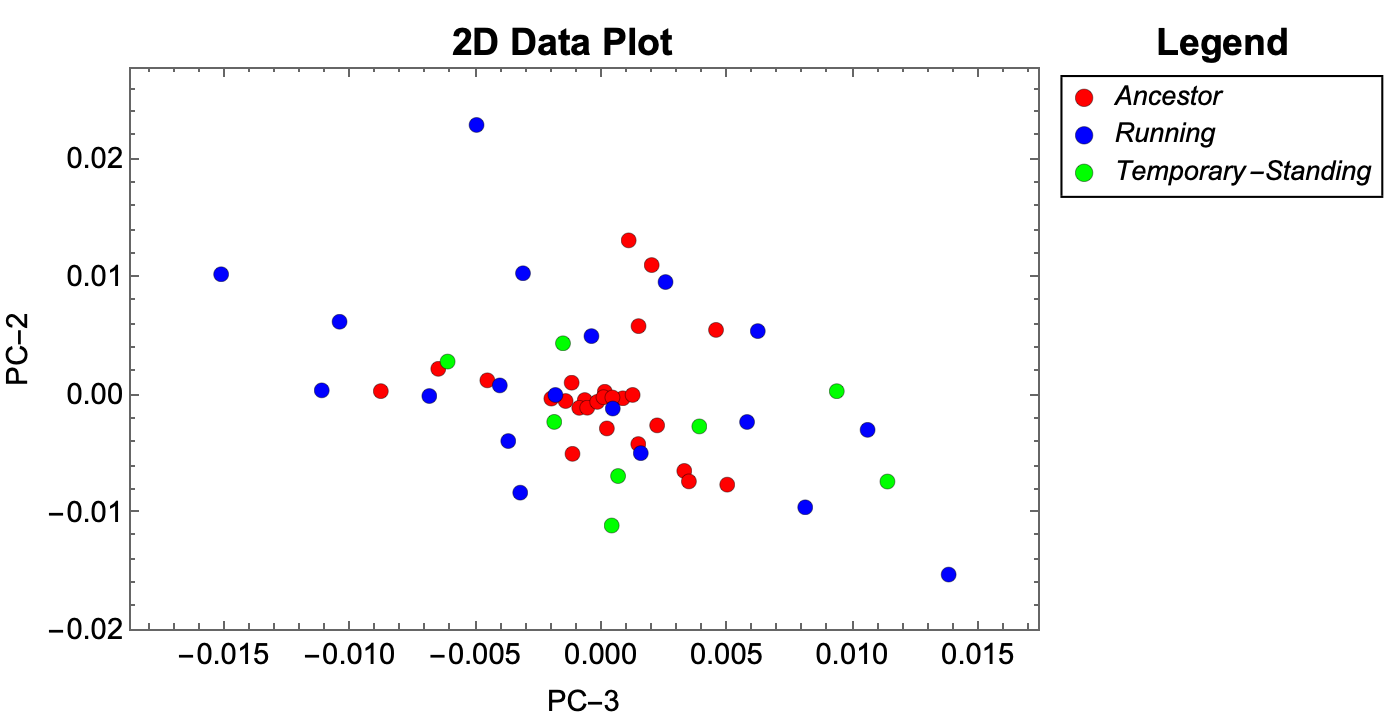

Supplement: Supplementary file 2 — Additional file 2: Datasets and results archive. [file 12862_2022_1978_MOESM2_ESM.zip › Additional Files 2/Datasets & Results Archive/Geometric Morphometrics (Landmarks) Analyses/Phylogenetic Covariation/Forewings Phylogenetic Signal Tests/PC-3 vs PC-2 (Water Body Groups).tif]

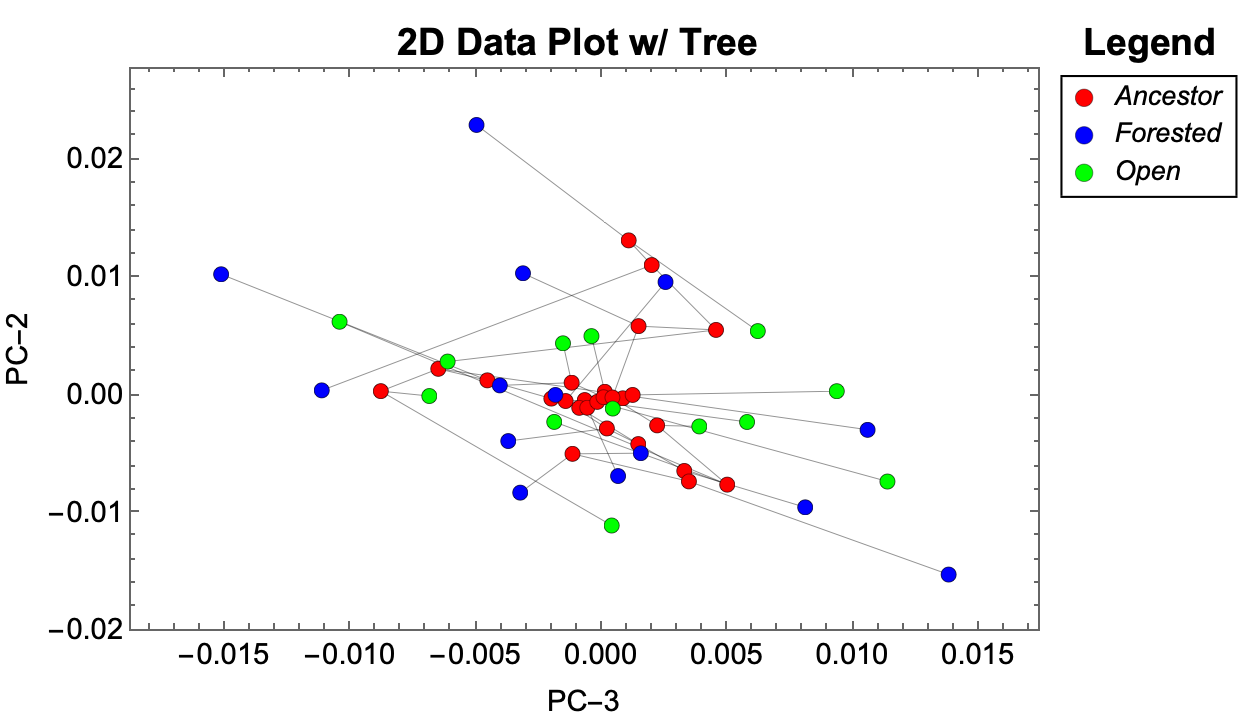

Supplement: Supplementary file 2 — Additional file 2: Datasets and results archive. [file 12862_2022_1978_MOESM2_ESM.zip › Additional Files 2/Datasets & Results Archive/Geometric Morphometrics (Landmarks) Analyses/Phylogenetic Covariation/Forewings Phylogenetic Signal Tests/PC-3 vs PC-2 (Landscape Groups w: Tree).tif]

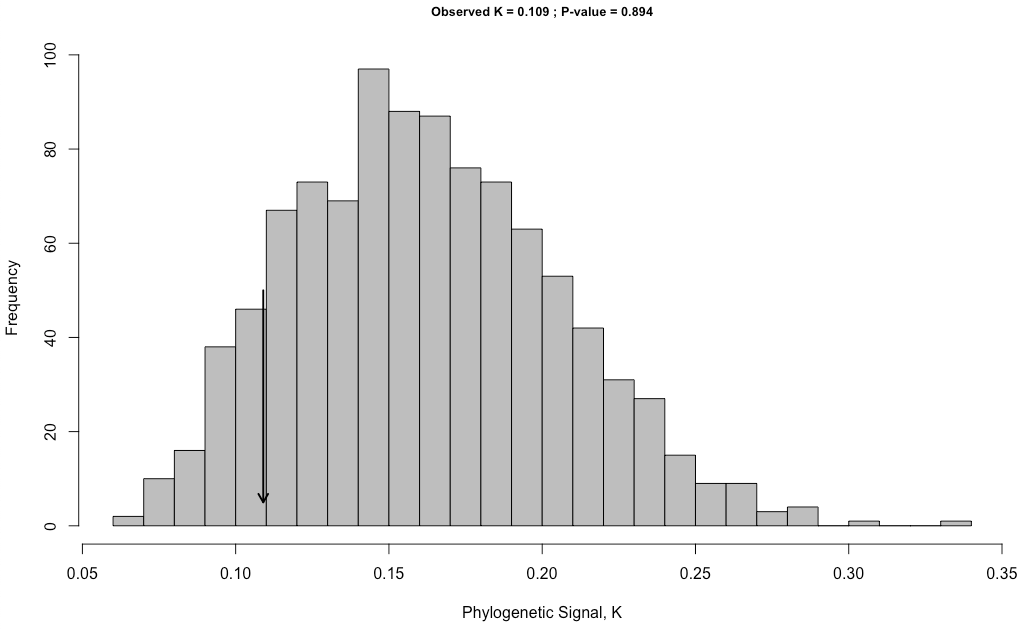

Supplement: Supplementary file 2 — Additional file 2: Datasets and results archive. [file 12862_2022_1978_MOESM2_ESM.zip › Additional Files 2/Datasets & Results Archive/Geometric Morphometrics (Landmarks) Analyses/Phylogenetic Covariation/Forewings Phylogenetic Signal Tests/K-Statistic Plot (BS).tiff]

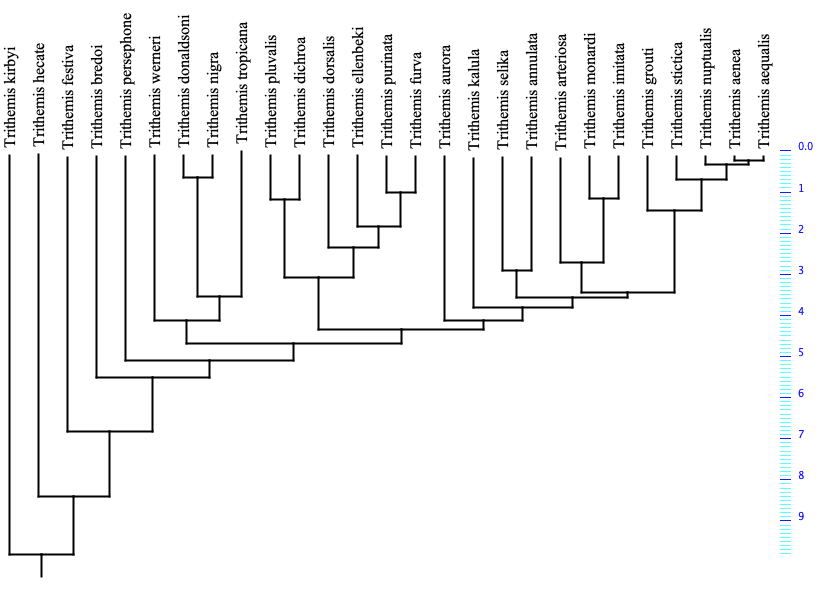

Supplement: Supplementary file 2 — Additional file 2: Datasets and results archive. [file 12862_2022_1978_MOESM2_ESM.zip › Additional Files 2/Datasets & Results Archive/Geometric Morphometrics (Landmarks) Analyses/Phylogenetic Covariation/Tree Files/Trithemis Tree.tiff]

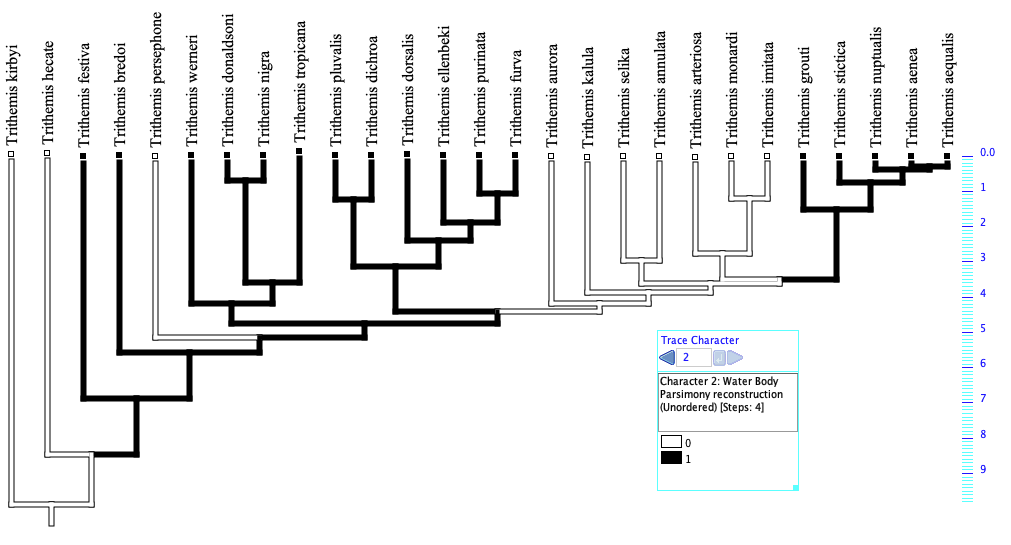

Supplement: Supplementary file 2 — Additional file 2: Datasets and results archive. [file 12862_2022_1978_MOESM2_ESM.zip › Additional Files 2/Datasets & Results Archive/Geometric Morphometrics (Landmarks) Analyses/Phylogenetic Covariation/Tree Files/Trithemis Water Body Map (Parsimony).tiff]

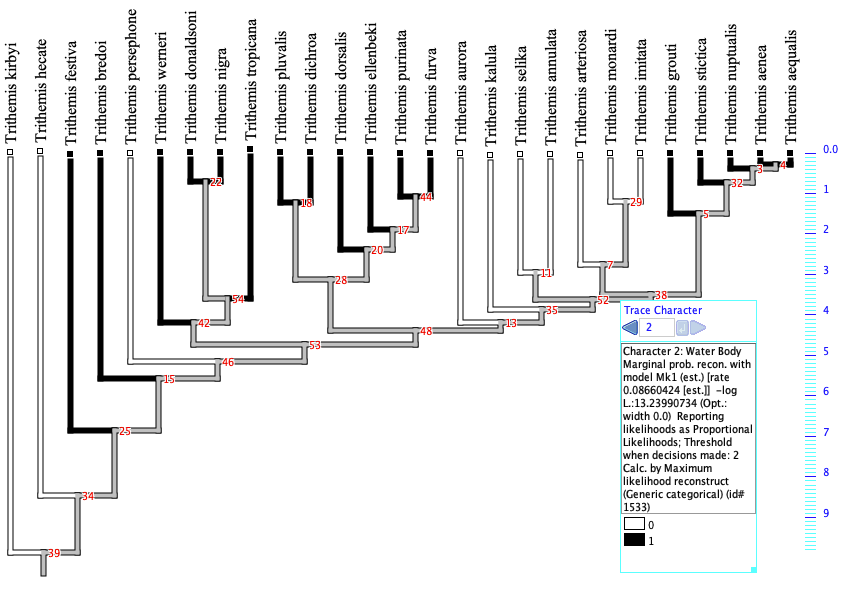

Supplement: Supplementary file 2 — Additional file 2: Datasets and results archive. [file 12862_2022_1978_MOESM2_ESM.zip › Additional Files 2/Datasets & Results Archive/Geometric Morphometrics (Landmarks) Analyses/Phylogenetic Covariation/Tree Files/Trithemis Water Body Map (Likelihood).tiff]

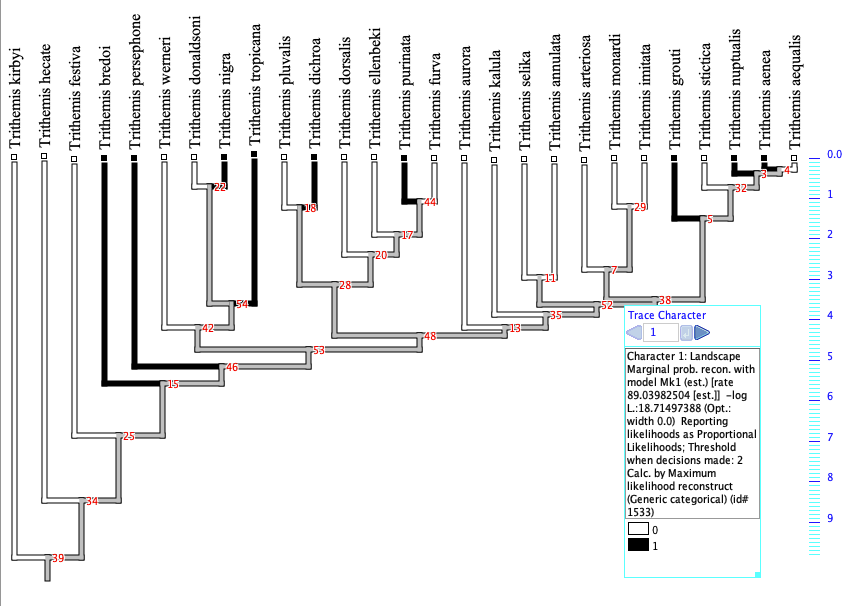

Supplement: Supplementary file 2 — Additional file 2: Datasets and results archive. [file 12862_2022_1978_MOESM2_ESM.zip › Additional Files 2/Datasets & Results Archive/Geometric Morphometrics (Landmarks) Analyses/Phylogenetic Covariation/Tree Files/Trithemis Landscape Map (Likelihood).tiff]

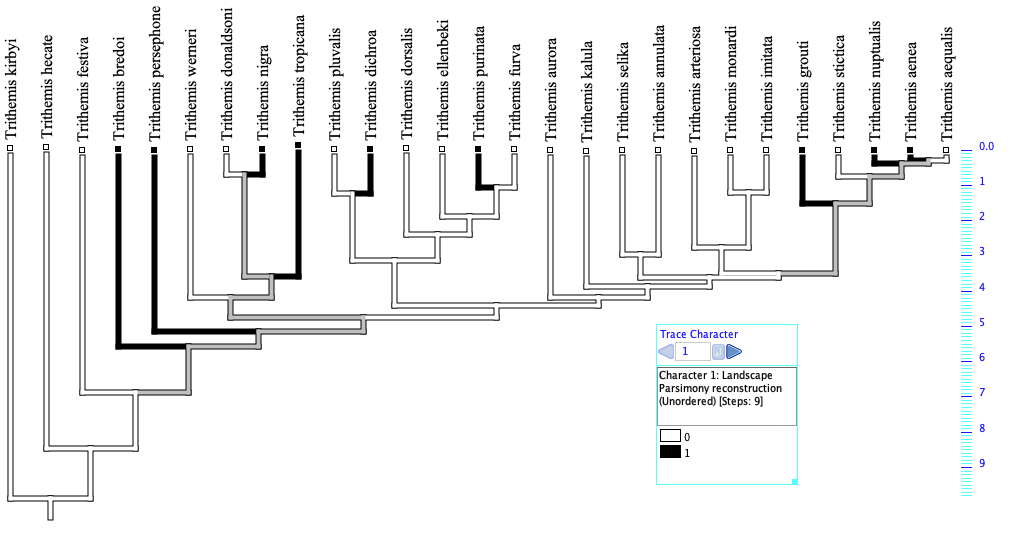

Supplement: Supplementary file 2 — Additional file 2: Datasets and results archive. [file 12862_2022_1978_MOESM2_ESM.zip › Additional Files 2/Datasets & Results Archive/Geometric Morphometrics (Landmarks) Analyses/Phylogenetic Covariation/Tree Files/Trithemis Landscape Map (Parsimony).tiff]

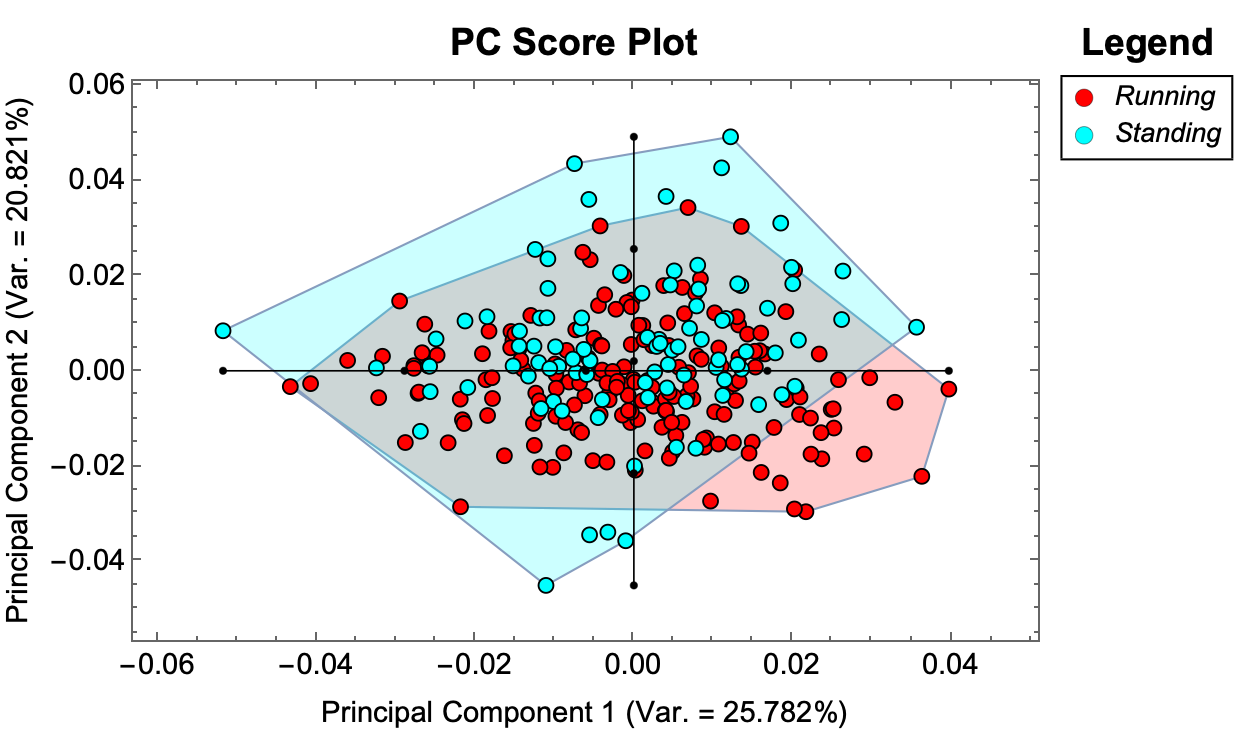

Supplement: Supplementary file 2 — Additional file 2: Datasets and results archive. [file 12862_2022_1978_MOESM2_ESM.zip › Additional Files 2/Datasets & Results Archive/Geometric Morphometrics (Landmarks) Analyses/Forewings/PCA Results/PC-1 vs PC-2 (Water Body Groups w: Model Coords).tif]

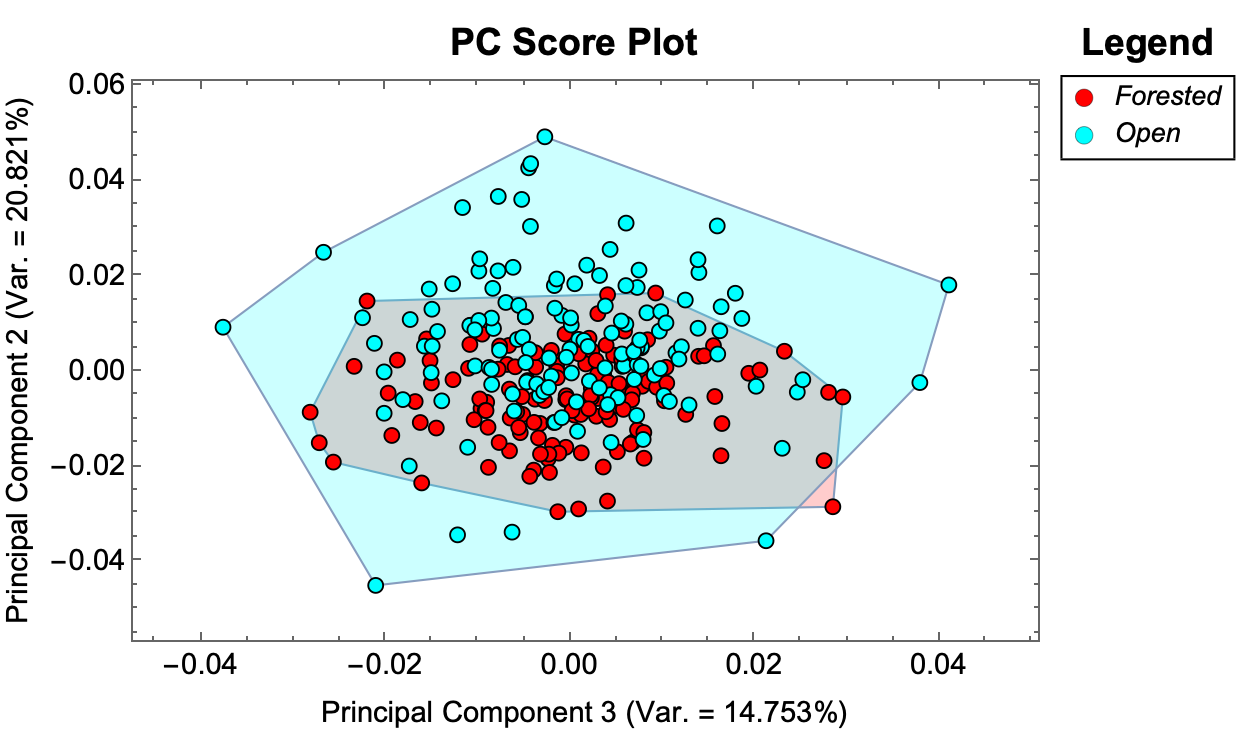

Supplement: Supplementary file 2 — Additional file 2: Datasets and results archive. [file 12862_2022_1978_MOESM2_ESM.zip › Additional Files 2/Datasets & Results Archive/Geometric Morphometrics (Landmarks) Analyses/Forewings/PCA Results/PC-3 vs PC-2 (Landscape Groups).tif]

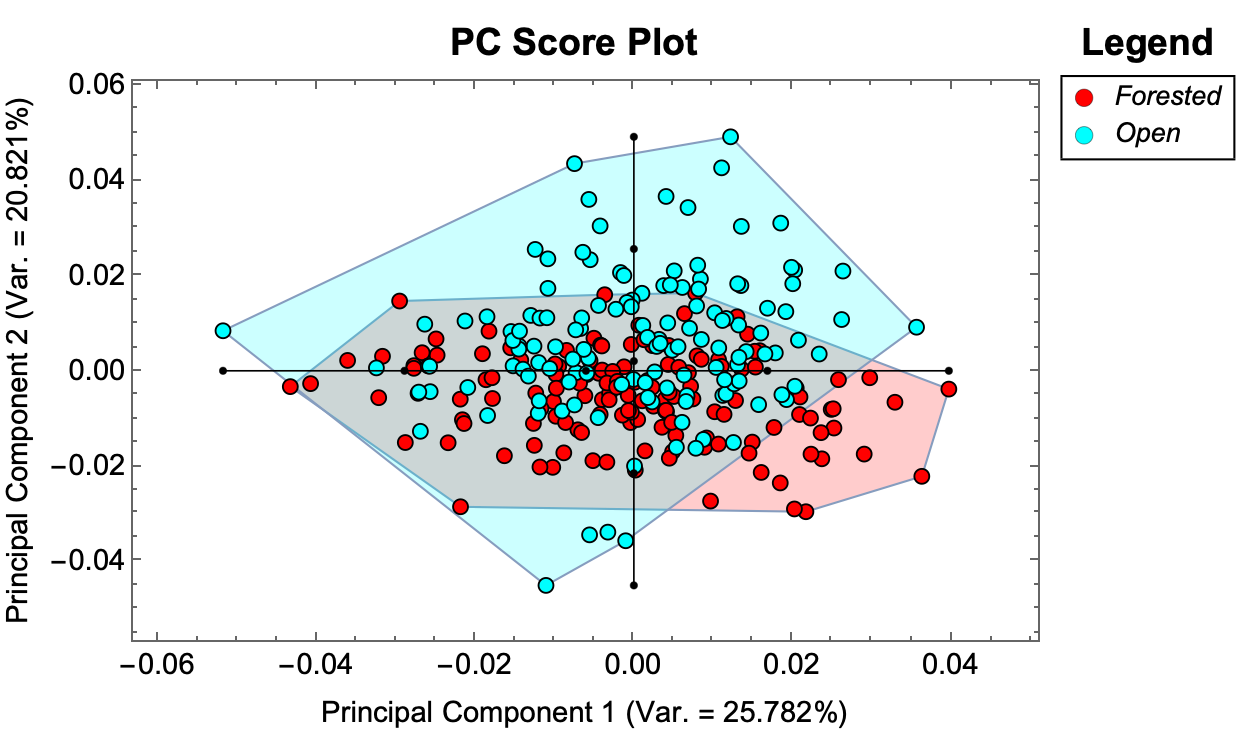

Supplement: Supplementary file 2 — Additional file 2: Datasets and results archive. [file 12862_2022_1978_MOESM2_ESM.zip › Additional Files 2/Datasets & Results Archive/Geometric Morphometrics (Landmarks) Analyses/Forewings/PCA Results/PC-1 vs PC-2 (Landscape Groups w: Model Coords).tif]

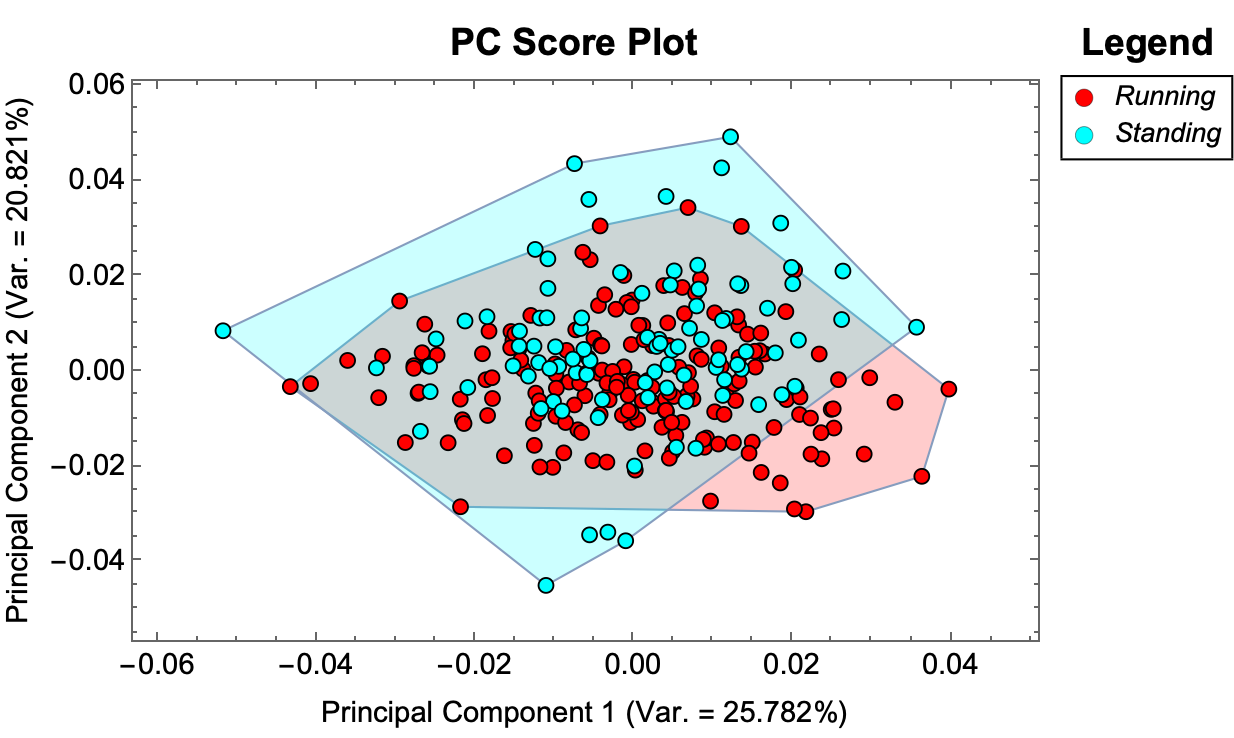

Supplement: Supplementary file 2 — Additional file 2: Datasets and results archive. [file 12862_2022_1978_MOESM2_ESM.zip › Additional Files 2/Datasets & Results Archive/Geometric Morphometrics (Landmarks) Analyses/Forewings/PCA Results/PC-1 vs PC-2 (Water Body Groups).tif]

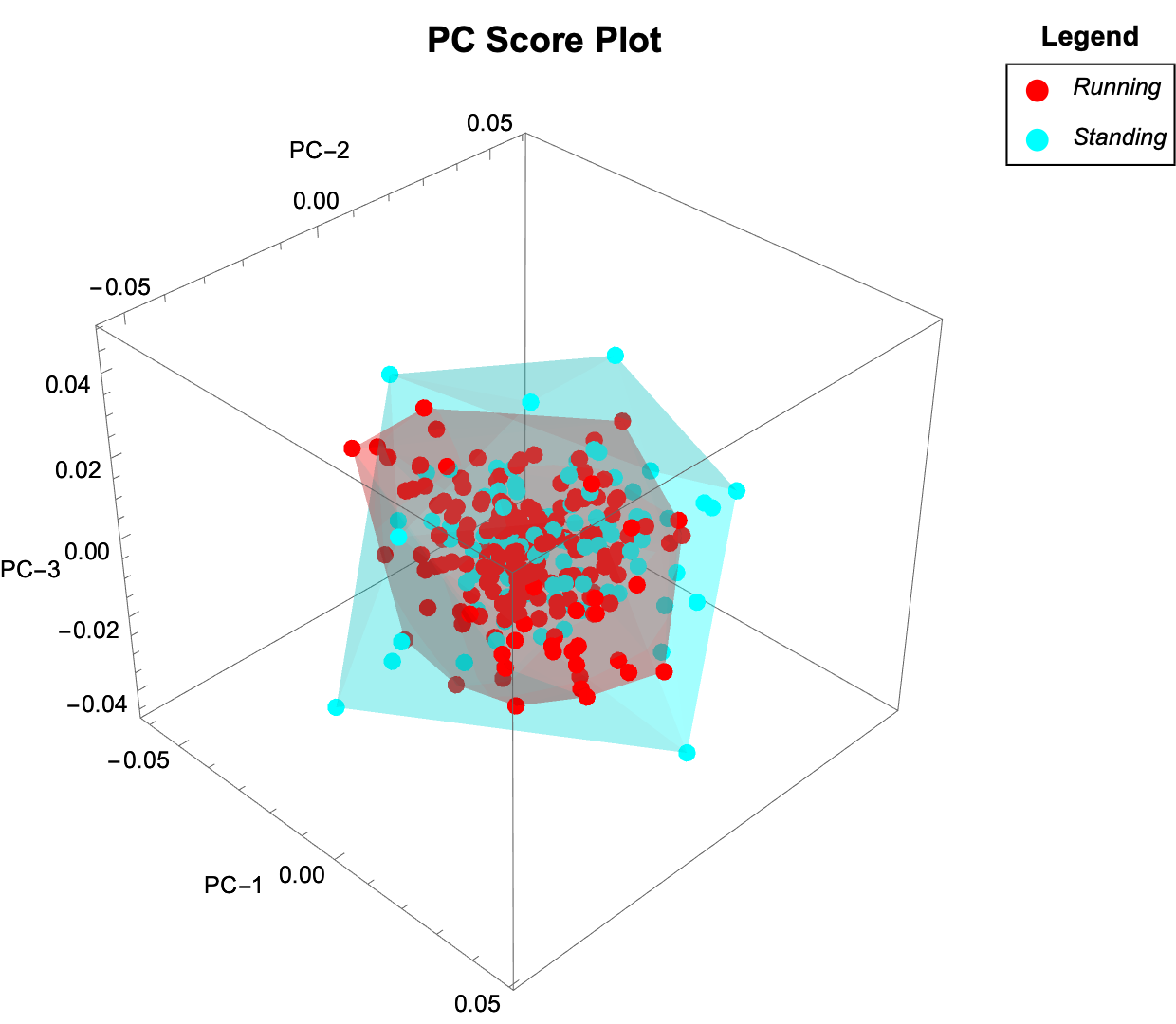

Supplement: Supplementary file 2 — Additional file 2: Datasets and results archive. [file 12862_2022_1978_MOESM2_ESM.zip › Additional Files 2/Datasets & Results Archive/Geometric Morphometrics (Landmarks) Analyses/Forewings/PCA Results/PC-1 vs PC-2 vs PC-3 (Water Body Groups).tif]

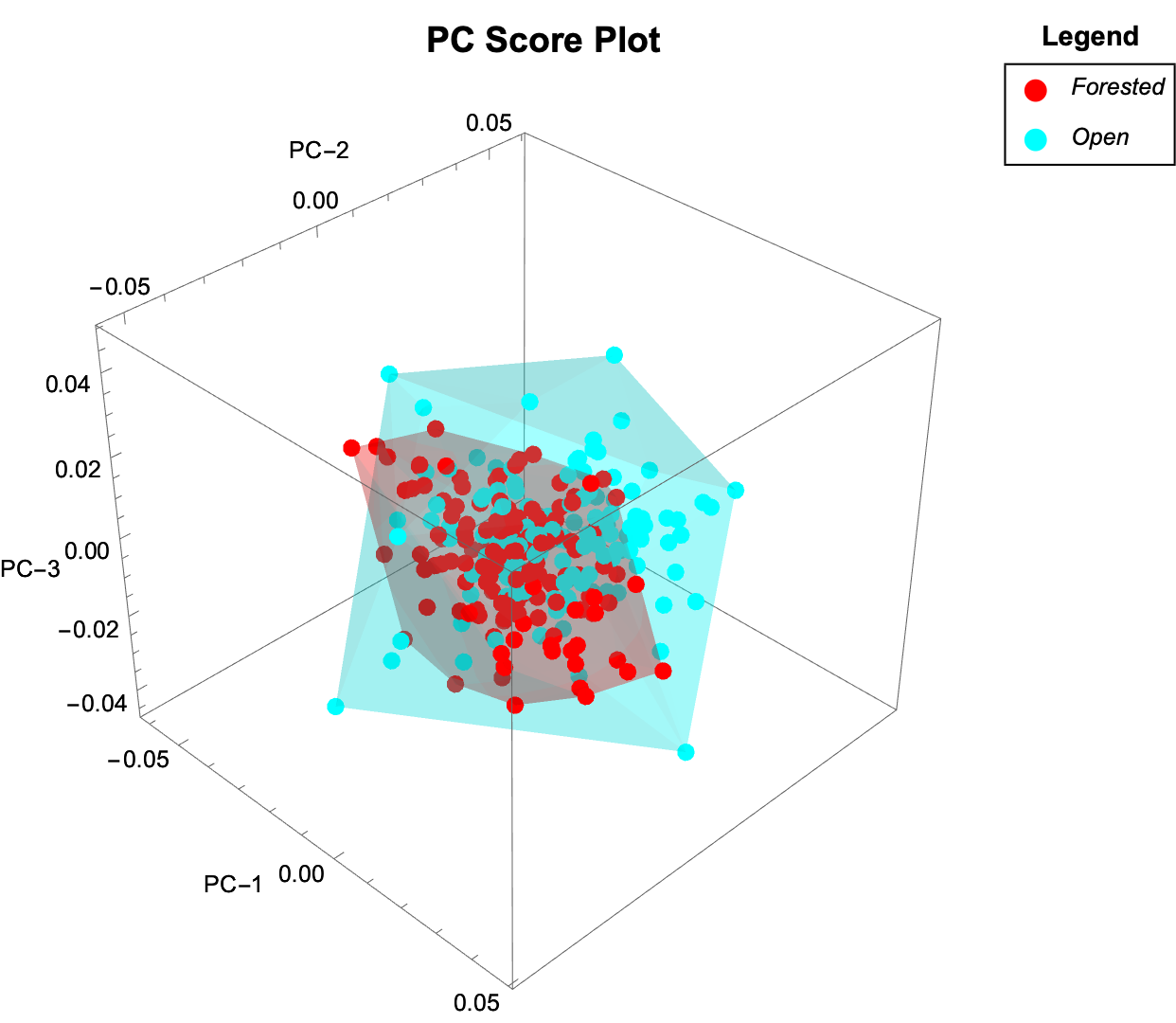

Supplement: Supplementary file 2 — Additional file 2: Datasets and results archive. [file 12862_2022_1978_MOESM2_ESM.zip › Additional Files 2/Datasets & Results Archive/Geometric Morphometrics (Landmarks) Analyses/Forewings/PCA Results/PC-1 vs PC-2 vs PC-3 (Landscape Groups).tif]

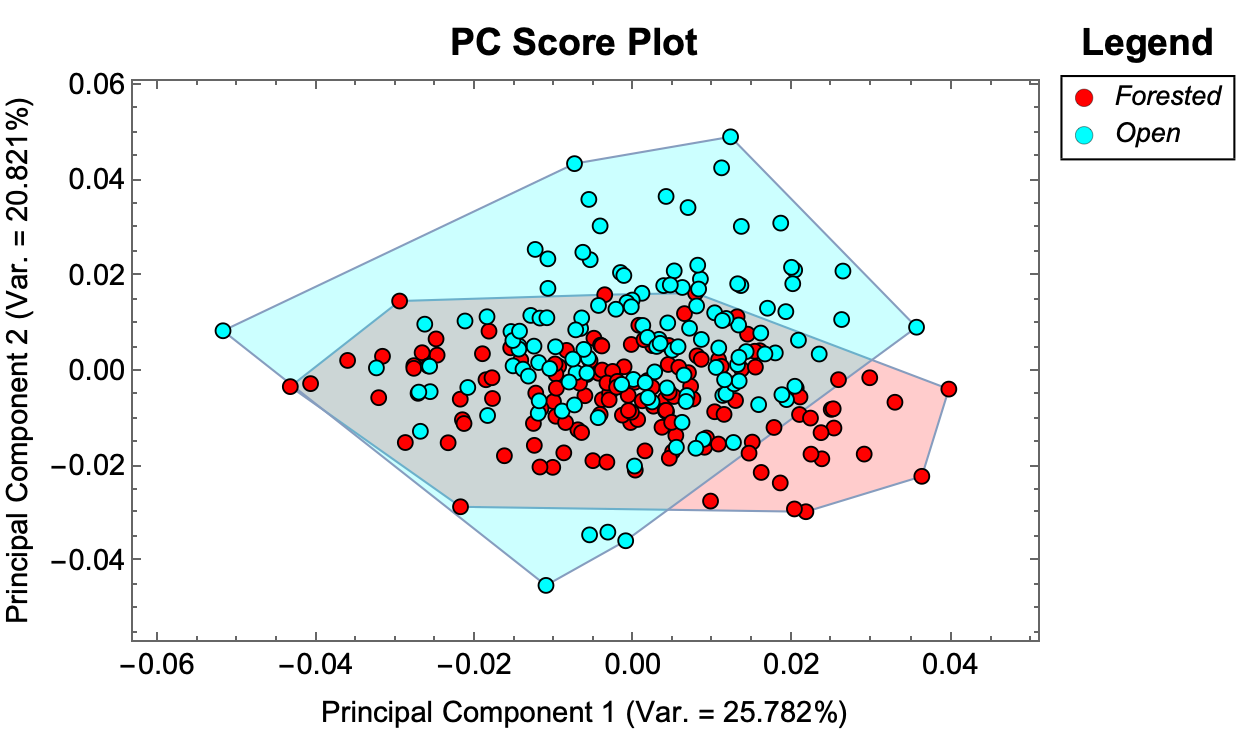

Supplement: Supplementary file 2 — Additional file 2: Datasets and results archive. [file 12862_2022_1978_MOESM2_ESM.zip › Additional Files 2/Datasets & Results Archive/Geometric Morphometrics (Landmarks) Analyses/Forewings/PCA Results/PC-1 vs PC-2 (Landscape Groups).tif]

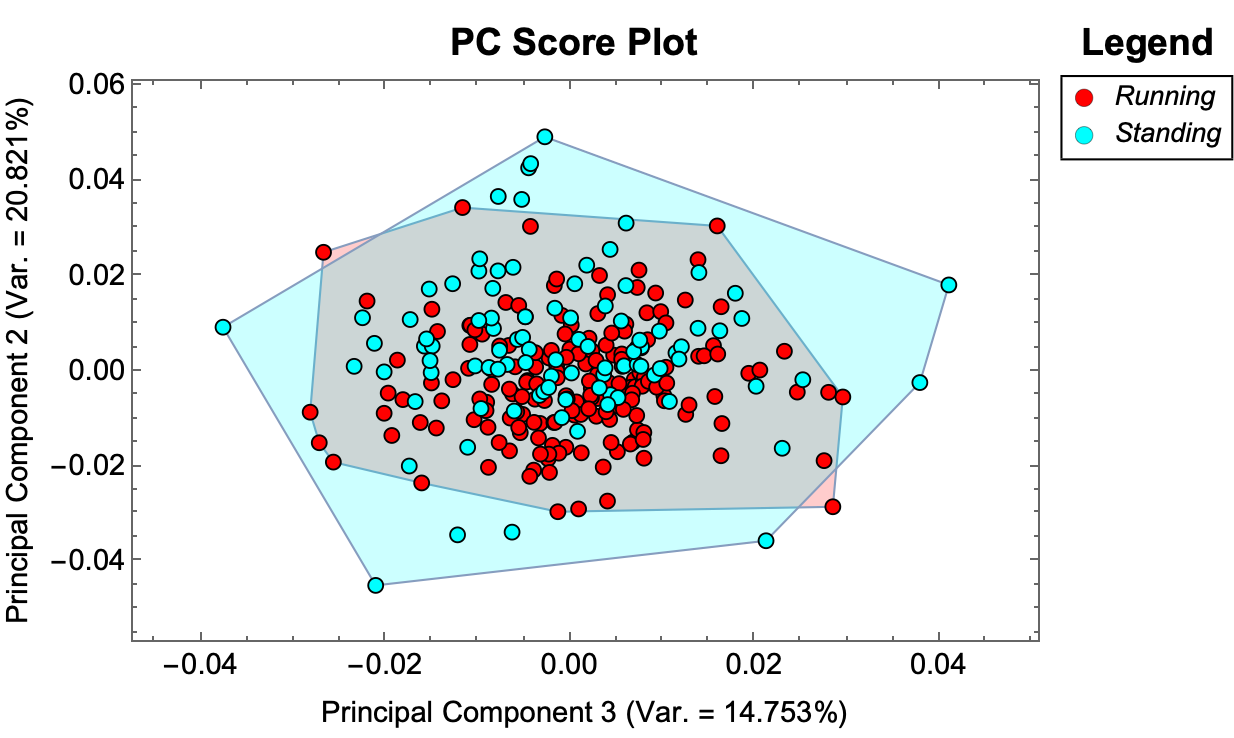

Supplement: Supplementary file 2 — Additional file 2: Datasets and results archive. [file 12862_2022_1978_MOESM2_ESM.zip › Additional Files 2/Datasets & Results Archive/Geometric Morphometrics (Landmarks) Analyses/Forewings/PCA Results/PC-3 vs PC-2 (Water Body Groups).tif]

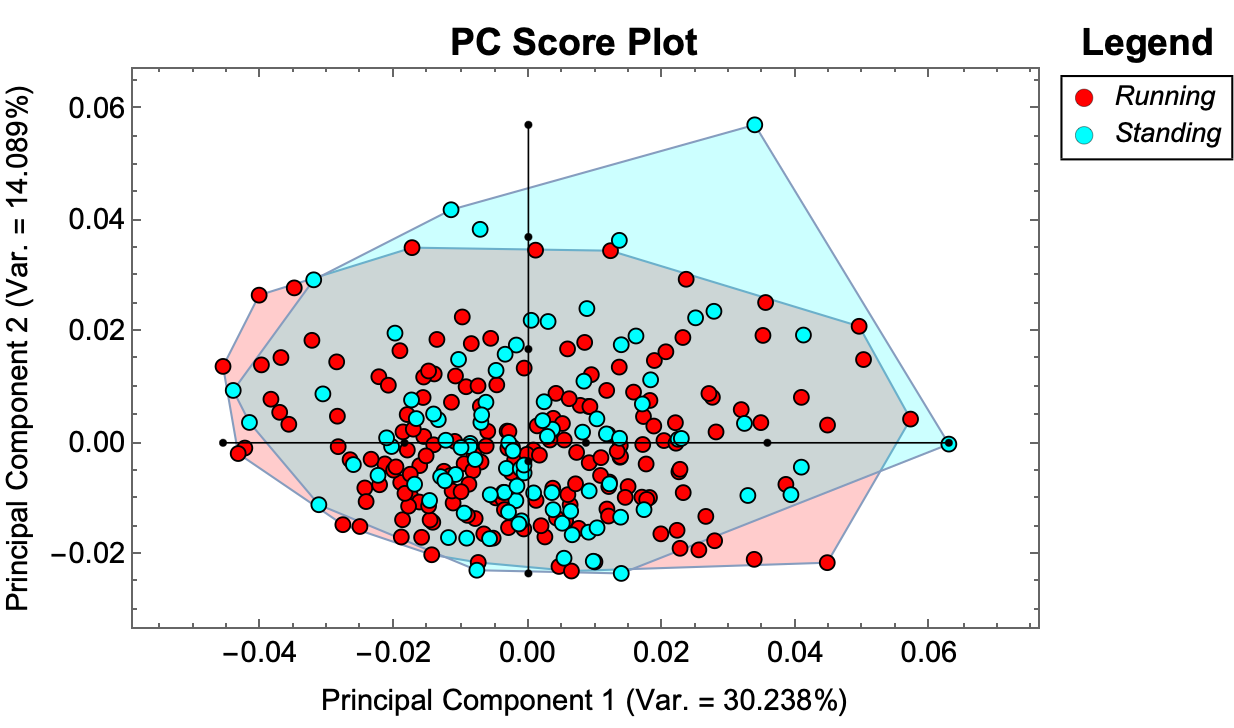

Supplement: Supplementary file 2 — Additional file 2: Datasets and results archive. [file 12862_2022_1978_MOESM2_ESM.zip › Additional Files 2/Datasets & Results Archive/Geometric Morphometrics (Landmarks) Analyses/Hindwings/PCA Results/PC-1 vs PC-2 (Water Body Groups w: Model Coords).tif]

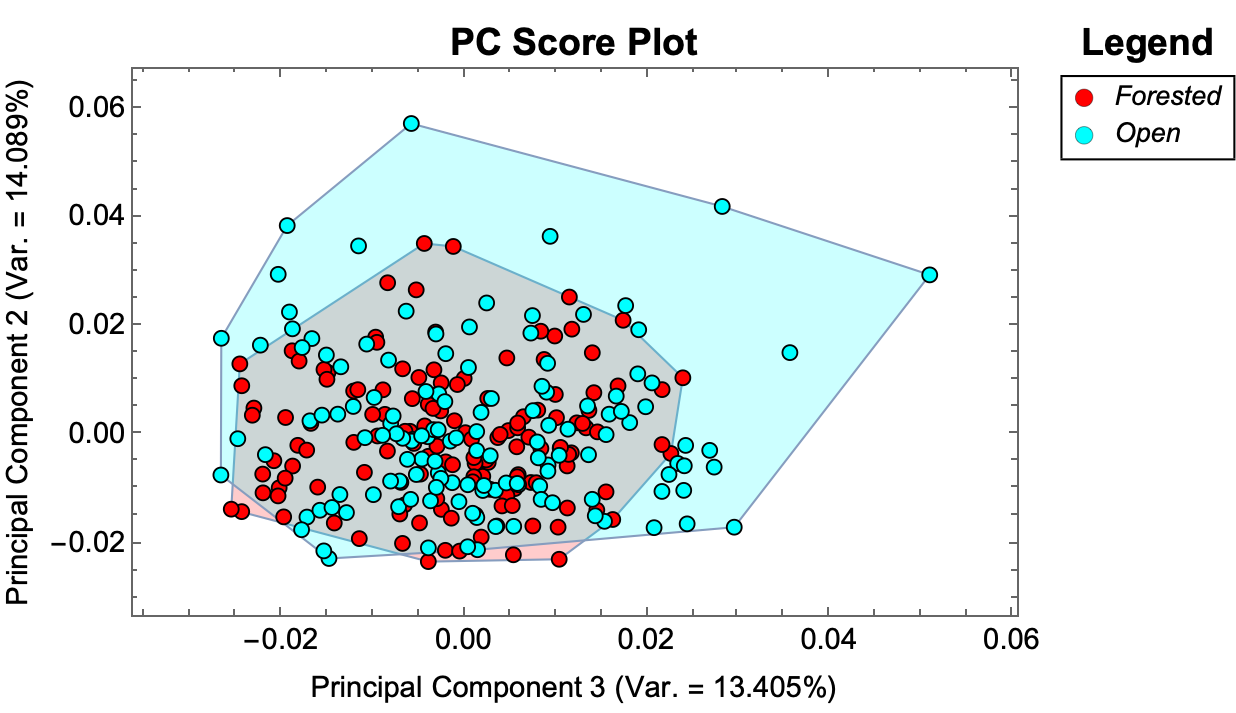

Supplement: Supplementary file 2 — Additional file 2: Datasets and results archive. [file 12862_2022_1978_MOESM2_ESM.zip › Additional Files 2/Datasets & Results Archive/Geometric Morphometrics (Landmarks) Analyses/Hindwings/PCA Results/PC-3 vs PC-2 (Landscape Groups).tif]

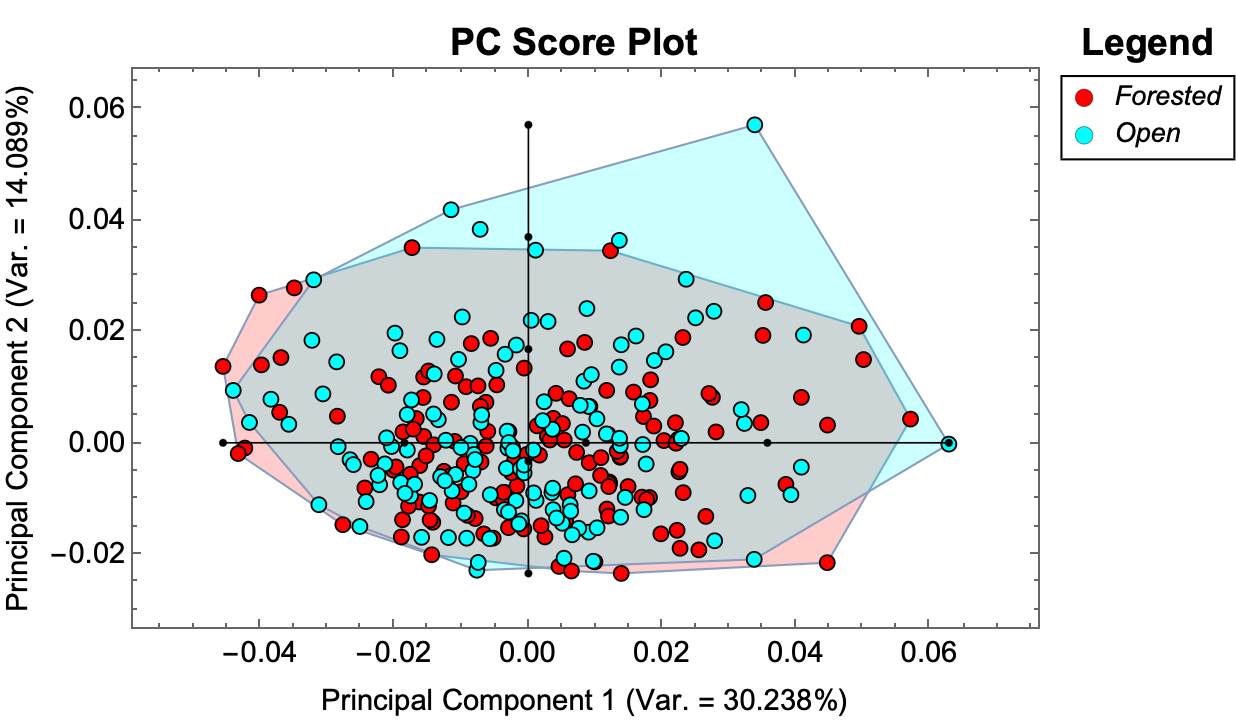

Supplement: Supplementary file 2 — Additional file 2: Datasets and results archive. [file 12862_2022_1978_MOESM2_ESM.zip › Additional Files 2/Datasets & Results Archive/Geometric Morphometrics (Landmarks) Analyses/Hindwings/PCA Results/PC-1 vs PC-2 (Landscape Groups w: Model Coords).tif]

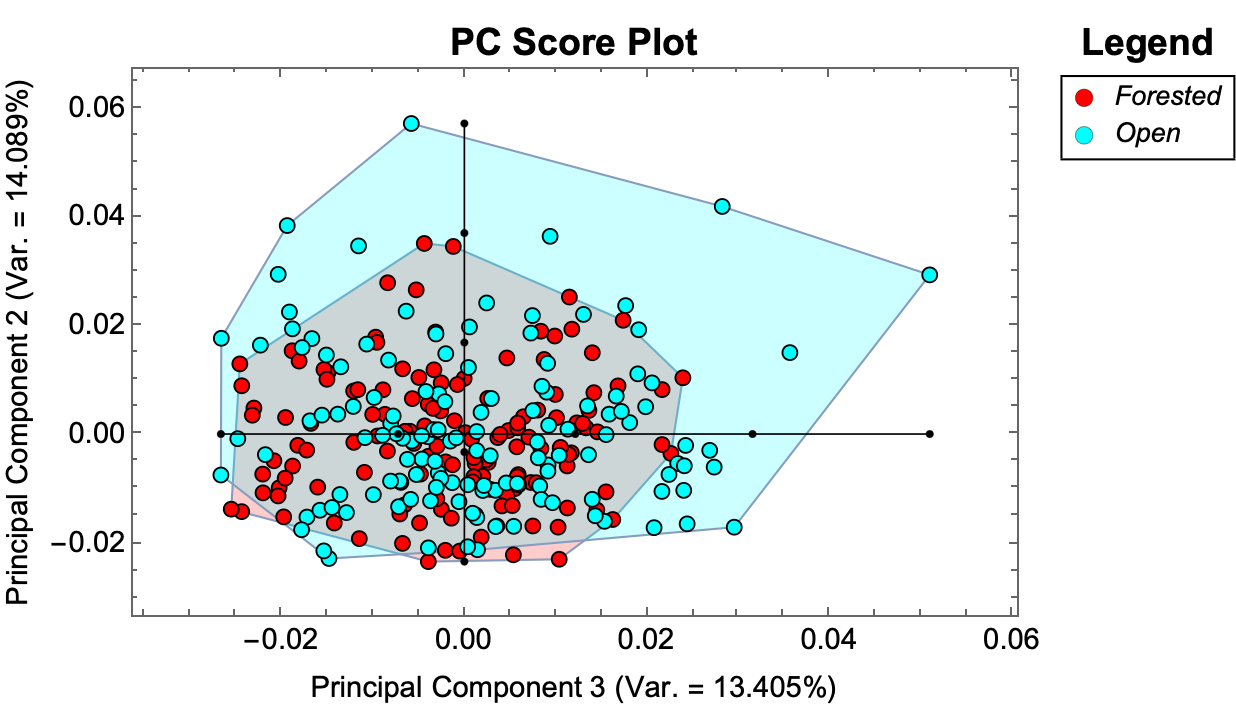

Supplement: Supplementary file 2 — Additional file 2: Datasets and results archive. [file 12862_2022_1978_MOESM2_ESM.zip › Additional Files 2/Datasets & Results Archive/Geometric Morphometrics (Landmarks) Analyses/Hindwings/PCA Results/PC-3 vs PC-2 (Landscape Groups w: Model Coords).tif]

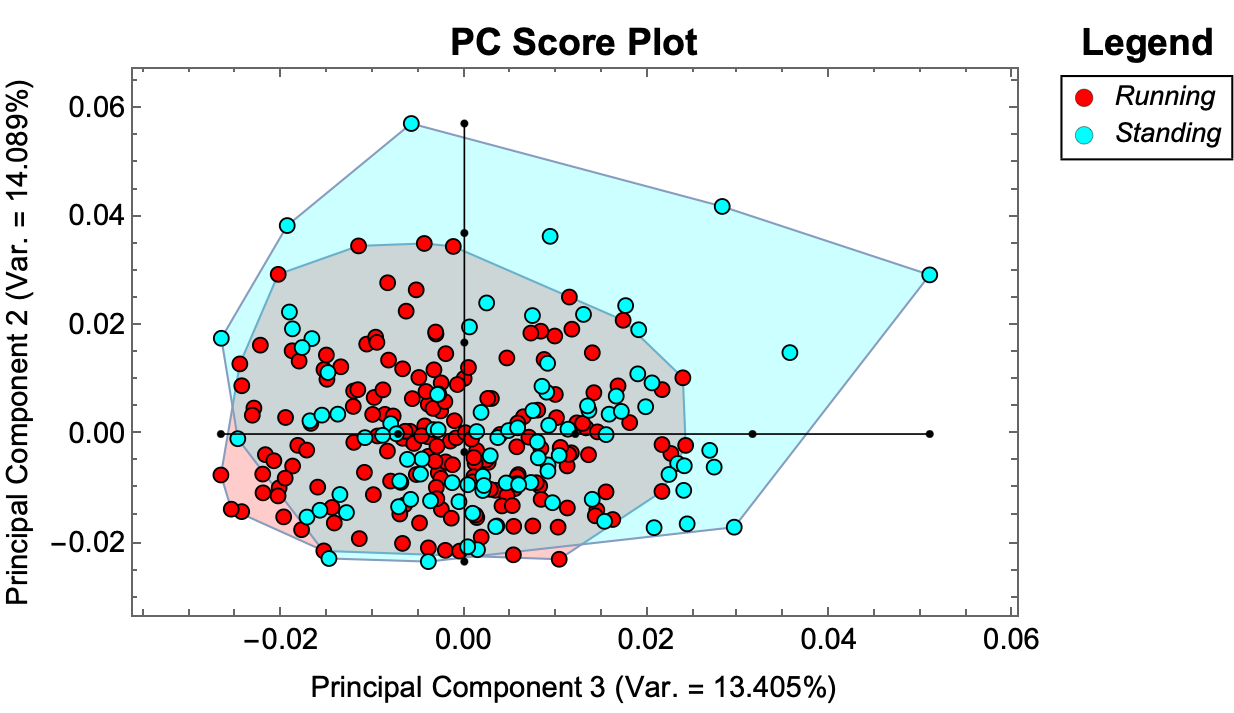

Supplement: Supplementary file 2 — Additional file 2: Datasets and results archive. [file 12862_2022_1978_MOESM2_ESM.zip › Additional Files 2/Datasets & Results Archive/Geometric Morphometrics (Landmarks) Analyses/Hindwings/PCA Results/PC-3 vs PC-2 (Water Body Groups w: Model Coords).tif]

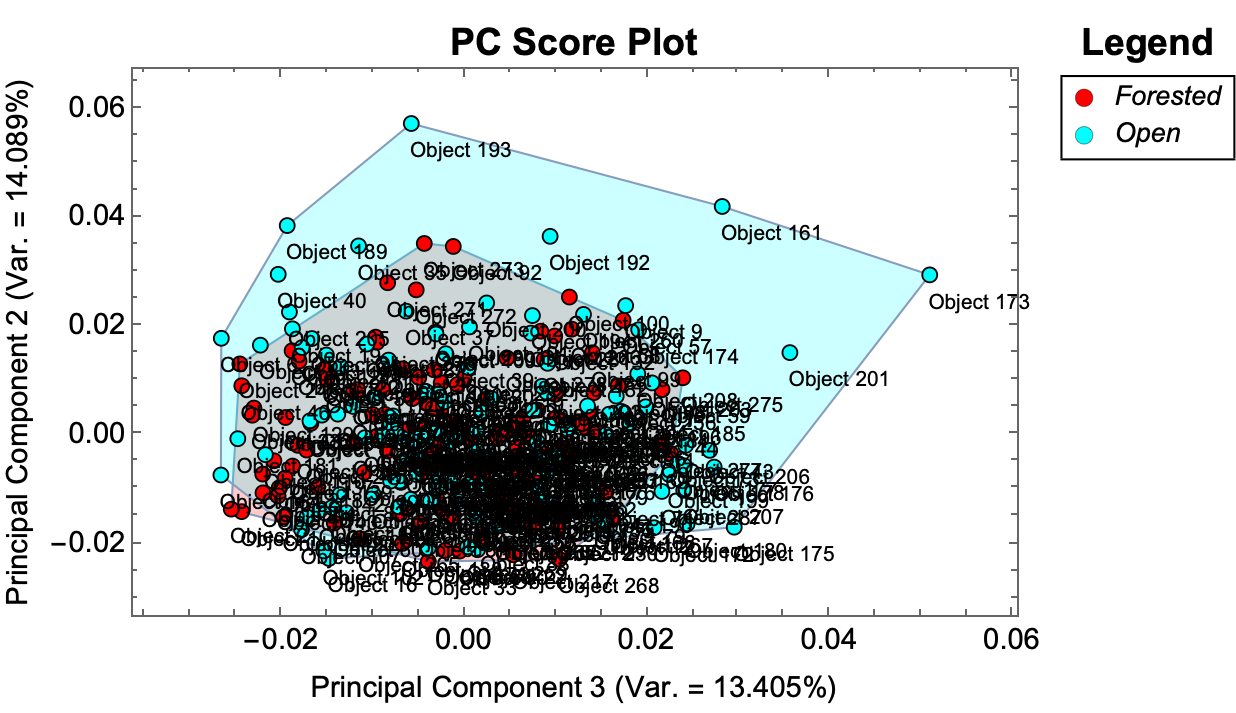

Supplement: Supplementary file 2 — Additional file 2: Datasets and results archive. [file 12862_2022_1978_MOESM2_ESM.zip › Additional Files 2/Datasets & Results Archive/Geometric Morphometrics (Landmarks) Analyses/Hindwings/PCA Results/PC-3 vs PC-2 (Landscape Groups w: Labels).tif]

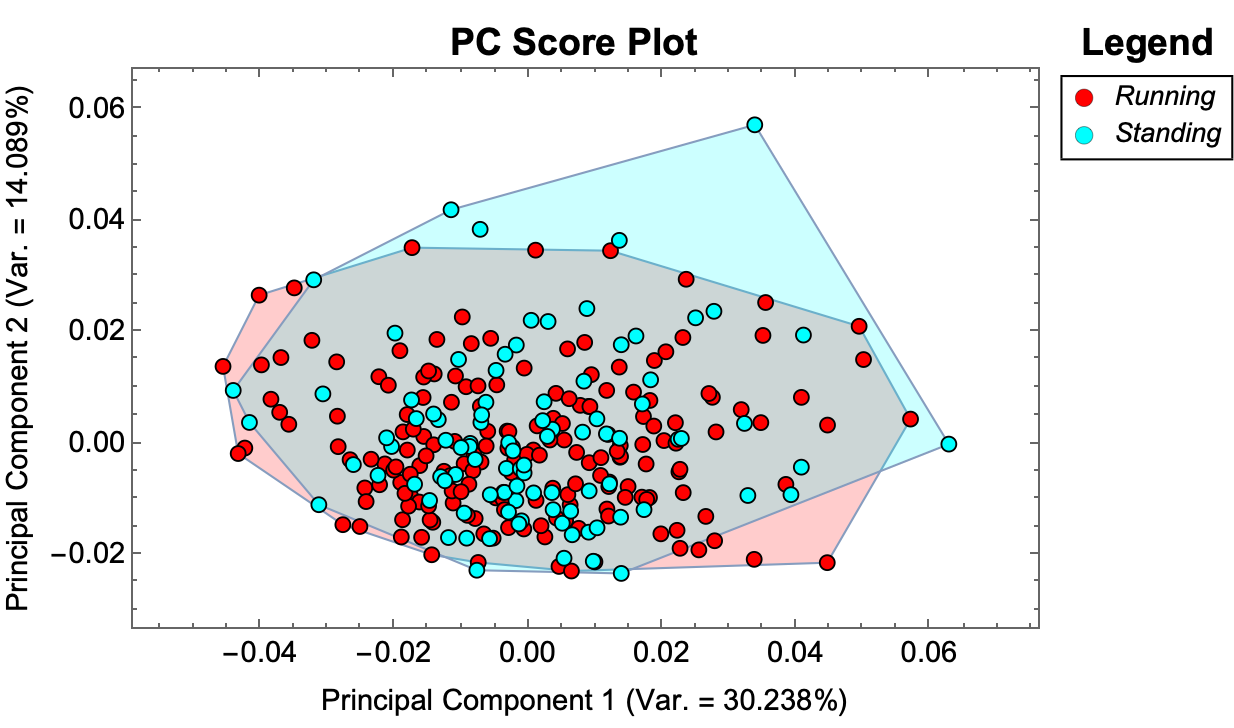

Supplement: Supplementary file 2 — Additional file 2: Datasets and results archive. [file 12862_2022_1978_MOESM2_ESM.zip › Additional Files 2/Datasets & Results Archive/Geometric Morphometrics (Landmarks) Analyses/Hindwings/PCA Results/PC-1 vs PC-2 (Water Body Groups).tif]

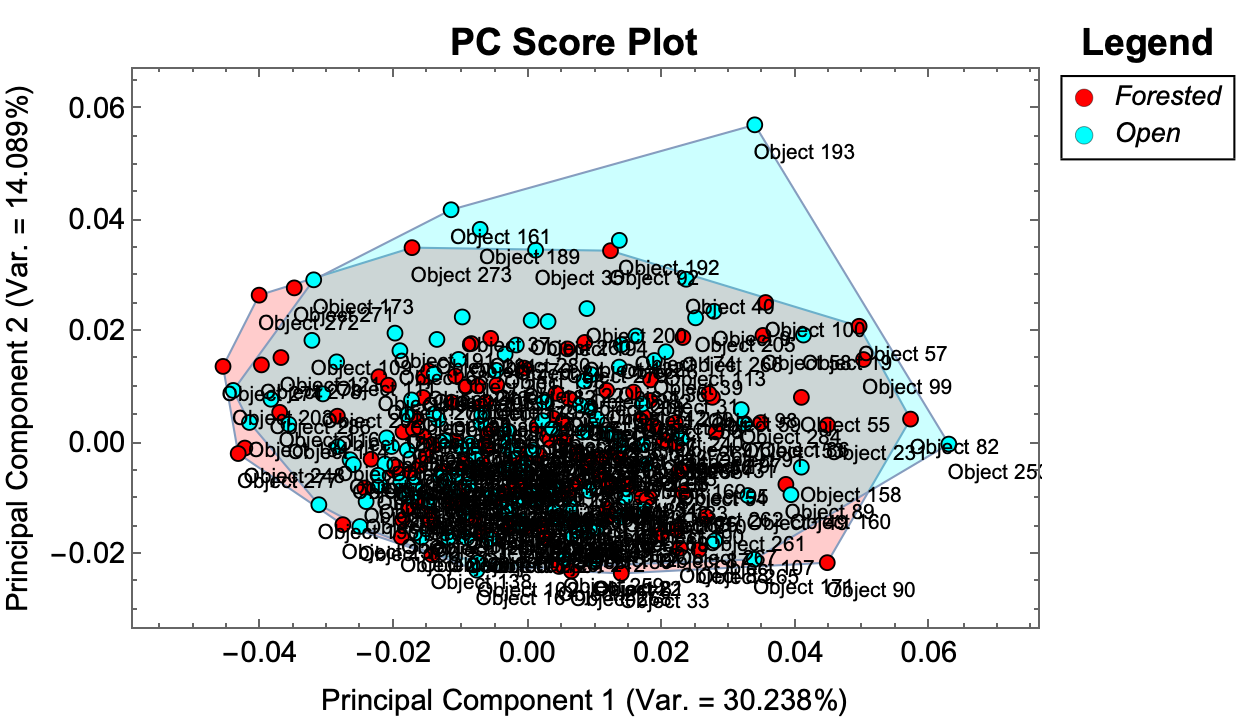

Supplement: Supplementary file 2 — Additional file 2: Datasets and results archive. [file 12862_2022_1978_MOESM2_ESM.zip › Additional Files 2/Datasets & Results Archive/Geometric Morphometrics (Landmarks) Analyses/Hindwings/PCA Results/PC-1 vs PC-2 (Landscape Groups w: Labels).tif]

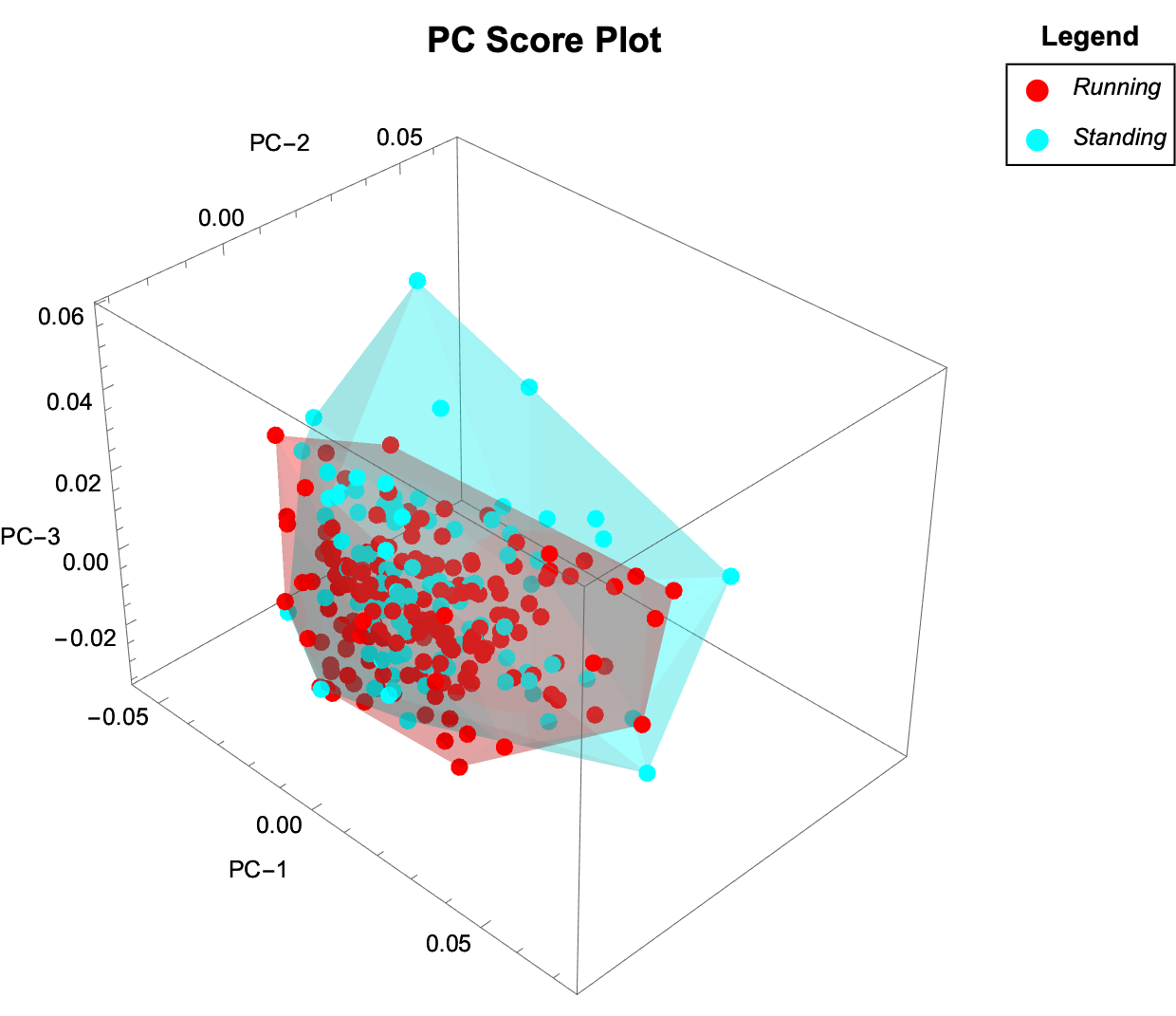

Supplement: Supplementary file 2 — Additional file 2: Datasets and results archive. [file 12862_2022_1978_MOESM2_ESM.zip › Additional Files 2/Datasets & Results Archive/Geometric Morphometrics (Landmarks) Analyses/Hindwings/PCA Results/PC-1 vs PC-2 vs PC-3 (Water Body Groups).tif]

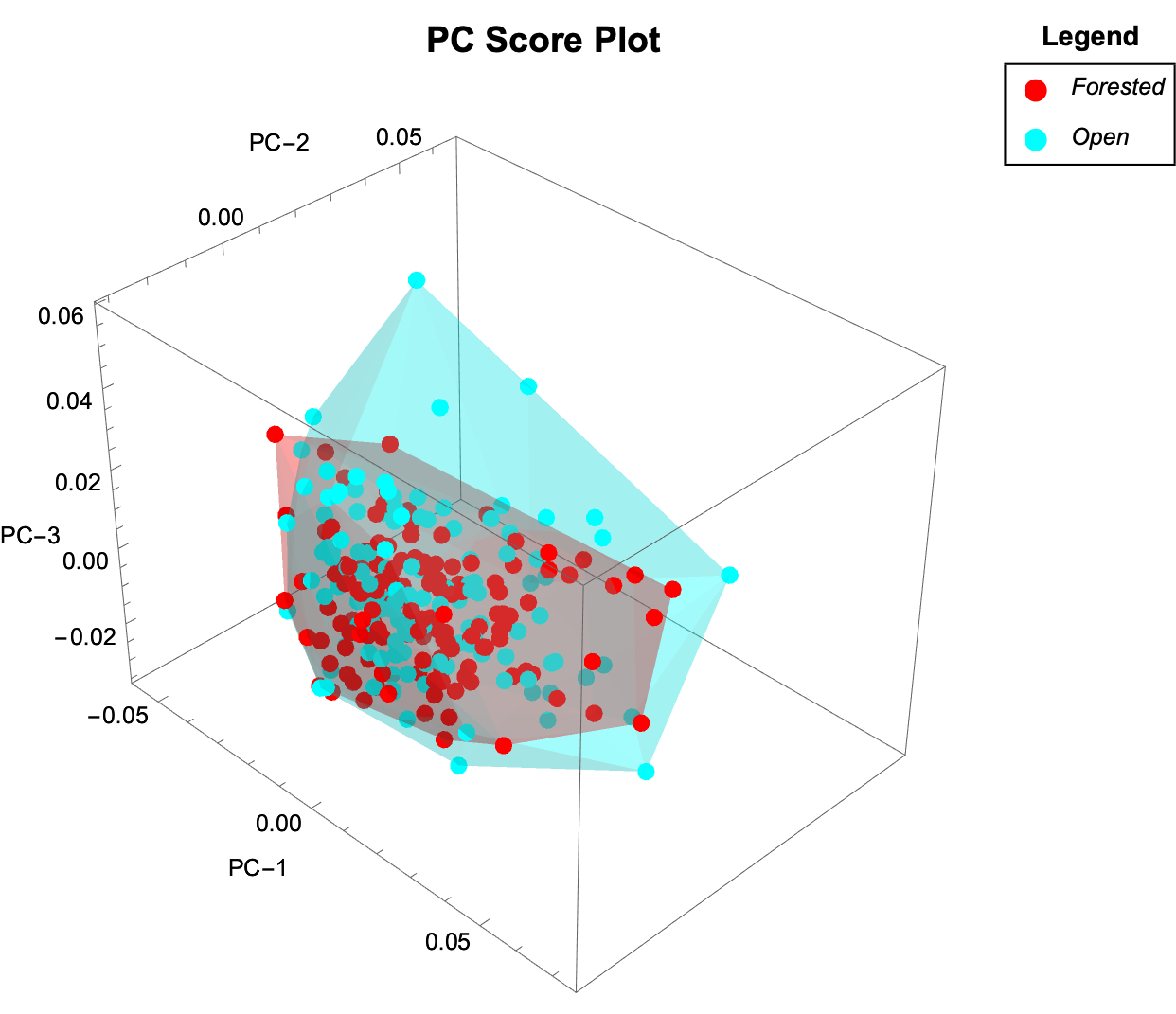

Supplement: Supplementary file 2 — Additional file 2: Datasets and results archive. [file 12862_2022_1978_MOESM2_ESM.zip › Additional Files 2/Datasets & Results Archive/Geometric Morphometrics (Landmarks) Analyses/Hindwings/PCA Results/PC-1 vs PC-2 vs PC-3 (Landscape Groups).tif]

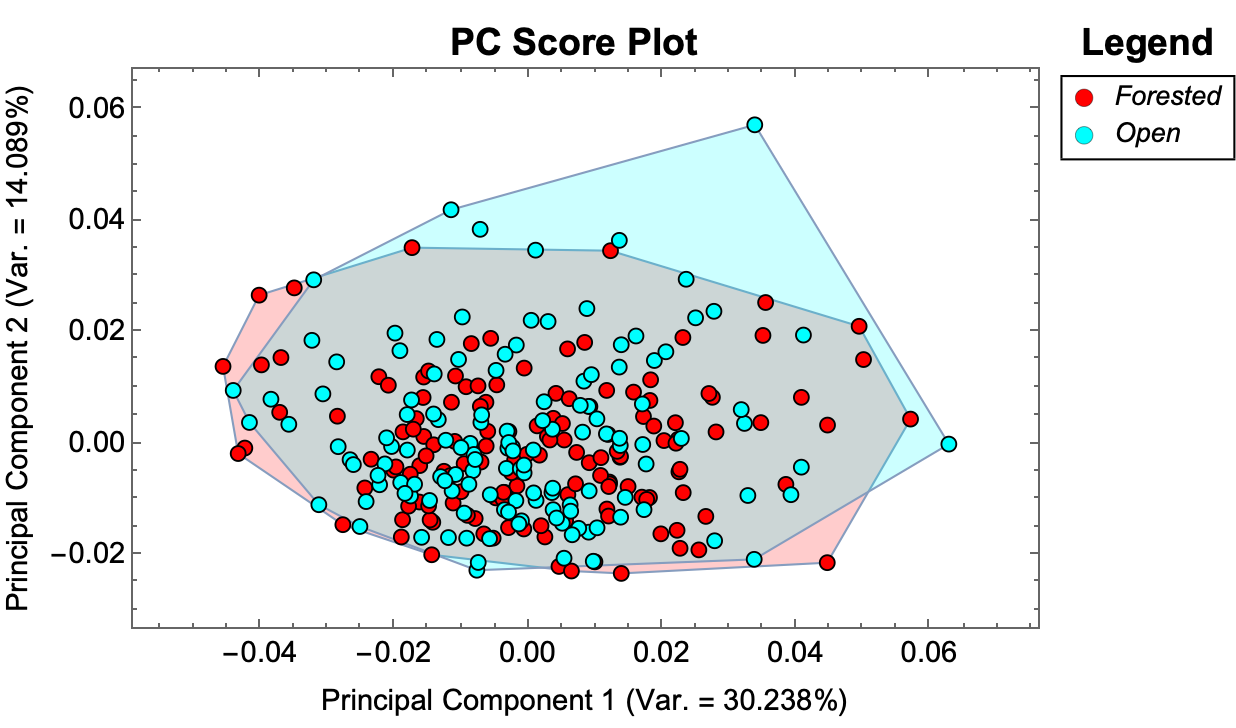

Supplement: Supplementary file 2 — Additional file 2: Datasets and results archive. [file 12862_2022_1978_MOESM2_ESM.zip › Additional Files 2/Datasets & Results Archive/Geometric Morphometrics (Landmarks) Analyses/Hindwings/PCA Results/PC-1 vs PC-2 (Landscape Groups).tif]

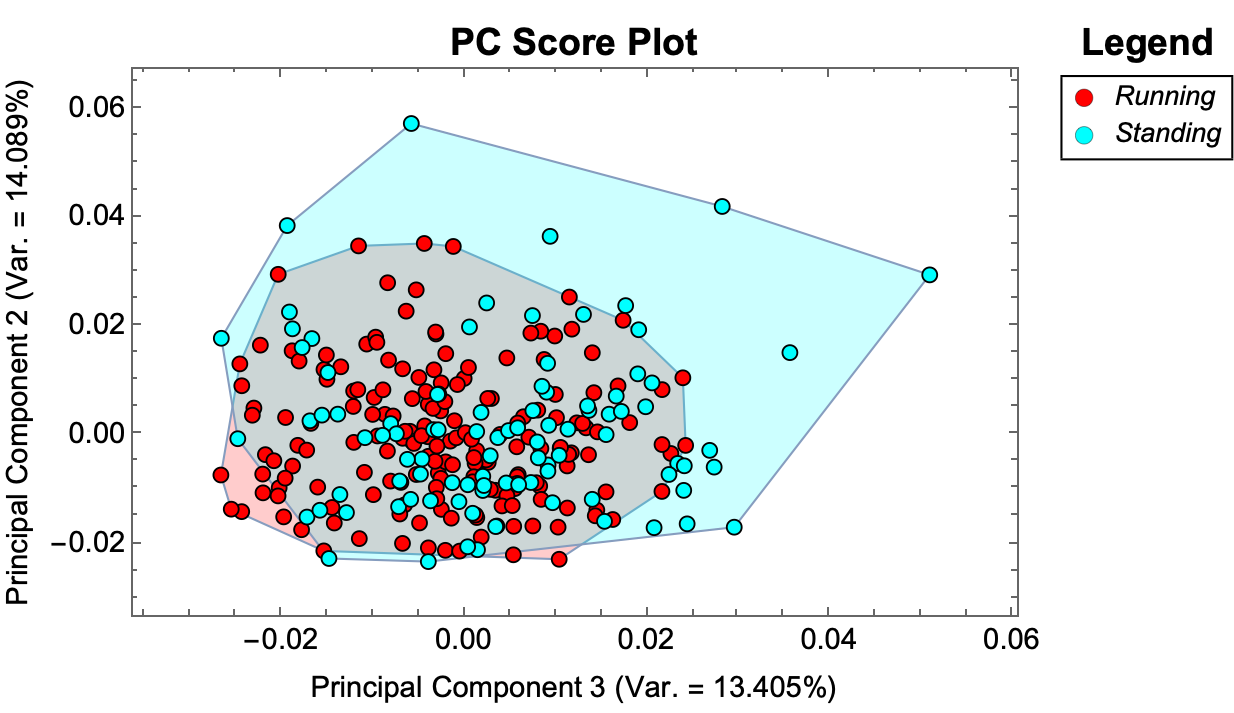

Supplement: Supplementary file 2 — Additional file 2: Datasets and results archive. [file 12862_2022_1978_MOESM2_ESM.zip › Additional Files 2/Datasets & Results Archive/Geometric Morphometrics (Landmarks) Analyses/Hindwings/PCA Results/PC-3 vs PC-2 (Water Body Groups).tif]
