# Supplementary material for: What you sample is what you get: ecomorphological variation in Trithemis (Odonata, Libellulidae) dragonfly wings reconsidered
Source: BMC Ecol Evol. 2022 Apr 11;22:43. doi: 10.1186/s12862-022-01978-y (PMC8996507; doi:10.1186/s12862-022-01978-y)
Supplement: Supplementary file 3 — Additional file 3: Software Archive. [file 12862_2022_1978_MOESM3_ESM.zip › Additional Files 3/Software Archive/Image Classification - Embedded LeNet (vers. 1.0).pdf]

Image Classification - LeNet (Embedded)

This code implements the LeNet CNN-5 architecture for the analysis of image training sets in an embedded learning mode.

Author : N. MacLeod
Date : 28 January 2020
Version : 1.0
Reference : MacLeod & Kolska Horwitz (2020)

Initialize libraries.

```
In[•]:= << ComputationalGeometry`
```

Read - in training-set images.

```
In[•]:= filenamein = SystemDialogInput["FileOpen"];
sourceDirectory = DirectoryName[filenamein]
SetDirectory[sourceDirectory];
dataFileList = Sort[Map[ToString, FileNames["*.tif"]]];
{kgTrain} = Dimensions[dataFileList];

x1 = Table[" ", {kgTrain}];
Do[xName = StringJoin[sourceDirectory, dataFileList[[i]]];
 x1[[i]] = Import[xName, "TIFF"], {i, kgTrain}]
objNamesTrain = dataFileList;
Do[objNamesTrain[[i]] = StringDrop[objNamesTrain[[i]], -4], {i, kgTrain}]
Print["No. of objects: " , kgTrain]
```

```
Out[•]= /Users/n.macleod/Desktop/Dragonflies (Final)/Data &
   Results/Images (ML)/Hindwings/Images-Reduced (w: Numbers)/

No. of objects: 230
```

Display original images (optional).

Select user - specified plot options.

In[•]:=
```
Panel[Labeled[Column[{Row[
      {Panel[Labeled[RadioButtonBar[Dynamic[sampOpt], {1 → "All", 2 → "Sample"},
          LabelStyle → (FontFamily → "Arial")], "Select image display option",
         Top, LabelStyle → Directive[FontSize → 12, FontFamily → "Arial"]]], " ",
       Panel[Labeled[InputField[Dynamic[kgImg], FieldSize → 5],
         "Enter no. of images to display.", Top,
         LabelStyle → Directive[FontSize → 12, Plain, FontFamily → "Arial"]]]}],
    Row[{Panel[Labeled[InputField[Dynamic[rowNum], FieldSize → 5],
         "Enter number of plots per grid row.", Top, LabelStyle →
          Directive[FontSize → 12, Plain, FontFamily → "Arial"]]], " ",
       Panel[Labeled[InputField[Dynamic[imgSize], FieldSize → 5],
         "Enter image size control parameter.", Top,
         LabelStyle → Directive[FontSize → 12, Plain, FontFamily → "Arial"]]]}],
    Panel[Labeled[InputField[Dynamic[grdSize], FieldSize → 5],
       "Enter grid size control parameter.", Top,
       LabelStyle → Directive[FontSize → 12, Plain, FontFamily → "Arial"]]]},
   Center], "Processed Image Display Options", Top,
  LabelStyle → Directive[FontSize → 16, Bold, FontFamily → "Arial"]]]
sampOpt = 1; kgImg = 50; rowNum = 4; imgSize = 100; grdSize = 700;
```

Out[•]=

Processed Image Display Options

Select image display option
(•) All ( ) Sample

Enter no. of images to display.
kgImg

Enter number of plots per grid row.
rowNum

Enter image size control parameter.
imgSize

Enter grid size control parameter.
grdSize

Display original images.

In[•]:=
```
If[sampOpt == 1,
  kgDisp = kgTrain;
  gridTitle = "Original Images (All)";
  imgDisp = Table[i, {i, kgTrain}],
  kgDisp = kgImg;
  gridTitle = "Original Images (Random Sample)";
  imgDisp = Sort[RandomSample[Range[kgTrain], kgDisp]]];

t1 = N[kgDisp / rowNum];
t2 = IntegerPart[N[kgDisp / rowNum]];
If[t1 - t2 > 0., kg2 = (t2 + 1) * rowNum, kg2 = t2 * rowNum];
imgTable = Table[" ", {kg2}];
Do[imgTable⟦k⟧ = Labeled[ImageResize[x1⟦imgDisp⟦k⟧⟧, imgSize],
   objNamesTrain⟦imgDisp⟦k⟧⟧, Top, LabelStyle →
    Directive[FontSize → 12, Italic, FontFamily → "Arial"]], {k, kgDisp}]
imgPlt1 = Labeled[GraphicsGrid[Partition[imgTable, rowNum],
   Frame → All, ImageSize → grdSize], gridTitle, Top,
  LabelStyle → Directive[FontSize → 18, Bold, FontFamily → "Arial"]]
```

Read in training-set group classification list & assemble training-set data file

In[•]:=
```
filenamein = SystemDialogInput["FileOpen"];
groupTrain = Import[filenamein, "CSV"];
filenamein

groupTrain = Flatten[groupTrain];
{nTrain} = Dimensions[groupTrain];
gpNamesTrain = Union[groupTrain];
nGps = Length[Union[groupTrain]];
mTrain = Length[gpNamesTrain];

trainData = Table[" ", {nTrain}];
Do[
 Do[
  If[groupTrain⟦i⟧ == gpNamesTrain⟦j⟧, trainData⟦i⟧ = x1⟦i⟧ → gpNamesTrain⟦j⟧],
  {j, nGps}], {i, nTrain}]
Print["No. of labels: " , nTrain]
```

Out[•]=
```
/Users/n.macleod/Desktop/Dragonflies (Final)/Data
  & Results/Images (ML)/Hindwings/Landscape Groups.csv

No. of labels: 230
```

Build a generic version of the LeNet CNN and train using the training-set contrasts

In[∘]:=
```
toRule[{img1_ → l1_, img2_ → l2_}] := {img1, img2} → l1 ≠ l2;
comb = (kgTrain * kgTrain) * 0.8;
trainingData = Table[toRule@RandomSample[trainData, 2], comb];

lenet = NetChain[{
    ConvolutionLayer[10, 4], Ramp, PoolingLayer[2, 2],
    ConvolutionLayer[20, 4], Ramp, PoolingLayer[2, 2],
    FlattenLayer[], 2},
   "Input" → NetEncoder[{"Image", {40, 40}, "Grayscale"}]];
net = NetPairEmbeddingOperator[lenet];
results =
  NetTrain[net, trainingData, All, MaxTrainingRounds → 3, TargetDevice → "CPU"]
trained = results["TrainedNet"];
embedding = NetExtract[trained, "Net"];

groups = KeySort@GroupBy[trainData, Last → First];
points = Map[embedding /* Normal, groups];
c = Classify[points];
cm = ClassifierMeasurements[c, points];
netTrainSum = Labeled[
   GraphicsColumn[{results["LossEvolutionPlot"]}], "Loss Evolution Plot", Top,
   LabelStyle → Directive[Black, FontSize → 18, Bold, FontFamily → "Arial"]];
```

Display error loss evolution plot

In[∘]:=
```
netTrainSum
```

Export error loss evolution plot

In[∘]:=
```
filenameout = SystemDialogInput["FileSave"];
Export[filenameout, netTrainSum, "TIFF", ImageResolution → 150]
```

Out[∘]=
```
/Users/n.macleod/Desktop/Dragonflies (Final)/Data & Results/Images
  (ML)/Hindwings/Landscape Groups Results/Loss Evolution Plot.tif
```

Calculate training-set confusion matrix (optional).

```
In[•]:= confMatrix = ClassifierMeasurements[c, points, "ConfusionMatrix"];
nc = Length[gpNamesTrain];
cMatrix = Table[" ", {nc + 4}, {nc + 4}];
cMatrix⟦1, 1⟧ = Text["Groups"];
cMatrix⟦1, nc + 2⟧ = Text[Rotate["No. Correct", 90 Degree]];
cMatrix⟦1, nc + 3⟧ = Text[Rotate["Group Total", 90 Degree]];
cMatrix⟦1, nc + 4⟧ = Text[Rotate["Percent Correct", 90 Degree]];
Do[cMatrix⟦i + 1, 1⟧ = Text[gpNamesTrain⟦i⟧], {i, nc}];
Do[cMatrix⟦1, i + 1⟧ = Text[Rotate[gpNamesTrain⟦i⟧, 90 Degree]], {i, nc}]
Do[
  If[confMatrix⟦i, j⟧ == 0, cMatrix⟦i + 1, j + 1⟧ = Text["-"],
   cMatrix⟦i + 1, j + 1⟧ = Text[confMatrix⟦i, j⟧]], {j, nc}, {i, nc}];
cMatrix⟦nc + 2, 1⟧ = Text["No. Correct"];
cMatrix⟦nc + 3, 1⟧ = Text["Group Total"];
cMatrix⟦nc + 4, 1⟧ = Text["Percent Correct"];
Do[cMatrix⟦i + 1, nc + 2⟧ = Text[confMatrix⟦i, i⟧], {i, nc}];
cMatrix⟦nc + 2, nc + 2⟧ = Text[Total[Diagonal[confMatrix]]];
cMatrix⟦nc + 2, nc + 3⟧ = Text[Total[Total[confMatrix]]];
cMatrix⟦nc + 2, nc + 4⟧ = Text[PaddedForm[
    N[Total[Diagonal[confMatrix]] / Total[Total[confMatrix]]] * 100.0, {4, 1}]];
Do[cMatrix⟦i + 1, nc + 3⟧ = Text[Total[confMatrix⟦i, All⟧]], {i, nc}]
Do[
 If[
  confMatrix⟦i, i⟧ == 0, cMatrix⟦i + 1, nc + 4⟧ = Text["0.0"], cMatrix⟦i + 1, nc + 4⟧ =
   Text[PaddedForm[N[Take[confMatrix⟦i, i⟧] / Total[Take[confMatrix⟦i, All⟧]]] *
      100.0, {4, 1}]]], {i, nc}]
Do[cMatrix⟦nc + 2, i + 1⟧ = Text[confMatrix⟦i, i⟧], {i, nc}]
Do[cMatrix⟦nc + 3, i + 1⟧ = Text[Total[confMatrix⟦All, i⟧]], {i, nc}]
cMatrix⟦nc + 3, nc + 2⟧ = Text[Total[Total[confMatrix]]];
cMatrix⟦nc + 4, nc + 2⟧ = Text[PaddedForm[
    N[Total[Diagonal[confMatrix]] / Total[Total[confMatrix]]] * 100.0, {4, 1}]];
Do[
 If[
  confMatrix⟦i, i⟧ == 0, cMatrix⟦nc + 4, i + 1⟧ = Text["0.0"],
  cMatrix⟦nc + 4, i + 1⟧ =
   Text[PaddedForm[N[Take[confMatrix⟦i, i⟧] / Total[Take[confMatrix⟦All, i⟧]]] *
      100.0, {4, 1}]]], {i, nc}]

trainConfMatrix =
 Labeled[Grid[cMatrix, Alignment → {{1 → Right, nc + 4 → Right}, {Bottom, Right}},
   Frame → True, Dividers → {{2 → True, -4 → True}, {2 → True, -4 → True}}],
  "Training-Set Confusion Matrix", Top,
  LabelStyle → Directive[FontSize → 16, Bold, FontFamily → "Arial"]]
```

Export training-set confusion matrix

In[•]:=
```
cMatrix = Table[" ", {nc + 4}, {nc + 4}];
cMatrix[[1, 1]] = "Groups";
cMatrix[[1, nc + 2]] = "No. Correct";
cMatrix[[1, nc + 3]] = "Group Total";
cMatrix[[1, nc + 4]] = "Percent Correct";
Do[cMatrix[[i + 1, 1]] = gpNamesTrain[[i]], {i, nc}];
Do[cMatrix[[1, i + 1]] = gpNamesTrain[[i]], {i, nc}];
Do[
  If[confMatrix[[i, j]] == 0, cMatrix[[i + 1, j + 1]] = "-",
   cMatrix[[i + 1, j + 1]] = confMatrix[[i, j]]], {j, nc}, {i, nc}];
cMatrix[[nc + 2, 1]] = "No. Correct";
cMatrix[[nc + 3, 1]] = "Group Total";
cMatrix[[nc + 4, 1]] = "Percent Correct";
Do[cMatrix[[i + 1, nc + 2]] = confMatrix[[i, i]], {i, nc}];
cMatrix[[nc + 2, nc + 2]] = Total[Diagonal[confMatrix]];
cMatrix[[nc + 2, nc + 3]] = Total[Total[confMatrix]];
cMatrix[[nc + 2, nc + 4]] =
  N[Total[Diagonal[confMatrix]] / Total[Total[confMatrix]]] * 100.0;
Do[cMatrix[[i + 1, nc + 3]] = Total[confMatrix[[i, All]]], {i, nc}];
Do[
  If[
   confMatrix[[i, i]] == 0, cMatrix[[i + 1, nc + 4]] = 0.0,
   cMatrix[[i + 1, nc + 4]] = N[Take[confMatrix[[i, i]]] /
       Total[Take[confMatrix[[i, All]]]] * 100.0], {i, nc}];
Do[cMatrix[[nc + 2, i + 1]] = confMatrix[[i, i]], {i, nc}];
Do[cMatrix[[nc + 3, i + 1]] = Total[confMatrix[[All, i]]], {i, nc}];
cMatrix[[nc + 3, nc + 2]] = Total[Total[confMatrix]];
cMatrix[[nc + 4, nc + 2]] =
  N[Total[Diagonal[confMatrix]] / Total[Total[confMatrix]]] * 100.0;
Do[
  If[
   confMatrix[[i, i]] == 0, cMatrix[[nc + 4, i + 1]] = 0.0,
   cMatrix[[nc + 4, i + 1]] =
    N[Take[confMatrix[[i, i]]] / Total[Take[confMatrix[[All, i]]]] *
     100.0], {i, nc}];

filenameout = SystemDialogInput["FileSave"];
Export[filenameout, cMatrix, "CSV", "TextDelimiters" → ""]
```

Out[•]=
```
/Users/n.macleod/Desktop/Dragonflies (Final)/Data & Results/Images
  (ML)/Hindwings/Landscape Groups Results/Confusion Matrix (Raw).csv
```

Log Likelihood test for training set

In[•]:=
```
phi = Abs[ClassifierMeasurements[c, points, "LogLikelihood"]]
```

Out[•]= 4.4309×10^{-6}

In[•]:=
```
m = 40;
dof = m * (nGps - 1);
prob = N[(1 - CDF[ChiSquareDistribution[m * (nGps - 1)], phi]) * 100];
FTable = Table[" ", {3}, {2}];
FTable[[1, 1]] = "Log Likelihood Ratio";
FTable[[2, 1]] = "Degrees of Freedom";
FTable[[3, 1]] = "Probability (%)";
FTable[[1, 2]] = PaddedForm[phi, {4, 3}];
FTable[[2, 2]] = dof;
FTable[[3, 2]] = PaddedForm[prob, {5, 3}];
rng = phi * 2;
If[prob ≤ 5.0, hcolor = Green, hcolor = Red];
phiLegend =
  Labeled[Grid[FTable, BaseStyle → Directive[FontSize → 10, FontFamily → "Arial"],
    Alignment → {{Left, Right}}, Frame → True,
    Dividers → {{True, True}, {True}}], "Likelihood Ratio Table", Top,
   LabelStyle → Directive[FontSize → 12, Bold, FontFamily → "Arial"]];
plot5 = Plot[PDF[ChiSquareDistribution[dof], x], {x, 0, rng},
   ImageSize → 500, Filling → Bottom, FillingStyle → hcolor,
   PlotRange → Full, AxesLabel → {"χ² Ratio", "Prob. Density"},
   LabelStyle → Directive[Black, FontSize → 12, FontFamily → "Arial"]];
probPlot5 = Labeled[Overlay[{plot5, phiLegend}, Alignment → Right],
  "Parametric Probability Results", Top,
  LabelStyle → Directive[Black, Bold, FontSize → 16, FontFamily → "Arial"]]
```

Export Log Likelihood test results

In[•]:=
```
filenameout = SystemDialogInput["FileSave"];
Export[filenameout, probPlot5, "TIFF", ImageResolution → 150]
```

Out[•]= /Users/n.macleod/Desktop/Dragonflies (Final)/Data & Results/Images (ML)/Hindwings/Landscape Groups Results/Log-likelihood Test (PM).tif

Create 2 D feature space scatterplot .

Specify 2 D plot options.

You must run this code after you read in the data so it can pick up the proper variable names.

In[•]:=
```
axisNames = Table[StringJoin["FS Axis", ToString[i]], {i, 2}];
Panel[
 Labeled[Column[{Row[{Panel[Labeled[PopupMenu[Dynamic[xAxisName], axisNames],
       "Select variable to be plotted on x-Axis.", Top, LabelStyle →
        Directive[FontSize → 12, Bold, FontFamily → "Arial"]]], "  ",
     Panel[Labeled[PopupMenu[Dynamic[yAxisName], axisNames],
       "Select variable to be plotted on y-Axis.", Top,
       LabelStyle → Directive[FontSize → 12, Bold, FontFamily → "Arial"]]]}],
   Row[{
     Panel[Labeled[PopupMenu[Dynamic[pltAspect],
        {1 → "Golden Ratio Plot", 2 → "Square Plot (equi-length axes)",
         3 → "True-Scale Plot (actual axis scales)"}],
       "Enter plot aspect ratio type.", Top, LabelStyle →
        Directive[FontSize → 12, Bold, FontFamily → "Arial"]]], "  ",
     Panel[Labeled[PopupMenu[Dynamic[lch], {1 → "Simple scatterplot",
         2 → "Scatterplot w/ convex hulls"}],
       "Show group domians?", Top, LabelStyle →
        Directive[FontSize → 12, Bold, FontFamily → "Arial"]]], "  ",
     Panel[Labeled[PopupMenu[Dynamic[ptsJoin], {1 → "No", 2 → "Yes"}],
       "Join datapoints?", Top,
       LabelStyle → Directive[FontSize → 12, Bold, FontFamily → "Arial"]]]}],
   Row[{Panel[Labeled[InputField[Dynamic[pltSize], FieldSize → 10],
       "Enter plot size value.", Top,
       LabelStyle → Directive[FontSize → 12, Bold, FontFamily → "Arial"]]],
     "  ", Panel[Labeled[InputField[Dynamic[pltPad], FieldSize → 10],
       "Enter plot margin padding value.", Top, LabelStyle →
        Directive[FontSize → 12, Bold, FontFamily → "Arial"]]], "  ",
     Panel[Labeled[InputField[Dynamic[iconSize], FieldSize → 10],
       "Enter plot icon size value.", Top, LabelStyle →
        Directive[FontSize → 12, Bold, FontFamily → "Arial"]]]}]}, Center],
  "2D Plot Options", Top, LabelStyle → Directive[FontSize → 18,
    Bold, FontFamily → "Arial"]]]
pltSize = 500; iconSize = 0.03; pltPad = 0.1; xAxisName = axisNames〚1〛;
yAxisName = axisNames〚2〛;
 ptsJoin = 1; dataTrans = 1;  pltAspect = 1 ; lch = 2;
```

Out[∘]=

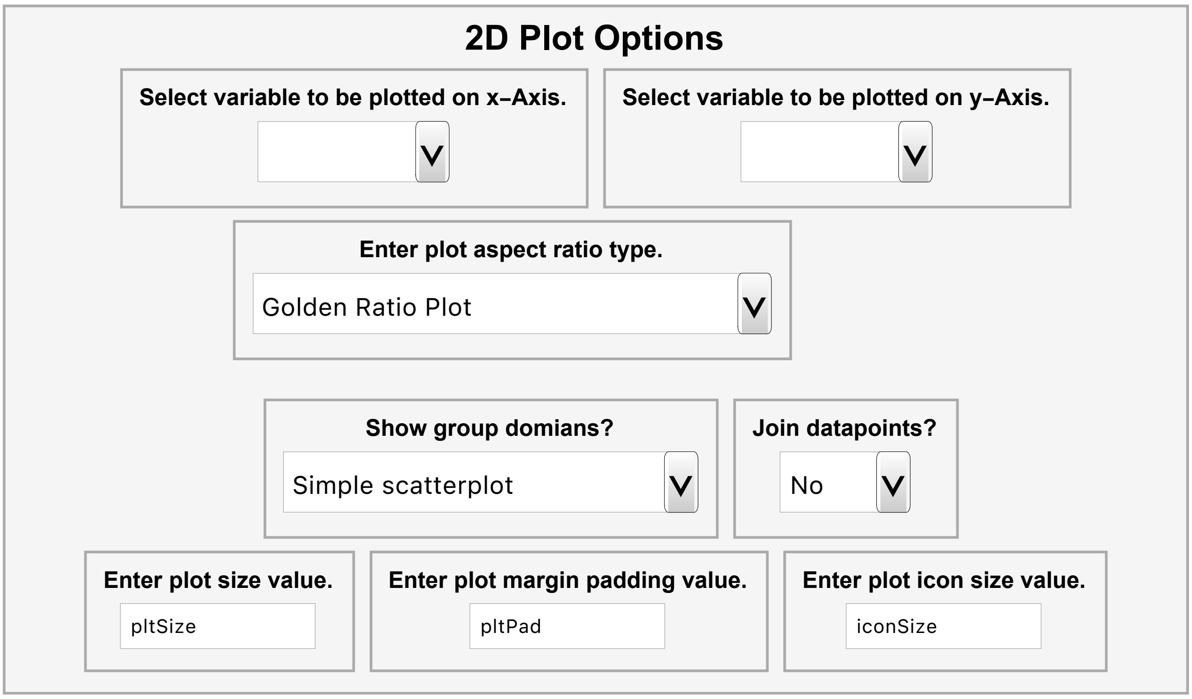

Plot trained feature space

In[∘]:=

```
points = Table[0.0, {kgTrain}, {2}];
Do[points⟦i⟧ = embedding[trainData⟦i, 1⟧], {i, kgTrain}];

pltTable = Table[" ", {nGps}, {3}];
Do[If[xAxisName == axisNames⟦j⟧, axis1 = j], {j, nGps}];
Do[If[yAxisName == axisNames⟦j⟧, axis2 = j], {j, nGps}];

groupNames = gpNamesTrain;
numGroups = Length[groupNames];
groupPosns =
  Table[Flatten[Position[groupTrain, groupNames⟦i⟧, 1]], {i, numGroups}];
pointsT = Transpose[points];

xAxis = pointsT⟦axis1⟧; yAxis = pointsT⟦axis2⟧;
lab1 = StringJoin[{"Feature Space Axis ", ToString[axis1]}];
lab2 = StringJoin[{"Feature Space Axis ", ToString[axis2]}];
maxx = Max[xAxis];
minx = Min[xAxis];
maxy = Max[yAxis];
miny = Min[yAxis];

If[pltAspect == 1 || pltAspect == 3,
  xPlotLow = minx; xPlotHi = maxx; yPlotLow = miny; yPlotHi = maxy];
If[pltAspect == 2,
  If[minx > miny,
   xPlotLow = miny; yPlotLow = miny,
```

```
    xPlotLow = minx; yPlotLow = minx ]];
If[pltAspect == 2,
  If[maxx < maxy,
   xPlotHi = maxy; yPlotHi = maxy,
   xPlotHi = maxx; yPlotHi = maxx]];
If[pltAspect == 1, aRatio = 1 / N[GoldenRatio]];
If[pltAspect == 2, aRatio = 1];
If[pltAspect == 3, aRatio = Automatic];

tmpPoints = Transpose[List[xAxis, yAxis]];
pltPoints = Table[tmpPoints⟦groupPosns⟦j⟧⟧, {j, numGroups}];
iconList = Flatten[Table[
    {Graphics[{EdgeForm[{Thickness[0.005], Black}],
       Hue[N[((numGroups + 1) - j) / numGroups]],
       Disk[{0, 0}, Scaled[iconSize]]}]}, {j, numGroups}]];

If[lch == 1 || ptsJoin == 1,
 Do[
  pltTable⟦k, 1⟧ =
   ListPlot[pltPoints⟦k⟧, AspectRatio → aRatio, Frame → True, Joined → False,
    Axes → False, PlotRange → {{xPlotLow, xPlotHi}, {yPlotLow, yPlotHi}},
    PlotRangePadding → Scaled[pltPad], Ticks → Automatic, FrameLabel →
     {lab1, lab2}, PlotMarkers → iconList⟦k⟧, ImageSize → pltSize, LabelStyle →
     Directive[FontSize → 14, Black, FontFamily → "Arial"]], {k, numGroups}],
 Do[
  pltTable⟦k, 1⟧ =
   ListPlot[pltPoints⟦k⟧, Frame → True, Axes → False, AspectRatio → aRatio,
    PlotRange → {{xPlotLow, xPlotHi}, {yPlotLow, yPlotHi}}, PlotRangePadding →
     Scaled[pltPad], Ticks → Automatic, FrameLabel → {lab1, lab2},
    LabelStyle → Directive[Black, FontSize → 14, FontFamily → "Arial"],
    ImageSize → pltSize, PlotStyle → Directive[Disk[],
      Hue[N[((numGroups + 1) - k) / numGroups]], EdgeForm[{Thickness[1.0], Black}],
      PointSize[Scaled[iconSize - 0.009]]]], {k, numGroups}]]

If[ptsJoin == 2,
  Do[
   pltTable⟦k, 2⟧ = ListLinePlot[pltPoints⟦k⟧,
     AspectRatio → aRatio, Frame → True, Joined → True, Axes → False,
     PlotStyle → Directive[Hue[N[((numGroups + 1) - k) / numGroups]], Thin],
     PlotRange → {{xPlotLow, xPlotHi}, {yPlotLow, yPlotHi}},
     PlotRangePadding → Scaled[pltPad], Ticks → Automatic,
     FrameLabel → {lab1, lab2}, ImageSize → pltSize, LabelStyle →
      Directive[FontSize → 14, Black, FontFamily → "Arial"]], {k, numGroups}]];

If[lch == 2,
  Do[
   hull = ConvexHullMesh[pltPoints⟦k⟧];
```

```
    pltTable⟦k, 3⟧ = HighlightMesh[hull,
      Style[2, Opacity[0.2], Hue[N[((numGroups + 1) - k) / numGroups]]],
      Frame → True, Axes → False, AspectRatio → aRatio,
      PlotRange → {{xPlotLow, xPlotHi}, {yPlotLow, yPlotHi}}, PlotRangePadding →
       Scaled[pltPad], Ticks → Automatic, FrameLabel → {lab1, lab2},
      LabelStyle → Directive[Black, FontSize → 14, FontFamily → "Arial"],
      ImageSize → pltSize], {k, numGroups}]];

If[ptsJoin == 1 && lch == 1, p0 = Show[pltTable⟦All, 1⟧]];
If[ptsJoin == 2 && lch == 1, p0 = Show[pltTable⟦All, 2⟧, pltTable⟦All, 1⟧]];
If[ptsJoin == 1 && lch == 2, p0 = Show[pltTable⟦All, 3⟧, pltTable⟦All, 1⟧]];
If[ptsJoin == 2 && lch == 2,
  p0 = Show[pltTable⟦All, 3⟧, pltTable⟦All, 2⟧, pltTable⟦All, 1⟧]];

p1 = Labeled[p0, "           Trained Feature-Space Plot", Top,
    LabelStyle → Directive[FontSize → 18, Bold, FontFamily → "Arial"]];
g1 = Grid[Table[
     {Graphics[{EdgeForm[{Thin, Black}], Hue[N[((numGroups + 1) - j) / numGroups]],
        Disk[]}]}, {j, numGroups}], Frame → False, ItemSize → 0.9];
g2 = Grid[Partition[groupNames, 1], Alignment → Left,
    BaseStyle → {FontFamily → "Arial", FontSize → 13, Italic}];
p2 = Labeled[Text[Grid[{{g1, g2}}, Alignment → Bottom, Frame → True]], "Legend",
    Top, LabelStyle → Directive[Black, FontSize → 18, Bold, FontFamily → "Arial"]];

plt2D = Grid[{{p1, p2}}, BaselinePosition → Top, Alignment → Top]
```

Export trained feature space plot

In[∘]:=
```
filenameout = SystemDialogInput["FileSave"];
Export[filenameout, plt2D, "TIFF", ImageResolution → 150]
```

Out[∘]= /Users/n.macleod/Desktop/Dragonflies (Final)/Data & Results/Images (ML)/Hindwings/Landscape Groups Results/Feature Space Plot.tif

Export trained feature space coordinates

In[∘]:=
```
filenameout = SystemDialogInput["FileSave"];
Export[filenameout, points, "CSV", "TextDelimiters" → ""]
```

Out[∘]= /Users/n.macleod/Desktop/Dragonflies (Final)/Data & Results/Images (ML)/Hindwings/Landscape Groups Results/Feature Space Points.csv

Calculate full jackknife estimate of classification performance.

Select user-specified options.

In[∘]:=
```
Panel[Labeled[
  Row[{Panel[Labeled[RadioButtonBar[Dynamic[jackMode], {1 → "Full Dataset",
        2 → "Partial Dataset"}, LabelStyle → (FontFamily → "Arial")],
      "Select jackknife estimate option", Top,
      LabelStyle → Directive[FontSize → 12, FontFamily → "Arial"]]], "  ",
    Panel[Labeled[InputField[Dynamic[pcentImg], FieldSize → 5],
      "Enter percent of images to use for partial jackknife estimate .", Top,
      LabelStyle → Directive[FontSize → 12, Plain, FontFamily → "Arial"]]]}],
  "Jacknife Estimate Options", Top, LabelStyle →
   Directive[FontSize → 18, Bold, , FontFamily → "Arial"]]]
pcentImg = 10;
```

Out[∘]=

Jacknife Estimate Options

Select jackknife estimate option
(●) Full Dataset ( ) Partial Dataset

Enter percent of images to use for partial jackknife estimate .
pcentI mg

Calculate jackknife estimate of classification performance.

In[∘]:=
```
If[jackMode == 2, paTrain = Round[N[kgTrain * (pcentImg / 100)]];
  paSample = Sort[RandomSample[Range[kgTrain], paTrain]]];
If[jackMode == 1, nTrain = kgTrain, nTrain = paTrain];
jackID = Table[" ", {nTrain}, {2}];
If[jackMode == 1, Do[jackID[[i, 1]] = groupTrain[[i]], {i, nTrain}],
 Do[jackID[[i, 1]] = groupTrain[[paSample[[i]]]], {i, nTrain}]]
```

In[∘]:=
```
paSample
jackID
```

Out[∘]=
```
{17, 24, 25, 62}
```

Out[∘]=
```
{{East-Indian Ocean,  }, {East-Indian Ocean,  },
 {East-Indian Ocean,  }, {Israel Med,  }}
```

In[∘]:=

```
Panel[
 Labeled[ProgressIndicator[Dynamic[i], {1, nTrain}], "Calculation Progress",
  Top, LabelStyle → Directive[FontSize → 12, Bold, FontFamily → "Arial"]]]

i = 1;
If[jackMode == 1,
  jackTrainData = Drop[trainData, {i}];
  jackGroupTrain = Drop[groupTrain, {i}];
  ,
  jackTrainData = Drop[trainData, {paSample⟦i⟧}];
  jackGroupTrain = Drop[groupTrain, {paSample⟦i⟧}]];

jackTrainingData = Table[toRule@RandomSample[jackTrainData, 2], comb];
jackResults = NetTrain[net, jackTrainingData,
   All, MaxTrainingRounds → 3, TargetDevice → "CPU"];
jackTrained = jackResults["TrainedNet"];
jackEmbedding = NetExtract[jackTrained, "Net"];

jackGroups = KeySort@GroupBy[jackTrainData, Last → First];
jackPoints = Map[embedding /* Normal, jackGroups];
jackC = Classify[jackPoints];

If[jackMode == 1,
  testImage = embedding[trainData⟦i, 1⟧],
  testImage = embedding[trainData[paSample⟦i⟧, 1]]];
jackID⟦i, 2⟧ = jackC[testImage]
```

Out[∘]=

Calculation Progress

Calculate full jackknife estimate of classification performance.

In[∘]:=
```
jackID = Table[" ", {kgTrain}, {2}];
Do[jackID⟦i, 1⟧ = groupTrain⟦i⟧, {i, kgTrain}];

Panel[Labeled[ProgressIndicator[Dynamic[i], {1, 3}], "Calculation Progress",
  Top, LabelStyle → Directive[FontSize → 12, Bold, FontFamily → "Arial"]]]

Do[
 jackTrainData = Drop[trainData, {i}];
 jackGroupTrain = Drop[groupTrain, {i}];
 jackTrainingData = Table[toRule@RandomSample[jackTrainData, 2], comb];
 jackResults = NetTrain[net, jackTrainingData,
   All, MaxTrainingRounds → 3, TargetDevice → "CPU"];
 jackTrained = jackResults["TrainedNet"];
 jackEmbedding = NetExtract[jackTrained, "Net"];

 jackGroups = KeySort@GroupBy[jackTrainData, Last → First];
 jackPoints = Map[embedding /* Normal, jackGroups];
 jackC = Classify[jackPoints];

 testImage = embedding[trainData⟦paSample⟦i⟧, 1⟧];
 jackID⟦i, 2⟧ = jackC[testImage], {i, 3}]
```

Out[∘]=

Calculation Progress

In[∘]:=
```
jackID
```

Out[∘]=
```
{{East-Indian Ocean, East-Indian Ocean}, {East-Indian Ocean, East-Indian Ocean},
 {Israel Med, Israel Med}, {Israel Med, Israel Med}}
```

Calculate partial jackknife estimate of classification performance.

In[∘]:=
```
paTrain = 25
paSample = Sort[RandomSample[Range[kgTrain], paTrain]]
```

Out[∘]=
```
25
```

Out[∘]=
```
{3, 4, 10, 23, 32, 37, 50, 70, 75, 76, 97, 100, 105,
 131, 136, 147, 166, 174, 180, 181, 187, 189, 203, 212, 225}
```

In[•]:=

```
jackID = Table[" ", {paTrain}, {2}];
Do[jackID⟦i, 1⟧ = groupTrain⟦paSample⟦i⟧⟧, {i, paTrain}];

Panel[
 Labeled[ProgressIndicator[Dynamic[i], {1, paTrain}], "Calculation Progress",
  Top, LabelStyle → Directive[FontSize → 12, Bold, FontFamily → "Arial"]]]

Do[
 jackTrainData = Drop[trainData, {paSample⟦i⟧}];
 jackGroupTrain = Drop[groupTrain, {paSample⟦i⟧}];
 jackTrainingData = Table[toRule@RandomSample[jackTrainData, 2], comb];
 jackResults = NetTrain[net, jackTrainingData,
   All, MaxTrainingRounds → 3, TargetDevice → "CPU"];
 jackTrained = jackResults["TrainedNet"];
 jackEmbedding = NetExtract[jackTrained, "Net"];

 jackGroups = KeySort@GroupBy[jackTrainData, Last → First];
 jackPoints = Map[embedding /* Normal, jackGroups];
 jackC = Classify[jackPoints];

 testImage = embedding[trainData⟦paSample⟦i⟧, 1⟧];
 jackID⟦i, 2⟧ = jackC[testImage], {i, paTrain}]
```

Out[•]=

Calculation Progress

Display jackknife confusion matrix.

In[•]:=

```
{nc} = Dimensions[gpNamesTrain];
jackConfMat = Table[0, {nc}, {nc}];

Do[
 Do[
  If[ jackID⟦i, 1⟧ == gpNamesTrain⟦j⟧ && jackID⟦i, 2⟧ == jackID⟦i, 1⟧,
   jackConfMat⟦j, j⟧ = jackConfMat⟦j, j⟧ + 1];
  If[ jackID⟦i, 1⟧ == gpNamesTrain⟦j⟧ && jackID⟦i, 2⟧ ≠ jackID⟦i, 1⟧,
   Do[
    If[jackID⟦i, 2⟧ == gpNamesTrain⟦jj⟧, jackConfMat⟦j, jj⟧ =
      jackConfMat⟦j, jj⟧ + 1], {jj, nGps}]], {j, nGps}], {i, paTrain}]

jcMatrix = Table[" ", {nGps + 4}, {nGps + 4}];
jcMatrix⟦1, 1⟧ = Text["Groups"];
jcMatrix⟦1, nGps + 2⟧ = Text[Rotate["No. Correct", 90 Degree]];
jcMatrix⟦1, nGps + 3⟧ = Text[Rotate["Group Total", 90 Degree]];
jcMatrix⟦1, nGps + 4⟧ = Text[Rotate["Percent Correct", 90 Degree]];
```

```
Do[jcMatrix⟦i + 1, 1⟧ = Text[gpNamesTrain⟦i⟧], {i, nGps}];
Do[jcMatrix⟦1, i + 1⟧ = Text[Rotate[gpNamesTrain⟦i⟧, 90 Degree]], {i, nGps}]
Do[
  If[jackConfMat⟦i, j⟧ == 0, jcMatrix⟦i + 1, j + 1⟧ = Text["-"],
   jcMatrix⟦i + 1, j + 1⟧ = Text[jackConfMat⟦i, j⟧]], {j, nGps}, {i, nGps}];
jcMatrix⟦nGps + 2, 1⟧ = Text["No. Correct"];
jcMatrix⟦nGps + 3, 1⟧ = Text["Group Total"];
jcMatrix⟦nGps + 4, 1⟧ = Text["Percent Correct"];
Do[jcMatrix⟦i + 1, nc + 2⟧ = Text[jackConfMat⟦i, i⟧], {i, nGps}];
jcMatrix⟦nGps + 2, nGps + 2⟧ = Text[Total[Diagonal[jackConfMat]]];
jcMatrix⟦nGps + 2, nGps + 3⟧ = Text[Total[Total[jackConfMat]]];
jcMatrix⟦nGps + 2, nGps + 4⟧ =
  Text[PaddedForm[N[Total[Diagonal[jackConfMat]] / Total[Total[jackConfMat]]] *
     100.0, {4, 1}]];
Do[jcMatrix⟦i + 1, nGps + 3⟧ = Text[Total[jackConfMat⟦i, All⟧]], {i, nGps}]
Do[
 If[
  jackConfMat⟦i, i⟧ == 0,
  cMatrix⟦i + 1, nGps + 4⟧ = Text["0.0"], jcMatrix⟦i + 1, nGps + 4⟧ = Text[
    PaddedForm[N[Take[jackConfMat⟦i, i⟧] / Total[Take[jackConfMat⟦i, All⟧]]] *
      100.0, {4, 1}]]]], {i, nGps}]
Do[jcMatrix⟦nGps + 2, i + 1⟧ = Text[jackConfMat⟦i, i⟧], {i, nGps}]
Do[jcMatrix⟦nGps + 3, i + 1⟧ = Text[Total[jackConfMat⟦All, i⟧]], {i, nGps}]
jcMatrix⟦nGps + 3, nc + 2⟧ = Text[Total[Total[jackConfMat]]];
jcMatrix⟦nGps + 4, nc + 2⟧ =
  Text[PaddedForm[N[Total[Diagonal[jackConfMat]] / Total[Total[jackConfMat]]] *
     100.0, {4, 1}]];
Do[
 If[
  jackConfMat⟦i, i⟧ == 0, cMatrix⟦nGps + 4, i + 1⟧ = Text["0.0"],
  jcMatrix⟦nGps + 4, i + 1⟧ = Text[
    PaddedForm[N[Take[jackConfMat⟦i, i⟧] / Total[Take[jackConfMat⟦All, i⟧]]] *
      100.0, {4, 1}]]]], {i, nGps}]

Labeled[Grid[jcMatrix, Alignment → {{1 → Right, nGps + 4 → Right}, {Bottom, Right}},
  Frame → True, Dividers → {{2 → True, -4 → True}, {2 → True, -4 → True}}],
 "Jackknife ID Confusion Matrix", Top,
 LabelStyle → Directive[FontSize → 16, Bold, FontFamily → "Arial"]]
```

Export jackknife ID confusion matrix

```
In[•]:= jcMatrix = Table[" ", {nGps + 4}, {nGps + 4}];
jcMatrix⟦1, 1⟧ = "Groups";
jcMatrix⟦1, nGps + 2⟧ = "No. Correct";
jcMatrix⟦1, nGps + 3⟧ = "Group Total";
jcMatrix⟦1, nGps + 4⟧ = "Percent Correct";
Do[jcMatrix⟦i + 1, 1⟧ = gpNamesTrain⟦i⟧, {i, nGps}];
Do[jcMatrix⟦1, i + 1⟧ = gpNamesTrain⟦i⟧, {i, nGps}];
Do[
  If[jackConfMat⟦i, j⟧ == 0, cMatrix⟦i + 1, j + 1⟧ = "-",
   jcMatrix⟦i + 1, j + 1⟧ = jackConfMat⟦i, j⟧], {j, nGps}, {i, nGps}];
jcMatrix⟦nGps + 2, 1⟧ = "No. Correct";
jcMatrix⟦nGps + 3, 1⟧ = "Group Total";
jcMatrix⟦nGps + 4, 1⟧ = "Percent Correct";
Do[jcMatrix⟦i + 1, nGps + 2⟧ = jackConfMat⟦i, i⟧, {i, nGps}];
jcMatrix⟦nGps + 2, nGps + 2⟧ = Total[Diagonal[jackConfMat]];
jcMatrix⟦nGps + 2, nGps + 3⟧ = Total[Total[jackConfMat]];
jcMatrix⟦nGps + 2, nGps + 4⟧ =
  N[Total[Diagonal[jackConfMat]] / Total[Total[jackConfMat]]] * 100.0;
Do[jcMatrix⟦i + 1, nc + 3⟧ = Total[jackConfMat⟦i, All⟧], {i, nGps}];
Do[
  If[
   jackConfMat⟦i, i⟧ == 0, jcMatrix⟦i + 1, nGps + 4⟧ = 0.0,
   jcMatrix⟦i + 1, nGps + 4⟧ = N[Take[jackConfMat⟦i, i⟧] /
        Total[Take[jackConfMat⟦i, All⟧]]] * 100.0], {i, nGps}];
Do[jcMatrix⟦nGps + 2, i + 1⟧ = jackConfMat⟦i, i⟧, {i, nGps}];
Do[jcMatrix⟦nGps + 3, i + 1⟧ = Total[jackConfMat⟦All, i⟧], {i, nGps}];
jcMatrix⟦nGps + 3, nGps + 2⟧ = Total[Total[jackConfMat]];
jcMatrix⟦nGps + 4, nGps + 2⟧ =
  N[Total[Diagonal[jackConfMat]] / Total[Total[jackConfMat]]] * 100.0;
Do[
  If[
   jackConfMat⟦i, i⟧ == 0, cMatrix⟦nGps + 4, i + 1⟧ = 0.0,
   jcMatrix⟦nGps + 4, i + 1⟧ =
    N[Take[jackConfMat⟦i, i⟧] / Total[Take[jackConfMat⟦All, i⟧]]] *
     100.0], {i, nGps}];

filenameout = SystemDialogInput["FileSave"];
Export[filenameout, jcMatrix, "CSV", "TextDelimiters" → ""]
```
