# Supplementary material for: What you sample is what you get: ecomorphological variation in Trithemis (Odonata, Libellulidae) dragonfly wings reconsidered
Source: BMC Ecol Evol. 2022 Apr 11;22:43. doi: 10.1186/s12862-022-01978-y (PMC8996507; doi:10.1186/s12862-022-01978-y)
Supplement: Supplementary file 3 — Additional file 3: Software Archive. [file 12862_2022_1978_MOESM3_ESM.zip › Additional Files 3/Software Archive/Eigenimage Axis Models (1.5).pdf]

Eigenimage Axis Models

This program reads in a set of data matrices produced by the PCA (eigenvectors), CVA Models (model coordinates), and Procrustes superposition (mean shape) programs, calculates form and texture shape models, and plots these models out for inspection, interpretation, and/or export. Use this program for analyses that have been performed on a gray – level (= monochrome) dataset.

Author : N. MacLeod

Version : 1.5

Date : 24 August 2021

Reference : MacLeod (2015)

Initialize libraries.

In[∘]:=
```
<< ComputationalGeometry`
```

Read in eigenvectors dataset.

In[∘]:=
```
filenamein = SystemDialogInput["FileOpen"]
eShpVectors = Import[filenamein, "CSV"];
```

Out[∘]=
```
/Users/n.macleod/Desktop/Butterflies 2/Eigenvectors.csv
```

Read in model coordinates dataset.

In[∘]:=
```
filenamein = SystemDialogInput["FileOpen"]
modelCoords = Import[filenamein, "CSV"];
{nModels, mModels1} = Dimensions[modelCoords];
usrAxes = nModels / 5;
Print["No. of modeled axes: ", usrAxes]
Print[" "];
```

Out[∘]=
```
/Users/n.macleod/Desktop/Butterflies 2/PCA Model Coords.csv
```

```
No. of modeled axes: 3
```

Read in mean form/texture dataset.

In[∘]:=
```
filenamein = SystemDialogInput["FileOpen"]
mShape = Import[filenamein, "CSV"];
```

Out[∘]=
```
/Users/n.macleod/Desktop/Butterflies 2/Butterflies - Mean Image.csv
```

Obtain dimensions of the model matrices.

In[•]:=
```
Panel[
 Panel[Grid[{{Style["Enter number of modelled axes.  ", Bold], SpanFromLeft},
     {InputField[Dynamic[usrAxes]], Dynamic[usrAxes];}}]] ×
  Panel[
   Grid[{{Style["Enter number of models per axis.  ", Bold], SpanFromLeft},
     {InputField[Dynamic[numModels]], Dynamic[numModels];}}]]]
numModels = 5;
```

Out[•]=

**Enter number of modelled axes.**	**Enter number of models per axis.**
usrAxes	numModels

Calculate models.

In[•]:=
```
{totModels, axes} = Dimensions[modelCoords];
modperAxis = totModels / usrAxes;
totModels = modperAxis * usrAxes;
mods = Table[0.0, {totModels}, {20}, {2}];

eShpVectorsT = Transpose[eShpVectors];
eVectorsTInv = Take[Inverse[eShpVectorsT], All, usrAxes];
modelCoordsR = Transpose[Take[modelCoords, All, usrAxes]];
models = eVectorsTInv.modelCoordsR;

{nCols, nC2} = Dimensions[models];
mods2 = Table[0.0, {nCols}, {nC2}];
Do[mods2⟦i, j⟧ = mShape⟦i⟧ + models⟦i, j⟧, {i, nCols}, {j, nC2}]
Do[mods2⟦i⟧ = Flatten[mods2⟦i⟧], {i, nCols}]
```

Obtain dimensions of the input image matrices and sizes of the output graphics.

In[∘]:=
```
Panel[Labeled[Column[
   {Row[{Panel[Labeled[PopupMenu[Dynamic[cSpace], {1 → "Color (RGB) Images", 2 →
           "Greyscale Images"}], "Specify image colorspace.", Top, LabelStyle →
         Directive[FontSize → 12, Plain, FontFamily → "Arial"]]], "  "}],
    Row[{Panel[Labeled[InputField[Dynamic[xAxisDim], FieldSize → 10],
         "Enter x-axis pixel dimension.", Top, LabelStyle →
          Directive[FontSize → 12, Plain, FontFamily → "Arial"]]], "  ",
      Panel[Labeled[InputField[Dynamic[yAxisDim], FieldSize → 10],
        "Enter y-axis pixel dimension.", Top, LabelStyle →
         Directive[FontSize → 12, Plain, FontFamily → "Arial"]]], "  ",
      Panel[Labeled[InputField[Dynamic[aRatio], FieldSize → 10],
        "Enter table cell aspect ratio.", Top,
        LabelStyle → Directive[FontSize → 12, Plain, FontFamily → "Arial"]]]}],
    Row[{Panel[Labeled[InputField[Dynamic[imgSize], FieldSize → 10],
         "Enter size of modeled images.", Top, LabelStyle →
          Directive[FontSize → 12, Plain, FontFamily → "Arial"]]], "  ",
      Panel[Labeled[InputField[Dynamic[tabSz], FieldSize → 10],
        "Enter size of image model grid.", Top,
        LabelStyle → Directive[FontSize → 12, Plain, FontFamily → "Arial"]]]}]},
   Center], "Model Reconstruction Plot Options", Top,
  LabelStyle → Directive[FontSize → 16, Bold, FontFamily → "Arial"]]]
cSpace =
 1;
xAxisDim = 32;
yAxisDim = 32;
aRatio = 0.5;
imgSize = 150;
tabSz = 1000;
```

Out[∘]=

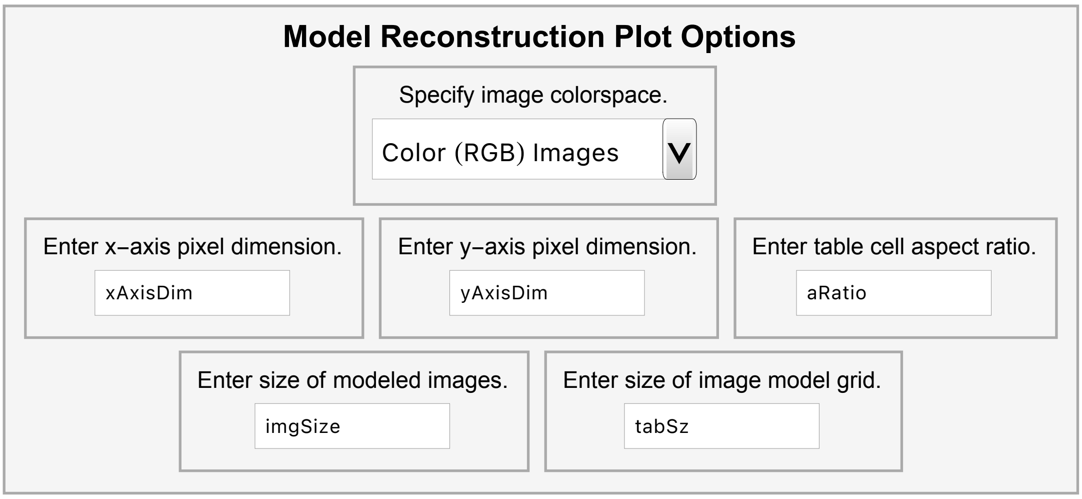

Parse data & construct image models.

```
In[•]:= numModels = nC2 / usrAxes;
objNames = Partition[Table[" ", {nC2}], numModels];
cTable = xAxisDim * yAxisDim;
model = Table[0, {xAxisDim}, {yAxisDim}];
gTable = Table[" ", {nC2}];
mTable = Table[" ", {usrAxes}, {numModels}];
Do[Do[objNames[[i, j]] = StringJoin["Axis ", ToString[i], ", Model ", ToString[j]],
  {j, numModels}], {i, usrAxes}]

If[cSpace == 1,
 Do[
  gTable[[k]] = ImageResize[Image[Partition[Partition[mods2[[All, k]], 3], xAxisDim],
     ColorSpace → "RGB", ImageSize → imgSize], imgSize], {k, nC2}],
 Do[gTable[[k]] = ImageResize[Image[Partition[mods2[[All, k]], xAxisDim],
     ColorSpace → "Grayscale"], imgSize], {k, nC2}]]

kount = 0;
Do[
 Do[
  kount = kount + 1;
  mTable[[i, j]] = Labeled[gTable[[kount]], objNames[[i, j]], Bottom,
    LabelStyle → Directive[FontSize → 14, FontFamily → "Arial"]],
  {j, numModels}], {i, usrAxes}]
mPlt = Labeled[GraphicsGrid[mTable, Frame → All, ImageSize → tabSz,
   AspectRatio → aRatio], "Matrix of Modeled Shapes", Top,
  LabelStyle → Directive[FontSize → 24, FontFamily → "Arial"]]
```

Export current grid plot.

```
In[•]:= filenameout = SystemDialogInput["FileSave"];
Export[filenameout, mPlt, "TIFF", ImageResolution → 150]

Out[•]= /Users/n.macleod/Desktop/Dragonflies
  (Final)/Data & Results/Images (PCA-CVA)/Hindwings/CVA
  Results/Water Body Groups/Along-Axis Shape Models (Grid).tif
```

Calculate difference plots

Specify difference plot threshold value.
This value establishes a "floor" on the difference plot that eliminates " background " difference " noise ". If you're not seeing much detail (or too much detail) in the difference plot try adjusting this value and recomputing the difference plot.

Select user – specified plot options

In[∘]:=
```
Panel[
 Labeled[Column[{Row[{Panel[Labeled[InputField[Dynamic[rowNum], FieldSize → 10],
        "Enter number of plots per grid row.", Top, LabelStyle →
         Directive[FontSize → 12, Plain, FontFamily → "Arial"]]], "  ",
      Panel[Labeled[InputField[Dynamic[thresh], FieldSize → 7],
        "Enter value of the plot floor threshold.", Top,
        LabelStyle → Directive[FontSize → 12, Plain, FontFamily → "Arial"]]]}],
    Row[{Panel[Labeled[InputField[Dynamic[imgSize], FieldSize → 10],
        "Enter image size control parameter.", Top, LabelStyle →
         Directive[FontSize → 12, Plain, FontFamily → "Arial"]]], "  ",
      Panel[Labeled[InputField[Dynamic[grdSize], FieldSize → 10],
        "Enter grid size control parameter.", Top,
        LabelStyle → Directive[FontSize → 12, Plain, FontFamily → "Arial"]]]}]},
   Center], "Difference Image Display Options", Top,
  LabelStyle → Directive[FontSize → 16, Bold, FontFamily → "Arial"]]]
rowNum = 5; imgSize = 150; grdSize = 1000; thresh = 0.0001;
```

Out[∘]=

Difference Image Display Options

Enter number of plots per grid row.	Enter value of the plot floor threshold.
rowNum	thresh
Enter image size control parameter.	Enter grid size control parameter.
imgSize	grdSize

Calculate and display axis difference plot.

```
In[•]:= difPlt1 = Table[" ", {5}, {usrAxes}];
diff2 = Table[0, {yAxisDim}, {xAxisDim}];
difPlt1[[1]] = Flatten[Take[Partition[gTable, numModels], All, 1]];
difPlt1[[2]] = Flatten[Take[Partition[gTable, numModels], All, -1]];
difPlt1 = Transpose[difPlt1];
difPlt2 = Table[" ", {usrAxes}, {5}];

Do[
 pdif = ImageSubtract[difPlt1[[k, 1]], difPlt1[[k, 2]]];
 pdata = ImageData[pdif];
 pMean = Mean[pdata];
 {imgy, imgx} = ImageDimensions[pdif];
 pdataAbs1 = Table[0.0, {imgx}, {imgy}];
 pdataAbs2 = pdataAbs1;
 If[cSpace == 1,
  Do[pdataAbs1[[i, j]] = Total[pdata[[i, j, All]]], {i, imgx}, {j, imgy}];
  Do[pdataAbs2[[i, j]] = Abs[Total[pdata[[i, j, All]]]], {i, imgx}, {j, imgy}]];
 If[cSpace == 2,
  Do[pdataAbs1[[i, j]] = pdata[[i, j]], {i, imgx}, {j, imgy}];
  Do[pdataAbs2[[i, j]] = Abs[pdata[[i, j]]], {i, imgx}, {j, imgy}]];

 difPlt2[[k, 1]] =
  Labeled[difPlt1[[k, 1]], StringJoin["Axis ", ToString[k], " (Low)"],
   Bottom, LabelStyle → Directive[FontSize → 14, FontFamily → "Arial"]];
 difPlt2[[k, 2]] = Labeled[difPlt1[[k, 2]],
   StringJoin["Axis ", ToString[k], " (High)"], Bottom,
   LabelStyle → Directive[FontSize → 14, FontFamily → "Arial"]];
 difPlt1[[k, 3]] = ArrayPlot[pdata, ImageSize → imgSize, Frame → False];
 difPlt2[[k, 3]] = Labeled[
   ArrayPlot[pdata, ImageSize → imgSize, Frame → False], "Superposed Images",
   Bottom, LabelStyle → Directive[FontSize → 14, FontFamily → "Arial"]];
 difPlt1[[k, 4]] = ArrayPlot[pdataAbs1, ColorFunction → "TemperatureMap",
   ImageSize → imgSize, Frame → False];
 difPlt2[[k, 4]] = Labeled[ArrayPlot[pdataAbs1, ColorFunction → "TemperatureMap",
    ImageSize → imgSize, Frame → False], "Difference Image", Bottom,
   LabelStyle → Directive[FontSize → 14, FontFamily → "Arial"]];
 difPlt1[[k, 5]] = ArrayPlot[pdataAbs2, ColorFunction → "TemperatureMap",
   ImageSize → imgSize, Frame → False];
 difPlt2[[k, 5]] = Labeled[ArrayPlot[pdataAbs2, ColorFunction → "TemperatureMap",
    ImageSize → imgSize, Frame → False], "Abs. Difference Image", Bottom,
   LabelStyle → Directive[FontSize → 14, FontFamily → "Arial"]], {k, usrAxes}]

difGrid2 = Labeled[GraphicsGrid[difPlt2, Frame → All, ImageSize → grdSize,
   AspectRatio → aRatio, Spacings → 0], "Matrix of Shape-Difference Models",
  Top, LabelStyle → Directive[FontSize → 24, FontFamily → "Arial"]]
```

Export difference plot grid .

In[∘]:=
```
filenameout = SystemDialogInput["FileSave"];
Export[filenameout, difGrid2, "TIFF", ImageResolution → 150]
```

Out[∘]=
```
/Users/n.macleod/Desktop/Dragonflies (Final)/Data
  & Results/Images (PCA-CVA)/Hindwings/CVA Results/Water
  Body Groups/Along-Axis Shape-Difference Models (Grid).tif
```

Dsplay axis difference plot without labeling.

In[∘]:=
```
difGrid1 = Labeled[GraphicsGrid[difPlt1, Frame → All, ImageSize → 1000,
    AspectRatio → aRatio, Spacings → 0], "Matrix of Shape-Difference Models",
  Top, LabelStyle → Directive[FontSize → 24, FontFamily → "Arial"]]
```

Export difference plot grid without labeling .

```
filenameout = SystemDialogInput["FileSave"];
Export[filenameout, difGrid1, "TIFF"]
```

```
/Users/nm/Desktop/Carnivores (Large Dataset)/CVA
  Results/Along-Axis Difference Models (w:o Labels).tif
```

Display modeled image or image difference plot at a larger size

Obtain plot control parameters

In[•]:=
```
Panel[Labeled[Column[{Row[{Panel[Labeled[InputField[Dynamic[n2], FieldSize → 5],
        "Enter image matrix row number.", Top, LabelStyle →
         Directive[FontSize → 12, Plain, FontFamily → "Arial"]]], "  ",
      Panel[Labeled[InputField[Dynamic[m2], FieldSize → 5],
        "Enter image matrix column number.", Top,
        LabelStyle → Directive[FontSize → 12, Plain, FontFamily → "Arial"]]]}],
    Row[{Panel[Labeled[InputField[Dynamic[imgSize2], FieldSize → 5],
        "Enter length of displayed image (in pixels).", Top,
        LabelStyle → Directive[FontSize → 12, Plain, FontFamily → "Arial"]]]}]},
   Center], "Single Model Display Options", Top, LabelStyle →
   Directive[FontSize → 16, Bold, FontFamily → "Arial"]]]
n2 = 1; m2 = 5; imgSize2 = 500;
```

Out[•]=

Single Model Display Options

Enter image matrix row number. [n2]

Enter image matrix column number. [m2]

Enter length of displayed image (in pixels). [imgSize2]

Display modeled image or image difference plot at a larger size.

In[•]:=
```
If[m2 == 1, lab = StringJoin["Axis Model (Low): Axis ", ToString[n2]]];
If[m2 == 2, lab = StringJoin["Axis Model (High): Axis ", ToString[n2]]];
If[m2 == 3,
  lab = StringJoin["Superimposed Images (Low-High): Axis ", ToString[n2]]];
If[m2 == 4, lab = StringJoin["Difference Image (Blue=Low, Red=High): Axis ",
    ToString[n2]]];
If[m2 == 5, lab = StringJoin[
    "Absolute Difference Image (Blue=Low, Red=High): Axis ", ToString[n2]]];
imgDisplay = Labeled[ImageResize[difPlt1⟦n2, m2⟧, imgSize2], lab,
  Top, LabelStyle → Directive[FontSize → 18, FontFamily → "Arial"]]
```

Export detail plot.

In[•]:=
```
filenameout = SystemDialogInput["FileSave"];
Export[filenameout, imgDisplay, "TIFF", ImageResolution → 150]
```

Out[•]=
```
/Users/n.macleod/Desktop/Dragonflies (Final)/Data
  & Results/Images (PCA-CVA)/Hindwings/CVA Results/Water
  Body Groups/Along-Axis Shape-Difference Models (Detail).tif
```
