# Supplementary material for: What you sample is what you get: ecomorphological variation in Trithemis (Odonata, Libellulidae) dragonfly wings reconsidered
Source: BMC Ecol Evol. 2022 Apr 11;22:43. doi: 10.1186/s12862-022-01978-y (PMC8996507; doi:10.1186/s12862-022-01978-y)
Supplement: Supplementary file 3 — Additional file 3: Software Archive. [file 12862_2022_1978_MOESM3_ESM.zip › Additional Files 3/Software Archive/Axis Models (CPT & Strobe) (1.8).pdf]

Axis Models (Coordinate Point & Strobe Plots)

This program reads in a set of data matrices produced by the PCA (eigenvectors),
CVA Models (model coordinates), and Procrustes superposition (mean shape) programs,
calculates coordinate plot shape models and strobe plots for each of the model coordinate positions,
and plots these models out for inspection, interpretation, and/or export.

Author : N. MacLeod

Version : 1.8
Date : 12 May 2017

Reference : MacLeod, N., 2009,
Form & shape models : Palaeontological Association Newsletter, v. 72, p. 14–27.

Initialize libraries.

```
In[•]:= << ComputationalGeometry`
```

Read in eigenvectors dataset.

```
In[•]:= filenamein = SystemDialogInput["FileOpen"]
        vectors = Import[filenamein, "CSV"];

Out[•]= /Users/n.macleod/Desktop/Drangonflies (Final)/Data
          & Results/GM/ Hindwings/PCA Results/Eigenvectors.csv
```

Read in model coordinates dataset.

```
In[•]:= filenamein = SystemDialogInput["FileOpen"]
        mCoords = Import[filenamein, "CSV"];
        {ax, ay} = Dimensions[mCoords];

Out[•]= /Users/n.macleod/Desktop/Drangonflies (Final)/Data & Results/GM/
          Hindwings/CVA Results/Water Body Groups/PCA-CVA Model Coords.csv
```

Read in mean shape dataset (optional, use for Procrustes – aligned data).

```
In[•]:= filenamein = SystemDialogInput["FileOpen"]
        mShape = Import[filenamein, "CSV"];

Out[•]= /Users/n.macleod/Desktop/Drangonflies
          (Final)/Data & Results/GM/ Hindwings/Mean Shape.csv
```

Obtain interactive control parameters.

In[•]:=
```
Panel[Labeled[
  Column[{Row[{Panel[Labeled[RadioButtonBar[Dynamic[modOrigin], {1 → "PCA",
          2 → "CVA"}], "Model Orign", Top, LabelStyle →
         Directive[FontSize → 12, Bold, FontFamily → "Arial"]]], "  ",
      Panel[Labeled[RadioButtonBar[Dynamic[modSet],
         {1 → "Along-axis Models", 2 → "Group-Differece Models",
          3 → "Subspace Models"}], "Model Type", Top,
        LabelStyle → Directive[FontSize → 12, Bold, FontFamily → "Arial"]]]}],
    Row[{Panel[Labeled[InputField[Dynamic[axes], FieldSize → 5],
        "No. of Modeled Axes/Chords", Top, LabelStyle →
         Directive[FontSize → 12, Bold, FontFamily → "Arial"]]], "  ",
      Panel[Labeled[InputField[Dynamic[mPerAxis], FieldSize → 5],
        "No. of Models per Axis/Row", Top,
        LabelStyle → Directive[FontSize → 12, Bold, FontFamily → "Arial"]]],
      "  ", Panel[Labeled[InputField[Dynamic[rows], FieldSize → 5],
        "No. of Modelled Rows (Subspace Models Only)", Top,
        LabelStyle → Directive[FontSize → 12, Bold, FontFamily → "Arial"]]]}],
    Row[{Panel[Labeled[InputField[Dynamic[isf], FieldSize → 5],
        "Icon Size Value", Top, LabelStyle →
         Directive[FontSize → 12, Bold, FontFamily → "Arial"]]], "  ",
      Panel[Labeled[InputField[Dynamic[pltSz], FieldSize → 5],
        "Plot Size Value", Top, LabelStyle →
         Directive[FontSize → 12, Bold, FontFamily → "Arial"]]], "  ",
      Panel[Labeled[InputField[Dynamic[grdSz], FieldSize → 5],
        "Grid Size Value", Top,
        LabelStyle → Directive[FontSize → 12, Bold, FontFamily → "Arial"]]]}]},
   Center], "Modelling Control Parameters", Top, LabelStyle →
   Directive[FontSize → 18, Bold, FontFamily → "Arial"]]]
modOrigin =
 1;
modSet = 1;
rows = 4;
mPerAxis = 5; isf = 0.03; modType = 1;
pltSz = 100;
axes = IntegerPart[N[ax / 5]]; grdSz = 900;
```

Out[•]=

Modelling Control Parameters

Model Orign

(•) PCA ( ) CVA

Model Type

(•) Along–axis Models ( ) Group–Differece Models ( ) Subspace Models

No. of Modeled Axes/Chords

axes

No. of Models per Axis/Row

mPerAx⋱
is

No. of Modelled Rows (Subspace Models Only)

rows

Icon Size Value

isf

Plot Size Value

pltSz

Grid Size Value

grdSz

Calculate models.

In[•]:=

```
{nModels, m2} = Dimensions[mCoords];
{m, tmp} = Dimensions[vectors];

If[modOrigin == 1,
  vectorsInv = Take[Inverse[vectors], axes, All];
  models = Take[mCoords, All, axes].vectorsInv];

If[modOrigin == 2,
  vectorsInv = Take[Inverse[vectors], axes, All];
  models = Take[mCoords, All, axes].vectorsInv];

If[modOrigin == 3,
  vectorsInv = Take[Inverse[vectors], m2, All];
  models = mCoords.vectorsInv];

mShapeF = Flatten[mShape];
Do[models[[i]] = Partition[models[[i]] + mShapeF, 2], {i, nModels}]
```

Plot models (parsed by rows).

In[•]:=

```
If[modSet == 1,
  modelPlots = Table[" ", {axes * (mPerAxis + 1)}];
  rw = axes];
If[modSet == 2,
  modelPlots = Table[" ", {axes * (mPerAxis)}];
  rw = axes];
```

```
If[modSet == 3,
  modelPlots = Table[" ", {rows * mPerAxis}];
  rw = rows];

maxi = Max[models];
mini = Min[models];
If[modType == 1, jn = False, jn = True];
k1 = 0;
k2 = 0;
Do[
 Do[
  k1 = k1 + 1;
  k2 = k2 + 1;
  icon = Graphics[{EdgeForm[{Thin, Black}],
     Hue[1 - ((i - 1) * 0.1)], Disk[{0, 0}, Scaled[isf]]}];
  If[modType == 1, pltFile = models〚k2〛,
   pltFile = Append[models〚k2〛, models〚k2, 1〛]];
  modelPlots〚k1〛 = ListPlot[pltFile, Joined → False,
    PlotStyle → {Black, Thickness[(maxi - mini) / 100]},
    AspectRatio → 1, Frame → False, Axes → False, ImageSize → pltSz,
    FrameLabel → {"Axis1", "Axis2"}, PlotRange → {{mini, maxi}, {mini, maxi}},
    PlotRangePadding → Scaled[.05], PlotMarkers → icon], {i, mPerAxis}];
 If[modSet == 1, k1 = k1 + 1], {k, rw}]
If[modSet == 1, mp = Partition[modelPlots, {mPerAxis + 1}],
  mp = Partition[modelPlots, mPerAxis]];
If[modSet == 1, Do[mp〚i, 6〛 = Show[Take[mp〚i〛, 5]], {i, rw}]];

If[modSet == 1, lb = "Axis "];
If[modSet == 2, lb = "Chord "];
If[modSet == 3, lb = "Row "];
Do[mp〚i, j〛 = Labeled[mp〚i, j〛,
   StringJoin[lb, ToString[i], ToString[", Model "], ToString[j]],
   Bottom, LabelStyle → Directive[FontSize → 12, FontFamily → "Arial"]],
 {i, rw}, {j, mPerAxis}]
If[modSet == 1, Do[mp〚i, 6〛 = Labeled[mp〚i, 6〛,
    StringJoin[lb, ToString[i], ToString[", Strobe Plot"]], Bottom,
    LabelStyle → Directive[FontSize → 12, FontFamily → "Arial"]], {i, rw}]]

If[modSet == 1, title = "Along-Axis Model Table"];
If[modSet == 2, title = "Group-Difference Model Table"];
If[modSet == 3, title = "Subspace Model Table"];
gdPlt = Labeled[GraphicsGrid[mp, Frame → All, ImageSize → grdSz], title,
  Top, LabelStyle → Directive[FontSize → 22, Bold, FontFamily → "Arial"]]
```

Specify plot orientation.

In[•]:=
```
PopupMenu[Dynamic[flip], {0 → "No Change", 1 → "Reverse row order",
   2 → "Flip across y-axis", 3 → "Flip across x-axis", 4 → "Reverse & Flip Both"}]
flip =
   0;
```

Out[•]= No Change

Change plot orientation (if necessary).

In[•]:=
```
If[flip == 1 || flip == 4,
 Do[
  If[row1 == 1, k = 1, k = mPerAxis * (row1 - 1) + 1];
  k2 = k + (mPerAxis - 1);
  rMat = Reverse[Take[models, {k, k2}]];
  kount = k - 1;
  Do[
   kount = kount + 1;
   models[[kount]] = rMat[[i]], {i, mPerAxis}], {row1, axes}]]
If[flip == 2 || flip == 4,
 Do[models[[k, i, 2]] = models[[k, i, 2]] * (-1.0), {k, nModels}, {i, m / 2}]]
If[flip == 3 || flip == 4,
 Do[models[[k, i, 1]] = models[[k, i, 1]] * (-1.0), {k, nModels}, {i, m / 2}]]
```

Export current grid plot.

In[•]:=
```
filenameout = SystemDialogInput["FileSave"];
Export[filenameout, gdPlt, "TIFF", ImageResolution → 150]
```

Out[•]= /Users/n.macleod/Desktop/Drangonflies (Final)/Data & Results/GM/ Hindwings/CVA Results/Water Body Groups/Along-Axis Shape Models (Grid).tif

Calculate vector comparison plots (if necessary).

Obtain interactive control parameters.

In[•]:=

```
Panel[
 Labeled[Column[{Row[{Panel[Labeled[InputField[Dynamic[bAxis], FieldSize → 7],
        "Base Model Axis/Row Number", Top, LabelStyle →
         Directive[FontSize → 12, Bold, FontFamily → "Arial"]]], "  ",
      Panel[Labeled[InputField[Dynamic[bModel], FieldSize → 7],
        "Base Model Number", Top,
        LabelStyle → Directive[FontSize → 12, Bold, FontFamily → "Arial"]]]}],
    Row[{Panel[Labeled[InputField[Dynamic[cAxis], FieldSize → 7],
        "Comparison Model Axis/Row Number", Top, LabelStyle →
         Directive[FontSize → 12, Bold, FontFamily → "Arial"]]], "  ",
      Panel[Labeled[InputField[Dynamic[cModel], FieldSize → 7],
        "Comparison Model Number", Top,
        LabelStyle → Directive[FontSize → 12, Bold, FontFamily → "Arial"]]]}],
    Row[{Panel[Labeled[InputField[Dynamic[vScale], FieldSize → 7],
        "Vector Scaling Value.", Top,
        LabelStyle → Directive[FontSize → 12, Bold, FontFamily → "Arial"]]],
      "  ", Panel[Labeled[InputField[Dynamic[visf], FieldSize → 7],
        "Icon Size Value.", Top, LabelStyle →
         Directive[FontSize → 12, Bold, FontFamily → "Arial"]]], "  ",
      Panel[Labeled[InputField[Dynamic[vpltSz], FieldSize → 7],
        "Plot Size Value.", Top,
        LabelStyle → Directive[FontSize → 12, Bold, FontFamily → "Arial"]]]}]},
   Center], "Vector Comparison Plot Control Parameters.", Top,
  LabelStyle → Directive[FontSize → 18, Bold, FontFamily → "Arial"]]]
bAxis =
 1;
bModel = 1;
cAxis = 1;
cModel = 5; visf = 0.035; vpltSz = 500; vScale = 2.0;
```

Out[•]=

Vector Comparison Plot Control Parameters.

Base Model Axis/Row Number	Base Model Number
bAxis	bModel

Comparison Model Axis/Row Number	Comparison Model Number
cAxis	cModel

Vector Scaling Value.	Icon Size Value.	Plot Size Value.
vScale	visf	vpltSz

Calculate & display vector plot.

In[•]:=
```
modGrid = Partition[models, mPerAxis];
{m3, m4} = Dimensions[vectors];
dLines = Table[" ", {m4 / 2}];
icon2 = Graphics[{EdgeForm[{Thickness[0.0025], Black}],
    Hue[1 - ((bModel - 1) * 0.1)], Disk[{0, 0}, Scaled[visf]]}];
end1 = ListPlot[modGrid⟦bAxis, bModel⟧, Joined → False,
   PlotStyle → {Black, Thickness[(maxi - mini) / 100]},
   AspectRatio → 1, Frame → False, Axes → False, ImageSize → vpltSz,
   FrameLabel → {"Axis1", "Axis2"}, PlotRange → {{mini, maxi}, {mini, maxi}},
   PlotRangePadding → Scaled[1.00], PlotMarkers → icon2];
Do[
  origin = modGrid⟦bAxis, bModel, i⟧;
  dif = modGrid⟦bAxis, bModel, i⟧ - modGrid⟦cAxis, cModel, i⟧;
  sDif = dif / (1 / vScale);
  extrap = modGrid⟦cAxis, cModel, i⟧ - sDif;
  dLines⟦i⟧ = Graphics[{Arrowheads[0.03], Arrow[{origin, extrap}]},
    ImageSize → vpltSz], {i, m4 / 2}];

If[modSet == 1,
  lab = StringJoin["Base: Axis ", ToString[bAxis], ", Model ", ToString[bModel],
     "; Comparison: Axis ", ToString[cAxis], ", Model ", ToString[cModel]];,
  lab = StringJoin["Base: Row ", ToString[bAxis], ", Model  ", ToString[bModel],
    "; Comparison: Row ", ToString[cAxis], ", Model ", ToString[cModel]]];
vecPlt = Labeled[Labeled[Show[dLines, end1], lab, Top, LabelStyle →
    Directive[FontSize → 14, Bold, FontFamily → "Arial"]], "Vector Plot",
  Top, LabelStyle → Directive[FontSize → 18, Bold, FontFamily → "Arial"]]
```

Export vector plot.

In[•]:=
```
filenameout = SystemDialogInput["FileSave"];
Export[filenameout, vecPlt, "TIFF", ImageResolution → 150]
```

Out[•]=
```
/Users/n.macleod/Desktop/Drangonflies (Final)/Data & Results/GM/ Hindwings/CVA
  Results/Water Body Groups/Along-Axis Shape Models (Vector Plot).tif
```
