# Supplementary material for: What you sample is what you get: ecomorphological variation in Trithemis (Odonata, Libellulidae) dragonfly wings reconsidered
Source: BMC Ecol Evol. 2022 Apr 11;22:43. doi: 10.1186/s12862-022-01978-y (PMC8996507; doi:10.1186/s12862-022-01978-y)
Supplement: Supplementary file 3 — Additional file 3: Software Archive. [file 12862_2022_1978_MOESM3_ESM.zip › Additional Files 3/Software Archive/Lines (vers. 1.11).pdf]

Lines

This program accepts a data matrix in standard format and performs a linear regression analysis on the data columns,
and allows the result to be viewed as 2D scatterplots. Regression models include the following:

Least Squares
Standardized (Reduced) Major Axis
Major Axis

Full support is provided for tracking groups that have been identified in the datafile.

Author: N. MacLeod
Version: 1.10
Date: 20 July 2020
Reference: Warton et al. (2006), MacLeod (in press)

Initialize libraries.

```
In[∘]:= << ComputationalGeometry`
```

Read in data file & partition into datasets.

```
In[•]:= filenamein = SystemDialogInput["FileOpen"];
x1 = Import[filenamein, "CSV"];
filenamein

{n1, m1} = Dimensions[x1];

varNames = Flatten[Take[x1, 1]];
x2 = Drop[x1, 1];
varNames = Drop[varNames, 1];
varNames = Drop[varNames, 1];

objNames = Flatten[Take[x2, n1 - 1, 1]];
x2 = Drop[x2, 0, 1];

Group = Flatten[Take[x2, n1 - 1, 1]];
x2 = Drop[x2, 0, 1];

{n2, m2} = Dimensions[x2];
Print["No. of objects: ", n2]
Print["No. of variables: ", m2]
m3 = m2;

varNames;
objNames;
Group;
x2;
```

```
Out[•]= /Users/n.macleod/Desktop/Drangonflies (Final)/Data & Results/GM/
  Hindwings/CVA Results/LS vs WB Comparison/Hindwings CV-1 Scores.csv

No. of objects: 276

No. of variables: 2
```

Specify 2D plot options.

You must run this code after you read in the data so it can pick up the proper variable names.

In[•]:=
```
Panel[
 Labeled[Column[{Row[{Panel[Labeled[PopupMenu[Dynamic[xAxisName], varNames],
       "Select variable to be plotted on x-Axis.", Top,
       LabelStyle → Directive[FontSize → 12, Bold, FontFamily → "Arial"]]],
     Panel[Labeled[PopupMenu[Dynamic[yAxisName], varNames],
       "Select variable to be plotted on y-Axis.", Top,
       LabelStyle → Directive[FontSize → 12, Bold, FontFamily → "Arial"]]]}],
   Row[{Panel[Labeled[PopupMenu[Dynamic[rModel],
        {1 → "Least Squares", 2 → "Standardized Major Axis", 3 → "Major Axis"}],
       "Enter regression model.", Top, LabelStyle →
        Directive[FontSize → 12, Bold, FontFamily → "Arial"]]]}],
   Row[{Panel[Labeled[PopupMenu[Dynamic[logTrans],
        {1 → "No Transform", 2 → "Log-Transform x", 3 → "Log-Transform y",
         4 → "Log-Transform x & y"}], "Log-Transform data?", Top,
       LabelStyle → Directive[FontSize → 12, Bold, FontFamily → "Arial"]]],
     Panel[Labeled[PopupMenu[Dynamic[axesType], {1 → "Arithmetic Axes",
         2 → "Log (x) - Linear (y) Axes", 3 → "Linear (x) - Log (y) Axes",
         4 → "Log-Log Axes", 5 → "Polar Axes"}], "Enter plot axis type.", Top,
       LabelStyle → Directive[FontSize → 12, Bold, FontFamily → "Arial"]]]}],
   Panel[Labeled[PopupMenu[Dynamic[pltAspect],
      {1 → "Golden Ratio Plot", 2 → "Square Plot (same axes)",
       3 → "Full Plot (actual axis scale differences)"}],
     "Enter plot aspect ratio type.", Top, LabelStyle →
      Directive[FontSize → 12, Bold, FontFamily → "Arial"]]],
   Row[{
     Panel[Labeled[InputField[Dynamic[pltSize], FieldSize → 10],
       "Enter plot size value.", Top,
       LabelStyle → Directive[FontSize → 12, Bold, FontFamily → "Arial"]]],
     Panel[Labeled[InputField[Dynamic[pltPad], FieldSize → 10],
       "Enter plot margin padding value.", Top,
       LabelStyle → Directive[FontSize → 12, Bold, FontFamily → "Arial"]]],
     Panel[Labeled[InputField[Dynamic[iconSize], FieldSize → 10],
       "Enter plot icon size value.", Top,
       LabelStyle → Directive[FontSize → 12, Bold, FontFamily → "Arial"]]]}]},
  Center], "Regression Options Panel", Top, LabelStyle →
  Directive[FontSize → 16, Bold, FontFamily → "Arial"]]]
pltSize = 500; iconSize = 0.02; pltPad = 0.2; xAxisName = varNames⟦1⟧;
yAxisName = varNames⟦2⟧; axesType = 1; pltAspect = 1;
```

Out[∘]=

Regression Options Panel

Select variable to be plotted on x–Axis.	Select variable to be plotted on y–Axis.
∨	∨

Enter regression model.
Least Squares ∨

Log–Transform data?	Enter plot axis type.
No Transform ∨	Arithmetic Axes ∨

Enter plot aspect ratio type.
Golden Ratio Plot ∨

Enter plot size value.	Enter plot margin padding value.	Enter plot icon size value.
pltSize	pltPad	iconSize

Calculate and plot regression

In[∘]:=

```
Do[If[xAxisName == varNames⟦j⟧, axis1 = j], {j, m3}]
Do[If[yAxisName == varNames⟦j⟧, axis2 = j], {j, m3}]
x2T = Transpose[x2];
If[logTrans == 1, xAxis = x2T⟦axis1⟧;
  yAxis = x2T⟦axis2⟧, xAxis = Log[10, x2T⟦axis1⟧];
  yAxis = Log[10, x2T⟦axis2⟧]];
points = Transpose[List[xAxis, yAxis]];
Clear[xAxis];
Clear[yAxis];
meanX = Mean[points⟦All, 1⟧];
meanY = Mean[points⟦All, 2⟧];
sumX = Sum[points⟦i, 1⟧, {i, n2}];
sumY = Sum[points⟦i, 2⟧, {i, n2}];
sumX2 = Sum[points⟦i, 1⟧^2, {i, n2}];
sumY2 = Sum[points⟦i, 2⟧^2, {i, n2}];
sumXY = Sum[points⟦i, 1⟧ * points⟦i, 2⟧, {i, n2}];
sumx2 = Sum[(meanX - points⟦i, 1⟧)^2, {i, n2}];
sumy2 = Sum[(meanY - points⟦i, 2⟧)^2, {i, n2}];
sumxy = sumXY - ((sumX * sumY) / n2);

If[rModel == 1,
  m = N[sumxy / sumx2]];
If[rModel == 2,
```

```
  stdevX = StandardDeviation[points[[All, 1]]];
  stdevY = StandardDeviation[points[[All, 2]]];
  m = stdevY / stdevX; 0.;
  If[sumxy < 0.0, m = m * (-1)];]
 If[rModel == 3,
  s2x = sumx2 / (n2 - 1);
  s2y = sumy2 / (n2 - 1);
  sxy = Sum[((meanX - points[[i, 1]]) * (meanY - points[[i, 2]])) / (n2 - 1), {i, n2}];
  Dval = Sqrt[(s2x + s2y)^2 - 4 ((s2x * s2y) - sxy^2)];
  λ1 = (s2x + s2y + Dval) / 2;
  m = sxy / (λ1 - s2y);]
 b = Chop[N[meanY - (m * meanX)], 10^-5];
 If[b == 0, b = 0.000];
 eqn = StringJoin[{"y = "}, ToString[PaddedForm[m, {4, 3}]],
    {"x + "}, ToString[PaddedForm[b, {4, 3}]]];
 Grid[{{eqn}}, BaseStyle → (FontFamily → "Arial")];

 If[pltAspect == 1, aRatio = 1 / N[GoldenRatio]];
 If[pltAspect == 2, aRatio = 1];
 If[pltAspect == 3, aRatio = Full];

 groupNames = Union[Group];
 numGroups = Length[groupNames];
 groupPosns = Table[Flatten[Position[Group, groupNames[[i]], 1]], {i, numGroups}];
 x2T = Transpose[x2];
 If[logTrans == 1, xAxis = x2T[[axis1]];
   yAxis = x2T[[axis2]], xAxis = Log[10, x2T[[axis1]]];
   yAxis = Log[10, x2T[[axis2]]]];
 tmpPoints = Transpose[List[xAxis, yAxis]];
 pltPoints = Table[tmpPoints[[groupPosns[[j]]]], {j, numGroups}];
 If[logTrans == 1, lab1 = varNames[[axis1]]; lab2 = varNames[[axis2]]];
 If[logTrans == 2, StringJoin["Log ", varNames[[axis1]]];
   lab2 = varNames[[axis2]]];
 If[logTrans == 3, lab1 = varNames[[axis1]];
   lab2 = StringJoin["Log ", varNames[[axis2]]]];
 If[logTrans == 4, lab1 = StringJoin["Log ", varNames[[axis1]]];
   lab2 = StringJoin["Log ", varNames[[axis2]]]];

 minx = Min[tmpPoints[[All, 1]]];
 maxx = Max[tmpPoints[[All, 1]]];
 miny = Min[tmpPoints[[All, 2]]];
 maxy = Max[tmpPoints[[All, 2]]];
 sf = 0.10;
 xsf = (maxx - minx) * sf;
 ysf = (maxy - miny) * sf;
 minx = minx - xsf;
```

```
maxx = maxx + xsf;
miny = miny - ysf;
maxy = maxy + ysf;

If[pltAspect == 1, xPlotLow = minx;
  xPlotHi = maxx;
  yPlotLow = miny;
  yPlotHi = maxy];
If[pltAspect == 2, If[minx > miny, xPlotLow = miny;
   yPlotLow = miny, xPlotLow = minx, yPlotLow = minx ]];
If[pltAspect == 2, If[maxx < maxy, xPlotHi = maxy;
   yPlotHi = maxy, xPlotHi = maxx;
   yPlotHi = maxx]];
If[pltAspect == 3, xPlotLow = minx;
  xPlotHi = maxx;
  yPlotLow = miny;
  yPlotHi = maxy];

iconList = Flatten[Table[
    {Graphics[{EdgeForm[{Thin, Black}], Hue[N[((numGroups + 1) - j) / numGroups]],
       Disk[{0, 0}, Scaled[iconSize]]}]}, {j, numGroups}]];

If[axesType == 1,
  ptPlot = ListPlot[pltPoints, AspectRatio → aRatio, Frame → True, Joined → False,
    Axes → False, PlotRange → {{xPlotLow, xPlotHi}, {yPlotLow, yPlotHi}},
    PlotRangePadding → Scaled[pltPad], Ticks → Automatic,
    FrameLabel → {lab1, lab2}, PlotMarkers → iconList, ImageSize → pltSize,
    LabelStyle → Directive[FontSize → 14, Black, FontFamily → "Arial"]]];
If[axesType == 2, ptPlot = Labeled[ListLogLinearPlot[pltPoints,
     AspectRatio → aRatio, Frame → True, Joined → False, Axes → False, PlotRange →
      Automatic, PlotRange → {{xPlotLow, xPlotHi}, {yPlotLow, yPlotHi}},
     PlotRangePadding → Scaled[pltPad], Ticks → Automatic,
     FrameLabel → {lab1, lab2}, PlotMarkers → iconList, ImageSize → pltSize,
     LabelStyle → Directive[FontSize → 14, Black, FontFamily → "Arial"]],
    "              Data Plot", Top,
    LabelStyle → Directive[FontSize → 18, Black, FontFamily → "Arial"]]];
If[axesType == 3, ptPlot = Labeled[ListLogPlot[pltPoints, AspectRatio → aRatio,
     Frame → True, Joined → False, Axes → False, PlotRange → Automatic,
     PlotRange → {{xPlotLow, xPlotHi}, {yPlotLow, yPlotHi}},
     PlotRangePadding → Scaled[pltPad], Ticks → Automatic,
     FrameLabel → {lab1, lab2}, PlotMarkers → iconList, ImageSize → pltSize,
     LabelStyle → Directive[FontSize → 14, Black, FontFamily → "Arial"]],
    "              Data Plot", Top,
    LabelStyle → Directive[FontSize → 18, Black, FontFamily → "Arial"]]];
If[axesType == 4, ptPlot = Labeled[ListLogLogPlot[pltPoints, AspectRatio → aRatio,
     Frame → True, Joined → False, Axes → False, PlotRange → Automatic,
     PlotRange → {{xPlotLow, xPlotHi}, {yPlotLow, yPlotHi}},
```

```
     PlotRangePadding → Scaled[pltPad], Ticks → Automatic,
     FrameLabel → {lab1, lab2}, PlotMarkers → iconList, ImageSize → pltSize,
     LabelStyle → Directive[FontSize → 14, Black, FontFamily → "Arial"]],
    "              Data Plot", Top,
    LabelStyle → Directive[FontSize → 18, Black, FontFamily → "Arial"]]];
If[axesType == 5, ptPlot = Labeled[ListPolarPlot[pltPoints, AspectRatio → aRatio,
     Frame → True, Joined → False, Axes → False, PlotRange → Automatic,
     PlotRange → {{xPlotLow, xPlotHi}, {yPlotLow, yPlotHi}},
     PlotRangePadding → Scaled[pltPad], Ticks → Automatic,
     FrameLabel → {lab1, lab2}, PlotMarkers → iconList, ImageSize → pltSize,
     LabelStyle → Directive[FontSize → 14, Black, FontFamily → "Arial"]],
    "              Data Plot", Top,
    LabelStyle → Directive[FontSize → 18, Black, FontFamily → "Arial"]]];
g1 = Grid[Table[
    {Graphics[{EdgeForm[{Thin, Black}], Hue[N[((numGroups + 1) - j) / numGroups]],
       Disk[]}]}, {j, numGroups}], Frame → False, ItemSize → 1.075];
g2 = Grid[Partition[groupNames, 1], Alignment → Left,
   BaseStyle → {FontFamily → "Arial", Italic}];
p2 = Labeled[Text[Grid[{{g1, g2}}, Alignment → Top, Frame → True]], "Legend",
   Top, LabelStyle → Directive[FontSize → 18, Black, FontFamily → "Arial"]];

lnPlot = Plot[(m * x) + b, {x, minx, maxx},
   PlotStyle → {Black, Thickness[0.0025]}, AspectRatio → aRatio, Frame → True,
   Axes → False, PlotRange → {{xPlotLow, xPlotHi}, {yPlotLow, yPlotHi}},
   PlotRangePadding → Scaled[pltPad], Ticks → Automatic,
   FrameLabel → {lab1, lab2}, ImageSize → pltSize,
   LabelStyle → Directive[FontSize → 14, Black, FontFamily → "Arial"]];

pos = Flatten[Table[{Min[points⟦All, 1⟧] + 0, Max[points⟦All, 2⟧] + 0}, {1}]];
eqnTable = Text[eqn, pos, {-1, 0}];
eqnPlot = Graphics[eqnTable,
   BaseStyle → {Directive[FontSize → 11, FontFamily → "Arial"]}];

If[rModel == 1, pltLabel = "          Least Squares Regression Plot"];
If[rModel == 2, pltLabel = "      Standardized Major Axis Regression Plot"];
If[rModel == 3, pltLabel = "             Major Axis Regression Plot"];

p1 = Labeled[Show[{lnPlot, ptPlot, eqnPlot},
    BaseStyle → {Directive[FontSize → 11, FontFamily → "Arial"]},
    ImageSize → pltSize], pltLabel, Top,
   LabelStyle → Directive[FontSize → 18, Black, FontFamily → "Arial"]];

Plt2D = Grid[{{p1, p2}}, BaselinePosition → Top, Alignment → Top]
```

Export current plot.

In[∘]:=
```
filenameout = SystemDialogInput["FileSave"];
Export[filenameout, Plt2D, "TIFF", ImageResolution → 150]
```

Out[∘]=
```
/Users/n.macleod/Desktop/Drangonflies (Final)/Data & Results/GM/
  Hindwings/CVA Results/LS vs WB Comparison/SMS Regression Plot.tif
```

Label plotted points

```
In[•]:= If[axesType == 1,
   pn1 = ListPlot[pltPoints, AspectRatio → aRatio, Frame → True, Axes → False,
     Joined → False, PlotRange → {{xPlotLow, xPlotHi}, {yPlotLow, yPlotHi}},
     PlotRangePadding → Scaled[pltPad], Ticks → Automatic,
     FrameLabel → {lab1, lab2}, PlotMarkers → iconList, ImageSize → pltSize,
     LabelStyle → Directive[FontSize → 14, FontFamily → "Arial"]]];
 If[axesType == 2, pn1 = ListLogLinearPlot[pltPoints, AspectRatio → aRatio,
     Frame → True, Axes → False, Joined → False,
     PlotRangePadding → Scaled[pltPad], Ticks → Automatic,
     FrameLabel → {lab1, lab2}, PlotMarkers → iconList, ImageSize → pltSize,
     LabelStyle → Directive[FontSize → 14, FontFamily → "Arial"]]];
 If[axesType == 3, pn1 = ListLogPlot[pltPoints, AspectRatio → aRatio,
     Frame → True, Axes → False, Joined → False,
     PlotRangePadding → Scaled[pltPad], Ticks → Automatic,
     FrameLabel → {lab1, lab2}, PlotMarkers → iconList, ImageSize → pltSize,
     LabelStyle → Directive[FontSize → 14, FontFamily → "Arial"]]];
 If[axesType == 4, pn1 = ListLogLogPlot[pltPoints, AspectRatio → aRatio,
     Frame → True, Axes → False, Joined → False,
     PlotRangePadding → Scaled[pltPad], Ticks → Automatic,
     FrameLabel → {lab1, lab2}, PlotMarkers → iconList, ImageSize → pltSize,
     LabelStyle → Directive[FontSize → 14, FontFamily → "Arial"]]];
 If[axesType == 5, pn1 = ListPolarPlot[pltPoints, AspectRatio → aRatio,
     Frame → True, Axes → False, Joined → False,
     PlotRangePadding → Scaled[pltPad], Ticks → Automatic,
     FrameLabel → {lab1, lab2}, PlotMarkers → iconList, ImageSize → pltSize,
     LabelStyle → Directive[FontSize → 14, FontFamily → "Arial"]]];

 namePoints = tmpPoints;
 tempPointsT = Transpose[tmpPoints];
 mxY = Max[tempPointsT[[2]]];
 mnY = Min[tempPointsT[[2]]];
 incY = N[(mxY - mnY) / 20];
 Do[namePoints[[i, 2]] = tmpPoints[[i, 2]] - incY, {i, n2}]
 namePoints;
 nPointsTable = Table[{Text[objNames[[i]], namePoints[[i]], {-1, 0}]}, {i, n2}];
 pn2 = Graphics[nPointsTable, Frame → True, AspectRatio → aRatio , Axes → False,
    FrameLabel → {lab1, lab2}, PlotRangePadding → Scaled[pltPad], BaseStyle →
     Directive[FontSize → 14, FontFamily → "Arial"], ImageSize → pltSize];

 p1 = Labeled[Show[lnPlot, pn1, pn2, BaseStyle → {FontFamily → "Arial"}], pltLabel,
    Top, LabelStyle → Directive[FontSize → 18, FontFamily → "Arial"]];

 Plt2D = Grid[{{p1, p2}}, BaselinePosition → Top, Alignment → Top]
```

Export current plot.

```
In[•]:= filenameout = SystemDialogInput["FileSave"];
        Export[filenameout, Plt2D, "TIFF", ImageResolution → 150]

Out[•]= /Users/n.macleod/Projects/Storage/MacLeod/Nanjing
          University/NJU Courses/2020/Statistics & Data Analysis
          Course/Lectures/Week 5 (Spatial Data)/Coast of Britian Plot.tif
```

Calculate regression/trend line statistics.

```
In[•]:= estXY = Table[0.0, {n2}, {2}];
        resid = Table[0.0, {n2}];

        If[rModel == 1,
          Do[estXY⟦i, 1⟧ = points⟦i, 1⟧;
           estXY⟦i, 2⟧ = (m * points⟦i, 1⟧) + b, {i, n2}]];

        If[rModel == 2,
         Do[
           xc1 = points⟦i, 1⟧;
           yc1 = (m * xc1) + b;
           yc2 = points⟦i, 2⟧;
           xc2 = (yc2 - b) / m;
           d1 = EuclideanDistance[points⟦i⟧, {xc1, yc1}];
           xc3 = xc2;
           If[points⟦i, 2⟧ > (points⟦i, 1⟧ * m) + b, yc3 = yc2 - d1, yc3 = yc2 + d1];
           estXY⟦i, 1⟧ = points⟦i, 1⟧ - ((points⟦i, 1⟧ - xc3) / 2);
           estXY⟦i, 2⟧ = points⟦i, 2⟧ - ((points⟦i, 2⟧ - yc3) / 2);
           resid⟦i⟧ = EuclideanDistance[points⟦i⟧, estXY⟦i⟧];
           If[points⟦i, 2⟧ < (points⟦i, 1⟧ * m) + b,
            resid⟦i⟧ = resid⟦i⟧ * (-1.0)], {i, n2}];]

        If[rModel == 3,
         mFact = -1 / m;
         Do[
          b2 = points⟦i, 2⟧ - (points⟦i, 1⟧ * mFact);
          estXY⟦i, 1⟧ = (b2 - b) / (m - mFact);
          estXY⟦i, 2⟧ = (m * estXY⟦i, 1⟧) + b;
          resid⟦i⟧ = EuclideanDistance[points⟦i⟧, estXY⟦i⟧];
          If[points⟦i, 2⟧ < (points⟦i, 1⟧ * m) + b, resid⟦i⟧ = resid⟦i⟧ * (-1.0)], {i, n2}];]

        SSt = N[Sum[(points⟦i, 2⟧ - meanY)^2, {i, n2}]];
        SSr = N[Sum[(estXY⟦i, 2⟧ - meanY)^2, {i, n2}]];
        SSd = SSt - SSr;
        dofSSt = n2 - 1;
        dofSSr = 1;
        dofSSe = n2 - 2;
        MSr = SSr / dofSSr;
```

```
MSd = SSd / dofSSe;
F = MSr / MSd;

r2 = Chop[(SSr / SSt), 0.00001];
r2adj = Chop[(SSr / SSt) * (N[(n2 - 1) / (n2 - 2)]), 0.00001];
r = Chop[Sqrt[r2], 0.00001];
radj = Chop[Sqrt[r2adj], 0.00001];
resid = points⟦All, 2⟧ - estXY⟦All, 2⟧;

If[rModel == 1, label = "Regression ANOVA Table"];
If[rModel == 2, label = "Standardized Major Axis Trend Line ANOVA Table"];
If[rModel == 3, label = "Major Axis Trend Line ANOVA Table"];
statTable = Table[" ", {i, 4}, {j, 5}];
statTable⟦1, 1⟧ = "Source of Variation";
statTable⟦1, 2⟧ = "Sum of Squares";
statTable⟦1, 3⟧ = "Degrees of Freedom";
statTable⟦1, 4⟧ = "Mean Squares";
statTable⟦1, 5⟧ = "F";
If[rModel == 1, statTable⟦2, 1⟧ = "Regression",
  statTable⟦2, 1⟧ = "Trend Line Residuals"];
If [rModel == 1, statTable⟦3, 1⟧ = "Deviation",
  statTable⟦3, 1⟧ = "Deviation Residuals"];
statTable⟦4, 1⟧ = "Total";
statTable⟦2, 2⟧ = PaddedForm[SSr, {4, 3}];
statTable⟦3, 2⟧ = PaddedForm[SSd, {4, 3}];
statTable⟦4, 2⟧ = PaddedForm[SSt, {4, 3}];
statTable⟦2, 3⟧ = dofSSr;
statTable⟦3, 3⟧ = dofSSe;
statTable⟦4, 3⟧ = dofSSt;
statTable⟦2, 4⟧ = PaddedForm[MSr, {4, 3}];
statTable⟦3, 4⟧ = PaddedForm[MSd, {4, 3}];
statTable⟦2, 5⟧ = PaddedForm[F, {6, 3}];
Labeled[Grid[statTable, BaseStyle → (FontFamily → "Arial"),
  Alignment → {{Left, Center, Center, Center, Center}}, Frame → True,
  Dividers → {{True, True, True, True, True}, {True, True}}], label,
 Top, LabelStyle → Directive[FontSize → 18, FontFamily → "Arial"]]

pf = Labeled[Plot[PDF[FRatioDistribution[dofSSr, dofSSe], x], {x, 0, F + 1},
   ImageSize → 400, PlotRange → All, Filling → Bottom, FillingStyle → Green,
   AxesLabel → {"F-Ratio", "Frequency"}, LabelStyle → (FontFamily → "Arial")],
  "F-Ratio Probability Density Function", Top,
  LabelStyle → (FontFamily → "Arial")]

probTable = Table[" ", {i, 4}, {j, 2}];
probTable⟦1, 1⟧ = "Observed F-value";
probTable⟦2, 1⟧ = "Degrees of Freedom: Num.";
probTable⟦3, 1⟧ = "Degrees of Freedom: Denom.";
```

```
probTable[[4, 1]] = "Probability (%)";
probTable[[1, 2]] = PaddedForm[F, {6, 3}];
probTable[[2, 2]] = dofSSr;
probTable[[3, 2]] = dofSSe;
pval = Chop[(1 - CDF[FRatioDistribution[dofSSr, dofSSe], F]) * 100, 0.00001];
probTable[[4, 2]] = PaddedForm[pval, {4, 3}];

tProb = Labeled[Grid[probTable, BaseStyle → (FontFamily → "Arial"),
     Alignment → {{Left, Right}}, Frame → True, Dividers →
      {{True, True}, {True, False, False, False, True}}], "Probability Table",
    Top, LabelStyle → Directive[FontSize → 14, FontFamily → "Arial"]];

CoD =
  StringJoin["Coef. of Dispersion (r^2) = ", ToString[PaddedForm[r2, {5, 4}]]];
MCC = StringJoin["Multiple Correlation Coef. (r) = ",
   ToString[PaddedForm[r, {5, 4}]]];
CoDadj = StringJoin["Adjusted Coef. of Dispersion (r^2_adj) = ",
   ToString[PaddedForm[r2adj, {5, 4}]]];
MCCadj = StringJoin["Adjusted Multiple Correlation Coef. (r_adj) = ",
   ToString[PaddedForm[radj, {5, 4}]]];

Needs["HypothesisTesting`"];
If[rModel == 1,
  s2x = Variance[points[[All, 1]]];
  s2y = Variance[points[[All, 2]]];
  r2xy = Correlation[points[[All, 1]], points[[All, 2]]]^2;
  s2b = (1 / (n2 - 2)) * (s2y / s2x) * (1 - r2xy);
  sb = √s2b;
  test = 0.0;
  Do[
   test = test + 0.001;
   critVal = CDF[StudentTDistribution[n2 - 2], test];
   If[critVal > 0.95, Break[]], {i, 10 000}];
  test = test - 0.001;
  mCIup = m + (sb * test);
  mCIlow = m - (sb * test);]
If[rModel == 2,
  test = 0.0;
  Do[
   test = test + 0.001;
   critVal = CDF[FRatioDistribution[1, n2 - 2], test];
   If[critVal > 0.95, Break[]], {i, 10 000}];
  r2xy = Correlation[points[[All, 1]], points[[All, 2]]]^2;
  bt1 = ((1 - r2xy) / (n2 - 2)) * test;
  mCIup = m * (√(bt1 + 1) + √bt1);
```

```
  mCIlow = m * (√(bt1 + 1) - √bt1);]
 If[rModel == 3,
  test = 0.0;
  Do[
   test = test + 0.001;
   critVal = CDF[FRatioDistribution[1, n2 - 2], test];
   If[critVal > 0.95, Break[]], {i, 10 000}];
  test = test - 0.001;
  s2xy = sxy * sxy;
  qt1 = N[1 / (n2 - 2)];
  qt2 = s2x * s2y - s2xy;
  qt3 = test;
  Q = qt1 * qt2 * qt3;
  dval2 = √((s2x + s2y)^2 - 4 ((s2x * s2y) - sxy^2) - 4 Q);
  λ1CI = (s2x + s2y + dval2) / 2;
  mCIup = (sxy + √Q) / (λ1CI - s2y);
  mCIlow = (sxy - √Q) / (λ1CI - s2y);]
 bCIlow = meanY - (mCIlow * meanX);
 bCIup = meanY - (mCIup * meanX);

 dspTable = Table[" ", {3}, {3}];
 dspTable[[1, 2]] = "Coef. of Dispersion";
 dspTable[[1, 3]] = "Multiple Correlation Coef.";
 dspTable[[2, 1]] = "Raw";
 dspTable[[3, 1]] = "Adjusted";
 dspTable[[2, 2]] = PaddedForm[r2, {4, 3}];
 dspTable[[3, 2]] = PaddedForm[r2adj, {4, 3}];
 dspTable[[2, 3]] = PaddedForm[r, {4, 3}];
 dspTable[[3, 3]] = PaddedForm[radj, {4, 3}];
 tableDSP = Labeled[Grid[dspTable,
     BaseStyle → (FontFamily → "Arial"), Alignment → Center, Frame → True,
     Dividers → {{True, True, True}, {True, True}}], "Dispersion Measures",
    Top, LabelStyle → Directive[FontSize → 14, FontFamily → "Arial"]];

 ciTable = Table[" ", {3}, {3}];
 ciTable[[1, 2]] = "Slope";
 ciTable[[1, 3]] = "y-intercept";
 ciTable[[2, 1]] = "Upper";
 ciTable[[3, 1]] = "Lower";
 ciTable[[2, 2]] = PaddedForm[mCIup, {4, 3}];
 ciTable[[3, 2]] = PaddedForm[mCIlow, {4, 3}];
 ciTable[[2, 3]] = PaddedForm[bCIup, {4, 3}];
 ciTable[[3, 3]] = PaddedForm[bCIlow, {4, 3}];
 tableCI = Labeled[Grid[ciTable, BaseStyle → (FontFamily → "Arial"),
     Alignment → {{Center}, {Left, Center, Center}}, Frame → True,
```

```
     Dividers → {{True, True, True}, {True, True}}], "Confidence Intervals",
    Top, LabelStyle → Directive[FontSize → 14, FontFamily → "Arial"]];
probSlope = StringJoin["Percent probability that slope (m) is not zero = ",
    ToString[PaddedForm[100.0 - pval, {4, 3}]]]];

tProb
Grid[{{tableDSP, tableCI}}]
Grid[{{probSlope}}, BaseStyle → (FontFamily → "Arial"), Alignment → Left]
```

Plot residual datapoints.

```
In[•]:= residPoints = Table[0.0, {n2}, {2}];
If[rModel == 1,
   residPoints = points;
   Do[residPoints⟦i, 2⟧ = residPoints⟦i, 2⟧ - estXY⟦i, 2⟧, {i, n2}]];
If[rModel == 2,
   rotMat = Table[" ", {2}, {2}];
   rotMat⟦1, 1⟧ = Cos[ArcTan[m]];
   rotMat⟦1, 2⟧ = (Sin[ArcTan[m]]) * (-1.0);
   rotMat⟦2, 1⟧ = Sin[ArcTan[m]];
   rotMat⟦2, 2⟧ = Cos[ArcTan[m]];
   rotPoints = estXY;
   Do[rotPoints⟦i⟧ = estXY⟦i⟧.rotMat, {i, n2}];
   {meanXrot, meanYrot} = Mean[rotPoints];
   Do[
    residPoints⟦i, 1⟧ = rotPoints⟦i, 1⟧ - meanXrot;
    residPoints⟦i, 2⟧ = resid⟦i⟧, {i, n2}]];
If[rModel == 3,
   rotMat = Table[" ", {2}, {2}];
   rotMat⟦1, 1⟧ = Cos[ArcTan[m]];
   rotMat⟦1, 2⟧ = (Sin[ArcTan[m]]) * (-1.0);
   rotMat⟦2, 1⟧ = Sin[ArcTan[m]];
   rotMat⟦2, 2⟧ = Cos[ArcTan[m]];
   rotPoints = estXY;
   Do[rotPoints⟦i⟧ = points⟦i⟧.rotMat, {i, n2}];
   {meanXrot, meanYrot} = Mean[rotPoints];
   Do[
    residPoints⟦i, 1⟧ = rotPoints⟦i, 1⟧ - meanXrot;
    residPoints⟦i, 2⟧ = rotPoints⟦i, 2⟧ - meanYrot, {i, n2}]];

sdResid = StandardDeviation[residPoints⟦All, 2⟧];
Do[residPoints⟦i, 2⟧ = residPoints⟦i, 2⟧ / sdResid, {i, n2}];
residMeanX = Mean[residPoints⟦All, 1⟧];
residMeanY = Mean[residPoints⟦All, 2⟧];
residSumY = Sum[residPoints⟦i, 2⟧, {i, n2}];
```

```
residSumY2 = Sum[(residPoints[[i, 2]])^2, {i, n2}];
residSumXY = Sum[residPoints[[i, 1]] * residPoints[[i, 2]], {i, n2}];
residSumy2 = Sum[(residMeanY - residPoints[[i, 2]])^2, {i, n2}];
residSumxy = residSumXY - ((sumX * residSumY) / n2);
residm = residSumxy / sumx2;
residb = residMeanY - (residm * residMeanX);
resideqn = StringJoin[{"y = "}, ToString[PaddedForm[Chop[residm], {4, 3}]],
   {"x + "}, ToString[PaddedForm[Chop[residb], {4, 3}]]];
Grid[{{resideqn}}, BaseStyle → (FontFamily → "Arial")];

groupNames = Union[Group];
numGroups = Length[groupNames];
groupPosns = Table[Flatten[Position[Group, groupNames[[i]], 1]], {i, numGroups}];

x2T = Transpose[residPoints];
ax1 = x2T[[1]];
ax2 = x2T[[2]];
tmpPoints = Transpose[List[ax1, ax2]];
pltPoints = Table[tmpPoints[[groupPosns[[j]]]], {j, numGroups}];
minrotx = Min[tmpPoints[[All, 1]]];
maxrotx = Max[tmpPoints[[All, 1]]];
sfr = (maxrotx - minrotx) * 0.1;
minrotx = minrotx - sfr;
maxrotx = maxrotx + sfr;

If[rModel == 1, lab1 = varNames[[axis1]], lab1 = "Trend Model Axis"];
lab2 = "Standarized Residual Deviations";

ptPlot = ListPlot[pltPoints, AspectRatio → 1 / GoldenRatio, Frame → True,
   Axes → False, PlotRangePadding → Scaled[pltPad], Ticks → Automatic,
   FrameLabel → {lab1, lab2}, PlotMarkers → iconList, ImageSize → pltSize,
   LabelStyle → Directive[FontSize → 14, Black, FontFamily → "Arial"]];

lnPlot = Plot[(residm * x) + residb, {x, minrotx, maxrotx},
   PlotStyle → {Black, Thickness[0.0025]}, AspectRatio → 1 / GoldenRatio,
   Frame → True, Axes → False, PlotRangePadding → Scaled[pltPad],
   FrameLabel → {lab1, lab2}, ImageSize → pltSize,
   LabelStyle → Directive[FontSize → 14, FontFamily → "Arial"]];

pos =
  Flatten[Table[{Min[tmpPoints[[All, 1]]] + 0, Max[tmpPoints[[All, 2]]] + 0}, {1}]];
eqnTable = Text[resideqn, pos, {-2, 0}];
eqnPlot = Graphics[eqnTable, BaseStyle → (FontFamily → "Arial")];

If[rModel == 1, pltLabel = "         OLS Residual Regression Plot"];
If[rModel == 2, pltLabel = "         SMA Residual Regression Plot"];
If[rModel == 3, pltLabel = "          MA Residual Regression Plot"];
```

```
p1 = Labeled[
    Show[ptPlot, lnPlot, ptPlot, eqnPlot, BaseStyle → (FontFamily → "Arial")],
    pltLabel, Top, LabelStyle → Directive[FontSize → 18, FontFamily → "Arial"]];

resPlot = Grid[{{p1, p2}}, BaselinePosition → Top, Alignment → Top]
```

Export the residual plot.

In[∘]:=
```
filenameout = SystemDialogInput["FileSave"];
Export[filenameout, resPlot, "TIFF", ImageResolution → 150]
```

Out[∘]=
```
/Users/n.macleod/Desktop/Drangonflies (Final)/Data & Results/GM/ Hindwings/CVA
  Results/LS vs WB Comparison/SMS Regression Residuals Plot.tif
```

Perform and print residual error analysis

In[•]:=
```
residestY = Table[0.0, {n2}];
Do[residestY[[i]] = (residm * residPoints[[i, 1]]) + residb, {i, n2}];
residSSt = Sum[(residestY[[i]] - meanY)^2, {i, n2}];
residSSr = Sum[(residMeanY - residestY[[i]])^2, {i, n2}];
residSSd = residSSt - residSSr;
residMSr = residSSr / 1;
residMSd = residSSd / (n2 - 2);
residF = residMSr / residMSd;

residprob = (1 - CDF[FRatioDistribution[1, dofSSe], residF]) * 100;

statTable[[2, 2]] = PaddedForm[Chop[residSSr], {4, 3}];
statTable[[3, 2]] = PaddedForm[Chop[residSSd], {4, 3}];
statTable[[4, 2]] = PaddedForm[Chop[residSSt], {4, 3}];
statTable[[2, 3]] = 1;
statTable[[3, 3]] = n2 - 2;
statTable[[4, 3]] = n2 - 1;
statTable[[2, 4]] = PaddedForm[Chop[residMSr], {4, 3}];
statTable[[3, 4]] = PaddedForm[Chop[residMSd], {4, 3}];
statTable[[2, 5]] = PaddedForm[Chop[residF], {4, 3}];

Labeled[Grid[statTable, BaseStyle → (FontFamily → "Arial"),
   Alignment → {{Left, Center, Center, Center, Center}}, Frame → True,
   Dividers → {{True, True, True, True, True}, {True, True}}],
  "Residual Regression ANOVA Table", Top,
  LabelStyle → Directive[FontSize → 18, FontFamily → "Arial"]]

rProb = StringJoin["Percent probability no linear relation exists: ",
    ToString[PaddedForm[Chop[residprob, 10^-5], {4, 3}]]];

Grid[{{rProb}}, BaseStyle → (FontFamily → "Arial"), Alignment → Left]
```
