# Supplementary material for: What you sample is what you get: ecomorphological variation in Trithemis (Odonata, Libellulidae) dragonfly wings reconsidered
Source: BMC Ecol Evol. 2022 Apr 11;22:43. doi: 10.1186/s12862-022-01978-y (PMC8996507; doi:10.1186/s12862-022-01978-y)
Supplement: Supplementary file 3 — Additional file 3: Software Archive. [file 12862_2022_1978_MOESM3_ESM.zip › Additional Files 3/Software Archive/Procrustes (GLS) Superposition (v. 5.8).pdf]

Procrustes (GLS) Superposition (2 D or 3 D)

Author : N. MacLeod

Version : 5.7

Date : 3 Octiber 2015

Reference : Rohlf and Slice (1990)

Initialize libraries.

In[∘]:=
```
<< ComputationalGeometry`
```

Enter control parameters.

In[•]:=
```
Panel[
 Labeled[Column[{Row[{Panel[Labeled[InputField[Dynamic[kg], FieldSize → 10],
        "Enter no. of objects.", Top, LabelStyle →
         Directive[FontSize → 12, Bold, FontFamily → "Arial"]]], "  ",
      Panel[Labeled[InputField[Dynamic[m], FieldSize → 10],
        "Enter no. of variables.", Top, LabelStyle →
         Directive[FontSize → 12, Bold, FontFamily → "Arial"]]], "  ",
      Panel[Labeled[InputField[Dynamic[h1], FieldSize → 10],
        "Enter no. of header rows (tps files only).", Top,
        LabelStyle → Directive[FontSize → 12, Bold, FontFamily → "Arial"]]]}],
    Row[{Panel[Labeled[RadioButtonBar[Dynamic[fnf], {1 → "Exus_albus",
          2 → "Exus  albus"}, LabelStyle → (FontFamily → "Arial")],
        "Select object name format.", Top, LabelStyle →
         Directive[FontSize → 12, Bold, FontFamily → "Arial"]]], "  ",
      Panel[Labeled[RadioButtonBar[Dynamic[fne], {1 → "None",
          2 → ".xxx", 3 → ".xxxx"}, LabelStyle → (FontFamily → "Arial")],
        "Select object name extension format.", Top,
        LabelStyle → Directive[FontSize → 12, Bold, FontFamily → "Arial"]]]}],
    Row[{Panel[Labeled[RadioButtonBar[Dynamic[s1], {1 → "Yes", 2 → "No"},
         LabelStyle → (FontFamily → "Arial")],
        "Is a scale present? (tps files only)", Top, LabelStyle →
         Directive[FontSize → 12, Bold, FontFamily → "Arial"]]], "  ",
      Panel[Labeled[PopupMenu[Dynamic[fileType], {1 → ".tps-outline datafile",
          2 → ".tps-landmark datafile", 3 → ".dat datafile"}],
        "Select outline format.", Top, LabelStyle →
         Directive[FontSize → 12, Bold, FontFamily → "Arial"]]]}]},
   Center], "Input Options Panel", Top, LabelStyle →
   Directive[FontSize → 16,
    Bold, FontFamily → "Arial"]]]
kg = 24; m = 2; fnf = 2; fne = 2; s1 = 2; fileType = 3; h1 = 3;
```

Out[•]=

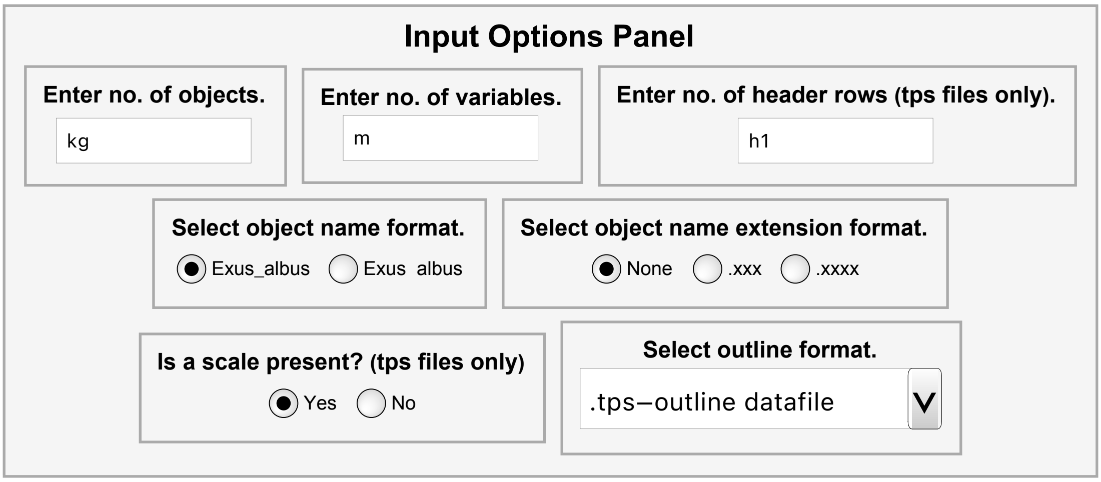

Read in dataset.

In[∘]:=
```
filenamein = SystemDialogInput["FileOpen"]
x1 = Import[filenamein, "Table"];
```

Out[∘]= /Users/n.macleod/Desktop/Landmarks.dat

Process datasets

In[∘]:=
```
If [fileType == 1,
 {ndf} = Dimensions[x1];
 ntot = ndf / kg;
 If[s1 == 1, n = ntot - h1 - 2, n = ntot - h1 - 1];
 x1Coords = Table[0, {n}, {m}, {kg}];
 objNames = Table[0, {kg}];
 Do[
  knt = k - 1;
  iup = (ntot * knt) + 1;
  ilow = ntot * (knt + 1);
  tmp1 = Take[x1, {iup, ilow}, All];
  tmp2 = Drop[tmp1, h1];
  tmp3 = Take[tmp2, n];
  If[s1 == 1,
   scl = First[ToExpression[StringDrop[Flatten[Take[tmp2, -1]], 6]]];
   tmp4 = Drop[tmp2, -1], scl = 1; tmp4 = tmp2];
  nme = Flatten[Take[tmp4, -1]];
  If[fnf == 2,
   If[fne == 1,
    nme = StringJoin["Specimen ", k]];
   If[fne == 2,
    nme = StringDrop[StringDrop[StringJoin[nme[[1]], " ", nme[[2]]], -4], 6]];
   If[fne == 3,
    nme = StringDrop[StringDrop[StringJoin[nme[[1]], " ", nme[[2]]], -5], 6]]];
  objNames[[k]] = nme;
  tmp5 = Drop[tmp4, -1];
  If[s1 == 1, Do[x1Coords[[i, j, k]] = tmp5[[i, j]] * scl, {i, n}, {j, m}],
   Do[x1Coords[[i, j, k]] = tmp5[[i, j]], {i, n}, {j, m}]], {k, kg}]]

If [fileType == 2,
 {ndf} = Dimensions[x1];
 ntot = ndf / kg;
 If[s1 == 1, f1 = 2, f1 = 1];
 n = ntot - h1 - f1;
 x1Coords = Table[0, {n}, {m}, {kg}];
 objNames = Table[" ", {kg}];

 Do[
  knt = k - 1;
  iup = (ntot * knt) + 1;
```

```
    ilow = ntot * (knt + 1);
    tmp1 = Take[x1, {iup, ilow}, All];
    tmp2 = Drop[tmp1, 1];
    If[s1 == 1, scl = First[ToExpression[StringDrop[Flatten[Take[tmp2, -1]], 6]]];
     tmp3 = Drop[tmp2, -1], scl = 1; tmp3 = tmp2];
    nme = Flatten[Take[tmp3, -1]];
    nme = StringDrop[StringDrop[StringJoin[nme⟦1⟧, " ", nme⟦2⟧], -4], 6];
    objNames⟦k⟧ = nme;
    Do[x1Coords⟦i, j, k⟧ = tmp3⟦i, j⟧ * scl, {i, n}, {j, m}], {k, kg}]]

If [fileType == 3,
 If[Length[Dimensions[x1]] == 1,
  {ndf} = Dimensions[x1], {ndf, m} = Dimensions[x1]];
 ntot = ndf / kg;
 n = (ndf - kg) / kg;
 x1Coords = Table[0, {n}, {m}, {kg}];
 objNames = Table[" ", {kg}];
 Do[
  ilow = (n + 1) * k;
  iup = (ilow) - (n);
  b = Flatten[Take[x1, {iup}]];
  objNames⟦k⟧ = b;
  x2 = Take[x1, {iup + 1, ilow}];
  Do[x1Coords⟦i, j, k⟧ = x2⟦i, j⟧, {i, n}, {j, m}], {k, kg}]]

objNames = Partition[Flatten[objNames], 2];
Do[
 objNames⟦k⟧ = StringJoin[objNames⟦k, 1⟧, " ", ToString[objNames⟦k, 2⟧]], {k, kg}]
```

Choose alignment mode.

In[∘]:=
```
Panel[PopupMenu[Dynamic[alignMode],
  {1 → "With Size Standarization", 2 → "Without Size Standarization"}]]
alignMode =
  1;
```

Out[∘]= With Size Standarization

Perform first – pass superposition (reference is first object)
First : read raw dataset into 'a'
Second : translate and scale coordinate (calculating centroid size)
Third : perform rigid rotation
Then, perform second – pass superposition (reference is mean of previous pass)
Rotation loop iterates until the distance between successive mean landmark configurations falls below 0.0001 (= tol). Bar chart illustrates decay of mean landmark configuration distances over total iteration cycles.

In[∘]:=
```
a = Table[0.0, {n}, {m}, {kg}];
Do[a⟦i, j, k⟧ = x1Coords⟦i, j, k⟧, {i, n}, {j, m}, {k, kg}]
```

```
ref = Table[0.0, {i, n}, {j, m}];
comp = Table[0.0, {i, n}, {j, m}];
csize = Table[0.0, {k, kg}];
IM = IdentityMatrix[n];
PM = Table[1 / n, {i, n}, {j, n}];

Do[ref⟦i, j⟧ = a⟦i, j, 1⟧, {i, n}, {j, m}];
ref = (IM - PM).ref;
s1 = √(Tr[((IM - PM).ref.(Transpose[ref]).(IM - PM))]) ;
csize⟦1⟧ = N[s1];
If[alignMode == 1, ref = ref / s1];
Do[a⟦i, j, 1⟧ = ref⟦i, j⟧, {i, n}, {j, m}]
a;

Do[
 Do[comp⟦i, j⟧ = a⟦i, j, k⟧, {i, n}, {j, m}];
 comp = (IM - PM).comp;
 s2 = √(Tr[((IM - PM).comp.(Transpose[comp]).(IM - PM))]) ;
 csize⟦k⟧ = N[s2];
 If[alignMode == 1, comp = comp / s2];

 Rot = (Transpose[ref]).comp;
 {U, Sig, Vt} = SingularValueDecomposition[Rot];
 S = IdentityMatrix[m];
 H = Vt.S.(Transpose[U]);
 comp' = comp.H;
 Do[a⟦i, j, k⟧ = comp'⟦i, j⟧, {i, n}, {j, m}], {k, 2, kg}]
csize;
a;

park = ref;
tol = 0.001;
cycles = 10;
dist = Table[0.0, {i, cycles}];

Do[
 Do[
  sum = 0.0;
  Do[sum = sum + a⟦i, j, k⟧, {k, kg}];
  ref⟦i, j⟧ = sum / kg, {i, n}, {j, m}];
 ref;

 Do[
   Do[comp⟦i, j⟧ = a⟦i, j, k⟧, {i, n}, {j, m}];
   Rot = (Transpose[ref]).comp;
```

```
    {U, Sig, Vt} = SingularValueDecomposition[Rot];
    S = IdentityMatrix[m];
    H = Vt.S.(Transpose[U]);
    comp' = comp.H;
    Do[a[[i, j, k]] = comp'[[i, j]], {i, n}, {j, m}], {k, kg}] ×
  a;

 Do[
  sum = 0.0;
  Do[sum = sum + a[[i, j, k]], {k, kg}];
  ref[[i, j]] = sum / kg, {i, n}, {j, m}];
 ref;

 Do[
  sum = 0.0;
  Do[sum = sum + (park[[i, j]] - ref[[i, j]])^2, {j, m}];
  dist[[inc]] = √sum, {i, n}];
 park = ref;
 If[dist[[inc]] < tol, Break[]], {inc, cycles}]
dist;
p1 = BarChart[dist, LabelStyle → (FontFamily → "Arial"),
   ChartElementFunction → "GlassRectangle", ChartStyle → {Blue},
   ChartLabels → {"1", "2", "3", "4", "5", "6", "7", "8", "9", "10"}];

xMax = Max[a[[All, 1, All]]];
xMin = Min[a[[All, 1, All]]];
yMax = Max[a[[All, 2, All]]];
yMin = Min[a[[All, 2, All]]];
xRng = xMax - xMin;
yRng = yMax - yMin;
If[xRng > yRng,
  pltMax = xMax;
  pltMin = xMin,
  pltMax = yMax;
  pltMin = yMin];
```

Plot landmark/semilandmark point configurations in 2 D.

Obtain interactive plot parameters.

In[•]:=
```
Panel[
 Labeled[Column[{Row[{Panel[Labeled[InputField[Dynamic[config], FieldSize → 10],
        "Enter number of specimen to be plotted.", Top,
        LabelStyle → Directive[FontSize → 12, Bold, FontFamily → "Arial"]]], , ,
      Panel[Labeled[InputField[Dynamic[iconSize1], FieldSize → 10],
        "Enter plot icon size.", Top,
        LabelStyle → Directive[FontSize → 12, Bold, FontFamily → "Arial"]]]}],
    Row[{Panel[Labeled[InputField[Dynamic[pltPad], FieldSize → 10],
        "Enter plot padding factor.", Top,
        LabelStyle → Directive[FontSize → 12, Bold, FontFamily → "Arial"]]], , ,
      Panel[Labeled[InputField[Dynamic[pltSize1], FieldSize → 10],
        "Enter plot size factor.", Top,
        LabelStyle → Directive[FontSize → 12, Bold, FontFamily → "Arial"]]]}]},
   Center], "Single Plot Options Panel", Top, LabelStyle →
   Directive[FontSize → 16, Bold, FontFamily → "Arial"]]]
iconSize1 = 0.025; pltSize1 = 400; config = 1; pltPad = 0.2;
```

Out[•]=

Single Plot Options Panel

Enter number of specimen to be plotted.	Enter plot icon size.
config	iconSize1

Enter plot padding factor.	Enter plot size factor.
pltPad	pltSize1

Plot script

In[•]:=
```
iconList = Flatten[Table[
    {Graphics[{EdgeForm[{Thin, Black}], Hue[N[((kg + 1) - config) / kg]],
       Disk[{0, 0}, Scaled[iconSize1]]}]}, {j, 1}]];
cfigdat = a[[All, {1, 2}, config]];
lab1 = StringJoin["           ", ToString[objNames[[config]]]];
plot2D = Labeled[ListPlot[cfigdat, AspectRatio → 1, Frame → True, Axes → False,
    Joined → False, PlotRange → {{pltMin, pltMax}, {pltMin, pltMax}},
    PlotRangePadding → Scaled[pltPad], Ticks → Automatic,
    PlotMarkers → iconList, FrameLabel → {"v_x", "v_y"}, ImageSize → pltSize1,
    LabelStyle → Directive[FontSize → 12, FontFamily → "Arial"]], lab1, {Top},
   LabelStyle → Directive[FontSize → 18, Italic, FontFamily → "Arial"]];
plot2D
```

Export current 2 D plot.

In[∘]:=
```
filenameout = SystemDialogInput["FileSave"];
Export[filenameout, plot2D, "TIFF", ImageResolution → 150]
```

Out[∘]=
```
/Users/nm/Desktop/Leaves/Procrustes Results/Leaf 14.tif
```

Reorient point configuration.

Select transformation.

In[∘]:=
```
Panel[Labeled[Panel[PopupMenu[Dynamic[reOrient], {1 → "Flip across x-axis.",
      2 → "Flip across y-axis.", 2 → "Flip across both axes."}]],
   "Select Reorientation Option", Top, LabelStyle →
    Directive[FontSize → 14, Bold, FontFamily → "Arial"]]]
reOrient = 2;
```

Out[∘]=

Select Reorientation Option

Flip across x–axis. ∨

Reorientation calculations

In[∘]:=
```
If[reOrient == 1, Do[a[[i, 1, k]] = a[[i, 1, k]] * (-1.0), {i, n}, {k, kg}]];
If[reOrient == 2, Do[a[[i, 2, k]] = a[[i, 2, k]] * (-1.0), {i, n}, {k, kg}]];
If[reOrient == 3, Do[a[[i, j, k]] = a[[i, j, k]] * (-1.0), {i, n}, {j, m}, {k, kg}]];
```

Plot grid of all landmark/semilandmark point configurations in 2 D

Obtain interactive plot parameters.

In[∘]:=
```
Panel[Labeled[
  Column[{Row[{Panel[Labeled[InputField[Dynamic[iconSize], FieldSize → 10],
        "Enter icon size control parameter.", Top,
        LabelStyle → Directive[FontSize → 12, Bold, FontFamily → "Arial"]]], , ,
      Panel[Labeled[InputField[Dynamic[pltSize], FieldSize → 10],
        "Enter plot size control parameter.", Top,
        LabelStyle → Directive[FontSize → 12, Bold, FontFamily → "Arial"]]]}],
    Row[{Panel[Labeled[InputField[Dynamic[rowNum], FieldSize → 10],
        "Enter number of plots per grid row.", Top,
        LabelStyle → Directive[FontSize → 12, Bold, FontFamily → "Arial"]]], , ,
      Panel[Labeled[InputField[Dynamic[gridSize], FieldSize → 10],
        "Enter grid size control parameter.", Top,
        LabelStyle → Directive[FontSize → 12, Bold, FontFamily → "Arial"]]]}]},
   Center], "Grid Plot Options Panel", Top, LabelStyle →
   Directive[FontSize → 16, Bold, FontFamily → "Arial"]]]
iconSize = 0.025; pltSize = 275; gridSize = 900; rowNum = 3;
```

Out[∘]=
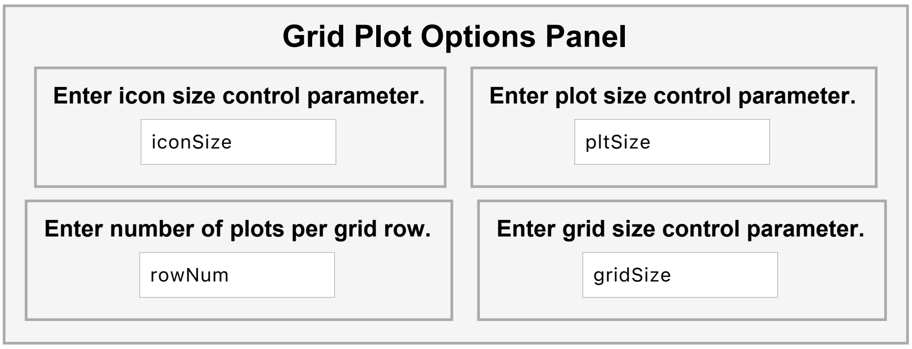

Plot all configurations in 2D

In[◦]:=
```
t1 = N[kg / rowNum];
t2 = IntegerPart[N[kg / rowNum]];
If[t1 - t2 > 0., kg2 = (t2 + 1) * rowNum, kg2 = t2 * rowNum];
pltArray = Table[" ", {kg2}];

Panel[Labeled[ProgressIndicator[Dynamic[k], {1, kg}], "Calculation Progress",
  Top, LabelStyle → Directive[FontSize → 12, Bold, FontFamily → "Arial"]]]

iconList = Flatten[Table[
    {Graphics[{EdgeForm[{Thin, Black}], Hue[N[((kg + 1) - j) / kg]],
       Disk[{0, 0}, Scaled[iconSize]]}]}, {j, kg}]];
Do[
 lab = StringJoin["                ", ToString[objNames[[k]]]];
 cfig2dat = a[[All, {1, 2}, k]];
 pltArray[[k]] = Labeled[ListPlot[cfig2dat, AspectRatio → 1, Frame → True,
    Axes → False, Joined → False, PlotStyle → Directive[Black, Thickness[0.004]],
    PlotRange → {{pltMin, pltMax}, {pltMin, pltMax}},
    PlotRangePadding → Scaled[pltPad], Ticks → Automatic,
    PlotMarkers → iconList[[k]], FrameLabel → {"vx", "vy"}, ImageSize → pltSize,
    LabelStyle → Directive[FontSize → 12, Black, FontFamily → "Arial"]],
   lab, Top, LabelStyle → Directive[FontSize → 14, Black,
     Italic, FontFamily → "Arial"]], {k, kg}]
pltGrid = Partition[pltArray, rowNum];
intPlotGrid = GraphicsGrid[pltGrid, ImageSize → gridSize]
```

Out[◦]=

Calculation Progress

Export 2 D plot of grid data.

In[◦]:=
```
filenameout = SystemDialogInput["FileSave"];
Export[filenameout, intPlotGrid, "TIFF", ImageResolution → 150]
```

Out[◦]= /Users/n.macleod/Desktop/Landmark Results/Grid Plots.tif

Plot overlay all landmark/semilandmark point configurations in 2 D

Obtain interactive plot parameters.

In[∘]:=
```
Panel[Labeled[
  Column[{Row[{Panel[Labeled[InputField[Dynamic[iconSize], FieldSize → 10],
        "Enter icon size control parameter.", Top,
        LabelStyle → Directive[FontSize → 12, Bold, FontFamily → "Arial"]]], , ,
      Panel[Labeled[InputField[Dynamic[pltSize], FieldSize → 10],
        "Enter plot size control parameter.", Top,
        LabelStyle → Directive[FontSize → 12, Bold, FontFamily → "Arial"]]]}],
    Row[{Panel[Labeled[InputField[Dynamic[rowNum], FieldSize → 10],
        "Enter number of plots per grid row.", Top,
        LabelStyle → Directive[FontSize → 12, Bold, FontFamily → "Arial"]]], , ,
      Panel[Labeled[InputField[Dynamic[gridSize], FieldSize → 10],
        "Enter grid size control parameter.", Top,
        LabelStyle → Directive[FontSize → 12, Bold, FontFamily → "Arial"]]]}]},
   Center], "Overlay Plot Options Panel", Top, LabelStyle →
   Directive[FontSize → 16, Bold, FontFamily → "Arial"]]]
iconSize = 0.025; pltSize = 275; gridSize = 900; rowNum = 3;
```

Out[∘]=

Overlay Plot Options Panel

**Enter icon size control parameter.** iconSize	**Enter plot size control parameter.** pltSize
**Enter number of plots per grid row.** rowNum	**Enter grid size control parameter.** gridSize

Plot of overlayed configurations in 2 D
User – defined parameters
  iconSize – control parameter for plot icon size

In[∘]:=
```
overArray = Table[" ", {kg}];
iconList = Table[
   {Graphics[{EdgeForm[{Thin, Black}],
      Hue[N[((kg + 1) - j) / kg]], Disk[{0, 0}, Scaled[iconSize]]}]}, {j, kg}];
Do[
 overArray〚k〛 = ListPlot[a〚All, {1, 2}, k〛, AspectRatio → 1, Frame → True,
   Joined → False, Axes → False, PlotRange → {{pltMin, pltMax}, {pltMin, pltMax}},
   PlotRangePadding → Scaled[0.15], Ticks → Automatic,
   PlotMarkers → iconList〚k〛, FrameLabel → {"v_x", "v_y"}, ImageSize → pltSize1,
   LabelStyle → Directive[FontSize → 14, Black, FontFamily → "Arial"]], {k, kg}]

overlayPlot = Labeled[Show[overArray], "             Overlay Plot", Top,
  LabelStyle → Directive[FontSize → 18, Black, FontFamily → "Arial"]]
```

Export 2 D plot of overlayed shapes.

In[∘]:=
```
filenameout = SystemDialogInput["FileSave"];
Export[filenameout, overlayPlot, "TIFF", ImageResolution → 150]
```

Out[∘]= /Users/n.macleod/Desktop/Landmark Results/Overlay Plot.tif

Plot mean of all landmark/semilandmark point configurations in 2 D

Obtain interactive plot parameters.

In[∘]:=
```
Panel[Labeled[
  Column[{Row[{Panel[Labeled[InputField[Dynamic[iconSize], FieldSize → 10],
        "Enter icon size control parameter.", Top,
        LabelStyle → Directive[FontSize → 12, Bold, FontFamily → "Arial"]]], , ,
      Panel[Labeled[InputField[Dynamic[pltSize], FieldSize → 10],
        "Enter plot size control parameter.", Top,
        LabelStyle → Directive[FontSize → 12, Bold, FontFamily → "Arial"]]]}],
    Row[{Panel[Labeled[InputField[Dynamic[rowNum], FieldSize → 10],
        "Enter number of plots per grid row.", Top,
        LabelStyle → Directive[FontSize → 12, Bold, FontFamily → "Arial"]]], , ,
      Panel[Labeled[InputField[Dynamic[gridSize], FieldSize → 10],
        "Enter grid size control parameter.", Top,
        LabelStyle → Directive[FontSize → 12, Bold, FontFamily → "Arial"]]]}]},
   Center], "Mean Plot Options Panel", Top, LabelStyle →
   Directive[FontSize → 16, Bold, FontFamily → "Arial"]]]
iconSize = 0.025; pltSize = 275; gridSize = 900; rowNum = 3;
```

Out[∘]=

Mean Plot Options Panel

**Enter icon size control parameter.**	**Enter plot size control parameter.**
iconSize	pltSize
**Enter number of plots per grid row.**	**Enter grid size control parameter.**
rowNum	gridSize

Plot mean shape in 2 D

User – defined parameters

iconSize – control parameter for plot icon size

In[∘]:=

```
mShape = Table[0.0, {i, n}, {j, m}];
Do[mShape = mShape + a⟦All, All, k⟧ / kg, {k, kg}]
mShape;

iconList = Flatten[Table[
    {Graphics[{EdgeForm[{Thickness[0.005], White}],
       Black, Disk[{0, 0}, Scaled[iconSize]]}]}, {j, 1}]];

mShape12 = Take[mShape, All, 2];
mShapePlot = Labeled[ListPlot[mShape12, AspectRatio → 1, Frame → True,
   Axes → False, PlotRange → {{pltMin, pltMax}, {pltMin, pltMax}},
   PlotRangePadding → Scaled[.15], Joined → False, Ticks → Automatic,
   PlotMarkers → iconList, FrameLabel → {"vx", "vy"}, ImageSize → pltSize1,
   LabelStyle → Directive[FontSize → 14, Black, FontFamily → "Arial"]],
  "              Mean Shape", {Top},
  LabelStyle → Directive[FontSize → 18, Black, FontFamily → "Arial"]]
```

Export 2 D plot of mean shape.

In[∘]:=

```
filenameout = SystemDialogInput["FileSave"];
Export[filenameout, mShapePlot, "TIFF", ImageResolution → 150]
```

Out[∘]= /Users/n.macleod/Desktop/Landmark Results/Mean Shape.tif

Export mean shape coordinates for external analysis.

In[∘]:=

```
filenameout = SystemDialogInput["FileSave"];
If[outFileType == 1, Export[filenameout, Flatten[mShape], "CSV"],
 Export[filenameout, mShape, "Table"]]
```

Out[∘]= /Users/n.macleod/Desktop/Landmark Results/Mean Shape.csv

Plot individual coordinate dataset in 3 D (use only with 3 D data)

Set plot parameters

In[∘]:=
```
Panel[Labeled[Column[{Panel[Labeled[
      PopupMenu[Dynamic[specName], objNames], "Select specimen to be plotted.",
      Top, LabelStyle → Directive[FontSize → 12, Bold, FontFamily → "Arial"]]],
    Row[{Panel[Labeled[InputField[Dynamic[iconSz1], FieldSize → 10],
        "Enter icon size value.", Top, LabelStyle →
         Directive[FontSize → 12, Bold, FontFamily → "Arial"]]], "   ",
      Panel[Labeled[InputField[Dynamic[pltSz1], FieldSize → 10],
        "Enter plot size value.", Top, LabelStyle →
         Directive[FontSize → 12, Bold, FontFamily → "Arial"]]]}]}, Center],
  "3D Plot Options", Top, LabelStyle → Directive[FontSize → 18,
    Bold, FontFamily → "Arial"]]]
specName = objNames⟦1⟧; iconSz1 = 0.002; pltSz1 = 600;
```

Out[∘]=

3D Plot Options

**Select specimen to be plotted.**
PopupMenu[specName, objNames]

**Enter icon size value.**	**Enter plot size value.**
iconSz1	pltSz1

••• Part: Part specification objNames⟦1⟧ is longer than depth of object.

Plot specimen

In[∘]:=
```
Do[If[specName == objNames⟦i⟧, specimenNum = i], {i, kg}];
singPdPlt = a⟦All, All, specimenNum⟧;
Do[singPdPlt⟦i⟧ = Table[Sphere[singPdPlt⟦i⟧, iconSz1], {1}], {i, n}]
Flatten[singPdPlt];
plt3D = Graphics3D[
   {Hue[N[((kg + 1) - specimenNum) / kg]], PointSize[0.5], singPdPlt}, Axes → True,
   Boxed → True, LabelStyle → Directive[FontSize → 10, FontFamily → "Arial"],
   AxesLabel → {"X", "Y", "Z"}];
Show[plt3D, ImageSize → pltSz1]
```

Export specimen plot

```
filenameout = SystemDialogInput["FileSave"];
Export[filenameout, plot3D, "TIFF"]
```

Plot overlayed 3 D coordinate datasets (use only with 3 D data)

Set plot parameters

In[•]:=
```
Panel[Labeled[Row[{Panel[Labeled[InputField[Dynamic[iconSz1], FieldSize → 10],
      "Enter icon size value.", Top,
      LabelStyle → Directive[FontSize → 12, Bold, FontFamily → "Arial"]]], "  ",
    Panel[Labeled[InputField[Dynamic[pltSz2], FieldSize → 10],
      "Enter plot size value.", Top,
      LabelStyle → Directive[FontSize → 12, Bold, FontFamily → "Arial"]]]}],
  "3D Overlay Plot Options", Top, LabelStyle →
   Directive[FontSize → 18, Bold, FontFamily → "Arial"]]]
specName = objNames⟦1⟧; iconSz2 = 0.002; pltSz1 = 600;
```

Out[•]=

3D Overlay Plot Options

Enter icon size value.	Enter plot size value.
iconSz1	pltSz2

Part: Part specification objNames⟦1⟧ is longer than depth of object.

Plot overlayed point clouds

In[•]:=
```
ptPlots3D = Table[" ", {kg}];
Do [
 singPdPlt = a⟦All, All, k⟧;
 Do[singPdPlt⟦i⟧ = Table[Sphere[singPdPlt⟦i⟧, iconSz2], {1}], {i, n}] ×
  Flatten[singPdPlt];
 ptPlots3D⟦k⟧ = Graphics3D[{Hue[N[((kg + 1) - k) / kg]],
     PointSize[0.2], singPdPlt}, Axes → True, Boxed → True,
    LabelStyle → Directive[Black, FontSize → 12, FontFamily → "Arial"],
    AxesLabel → {"x-Axis", "y-Axis", "z-Axis"}], {k, kg}]
overlay3D = Labeled[Show[ptPlots3D, ImageSize → pltSz2], "Overlay Plot",
  Top, LabelStyle → Directive[Black, FontSize → 16, FontFamily → "Arial"]]
```

Export 3 D overtlay plot . [Use only with 3 D data.]

In[•]:=
```
filenameout = SystemDialogInput["FileSave"];
Export[filenameout, overlay3D, "TIFF"]
```

Out[•]= /Users/n.macleod/Desktop/Small-2/Procrustes Results/3D Overlay Plot.tif

Plot mean point clouds

In[∘]:=
```
mShape = Table[" ", {n}, {m}];
Do[mShape⟦i⟧ = Mean[Transpose[a⟦i⟧]], {i, n}]
mShapePts = Table[" ", {n}];
Do[mShapePts⟦i⟧ = Table[Sphere[mShape⟦i⟧, iconSz2], {1}], {i, n}]
mShpPlot3D =
  Graphics3D[{Hue[0, 0, 0], PointSize[0.2], mShapePts}, Axes → True, Boxed → True,
   LabelStyle → Directive[Black, FontSize → 12, FontFamily → "Arial"],
   AxesLabel → {"x-Axis", "y-Axis", "z-Axis"}];
mShpPlot = Labeled[Show[mShpPlot3D, ImageSize → pltSz2], "Mean Shape",
  Top, LabelStyle → Directive[Black, FontSize → 14, FontFamily → "Arial"]]
```

Export 3 D mean shape plot. [Use only with 3 D data.]

In[∘]:=
```
filenameout = SystemDialogInput["FileSave"];
Export[filenameout, mShapePlot, "TIFF"]
```

Out[∘]= /Users/n.macleod/Desktop/Small-2/Procrustes Results/3D Mean Plot.tif

Datafile Export Routines

Export centroid size values.

In[∘]:=
```
filenameout = SystemDialogInput["FileSave"];
Export[filenameout, csize, "CSV"]
```

Out[∘]= /Users/n.macleod/Desktop/Drangonflies (Final)/Data & Results/GM/ Hindwings/Centroid Sizes.csv

Assign the images to groups.

Select user – specified plot options.
You don't have to run this section, just make whatever changes, if any, you'd like.

In[∘]:=
```
Panel[
 Labeled[PopupMenu[Dynamic[gpOption], {1 → "Assign all images to 'Group 1'",
    2 → "Use external file to make group assignments"}], "Specify group names",
  Top, LabelStyle → Directive[FontSize → 12, Bold, FontFamily → "Arial"]]]
gpOption = 1;
```

Out[∘]=

Specify group names

Assign all images to 'Group 1' ⌄

Generate or read – in group file.

In[∘]:=
```
If[gpOption == 1, groupNames = Table["Group 1", {kg}];,
 filenamein = SystemDialogInput["FileOpen"];
 sourceDirectory = DirectoryName[filenamein];
 groupNames = Flatten[Import[filenamein, "CSV"]];
 filenamein]
```

Select datafile export format.

In[∘]:=
```
Panel[PopupMenu[Dynamic[outFileType],
   {1 → "Standard Data Matrix Format", 2 → ".dat Format"}]]
outFileType = 1;
```

Out[∘]=
Standard Data Matrix Format

Export superposed coordinates for external analysis.

In[•]:=
```
If[outFileType == 1,
 adata = Table[0.0, {i, kg}, {j, (n * m)}];
 inc = 0;
 Do[
  Do[
   inc = inc + 1;
   Do[
    adata⟦k, inc⟧ = a⟦i, j, k⟧, {k, kg}], {j, m}], {i, n}];
 adata;

 hdata = Table[0, {kg + 1}, {(n * m) + 2}];
 hdata⟦1, 1⟧ = "Object";
 hdata⟦1, 2⟧ = "Group";
 Do[hdata⟦k + 1, 1⟧ = objNames⟦k⟧, {k, kg}];
 Do[hdata⟦k + 1, 2⟧ = groupNames⟦k⟧, {k, kg}];
 knt = 0;
 Do[
  knt = knt + 1;
  hdata⟦1, j + 2⟧ = StringJoin["x", ToString[knt]];
  hdata⟦1, j + 3⟧ = StringJoin["y", ToString[knt]];
  If[m == 3,
   hdata⟦1, j + 4⟧ = StringJoin["z", ToString[knt]]], {j, 1, (n * m), m}];

 Do[hdata⟦i + 1, j + 2⟧ = adata⟦i, j⟧, {i, kg}, {j, (n * m)}];

 filenameout = SystemDialogInput["FileSave"];
 Export[filenameout, hdata, "CSV", "TextDelimiters" → ""]]

If[outFileType == 2,
 {n, m, kg} = Dimensions[a];
 adata = Table[" ", {n * kg + kg}, {m}];
 knt = 0;
 Do[
  knt = knt + 1;
  adata⟦knt, 1⟧ = objNames⟦k⟧;
  Do[
   knt = knt + 1;
   adata⟦knt⟧ = a⟦i, All, k⟧, {i, n}], {k, kg}];

 filenameout = SystemDialogInput["FileSave"];
 Export[filenameout, adata, "Table"]]
```

Out[•]= /Users/n.macleod/Desktop/Landmark Results/Landmarks (Super).csv

Export reformated input data.

Select data export format

In[ ]:=
```
Panel[PopupMenu[Dynamic[outFileType],
  {1 → "Standard Data Matrix Format", 2 → ".dat Format"}]]
outFileType = 1;
```

Out[ ]=
Standard Data Matrix Format

Export input data for external analysis.

```
In[•]:= If[outFileType == 1,
  adata = Table[0.0, {i, kg}, {j, (n * m)}];
  inc = 0;
  Do[
    Do[
     inc = inc + 1;
     Do[
      adata⟦k, inc⟧ = x1Coords⟦i, j, k⟧, {k, kg}], {j, m}], {i, n}] ×
   adata;

  hdata = Table[0, {kg + 1}, {(n * m) + 2}];
  hdata⟦1, 1⟧ = "Object";
  hdata⟦1, 2⟧ = "Group";
  Do[hdata⟦k + 1, 1⟧ = objNames⟦k⟧, {k, kg}] ×
   Do[hdata⟦k + 1, 2⟧ = "Group 1", {k, kg}] ×
   knt = 0;
  Do[
    knt = knt + 1;
    hdata⟦1, j + 2⟧ = StringJoin["x", ToString[knt]];
    hdata⟦1, j + 3⟧ = StringJoin["y", ToString[knt]];
    If[m == 3,
     hdata⟦1, j + 4⟧ = StringJoin["z", ToString[knt]]], {j, 1, (n * m), m}]
   ×
   Do[hdata⟦i + 1, j + 2⟧ = adata⟦i, j⟧, {i, kg}, {j, (n * m)}] ×
   hdata;

  filenameout = SystemDialogInput["FileSave"];
  Export[filenameout, hdata, "CSV"]]

 If[outFileType == 2,
  {n, m, kg} = Dimensions[a];
  adata = Table[" ", {n * kg + kg}, {m}];
  knt = 0;
  Do[
   knt = knt + 1;
   adata⟦knt, 1⟧ = objNames⟦k⟧;
   Do[
    knt = knt + 1;
    adata⟦knt⟧ = x1Coords⟦i, All, k⟧, {i, n}], {k, kg}];

  filenameout = SystemDialogInput["FileSave"];
  Export[filenameout, adata, "Table"]]

Out[•]= /Users/n.macleod/Desktop/Landmark Results/Landmarks.csv
```
