# Supplementary material for: What you sample is what you get: ecomorphological variation in Trithemis (Odonata, Libellulidae) dragonfly wings reconsidered
Source: BMC Ecol Evol. 2022 Apr 11;22:43. doi: 10.1186/s12862-022-01978-y (PMC8996507; doi:10.1186/s12862-022-01978-y)
Supplement: Supplementary file 3 — Additional file 3: Software Archive. [file 12862_2022_1978_MOESM3_ESM.zip › Additional Files 3/Software Archive/PCA (5.12).pdf]

Principal Component Analysis (PCA)

This program accepts a data matrix in standard format , performs a principal component analysis on the data columns, and allows the result to be viewed as 2D or (interactive) 3D scatterplots. Full support is provided for tracking groups that have been identified in the datafile.

Author: N. MacLeod
Version: 5.12
Date: 22 August  2021
Reference: MacLeod (2007)

Initialize libraries.

In[∘]:=
```
<< ComputationalGeometry`
```

Read in data file & partition into datasets.

In[∘]:=
```
filenamein = SystemDialogInput["FileOpen"];
x1 = Import[filenamein, "CSV"];
filenamein

{n1, m1} = Dimensions[x1];

varNames = Flatten[Take[x1, 1]];
x2 = Drop[x1, 1];
varNames = Drop[varNames, 1];
varNames = Drop[varNames, 1];

objNames = Flatten[Take[x2, n1 - 1, 1]];
x2 = Drop[x2, 0, 1];

Group = Flatten[Take[x2, n1 - 1, 1]];
numGroups = Length[Union[Group]];
x2 = Drop[x2, 0, 1];
kg = Length[Union[Group]];

{n2, m2} = Dimensions[x2];
Print["No. of Objects: ", n2]
Print["No. of Variables: ", m2]
Print["No. of Groups: ", kg]

varNames;
objNames;
Group;
x2;
```

Out[∘]=
```
/Users/n.macleod/Desktop/Data (20).csv
```

```
No. of Objects: 80

No. of Variables: 4

No. of Groups: 4
```

## Data transformations (optional)

Specify global data transformations (if any).
Note : Remember you cannot subsequently take the logarithm of mean –
centered or standardized data. If you wish
to perform such an analysis you must shift the mean –
centered or standardized data by a constant (e.g., 1, 10)

In[∘]:=
```
Panel[Labeled[Column[
  {Row[{Panel[Labeled[PopupMenu[Dynamic[meanTrans], {1 → "No", 2 → "Yes"}],
       "Mean center data?", Top, LabelStyle →
        Directive[FontSize → 12, Bold, FontFamily → "Arial"]]], "  ",
     Panel[Labeled[PopupMenu[Dynamic[logTrans], {1 → "No", 2 → "Yes"}],
       "Log10-trasform data?", Top, LabelStyle →
        Directive[FontSize → 12, Bold, FontFamily → "Arial"]]], "  ",
     Panel[Labeled[PopupMenu[Dynamic[stdTrans], {1 → "No", 2 → "Yes"}],
       "Standardize data?", Top,
       LabelStyle → Directive[FontSize → 12, Bold, FontFamily → "Arial"]]]}],
   Row[{Panel[Labeled[PopupMenu[Dynamic[shiftTrans], {1 → "No", 2 → "Yes"}],
       "Shift data by a constant?", Top, LabelStyle →
        Directive[FontSize → 12, Bold, FontFamily → "Arial"]]], "  ",
     Panel[Labeled[InputField[Dynamic[knsnt], FieldSize → 5],
       "Enter shift constant value.", Top,
       LabelStyle → Directive[FontSize → 12, Bold, FontFamily → "Arial"]]]}]},
  Center], "Global Data Transform Options", Top, LabelStyle →
  Directive[FontSize → 18, Bold, FontFamily → "Arial"]]]
meanTrans = 1; logTrans = 1; stdTrans = 1; shiftTrans = 1; knsnt = 10;
```

Out[∘]=

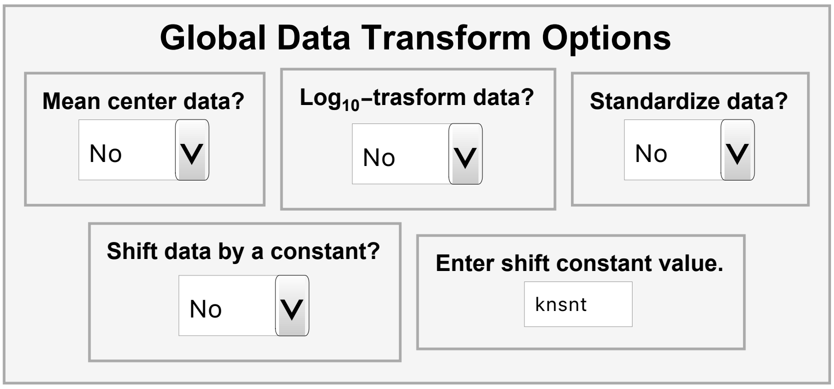


## Perform global data transformation (optional)

In[∘]:=
```
If[meanTrans == 2,
  mVec = N[Mean[x2]];
  Do[x2[[i]] = x2[[i]] - mVec, {i, n2}]];
If[logTrans == 2, x2 = N[Log10[x2]]];
If[stdTrans == 2, x2 = Standardize[x2]];
If[shiftTrans == 2, x2 = x2 + knsnt];
```

Export mean vector (optional; may be useful in modelling).

In[∘]:=
```
filenameout = SystemDialogInput["FileSave"];
Export[filenameout, mVec, "csv"]
```

Out[∘]=
```
/Users/nm/Desktop/Lupus II/Lateral/EFourier Data
  & Results/PCA Results/Lateral Outlines (Mean Vector).csv
```

Export processed dataset (optional; may be useful in other analyses).

```
x2Trans = Table[" ", {n2 + 1}, {m2 + 2}];
x2Trans[[1, 1]] = "Object";
x2Trans[[1, 2]] = "Group";
Do[x2Trans[[1, j + 2]] = varNames[[j]], {j, m2}]
Do[x2Trans[[i + 1, 1]] = objNames[[i]], {i, n2}]
Do[x2Trans[[i + 1, 2]] = Group[[i]], {i, n2}]
Do[x2Trans[[i + 1, j + 2]] = x2[[i, j]], {i, n2}, {j, m2}]

filenameout = SystemDialogInput["FileSave"];
Export[filenameout, x2Trans, "CSV", "TextDelimiters" → ""]
```

Choose basis matrix and eigenanalysis method

In[∘]:=
```
Panel[Labeled[Column[{Row[{Panel[Labeled[
        PopupMenu[Dynamic[basisMat], {1 → "Covariance Matrix (Calculated)", 2 →
           "Correlation Matrix (Calculated)", 3 → "Covariance Matrix (Input)",
          4 → "Correlation Matrix (Input)"}], "Choose similarity matrix.", Top,
        LabelStyle → Directive[FontSize → 14, FontFamily → "Arial"]]], "  ",
      Panel[Labeled[PopupMenu[Dynamic[eMethod], {1 → "Std. Eigenanalysis",
          2 → "Singular Value Decomposition"}], "Choose eigenanalysis method.",
        Top, LabelStyle → Directive[FontSize → 14, FontFamily → "Arial"]]]}],
    Panel[Labeled[RadioButtonBar[Dynamic[scal], {1 → "Classic PCA",
        2 → "Eigenvalue Scaling"}], "Specify eigenvector scaling method.",
      Top, LabelStyle → Directive[FontSize → 14, FontFamily → "Arial"]]]},
   Center], "Choose Analysis Options", Top, LabelStyle →
   Directive[FontSize → 16, Bold, FontFamily → "Arial"]]]
basisMat = 1; eMethod = 1; scal = 2;
```

Out[∘]=

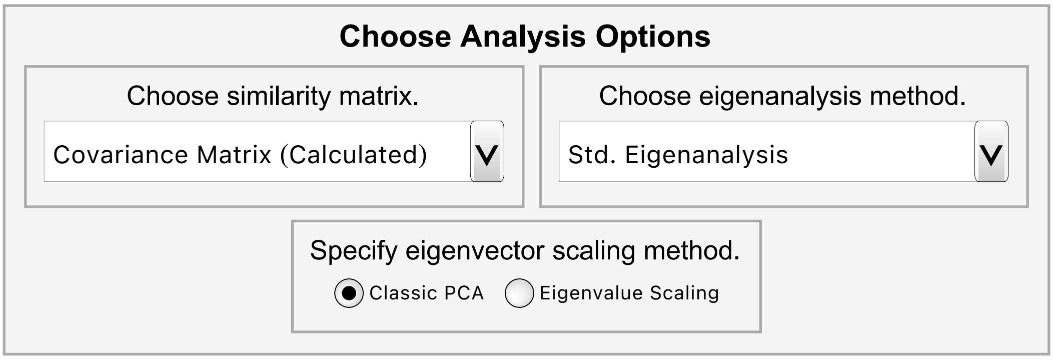


## Perform eigenanalysis.

```
In[•]:= If[basisMat == 1, coVar = Covariance[x2]];
If[basisMat == 2, coVar = Correlation[x2]];
If[basisMat > 2, filenamein = SystemDialogInput["FileOpen"];
  coVar = Import[filenamein, "CSV"]];
If[n2 > m2, mvecs = m2, mvecs = n2];
If[n2 > m2, mvecs = m2, mvecs = n2];
If[eMethod == 1,
  eVals = Eigenvalues[coVar];
  w = Eigenvectors[coVar];
  eVecs = Transpose[w]];
If[eMethod == 2,
  {w, u, v} = SingularValueDecomposition[coVar];
  eVals = N[Diagonal[u], 3];
  eVecs = w];
If[scal == 2,
  Λ = Table[0.0, {m2}, {m2}];
  Do[Λ[[i, i]] = Sqrt[eVals[[i]]], {i, m2}];
  eVecs = eVecs.Λ];
If[basisMat == 1 || basisMat == 3,
  eScores = x2.eVecs, eScores = Standardize[x2].eVecs];

s1 = Total[eVals];
evalTable = Table[0.0, {i, mvecs}, {j, 4}];
Do[evalTable[[i, 1]] = i, {i, 1, mvecs}];
Do[evalTable[[i, 2]] = eVals[[i]], {i, 1, mvecs}];
Do[evalTable[[i, 3]] = (eVals[[i]] 100.`)/s1, {i, 1, mvecs}];
Do[evalTable[[i, 4]] = evalTable[[i, 3]], {i, 1, mvecs}];
Do[evalTable[[i, 4]] = evalTable[[i - 1, 4]] + evalTable[[i, 3]], {i, 2, mvecs}];

tabHeads =
  {"Component", " Eigenvalue", "  Variance (%)", "  Cum. Variance (%)"};
t2 = Partition[Flatten[Join[tabHeads, evalTable]], 4];
t3 = Partition[Flatten[t2], 4];

Do[
 Do[
  t3[[i, j]] = PaddedForm[t3[[i, j]], {5, 3}], {i, 2, mvecs + 1}], {j, 2, 4}]

Labeled[Grid[t3, BaseStyle → (FontFamily → "Arial"),
  Alignment → {{Center, Right, Right, Right}}, Frame → True,
  Dividers → {{True, True}, {True, True}}], "Eigenvalue Table", Top,
 LabelStyle → Directive[FontSize → 14, Bold, FontFamily → "Arial"]]
```

## Specify no. of axes to display

In[∘]:=
```
Panel[Labeled[InputField[Dynamic[noAxes], FieldSize → 10],
   "Enter number of PC axes to retain.", Top,
   LabelStyle → Directive[FontSize → 12, FontFamily → "Arial"]]]
noAxes = mvecs;
```

Out[∘]=

Enter number of PC axes to retain.

noAxes

## Display PCA Results (optional).

### Display PC loadings (optional).

In[∘]:=
```
evecTable = Table[0.0, {i, m2 + 1}, {j, noAxes + 1}];
Do[evecTable⟦i + 1, 1⟧ = varNames⟦i⟧, {i, m2}]
evecTable⟦1, 1⟧ = "Variable";
Do[evecTable⟦1, i + 1⟧ = StringJoin["PC-", ToString[i]], {i, noAxes}]
Do[Do[evecTable⟦i + 1, j + 1⟧ = PaddedForm[eVecs⟦i, j⟧, {5, 3}], {i, m2}],
  {j, noAxes}]
Labeled[Grid[evecTable, BaseStyle → (FontFamily → "Arial"),
   Alignment → {{Center, Right, Right, Right, Right, Right}}, Frame → True,
   Dividers → {{True, True}, {True, True}}], "Eigenvector Table", Top,
  LabelStyle → Directive[Black, Bold, FontSize → 14, FontFamily → "Arial"]]
```

### Reverse direction of eigenvectors (optional).

In[∘]:=
```
eVecs = eVecs * -1;
If[basisMat == 1 || basisMat == 3,
   eScores = x2.eVecs, eScores = Standardize[x2].eVecs];
```

### Display PC scores (optional).

In[∘]:=
```
scrTable = Table[0.0, {i, n2 + 1}, {j, noAxes + 1}];
Do[scrTable⟦i + 1, 1⟧ = objNames⟦i⟧, {i, n2}]
scrTable⟦1, 1⟧ = "Objects";
Do[scrTable⟦1, i + 1⟧ = StringJoin["PC-", ToString[i]], {i, noAxes}]
Do[Do[scrTable⟦i + 1, j + 1⟧ = PaddedForm[eScores⟦i, j⟧, {5, 3}], {i, n2}],
  {j, noAxes}]
Labeled[Grid[scrTable, BaseStyle → (FontFamily → "Arial"),
   Alignment → {{Left, Right, Right, Right, Right, Right}}, Frame → True,
   Dividers → {{True, True}, {True, True}}], "Eigenscore Table", Top,
  LabelStyle → Directive[Black, Bold, FontSize → 14, FontFamily → "Arial"]]
```

## Calculate original, reproduced and residual covariance/correlation matrices

In[∘]:=
```
Λ = Table[0.0, {noAxes}, {noAxes}];
Do[Λ⟦i, i⟧ = √eVals⟦i⟧ , {i, noAxes}];
fVecs = Take[eVecs, All, noAxes].Λ;
eVecs = Take[eVecs, All, noAxes];
If[basisMat == 1 || basisMat == 3 ,
  If[scal == 1 && noAxes == m2,
   eVecsT = Transpose[eVecs];
   fVecsT = Transpose[fVecs];
   repro = eVecs.eVecsT;
   reproScal = fVecs.fVecsT;
   repro = Chop[repro * reproScal]]];
If[basisMat == 1 || basisMat == 3 ,
  If[scal == 1 && noAxes ≠ m2,
   eVecsT = Transpose[eVecs];
   repro = eVecs.eVecsT]];
If[basisMat == 2 || basisMat == 4 && scal == 1,
  eVecsT = Transpose[eVecs];
  repro = Chop[eVecs.eVecsT]];
If[scal == 2,
  eVecsT = Transpose[eVecs];
  repro = eVecs.eVecsT];
resid = Chop[coVar - repro];
```

## Display original, reproduced and residual covariance/correlation matrices

In[∘]:=
```
covarTable = Table[" ", {m2 + 1}, {m2 + 1}];
reproTable = Table[" ", {m2 + 1}, {m2 + 1}];
residTable = Table[" ", {m2 + 1}, {m2 + 1}];

If[basisMat == 1 || basisMat == 3,
  title1 = StringJoin["Original ", ToString["Covariances"]],
  title1 = StringJoin["Original ", ToString["Correlations"]]];
If[basisMat == 1 || basisMat == 3,
  title2 = StringJoin["Reproduced ", ToString["Covariances"]],
  title2 = StringJoin["Reproduced ", ToString["Correlations"]]];
If[basisMat == 1 || basisMat == 3,
  title3 = StringJoin["Residual ", ToString["Covariances"]],
  title3 = StringJoin["Residual ", ToString["Correlations"]]];

covarTable⟦1, 1⟧ = "Variable";
Do[covarTable⟦1, j + 1⟧ = varNames⟦j⟧, {j, m2}]
Do[covarTable⟦i + 1, 1⟧ = varNames⟦i⟧, {i, m2}]
Do[covarTable⟦i + 1, j + 1⟧ = coVar⟦i, j⟧, {i, m2}, {j, m2}]
t5 = covarTable;
```

```
Do[
 Do[
  t5⟦i, j⟧ = PaddedForm[t5⟦i, j⟧, {5, 4}], {i, 2, m2 + 1}], {j, 2, m2 + 1}]
Labeled[Grid[t5, BaseStyle → (FontFamily → "Arial"), Alignment → {{Left, Center}},
  Frame → True, Dividers → {{True, True}, {True, True}}], title1, Top,
 LabelStyle → Directive[Bold, FontSize → 14, FontFamily → "Arial"]]

reproTable⟦1, 1⟧ = "Variable";
Do[reproTable⟦1, j + 1⟧ = varNames⟦j⟧, {j, m2}]
Do[reproTable⟦i + 1, 1⟧ = varNames⟦i⟧, {i, m2}]
Do[reproTable⟦i + 1, j + 1⟧ = repro⟦i, j⟧, {i, m2}, {j, m2}]
t6 = reproTable;
Do[
 Do[
  t6⟦i, j⟧ = PaddedForm[t6⟦i, j⟧, {5, 4}], {i, 2, m2 + 1}], {j, 2, m2 + 1}]
Labeled[Grid[t6, BaseStyle → (FontFamily → "Arial"), Alignment → {{Left, Center}},
  Frame → True, Dividers → {{True, True}, {True, True}}], title2, Top,
 LabelStyle → Directive[Bold, FontSize → 14, FontFamily → "Arial"]]

residTable⟦1, 1⟧ = "Variable";
Do[residTable⟦1, j + 1⟧ = varNames⟦j⟧, {j, m2}]
Do[residTable⟦i + 1, 1⟧ = varNames⟦i⟧, {i, m2}]
Do[residTable⟦i + 1, j + 1⟧ = resid⟦i, j⟧, {i, m2}, {j, m2}]
t7 = residTable;
Do[
 Do[
  t7⟦i, j⟧ = PaddedForm[t7⟦i, j⟧, {5, 4}], {i, 2, m2 + 1}], {j, 2, m2 + 1}]
Labeled[Grid[t7, BaseStyle → (FontFamily → "Arial"), Alignment → {{Left, Center}},
  Frame → True, Dividers → {{True, True}, {True, True}}], title3, Top,
 LabelStyle → Directive[Bold, FontSize → 14, FontFamily → "Arial"]]
```

## Export original reproduced and residual covariances/correlations tables (optional).

```
filenameout = SystemDialogInput["FileSave"];
Export[filenameout, covarTable, "CSV", "TextDelimiters" → ""]
filenameout = SystemDialogInput["FileSave"];
Export[filenameout, reproTable, "CSV", "TextDelimiters" → ""]
filenameout = SystemDialogInput["FileSave"];
Export[filenameout, residTable, "CSV", "TextDelimiters" → ""]
```

## Display scatterplot of original vs reproduced covariance/correlation values w/ cophenetic correlation (optional)

In[*]:=
```
pltPad = 0.10;
iconList = Flatten[Table[
    {Graphics[{EdgeForm[{Thin, Black}], Hue[1 / 3], Disk[{0, 0}, Scaled[0.035]]}]},
    {j, 1}]];
If[basisMat == 1 || basisMat == 3, tit1 = "Covariance Values",
  tit1 = "Correlation Values"];
title = StringJoin["      Original vs. Reproduced ", ToString[tit1]];
orig = Flatten[coVar];
rep = Flatten[repro];
ccText = StringJoin["Cophenetic Correlation:",
   ToString[PaddedForm[Correlation[orig, rep], {5, 4}]]];
ccText = Text[ccText, BaseStyle → {FontSize → 14, FontFamily → "Arial"}];
lData = Riffle[Flatten[coVar], Flatten[repro]];
pos = {Min[lData], Max[lData]};
int = (pos[[2]] - pos[[1]]) * 0.05;
lowpos = pos[[1]] - int;
highpos = pos[[2]] + int;
txtData = {Text[ccText, pos, {-1, 0}]};
pData = Partition[lData, 2];

lnPlot = Graphics[
   {Black, Thickness[0.0010], Line[{{lowpos, lowpos}, {highpos, highpos}}]},
   AspectRatio → 1 / GoldenRatio, Frame → True, Axes → False,
   PlotRange → {{pos[[1]], pos[[2]]}, {pos[[1]], pos[[2]]}},
   PlotRangePadding → Scaled[pltPad], Ticks → Automatic,
   FrameLabel → {"Original Values", "Reproduced Values"}, ImageSize → 500,
   LabelStyle → Directive[Black, FontSize → 14, FontFamily → "Arial"]];
ptPlot = ListPlot[pData, AspectRatio → 1 / GoldenRatio, Frame → True,
   Axes → False, PlotRange → Automatic, PlotMarkers → iconList,
   PlotRangePadding → Scaled[pltPad], Ticks → Automatic,
   FrameLabel → {"Original Values", "Reproduced Values"}, ImageSize → 500,
   LabelStyle → Directive[Black, FontSize → 14, FontFamily → "Arial"]];
txtPlot = Graphics[txtData, AspectRatio → 1 / GoldenRatio, Frame → True,
   Axes → False, PlotRange → {{pos[[1]], pos[[2]]}, {pos[[1]], pos[[2]]}},
   PlotRangePadding → Scaled[pltPad], Ticks → Automatic,
   FrameLabel → {"Original Values", "Reproduced Values"}, ImageSize → 500,
   LabelStyle → Directive[Black, FontSize → 14, FontFamily → "Arial"]];
ccPlot = Labeled[Show[{lnPlot, ptPlot, txtPlot}], title, Top,
  LabelStyle → Directive[FontSize → 18, FontFamily → "Arial"]]
```

Export the cophenetic correlation plot.

In[•]:=
```
filenameout = SystemDialogInput["FileSave"];
Export[filenameout, ccPlot, "TIFF", ImageResolution → 150]
```

Out[•]=
```
/Users/nm/Projects/Storage/MacLeod/Meetings/2018/06
  China Trip/Presentations/Short Course/GBDB
  Morphometrics Workshop/Data Analysis Results/Procrustes
  Alignment/Outlines/PCA Results/Cophenetic Correlation Plot.tif
```

Display PC score distances from origin ( = mean ).

```
distTable = Table[" ", {n2 + 1}, {2}];
origin = Table[0.0, {noAxes}];
distTable[[1, 1]] = "Objects";
distTable[[1, 2]] = "Distance From Origin";
rScores = Take[eScores, All, noAxes];
Do[distTable[[i + 1, 1]] = objNames[[i]];
 distTable[[i + 1, 2]] = EuclideanDistance[origin, rScores[[i]]], {i, n2}]
Labeled[Grid[distTable, BaseStyle → (FontFamily → "Arial"),
  Alignment → {{Left, Center}}, Frame → True,
  Dividers → {{True, True}, {True, True}}], "Distance Table", Top,
 LabelStyle → Directive[Black, Bold, FontSize → 14, FontFamily → "Arial"]]
```

Export distances from origin ( = mean) .

```
filenameout = SystemDialogInput["FileSave"];
Export[filenameout, distTable, "CSV", "TextDelimiters" → ""]
```

Detect groups based on local optimization (optional).

Specify group inference method (if applicable)

In[•]:=
```
Panel[
 Labeled[Column[{Panel[Labeled[PopupMenu[Dynamic[klustMeth], {1 → "Automatic",
        2 → "Single-linkage clustering", 3 → "Density-based spatial clustering",
        4 → "High-density region displacement",
        5 → "Jarvis-Patrick clustering", 6 → "k-means clustering",
        7 → "Mean-shift clustering", 8 → "Medoid partitioning",
        9 → "Minimum spanning tree clustering", 10 → "Spectral clustering",
        11 → "Variational Gaussian mixture clustering"}],
      "Select data clustering method.", Top, LabelStyle →
       Directive[FontSize → 12, Bold, FontFamily → "Arial"]]],
     Row[{Panel[Labeled[InputField[Dynamic[noVar], FieldSize → 5],
        "Enter number of varables to include.", Top,
        LabelStyle → Directive[FontSize → 12, Bold, FontFamily → "Arial"]]],
      "  ", Panel[Labeled[InputField[Dynamic[ng], FieldSize → 5],
        "Enter number of groups (if applicable).", Top,
        LabelStyle → Directive[FontSize → 12, Bold, FontFamily → "Arial"]]]}]},
   Center], "Data Clustering Options", Top, LabelStyle →
   Directive[FontSize → 18, Bold, FontFamily → "Arial"]]]
klustMeth = 1; noVar = noAxes; ng = 3;
```

Out[•]=

**Data Clustering Options**

**Select data clustering method.**

Automatic

**Enter number of varables to include.**

noVar

**Enter number of groups (if applicable).**

ng

## Perform data clustering (optional).

In[∘]:=
```
If[klustMeth == 1, meth = Automatic];
If[klustMeth == 2, meth = "Agglomerate"];
If[klustMeth == 3, meth = "DBSCAN"];
If[klustMeth == 4, meth = "NeighborhoodContraction"];
If[klustMeth == 5, meth = "JarvisPatrick"];
If[klustMeth == 6, meth = "KMeans"];
If[klustMeth == 7, meth = "MeanShift"];
If[klustMeth == 8, meth = "KMedoids"];
If[klustMeth == 9, meth = "SpanningTree"];
If[klustMeth == 10, meth = "Spectral"];
If[klustMeth == 11, meth = "GaussianMixture"];

eScrs = Take[eScores, All, noAxes];
If[klustMeth == 6 || klustMeth == 8,
  optGroups = FindClusters[eScrs, ng, Method → meth],
  optGroups = FindClusters[eScrs, Method → meth, PerformanceGoal → "Quality"]];
clustDim = Dimensions[optGroups];

Panel[Labeled[Panel[Labeled[InputField[Dynamic[clustDim⟦1⟧], FieldSize → 5],
    "No. of clusters identified:", Top,
    LabelStyle → Directive[FontSize → 12, Bold, FontFamily → "Arial"]]],
  "Data Clustering Results", Top, LabelStyle →
   Directive[FontSize → 16, Bold, FontFamily → "Arial"]]]
```

Out[∘]=

**Data Clustering Results**

**No. of clusters identified:**

clustDim⟦1⟧

Accept data clustering result (optional).

In[∘]:=
```
optGpNames = Table[" ", {n2}];
Do[
 {n3, m3} = Dimensions[optGroups⟦k⟧];
 l1 = StringJoin["Group ", ToString[k]];
 Do[
  Do[
   If[optGroups⟦k, j⟧ == eScrs⟦i⟧, optGpNames⟦i⟧ = l1], {i, n2}], {j, n3}],
 {k, clustDim⟦1⟧}]
kg = clustDim⟦1⟧;
numGroups = kg;
Group = optGpNames;
```

Plot single – axis histogram.

Specify single – variable plot (histogram) options.

In[∘]:=
```
pcNames = Table[StringJoin["PC-", ToString[i]], {i, noAxes}];
Panel[Labeled[Column[{
    Row[{Panel[Labeled[PopupMenu[Dynamic[axisName], pcNames],
        "Select component to be plotted.", Top,
        LabelStyle → Directive[FontSize → 12, Bold, FontFamily → "Arial"]]],
      "  ", Panel[Labeled[PopupMenu[Dynamic[histType],
         {1 → "Stacked", 2 → "Overlapped"}], "Select histogram type.", Top,
        LabelStyle → Directive[FontSize → 12, Bold, FontFamily → "Arial"]]]}],
    Row[{Panel[Labeled[InputField[Dynamic[noBins], FieldSize → 5],
        "Enter no. of histogram bins.", Top, LabelStyle →
         Directive[FontSize → 12, Bold, FontFamily → "Arial"]]], "  ",
      Panel[Labeled[InputField[Dynamic[hSize], FieldSize → 5],
        "Enter histogram plot size.", Top,
        LabelStyle → Directive[FontSize → 12, Bold, FontFamily → "Arial"]]]}]},
   Center], "Single Axis (Histogram) Plot Options", Top,
  LabelStyle → Directive[FontSize → 16, Bold, FontFamily → "Arial"]]]
axisName = pcNames⟦1⟧; noBins = 15; hSize = 500; histType = 1;
```

Out[∘]=

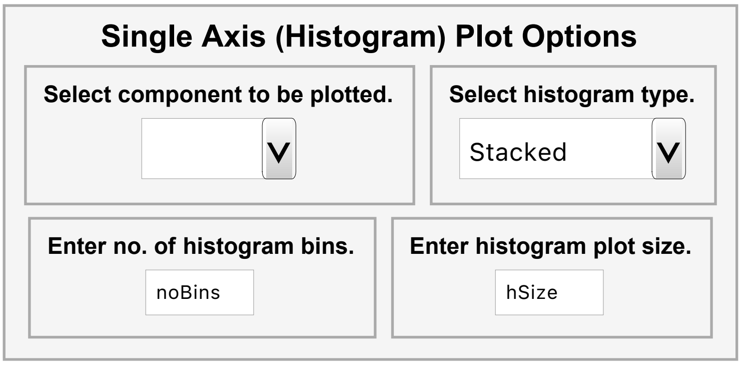


Constrruct and display histogram plot .

```
In[•]:= Do[If[axisName == pcNames[[j]], axis = j], {j, noAxes}];
pltScores = Flatten[Take[eScores, All, {axis}]];
If[histType == 1, htype = "Stacked", htype = "Overlapped"];
gpNames = Union[Group];
groupPosns = Table[Flatten[Position[Group, gpNames[[i]], 1]], {i, numGroups}];
pltPoints = Table[pltScores[[groupPosns[[j]]]], {j, numGroups}];
hueList = Table[Hue[N[((numGroups + 1) - j) / numGroups]], {j, numGroups}];
h1 =
 Labeled[Histogram[pltPoints, noBins, ChartStyle → {hueList}, ChartLayout → htype,
   LabelStyle → Directive[FontSize → 12, Black, FontFamily → "Arial"],
   AxesLabel → {"PC Score", "Frequency"}, ImageSize → hSize,
   ChartLegends → gpNames], StringJoin["Principal Component ", ToString[axis]],
  Top, LabelStyle → Directive[FontSize → 16, Bold, FontFamily → "Arial"]]
```

Export current histogram.

```
filenameout = SystemDialogInput["FileSave"];
Export[filenameout, h1, "TIFF", ImageResolution → 150]
/Users/nm/Desktop/Varotis Results/CVA Results/CV-1.tif
```

Create 2 D scatterplot .

Specify 2 D plot options.
You must run this code after you read in the data so it can pick up the proper variable names.

```
In[•]:= pcNames = Table[StringJoin["PC-", ToString[i]], {i, noAxes}];
Panel[Labeled[
  Column[{Row[{Panel[Labeled[PopupMenu[Dynamic[xAxisName], pcNames],
        "Select variable to be plotted on x-Axis.", Top, LabelStyle →
         Directive[FontSize → 12, Bold, FontFamily → "Arial"]]], "  ",
      Panel[Labeled[PopupMenu[Dynamic[yAxisName], pcNames],
        "Select variable to be plotted on y-Axis.", Top,
        LabelStyle → Directive[FontSize → 12, Bold, FontFamily → "Arial"]]]}],
Row[{
      Panel[Labeled[PopupMenu[Dynamic[pltAspect],
         {1 → "Golden Ratio Plot", 2 → "Square Plot (equi-length axes)",
          3 → "True-Scale Plot (actual axis scales)"}],
        "Enter plot aspect ratio type.", Top, LabelStyle →
         Directive[FontSize → 12, Bold, FontFamily → "Arial"]]], "  ",
      Panel[Labeled[PopupMenu[Dynamic[lch], {1 → "Simple scatterplot",
          2 → "Scatterplot w/ convex hulls"}],
        "Show group domians?", Top, LabelStyle →
         Directive[FontSize → 12, Bold, FontFamily → "Arial"]]], "  ",
      Panel[Labeled[PopupMenu[Dynamic[ptsJoin], {1 → "No", 2 → "Yes"}],
        "Join datapoints?", Top, LabelStyle →
         Directive[FontSize → 12, Bold, FontFamily → "Arial"]]], "  ",
      Panel[Labeled[PopupMenu[Dynamic[rq], {1 → "R-Score Plot",
          2 → "R-Q Biplot"}], "Specify plot type.", Top,
        LabelStyle → Directive[FontSize → 12, Bold, FontFamily → "Arial"]]]}],
Row[{Panel[Labeled[InputField[Dynamic[pltSize], FieldSize → 10],
        "Enter plot size value.", Top,
        LabelStyle → Directive[FontSize → 12, Bold, FontFamily → "Arial"]]],
      "  ", Panel[Labeled[InputField[Dynamic[pltPad], FieldSize → 10],
        "Enter plot margin padding value.", Top, LabelStyle →
         Directive[FontSize → 12, Bold, FontFamily → "Arial"]]], "  ",
      Panel[Labeled[InputField[Dynamic[iconSize], FieldSize → 10],
        "Enter plot icon size value.", Top, LabelStyle →
         Directive[FontSize → 12, Bold, FontFamily → "Arial"]]]}]}, Center],
  "2D Plot Options", Top, LabelStyle → Directive[FontSize → 18,
    Bold, FontFamily → "Arial"]]]
pltSize = 500; iconSize = 0.025; pltPad = 0.1; xAxisName = pcNames⟦1⟧;
yAxisName = pcNames⟦2⟧;
 ptsJoin = 1; rq = 1; dataTrans = 1;  pltAspect = 1 ; lch = 2;
```

Out[∘]=

## 2D Plot Options

| Select variable to be plotted on x–Axis. | Select variable to be plotted on y–Axis. |
|---|---|
| [ ] V | [ ] V |

Enter plot aspect ratio type.
Golden Ratio Plot V

| Show group domians? | Join datapoints? | Specify plot type. |
|---|---|---|
| Simple scatterplot V | No V | R–Score Plot V |

| Enter plot size value. | Enter plot margin padding value. | Enter plot icon size value. |
|---|---|---|
| pltSize | pltPad | iconSize |

### Plot script

In[∘]:=

```
pltTable = Table[" ", {numGroups}, {3}];
Do[If[xAxisName == pcNames[[j]], axis1 = j], {j, noAxes}];
Do[If[yAxisName == pcNames[[j]], axis2 = j], {j, noAxes}];

groupNames = Union[Group];
numGroups = Length[groupNames];
groupPosns = Table[Flatten[Position[Group, groupNames[[i]], 1]], {i, numGroups}];
eScoresT = Transpose[eScores];

xAxis = eScoresT[[axis1]]; yAxis = eScoresT[[axis2]];
lab1 = StringJoin[{"Principal Component ", ToString[axis1]},
   {" (Var. =", ToString[t3[[axis1 + 1, 3]]]}, {"%)"}];
lab2 = StringJoin[{"Principal Component ", ToString[axis2]},
   {" (Var. =", ToString[t3[[axis2 + 1, 3]]]}, {"%)"}];
maxx =
 Max[
  xAxis];
minx = Min[xAxis];
maxy = Max[yAxis];
miny = Min[yAxis];

If[pltAspect == 1 || pltAspect == 3,
  xPlotLow = minx; xPlotHi = maxx; yPlotLow = miny; yPlotHi = maxy];
If[pltAspect == 2,
  If[minx > miny,
   xPlotLow = miny; yPlotLow = miny,
```

```
    xPlotLow = minx; yPlotLow = minx ]];
If[pltAspect == 2,
  If[maxx < maxy,
   xPlotHi = maxy; yPlotHi = maxy,
   xPlotHi = maxx; yPlotHi = maxx]];
If[pltAspect == 1, aRatio = 1 / N[GoldenRatio]];
If[pltAspect == 2, aRatio = 1];
If[pltAspect == 3, aRatio = Automatic];

tmpPoints = Transpose[List[xAxis, yAxis]];
pltPoints = Table[tmpPoints[[groupPosns[[j]]]], {j, numGroups}];
iconList = Flatten[Table[
    {Graphics[{EdgeForm[{Thickness[0.005], Black}],
       Hue[N[((numGroups + 1) - j) / numGroups]],
       Disk[{0, 0}, Scaled[iconSize]]}]}, {j, numGroups}]];

If[lch == 1 || ptsJoin == 1,
 Do[
  pltTable[[k, 1]] =
   ListPlot[pltPoints[[k]], AspectRatio → aRatio, Frame → True, Joined → False,
    Axes → False, PlotRange → {{xPlotLow, xPlotHi}, {yPlotLow, yPlotHi}},
    PlotRangePadding → Scaled[pltPad], Ticks → Automatic, FrameLabel →
     {lab1, lab2}, PlotMarkers → iconList[[k]], ImageSize → pltSize, LabelStyle →
     Directive[FontSize → 14, Black, FontFamily → "Arial"]], {k, numGroups}],
 Do[
  pltTable[[k, 1]] =
   ListPlot[pltPoints[[k]], Frame → True, Axes → False, AspectRatio → aRatio,
    PlotRange → {{xPlotLow, xPlotHi}, {yPlotLow, yPlotHi}}, PlotRangePadding →
     Scaled[pltPad], Ticks → Automatic, FrameLabel → {lab1, lab2},
    LabelStyle → Directive[Black, FontSize → 14, FontFamily → "Arial"],
    ImageSize → pltSize, PlotStyle → Directive[Disk[],
      Hue[N[((numGroups + 1) - k) / numGroups]], EdgeForm[{Thickness[1.0], Black}],
      PointSize[Scaled[iconSize - 0.009]]]], {k, numGroups}]]

If[ptsJoin == 2,
  Do[
   pltTable[[k, 2]] = ListLinePlot[pltPoints[[k]],
     AspectRatio → aRatio, Frame → True, Joined → True, Axes → False,
     PlotStyle → Directive[Hue[N[((numGroups + 1) - k) / numGroups]], Thin],
     PlotRange → {{xPlotLow, xPlotHi}, {yPlotLow, yPlotHi}},
     PlotRangePadding → Scaled[pltPad], Ticks → Automatic,
     FrameLabel → {lab1, lab2}, ImageSize → pltSize, LabelStyle →
      Directive[FontSize → 14, Black, FontFamily → "Arial"]], {k, numGroups}]];

If[lch == 2,
  Do[
   hull = ConvexHullMesh[pltPoints[[k]]];
```

```
    pltTable⟦k, 3⟧ = HighlightMesh[hull,
      Style[2, Opacity[0.2], Hue[N[((numGroups + 1) - k) / numGroups]]],
      Frame → True, Axes → False, AspectRatio → aRatio,
      PlotRange → {{xPlotLow, xPlotHi}, {yPlotLow, yPlotHi}}, PlotRangePadding →
       Scaled[pltPad], Ticks → Automatic, FrameLabel → {lab1, lab2},
      LabelStyle → Directive[Black, FontSize → 14, FontFamily → "Arial"],
      ImageSize → pltSize], {k, numGroups}]];

vecTable = Table[" ", {m2}];
comp = Table[0.0, {4}];
comp⟦1⟧ = xPlotHi / Max[Flatten[Take[eVecs, All, {axis1}]]];
comp⟦2⟧ = xPlotLow / Min[Flatten[Take[eVecs, All, {axis1}]]];
comp⟦3⟧ = yPlotHi / Max[Flatten[Take[eVecs, All, {axis2}]]];
comp⟦4⟧ = yPlotLow / Min[Flatten[Take[eVecs, All, {axis2}]]];
eVecsSF = Min[comp];
Do[
 vecTable⟦k⟧ = Graphics[{Black, Arrowheads[0.025],
     Arrow[{{0.0, 0.0}, {eVecs⟦k, axis1⟧ * eVecsSF, eVecs⟦k, axis2⟧ * eVecsSF}}]},
   AspectRatio → aRatio, Frame → True, Axes → False,
   PlotRange → {{xPlotLow, xPlotHi}, {yPlotLow, yPlotHi}},
   PlotRangePadding → Scaled[pltPad], FrameLabel → {lab1, lab2},
   Ticks → Automatic, ImageSize → pltSize,
   LabelStyle → Directive[Black, FontSize → 14, FontFamily → "Arial"]], {k, m2}]
vecPlt = Show[vecTable];

If[ptsJoin == 1 && lch == 1 && rq == 1, p0 = Show[pltTable⟦All, 1⟧]];
If[ptsJoin == 2 && lch == 1 && rq == 1,
  p0 = Show[pltTable⟦All, 2⟧, pltTable⟦All, 1⟧]];
If[ptsJoin == 1 && lch == 2 && rq == 1, p0 = Show[pltTable⟦All, 3⟧, pltTable⟦All, 1⟧]];
If[ptsJoin == 2 && lch == 2 && rq == 1,
  p0 = Show[pltTable⟦All, 3⟧, pltTable⟦All, 2⟧, pltTable⟦All, 1⟧]];
If[ptsJoin == 1 && lch == 1 && rq == 2, p0 = Show[vecPlt, pltTable⟦All, 1⟧]];
If[ptsJoin == 2 && lch == 1 && rq == 2,
  p0 = Show[vecPlt, pltTable⟦All, 2⟧, pltTable⟦All, 1⟧]];
If[ptsJoin == 1 && lch == 2 && rq == 2,
  p0 = Show[vecPlt, pltTable⟦All, 3⟧, pltTable⟦All, 1⟧]];
If[ptsJoin == 2 && lch == 2 && rq == 2,
  p0 = Show[vecPlt, pltTable⟦All, 3⟧, pltTable⟦All, 2⟧, pltTable⟦All, 1⟧]];

p1 = Labeled[p0, "           PC Score Plot", Top,
   LabelStyle → Directive[FontSize → 18, Bold, FontFamily → "Arial"]];
g1 = Grid[Table[
     {Graphics[{EdgeForm[{Thin, Black}], Hue[N[((numGroups + 1) - j) / numGroups]],
        Disk[]}, ImageSize → 13]}, {j, numGroups}], Frame → False];
g2 = Grid[Partition[groupNames, 1], Alignment → Left,
   BaseStyle → {FontFamily → "Arial", FontSize → 14
     , Italic}];
```

```
p2 = Labeled[Text[Grid[{{g1, g2}}, Alignment → Bottom, Frame → True]], "Legend",
    Top, LabelStyle → Directive[Black, FontSize → 18, Bold, FontFamily → "Arial"]];

plt2D = Grid[{{p1, p2}}, BaselinePosition → Top, Alignment → Top]
```

Export current 2 D plot.

In[∘]:=
```
filenameout = SystemDialogInput["FileSave"];
Export[filenameout, plt2D, "TIFF", ImageResolution → 150]
```

Out[∘]=
```
/Users/n.macleod/Desktop/Wesy Lyons/PC-3 vs PC-2.tif
```

Specify 2 D plot labeling options.

In[∘]:=
```
Panel[Labeled[
  Column[{Panel[Labeled[PopupMenu[Dynamic[ptLabOpt], {1 → "Label objects/cases",
        2 → "Label variable axes", 3 → "Label objects/cases & variable axes"}],
      "Select point label option.", Top, LabelStyle →
       Directive[FontSize → 12, Bold, FontFamily → "Arial"]]],
    Row[{Panel[Labeled[InputField[Dynamic[labPtSz], FieldSize → 10],
        "Specify label point size.", Top, LabelStyle →
         Directive[FontSize → 12, Bold, FontFamily → "Arial"]]], "   ",
      Panel[Labeled[InputField[Dynamic[offSet], FieldSize → 10],
        "Specify label offset factor.", Top,
        LabelStyle → Directive[FontSize → 12, Bold, FontFamily → "Arial"]]]}]},
   Center], "2D Plot Labeling Options", Top, LabelStyle →
   Directive[FontSize → 18, Bold, FontFamily → "Arial"]]]
ptLabOpt = 1; labPtSz = 10; offSet = 15;
```

Out[∘]=

**2D Plot Labeling Options**

**Select point label option.**

Label objects/cases

**Specify label point size.** labPtSz

**Specify label offset factor.** offSet

Label plotted points.

In[]:=
```
pn1 = p0;
namePoints = tmpPoints;
tempPointsT = Transpose[tmpPoints];
mxY = Max[tempPointsT[[2]]];
mnY = Min[tempPointsT[[2]]];
incY = N[(mxY - mnY) / offSet];
Do[namePoints[[i, 2]] = tmpPoints[[i, 2]] - incY, {i, n2}]

nPointsTable = Table[{Text[objNames[[i]], namePoints[[i]], {-1, 0}]}, {i, n2}];
vecs = Table[0.0, {m2}, {2}];
Do[vecs[[i, 1]] = eVecs[[i, axis1]] * eVecsSF;
 vecs[[i, 2]] = eVecs[[i, axis2]] * eVecsSF, {i, m2}]
vecPoints = vecs;
Do[
 If[vecPoints[[i, 2]] ≥ 0.0, vecPoints[[i, 2]] = vecPoints[[i, 2]] + incY,
  vecPoints[[i, 2]] = vecPoints[[i, 2]] - incY], {i, m2}]
vPointsTable = Table[Text[Style[varNames[[i]], Blue], vecPoints[[i]]], {i, m2}];

If[ptLabOpt == 1 || ptLabOpt == 3,
  pn2 = Graphics[nPointsTable, Frame → True, AspectRatio → aRatio , Axes → False,
    FrameLabel → {lab1, lab2}, PlotRangePadding → Scaled[pltPad],
    BaseStyle → Directive[FontSize → 12, Black, FontFamily → "Arial"],
    ImageSize → pltSize]];
If[ptLabOpt == 2 || ptLabOpt == 3, pv2 = Graphics[vPointsTable,
    Frame → True, AspectRatio → aRatio , Axes → False,
    FrameLabel → {lab1, lab2}, PlotRangePadding → Scaled[pltPad],
    BaseStyle → Directive[FontSize → 12, Black, FontFamily → "Arial"],
    ImageSize → pltSize]];
If[ptLabOpt == 1, p1 = Labeled[Show[{pn1, pn2}, BaseStyle → {FontFamily → "Arial"}],
    "          PC Score Plot", Top,
    LabelStyle → Directive[FontSize → 18, Bold, FontFamily → "Arial"]]];
If[ptLabOpt == 2, p1 = Labeled[Show[{pn1, pv2}, BaseStyle → {FontFamily → "Arial"}],
    "          PC Score Plot", Top,
    LabelStyle → Directive[FontSize → 18, Bold, FontFamily → "Arial"]]];
If[ptLabOpt == 3, p1 = Labeled[Show[{pn1, pn2, pv2},
     BaseStyle → {FontFamily → "Arial"}], "          PC Score Plot", Top,
    LabelStyle → Directive[FontSize → 18, Bold, FontFamily → "Arial"]]];

plt2D = Grid[{{p1, p2}}, BaselinePosition → Top, Alignment → Top]
```

Export current 2 D plot.

In[•]:=
```
filenameout = SystemDialogInput["FileSave"];
Export[filenameout, plt2D, "TIFF", ImageResolution → 150]
```

Out[•]=
```
/Users/n.macleod/Desktop/Drangonflies (Final)/Data & Results/GM/
  Hindwings/PCA Results/PC-3 vs PC-2 (Landscape Groups w: Labels).tif
```

Create 3 D scatterplot.

Specify 3 D plot options.
You must run this code after you read in the data so it can pick up the proper variable names.

```
In[•]:= pcNames = Table[StringJoin["PC-", ToString[i]], {i, noAxes}];
Panel[
 Labeled[Column[{Row[{Panel[Labeled[PopupMenu[Dynamic[xAxisName], pcNames],
        "Select variable to be plotted on x-Axis.", Top, LabelStyle →
         Directive[FontSize → 12, Bold, FontFamily → "Arial"]]], "  ",
      Panel[Labeled[PopupMenu[Dynamic[yAxisName], pcNames],
        "Select variable to be plotted on y-Axis.", Top, LabelStyle →
         Directive[FontSize → 12, Bold, FontFamily → "Arial"]]], "  ",
      Panel[Labeled[PopupMenu[Dynamic[zAxisName], pcNames],
        "Select variable to be plotted on z-Axis.", Top,
        LabelStyle → Directive[FontSize → 12, Bold, FontFamily → "Arial"]]]}],
    Row[{
      Panel[Labeled[PopupMenu[Dynamic[pltAspect],
          {1 → "Golden Ratio Plot", 2 → "Square Plot (equi-length axes)",
           3 → "True-Scale Plot (actual axis scales)"}],
        "Enter plot aspect ratio type.", Top, LabelStyle →
         Directive[FontSize → 12, Bold, FontFamily → "Arial"]]], "  ",
      Panel[Labeled[PopupMenu[Dynamic[lch], {1 → "Simple scatterplot",
           2 → "Scatterplot w/ convex hulls"}],
        "Show group domians?", Top, LabelStyle →
         Directive[FontSize → 12, Bold, FontFamily → "Arial"]]], " ",
      Panel[Labeled[PopupMenu[Dynamic[ptsJoin], {1 → "No", 2 → "Yes"}],
        "Join datapoints?", Top,
        LabelStyle → Directive[FontSize → 12, Bold, FontFamily → "Arial"]]],
      "  ", Panel[Labeled[PopupMenu[Dynamic[rq],
          {1 → "R-Score Plot", 2 → "R-Q Biplot"}], "Specify plot type.", Top,
        LabelStyle → Directive[FontSize → 12, Bold, FontFamily → "Arial"]]]}],
    Row[{Panel[Labeled[InputField[Dynamic[pltSize], FieldSize → 5],
        "Enter plot size value.", Top,
        LabelStyle → Directive[FontSize → 12, Bold, FontFamily → "Arial"]]],
      " ", Panel[Labeled[InputField[Dynamic[pltPad], FieldSize → 5],
        "Enter plot margin padding value.", Top, LabelStyle →
         Directive[FontSize → 12, Bold, FontFamily → "Arial"]]], " ",
      Panel[Labeled[InputField[Dynamic[icSz3D], FieldSize → 5],
        "Enter plot icon size value.", Top, LabelStyle →
         Directive[FontSize → 12, Bold, FontFamily → "Arial"]]]}]}, Center],
  "3D Plot Options", Top, LabelStyle → Directive[FontSize → 18,
    Bold, FontFamily → "Arial"]]]
pltSize = 500; icSz3D = 50; pltPad = 0.1; xAxisName = pcNames⟦1⟧;
yAxisName = pcNames⟦2⟧;
zAxisName = pcNames⟦3⟧; ptsJoin = 1;
dataTrans = 1; pltAspect = 3 ;
lch = 2;
rq = 1;
```

Out[∘]=

**3D Plot Options**

**Select variable to be plotted on x–Axis.**

**Select variable to be plotted on y–Axis.**

**Select variable to be plotted on z–Axis.**

**Enter plot aspect ratio type.**

Golden Ratio Plot

**Show group domians?**

Simple scatterplot

**Join datapoints?**

No

**Specify plot type.**

R–Score Plot

**Enter plot size value.**

pltSize

**Enter plot margin padding value.**

pltPad

**Enter plot icon size value.**

icSz3D

## Plot script

In[∘]:=

```
Do[If[xAxisName == pcNames⟦j⟧, axis1 = j], {j, noAxes}]
Do[If[yAxisName == pcNames⟦j⟧, axis2 = j], {j, noAxes}]
Do[If[zAxisName == pcNames⟦j⟧, axis3 = j], {j, noAxes}]

groupNames = Union[Group];
numGroups = Length[groupNames];
groupPosns = Table[Flatten[Position[Group, groupNames⟦i⟧, 1]], {i, numGroups}];
eScoresT = Transpose[eScores];

xAxis = eScoresT⟦axis1⟧;
yAxis = eScoresT⟦axis2⟧;
zAxis = eScoresT⟦axis3⟧;
lab1 = StringJoin["PC-", ToString[axis1]];
lab2 = StringJoin["PC-", ToString[axis2]];
lab3 = StringJoin["PC-", ToString[axis3]];
maxx = Max[xAxis];
minx = Min[xAxis];
maxy = Max[yAxis];
miny = Min[yAxis];
maxz = Max[zAxis]; minz = Min[zAxis];

mxax = Max[xAxis];
```

```
mnax = Min[xAxis];
If[pltAspect < 3, f = N[icSz3D * 0.020]];
If[pltAspect == 3, f = N[icSz3D * 0.0005]];
iconSize3D = (mxax - mnax) * f;

If[pltAspect == 1 || pltAspect == 3,
  xPlotLow = minx; xPlotHi = maxx; yPlotLow = miny; yPlotHi = maxy];
If[pltAspect == 2,
  If[minx > miny,
   xPlotLow = miny; yPlotLow = miny,
   xPlotLow = minx; yPlotLow = minx ]];
If[pltAspect == 2,
  If[maxx < maxy,
   xPlotHi = maxy; yPlotHi = maxy,
   xPlotHi = maxx; yPlotHi = maxx]];

If[pltAspect == 1, bRatio = {1.61803, 1, 1}];
If[pltAspect == 2, bRatio = {1, 1, 1}];
If[pltAspect == 3, bRatio = Automatic];

points3 = Transpose[List[xAxis, yAxis, zAxis]];
gp1 = groupPosns;
h = Table[0, {n2}];
Do[
 tmp = gp1[[i]];
 itr = Length[tmp];
 Do[h[[tmp[[j]]]] = Hue[N[((numGroups + 1) - i) / numGroups]], {j, itr}],
 {i, numGroups}]

If[pltAspect == 3,
  pltPoints = Table[{h[[i]], Sphere[points3[[i]], iconSize3D]}, {i, n2}];
  p0 = Graphics3D[pltPoints, Axes → True,
    Boxed → True, PlotRangePadding → Scaled[pltPad],
    LabelStyle → Directive[FontSize → 12, Black, FontFamily → "Arial"],
    AxesLabel → {lab1, lab2, lab3}, ImageSize → pltSize, BoxRatios → Automatic]];
If[pltAspect ≠ 3,
  pltPoints =
   Table[{h[[i]], AbsolutePointSize[iconSize3D], Point[points3[[i]]]}, {i, n2}];
  p0 = Graphics3D[pltPoints, Axes → True, Boxed → True,
    PlotRangePadding → Scaled[pltPad],
    LabelStyle → Directive[FontSize → 12, Black, FontFamily → "Arial"],
    AxesLabel → {lab1, lab2, lab3}, ImageSize → pltSize, BoxRatios → bRatio]];
If[ptsJoin == 2,
  pltLineTable = Table[" ", {numGroups}];
  gpPoints = Table[points3[[groupPosns[[j]]]], {j, numGroups}];
  Do[
   pltLineTable[[k]] =
```

```
      Graphics3D[{Hue[N[((numGroups + 1) - k) / numGroups]], Line[gpPoints⟦k⟧]},
       Axes → True, Boxed → True, PlotRangePadding → Scaled[pltPad],
       LabelStyle → Directive[FontSize → 12, Black, FontFamily → "Arial"],
       AxesLabel → {lab1, lab2, lab3}, ImageSize → pltSize,
       BoxRatios → bRatio, ViewPoint → {xax, yax, zax}], {k, numGroups}];
    p0 = Show[{pltLineTable, p0}]];
  If[lch == 2,
    pltMeshTable = Table[" ", {numGroups}];
    gpPoints = Table[points3⟦groupPosns⟦j⟧⟧, {j, numGroups}];
    Do[
     {n3, m3} = Dimensions[gpPoints⟦k⟧];
     cHull3D = ConvexHullMesh[gpPoints⟦k⟧, BaseStyle → {EdgeForm[]},
       Boxed → True, Axes → True, PlotRangePadding → Scaled[pltPad],
       LabelStyle → Directive[FontSize → 12, Black, FontFamily → "Arial"],
       AxesLabel → {lab1, lab2, lab3}, ImageSize → pltSize,
       BoxRatios → bRatio, ViewPoint → {xax, yax, zax}];
     pltMeshTable⟦k⟧ = Show[{HighlightMesh[cHull3D, Style[2, Opacity[0.2],
          Hue[N[((numGroups + 1) - k) / numGroups]]]], p0}], {k, numGroups}];
    p0 = Show[pltMeshTable]];
  If[rq > 1,
    vecTable = Table[0.0, {m2}, {3}];
    vecPltTable = Table[" ", {m2}];
    Do[
     vecTable⟦i, 1⟧ = eVecs⟦i, axis1⟧;
     vecTable⟦i, 2⟧ = eVecs⟦i, axis2⟧;
     vecTable⟦i, 3⟧ = eVecs⟦i, axis3⟧, {i, m2}];
    xPlotHi = Max[Take[points3, All, {axis1}]];
    xPlotLo = Min[Take[points3, All, {axis1}]];
    yPlotHi = Max[Take[points3, All, {axis2}]];
    yPlotLo = Min[Take[points3, All, {axis2}]];
    zPlotHi = Max[Take[points3, All, {axis3}]];
    zPlotLo = Min[Take[points3, All, {axis3}]];
    comp3 = Table[0.0, {6}];
    comp3⟦1⟧ = xPlotHi / Max[Flatten[Take[vecTable, All, {1}]]];
    comp3⟦2⟧ = xPlotLo / Min[Flatten[Take[vecTable, All, {1}]]];
    comp3⟦3⟧ = xPlotHi / Max[Flatten[Take[vecTable, All, {2}]]];
    comp3⟦4⟧ = xPlotLo / Min[Flatten[Take[vecTable, All, {2}]]];
    comp3⟦5⟧ = xPlotHi / Max[Flatten[Take[vecTable, All, {3}]]];
    comp3⟦6⟧ = xPlotLo / Min[Flatten[Take[vecTable, All, {3}]]];
    eVecs3DSF = Min[comp3] / 2;
    vecTable = vecTable * eVecs3DSF;
    Do[
     vecPltTable⟦i⟧ =
      Graphics3D[{Arrowheads[0.005], Arrow[{{0, 0, 0}, vecTable⟦i⟧}]}], {i, m2}];
    vecPlt3D = Show[vecPltTable];
    p0 = Show[{p0, vecPlt3D}]];
```

```
p1 = Labeled[p0, "           PC Score Plot", Top,
   LabelStyle → Directive[FontSize → 18, Bold, FontFamily → "Arial"]];
h2 = Table[0, {numGroups}];
l1 = Table[" ", {numGroups}, {2}];
horiz = Table["Left", numGroups];
vert = Table["Center", numGroups];
Do[h2⟦i⟧ = Hue[N[((numGroups + 1) - i) / numGroups]], {i, numGroups}];
If[pltAspect == 3,
  i1 = Table[{h2⟦i⟧, Sphere[{0, 0, 0}, 0.01]}, {i, numGroups}];
  Do[
   l1⟦i, 1⟧ = Graphics3D[i1⟦i⟧, Boxed → False, ImageSize → 16], {i, numGroups}]];
If[pltAspect ≠ 3,
 i1 = Table[{h2⟦i⟧, EdgeForm[{Directive[{Thickness[0.05]}]}],
    Disk[{0, 0}, 0.001]}, {i, numGroups}];
 Do[l1⟦i, 1⟧ = Graphics[i1⟦i⟧, ImageSize → 12], {i, numGroups}]]
Do[l1⟦i, 2⟧ = Style[groupNames⟦i⟧, FontFamily → "Arial",
    FontSlant → Italic, FontSize → 16], {i, numGroups}];
g2 = Labeled[Grid[l1, Frame → True, Alignment → {horiz, vert},
    Spacings → {1, 0.5}], "Legend", Top,
   LabelStyle → Directive[Black, FontSize → 16, Bold, FontFamily → "Arial"]];

plt3D = Grid[{{p1, g2}}, BaselinePosition → Top, Alignment → Top]
```

Adjust orientation of 3 D plot (if necessary).
You must replot the data to activate the changes. These changes will be able to be exported using the script below.

In[∘]:=
```
Panel[
 Labeled[Row[{Labeled[Slider[Dynamic[xax], {-10, 10}, Appearance → "Labeled"],
      "x-Axis Viewpoint", Top,
      LabelStyle → Directive[FontSize → 10, Bold, FontFamily → "Arial"]] ×
     Labeled[Slider[Dynamic[yax], {-10, 10}, Appearance → "Labeled"],
      "y-Axis Viewpoint", Top,
      LabelStyle → Directive[FontSize → 10, Bold, FontFamily → "Arial"]] ×
     Labeled[Slider[Dynamic[zax], {-10, 10}, Appearance → "Labeled"],
      "z-Axis Viewpoint", Top,
      LabelStyle → Directive[FontSize → 10, Bold, FontFamily → "Arial"]]}],
  "3D Plot Orientation Controls", Top, LabelStyle →
   Directive[FontSize → 14, Bold, FontFamily → "Ariel"]]]
xax = 2.5; yax = -2.5; zax = 2.5;
```

Out[∘]=

**3D Plot Orientation Controls**

x–Axis Viewpoint: −10.

y–Axis Viewpoint: −10.

z–Axis Viewpoint: −10.

## Export current 3 D plot.

In[∘]:=
```
filenameout = SystemDialogInput["FileSave"];
Export[filenameout, plt3D, "TIFF", ImageResolution → 150]
```

Out[∘]= /Users/n.macleod/Desktop/Drangonflies (Final)/Data & Results/Images (PCA-CVA)/Hindwings/PCA Results/PC-1 vs PC-2 vs PC-3 (Water Body Groups).tif

# Export PCA results (optional).

## Export eigenvalue table (optional).

In[∘]:=
```
filenameout = SystemDialogInput["FileSave"];
Export[filenameout, t2, "CSV", "TextDelimiters" → ""]
```

Out[∘]= /Users/n.macleod/Desktop/Butterflies 2/Eigenvalues.csv

## Export eigenvector table (optional).

In[∘]:=
```
filenameout = SystemDialogInput["FileSave"];
Export[filenameout, eVecs, "CSV", "TextDelimiters" → ""]
```

Out[∘]= /Users/n.macleod/Desktop/Butterflies 2/Eigenvectors.csv

### Export PCA scores (optional).

In[•]:=
```
adata = Table[" ", {n2 + 1}, {noAxes + 2}];
adata⟦1, 1⟧ = "Object";
adata⟦1, 2⟧ = "Group";
Do[adata⟦i + 1, 1⟧ = objNames⟦i⟧, {i, n2}]
Do[adata⟦i + 1, 2⟧ = Group⟦i⟧, {i, n2}]
Do[
 adata⟦1, j + 2⟧ = StringJoin["PC-", ToString[j]], {j, 1, noAxes}]
Do[adata⟦i + 1, j + 2⟧ = eScores⟦i, j⟧, {i, n2}, {j, noAxes}]

filenameout = SystemDialogInput["FileSave"];
Export[filenameout, adata, "CSV", "TextDelimiters" → ""]
```

Out[•]=
```
/Users/n.macleod/Desktop/Butterflies 2/Eigenscores.csv
```

## Modelling Section (optional).

### Calculate evenly spaced models along a set of axes (optional).

#### Specify no. of model axes and no. of models to be calcuated per axis

In[•]:=
```
Panel[Labeled[Row[{Panel[Labeled[InputField[Dynamic[modelAxes], FieldSize → 5],
      "Enter no. of axes to be modeled.", Top,
      LabelStyle → Directive[FontSize → 12, FontFamily → "Arial"]]], , ,
    Panel[Labeled[InputField[Dynamic[modelsPerAxis], FieldSize → 5],
      "Enter no. models to be plotted per axis.", Top,
      LabelStyle → Directive[FontSize → 12, FontFamily → "Arial"]]]}],
  "Along-Axis Modeling Parameters", Top, LabelStyle →
   Directive[FontSize → 16, Bold, FontFamily → "Arial"]]]
modelsPerAxis = 5; modelAxes = 3;
```

Out[•]=

**Along–Axis Modeling Parameters**

Enter no. of axes to be modeled. modelA⋱ xes

Enter no. models to be plotted per axis. models⋱ PerAx⋱ is

### Calculate along – axis PCA model coordinates.

In[•]:=
```
modTable = Table[0.0, {modelAxes}, {modelsPerAxis}, {modelAxes}];
Do[
 Do[
  Do[modTable[[k, j, i]] = Mean[Take[eScores[[All, i]]], {j, modelsPerAxis}],
  {i, modelAxes}], {k, modelAxes}]
eScoresT = Transpose[eScores];
Do[
 xMax = Max[eScoresT[[k]]];
 xMin = Min[eScoresT[[k]]];
 xRng = xMax - xMin;
 xInt = xRng / (modelsPerAxis - 1);
 xBase = xMin - xInt;
 Do[modTable[[k, i, k]] = xBase + (xInt * i), {i, modelsPerAxis}], {k, modelAxes}]
modSet = 1;
```

Calculate evenly spaced models across a planar subspace (optional).

Specify subspace modelling options.

In[∘]:=
```
Panel[
 Labeled[Column[{Row[{Panel[Labeled[PopupMenu[Dynamic[xAxisName], pcNames],
       "Select principal component to be modeled on x-axis.", Top,
       LabelStyle → Directive[FontSize → 12, FontFamily → "Arial"]]], , ,
     Panel[Labeled[InputField[Dynamic[xModelNum], FieldSize → 5],
       "Enter no. of models along the x-axis.", Top,
       LabelStyle → Directive[FontSize → 12, FontFamily → "Arial"]]]}],
    Row[{Panel[Labeled[PopupMenu[Dynamic[yAxisName], pcNames],
       "Select principal component to be modeled on y-axis.", Top,
       LabelStyle → Directive[FontSize → 12, FontFamily → "Arial"]]], , ,
     Panel[Labeled[InputField[Dynamic[yModelNum], FieldSize → 5],
       "Enter no. of models along the y-axis.", Top,
       LabelStyle → Directive[FontSize → 12, FontFamily → "Arial"]]]}]}],
  "Subspace Modeling Parameters", Top, LabelStyle →
   Directive[FontSize → 18, Bold, FontFamily → "Arial"]]]
xAxisName = pcNames〚1〛;
yAxisName = pcNames〚2〛;
xModelNum = 5;
yModelNum = 4;
```

Out[∘]=

**Subspace Modeling Parameters**

Select principal component to be modeled on x-axis.
PopupMenu[CV−1, Table[PC−<>ToString[i], {i, mvecs}]]

Enter no. of models along the x-axis.
xModel⋱Num

Select principal component to be modeled on y-axis.
PopupMenu[CV−1, Table[PC−<>ToString[i], {i, mvecs}]]

Enter no. of models along the y-axis.
yModel⋱Num

## Perform subspace model location calculations

In[∘]:=
```
modTable = Table[0.0, {yModelNum}, {xModelNum}, {2}];
eScoresT = Transpose[eScores];
Do[If[xAxisName == pcNames[[j]], axis1 = j], {j, noAxes}]
Do[If[yAxisName == pcNames[[j]], axis2 = j], {j, noAxes}]
xMax = Max[eScoresT[[axis1]]];
xMin = Min[eScoresT[[axis1]]];
xRng = xMax - xMin;
xInt = xRng / (xModelNum - 1);
yMax = Max[eScoresT[[axis2]]];
yMin = Min[eScoresT[[axis2]]];
yRng = yMax - yMin;
yInt = yRng / (yModelNum - 1);

xBase = xMin - xInt;
yBase = yMin - yInt;
Do[
 yVal = yBase + (yInt * j);
 Do[modTable[[j, i, 1]] = xBase + (xInt * i);
  modTable[[j, i, 2]] = yVal, {i, xModelNum}], {j, yModelNum}]
modSet = 2;
```

Import external set of model coordinates
(total must match along – axis model specifications [above] and contain the complete variable set) .

```
filenamein = SystemDialogInput["FileOpen"];
modTable = Import[filenamein, "CSV"];
```

Plot model coordinates in the PC space.

Specify 2 D plot options.
You must run this code after you read in the data so it can pick up the proper variable names.

```
In[•]:= pcNames = Table[StringJoin["PC-", ToString[i]], {i, noAxes}];
Panel[Labeled[Column[
   {Panel[Labeled[RadioButtonBar[Dynamic[modSet], {1 → "Along-axis Models",
        2 → "Subspace models"}], "Enter type of model set.", Top,
      LabelStyle → Directive[FontSize → 12, FontFamily → "Arial"]]],
    Row[{Panel[Labeled[PopupMenu[Dynamic[xAxisName], pcNames],
        "Select variable to be plotted on x-Axis.", Top,
        LabelStyle → Directive[FontSize → 12, FontFamily → "Arial"]]], "  ",
      Panel[Labeled[PopupMenu[Dynamic[yAxisName], pcNames],
        "Select variable to be plotted on y-Axis.", Top,
        LabelStyle → Directive[FontSize → 12, FontFamily → "Arial"]]]}],
    Row[{
      Panel[Labeled[PopupMenu[Dynamic[pltAspect],
         {1 → "Golden Ratio Plot", 2 → "Square Plot (equi-length axes)",
          3 → "True-Scale Plot (actual axis scales)"}],
        "Enter plot aspect ratio type.", Top, LabelStyle →
         Directive[FontSize → 12, Bold, FontFamily → "Arial"]]], "  ",
      Panel[Labeled[PopupMenu[Dynamic[lch], {1 → "Simple scatterplot",
          2 → "Scatterplot w/ convex hulls"}],
        "Show group domians?", Top, LabelStyle →
         Directive[FontSize → 12, Bold, FontFamily → "Arial"]]], "  ",
      Panel[Labeled[PopupMenu[Dynamic[ptsJoin], {1 → "No", 2 → "Yes"}],
        "Join datapoints?", Top,
        LabelStyle → Directive[FontSize → 12, Bold, FontFamily → "Arial"]]]}],
    Row[{Panel[Labeled[InputField[Dynamic[pltSz], FieldSize → 5],
        "Enter plot size value.", Top,
        LabelStyle → Directive[FontSize → 12, FontFamily → "Arial"]]], "  ",
      Panel[Labeled[InputField[Dynamic[iconSizeMods], FieldSize → 5],
        "Enter plot icon size value.", Top,
        LabelStyle → Directive[FontSize → 12, FontFamily → "Arial"]]], "  ",
      Panel[Labeled[InputField[Dynamic[pltPad], FieldSize → 5],
        "Enter plot margin padding value.", Top,
        LabelStyle → Directive[FontSize → 12, FontFamily → "Arial"]]]}]},
   Center], "Model Coordinate Options", Top, LabelStyle →
   Directive[FontSize → 18, Bold, FontFamily → "Arial"]]]
pltSize = 500; iconSizeMods = 0.02; pltPad = 0.1;
xAxisName = pcNames⟦1⟧;
pltSz = 500; yAxisName = pcNames⟦2⟧; ptsJoin = 1;
dataTrans = 1;  pltAspect = 1 ;
lch = 2;
```

Out[∘]=

**Model Coordinate Options**

Enter type of model set.

◉ Along–axis Models ○ Subspace models

Select variable to be plotted on x–Axis.

Select variable to be plotted on y–Axis.

**Enter plot aspect ratio type.**

Golden Ratio Plot

**Show group domians?**

Simple scatterplot

**Join datapoints?**

No

Enter plot size value.

pltSz

Enter plot icon size value.

iconSiz⋱ eMod⋱ s

Enter plot margin padding value.

pltPad

## Plot model coordinates in space of a PCA plane (optional).

In[∘]:=

```
modpts1 = Table[0.0, {modelsPerAxis}, {2}];
modpts2 = Table[0.0, {modelsPerAxis}, {2}];
Do[If[xAxisName == pcNames⟦j⟧, axis1 = j], {j, noAxes}]
Do[If[yAxisName == pcNames⟦j⟧, axis2 = j], {j, noAxes}]

If[modSet == 1,
  If[meanTrans == 1,
   modpts1 = Take[modTable⟦axis1, All, {axis1, axis2}⟧];
   modpts2 = Take[modTable⟦axis2, All, {axis1, axis2}⟧];
   modpts = Partition[Flatten[modpts], 2]];
  If[meanTrans == 2,
   modpts1⟦All, 1⟧ = Take[modTable⟦axis1, All, axis1⟧];
   modpts2⟦All, 2⟧ = Take[modTable⟦axis2, All, axis2⟧]];
  modpts = Partition[Flatten[Append[modpts1, modpts2]], 2]];
modpts = Partition[modpts, modelsPerAxis];

If[modSet == 2, modpts = Partition[Flatten[modTable], 2]];
axisPlots = Table[" ", {2}];

groupNames = Union[Group];
numGroups = Length[groupNames];
groupPosns = Table[Flatten[Position[Group, groupNames⟦i⟧, 1]], {i, numGroups}];
```

```
eScoresT = Transpose[eScores];

xAxis = eScoresT⟦axis1⟧; yAxis = eScoresT⟦axis2⟧;
lab1 = StringJoin[{"Principal Component ", ToString[axis1]},
   {" (Var. =", ToString[t3⟦axis1 + 1, 3⟧]}, {"%)"}];
lab2 = StringJoin[{"Principal Component ", ToString[axis2]},
   {" (Var. =", ToString[t3⟦axis2 + 1, 3⟧]}, {"%)"}];
maxx =
 Max[
  xAxis];
minx = Min[xAxis];
maxy = Max[yAxis];
miny = Min[yAxis];

If[pltAspect == 1 || pltAspect == 3,
  xPlotLow = minx; xPlotHi = maxx; yPlotLow = miny; yPlotHi = maxy];
If[pltAspect == 2,
  If[minx > miny,
   xPlotLow = miny; yPlotLow = miny,
   xPlotLow = minx; yPlotLow = minx ]];
If[pltAspect == 2,
  If[maxx < maxy,
   xPlotHi = maxy; yPlotHi = maxy,
   xPlotHi = maxx; yPlotHi = maxx]];
If[pltAspect == 1, aRatio = 1 / N[GoldenRatio]];
If[pltAspect == 2, aRatio = 1];
If[pltAspect == 3, aRatio = Automatic];

tmpPoints = Transpose[List[xAxis, yAxis]];
pltPoints = Table[tmpPoints⟦groupPosns⟦j⟧⟧, {j, numGroups}];
iconList = Flatten[Table[
    {Graphics[{EdgeForm[{Thickness[0.005], Black}],
       Hue[N[((numGroups + 1) - j) / numGroups]],
       Disk[{0, 0}, Scaled[iconSizeMods]]}]}, {j, numGroups}]];

If[lch == 1 || ptsJoin == 1,
 Do[
  pltTable⟦k, 1⟧ = pTmp =
    ListPlot[pltPoints⟦k⟧, AspectRatio → aRatio, Frame → True, Joined → False,
     Axes → False, PlotRange → {{xPlotLow, xPlotHi}, {yPlotLow, yPlotHi}},
     PlotRangePadding → Scaled[pltPad], Ticks → Automatic, FrameLabel →
      {lab1, lab2}, PlotMarkers → iconList⟦k⟧, ImageSize → pltSize, LabelStyle →
      Directive[FontSize → 14, Black, FontFamily → "Arial"]], {k, kg}],
 Do[
  pltTable⟦k, 1⟧ =
   ListPlot[pltPoints⟦k⟧, Frame → True, Axes → False, AspectRatio → aRatio,
    PlotRange → {{xPlotLow, xPlotHi}, {yPlotLow, yPlotHi}}, PlotRangePadding →
```

```
    Scaled[pltPad], Ticks → Automatic, FrameLabel → {lab1, lab2},
   LabelStyle → Directive[Black, FontSize → 14, FontFamily → "Arial"],
   ImageSize → pltSize, PlotStyle →
    Directive[Disk[], Hue[N[((+1) - k) / kg]], EdgeForm[{Thickness[1.0], Black}],
     PointSize[Scaled[iconSize - 0.009]]]], {k, kg}]]

If[ptsJoin == 2,
  Do[
   pltTable⟦k, 2⟧ =
    ListLinePlot[pltPoints⟦k⟧, AspectRatio → aRatio, Frame → True, Joined → True,
     Axes → False, PlotStyle → Directive[Hue[N[((+1) - k) / kg]], Thin],
     PlotRange → {{xPlotLow, xPlotHi}, {yPlotLow, yPlotHi}},
     PlotRangePadding → Scaled[pltPad], Ticks → Automatic,
     FrameLabel → {lab1, lab2}, ImageSize → pltSize, LabelStyle →
      Directive[FontSize → 14, Black, FontFamily → "Arial"]], {k, kg}]];

If[lch == 2,
  Do[
   hull = ConvexHullMesh[pltPoints⟦k⟧];
   pltTable⟦k, 3⟧ = HighlightMesh[hull,
     Style[2, Opacity[0.2], Hue[N[((+1) - k) / kg]]], Frame → True, Axes → False,
     AspectRatio → aRatio, PlotRange → {{xPlotLow, xPlotHi}, {yPlotLow, yPlotHi}},
     PlotRangePadding → Scaled[pltPad],
     Ticks → Automatic, FrameLabel → {lab1, lab2},
     LabelStyle → Directive[Black, FontSize → 14, FontFamily → "Arial"],
     ImageSize → pltSize], {k, kg}]];

If[ptsJoin == 1 && lch == 1, p0 = Show[pltTable⟦All, 1⟧]];
If[ptsJoin == 2 && lch == 1, p0 = Show[pltTable⟦All, 2⟧, pltTable⟦All, 1⟧]];
If[ptsJoin == 1 && lch == 2, p0 = Show[pltTable⟦All, 3⟧, pltTable⟦All, 1⟧]];
If[ptsJoin == 2 && lch == 2,
  p0 = Show[pltTable⟦All, 3⟧, pltTable⟦All, 2⟧, pltTable⟦All, 1⟧]];

iconList = Flatten[Table[
    {Graphics[{EdgeForm[{Thin, Black}],
       Black, Disk[{0, 0}, Scaled[iconSizeMods / 2]]}]}, {j, 1}]];
If[modSet == 1,
  Do[
   axisPlots⟦i⟧ = ListPlot[modpts⟦i⟧, AspectRatio → aRatio,
     Frame → True, Joined → True, Axes → False, PlotRange → All,
     PlotStyle → Directive[Black, AbsoluteThickness[0.8]],
     PlotRangePadding → Scaled[pltPad], Ticks → Automatic,
     FrameLabel → {lab1, lab2}, PlotMarkers → iconList], {i, 2}];
  p4 = Show[axisPlots]];
If[modSet == 2,
  subSpacePlot = ListPlot[modpts, AspectRatio → aRatio,
    Frame → True, Joined → False, Axes → False, PlotRange → All,
```

```
      PlotStyle → Directive[Black, AbsoluteThickness[0.8]],
      PlotRangePadding → Scaled[pltPad], Ticks → Automatic,
      FrameLabel → {lab1, lab2}, PlotMarkers → iconList];
    p4 = subSpacePlot];
p1 = Labeled[Show[p0, p4], "          PC Score Plot", Top,
    LabelStyle → Directive[FontSize → 18, Bold, FontFamily → "Arial"]];
g1 = Grid[Table[
      {Graphics[{EdgeForm[{Thin, Black}], Hue[N[((numGroups + 1) - j) / numGroups]],
         Disk[]}, ImageSize → 13]}, {j, numGroups}], Frame → False];
g2 = Grid[Partition[groupNames, 1], Alignment → Left,
    BaseStyle → {FontFamily → "Arial", FontSize → 14
      , Italic}];
p2 = Labeled[Text[Grid[{{g1, g2}}, Alignment → Bottom, Frame → True]], "Legend",
    Top, LabelStyle → Directive[Black, FontSize → 18, Bold, FontFamily → "Arial"]];

plt2D = Grid[{{p1, p2}}, BaselinePosition → Top, Alignment → Top]
```

Export plot (optional).

```
In[•]:= filenameout = SystemDialogInput["FileSave"];
Export[filenameout, plt2D, "TIFF", ImageResolution → 150]
```

```
Out[•]= /Users/n.macleod/Desktop/Drangonflies
   (Final)/Data & Results/Images (PCA-CVA)/Hindwings/PCA
   Results/PC-1 vs PC-2 (Water Body Groups w: Model Coords).tif
```

Export PCA model coordinates (optional).

```
In[•]:= If[modSet == 1, partNum = modelAxes, partNum = 2];
If[modSet == 2, modTable = Reverse[modTable]];
modelsOut = Partition[Flatten[modTable], partNum];
filenameout = SystemDialogInput["FileSave"];
Export[filenameout, modelsOut, "CSV", "TextDelimiters" → ""]
```

```
Out[•]= /Users/n.macleod/Desktop/Butterflies 2/PCA Model Coords.csv
```

Data – Projection Module (optional)

This routine allows you to import data for objects that were not used to contruct the PCA space and project them into the PCA space with full support for data – visualization graphics

Read in projection datafile & partition into datasets.

In[]:=
```
filenamein = SystemDialogInput["FileOpen"];
x3 = Import[filenamein, "CSV"];
filenamein

{nProj, mProj} = Dimensions[x3];
varNames = Flatten[Take[x3, 1]];
x4 = Drop[x3, 1];
varNames = Drop[varNames, 1];
varNames = Drop[varNames, 1];

projObjNames = Flatten[Take[x4, nProj - 1, 1]];
x4 = Drop[x4, 0, 1];

projGroup = Flatten[Take[x4, nProj - 1, 1]];
x4 = Drop[x4, 0, 1];
numProjGroups = Length[Union[projGroup]];

{n4, m4} = Dimensions[x4];
Print["No. of objects: ", n4]
Print["No. of variables: ", m4]
```

Out[]=
```
/Users/n.macleod/Desktop/Data (5).csv

No. of objects: 20

No. of variables: 4
```

Project into PCA space and combine with original data.

In[]:=
```
If[meanTrans == 2, Do[x4[[i]] = x4[[i]] - mVec, {i, n4}]];
If[logTrans == 2, x2 = N[Log10[x4]]];
If[stdTrans == 2, x2 = Standardize[x4]];
If[shiftTrans == 2, x4 = x4 + knsnt];

projEScores = x4.eVecs;
combEScores = Join[Take[eScores, All, noAxes], projEScores];
combEScoresT = Transpose[combEScores];
combN = n2 + n4;

projGpNames = projGroup;
Do[projGpNames[[i]] = StringJoin["proj-", projGroup[[i]]], {i, n4}]
Do[projObjNames[[i]] = StringJoin[ToString[projObjNames[[i]]], "*"], {i, n4}]
combGroups = Join[Group, projGpNames];
combObjectNames = Join[objNames, projObjNames];
combGpNames = Union[combGroups];
numProjGroups = Length[combGpNames];
combProjPosns =
  Table[Flatten[Position[combGroups, combGpNames[[i]], 1]], {i, numProjGroups}];
```

# Projection Plot section

## Plot single – axis histogram.

### Specify single – variable plot (histogram) options.

In[∘]:=
```
pcNames = Table[StringJoin["PC-", ToString[i]], {i, noAxes}];
Panel[Labeled[Column[{
    Row[{Panel[Labeled[PopupMenu[Dynamic[axisName], pcNames],
        "Select component to be plotted.", Top,
        LabelStyle → Directive[FontSize → 12, Bold, FontFamily → "Arial"]]],
      "  ", Panel[Labeled[PopupMenu[Dynamic[histType],
         {1 → "Stacked", 2 → "Overlapped"}], "Select histogram type.", Top,
        LabelStyle → Directive[FontSize → 12, Bold, FontFamily → "Arial"]]]}],
    Row[{Panel[Labeled[InputField[Dynamic[noBins], FieldSize → 5],
        "Enter no. of histogram bins.", Top, LabelStyle →
         Directive[FontSize → 12, Bold, FontFamily → "Arial"]]], "  ",
      Panel[Labeled[InputField[Dynamic[hSize], FieldSize → 5],
        "Enter histogram plot size.", Top,
        LabelStyle → Directive[FontSize → 12, Bold, FontFamily → "Arial"]]]}]},
   Center], "Single Axis (Histogram) Plot Options", Top,
  LabelStyle → Directive[FontSize → 16, Bold, FontFamily → "Arial"]]]
axisName = pcNames⟦1⟧; noBins = 15; hSize = 500; histType = 1;
```

Out[∘]=

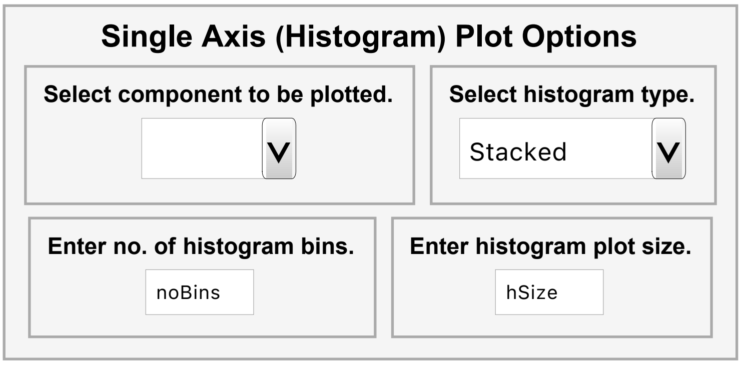


### Constrruct and display histogram plot .

In[•]:=
```
Do[If[axisName == pcNames[[j]], axis = j], {j, noAxes}];
Do[If[axisName == pcNames[[j]], axis = j], {j, noAxes}];
hScores = Flatten[Take[combEScores, All, {axis}]];
If[histType == 1, htype = "Stacked", htype = "Overlapped"];
projGpNames = Union[combGroups];
hPltPoints = Table[hScores[[combProjPosns[[j]]]], {j, numProjGroups}];
hueList =
  Table[Hue[N[((numProjGroups + 1) - j) / numProjGroups]], {j, numProjGroups}];
h2 = Labeled[Histogram[hPltPoints, noBins,
    ChartStyle → {hueList}, ChartLayout → htype,
    LabelStyle → Directive[FontSize → 12, Black, FontFamily → "Arial"], AxesLabel →
     {"PC Score", "Frequency"}, ImageSize → hSize, ChartLegends → projGpNames],
  StringJoin["Principal Component ", ToString[axis]], Top,
  LabelStyle → Directive[FontSize → 16, Bold, FontFamily → "Arial"]]
```

Export data projection histogram.

```
filenameout = SystemDialogInput["FileSave"];
Export[filenameout, projh1, "TIFF"]
```

```
/Users/nm/Projects/Storage/MacLeod/Manuscripts/01
  Developing/Wolf Dimorphism/Wolf Data/Mandibles/Data &
  Results 2/CVA Results/CV-1 Histogram (w: Unknowns).tif
```

Create 2 D scatterplot .

Specify 2 D plot options.
You must run this code after you read in the data so it can pick up the proper variable names.

```
In[•]:= pcNames = Table[StringJoin["PC-", ToString[i]], {i, noAxes}];
Panel[
 Labeled[Column[{Row[{Panel[Labeled[PopupMenu[Dynamic[xAxisName], pcNames],
       "Select variable to be plotted on x-Axis.", Top, LabelStyle →
        Directive[FontSize → 12, Bold, FontFamily → "Arial"]]], "  ",
     Panel[Labeled[PopupMenu[Dynamic[yAxisName], pcNames],
       "Select variable to be plotted on y-Axis.", Top,
       LabelStyle → Directive[FontSize → 12, Bold, FontFamily → "Arial"]]]}],
   Row[{
     Panel[Labeled[PopupMenu[Dynamic[pltAspect],
        {1 → "Golden Ratio Plot", 2 → "Square Plot (equi-length axes)",
         3 → "True-Scale Plot (actual axis scales)"}],
       "Enter plot aspect ratio type.", Top, LabelStyle →
        Directive[FontSize → 12, Bold, FontFamily → "Arial"]]], "  ",
     Panel[Labeled[PopupMenu[Dynamic[lch], {1 → "Simple scatterplot",
         2 → "Scatterplot w/ convex hulls"}],
       "Show group domians?", Top, LabelStyle →
        Directive[FontSize → 12, Bold, FontFamily → "Arial"]]], "  ",
     Panel[Labeled[PopupMenu[Dynamic[ptsJoin], {1 → "No", 2 → "Yes"}],
       "Join datapoints?", Top,
       LabelStyle → Directive[FontSize → 12, Bold, FontFamily → "Arial"]]]}],
   Row[{Panel[Labeled[InputField[Dynamic[pltSize], FieldSize → 10],
       "Enter plot size value.", Top,
       LabelStyle → Directive[FontSize → 12, Bold, FontFamily → "Arial"]]],
     "  ", Panel[Labeled[InputField[Dynamic[pltPad], FieldSize → 10],
       "Enter plot margin padding value.", Top, LabelStyle →
        Directive[FontSize → 12, Bold, FontFamily → "Arial"]]], "  ",
     Panel[Labeled[InputField[Dynamic[iconSize], FieldSize → 10],
       "Enter plot icon size value.", Top, LabelStyle →
        Directive[FontSize → 12, Bold, FontFamily → "Arial"]]]}]}, Center],
  "2D Plot Options", Top, LabelStyle → Directive[FontSize → 18,
    Bold, FontFamily → "Arial"]]]
pltSize = 500; iconSize = 0.025; pltPad = 0.1; xAxisName = pcNames⟦1⟧;
yAxisName = pcNames⟦2⟧;
 ptsJoin = 1; dataTrans = 1;  pltAspect = 1 ; lch = 2;
```

Out[∘]=

## 2D Plot Options

**Select variable to be plotted on x–Axis.**

**Select variable to be plotted on y–Axis.**

**Enter plot aspect ratio type.**

Golden Ratio Plot

**Show group domians?**

Simple scatterplot

**Join datapoints?**

No

**Enter plot size value.**

pltSize

**Enter plot margin padding value.**

pltPad

**Enter plot icon size value.**

iconSize

Plot script

In[∘]:=

```
projPltTable = Table[" ", {numProjGroups}, {noAxes}];
Do[If[xAxisName == pcNames⟦j⟧, axis1 = j], {j, noAxes}];
Do[If[yAxisName == pcNames⟦j⟧, axis2 = j], {j, noAxes}];

xAxis = combEScoresT⟦axis1⟧; yAxis = combEScoresT⟦axis2⟧;
lab1 = StringJoin[{"Principal Component ", ToString[axis1]},
   {" (Var. =", ToString[t3⟦axis1 + 1, 3⟧]}, {"%)"}];
lab2 = StringJoin[{"Principal Component ", ToString[axis2]},
   {" (Var. =", ToString[t3⟦axis2 + 1, 3⟧]}, {"%)"}];
maxx =
 Max[
  xAxis];
minx = Min[xAxis];
maxy = Max[yAxis];
miny = Min[yAxis];

If[pltAspect == 1 || pltAspect == 3,
  xPlotLow = minx; xPlotHi = maxx; yPlotLow = miny; yPlotHi = maxy];
If[pltAspect == 2,
  If[minx > miny,
   xPlotLow = miny; yPlotLow = miny,
   xPlotLow = minx; yPlotLow = minx ]];
If[pltAspect == 2,
  If[maxx < maxy,
   xPlotHi = maxy; yPlotHi = maxy,
   xPlotHi = maxx; yPlotHi = maxx]];
```

```
If[pltAspect == 1, aRatio = 1 / N[GoldenRatio]];
If[pltAspect == 2, aRatio = 1];
If[pltAspect == 3, aRatio = Automatic];

projTmpPoints = Transpose[List[xAxis, yAxis]];
projPltPoints = Table[projTmpPoints[[combProjPosns[[j]]]], {j, numProjGroups}];
iconList = Flatten[Table[
    {Graphics[
      {EdgeForm[{Thin, Black}], Hue[N[((numProjGroups + 1) - j) / numProjGroups]],
       Disk[{0, 0}, Scaled[iconSize]]}]}, {j, numProjGroups}]];

If[lch == 1 || ptsJoin == 1,
  Do[
   projPltTable[[k, 1]] =
    ListPlot[projPltPoints[[k]], AspectRatio → aRatio, Frame → True, Joined → False,
     Axes → False, PlotRange → {{xPlotLow, xPlotHi}, {yPlotLow, yPlotHi}},
     PlotRangePadding → Scaled[pltPad], Ticks → Automatic,
     FrameLabel → {lab1, lab2}, PlotMarkers → iconList[[k]],
     ImageSize → pltSize, LabelStyle → Directive[FontSize → 14,
       Black, FontFamily → "Arial"]], {k, numProjGroups}],
  Do[
   projPltTable[[k, 1]] =
    ListPlot[projPltPoints[[k]], Frame → True, Axes → False, AspectRatio → aRatio,
     PlotRange → {{xPlotLow, xPlotHi}, {yPlotLow, yPlotHi}}, PlotRangePadding →
      Scaled[pltPad], Ticks → Automatic, FrameLabel → {lab1, lab2},
     LabelStyle → Directive[Black, FontSize → 14, FontFamily → "Arial"],
     ImageSize → pltSize, PlotStyle → Directive[Disk[], Hue[
        N[((numGroups + 1) - k) / numGroups]], EdgeForm[{Thickness[1.0], Black}],
       PointSize[Scaled[iconSize - 0.009]]]], {k, numProjGroups}]];

If[ptsJoin == 2,
  Do[
   projPltTable[[k, 2]] = ListLinePlot[projPltPoints[[k]],
     AspectRatio → aRatio, Frame → True, Joined → True, Axes → False, PlotStyle →
      Directive[Hue[N[((numProjGroups + 1) - k) / numProjGroups]], Thin],
     PlotRange → {{xPlotLow, xPlotHi}, {yPlotLow, yPlotHi}},
     PlotRangePadding → Scaled[pltPad], Ticks → Automatic,
     FrameLabel → {lab1, lab2}, ImageSize → pltSize, LabelStyle → Directive[
       FontSize → 14, Black, FontFamily → "Arial"]], {k, numProjGroups}]];

If[lch == 2,
  Do[
   hull = ConvexHullMesh[projPltPoints[[k]]];
   projPltTable[[k, 3]] = HighlightMesh[hull,
     Style[2, Opacity[0.2], Hue[N[((numProjGroups + 1) - k) / numProjGroups]]],
     Frame → True, Axes → False, AspectRatio → aRatio,
     PlotRange → {{xPlotLow, xPlotHi}, {yPlotLow, yPlotHi}}, PlotRangePadding →
```

```
      Scaled[pltPad], Ticks → Automatic, FrameLabel → {lab1, lab2},
     LabelStyle → Directive[Black, FontSize → 14, FontFamily → "Arial"],
     ImageSize → pltSize], {k, numProjGroups}]];

If[ptsJoin == 1 && lch == 1, p0 = Show[projPltTable[[All, 1]]]];
If[ptsJoin == 2 && lch == 1,
  p0 = Show[projPltTable[[All, 2]], projPltTable[[All, 1]]]];
If[ptsJoin == 1 && lch == 2, p0 = Show[projPltTable[[All, 3]], projPltTable[[All, 1]]]];
If[ptsJoin == 2 && lch == 2,
  p0 = Show[projPltTable[[All, 3]], projPltTable[[All, 2]], projPltTable[[All, 1]]]];

p1 = Labeled[p0, "           PC Score Plot", Top,
   LabelStyle → Directive[FontSize → 18, Bold, FontFamily → "Arial"]];
g1 = Grid[Table[
    {Graphics[{EdgeForm[{Thin, Black}],
       Hue[N[((numProjGroups + 1) - j) / numProjGroups]], Disk[]},
      ImageSize → 13]}, {j, numProjGroups}], Frame → False];
g2 = Grid[Partition[combGpNames, 1], Alignment → Left,
   BaseStyle → {FontFamily → "Arial", FontSize → 14
     , Italic}];
p2 = Labeled[
    Text[Grid[{{g1, g2}}, Alignment → Bottom, Frame → True]], "Legend", Top,
    LabelStyle → Directive[Black, FontSize → 18, Bold, FontFamily → "Arial"]];;

jPlt2D = Grid[{{p1, p2}}, BaselinePosition → Top, Alignment → Top]
```

Export current 2 D plot.

```
In[∘]:= filenameout = SystemDialogInput["FileSave"];
        Export[filenameout, projPlt2D, "TIFF", ImageResolution → 150]

Out[∘]= /Users/n.macleod/Desktop/Archaeological
          Types/Ford's Houses/Figure 1/Images (Houses + Piers)/Circle
          Groups/PCA Results/PC-3 vs PC-2 (w: figs 2 & 3 Means).tif
```

Label plotted points.

```
In[•]:= pn1 = p0;
projNamePoints = projTmpPoints;
projTempPointsT = Transpose[projTmpPoints];
mxY = Max[projTempPointsT[[2]]];
mnY = Min[projTempPointsT[[2]]];
incY = N[(mxY - mnY) / 15];
Do[projNamePoints[[i, 2]] = projTmpPoints[[i, 2]] - incY, {i, combN}]
nPointsTable =
  Table[{Text[combObjectNames[[i]], projNamePoints[[i]], {-1, 0}]}, {i, combN}];
pn2 = Graphics[nPointsTable, Frame → True, AspectRatio → aRatio , Axes → False,
   FrameLabel  → {lab1, lab2}, PlotRangePadding → Scaled[pltPad], BaseStyle →
    Directive[FontSize → 12, FontFamily → "Arial"], ImageSize → pltSize];
p1 = Labeled[Show[pn1, pn2, BaseStyle → {FontFamily → "Arial"}],
   "           PC Score Plot", Top,
   LabelStyle → Directive[FontSize → 18, Bold, FontFamily → "Arial"]];

Plt2D = Grid[{{p1, p2}}, BaselinePosition → Top, Alignment → Top]
Print["Projected point names marked with '*'."]
```

```
Projected point names marked with '*'.
```

Export current 2 D plot.

```
filenameout = SystemDialogInput["FileSave"];
Export[filenameout, Plt2D, "TIFF", ImageResolution → 150]
```

Create  3 D scatterplot .

Specify 3 D plot options.
You must run this code after you read in the data so it can pick up the proper variable names.

In[•]:=
```
pcNames = Table[StringJoin["PC-", ToString[i]], {i, noAxes}];
Panel[
 Labeled[Column[{Row[{Panel[Labeled[PopupMenu[Dynamic[xAxisName], pcNames],
       "Select variable to be plotted on x-Axis.", Top, LabelStyle →
        Directive[FontSize → 12, Bold, FontFamily → "Arial"]]], "  ",
      Panel[Labeled[PopupMenu[Dynamic[yAxisName], pcNames],
        "Select variable to be plotted on y-Axis.", Top, LabelStyle →
         Directive[FontSize → 12, Bold, FontFamily → "Arial"]]], "  ",
      Panel[Labeled[PopupMenu[Dynamic[zAxisName], pcNames],
        "Select variable to be plotted on z-Axis.", Top,
        LabelStyle → Directive[FontSize → 12, Bold, FontFamily → "Arial"]]]}],
    Row[{
      Panel[Labeled[PopupMenu[Dynamic[pltAspect],
          {1 → "Golden Ratio Plot", 2 → "Square Plot (equi-length axes)",
           3 → "True-Scale Plot (actual axis scales)"}],
        "Enter plot aspect ratio type.", Top, LabelStyle →
         Directive[FontSize → 12, Bold, FontFamily → "Arial"]]], "  ",
      Panel[Labeled[PopupMenu[Dynamic[lch], {1 → "Simple scatterplot",
           2 → "Scatterplot w/ convex hulls"}],
        "Show group domians?", Top, LabelStyle →
         Directive[FontSize → 12, Bold, FontFamily → "Arial"]]], " ",
      Panel[Labeled[PopupMenu[Dynamic[ptsJoin], {1 → "No", 2 → "Yes"}],
        "Join datapoints?", Top,
        LabelStyle → Directive[FontSize → 12, Bold, FontFamily → "Arial"]]]}],
    Row[{Panel[Labeled[InputField[Dynamic[pltSize], FieldSize → 5],
        "Enter plot size value.", Top,
        LabelStyle → Directive[FontSize → 12, Bold, FontFamily → "Arial"]]],
      " ", Panel[Labeled[InputField[Dynamic[pltPad], FieldSize → 5],
        "Enter plot margin padding value.", Top, LabelStyle →
         Directive[FontSize → 12, Bold, FontFamily → "Arial"]]], " ",
      Panel[Labeled[InputField[Dynamic[iconSize3D], FieldSize → 5],
        "Enter plot icon size value.", Top, LabelStyle →
         Directive[FontSize → 12, Bold, FontFamily → "Arial"]]]}]}, Center],
  "3D Plot Options", Top, LabelStyle → Directive[FontSize → 18,
    Bold, FontFamily → "Arial"]]]
pltSize = 500; iconSize3D = 50; pltPad = 0.1; xAxisName = pcNames⟦1⟧;
 yAxisName = pcNames⟦2⟧;
zAxisName = pcNames⟦3⟧; ptsJoin = 1;
dataTrans = 1;
 pltAspect = 3 ; lch = 2;
```

Out[•]=

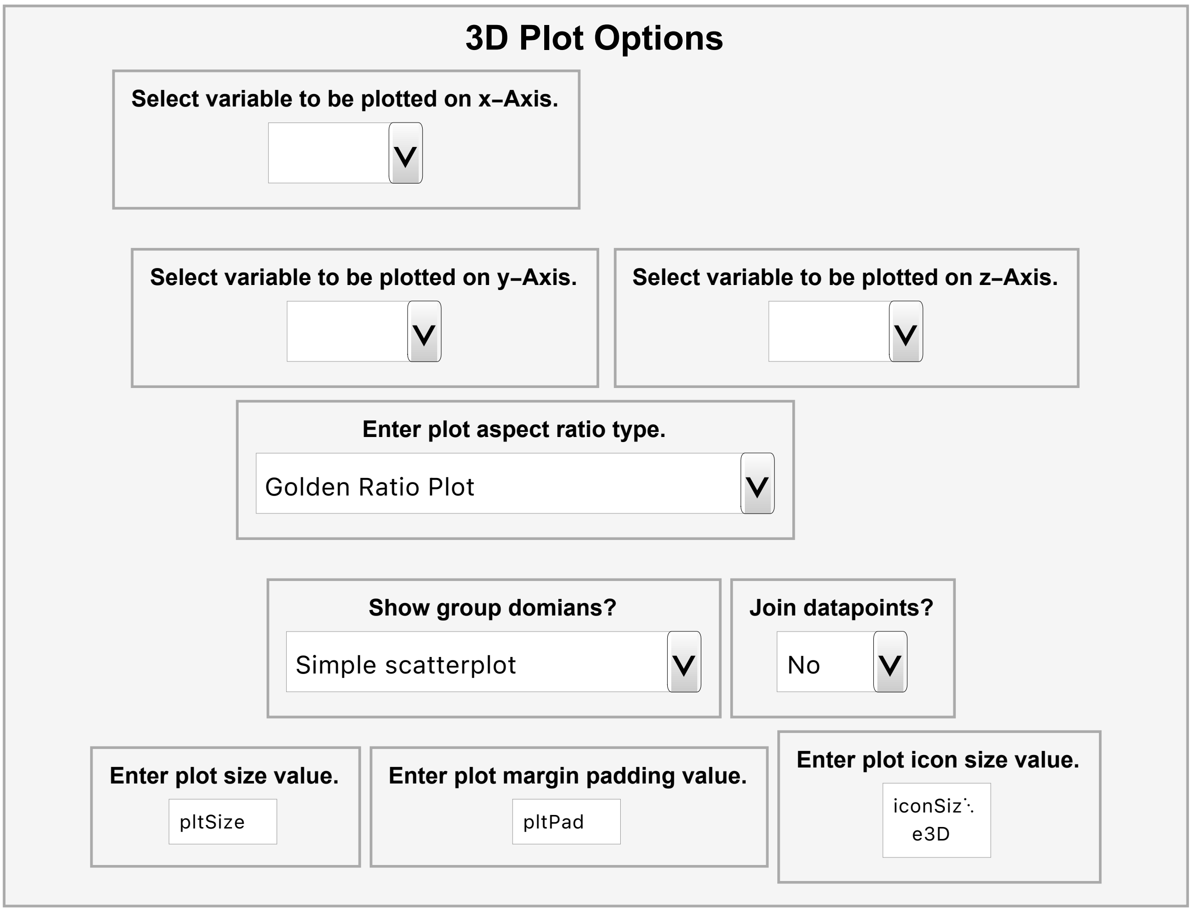


## Plot script

In[•]:=

```
Do[If[xAxisName == pcNames[[j]], axis1 = j], {j, noAxes}]
Do[If[yAxisName == pcNames[[j]], axis2 = j], {j, noAxes}]
Do[If[zAxisName == pcNames[[j]], axis3 = j], {j, noAxes}]

xAxis = combEScoresT[[axis1]];
yAxis = combEScoresT[[axis2]];
zAxis = combEScoresT[[axis3]];
lab1 = StringJoin["CV-", ToString[axis1]];
lab2 = StringJoin["CV-", ToString[axis2]];
lab3 = StringJoin["CV-", ToString[axis3]];
maxx = Max[xAxis];
minx = Min[xAxis];
maxy = Max[yAxis];
miny = Min[yAxis];
maxz = Max[zAxis]; minz = Min[zAxis];

mxax = Max[xAxis];
mnax = Min[xAxis];
If[pltAspect < 3, f = N[iconSize3D * 0.020]];
If[pltAspect == 3, f = N[iconSize3D * 0.0005]];
iSz3D = (mxax - mnax) * f;
```

```
If[pltAspect == 1 || pltAspect == 3,
  xPlotLow = minx; xPlotHi = maxx; yPlotLow = miny; yPlotHi = maxy];
If[pltAspect == 2,
  If[minx > miny,
   xPlotLow = miny; yPlotLow = miny,
   xPlotLow = minx; yPlotLow = minx ]];
If[pltAspect == 2,
  If[maxx < maxy,
   xPlotHi = maxy; yPlotHi = maxy,
   xPlotHi = maxx; yPlotHi = maxx]];

If[pltAspect == 1, bRatio = {1.61803, 1, 1}];
If[pltAspect == 2, bRatio = {1, 1, 1}];
If[pltAspect == 3, bRatio = Automatic];

projPoints3 = Transpose[List[xAxis, yAxis, zAxis]];
comGp1 = combProjPosns;
combProjH = Table[0, {combN}];
Do[
  tmp = comGp1[[i]];
  itr = Length[tmp];
  Do[combProjH[[tmp[[j]]]] = Hue[N[((numProjGroups + 1) - i) / numProjGroups]],
   {j, itr}], {i, numProjGroups}];

If[pltAspect == 3,
  pltPoints = Table[{combProjH[[i]], Sphere[projPoints3[[i]], iSz3D]}, {i, combN}];
  p0 = Graphics3D[pltPoints, Axes → True,
    Boxed → True, PlotRangePadding → Scaled[pltPad],
    LabelStyle → Directive[FontSize → 12, Black, FontFamily → "Arial"],
    AxesLabel → {lab1, lab2, lab3}, ImageSize → pltSize, BoxRatios → Automatic]];
If[pltAspect ≠ 3,
  pltPoints = Table[{combProjH[[i]],
     AbsolutePointSize[iSz3D], Point[projPoints3[[i]]]}, {i, combN}];
  p0 = Graphics3D[pltPoints, Axes → True, Boxed → True,
    PlotRangePadding → Scaled[pltPad],
    LabelStyle → Directive[FontSize → 12, Black, FontFamily → "Arial"],
    AxesLabel → {lab1, lab2, lab3}, ImageSize → pltSize, BoxRatios → bRatio]];
If[ptsJoin == 2,
  pltLineTable = Table[" ", {numProjGroups}];
  gpPoints = Table[projPoints3[[combProjPosns[[j]]]], {j, numProjGroups}];
  Do[
   pltLineTable[[k]] = Graphics3D[
     {Hue[N[((numProjGroups + 1) - k) / numProjGroups]], Line[gpPoints[[k]]]},
     Axes → True, Boxed → True, PlotRangePadding → Scaled[pltPad],
     LabelStyle → Directive[FontSize → 12, Black, FontFamily → "Arial"],
     AxesLabel → {lab1, lab2, lab3}, ImageSize → pltSize,
```

```
      BoxRatios → bRatio, ViewPoint → {xax, yax, zax}], {k, numProjGroups}];
   p0 = Show[{pltLineTable, p0}]];
 If[lch == 2,
   pltMeshTable = Table[" ", {numProjGroups}];
   gpPoints = Table[projPoints3[[combProjPosns[[j]]]], {j, numProjGroups}];
   Do[
    {n3, m3} = Dimensions[gpPoints[[k]]];
    cHull3D = ConvexHullMesh[gpPoints[[k]], BaseStyle → {EdgeForm[]},
      Boxed → True, Axes → True, PlotRangePadding → Scaled[pltPad],
      LabelStyle → Directive[FontSize → 12, Black, FontFamily → "Arial"],
      AxesLabel → {lab1, lab2, lab3}, ImageSize → pltSize,
      BoxRatios → bRatio, ViewPoint → {xax, yax, zax}];
    pltMeshTable[[k]] = Show[{HighlightMesh[cHull3D,
        Style[2, Opacity[0.2], Hue[N[((numProjGroups + 1) - k) / numProjGroups]]]],
       p0}], {k, numProjGroups}];
   p0 = Show[pltMeshTable]];

 p1 = Labeled[p0, "           PC Score Plot", Top,
    LabelStyle → Directive[FontSize → 18, Bold, FontFamily → "Arial"]];
 h2 = Table[0, {numProjGroups}];
 l1 = Table[" ", {numProjGroups}, {2}];
 horiz = Table["Left", numProjGroups];
 vert = Table["Center", numProjGroups];
 Do[h2[[i]] = Hue[N[((numProjGroups + 1) - i) / numProjGroups]], {i, numProjGroups}];
 If[pltAspect == 3,
   i1 = Table[{h2[[i]], Sphere[{0, 0, 0}, 0.01]}, {i, numProjGroups}];
   Do[l1[[i, 1]] = Graphics3D[i1[[i]], Boxed → False, ImageSize → 16],
    {i, numProjGroups}]];
 If[pltAspect ≠ 3,
  i1 = Table[{h2[[i]], EdgeForm[{Directive[{Thickness[0.05]}]}],
     Disk[{0, 0}, 0.001]}, {i, numProjGroups}];
  Do[l1[[i, 1]] = Graphics[i1[[i]], ImageSize → 12], {i, numProjGroups}]]
 Do[l1[[i, 2]] = Style[combGpNames[[i]], FontFamily → "Arial",
     FontSlant → Italic, FontSize → 14], {i, numProjGroups}];
 g2 = Labeled[Grid[l1, Frame → True, Alignment → {horiz, vert},
     Spacings → {1, 0.5}], "Legend", Top,
    LabelStyle → Directive[Black, FontSize → 16, Bold, FontFamily → "Arial"]];

 plt3D = Grid[{{p1, g2}}, BaselinePosition → Top, Alignment → Top]
```

In[∘]:= iSz3D

Out[∘]= 0.370176

Adjust orientation of 3 D plot (if necessary).

You must replot the data to activate the changes. These changes will be able to be exported using the script below.

In[∘]:=
```
Panel[
 Labeled[Row[{Labeled[Slider[Dynamic[xax], {-10, 10}, Appearance → "Labeled"],
      "x-Axis Viewpoint", Top,
      LabelStyle → Directive[FontSize → 10, Bold, FontFamily → "Arial"]] ×
     Labeled[Slider[Dynamic[yax], {-10, 10}, Appearance → "Labeled"],
      "y-Axis Viewpoint", Top,
      LabelStyle → Directive[FontSize → 10, Bold, FontFamily → "Arial"]] ×
     Labeled[Slider[Dynamic[zax], {-10, 10}, Appearance → "Labeled"],
      "z-Axis Viewpoint", Top,
      LabelStyle → Directive[FontSize → 10, Bold, FontFamily → "Arial"]]}],
  "3D Plot Orientation Controls", Top, LabelStyle →
   Directive[FontSize → 14, Bold, FontFamily → "Ariel"]]]
xax = 2.5; yax = -2.5; zax = 2.5;
```

Out[∘]=

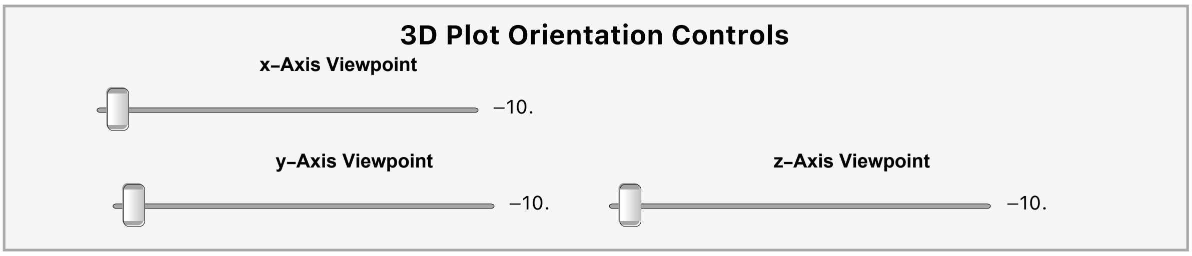


Export current 3 D plot.

In[∘]:=
```
filenameout = SystemDialogInput["FileSave"];
Export[filenameout, plt3D, "TIFF", ImageResolution → 150]
```

Out[∘]=
```
/Users/n.macleod/Desktop/Archaeological Types/Ford's
  Houses/Figure 1/Images (Houses + Piers)/Circle Groups/PCA
  Results/PC-1 vs PC-2 vs PC-3 (w: figs 2 & 3 Means).tif
```

Export projected PCA scores.

```
Proj = Length[Union[projGroup]];
adata = Table[" ", {n4 + 1}, {noAxes + 2}];
adata[[1, 1]] = "Object";
adata[[1, 2]] = "Group";
Do[adata[[i + 1, 1]] = projObjectNames[[i]], {i, n4}]
Do[adata[[i + 1, 2]] = projGroup[[i]], {i, n4}]
Do[
 adata[[1, j + 2]] = StringJoin["PC-", ToString[j]], {j, 1, noAxes}]
Do[adata[[i + 1, j + 2]] = projEScores[[i, j]], {i, n4}, {j, noAxes}]

filenameout = SystemDialogInput["FileSave"];
Export[filenameout, adata, "CSV", "TextDelimiters" → ""]
```
