# Supplementary material for: What you sample is what you get: ecomorphological variation in Trithemis (Odonata, Libellulidae) dragonfly wings reconsidered
Source: BMC Ecol Evol. 2022 Apr 11;22:43. doi: 10.1186/s12862-022-01978-y (PMC8996507; doi:10.1186/s12862-022-01978-y)
Supplement: Supplementary file 3 — Additional file 3: Software Archive. [file 12862_2022_1978_MOESM3_ESM.zip › Additional Files 3/Software Archive/Image Processing (vers. 3.0).pdf]

Image Processing

This program reads in a series of .tif images and processes them in preparation for eigenimage analysis.

Author : N. MacLeod

Version : 3.0

Date : 7 May 2018

Reference : MacLeod (2015)

Initialize libraries.

```
In[•]:= << ComputationalGeometry`
```

Read in data file & partition into datasets.
[Note : If the image files are large this can take some time.]

In[∘]:=
```
filenamein = SystemDialogInput["FileOpen"];
sourceDirectory = DirectoryName[filenamein]
SetDirectory[sourceDirectory];
dataFileList = Sort[Map[ToString, FileNames["*.tif"]]];
{kg} = Dimensions[dataFileList];
groupNames = Table[" ", {kg}];

x1 = Table[" ", {kg}];
Do[xName = StringJoin[sourceDirectory, dataFileList⟦k⟧];
 x1⟦k⟧ = Import[xName, "TIFF"], {k, kg}]
objNames = dataFileList;
Do[objNames⟦k⟧ = StringDrop[objNames⟦k⟧, -4], {k, kg}]
Do[groupNames⟦k⟧ = "Group 1", {k, kg}];
x2 = x1;
{iWidth, iHeight} = ImageDimensions[x1⟦1⟧];
Do[
 {tstWidth, tstHeight} = ImageDimensions[x1⟦k⟧];
 If[tstWidth < iWidth, iWidth = tstWidth];
 If[tstHeight < iHeight, iHeight = tstHeight], {k, 2, kg}]
Panel[
 Labeled[
  Column[{Panel[Labeled[InputField[Dynamic[kg], FieldSize → 5], "No. of Images", Top,
      LabelStyle → Directive[FontSize → 10, FontFamily → "Arial"]]],
    Row[{Panel[Labeled[InputField[Dynamic[iWidth], FieldSize → 5], "Common Width",
         Top, LabelStyle → Directive[FontSize → 10, FontFamily → "Arial"]]], " ",
       Panel[Labeled[InputField[Dynamic[iHeight], FieldSize → 5], "Common Height",
         Top, LabelStyle → Directive[FontSize → 10, FontFamily → "Arial"]]]}]},
   Center], "Original Images Summary", Top, LabelStyle →
   Directive[FontSize → 12, Bold, FontFamily → "Arial"]]]
```

Out[∘]= /Users/n.macleod/Desktop/Drangonflies (Final)/Data & Results/Images (PCA-CVA)/Hindwings/Images (Reduced + Phylogeny)/

Out[∘]=

Original Images Summary

No. of Images
kg

Common Width	Common Height
iWidth	iHeight

Display original images (optional).

Select user – specified plot options.

In[•]:=
```
Panel[Labeled[Column[{Row[
      {Panel[Labeled[RadioButtonBar[Dynamic[sampOpt], {1 → "All", 2 → "Sample"},
          LabelStyle → (FontFamily → "Arial")], "Select image display option",
         Top, LabelStyle → Directive[FontSize → 12, FontFamily → "Arial"]]], " ",
       Panel[Labeled[InputField[Dynamic[kgImg], FieldSize → 5],
         "Enter no. of images to display.", Top,
         LabelStyle → Directive[FontSize → 12, Plain, FontFamily → "Arial"]]]}],
    Row[{Panel[Labeled[InputField[Dynamic[rowNum], FieldSize → 5],
         "Enter number of plots per grid row.", Top, LabelStyle →
          Directive[FontSize → 12, Plain, FontFamily → "Arial"]]], " ",
       Panel[Labeled[InputField[Dynamic[imgSize], FieldSize → 5],
         "Enter image size control parameter.", Top,
         LabelStyle → Directive[FontSize → 12, Plain, FontFamily → "Arial"]]]}],
    Panel[Labeled[InputField[Dynamic[grdSize], FieldSize → 5],
       "Enter grid size control parameter.", Top,
       LabelStyle → Directive[FontSize → 12, Plain, FontFamily → "Arial"]]]},
   Center], "Processed Image Display Options", Top,
  LabelStyle → Directive[FontSize → 16, Bold, FontFamily → "Arial"]]]
sampOpt = 1; kgImg = 50; rowNum = 4; imgSize = 100; grdSize = 700;
```

Out[•]=
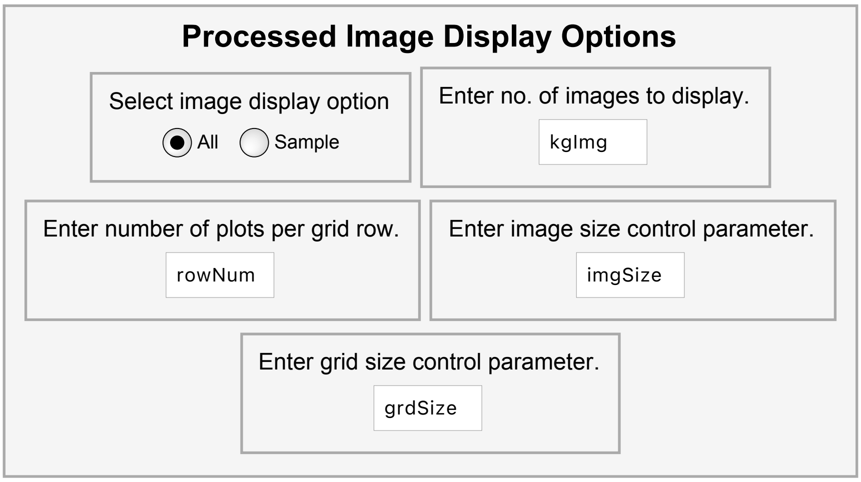

Display original images.

In[•]:=
```
If[sampOpt == 1,
  kgDisp = kg;
  gridTitle = "Original Images (All)";
  imgDisp = Table[i, {i, kg}],
  kgDisp = kgImg;
  gridTitle = "Original Images (Random Sample)";
  imgDisp = Sort[RandomSample[Range[kg], kgDisp]]];

t1 = N[kgDisp / rowNum];
t2 = IntegerPart[N[kgDisp / rowNum]];
If[t1 - t2 > 0., kg2 = (t2 + 1) * rowNum, kg2 = t2 * rowNum];
imgTable = Table[" ", {kg2}];
Do[imgTable〚k〛 = Labeled[ImageResize[x1〚imgDisp〚k〛〛, imgSize],
   objNames〚imgDisp〚k〛〛, Top, LabelStyle →
    Directive[FontSize → 12, Italic, FontFamily → "Arial"]], {k, kgDisp}]
imgPlt1 = Labeled[GraphicsGrid[Partition[imgTable, rowNum],
   Frame → All, ImageSize → grdSize], gridTitle, Top,
  LabelStyle → Directive[FontSize → 18, Bold, FontFamily → "Arial"]]
```

Export original image table (optional).

In[•]:=
```
filenameout = SystemDialogInput["FileSave"];
Export[filenameout, imgPlt1, "TIFF"]
```

Out[•]=
```
/Users/n.macleod/Desktop/Fish Table.tif
```

Image processing options (please run this code to pick up image names).

In[•]:=
```
Panel[Labeled[
  Column[{Row[{Panel[Labeled[Row[{Panel[Labeled[RadioButtonBar[Dynamic[imgRot],
           {1 → "No", 2 → "Yes"}, LabelStyle → (FontFamily → "Arial")],
          "Rotate Images", Top, LabelStyle →
           Directive[FontSize → 12, FontFamily → "Arial"]]], " ",
        Panel[Labeled[InputField[Dynamic[imgRotVal], FieldSize → 5],
          "Enter rotation value (in degrees).", Top, LabelStyle →
           Directive[FontSize → 12, Plain, FontFamily → "Arial"]]]}],
      "Rotation Values", Top, LabelStyle → Directive[FontSize → 14,
        Bold, FontFamily → "Arial"]]], "  ",
     Panel[Labeled[Row[{Panel[Labeled[PopupMenu[Dynamic[imgColor],
           {1 → "RGB", 2 → "Greyscale"}], "Specify input image color.", Top,
          LabelStyle → Directive[FontSize → 12, FontFamily → "Arial"]]], "  ",
        Panel[Labeled[PopupMenu[Dynamic[bColor], {1 → "Black", 2 → "White"}],
          "Specify background color.", Top, LabelStyle → Directive[
            FontSize → 12, FontFamily → "Arial"]]]}], "Color Values", Top,
      LabelStyle → Directive[FontSize → 14, Bold, FontFamily → "Arial"]]]}],
    Row[{Panel[Labeled[Row[{Panel[Labeled[RadioButtonBar[Dynamic[imgFSizeOpt],
           {1 → "No", 2 → "Yes"}, LabelStyle → (FontFamily → "Arial")],
```

```
            "Standardize Image Framesizes", Top,
            LabelStyle → Directive[FontSize → 12, FontFamily → "Arial"]]]}],
        "Frame Size Standardization", Top, LabelStyle →
         Directive[FontSize → 14, Bold, FontFamily → "Arial"]]], "  ",
      Panel[Labeled[Row[{Panel[Labeled[RadioButtonBar[Dynamic[imgFSRedOpt],
             {1 → "No", 2 → "Yes"}, LabelStyle → (FontFamily → "Arial")],
            "Framesize Reduction Option", Top, LabelStyle →
             Directive[FontSize → 12, FontFamily → "Arial"]]], " ",
          Panel[Labeled[InputField[Dynamic[fmSize], FieldSize → 5],
            "Enter length (in pixels) of image x-axis.", Top, LabelStyle →
             Directive[FontSize → 12, Plain, FontFamily → "Arial"]]]}],
        "Frame Size Adjustments", Top, LabelStyle → Directive[
          FontSize → 14, Bold, FontFamily → "Arial"]]]}],
    Row[{Panel[Labeled[Row[{Panel[Labeled[RadioButtonBar[Dynamic[imgSizOpt],
             {1 → "No", 2 → "Yes"}, LabelStyle → (FontFamily → "Arial")],
            "Standardize Image Sizes", Top, LabelStyle →
             Directive[FontSize → 12, FontFamily → "Arial"]]]}],
        "Image Size Standardization", Top, LabelStyle →
         Directive[FontSize → 14, Bold, FontFamily → "Arial"]]], "  ",
      Panel[Labeled[Row[{Panel[Labeled[RadioButtonBar[Dynamic[gScaleOpt],
             {1 → "No", 2 → "Yes", 3 → "Convert & Standardize Shade"},
             LabelStyle → (FontFamily → "Arial")], "Convert to Grayscale", Top,
            LabelStyle → Directive[FontSize → 12, FontFamily → "Arial"]]],
          " ", Panel[Labeled[InputField[Dynamic[gsShade], FieldSize → 5],
            "Greyscale Shade Standardization Value", Top, LabelStyle →
             Directive[FontSize → 12, Plain, FontFamily → "Arial"]]]}],
        "Greyscale Conversions", Top, LabelStyle → Directive[
          FontSize → 14, Bold, FontFamily → "Arial"]]]}],
    Panel[Labeled[Row[{Panel[Labeled[RadioButtonBar[Dynamic[imgAlignOpt],
           {1 → "None", 2 → "Align Images to a Reference",
            3 → "Align Images' Major Axes"},
           LabelStyle → (FontFamily → "Arial")], "Image Alignments", Top,
          LabelStyle → Directive[FontSize → 12, FontFamily → "Arial"]]],
        " ", Panel[Labeled[PopupMenu[Dynamic[refName], objNames],
          "Select image to be used as the alignment reference.", Top,
          LabelStyle → Directive[FontSize → 12, FontFamily → "Arial"]]]}],
      "Image Alignment Options", Top, LabelStyle → Directive[
        FontSize → 14, Bold, FontFamily → "Arial"]]]},
   Center], "Image Processing Options", Top,
  LabelStyle →
   Directive[
    FontSize → 18,
    Bold,
    FontFamily → "Arial"]]]
imgRot = 1; imgRotVal = 90; imgColor = 1; bColor = 1; imgFSizeOpt = 1;
imgFSRedOpt = 1;
fmSize = 100;
```

```
imgSizOpt = 1;
gScaleOpt = 1;
gsShade = 0.7;
imgAlignOpt = 1; refName = objNames⟦1⟧;
```

Out[∘]=

Image Processing Options

Rotation Values

Rotate Images

(●) No ( ) Yes

Enter rotation value (in degrees).

imgRot⋱Val

Color Values

Specify input image color.

RGB

Specify background color.

Black

Frame Size Standardization

Standardize Image Framesizes

(●) No ( ) Yes

Frame Size Adjustments

Framesize Reduction Option

(●) No ( ) Yes

Enter length (in pixels) of image x–axis.

fmSize

Image Size Standardization

Standardize Image Sizes

(●) No ( ) Yes

Greyscale Conversions

Convert to Grayscale

(●) No ( ) Yes ( ) Convert & Standardize Shade

Greyscale Shade Standardization Value

gsShade

Image Alignment Options

Image Alignments

(●) None ( ) Align Images to a Reference ( ) Align Images' Major Axes

Select image to be used as the alignment reference.

001

Process images.

```
In[◦]:= x2 = x1;
Panel[Labeled[ProgressIndicator[Dynamic[k / kg]], Dynamic[progTitle], Top,
   LabelStyle → Directive[FontSize → 12, Bold, FontFamily → "Arial"]]]

If[imgRot == 2,
   progTitle = "Image Rotation Calculations";
   Do[x2⟦k⟧ = ImageRotate[x2⟦k⟧, imgRotVal], {k, kg}]];

If[imgFSizeOpt == 2,
   progTitle = "Frame Size Standardization Calculations";
   x2 = x1;
   If[bColor == 1, bkgnd = Black, bkgnd = White];
   imgSizes = Table[" ", {kg}];
   imgAR = Table[" ", {kg}];
   Do[x2⟦k⟧ = ImageCrop[x2⟦k⟧], {k, kg}];
   Do[imgSizes⟦k⟧ = ImageDimensions[x2⟦k⟧], {k, kg}];

   maxWidth = Max[Take[imgSizes⟦All, 1⟧]];
   maxHeight = Max[Take[imgSizes⟦All, 2⟧]];
   frameWidth = maxWidth + Round[maxWidth * 0.10];
   frameHeight = maxHeight + Round[maxHeight * 0.10];

   Do[
    {imgWidth, imgHeight} = ImageDimensions[x2⟦k⟧];
    difWidth = frameWidth - imgWidth;
    difHeight = frameHeight - imgHeight;
    If[OddQ[difHeight] == True, rit = 1, rit = 0];
    pad = IntegerPart[difHeight / 2];
    padTop = pad;
    padBot = pad + rit;
    If[OddQ[difWidth] == True, rit = 1, rit = 0];
    pad = IntegerPart[difWidth / 2];
    padLeft = pad;
    padRight = pad + rit;
    x2⟦k⟧ =
     ImagePad[x2⟦k⟧, {{padLeft, padRight}, {padTop, padBot}}, bkgnd], {k, kg}];
   {imgWidth, imgHeight} = ImageDimensions[x2⟦1⟧]];

If[imgFSRedOpt == 2,
```

```
    progTitle = "Frame Size Reduction Calculations";
    Do[x2⟦k⟧ = ImageResize[x2⟦k⟧, fmSize], {k, kg}];
    {fmSizeX, fmSizeY} = ImageDimensions[x2⟦1⟧]];

  If[imgSizOpt == 2,
    progTitle = "Image Size Calculations";
    imgArea = Table[0, {kg}];
    {frameLength, frameHeight} = ImageDimensions[x2⟦1⟧];
    If[imgColor == 1, If[bColor == 1, bgnd = {0.0, 0.0, 0.0}, bgnd = {1.0, 1.0, 1.0}],
     If[bColor == 1, bgnd = 0.0, bgnd = 1.0]];
    Do[
     k1 = k;
     x3 = ImageData[x2⟦k⟧];
     Do[If[EuclideanDistance[bgnd, x3⟦i, j⟧] ≤ 0.05, x3⟦i, j⟧ = bgnd,
       imgArea⟦k⟧ = imgArea⟦k⟧ + 1], {i, frameHeight}, {j, frameLength}], {k, kg}];
    minImgArea = Min[imgArea];

    Do[
     progTitle = "Image Size Difference Calculations";
     knt = 0;
     Do[
      k2 = k;
      knt = knt + 1;
      imgAreaTest = 0;
      imgLength = (frameLength - iter);
      lgnProp = imgLength / frameLength;
      imgHeight = Round[frameHeight * lgnProp];
      x3 = ImageData[ImageResize[x2⟦k⟧, {imgLength, imgHeight}]];
      Do[If[EuclideanDistance[bgnd, x3⟦i, j⟧] > 0.05,
        imgAreaTest = imgAreaTest + 1], {i, imgHeight}, {j, imgLength}];
      If[imgAreaTest ≤ minImgArea, Break[]], {iter, frameLength}];
     imgLength = (frameLength - (knt - 1));
     lgnProp = N[imgLength / frameLength];
     imgHeight = Round[frameHeight * lgnProp];
     x2⟦k⟧ = ImageResize[x2⟦k⟧, {imgLength, imgHeight}], {k, kg}];

    Do[x2⟦k⟧ = ImageCrop[x2⟦k⟧], {k, kg}];
    Do[imgSizes⟦k⟧ = ImageDimensions[x2⟦k⟧], {k, kg}];
    maxWidth = Max[Take[imgSizes⟦All, 1⟧]];
    minWidth = Min[Take[imgSizes⟦All, 1⟧]];
    maxHeight = Max[Take[imgSizes⟦All, 2⟧]];
    minHeight = Min[Take[imgSizes⟦All, 2⟧]];

    maxWidth = Max[Take[imgSizes⟦All, 1⟧]];
    maxHeight = Max[Take[imgSizes⟦All, 2⟧]];
    frameWidth = maxWidth + Round[maxWidth * 0.20];
    frameHeight = maxHeight + Round[maxHeight * 0.20];
```

```
  Do[
   progTitle = "Image Size Standardization Calculations";
   {imgWidth, imgHeight} = ImageDimensions[x2[[k]]];
   difWidth = frameWidth - imgWidth;
   difHeight = frameHeight - imgHeight;
   If[OddQ[difHeight] == True, rit = 1, rit = 0];
   pad = IntegerPart[difHeight / 2];
   padTop = pad;
   padBot = pad + rit;
   If[OddQ[difWidth] == True, rit = 1, rit = 0];
   pad = IntegerPart[difWidth / 2];
   padLeft = pad;
   padRight = pad + rit;
   x2[[k]] =
    ImagePad[x2[[k]], {{padLeft, padRight}, {padTop, padBot}}, bkgnd], {k, kg}];
  {imgWidth, imgHeight} = ImageDimensions[x2[[1]]]];

If[gScaleOpt ≥ 2,
  Do[x2[[k]] = ColorConvert[x2[[k]], "Grayscale"], {k, kg}]];

If[imgAlignOpt == 2,
  progTitle = "Image Alignment Calculations";
  Do[If[objNames[[k]] == refName, refNum = k], {k, kg}];
  ref = x2[[refNum]];
  inc = 1;
  {x, y} = ImageDimensions[ref];
  If[x > y, buf = x, buf = y];
  pad = IntegerPart[N[buf / 3]];
  padRef = ImagePad[ref, pad, White];
  binRef = Binarize[ref, 0.9];
  datRef = Flatten[ImageData[binRef]];
  rotTab = Table[0.0, {360 / inc}];
  dtor = N[Pi / 180];

  k = 1;
  Do[
   If[k ≠ refNum,
    padTest = ImagePad[x2[[k]], pad, White];
    knt = 0;
    Do[
     knt = knt + 1;
     rotTest = ImageRotate[padTest, i * dtor, Background → bkgnd, Masking → All];
     cropTest = ImageCrop[rotTest, {x, y}];
     binTest = Binarize[cropTest, 0.9];
     datTest = Flatten[ImageData[binTest]];
     rotTab[[knt]] = N[Correlation[datRef, datTest]], {i, 1, 360, inc}];
```

```
     maxCor = Max[rotTab];
     Do[If[rotTab⟦i⟧ == maxCor, ang = i], {i, 360}];
     rotTest =
      ImageRotate[padTest, ang * dtor, Background → bkgnd, Masking → All];
     x2⟦k⟧ = ImageCrop[rotTest, {x, y}]], {k, kg}]];

 If[imgAlignOpt == 3,
   progTitle = "Image Alignment Calculations";
   imgSizes = Table[" ", {kg}];
   Do[
    k2 = k;
    x3 = ImageData[x2⟦k⟧];
    {m2, n2} = Dimensions[x3];
    n3 = n2 * m2;
    sil = Table[0.0, {n3}, {2}];
    knt = 0;
    Do[
     If[x3⟦i, j⟧ < 1.0,
      knt = knt + 1;
      sil⟦knt, 2⟧ = i;
      sil⟦knt, 1⟧ = j], {j, n2}, {i, m2}];
    sil = Take[sil, knt, All];
    mSil = Mean[sil];
    Do[sil⟦i⟧ = sil⟦i⟧ - mSil, {i, knt}];
    Do[sil⟦i, 2⟧ = sil⟦i, 2⟧ * -1.0, {i, knt}];

    coVar = Covariance[sil];
    eVecs = Chop[Eigenvectors[coVar]];
    If[eVecs⟦1, 2⟧ > 0.01,
     x2⟦k⟧ = ImageRotate[x2⟦k⟧, eVecs⟦1, 2⟧, Background → bkgnd]], {k, kg}];

   Do[
    x2⟦k⟧ = ImageCrop[x2⟦k⟧];
    imgSizes⟦k⟧ = ImageDimensions[x1⟦k⟧], {k, kg}];

   maxWidth = Max[Take[imgSizes⟦All, 1⟧]];
   maxHeight = Max[Take[imgSizes⟦All, 2⟧]];
   frameWidth = maxWidth;
   frameHeight = maxHeight;

   Do[
    {imgWidth, imgHeight} = ImageDimensions[x2⟦k⟧];
    difWidth = frameWidth - imgWidth;
    difHeight = frameHeight - imgHeight;
    If[OddQ[difHeight] == True, rit = 1, rit = 0];
    pad = IntegerPart[difHeight / 2];
    padTop = pad;
```

```
    padBot = pad + rit;
    If[OddQ[difWidth] == True, rit = 1, rit = 0];
    pad = IntegerPart[difWidth / 2];
    padLeft = pad;
    padRight = pad + rit;
    x2[[k]] =
     ImagePad[x2[[k]], {{padLeft, padRight}, {padTop, padBot}}, bkgnd], {k, kg}]];

{imgWidth, imgHeight} = ImageDimensions[x2[[1]]];
Panel[
 Labeled[Panel[Row[{Labeled[InputField[Dynamic[imgWidth], FieldSize → 5], "Width", Top,
       LabelStyle → Directive[FontSize → 12, FontFamily → "Arial"]], " ",
      Labeled[InputField[Dynamic[imgHeight], FieldSize → 5], "Height", Top,
       LabelStyle → Directive[FontSize → 12, FontFamily → "Arial"]]}]],
   "Current Image Size", Top, LabelStyle → Directive[
     FontSize → 14, FontFamily → "Arial"]]]
```

Out[▫]=

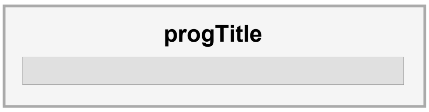

Out[▫]=

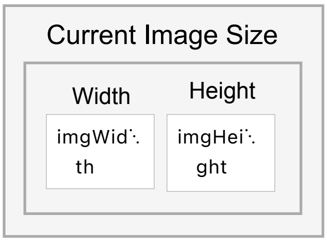

Display processed images.

In[∘]:=
```
If[sampOpt == 1,
  kgDisp = kg;
  gridTitle = "Processed Images (All)";
  imgDisp = Table[i, {i, kg}],
  kgDisp = kgImg;
  gridTitle = "Processed Images (Random Sample)";
  imgDisp = Sort[RandomSample[Range[kg], kgDisp]]];

t1 = N[kgDisp / rowNum];
t2 = IntegerPart[N[kgDisp / rowNum]];
If[t1 - t2 > 0., kg2 = (t2 + 1) * rowNum, kg2 = t2 * rowNum];
imgTable = Table[" ", {kg2}];
Do[imgTable[[k]] = Labeled[ImageResize[x2[[imgDisp[[k]]]], imgSize],
   objNames[[imgDisp[[k]]]], Top, LabelStyle →
    Directive[FontSize → 12, Italic, FontFamily → "Arial"]], {k, kgDisp}]
imgPlt1 = Labeled[GraphicsGrid[Partition[imgTable, rowNum],
   Frame → All, ImageSize → grdSize], gridTitle, Top,
  LabelStyle → Directive[FontSize → 18, Bold, FontFamily → "Arial"]]
```

Accept processing results (optional)

In[∘]:=
```
x1 = x2;
```

In[∘]:=
```
x2 = x1;
```

Export processed image table (optional).

In[∘]:=
```
filenameout = SystemDialogInput["FileSave"];
Export[filenameout, imgPlt1, "TIFF"]
```

Out[∘]= /Users/n.macleod/Desktop/AUS-Peru/Combined (Reduced)/sdcs

Finalize processed images' frame size.

Square up frame aspect ratio.

Obtain desired framesize dimension (optional).

In[∘]:=
```
Panel[Labeled[InputField[Dynamic[fmSize], FieldSize → 10],
  "Enter length (in pixels) of image x-axis.", Top,
  LabelStyle → Directive[FontSize → 12, Bold, FontFamily → "Arial"]]]
```

Out[∘]=

Enter length (in pixels) of image x-axis.

fmSize

Reduce image sizes (optional).

In[∘]:=
```
x2 = x1;
Do[x2⟦k⟧ = ImageResize[x2⟦k⟧, fmSize], {k, kg}]
{fmSizeX, fmSizeY} = ImageDimensions[x2⟦1⟧];

Panel[
 Labeled[Panel[Row[{Labeled[InputField[Dynamic[fmSizeX], FieldSize → 5], "Width", Top,
      LabelStyle → Directive[FontSize → 12, FontFamily → "Arial"]], " ",
     Labeled[InputField[Dynamic[fmSizeY], FieldSize → 5], "Height", Top, LabelStyle →
       Directive[FontSize → 12, FontFamily → "Arial"]]}]], "Current Image Size",
  Top, LabelStyle → Directive[FontSize → 14, Bold, FontFamily → "Arial"]]]
```

Out[∘]=

Current Image Size

Width | Height
fmSizeX | fmSizeY

Accept processing results (optional)

In[∘]:=
```
x1 = x2;
```

Export processed images (optional).

In[∘]:=
```
filenameout = SystemDialogInput["FileSave"];
sourceDirectory = DirectoryName[filenameout];
SetDirectory[sourceDirectory];
Do[filenameout = StringJoin[objNames⟦k⟧, ".tif"];
 Export[filenameout, x1⟦k⟧, "TIFF"], {k, kg}]
```

Assemble and write – out data and image files for eigenimage analysis

Specify colorspace of output images.

In[∘]:=
```
Panel[Labeled[PopupMenu[Dynamic[cSpace], {1 → "Color (RGB)", 2 → "Greyscale"}],
  "Specify colorspace of images.", Top,
  LabelStyle → Directive[FontSize → 12, Bold, FontFamily → "Arial"]]]
cSpace = 1;
```

Out[∘]=

Specify colorspace of images.

Color (RGB) ∨

Assign the images to groups.

Select user – specified plot options.

You don't have to run this section, just make whatever changes, if any, you'd like.

In[∘]:=
```
Panel[
 Labeled[PopupMenu[Dynamic[gpOption], {1 → "Assign all images to 'Group 1'",
    2 → "Use external file to make group assignments"}],
  "Specify input image color.", Top, LabelStyle →
   Directive[FontSize → 12, Bold, FontFamily → "Arial"]]]
gpOption = 1;
```

Out[∘]=

Specify input image color.

Assign all images to 'Group 1'

Generate or read – in group file.

In[∘]:=
```
If[gpOption == 1, groupNames = Table["Group 1", {kg}];,
 filenamein = SystemDialogInput["FileOpen"];
 sourceDirectory = DirectoryName[filenamein];
 groupNames = Flatten[Import[filenamein, "CSV"]];
 filenamein]
```

Out[∘]=
```
/Users/n.macleod/Desktop/Drangonflies (Final)/Data
  & Results/Images (PCA-CVA)/Hindwings/Water Body Groups.csv
```

Assemble and write out processed data.

```
In[•]:= {n1, m1} = ImageDimensions[x1⟦1⟧];
If[cSpace == 1, mtot = (n1 * m1) * 3, mtot = n1 * m1];
outData = Table["0", {kg + 1}, {mtot + 2}];
outData⟦1, 1⟧ = "Object";
outData⟦1, 2⟧ = "Group";

If[cSpace == 1,
  tot = 0;
  Do[
   Do[
    Do[
     If[k == 1, channel = "r"];
     If[k == 2, channel = "g"];
     If[k == 3, channel = "b"];
     tot = tot + 1;
     outData⟦1, tot + 2⟧ =
      StringJoin[{StringJoin[{StringJoin[{channel, ToString[i]}], "-"}],
        ToString[j]}], {k, 3}], {i, n1}], {j, m1}];

  Do[
   outData⟦k + 1, 1⟧ = objNames⟦k⟧;
   outData⟦k + 1, 2⟧ = groupNames⟦k⟧;
   flatImage = Flatten[ImageData[x1⟦k⟧]];
   Do[outData⟦k + 1, j + 2⟧ = flatImage⟦j⟧, {j, mtot}], {k, kg}]];

If[cSpace == 2,
  tot = 0;
  Do[
   Do[
    tot = tot + 1;
    outData⟦1, tot + 2⟧ =
     StringJoin[{StringJoin[{StringJoin[{"g", ToString[j]}], "-"}],
       ToString[i]}], {i, n1}], {j, m1}];
  Do[outData⟦i + 1, 1⟧ = objNames⟦i⟧, {i, kg}];
  Do[outData⟦i + 1, 2⟧ = groupNames⟦i⟧, {i, kg}];
  If[cSpace == 2,
   Do[
    imgMat = ImageData[x1⟦k⟧];
    kount = 0;
    Do[
     Do[
      kount = kount + 1;
      outData⟦k + 1, kount + 2⟧ = imgMat⟦j, i⟧, {i, n1}], {j, m1}], {k, kg}]]];

filenameout = SystemDialogInput["FileSave"];
Export[filenameout, outData, "CSV", "TextDelimiters" → ""]
```

Out[∘]= /Users/n.macleod/Desktop/Drangonflies (Final)/Data & Results/Images (PCA-CVA)/Hindwings/Images (Water Body Groups).csv

Calculate and display mean image.

```
In[∘]:= matX1 = Drop[outData, 1];
mImageMat = Take[matX1, All, {3, mtot + 2}];
meanImageMat = Mean[mImageMat];

If[cSpace == 1, mImage = Labeled[Image[Partition[Partition[meanImageMat, 3], n1],
     ColorSpace → "RGB", ImageSize → 400], "Mean Image", Top,
   LabelStyle → Directive[FontSize → 14, FontFamily → "Arial"]],
 mImage = Labeled[Image[Partition[meanImageMat, n1],
     ColorSpace → "Grayscale", ImageSize → 400], "Mean Image", Top,
   LabelStyle → Directive[FontSize → 14, FontFamily → "Arial"]]]
```

Write – out mean image (optional).

```
In[∘]:= filenameout = SystemDialogInput["FileSave"];
Export[filenameout, mImage, "TIFF", ImageResolution → 150]
```

Out[∘]= /Users/n.macleod/Desktop/Drangonflies (Final)/Data & Results/Images (PCA-CVA)/Hindwings/Hindwings Mean Image.tif

Write – out mean image data.

```
In[∘]:= filenameout = SystemDialogInput["FileSave"];
Export[filenameout, meanImageMat, "CSV", "TextDelimiters" → ""]
```

Out[∘]= /Users/n.macleod/Desktop/Drangonflies (Final)/Data & Results/Images (PCA-CVA)/Hindwings/Hindwings Mean Image.csv
