# Supplementary material for: What you sample is what you get: ecomorphological variation in Trithemis (Odonata, Libellulidae) dragonfly wings reconsidered
Source: BMC Ecol Evol. 2022 Apr 11;22:43. doi: 10.1186/s12862-022-01978-y (PMC8996507; doi:10.1186/s12862-022-01978-y)
Supplement: Supplementary file 3 — Additional file 3: Software Archive. [file 12862_2022_1978_MOESM3_ESM.zip › Additional Files 3/Software Archive/CVA (1.50).pdf]

Canonical Variates Analysis

This program accepts a data matrix in standard format,
performs a CVA on the data columns, and allows the result to be viewed
as 2 D or 3 D (interactive) scatterplots. Full support is provided for tracking groups
that have been identified in the datafile.

Author : N. MacLeod
Version : 1.41
Date : 24 August 2021
Reference : MacLeod (2007)

Initialize libraries

In[∘]:=
```
<< ComputationalGeometry`
```

Read in data file & partition into datasets.

In[∘]:=
```
filenamein = SystemDialogInput["FileOpen"];
x1 = Import[filenamein, "CSV"];
filenamein

{n1, m1} = Dimensions[x1];

varNames = Flatten[Take[x1, 1]];
x2 = Drop[x1, 1];
varNames = Drop[varNames, 1];
varNames = Drop[varNames, 1];

objNames = Flatten[Take[x2, n1 - 1, 1]];
x2 = Drop[x2, 0, 1];

Group = Flatten[Take[x2, n1 - 1, 1]];
x2 = Drop[x2, 0, 1];
numGroups = Length[Union[Group]];

{n2, m2} = Dimensions[x2];

Print["No. of Objects: ", n2];
Print["No. of Variables: ", m2];
Print["No. of Groups: ", Length[Union[Group]]];
```

Out[∘]=
```
/Users/n.macleod/Desktop/Data (20).csv
```

```
No. of Objects: 80

No. of Variables: 4

No. of Groups: 4
```

Data transformations (optional)

Specify global data transformations (if any).
Note : Remember you cannot subsequently take the logarithm of mean – centered or standardized data. If you wish to perform such an analysis you must shift the mean – centered or standardized data by a constant (e.g., 1, 10)

In[∘]:=
```
Panel[Labeled[Column[
  {Row[{Panel[Labeled[PopupMenu[Dynamic[meanTrans], {1 → "No", 2 → "Yes"}],
       "Mean center data?", Top, LabelStyle →
        Directive[FontSize → 12, Bold, FontFamily → "Arial"]]], "  ",
     Panel[Labeled[PopupMenu[Dynamic[logTrans], {1 → "No", 2 → "Yes"}],
       "Log10-trasform data?", Top, LabelStyle →
        Directive[FontSize → 12, Bold, FontFamily → "Arial"]]], "  ",
     Panel[Labeled[PopupMenu[Dynamic[stdTrans], {1 → "No", 2 → "Yes"}],
       "Standardize data?", Top,
       LabelStyle → Directive[FontSize → 12, Bold, FontFamily → "Arial"]]]}],
   Row[{Panel[Labeled[PopupMenu[Dynamic[shiftTrans], {1 → "No", 2 → "Yes"}],
       "Shift data by a constant?", Top, LabelStyle →
        Directive[FontSize → 12, Bold, FontFamily → "Arial"]]], "  ",
     Panel[Labeled[InputField[Dynamic[knsnt], FieldSize → 5],
       "Enter shift constant value.", Top,
       LabelStyle → Directive[FontSize → 12, Bold, FontFamily → "Arial"]]]}]},
  Center], "Global Data Transform Options", Top, LabelStyle →
  Directive[FontSize → 18, Bold, FontFamily → "Arial"]]]
meanTrans = 1; logTrans = 1; stdTrans = 1; shiftTrans = 1; knsnt = 10;
```

Out[∘]=

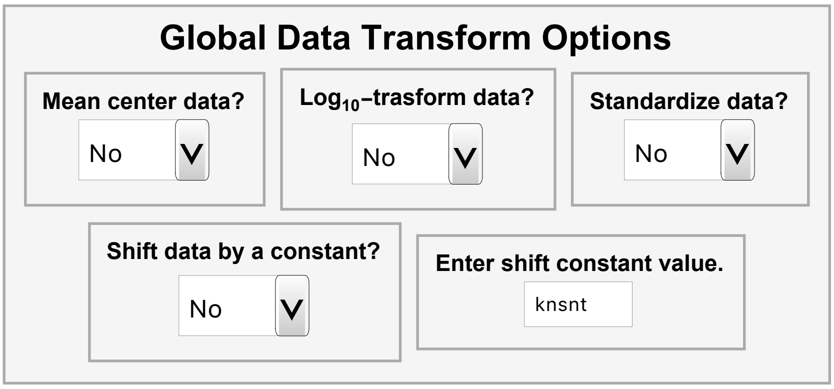

Perform global data transformation (optional)

In[∘]:=
```
If[meanTrans == 2, mVec = N[Mean[x2]];
  Do[x2⟦i⟧ = x2⟦i⟧ - mVec, {i, n2}]];
If[logTrans == 2, x2 = N[Log10[x2]]];
If[stdTrans == 2, x2 = Standardize[x2]];
If[shiftTrans == 2, x2 = x2 * knsnt];
```

Export mean vector (optional; may be useful in modelling).

```
filenameout = SystemDialogInput["FileSave"];
Export[filenameout, mVec, "csv"]
```

Export processed dataset (optional; may be useful in other analyses).

```
x2Trans = Table[" ", {n2 + 1}, {m2 + 2}];
x2Trans⟦1, 1⟧ = "Object";
x2Trans⟦1, 2⟧ = "Group";
Do[x2Trans⟦1, j + 2⟧ = varNames⟦j⟧, {j, m2}]
Do[x2Trans⟦i + 1, 1⟧ = objNames⟦i⟧, {i, n2}]
Do[x2Trans⟦i + 1, 2⟧ = Group⟦i⟧, {i, n2}]
Do[x2Trans⟦i + 1, j + 2⟧ = x2⟦i, j⟧, {i, n2}, {j, m2}]

filenameout = SystemDialogInput["FileSave"];
Export[filenameout, x2Trans, "CSV", "TextDelimiters" → ""]
```

Choose eigenanalysis method

In[∘]:=
```
Panel[Labeled[PopupMenu[Dynamic[eMethod],
    {1 → "Std. Eigenanalysis", 2 → "Singular Value Decomposition"}],
  "Choose eigenanalysis method", Top,
  LabelStyle → Directive[FontSize → 12, Bold, FontFamily → "Arial"]]]
```

Out[∘]=

Choose eigenanalysis method

Std. Eigenanalysis ∨

Perform eigenanalysis

In[∘]:=
```
pltTable = Table[" ", {numGroups}, {3}];
{gndMean, T, gNames, nGps, gpMeans, smpSize, W, B} = TWB[x2, n2, m2, Group];
kg = Length[Union[Group]];
WI = Inverse[W];
sCovar = WI.B;
If[n2 > m2, mvecs = m2, mvecs = n2];
If[n2 > m2, mvecs = m2, mvecs = n2];
```

```
If[eMethod == 1,
  eVals = Eigenvalues[sCovar];
  w = Eigenvectors[sCovar];
  eVecs = Transpose[w];
  eScores = x2.eVecs;
  eVecsTotal = eVecs];
If[eMethod == 2,
  {w, u, v} = SingularValueDecomposition[sCovar, mvecs];
  eVals = N[Diagonal[u], kg - 1];
  eVecs = Take[Transpose[w], mvecs];
  eScores = x2.w;
  eVecsTotal = eVecs];
eS2 = eScores;

noVals = Min[{nGps - 1, m2}];
noAxes = noVals;
eVals = Take[eVals, noVals];
s1 = Total[eVals];
evalTable = Table[0.0, {i, kg - 1}, {j, 4}];
Do[evalTable[[i, 1]] = i, {i, 1, kg - 1}];
Do[evalTable[[i, 2]] = eVals[[i]], {i, 1, kg - 1}];
Do[evalTable[[i, 3]] = (eVals[[i]] 100.`)/s1, {i, 1, kg - 1}];
Do[evalTable[[i, 4]] = evalTable[[i, 3]], {i, 1, kg - 1}];
Do[evalTable[[i, 4]] = evalTable[[i - 1, 4]] + evalTable[[i, 3]], {i, 2, kg - 1}];

tabHeads =
  {"Component", " Eigenvalue", "  Variance (%)", "  Cum. Variance (%)"};
t2 = Partition[Flatten[Join[tabHeads, evalTable]], 4];
t3 = Partition[Flatten[t2], 4];

Do[
 Do[
  t3[[i, j]] = PaddedForm[t3[[i, j]], {5, 3}], {i, 2, kg - 1 + 1}], {j, 2, 4}]

Labeled[Grid[t3, BaseStyle → (FontFamily → "Arial"),
  Alignment → {{Center, Right, Right, Right}},
  Frame → True, Dividers → {{True, True}, {True, True}}],
 "Eigenvalue Table", Top, LabelStyle → (FontFamily → "Arial")]
```

Display CVA Results (optional).

Display CV eigenvector loadings (optional).

In[•]:=
```
evecTable = Table[0.0, {i, m2 + 1}, {j, noAxes + 1}];
Do[evecTable[[i + 1, 1]] = varNames[[i]], {i, m2}]
evecTable[[1, 1]] = "Variable";
Do[evecTable[[1, i + 1]] = StringJoin["CV-", ToString[i]], {i, noAxes}]
Do[Do[evecTable[[i + 1, j + 1]] = PaddedForm[eVecs[[i, j]], {5, 3}], {i, m2}],
 {j, noAxes}]
Labeled[Grid[evecTable, BaseStyle → (FontFamily → "Arial"),
  Alignment → {{Center, Right, Right, Right, Right, Right}}, Frame → True,
  Dividers → {{True, True}, {True, True}}], "Eigenvector Table", Top,
 LabelStyle → Directive[Black, Bold, FontSize → 14, FontFamily → "Arial"]]
```

Display CV scores (optional).

In[•]:=
```
scrTable = Table[0.0, {i, n2 + 1}, {j, noAxes + 2}];
Do[scrTable[[i + 1, 1]] = objNames[[i]], {i, n2}]
scrTable[[1, 1]] = "Objects";
scrTable[[1, 2]] = "Groups";
Do[scrTable[[1, i + 2]] = StringJoin["CV-", ToString[i]], {i, noAxes}]
Do[scrTable[[i + 1, 2]] = Group[[i]], {i, n2}]
Do[scrTable[[i + 1, j + 2]] = PaddedForm[eScores[[i, j]], {5, 3}], {i, n2}, {j, noAxes}]
Labeled[Grid[scrTable, BaseStyle → (FontFamily → "Arial"),
  Alignment → {{Left, Left, Right, Right, Right, Right}}, Frame → True,
  Dividers → {{True, True, True}, {True, True}}], "Eigenscore Table", Top,
 LabelStyle → Directive[Black, Bold, FontSize → 14, FontFamily → "Arial"]]
```

Plot single – axis histogram.

Specify single – variable plot (histogram) options.
You must run this code after you read in the data so it can pick up the proper variable names.

In[∘]:=
```
cvNames = Table[StringJoin["CV-", ToString[i]], {i, noAxes}];
Panel[Labeled[Column[{
    Row[{Panel[Labeled[PopupMenu[Dynamic[axisName], cvNames],
        "Select variable to be plotted on x-Axis.", Top,
        LabelStyle → Directive[FontSize → 12, Bold, FontFamily → "Arial"]]],
      "  ", Panel[Labeled[PopupMenu[Dynamic[histType],
         {1 → "Stacked", 2 → "Overlapped"}], "Select histogram type.", Top,
        LabelStyle → Directive[FontSize → 12, Bold, FontFamily → "Arial"]]]}],
    Row[{Panel[Labeled[InputField[Dynamic[noBins], FieldSize → 5],
        "Enter no. of histogram bins.", Top, LabelStyle →
         Directive[FontSize → 12, Bold, FontFamily → "Arial"]]], "  ",
      Panel[Labeled[InputField[Dynamic[hSize], FieldSize → 5],
        "Enter histogram plot size.", Top,
        LabelStyle → Directive[FontSize → 12, Bold, FontFamily → "Arial"]]]}]},
   Center], "Single Axis (Histogram) Plot Options", Top,
  LabelStyle → Directive[FontSize → 16, Bold, FontFamily → "Arial"]]]
axisName = cvNames⟦1⟧; noBins = 15; hSize = 500; histType = 2;
```

Out[∘]=

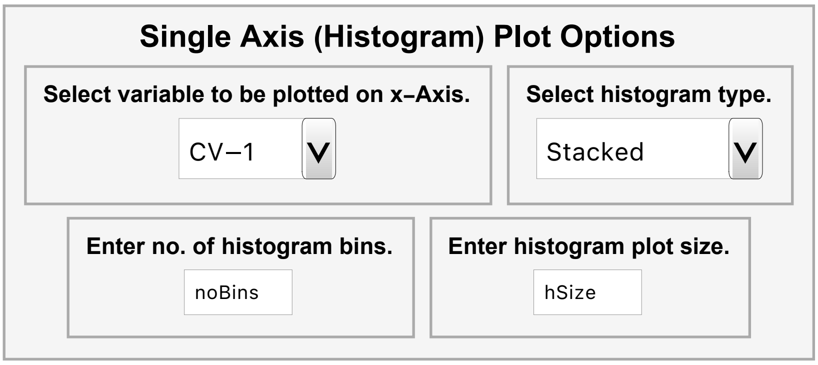

Construct and display histogram plot .

In[∘]:=
```
Do[If[axisName == cvNames⟦j⟧, axis = j], {j, noAxes}];
pltScores = Flatten[Take[eScores, All, {axis}]];
If[histType == 1, htype = "Stacked", htype = "Overlapped"];
gpNames = Union[Group];
groupPosns = Table[Flatten[Position[Group, gpNames⟦i⟧, 1]], {i, numGroups}];
pltPoints = Table[pltScores⟦groupPosns⟦j⟧⟧, {j, numGroups}];
hueList = Table[Hue[N[((numGroups + 1) - j) / numGroups]], {j, numGroups}];
h1 =
 Labeled[Histogram[pltPoints, noBins, ChartStyle → {hueList}, ChartLayout → htype,
   LabelStyle → Directive[FontSize → 12, Black, FontFamily → "Arial"],
   AxesLabel → {StringJoin["CV-", ToString[axis]], "Frequency"},
   ImageSize → hSize, ChartLegends → gpNames],
  StringJoin["Canonical Variate ", ToString[axis]], Top,
  LabelStyle → Directive[FontSize → 16, Bold, FontFamily → "Arial"]]
```

Export current histogram.

```
In[•]:= filenameout = SystemDialogInput["FileSave"];
Export[filenameout, h1, "TIFF", ImageResolution → 150]
Out[•]= /Users/n.macleod/Desktop/Bumpus Sparrow Results/CV-1.tif
```

Estimate statistical significance of group centroid separations.

Test Two – Group Separation w/ Hotelling's T^2 statistic.

Specify testing options

Standard (F – ratio) Distribution =

use this test if your data are normally distributed and the group covariance matrices are equal

Monte Carlo Simulated Distribution =

use this test if your data are not normally distributed and/or the group covariance matrices are not equal

Bootstrap Simulated Distribution =

use this test if your data are not normally distributed and/or the group covariance matrices are not equal

In[∘]:=
```
Panel[
 Labeled[Column[{Row[{Panel[Labeled[InputField[Dynamic[hSize2], FieldSize → 10],
       "Enter histogram size (in pixels).", Top,
       LabelStyle → Directive[FontSize → 12, Bold, FontFamily → "Arial"]]],
     " ", Panel[Labeled[InputField[Dynamic[m2], FieldSize → 10],
       "Enter number of variables.", Top, LabelStyle →
        Directive[FontSize → 12, Bold, FontFamily → "Arial"]]], " ",
     Panel[Labeled[InputField[Dynamic[n2], FieldSize → 10],
       "Enter number of objects.", Top,
       LabelStyle → Directive[FontSize → 12, Bold, FontFamily → "Arial"]]]}],
   Row[{Panel[Labeled[InputField[Dynamic[iterT2], FieldSize → 10],
       "No. of replicate iterations (Monte Carlo or bootstrapped only).",
       Top, LabelStyle → Directive[FontSize → 12,
         Bold, FontFamily → "Arial"]]], " ",
     Panel[Labeled[PopupMenu[Dynamic[statTestT2],
        {1 → "Standard Parametric (F-ratio) Distributions",
         2 → "Monte Carlo Simulated (F-ratio) Distributions ",
         3 → "Bootstrapped Modelled (F-ratio) Distributions"}],
       "Choose estimation procedure.", Top, LabelStyle →
        Directive[FontSize → 12, Bold, FontFamily → "Arial"]]]}]}, Center],
  "Hotelling's T^2 Mean Vector Test Control Parameters",
  Top,
  LabelStyle →
   Directive[FontSize → 14,
    Bold, FontFamily → "Arial"]]]
hSize2 = 500; nobins = 25; iterT2 = 1000; statTestT2 = 1;
```

Out[∘]=
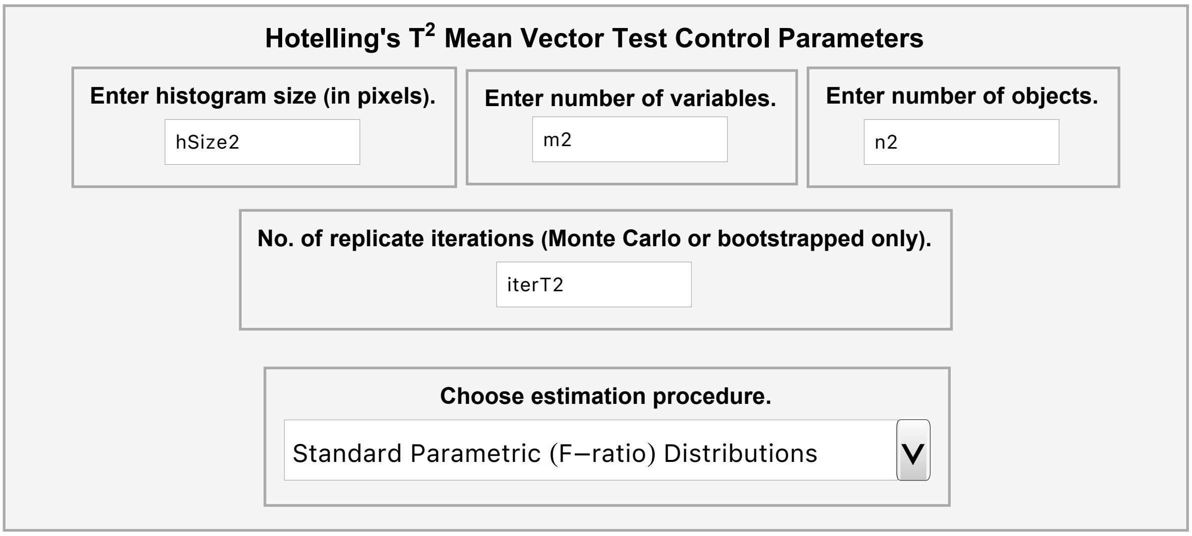

Calculate and display probability statistics

In[∘]:=
```
groupNames = Union[Group];
numGroups = Length[groupNames];
groupPosns = Table[Flatten[Position[Group, groupNames[[i]], 1]], {i, numGroups}];
```

```
groups = N[Table[x2⟦groupPosns⟦j⟧⟧, {j, numGroups}]];
{gpn1, m2} = Dimensions[groups⟦1⟧];
{gpn2, m2} = Dimensions[groups⟦2⟧];
c1 = Covariance[groups⟦1⟧];
c2 = Covariance[groups⟦2⟧];
c = (((gpn1 - 1) * c1) + ((gpn2 - 1) * c2)) / (gpn1 + gpn2 - 2);
cinv = PseudoInverse[c];
meanG1 = Mean[groups⟦1⟧];
meanG2 = Mean[groups⟦2⟧];
t1 = gpn1 * gpn2;
t2 = meanG1 - meanG2;
t4 = meanG1 - meanG2;
t5 = gpn1 + gpn2;
T2 = ((t1 * t2).cinv.t4) / t5;
FT2 = (gpn1 + gpn2 - m2 - 1) * T2 / ((gpn1 + gpn2 - 2) * m2);
num = m2;
denom = gpn1 + gpn2 - m2 - 1;
probT2 = N[(1 - CDF[FRatioDistribution[num, denom], FT2])];
pcentProbT2 = probT2 * 100;

If[statTestT2 == 1,
  FTable = Table[0, {4}, {2}];
  FTable⟦1, 1⟧ = "Hotelling's T²";
  FTable⟦2, 1⟧ = "Observed F-Ratio";
  FTable⟦3, 1⟧ = "Degrees of Freedom";
  FTable⟦4, 1⟧ = "Probability (%)";
  FTable⟦1, 2⟧ = PaddedForm[T2, {4, 3}];
  FTable⟦2, 2⟧ = PaddedForm[FT2, {5, 3}];
  FTable⟦3, 2⟧ =
   StringJoin[ToString[Round[num]], ",", ToString[Round[denom]]];
  FTable⟦4, 2⟧ = PaddedForm[pcentProbT2, {5, 3}];
  If[pcentProbT2 ≤ 5.0, hcolor = Green, hcolor = Red];
  hLegend = Labeled[
    Grid[FTable, BaseStyle → Directive[FontSize → 10, FontFamily → "Arial"],
     Alignment → {{Left, Right}}, Frame → True,
     Dividers → {{True, True}, {True}}], "Hotelling's T² Table", Top,
    LabelStyle → Directive[FontSize → 12, Bold, FontFamily → "Arial"]];
  plotT2 = Plot[PDF[FRatioDistribution[num, denom], x] // Evaluate,
    {x, 0, FT2 + 10}, PlotRange → Full, Filling → Axis, Exclusions → None,
    ImageSize → hSize2, Filling → Bottom, FillingStyle → hcolor,
    PlotRange → Full, AxesLabel → {"F-Ratio", "Frequency"},
    LabelStyle → Directive[Black, FontSize → 12, FontFamily → "Arial"]]];

Panel[
 Labeled[ProgressIndicator[Dynamic[it], {1, iterT2}], "Calculation Progress",
  Top, LabelStyle → Directive[FontSize → 12, Bold, FontFamily → "Arial"]]]
```

```
If[statTestT2 > 1,
  avg = Mean[x2];
  stDev = N[StandardDeviation[x2]];
  groupNames = Union[Group];
  numGroups = Length[groupNames];
  groupPosns =
   Table[Flatten[Position[Group, groupNames⟦i⟧, 1]], {i, numGroups}];
  x3 = Table[0.0, {n1}, {m1}];
  simFRatioT2 = Table[0.0, {iterT2}];

  Do[
   If[statTestT2 == 2,
    Do[
     Do[
      Do[
       in = groupPosns⟦k, i⟧;
       x3⟦in, j⟧ = RandomReal[NormalDistribution[avg⟦j⟧, stDev⟦j⟧]], {j, m1}],
      {i, smpSize⟦k⟧}], {k, kg}]];
   If[statTestT2 == 3,
    x3 = x2;
    Do[x3⟦i⟧ = N[x2⟦RandomInteger[{1, n2}]⟧], {i, n2}]];
   simgp1 = Table[x3⟦groupPosns⟦j⟧⟧, {j, numGroups}];
   {simgpn1, m2} = Dimensions[simgp1⟦1⟧];
   {simgpn2, m2} = Dimensions[simgp1⟦2⟧];
   simc1 = Covariance[simgp1⟦1⟧];
   simc2 = Covariance[simgp1⟦2⟧];
   simc =
    (((simgpn1 - 1) * simc1) + ((simgpn2 - 1) * simc2)) / (simgpn1 + simgpn2 - 2);
   simcinv = PseudoInverse[simc];
   simmeanG1 = Mean[simgp1⟦1⟧];
   simmeanG2 = Mean[simgp1⟦2⟧];
   simt1 = simgpn1 * simgpn2;
   simt2 = simmeanG1 - simmeanG2;
   simt4 = simmeanG1 - simmeanG2;
   simt5 = simgpn1 + simgpn2;
   simT2 = ((simt1 * simt2).simcinv.simt4) / simt5;
   simFT2 = (simgpn1 + simgpn2 - m2 - 1) * simT2 / ((simgpn1 + simgpn2 - 2) * m2);
   simFRatioT2⟦it⟧ = simFT2, {it, iterT2}];

  simFRatioT2 = Sort[simFRatioT2];
  knt = 0;
  Do[If[simFRatioT2⟦i⟧ > FT2, knt = knt + 1], {i, iterT2}];
  simProbT2 = (N[knt / iterT2]) * 100.0;

  FTable = Table[0, {5}, {2}];
  FTable⟦1, 1⟧ = "Hotelling's T²";
  FTable⟦2, 1⟧ = "Observed F-Ratio";
```

```
FTable[[3, 1]] = "Degrees of Freedom";
FTable[[4, 1]] = "No. of Iterations";
FTable[[5, 1]] = "Probability (%)";
FTable[[1, 2]] = PaddedForm[T2, {4, 3}];
FTable[[2, 2]] = PaddedForm[FT2, {5, 3}];
FTable[[3, 2]] =
  StringJoin[ToString[Round[num]], ",", ToString[Round[denom]]];
FTable[[4, 2]] = iterT2;
FTable[[5, 2]] = PaddedForm[simProbT2, {5, 3}];
If[simProbT2 ≤ 5.0, hcolor = Green, hcolor = Red];
hLegend = Labeled[
   Grid[FTable, BaseStyle → Directive[FontSize → 10, FontFamily → "Arial"],
    Alignment → {{Left, Right}}, Frame → True,
    Dividers → {{True, True}, {True}}], "Hotelling's T^2 Table", Top,
   LabelStyle → Directive[FontSize → 12, Bold, FontFamily → "Arial"]];
plotT2 = Histogram[simFRatioT2, "FreedmanDiaconis",
   ChartStyle → hcolor, ImageSize → hSize2, PlotRange → Full,
   LabelStyle → Directive[FontSize → 12, Black, FontFamily → "Arial"],
   AxesLabel → {"F-Ratio", "Frequency"}, ImageSize → hSize]];

label1 = "Parametric Probability Results";
label2 = "Monte Carlo Simulation Results";
label3 = "Bootstrap Modelling Results";
probPlot1 = Labeled[Overlay[{plotT2, hLegend}, Alignment → Right],
  ToExpression[StringJoin["label", ToString[statTestT2]]], Top,
  LabelStyle → Directive[FontSize → 18, Bold, FontFamily → "Arial"]]
```

Out[∘]=

Calculation Progress

Export mean vector test results

```
In[∘]:= filenameout = SystemDialogInput["FileSave"];
Export[filenameout, probPlot1, "TIFF", ImageResolution → 150]
```

Out[∘]= /Users/n.macleod/Desktop/Bumpus Sparrow Results/Hotelling's T2 Test (BS).tif

Export mean vector simulation values (Monte Carlo & bootstrap procedures only)

```
In[∘]:= filenameout = SystemDialogInput["FileSave"];
Export[filenameout, simFRatioT2, "CSV", "TextDelimiters" → ""]
```

Out[∘]= /Users/n.macleod/Desktop/PalAss 2018/Dried Leaf Results/Single Species Results I/Aesculus chinensis/CVA Results/Hotelling's T2 Test (BS).tif

Test Two – Group Separation w/ the Log – Likelihood statistic.

Specify testing options

Standard (χ^2 – ratio) Distribution =

use this test if your data are normally distributed and the group covariance matrices are equal
Monte Carlo Simulated Distribution =
use this test if your data are not normally distributed and/or the group covariance matrices are not equal
Bootstrap Simulated Distribution =
use this test if your data are not normally distributed and/or the group covariance matrices are not equal

In[•]:=
```
Panel[
 Labeled[Column[{Row[{Panel[Labeled[InputField[Dynamic[hSize2], FieldSize → 10],
        "Enter histogram size (in pixels).", Top,
        LabelStyle → Directive[FontSize → 12, Bold, FontFamily → "Arial"]]],
      " ", Panel[Labeled[InputField[Dynamic[m2], FieldSize → 10],
        "Enter number of variables.", Top, LabelStyle →
         Directive[FontSize → 12, Bold, FontFamily → "Arial"]]], " ",
      Panel[Labeled[InputField[Dynamic[n2], FieldSize → 10],
        "Enter number of objects.", Top,
        LabelStyle → Directive[FontSize → 12, Bold, FontFamily → "Arial"]]]}],
    Row[{Panel[Labeled[InputField[Dynamic[iterLLR], FieldSize → 10],
        "No. of replicate iterations (Monte Carlo or bootstrapped only).",
        Top, LabelStyle → Directive[FontSize → 12,
          Bold, FontFamily → "Arial"]]], " ",
      Panel[Labeled[PopupMenu[Dynamic[statTestLLR],
         {1 → "Standard Parametric (χ2-ratio) Distributions",
          2 → "Monte Carlo Simulated (χ2-ratio) Distributions ",
          3 → "Bootstrapped Modelled (χ2-ratio) Distributions"}],
        "Choose estimation procedure.", Top, LabelStyle →
         Directive[FontSize → 12, Bold, FontFamily → "Arial"]]]}]}, Center],
  "Log-Likelihood Mean Vector Test Control Parameters",
  Top,
  LabelStyle →
   Directive[FontSize → 14,
    Bold, FontFamily → "Arial"]]]
hSize2 = 500; nobins = 25; iterLLR = 1000; statTestLLR = 1;
```

Out[∘]=

Log–Likelihood Mean Vector Test Control Parameters

Enter histogram size (in pixels).	Enter number of variables.	Enter number of objects.
hSize2	m2	n2

No. of replicate iterations (Monte Carlo or bootstrapped only).

iterLLR

Choose estimation procedure.

Standard Parametric (χ^2–ratio) Distributions

Calculate Log – Likelihood Ratio

In[∘]:=

```
If[statTestLLR == 1,
  phi = (n2 - 1 - (0.5 * (m2 + nGps))) * (Log[(Det[T] / Det[W])]);
  dof = m2 * (nGps - 1);
  prob = N[(1 - CDF[ChiSquareDistribution[m2 * (nGps - 1)], phi]) * 100];
  FTable = Table[" ", {3}, {2}];
  FTable[[1, 1]] = "Log Likelihood Ratio";
  FTable[[2, 1]] = "Degrees of Freedom";
  FTable[[3, 1]] = "Probability (%)";
  FTable[[1, 2]] = PaddedForm[phi, {4, 3}];
  FTable[[2, 2]] = dof;
  FTable[[3, 2]] = PaddedForm[prob, {5, 3}];
  rng = phi * 2;
  If[prob ≤ 5.0, hcolor = Green, hcolor = Red];
  phiLegend = Labeled[
    Grid[FTable, BaseStyle → Directive[FontSize → 10, FontFamily → "Arial"],
     Alignment → {{Left, Right}}, Frame → True,
     Dividers → {{True, True}, {True}}], "Likelihood Ratio Table", Top,
    LabelStyle → Directive[FontSize → 12, Bold, FontFamily → "Arial"]];
  plot5 = Plot[PDF[ChiSquareDistribution[dof], x], {x, 0, rng},
    ImageSize → hSize2, Filling → Bottom, FillingStyle → hcolor,
    PlotRange → Full, AxesLabel → {"χ² Ratio", "Prob. Density"},
    LabelStyle → Directive[Black, FontSize → 12, FontFamily → "Arial"]];
  probPlot5 = Overlay[{plot5, phiLegend}, Alignment → Right];
  label = "Parametric Probability Results"];

If[statTestLLR > 1, Panel[
  Labeled[ProgressIndicator[Dynamic[it], {1, iterLLR}], "Calculation Progress",
   Top, LabelStyle → Directive[FontSize → 12, Bold, FontFamily → "Arial"]]]]

If[statTestLLR > 1,
```

```
avg = Mean[x2];
stDev = N[StandardDeviation[x2]];
groupNames = Union[Group];
numGroups = Length[groupNames];
groupPosns = Table[Flatten[Position[Group, groupNames⟦i⟧, 1]], {i, kg}];
x3 = Table[0.0, {n2}, {m2}];
simRatios = Table[0.0, {iterLLR}];

phiTest = (n2 - 1 - (0.5 * (m2 + kg))) * (Log[(Det[T] / Det[W])]);
chi2Phi = phiTest;

Do[
 Label[123];
 If[statTestLLR == 2,
  Do[
   Do[
    Do[
     inum = groupPosns⟦k, i⟧;
     x3⟦inum, j⟧ = RandomReal[NormalDistribution[avg⟦j⟧, stDev⟦j⟧]],
     {i, smpSize⟦k⟧}], {j, m2}], {k, kg}]];
 If[statTestLLR == 3,
  x3 = x2;
  Do[x3⟦i⟧ = N[x2⟦RandomInteger[{1, n2}]⟧], {i, n2}]];
 {gndMean, simT, gNames, nGps, gpMeans, smpSize, simW, simB} =
  TWB[x3, n2, m2, Group];
 simPhi = (n2 - 1 - (0.5 * (m2 + kg))) * (Log[(Det[simT] / Det[simW])]);
 simRatios⟦it⟧ = simPhi;
 If[Head[simRatios⟦it⟧] == Complex || simRatios⟦it⟧ < 0.0, Goto[123]],
 {it, iterLLR}];

simRatios = Sort[simRatios];
simProb = Table[0.0, {3}];
simProb⟦1⟧ = phiTest;
simProb⟦2⟧ = chi2Phi;
knt = 0;
Do[If[simRatios⟦i⟧ > phiTest, knt = knt + 1], {i, iterLLR}];
simProb⟦3⟧ = N[knt / iterLLR] * 100;

FTable = Table[" ", {4}, {2}];
FTable⟦1, 1⟧ = "Log Likelihood Ratio";
FTable⟦2, 1⟧ = "Degrees of Freedom";
FTable⟦3, 1⟧ = "No. of Iterations";
FTable⟦4, 1⟧ = "Probability (%)";
FTable⟦1, 2⟧ = PaddedForm[phiTest, {4, 3}];
FTable⟦2, 2⟧ = m2 * (kg - 1);
FTable⟦3, 2⟧ = iterLLR;
FTable⟦4, 2⟧ = PaddedForm[simProb⟦3⟧, {5, 3}];
```

```
  If[simProb⟦3⟧ ≤ 5.0, hcolor = Green, hcolor = Red];

  If[simProb⟦3⟧ ≥ 5.0, rng = phiTest * 2, rng = phiTest];
  phiLegend = Labeled[
    Grid[FTable, BaseStyle → Directive[FontSize → 10, FontFamily → "Arial"],
     Alignment → {{Left, Right}}, Frame → True,
     Dividers → {{True, True}, {True}}], "Likelihood Ratio Table", Top,
    LabelStyle → Directive[FontSize → 12, Bold, FontFamily → "Arial"]];
  plot5 = Histogram[simRatios, "FreedmanDiaconis",
    ChartStyle → hcolor, ImageSize → hSize2, PlotRange → Full,
    LabelStyle → Directive[FontSize → 12, Black, FontFamily → "Arial"],
    AxesLabel → {"χ² Ratio", "Frequency"}, ImageSize → hSizeMVT];
  probPlot5 = Overlay[{plot5, phiLegend}, Alignment → Right];
  If[statTestLLR == 2, label = "Monte Carlo Simulation Results"];
  If[statTestLLR == 3, label = "Bootstrap Modelling Results"]];

probPlot = Labeled[probPlot5, label, Top,
  LabelStyle → Directive[FontSize → 18, Bold, FontFamily → "Arial"]]
```

Export mean vector test results

```
In[∘]:= filenameout = SystemDialogInput["FileSave"];
        Export[filenameout, probPlot, "TIFF", ImageResolution → 150]

Out[∘]= /Users/n.macleod/Desktop/Bumpus Sparrow Results/Log Likelihood Test (BS).tif
```

Export mean vector simulation values (Monte Carlo & bootstrap procedures only)

```
In[∘]:= filenameout = SystemDialogInput["FileSave"];
        Export[filenameout, simRatios, "CSV", "TextDelimiters" → ""]

Out[∘]= /Users/nm/Desktop/Lupus II/Dorsal/Eigenimage
          (Dorsal)/CVA Results/Log Likelihood phi (BS) values.csv
```

Create 2 D scatterplot (use only for datasets containing three or more groups).

Specify 2 D plot options.
You must run this code after you read in the data so it can pick up the proper variable names.

```
In[•]:= cvNames = Table[StringJoin["CV-", ToString[i]], {i, noAxes}];
Panel[
 Labeled[Column[{Row[{Panel[Labeled[PopupMenu[Dynamic[xAxisName], cvNames],
       "Select variable to be plotted on x-Axis.", Top, LabelStyle →
        Directive[FontSize → 12, Bold, FontFamily → "Arial"]]], "  ",
     Panel[Labeled[PopupMenu[Dynamic[yAxisName], cvNames],
       "Select variable to be plotted on y-Axis.", Top,
       LabelStyle → Directive[FontSize → 12, Bold, FontFamily → "Arial"]]]}],
   Row[{
     Panel[Labeled[PopupMenu[Dynamic[pltAspect],
        {1 → "Golden Ratio Plot", 2 → "Square Plot (equi-length axes)",
         3 → "True-Scale Plot (actual axis scales)"}],
       "Enter plot aspect ratio type.", Top, LabelStyle →
        Directive[FontSize → 12, Bold, FontFamily → "Arial"]]], "  ",
     Panel[Labeled[PopupMenu[Dynamic[lch], {1 → "Simple scatterplot",
         2 → "Scatterplot w/ convex hulls"}],
       "Show group domians?", Top, LabelStyle →
        Directive[FontSize → 12, Bold, FontFamily → "Arial"]]], "  ",
     Panel[Labeled[PopupMenu[Dynamic[ptsJoin], {1 → "No", 2 → "Yes"}],
       "Join datapoints?", Top,
       LabelStyle → Directive[FontSize → 12, Bold, FontFamily → "Arial"]]]}],
   Row[{Panel[Labeled[InputField[Dynamic[pltSize], FieldSize → 10],
       "Enter plot size value.", Top,
       LabelStyle → Directive[FontSize → 12, Bold, FontFamily → "Arial"]]],
     "  ", Panel[Labeled[InputField[Dynamic[pltPad], FieldSize → 10],
       "Enter plot margin padding value.", Top, LabelStyle →
        Directive[FontSize → 12, Bold, FontFamily → "Arial"]]], "  ",
     Panel[Labeled[InputField[Dynamic[iconSize], FieldSize → 10],
       "Enter plot icon size value.", Top, LabelStyle →
        Directive[FontSize → 12, Bold, FontFamily → "Arial"]]]}]}, Center],
  "2D Plot Options", Top, LabelStyle → Directive[FontSize → 18,
    Bold, FontFamily → "Arial"]]]
pltSize = 500; iconSize = 0.02; pltPad = 0.1; xAxisName = cvNames⟦1⟧;
yAxisName = cvNames⟦2⟧;
 ptsJoin = 1; dataTrans = 1;  pltAspect = 1 ; lch = 2;
```

Out[∘]=

2D Plot Options

Select variable to be plotted on x–Axis.

CV–1

Select variable to be plotted on y–Axis.

CV–1

Enter plot aspect ratio type.

Golden Ratio Plot

Show group domians?

Simple scatterplot

Join datapoints?

No

Enter plot size value.

pltSize

Enter plot margin padding value.

pltPad

Enter plot icon size value.

iconSize

Plot script

In[∘]:=

```
Do[If[xAxisName == cvNames⟦j⟧, axis1 = j], {j, noAxes}];
Do[If[yAxisName == cvNames⟦j⟧, axis2 = j], {j, noAxes}];

groupNames = Union[Group];
numGroups = Length[groupNames];
groupPosns = Table[Flatten[Position[Group, groupNames⟦i⟧, 1]], {i, numGroups}];
eScoresT = Transpose[eScores];

xAxis = eScoresT⟦axis1⟧; yAxis = eScoresT⟦axis2⟧;
lab1 = StringJoin[{"Canonical Variate ", ToString[axis1]},
   {" (Var. =", ToString[t3⟦axis1 + 1, 3⟧]}, {"%)"}];
lab2 = StringJoin[{"Canonical Variate ", ToString[axis2]},
   {" (Var. =", ToString[t3⟦axis2 + 1, 3⟧]}, {"%)"}];
maxx =
 Max[
  xAxis];
minx = Min[xAxis];
maxy = Max[yAxis];
miny = Min[yAxis];

If[pltAspect == 1 || pltAspect == 3,
  xPlotLow = minx; xPlotHi = maxx; yPlotLow = miny; yPlotHi = maxy];
If[pltAspect == 2,
  If[minx > miny,
   xPlotLow = miny; yPlotLow = miny,
   xPlotLow = minx; yPlotLow = minx ]];
```

```
If[pltAspect == 2,
  If[maxx < maxy,
   xPlotHi = maxy; yPlotHi = maxy,
   xPlotHi = maxx; yPlotHi = maxx]];
If[pltAspect == 1, aRatio = 1 / N[GoldenRatio]];
If[pltAspect == 2, aRatio = 1];
If[pltAspect == 3, aRatio = Automatic];

tmpPoints = Transpose[List[xAxis, yAxis]];
pltPoints = Table[tmpPoints⟦groupPosns⟦j⟧⟧, {j, numGroups}];
iconList = Flatten[Table[
    {Graphics[{EdgeForm[{Thickness[0.003], Black}],
       Hue[N[((numGroups + 1) - j) / numGroups]],
       Disk[{0, 0}, Scaled[iconSize]]}]}, {j, numGroups}]];

If[lch == 1 || ptsJoin == 1,
 Do[
  pltTable⟦k, 1⟧ = pTmp =
    ListPlot[pltPoints⟦k⟧, AspectRatio → aRatio, Frame → True, Joined → False,
     Axes → False, PlotRange → {{xPlotLow, xPlotHi}, {yPlotLow, yPlotHi}},
     PlotRangePadding → Scaled[pltPad], Ticks → Automatic, FrameLabel →
      {lab1, lab2}, PlotMarkers → iconList⟦k⟧, ImageSize → pltSize, LabelStyle →
      Directive[FontSize → 14, Black, FontFamily → "Arial"]], {k, numGroups}],
 Do[
  pltTable⟦k, 1⟧ =
   ListPlot[pltPoints⟦k⟧, Frame → True, Axes → False, AspectRatio → aRatio,
    PlotRange → {{xPlotLow, xPlotHi}, {yPlotLow, yPlotHi}}, PlotRangePadding →
     Scaled[pltPad], Ticks → Automatic, FrameLabel → {lab1, lab2},
    LabelStyle → Directive[Black, FontSize → 14, FontFamily → "Arial"],
    ImageSize → pltSize, PlotStyle → Directive[Disk[],
      Hue[N[((numGroups + 1) - k) / numGroups]], EdgeForm[{Thickness[1.0], Black}],
      PointSize[Scaled[iconSize - 0.009]]]], {k, numGroups}]]

If[ptsJoin == 2,
  Do[
   pltTable⟦k, 2⟧ = ListLinePlot[pltPoints⟦k⟧,
     AspectRatio → aRatio, Frame → True, Joined → True, Axes → False,
     PlotStyle → Directive[Hue[N[((numGroups + 1) - k) / numGroups]], Thin],
     PlotRange → {{xPlotLow, xPlotHi}, {yPlotLow, yPlotHi}},
     PlotRangePadding → Scaled[pltPad], Ticks → Automatic,
     FrameLabel → {lab1, lab2}, ImageSize → pltSize, LabelStyle →
      Directive[FontSize → 14, Black, FontFamily → "Arial"]], {k, numGroups}]];

If[lch == 2,
  Do[
   hull = ConvexHullMesh[pltPoints⟦k⟧];
   pltTable⟦k, 3⟧ = HighlightMesh[hull,
```

```
        Style[2, Opacity[0.2], Hue[N[((numGroups + 1) - k) / numGroups]]],
        Frame → True, Axes → False, AspectRatio → aRatio,
        PlotRange → {{xPlotLow, xPlotHi}, {yPlotLow, yPlotHi}}, PlotRangePadding →
         Scaled[pltPad], Ticks → Automatic, FrameLabel → {lab1, lab2},
        LabelStyle → Directive[Black, FontSize → 14, FontFamily → "Arial"],
        ImageSize → pltSize], {k, numGroups}]];

If[ptsJoin == 1 && lch == 1, p0 = Show[pltTable[[All, 1]]]];
If[ptsJoin == 2 && lch == 1, p0 = Show[pltTable[[All, 2]], pltTable[[All, 1]]]];
If[ptsJoin == 1 && lch == 2, p0 = Show[pltTable[[All, 3]], pltTable[[All, 1]]]];
If[ptsJoin == 2 && lch == 2,
  p0 = Show[pltTable[[All, 3]], pltTable[[All, 2]], pltTable[[All, 1]]]];

p1 = Labeled[p0, "           CV Score Plot", Top,
   LabelStyle → Directive[FontSize → 18, Bold, FontFamily → "Arial"]];
g1 = Grid[Table[
     {Graphics[{EdgeForm[{Thin, Black}], Hue[N[((numGroups + 1) - j) / numGroups]],
        Disk[]}, ImageSize → 13]}, {j, numGroups}], Frame → False];
g2 = Grid[Partition[groupNames, 1], Alignment → Left,
   BaseStyle → {FontFamily → "Arial", FontSize → 13, Italic}];
p2 = Labeled[Text[Grid[{{g1, g2}}, Alignment → Bottom, Frame → True]], "Legend",
   Top, LabelStyle → Directive[Black, FontSize → 18, Bold, FontFamily → "Arial"]];

plt2D = Grid[{{p1, p2}}, BaselinePosition → Top, Alignment → Top]
```

Export current 2 D plot.

```
In[]:= filenameout = SystemDialogInput["FileSave"];
       Export[filenameout, plt2D, "TIFF", ImageResolution → 150]

Out[]= /Users/n.macleod/Documents/Iris Results/CV-1 vs CV-2.tif
```

Reverse eigenvector axes & recalculate scores.

```
If[eMethod == 1, eVecs = eVecs * -1, eVecs = w * -1];
eScores = x2.eVecs;
```

Label plotted points.

In[•]:=
```
pn1 = p0;
namePoints = tmpPoints;
tempPointsT = Transpose[tmpPoints];
mxY = Max[tempPointsT[[2]]];
mnY = Min[tempPointsT[[2]]];
incY = N[(mxY - mnY) / 15];
Do[namePoints[[i, 2]] = tmpPoints[[i, 2]] - incY, {i, n2}]
nPointsTable = Table[{Text[objNames[[i]], namePoints[[i]], {-1, 0}]}, {i, n2}];
pn2 = Graphics[nPointsTable, Frame → True, AspectRatio → aRatio , Axes → False,
    FrameLabel → {lab1, lab2}, PlotRangePadding → Scaled[pltPad], BaseStyle →
     Directive[FontSize → 12, FontFamily → "Arial"], ImageSize → pltSize];

p1 = Labeled[Show[pn1, pn2, BaseStyle → {FontFamily → "Arial"}],
    "           CV Score Plot", Top,
    LabelStyle → Directive[FontSize → 18, Bold, FontFamily → "Arial"]];

Plt2D = Grid[{{p1, p2}}, BaselinePosition → Top, Alignment → Top]
```

Export current 2 D plot.

In[•]:=
```
filenameout = SystemDialogInput["FileSave"];
Export[filenameout, Plt2D, "TIFF", ImageResolution → 150]
```

Out[•]=
```
/Users/n.macleod/Desktop/Vases/Whole Dataset/CVA Results/CV-3 vs CV-2.tif
```

Create 3 D scatterplot (use only for datasets containing four or more groups).

Specify 3 D plot options.
You must run this code after you read in the data so it can pick up the proper variable names.

```
In[•]:= cvNames = Table[StringJoin["CV-", ToString[i]], {i, noAxes}];
Panel[
 Labeled[Column[{Row[{Panel[Labeled[PopupMenu[Dynamic[xAxisName], cvNames],
        "Select variable to be plotted on x-Axis.", Top, LabelStyle →
         Directive[FontSize → 12, Bold, FontFamily → "Arial"]]], "  ",
      Panel[Labeled[PopupMenu[Dynamic[yAxisName], cvNames],
        "Select variable to be plotted on y-Axis.", Top, LabelStyle →
         Directive[FontSize → 12, Bold, FontFamily → "Arial"]]], "  ",
      Panel[Labeled[PopupMenu[Dynamic[zAxisName], cvNames],
        "Select variable to be plotted on z-Axis.", Top,
        LabelStyle → Directive[FontSize → 12, Bold, FontFamily → "Arial"]]]}],
    Row[{
      Panel[Labeled[PopupMenu[Dynamic[pltAspect],
         {1 → "Golden Ratio Plot", 2 → "Square Plot (equi-length axes)",
          3 → "True-Scale Plot (actual axis scales)"}],
        "Enter plot aspect ratio type.", Top, LabelStyle →
         Directive[FontSize → 12, Bold, FontFamily → "Arial"]]], "  ",
      Panel[Labeled[PopupMenu[Dynamic[lch], {1 → "Simple scatterplot",
          2 → "Scatterplot w/ convex hulls"}],
        "Show group domians?", Top, LabelStyle →
         Directive[FontSize → 12, Bold, FontFamily → "Arial"]]], " ",
      Panel[Labeled[PopupMenu[Dynamic[ptsJoin], {1 → "No", 2 → "Yes"}],
        "Join datapoints?", Top,
        LabelStyle → Directive[FontSize → 12, Bold, FontFamily → "Arial"]]]}],
    Row[{Panel[Labeled[InputField[Dynamic[pltSize], FieldSize → 5],
        "Enter plot size value.", Top,
        LabelStyle → Directive[FontSize → 12, Bold, FontFamily → "Arial"]]],
      " ", Panel[Labeled[InputField[Dynamic[pltPad], FieldSize → 5],
        "Enter plot margin padding value.", Top, LabelStyle →
         Directive[FontSize → 12, Bold, FontFamily → "Arial"]]], " ",
      Panel[Labeled[InputField[Dynamic[icSz3D], FieldSize → 5],
        "Enter plot icon size value.", Top, LabelStyle →
         Directive[FontSize → 12, Bold, FontFamily → "Arial"]]]}]}, Center],
  "3D Plot Options", Top, LabelStyle → Directive[FontSize → 18,
    Bold, FontFamily → "Arial"]]]
pltSize = 500; icSz3D = 60; pltPad = 0.1; xAxisName = cvNames[[1]];
 yAxisName = cvNames[[2]];
zAxisName = cvNames[[3]]; ptsJoin = 1;
dataTrans = 1;
pltAspect = 3 ; lch = 2;
```

Out[∘]=

3D Plot Options

Select variable to be plotted on x–Axis.

CV–1

Select variable to be plotted on y–Axis.

CV–1

Select variable to be plotted on z–Axis.

CV–1

Enter plot aspect ratio type.

Golden Ratio Plot

Show group domians?

Simple scatterplot

Join datapoints?

No

Enter plot size value.

pltSize

Enter plot margin padding value.

pltPad

Enter plot icon size value.

icSz3D

Plot script

In[∘]:=

```
Do[If[xAxisName == cvNames[[j]], axis1 = j], {j, noAxes}]
Do[If[yAxisName == cvNames[[j]], axis2 = j], {j, noAxes}]
Do[If[zAxisName == cvNames[[j]], axis3 = j], {j, noAxes}]

groupNames = Union[Group];
numGroups = Length[groupNames];
groupPosns = Table[Flatten[Position[Group, groupNames[[i]], 1]], {i, numGroups}];
eScoresT = Transpose[eScores];

xAxis = eScoresT[[axis1]];
yAxis = eScoresT[[axis2]];
zAxis = eScoresT[[axis3]];
lab1 = StringJoin["CV-", ToString[axis1]];
lab2 = StringJoin["CV-", ToString[axis2]];
lab3 = StringJoin["CV-", ToString[axis3]];
maxx = Max[xAxis];
minx = Min[xAxis];
maxy = Max[yAxis];
miny = Min[yAxis];
maxz = Max[zAxis]; minz = Min[zAxis];

mxax = Max[xAxis];
```

```
mnax = Min[xAxis];
If[pltAspect < 3, f = N[icSz3D * 0.020]];
If[pltAspect == 3, f = N[icSz3D * 0.0005]];
iconSize3D = (mxax - mnax) * f;

If[pltAspect == 1 || pltAspect == 3,
  xPlotLow = minx; xPlotHi = maxx; yPlotLow = miny; yPlotHi = maxy];
If[pltAspect == 2,
  If[minx > miny,
   xPlotLow = miny; yPlotLow = miny,
   xPlotLow = minx; yPlotLow = minx ]];
If[pltAspect == 2,
  If[maxx < maxy,
   xPlotHi = maxy; yPlotHi = maxy,
   xPlotHi = maxx; yPlotHi = maxx]];

If[pltAspect == 1, bRatio = {1.61803, 1, 1}];
If[pltAspect == 2, bRatio = {1, 1, 1}];
If[pltAspect == 3, bRatio = Automatic];

points3 = Transpose[List[xAxis, yAxis, zAxis]];
gp1 = groupPosns;
h = Table[0, {n2}];
Do[
 tmp = gp1[[i]];
 itr = Length[tmp];
 Do[h[[tmp[[j]]]] = Hue[N[((numGroups + 1) - i) / numGroups]], {j, itr}],
 {i, numGroups}]

If[pltAspect == 3,
  pltPoints = Table[{h[[i]], Sphere[points3[[i]], iconSize3D]}, {i, n2}];
  p0 = Graphics3D[pltPoints, Axes → True,
    Boxed → True, PlotRangePadding → Scaled[pltPad],
    LabelStyle → Directive[FontSize → 12, Black, FontFamily → "Arial"],
    AxesLabel → {lab1, lab2, lab3}, ImageSize → pltSize, BoxRatios → Automatic]];
If[pltAspect ≠ 3,
  pltPoints =
   Table[{h[[i]], AbsolutePointSize[iconSize3D], Point[points3[[i]]]}, {i, n2}];
  p0 = Graphics3D[pltPoints, Axes → True, Boxed → True,
    PlotRangePadding → Scaled[pltPad],
    LabelStyle → Directive[FontSize → 12, Black, FontFamily → "Arial"],
    AxesLabel → {lab1, lab2, lab3}, ImageSize → pltSize, BoxRatios → bRatio]];
If[ptsJoin == 2,
  pltLineTable = Table[" ", {numGroups}];
  gpPoints = Table[points3[[groupPosns[[j]]]], {j, numGroups}];
  Do[
   pltLineTable[[k]] =
```

```
    Graphics3D[{Hue[N[((numGroups + 1) - k) / numGroups]], Line[gpPoints⟦k⟧]},
     Axes → True, Boxed → True, PlotRangePadding → Scaled[pltPad],
     LabelStyle → Directive[FontSize → 12, Black, FontFamily → "Arial"],
     AxesLabel → {lab1, lab2, lab3}, ImageSize → pltSize,
     BoxRatios → bRatio, ViewPoint → {xax, yax, zax}], {k, numGroups}];
  p0 = Show[{pltLineTable, p0}]];
If[lch == 2,
  pltMeshTable = Table[" ", {numGroups}];
  gpPoints = Table[points3⟦groupPosns⟦j⟧⟧, {j, numGroups}];
  Do[
   {n3, m3} = Dimensions[gpPoints⟦k⟧];
   cHull3D = ConvexHullMesh[gpPoints⟦k⟧, BaseStyle → {EdgeForm[]},
     Boxed → True, Axes → True, PlotRangePadding → Scaled[pltPad],
     LabelStyle → Directive[FontSize → 12, Black, FontFamily → "Arial"],
     AxesLabel → {lab1, lab2, lab3}, ImageSize → pltSize,
     BoxRatios → bRatio, ViewPoint → {xax, yax, zax}];
   pltMeshTable⟦k⟧ = Show[{HighlightMesh[cHull3D, Style[2, Opacity[0.2],
        Hue[N[((numGroups + 1) - k) / numGroups]]]], p0}], {k, numGroups}];
  p0 = Show[pltMeshTable]];

p1 = Labeled[p0, "          CV Score Plot", Top,
   LabelStyle → Directive[FontSize → 18, Bold, FontFamily → "Arial"]];
h2 = Table[0, {numGroups}];
l1 = Table[" ", {numGroups}, {2}];
horiz = Table["Left", numGroups];
vert = Table["Center", numGroups];
Do[h2⟦i⟧ = Hue[N[((numGroups + 1) - i) / numGroups]], {i, numGroups}];
If[pltAspect == 3,
  i1 = Table[{h2⟦i⟧, Sphere[{0, 0, 0}, 0.01]}, {i, numGroups}];
  Do[
   l1⟦i, 1⟧ = Graphics3D[i1⟦i⟧, Boxed → False, ImageSize → 16], {i, numGroups}]];
If[pltAspect ≠ 3,
 i1 = Table[{h2⟦i⟧, EdgeForm[{Directive[{Thickness[0.05]}]}],
    Disk[{0, 0}, 0.001]}, {i, numGroups}];
 Do[l1⟦i, 1⟧ = Graphics[i1⟦i⟧, ImageSize → 12], {i, numGroups}]]
Do[l1⟦i, 2⟧ = Style[groupNames⟦i⟧, FontFamily → "Arial",
    FontSlant → Italic, FontSize → 16], {i, numGroups}];
g2 = Labeled[Grid[l1, Frame → True, Alignment → {horiz, vert},
    Spacings → {1, 0.5}], "Legend", Top,
   LabelStyle → Directive[Black, FontSize → 16, Bold, FontFamily → "Arial"]];

plt3D = Grid[{{p1, g2}}, BaselinePosition → Top, Alignment → Top]
```

Adjust orientation of 3 D plot (if necessary).

You must replot the data to activate the changes. These changes will be able to be exported using the script below.

In[∘]:=
```
Panel[
 Labeled[Row[{Labeled[Slider[Dynamic[xax], {-10, 10}, Appearance → "Labeled"],
      "x-Axis Viewpoint", Top,
      LabelStyle → Directive[FontSize → 10, Bold, FontFamily → "Arial"]] ×
     Labeled[Slider[Dynamic[yax], {-10, 10}, Appearance → "Labeled"],
      "y-Axis Viewpoint", Top,
      LabelStyle → Directive[FontSize → 10, Bold, FontFamily → "Arial"]] ×
     Labeled[Slider[Dynamic[zax], {-10, 10}, Appearance → "Labeled"],
      "z-Axis Viewpoint", Top,
      LabelStyle → Directive[FontSize → 10, Bold, FontFamily → "Arial"]]}],
  "3D Plot Orientation Controls", Top, LabelStyle →
   Directive[FontSize → 14, Bold, FontFamily → "Ariel"]]]
xax = 2.5; yax = -2.5; zax = 2.5;
```

Out[∘]=

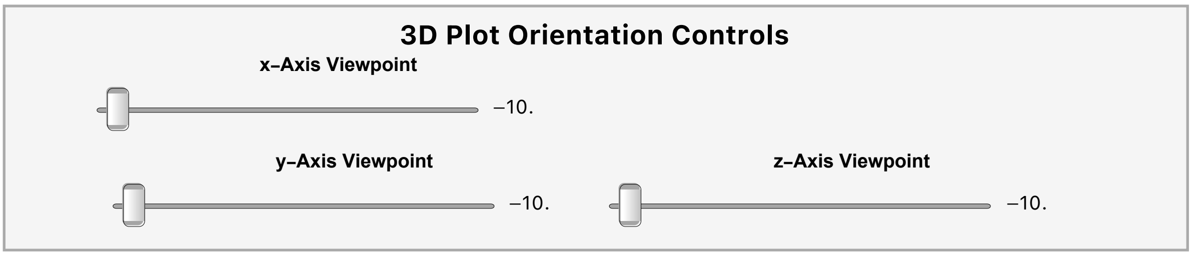

Export current 3 D plot.

In[∘]:=
```
filenameout = SystemDialogInput["FileSave"];
Export[filenameout, plt3D, "TIFF", ImageResolution → 150]
```

Out[∘]=
```
/Users/n.macleod/Projects/Storage/MacLeod/Manuscripts/In
  Progress/Dragonflies/Forewings/Data & Results/Reduced
  Dataset/EES Outline + Landmark Analysis/CVA Results/Water
  Body + Habitat Groups/CV-1 vs CV-2 vs CV-3.tif
```

Estimate statistical significance of group mean –vector differences using multi –group Wilks λ,
Roy' s Maximum Root,
Pillai' s Trace, Hotelling – Lawes Trace and Likelihood Ratio tests

Specify testing options

Standard (F – ratio) Distribution =
use this test if your data are normally distributed and the group covariance matrices are equal

Monte Carlo Simulated Distribution =
use this test if your data are not normally distributed and/or the group covariance matrices are not equal

Bootstrap Simulated Distribution =
use this test if your data are not normally distributed and/or the group covariance matrices are not equal

In[∘]:=
```
Panel[
 Labeled[
  Column[{Row[{Panel[Labeled[InputField[Dynamic[hSizeMVT], FieldSize → 10],
        "Enter histogram size (in pixels).", Top,
        LabelStyle → Directive[FontSize → 12, Bold, FontFamily → "Arial"]]],
      " ", Panel[Labeled[InputField[Dynamic[m2], FieldSize → 10],
        "Enter number of variables.", Top, LabelStyle →
         Directive[FontSize → 12, Bold, FontFamily → "Arial"]]], " ",
      Panel[Labeled[InputField[Dynamic[n2], FieldSize → 10],
        "Enter number of objects.", Top,
        LabelStyle → Directive[FontSize → 12, Bold, FontFamily → "Arial"]]]}],
    Row[{Panel[Labeled[InputField[Dynamic[iter], FieldSize → 10],
        "No. of replicate iterations (Monte Carlo or bootstrapped only).",
        Top, LabelStyle → Directive[FontSize → 12,
          Bold, FontFamily → "Arial"]]], " ",
      Panel[Labeled[PopupMenu[Dynamic[statTest],
         {1 → "Standard Parametric (F-ratio) Distributions",
          2 → "Monte Carlo Simulated (F-ratio) Distributions ",
          3 → "Bootstrapped Modelled (F-ratio) Distributions"}],
        "Choose estimation procedure.", Top, LabelStyle →
         Directive[FontSize → 12, Bold, FontFamily → "Arial"]]]}]}, Center],
  "Multi-Group Mean Vector Test Control Parameters", Top,
  LabelStyle →
   Directive[FontSize → 14,
    Bold, FontFamily → "Arial"]]]
hSizeMVT = 300; nobins = 25; iter = 1000; statTest = 1;
```

Out[∘]=

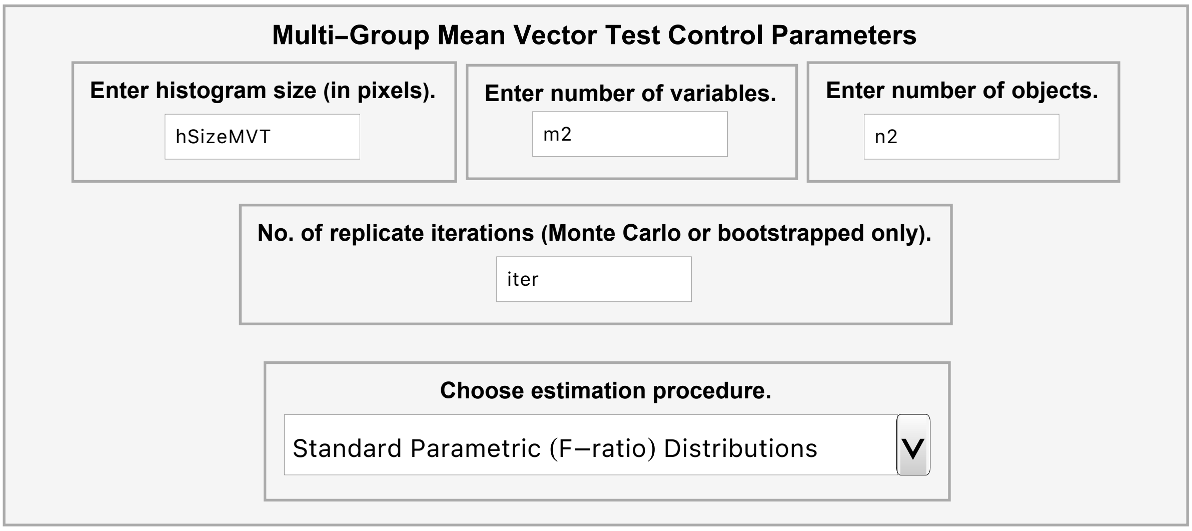

Calculate and display probability statistics

In[∘]:=
```
If[statTest == 1,
  lambda = Det[W] / Det[T];
  df1 = m2 * (kg - 1);
```

```
wCoef = N[n2 - 1 - (m2 + kg) / 2];
tCoef = N[ √((df1^2 - 4) / (m2^2 + (kg - 1)^2 - 5)) ];
df2 = (wCoef * tCoef) - (df1 / 2) + 1;
Flambda = N[((1 - lambda^(1/tCoef)) / lambda^(1/tCoef)) * (df2 / df1)];
prob = N[(1 - CDF[FRatioDistribution[df1, df2], Flambda]) * 100];
FTable = Table[" ", {4}, {2}];
FTable⟦1, 1⟧ = "Wilk's Lambda";
FTable⟦2, 1⟧ = "Observed F-Ratio";
FTable⟦3, 1⟧ = "Degrees of Freedom";
FTable⟦4, 1⟧ = "Probability (%)";
FTable⟦1, 2⟧ = PaddedForm[lambda, {4, 3}];
FTable⟦2, 2⟧ = PaddedForm[Flambda, {5, 3}];
FTable⟦3, 2⟧ = StringJoin[ToString[Round[df1]], ",", ToString[Round[df2]]];
FTable⟦4, 2⟧ = PaddedForm[prob, {5, 3}];
hLegend = Labeled[
  Grid[FTable, BaseStyle → Directive[FontSize → 10, FontFamily → "Arial"],
   Alignment → {{Left, Right}}, Frame → True,
   Dividers → {{True, True}, {True}}], "Wilk's λ Table", Top,
  LabelStyle → Directive[FontSize → 12, Bold, FontFamily → "Arial"]];
plot1 = Plot[Table[PDF[FRatioDistribution[n, df2], x], {n, {df1}}] // Evaluate,
  {x, 0, Flambda}, PlotRange → {{0.0, 50.0}, {0.0, 1.0}},
  Filling → Axis, Exclusions → None, ImageSize → hSizeMVT,
  Filling → Bottom, FillingStyle → Directive[Opacity[1.0], Red],
  AxesLabel → {"F-Ratio", "Prob. Density"},
  LabelStyle → Directive[Black, FontSize → 12, FontFamily → "Arial"]];
probPlot1 = Overlay[{plot1, hLegend}, Alignment → Right];

rmr = eVals⟦1⟧;
d = Max[m2, kg - 1];
df1 = m2;
df2 = n2 - kg - d - 1;
Frmr = (df2 / df1) * rmr;
prob = N[(1 - CDF[FRatioDistribution[df1, df2], Frmr]) * 100];
FTable = Table[" ", {4}, {2}];
FTable⟦1, 1⟧ = "Roy's Maximum Root";
FTable⟦2, 1⟧ = "Observed F-Ratio";
FTable⟦3, 1⟧ = "Degrees of Freedom";
FTable⟦4, 1⟧ = "Probability (%)";
FTable⟦1, 2⟧ = PaddedForm[rmr, {4, 3}];
FTable⟦2, 2⟧ = PaddedForm[Frmr, {5, 3}];
FTable⟦3, 2⟧ = StringJoin[ToString[Round[df1]], ",", ToString[Round[df2]]];
FTable⟦4, 2⟧ = PaddedForm[prob, {5, 3}];
hLegend = Labeled[
  Grid[FTable, BaseStyle → Directive[FontSize → 10, FontFamily → "Arial"],
   Alignment → {{Left, Right}}, Frame → True,
   Dividers → {{True, True}, {True}}], "Roy's Max. Root Table", Top,
```

```
  LabelStyle → Directive[FontSize → 12, Bold, FontFamily → "Arial"]];
plot2 = Plot[Table[PDF[FRatioDistribution[n, df2], x], {n, {df1}}] // Evaluate,
  {x, 0, Frmr}, PlotRange → {{0.0, 50.0}, {0.0, 1.0}},
  Filling → Axis, Exclusions → None, ImageSize → hSizeMVT,
  Filling → Bottom, FillingStyle → Directive[Opacity[1.0], Magenta],
  AxesLabel → {"F-Ratio", "Prob. Density"},
  LabelStyle → Directive[Black, FontSize → 12, FontFamily → "Arial"]];
probPlot2 = Overlay[{plot2, hLegend}, Alignment → Right];

V = 0.0;
Do[V = V + (eVals⟦j⟧ / (1 + eVals⟦j⟧)), {j, noVals}];
s = Min[m2, kg - 1];
d = Max[m2, kg - 1];
df1 = s * d;
df2 = s * (n2 - kg - m2 + s);
FV = ((n2 - kg - m2 + s) * V) / (d (s - V));
prob = N[(1 - CDF[FRatioDistribution[df1, df2], FV]) * 100];
FTable = Table[" ", {4}, {2}];
FTable⟦1, 1⟧ = "Pillai's Trace";
FTable⟦2, 1⟧ = "Observed F-Ratio";
FTable⟦3, 1⟧ = "Degrees of Freedom";
FTable⟦4, 1⟧ = "Probability (%)";
FTable⟦1, 2⟧ = PaddedForm[V, {4, 3}];
FTable⟦2, 2⟧ = PaddedForm[FV, {5, 3}];
FTable⟦3, 2⟧ = StringJoin[ToString[Round[df1]], ",", ToString[Round[df2]]];
FTable⟦4, 2⟧ = PaddedForm[prob, {5, 3}];
hLegend = Labeled[
  Grid[FTable, BaseStyle → Directive[FontSize → 10, FontFamily → "Arial"],
   Alignment → {{Left, Right}}, Frame → True,
   Dividers → {{True, True}, {True}}], "Pillai's Trace Table", Top,
  LabelStyle → Directive[FontSize → 12, Bold, FontFamily → "Arial"]];
plot3 = Plot[Table[PDF[FRatioDistribution[n, df2], x], {n, {df1}}] // Evaluate,
  {x, 0, FV}, PlotRange → {{0.0, 50.0}, {0.0, 1.0}}, Filling → Axis,
  Exclusions → None, ImageSize → hSizeMVT, Filling → Bottom, FillingStyle →
   Directive[Opacity[1.0], Blue], AxesLabel → {"F-Ratio", "Prob. Density"},
  LabelStyle → Directive[Black, FontSize → 12, FontFamily → "Arial"]];
probPlot3 = Overlay[{plot3, hLegend}, Alignment → Right];

U = 0.0;
Do[U = U + eVals⟦j⟧, {j, noVals}];
s = Min[m2, kg - 1];
A = N[(Abs[kg - m2 - 1] - 1) / 2];
B = N[(n2 - kg - m2 - 1) / 2];
df1 = s * ((2 * A) + s + 1);
df2 = 2 * ((s * B) + 1);
FU = (df2 * U) / (s * df1);
prob = N[(1 - CDF[FRatioDistribution[df1, df2], FU]) * 100];
```

```
FTable = Table[" ", {4}, {2}];
FTable[[1, 1]] = "Lawes-Hotelling Trace";
FTable[[2, 1]] = "Observed F-Ratio";
FTable[[3, 1]] = "Degrees of Freedom";
FTable[[4, 1]] = "Probability (%)";
FTable[[1, 2]] = PaddedForm[U, {4, 3}];
FTable[[2, 2]] = PaddedForm[FU, {5, 3}];
FTable[[3, 2]] = StringJoin[ToString[Round[df1]], ",", ToString[Round[df2]]];
FTable[[4, 2]] = PaddedForm[prob, {5, 3}];
hLegend = Labeled[
   Grid[FTable, BaseStyle → Directive[FontSize → 10, FontFamily → "Arial"],
    Alignment → {{Left, Right}}, Frame → True, Dividers → {{True, True}, {True}}],
   "Lawes-Hotelling Trace Table", Top,
   LabelStyle → Directive[FontSize → 12, Bold, FontFamily → "Arial"]];
plot4 = Plot[Table[PDF[FRatioDistribution[n, df2], x], {n, {df1}}] // Evaluate,
   {x, 0, FU}, PlotRange → {{0.0, 50.0}, {0.0, 1.0}}, Filling → Axis,
   Exclusions → None, ImageSize → hSizeMVT, Filling → Bottom, FillingStyle →
    Directive[Opacity[1.0], Green], AxesLabel → {"F-Ratio", "Prob. Density"},
   LabelStyle → Directive[Black, FontSize → 12, FontFamily → "Arial"]];
probPlot4 = Overlay[{plot4, hLegend}, Alignment → Right];

phi = (n2 - 1 - (0.5 * (m2 + nGps))) * (Log[(Det[T] / Det[W])]);
dof = m2 * (nGps - 1);
prob = N[(1 - CDF[ChiSquareDistribution[m2 * (nGps - 1)], phi]) * 100];
FTable = Table[" ", {4}, {2}];
FTable[[1, 1]] = "Log Likelihood Ratio";
FTable[[2, 1]] = "Observed χ² Ratio";
FTable[[3, 1]] = "Degrees of Freedom";
FTable[[4, 1]] = "Probability (%)";
FTable[[1, 2]] = PaddedForm[phi, {4, 3}];
FTable[[2, 2]] = PaddedForm[phi, {4, 3}];
FTable[[3, 2]] = dof;
FTable[[4, 2]] = PaddedForm[prob, {5, 3}];
If[prob ≥ 5.0, rng = phi * 2, rng = phi];
phiLegend = Labeled[
   Grid[FTable, BaseStyle → Directive[FontSize → 10, FontFamily → "Arial"],
    Alignment → {{Left, Right}}, Frame → True,
    Dividers → {{True, True}, {True}}], "Likelihood Ratio Table", Top,
   LabelStyle → Directive[FontSize → 12, Bold, FontFamily → "Arial"]];
plot5 = Plot[PDF[ChiSquareDistribution[dof], x], {x, 0, rng},
   ImageSize → hSizeMVT, Filling → Bottom, FillingStyle → Yellow,
   PlotRange → Full, AxesLabel → {"χ² Ratio", "Prob. Density"},
   LabelStyle → Directive[Black, FontSize → 12, FontFamily → "Arial"]];
probPlot5 = Overlay[{plot5, phiLegend}, Alignment → Right]];

Panel[Labeled[ProgressIndicator[Dynamic[it], {1, iter}], "Calculation Progress",
```

```
    Top, LabelStyle → Directive[FontSize → 12, Bold, FontFamily → "Arial"]]]

  If[statTest > 1,
    avg = Mean[x2];
    stDev = N[StandardDeviation[x2]];
    groupNames = Union[Group];
    numGroups = Length[groupNames];
    groupPosns = Table[Flatten[Position[Group, groupNames⟦i⟧, 1]], {i, kg}];
    x3 = Table[0.0, {n2}, {m2}];
    simRatios = Table[0.0, {iter}, {5}];

    lambda = Det[W] / Det[T];
    df1 = m2 * (kg - 1);
    wCoef = N[n2 - 1 - (m2 + kg) / 2];
    tCoef = N[ Sqrt[(df1^2 - 4) / (m2^2 + (kg - 1)^2 - 5)] ];
    df2 = (wCoef * tCoef) - (df1 / 2) + 1;
    Flambda = N[((1 - lambda^(1/tCoef)) / lambda^(1/tCoef)) * (df2 / df1)];

    rmr = eVals⟦1⟧;
    d = Max[m2, kg - 1];
    df1 = m2;
    df2 = n2 - kg - d - 1;
    Frmr = (df2 / df1) * rmr;

    V = 0.0;
    Do[V = V + (eVals⟦j⟧ / (1 + eVals⟦j⟧)), {j, noVals}];
    s = Min[m2, kg - 1];
    d = Max[m2, kg - 1];
    df1 = s * d;
    df2 = s * (n2 - kg - m2 + s);
    FV = ((n2 - kg - m2 + s) * V) / (d (s - V));

    U = 0.0;
    Do[U = U + eVals⟦j⟧, {j, noVals}];
    s = Min[m2, kg - 1];
    A = N[(Abs[kg - m2 - 1] - 1) / 2];
    B = N[(n2 - kg - m2 - 1) / 2];
    df1 = s * ((2 * A) + s + 1);
    df2 = 2 * ((s * B) + 1);
    FU = (df2 * U) / (s * df1);

    phiTest = (n2 - 1 - (0.5 * (m2 + kg))) * (Log[(Det[T] / Det[W])]);
    chi2Phi = phiTest;

    Do[
     Label[123];
```

```
If[statTest == 2,
 Do[
  Do[
   Do[
    inum = groupPosns⟦k, i⟧;
    x3⟦inum, j⟧ = RandomReal[NormalDistribution[avg⟦j⟧, stDev⟦j⟧]],
    {i, smpSize⟦k⟧}], {j, m2}], {k, kg}]];
If[statTest == 3,
 Do[x3⟦i⟧ = N[x2⟦RandomInteger[{1, n2}]⟧], {i, n2}]];
{gndMean, simT, gNames, nGps, gpMeans, smpSize, simW, simB} =
 TWB[x3, n2, m2, Group];

simWI = PseudoInverse[simW];
simCovar = simWI.simB;
If[n2 > m2, mvecs = m2, mvecs = n2];
If[eMethod == 1,
 simeVals = Eigenvalues[simCovar]];
If[eMethod == 2,
 {w, u, v} = SingularValueDecomposition[simCovar, mvecs];
 simeVals = N[Diagonal[u], kg - 1]];
simNoVals = Min[{nGps - 1, m2}];

simlambda = Det[simW] / Det[simT];
simdf1 = m2 * (kg - 1);
simwCoef = N[n2 - 1 - (m2 + kg) / 2];
simtCoef = N[Sqrt[(simdf1^2 - 4) / (m2^2 + (kg - 1)^2 - 5)]];
simdf2 = (simwCoef * simtCoef) - (simdf1 / 2) + 1;
simFlambda =
 N[((1 - simlambda^(1/simtCoef)) / simlambda^(1/simtCoef)) * (simdf2 / simdf1)];

simrmr = simeVals⟦1⟧;
simd = Max[m2, kg - 1];
simdf1 = m2;
simdf2 = n2 - kg - d - 1;
simFrmr = (simdf2 / simdf1) * simrmr;

simV = 0.0;
Do[simV = simV + (simeVals⟦j⟧ / (1 + simeVals⟦j⟧)), {j, simNoVals}];
sims = Min[m2, kg - 1];
simd = Max[m2, kg - 1];
simdf1 = sims * simd;
simdf2 = s * (n2 - kg - m2 + sims);
simFV = ((n2 - kg - m2 + sims) * simV) / (simd (sims - simV));

simU = 0.0;
Do[simU = simU + simeVals⟦j⟧, {j, simNoVals}];
```

```
 sims = Min[m2, kg - 1];
 simA = N[(Abs[kg - m2 - 1] - 1) / 2];
 simB = N[(n2 - kg - m2 - 1) / 2];
 simdf1 = sims * ((2 * simA) + sims + 1);
 simdf2 = 2 * ((sims * simB) + 1);
 simFU = (simdf2 * simU) / (sims * simdf1);

 simPhi = (n2 - 1 - (0.5 * (m2 + kg))) * (Log[(Det[simT] / Det[simW])]);

 simRatios[[it, 1]] = simFlambda;
 simRatios[[it, 2]] = simFrmr;
 simRatios[[it, 3]] = simFV;
 simRatios[[it, 4]] = simFU;
 simRatios[[it, 5]] = simPhi;
 Do[
  If[Head[simRatios[[it, j]]] == Complex, Goto[123]], {j, 5}], {it, iter}];

simRatiosT = Transpose[simRatios];
Do[simRatiosT[[j]] = Sort[simRatiosT[[j]]], {j, 5}];

simProb = Table[0.0, {5}, {3}];
simProb[[1, 1]] = lambda;
simProb[[2, 1]] = rmr;
simProb[[3, 1]] = V;
simProb[[4, 1]] = U;
simProb[[5, 1]] = phiTest;
simProb[[1, 2]] = Flambda;
simProb[[2, 2]] = Frmr;
simProb[[3, 2]] = FV;
simProb[[4, 2]] = FU;
simProb[[5, 2]] = chi2Phi;
Do[
 knt = 0;
 Do[If[simRatiosT[[j, i]] > simProb[[j, 2]], knt = knt + 1], {i, iter}];
 simProb[[j, 3]] = N[knt / iter] * 100, {j, 5}];
noBins = 40;

FTable = Table[" ", {4}, {2}];
FTable[[1, 1]] = "Wilk's Lambda";
FTable[[2, 1]] = "Observed F-Ratio";
FTable[[3, 1]] = "No. Iterations";
FTable[[4, 1]] = "Probability (%)";
FTable[[1, 2]] = PaddedForm[lambda, {4, 3}];
FTable[[2, 2]] = PaddedForm[Flambda, {5, 3}];
FTable[[3, 2]] = iter;
FTable[[4, 2]] = PaddedForm[simProb[[1, 3]], {5, 3}];
hLegend = Labeled[
```

```
  Grid[FTable, BaseStyle → Directive[FontSize → 10, FontFamily → "Arial"],
   Alignment → {{Left, Right}}, Frame → True,
   Dividers → {{True, True}, {True}}], "Wilk's λ Table", Top,
  LabelStyle → Directive[FontSize → 12, Bold, FontFamily → "Arial"]];
plot1 = Histogram[simRatiosT⟦1⟧, noBins, ChartStyle → {Red},
  ImageSize → hSizeMVT, PlotRange → Automatic,
  LabelStyle → Directive[FontSize → 12, Black, FontFamily → "Arial"],
  AxesLabel → {"F Ratio", "Frequency"}, ImageSize → hSize];
probPlot1 = Overlay[{plot1, hLegend}, Alignment → Right];

FTable = Table[" ", {4}, {2}];
FTable⟦1, 1⟧ = "Roy's Max. Root";
FTable⟦2, 1⟧ = "Observed F-Ratio";
FTable⟦3, 1⟧ = "No. Iterations";
FTable⟦4, 1⟧ = "Probability (%)";
FTable⟦1, 2⟧ = PaddedForm[rmr, {4, 3}];
FTable⟦2, 2⟧ = PaddedForm[Frmr, {5, 3}];
FTable⟦3, 2⟧ = iter;
FTable⟦4, 2⟧ = PaddedForm[simProb⟦2, 3⟧, {5, 3}];
hLegend = Labeled[
  Grid[FTable, BaseStyle → Directive[FontSize → 10, FontFamily → "Arial"],
   Alignment → {{Left, Right}}, Frame → True,
   Dividers → {{True, True}, {True}}], "Roy's Max. Root Table", Top,
  LabelStyle → Directive[FontSize → 12, Bold, FontFamily → "Arial"]];
plot2 = Histogram[simRatiosT⟦2⟧, noBins, ChartStyle → {Magenta},
  ImageSize → hSizeMVT, PlotRange → Automatic,
  LabelStyle → Directive[FontSize → 12, Black, FontFamily → "Arial"],
  AxesLabel → {"F Ratio", "Frequency"}, ImageSize → hSizeMVT];
probPlot2 = Overlay[{plot2, hLegend}, Alignment → Right];

FTable = Table[" ", {4}, {2}];
FTable⟦1, 1⟧ = "Pillai's Trace";
FTable⟦2, 1⟧ = "Observed F-Ratio";
FTable⟦3, 1⟧ = "No. Iterations";
FTable⟦4, 1⟧ = "Probability (%)";
FTable⟦1, 2⟧ = PaddedForm[V, {4, 3}];
FTable⟦2, 2⟧ = PaddedForm[FV, {5, 3}];
FTable⟦3, 2⟧ = iter;
FTable⟦4, 2⟧ = PaddedForm[simProb⟦3, 3⟧, {5, 3}];
hLegend = Labeled[
  Grid[FTable, BaseStyle → Directive[FontSize → 10, FontFamily → "Arial"],
   Alignment → {{Left, Right}}, Frame → True,
   Dividers → {{True, True}, {True}}], "Pillai's Trace Table", Top,
  LabelStyle → Directive[FontSize → 12, Bold, FontFamily → "Arial"]];
plot3 = Histogram[simRatiosT⟦3⟧, noBins, ChartStyle → {Blue},
  ImageSize → hSizeMVT, PlotRange → Automatic,
  LabelStyle → Directive[FontSize → 12, Black, FontFamily → "Arial"],
```

```
    AxesLabel → {"F Ratio", "Frequency"}, ImageSize → hSizeMVT];
  probPlot3 = Overlay[{plot3, hLegend}, Alignment → Right];

  FTable = Table[" ", {4}, {2}];
  FTable[[1, 1]] = "Lawes-Hotelling Trace";
  FTable[[2, 1]] = "Observed F-Ratio";
  FTable[[3, 1]] = "No. Iterations";
  FTable[[4, 1]] = "Probability (%)";
  FTable[[1, 2]] = PaddedForm[U, {4, 3}];
  FTable[[2, 2]] = PaddedForm[FU, {5, 3}];
  FTable[[3, 2]] = iter;
  FTable[[4, 2]] = PaddedForm[simProb[[4, 3]], {5, 3}];
  hLegend = Labeled[
    Grid[FTable, BaseStyle → Directive[FontSize → 10, FontFamily → "Arial"],
     Alignment → {{Left, Right}}, Frame → True, Dividers → {{True, True}, {True}}],
    "Lawes-Hotelling Trace Table", Top,
    LabelStyle → Directive[FontSize → 12, Bold, FontFamily → "Arial"]];
  plot4 = Histogram[simRatiosT[[4]], noBins, ChartStyle → {Green},
    ImageSize → hSizeMVT, PlotRange → Automatic,
    LabelStyle → Directive[FontSize → 12, Black, FontFamily → "Arial"],
    AxesLabel → {"F-Ratio", "Frequency"}, ImageSize → hSizeMVT];
  probPlot4 = Overlay[{plot4, hLegend}, Alignment → Right];

  FTable = Table[" ", {4}, {2}];
  FTable[[1, 1]] = "Log Likelihood Ratio (χ²)";
  FTable[[2, 1]] = "Degrees of Freedom";
  FTable[[3, 1]] = "Iterations";
  FTable[[4, 1]] = "Probability (%)";
  FTable[[1, 2]] = PaddedForm[phiTest, {4, 3}];
  FTable[[2, 2]] = m2 * (kg - 1);
  FTable[[3, 2]] = iter;
  FTable[[4, 2]] = PaddedForm[simProb[[5, 3]], {5, 3}];
  If[simProb[[5, 3]] ≥ 5.0, rng = phiTest * 2, rng = phiTest];
  phiLegend = Labeled[
    Grid[FTable, BaseStyle → Directive[FontSize → 10, FontFamily → "Arial"],
     Alignment → {{Left, Right}}, Frame → True,
     Dividers → {{True, True}, {True}}], "Likelihood Ratio Table", Top,
    LabelStyle → Directive[FontSize → 12, Bold, FontFamily → "Arial"]];
  plot5 = Histogram[simRatiosT[[5]], noBins, ChartStyle → {Yellow},
    ImageSize → hSizeMVT, PlotRange → Automatic,
    LabelStyle → Directive[FontSize → 12, Black, FontFamily → "Arial"],
    AxesLabel → {"χ² Ratio", "Frequency"}, ImageSize → hSizeMVT];
  probPlot5 = Overlay[{plot5, phiLegend}, Alignment → Right]];

label1 = "Parametric Probability Results";
label2 = "Monte Carlo Simulation Results";
```

```
label3 = "Bootstrap Modelling Results";
probGrid = Labeled[
  GraphicsGrid[{{probPlot1, probPlot2}, {probPlot3, probPlot4}, {probPlot5}},
   ImageSize → 700, AspectRatio → 1 / 0.80],
  ToExpression[StringJoin["label", ToString[statTest]]], Top,
  LabelStyle → Directive[FontSize → 18, Bold, FontFamily → "Arial"]]
```

Out[∘]= **Calculation Progress**

Export mean vector test results

```
In[∘]:= filenameout = SystemDialogInput["FileSave"];
        Export[filenameout, probGrid, "TIFF", ImageResolution → 150]
```

Out[∘]= /Users/n.macleod/Documents/Iris Results/Mean Vectopr Tests (BS).tif

Export list of simulated mean vector test index values

```
simRatiosOut = Table[" ", {iter + 1}, {5}];
simRatiosOut⟦1, 1⟧ = "Wilk's λ";
simRatiosOut⟦1, 2⟧ = "Roys Maximum Root";
simRatiosOut⟦1, 3⟧ = "Pillai's Trace";
simRatiosOut⟦1, 4⟧ = "Laws-Hotelling Trace";
simRatiosOut⟦1, 5⟧ = "Likelihood Ratio";
Do[simRatiosOut⟦i + 1, j⟧ = simRatiosT⟦j, i⟧, {i, iter}, {j, 5}]

filenameout = SystemDialogInput["FileSave"];
Export[filenameout, simRatiosOut, "CSV", "TextDelimiters" → ""]
```

/Users/nm/Desktop/Aubergine
  Dataset/CVA Results/Mean Vector Test Results (BS).csv

Export CVA results.

Export canonical variate eigenvalues.

```
In[∘]:= filenameout = SystemDialogInput["FileSave"];
        Export[filenameout, t2, "CSV", "TextDelimiters" → ""]
```

Out[∘]= /Users/n.macleod/Documents/Iris Results/Eigenvalues.csv

Export canonical variate loadings.

In[∘]:=
```
filenameout = SystemDialogInput["FileSave"];
Export[filenameout, eVecs, "CSV", "TextDelimiters" → ""]
```

Out[∘]=
```
/Users/n.macleod/Documents/Iris Results/Eigenvectors.csv
```

Export canonical variate scores.

In[∘]:=
```
outScores = Table[" ", {n2 + 1}, {noVals + 2}];
outScores[[1, 1]] = "Object";
outScores[[1, 2]] = "Group";
Do[outScores[[1, j + 2]] = StringJoin["CV-", ToString[j]], {j, noVals}]
Do[outScores[[i + 1, 1]] = objNames[[i]], {i, n2}]
Do[outScores[[i + 1, 2]] = Group[[i]], {i, n2}]
Do[outScores[[i + 1, j + 2]] = eScores[[i, j]], {i, n2}, {j, noVals}]

filenameout = SystemDialogInput["FileSave"];
Export[filenameout, outScores, "CSV", "TextDelimiters" → ""]
```

Out[∘]=
```
/Users/n.macleod/Desktop/Bumpus Sparrow Results/Eigenscores.csv
```

Calculate discriminant performance measures.

Calculate distance table.

In[∘]:=
```
gpCVMeans = Table[0.0, {nGps}, {noVals}];
mDist = Table[0.0, {n2}, {nGps}];
distTable = Table[0.0, {n2 + 1}, {nGps + 2}];
distTableOut = Table[0.0, {n2 + 1}, {nGps + 2}];
eScrs = Take[eScores, All, noVals];

Do[
 Do[
   sum = 0.0;
   Do[
    If[Group[[i]] == gNames[[k]],
     sum = sum + eScores[[i, j]],
     sum = sum],
    {i, n2}];
   gpCVMeans[[k, j]] = sum / smpSize[[k]],
   {j, noVals}];,
 {k, nGps}]
gpCVMeans;

Do[
 Do[
  mDist[[i, k]] = EuclideanDistance[gpCVMeans[[k]], eScrs[[i]]],
```

```
   {i, n2}],
  {k, nGps}]
mDist;

pDist = mDist;
Do[
  n = 0;
  ref = Min[pDist⟦k⟧];
  Do[
   If[pDist⟦k, j⟧ == ref, pDist⟦k, j⟧ = Style[pDist⟦k, j⟧, Bold]],
   {j, nGps}],
  {k, n2}]

distTable⟦1, 1⟧ = "Object";
distTableOut⟦1, 1⟧ = "Object";
distTable⟦1, 2⟧ = "Group";
distTableOut⟦1, 2⟧ = "Group";
Do[distTable⟦1, j + 2⟧ = Text[Rotate[gNames⟦j⟧, 90 Degree]], {j, nGps}]
Do[distTableOut⟦1, j + 2⟧ = gNames⟦j⟧, {j, nGps}]
Do[
  distTable⟦i + 1, 1⟧ = objNames⟦i⟧;
  distTableOut⟦i + 1, 1⟧ = objNames⟦i⟧;
  distTable⟦i + 1, 2⟧ = Group⟦i⟧;
  distTableOut⟦i + 1, 2⟧ = Group⟦i⟧;
  Do[distTable⟦i + 1, j + 2⟧ = PaddedForm[pDist⟦i, j⟧, {4, 3}], {j, nGps}];
  Do[distTableOut⟦i + 1, j + 2⟧ = mDist⟦i, j⟧, {j, nGps}],
  {i, n2}]

distances = Labeled[Grid[distTable, BaseStyle → (FontFamily → "Arial"),
    Alignment → {{Left, Center, Center, Center, Center, Center, Center, Center},
      {Bottom, Baseline}}, Frame → True,
    Dividers → {{True, True, True}, {True, True}}], "Distance Table", Top,
   LabelStyle → Directive[Black, Bold, FontSize → 14, FontFamily → "Arial"]]
```

Export group distance table.

```
In[•]:= filenameout = SystemDialogInput["FileSave"];
        Export[filenameout, distTableOut, "CSV", "TextDelimiters" → ""]
Out[•]= /Users/n.macleod/Desktop/Bumpus Sparrow Results/Distance Table (Raw).csv
```

Calculate confusion matrix.

```
In[•]:= cMat = Table[0, {nGps}, {nGps}];
        gPos = Table[Flatten[Position[Group, gNames⟦i⟧, 1]], {i, nGps}];
        pDist = mDist;

        Do[
```

```
 Do[
  Do[
   gPos⟦k, ig⟧;
   pDist⟦gPos⟦k, ig⟧⟧;
   ref = Min[pDist⟦gPos⟦k, ig⟧⟧];
   pDist⟦gPos⟦k, ig⟧, j⟧;
   If[pDist⟦gPos⟦k, ig⟧, j⟧ == ref,
    cMat⟦k, j⟧ = cMat⟦k, j⟧ + 1, cMat⟦k, j⟧ = cMat⟦k, j⟧],
   {j, nGps}],
  {ig, smpSize⟦k⟧}],
 {k, nGps}]
cMat;

c = Tr[cMat];
s = Total[cMat, nGps];
num = c * s;
Do[
  num = num - (Total[Take[cMat⟦i, All⟧]] * Total[Take[cMat⟦All, i⟧]]), {i, nGps}];
d1 = s^2;
Do[d1 = d1 - Total[Take[cMat⟦i, All⟧]]^2, {i, nGps}];
d2 = s^2;
Do[d2 = d2 - Total[Take[cMat⟦All, i⟧]]^2, {i, nGps}];
denom = N[√(d1 * d2)];
mcc = num / denom;

cmTable = Table[" ", {nGps + 5}, {nGps + 4}];
cmOut = Table[" ", {nGps + 5}, {nGps + 4}];
cmTable⟦1, 1⟧ = "Groups";
cmOut⟦1, 1⟧ = "Groups";
Do[cmTable⟦1, j + 1⟧ = Text[Rotate[gNames⟦j⟧, 90 Degree]], {j, nGps}];
Do[cmOut⟦1, j + 1⟧ = gNames⟦j⟧, {j, nGps}];
Do[cmTable⟦j + 1, 1⟧ = gNames⟦j⟧, {j, nGps}];
Do[cmOut⟦j + 1, 1⟧ = gNames⟦j⟧, {j, nGps}];
cmTable⟦nGps + 2, 1⟧ = "Total Correct";
cmOut⟦nGps + 2, 1⟧ = "Total Correct";
cmTable⟦nGps + 3, 1⟧ = "Total Estimated";
cmOut⟦nGps + 3, 1⟧ = "Total Estimated";
cmTable⟦nGps + 4, 1⟧ = "Percent Estimated Correctly";
cmOut⟦nGps + 4, 1⟧ = "Percent Estimated Correctly";
cmTable⟦nGps + 5, 1⟧ = "Matthew's Correlation Coef.";
cmOut⟦nGps + 5, 1⟧ = "Matthew's Correlation Coef.";
cmTable⟦1, nGps + 3⟧ = Text[Rotate["Group Totals", 90 Degree]];
cmOut⟦1, nGps + 3⟧ = "Group Totals";
cmTable⟦1, nGps + 2⟧ = Text[Rotate["Total Correct", 90 Degree]];
cmOut⟦1, nGps + 2⟧ = "Total Correct";
cmTable⟦1, nGps + 4⟧ = Text[Rotate["Percent Correct", 90 Degree]];
```

```
cmOut⟦1, nGps + 4⟧ = "Percent Correct";
Do[cmTable⟦i + 1, j + 1⟧ = cMat⟦i, j⟧, {i, nGps}, {j, nGps}];
Do[cmOut⟦i + 1, j + 1⟧ = cMat⟦i, j⟧, {i, nGps}, {j, nGps}];
cTot = Total[cMat];
rTot = Diagonal[cMat];
gpTot = Total[cMat, {2}];
gTot = Total[cTot];

Do[cmTable⟦i + 1, nGps + 2⟧ = rTot⟦i⟧, {i, nGps}];
Do[cmOut⟦i + 1, nGps + 2⟧ = rTot⟦i⟧, {i, nGps}];
Do[cmTable⟦i + 1, nGps + 3⟧ = gpTot⟦i⟧, {i, nGps}];
Do[cmOut⟦i + 1, nGps + 3⟧ = gpTot⟦i⟧, {i, nGps}];
Do[cmTable⟦i + 1, nGps + 4⟧ =
   PaddedForm[N[(rTot⟦i⟧ / gpTot⟦i⟧) * 100.0], {4, 2}], {i, nGps}];
Do[cmOut⟦i + 1, nGps + 4⟧ = N[(rTot⟦i⟧ / gpTot⟦i⟧) * 100.0], {i, nGps}];
cmTable⟦nGps + 2, nGps + 3⟧ = gTot;
cmOut⟦nGps + 2, nGps + 3⟧ = gTot;
cmTable⟦nGps + 2, nGps + 2⟧ = Total[rTot];
cmOut⟦nGps + 2, nGps + 2⟧ = Total[rTot];
cmTable⟦nGps + 2, nGps + 4⟧ = PaddedForm[N[(Total[rTot] / gTot) * 100.0], {4, 2}];
cmOut⟦nGps + 2, nGps + 4⟧ = N[(Total[rTot] / gTot) * 100.0];

Do[cmTable⟦nGps + 2, i + 1⟧ = rTot⟦i⟧, {i, nGps}]
Do[cmOut⟦nGps + 2, i + 1⟧ = rTot⟦i⟧, {i, nGps}]
Do[cmTable⟦nGps + 3, i + 1⟧ = cTot⟦i⟧, {i, nGps}]
Do[cmOut⟦nGps + 3, i + 1⟧ = cTot⟦i⟧, {i, nGps}]
Do[cmTable⟦nGps + 4, i + 1⟧ =
   PaddedForm[N[(rTot⟦i⟧ / cTot⟦i⟧) * 100.0], {4, 2}], {i, nGps}];
Do[cmOut⟦nGps + 4, i + 1⟧ = N[(rTot⟦i⟧ / cTot⟦i⟧) * 100.0], {i, nGps}];
cmTable⟦nGps + 3, nGps + 2⟧ = Total[cTot];
cmOut⟦nGps + 3, nGps + 2⟧ = Total[cTot];
cmTable⟦nGps + 4, nGps + 2⟧ =
  PaddedForm[N[(Total[rTot] / Total[cTot]) * 100.0], {4, 2}];
cmOut⟦nGps + 4, nGps + 2⟧ = N[(Total[rTot] / Total[cTot]) * 100.0];
cmTable⟦nGps + 5, 2⟧ = PaddedForm[mcc, {4, 3}];
cmOut⟦nGps + 5, 2⟧ = mcc;

confusion = Labeled[Grid[cmTable, Frame → True,
   Dividers → {{2 → True, -4 → True}, {2 → True, -2 → True, -5 → True}},
   BaseStyle → (FontFamily → "Arial"),
   Alignment → {{Right, Center}, {Bottom, Baseline}}], "Raw Confusion Matrix",
  Top, LabelStyle → Directive[FontSize → 14, Bold, FontFamily → "Arial"]]
```

Export confusion matrix table.

In[∘]:=
```
filenameout = SystemDialogInput["FileSave"];
Export[filenameout, cmOut, "CSV", "TextDelimiters" → ""]
```

Out[∘]= /Users/n.macleod/Desktop/Bumpus Sparrow Results/Confusion Matrix (Raw).csv

Modelling Section (optional).

Calculate along – axis models.

Specify axis modelling options.

In[∘]:=
```
Panel[Labeled[Row[{Panel[Labeled[InputField[Dynamic[modelAxes], FieldSize → 5],
      "Enter no. of axes to be modeled.", Top,
      LabelStyle → Directive[FontSize → 12, FontFamily → "Arial"]]], , ,
    Panel[Labeled[InputField[Dynamic[modelsPerAxis], FieldSize → 5],
      "Enter no. models to be plotted per axis.", Top,
      LabelStyle → Directive[FontSize → 12, FontFamily → "Arial"]]]}],
  "Along-Axis Modeling Parameters", Top, LabelStyle →
   Directive[FontSize → 16, Bold, FontFamily → "Arial"]]]
modelsPerAxis = 5; modelAxes = noAxes;
```

Out[∘]=

Along–Axis Modeling Parameters

Enter no. of axes to be modeled.	Enter no. models to be plotted per axis.
modelAxes	modelsPerAxis

Perform axis model location calculations.

In[•]:=
```
modSet = 1;
models = Table[0.0, {modelsPerAxis}, {modelAxes}];
modTable = Table[0.0, {modelAxes}, {modelsPerAxis}, {m2}];

scrMeans = Mean[eScores];

eScoresT = Transpose[eScores];
Do[
  xMax = Max[eScoresT[[k]]];
  xMin = Min[eScoresT[[k]]];
  xRng = xMax - xMin;
  xInt = xRng / (modelsPerAxis - 1);
  xBase = xMin - xInt;
  Do[models[[i, k]] = xBase + (xInt * i), {i, modelsPerAxis}];
  Do[modTable[[k, i, k]] = xBase + (xInt * i), {i, modelsPerAxis}], {k, modelAxes}];
modTable = Partition[Flatten[Chop[modTable]], m2];

If[meanTrans == 1,
 Do[
  If[modTable[[i, j]] == 0,
   modTable[[i, j]] = scrMeans[[j]]], {i, modelsPerAxis * modelAxes}, {j, m2}]]
```

Calculate group-difference models.

Specify group – difference modelling options.

In[•]:=
```
Panel[Labeled[Panel[Labeled[InputField[Dynamic[modelsPerAxis], FieldSize → 5],
    "Enter no. models to be plotted per axis.", Top,
    LabelStyle → Directive[FontSize → 12, FontFamily → "Arial"]]],
  "Group-Difference Modeling Parameters", Top,
  LabelStyle → Directive[FontSize → 16, Bold, FontFamily → "Arial"]]]
modelsPerAxis = 5;
```

Out[•]=

Group–Difference Modeling Parameters

Enter no. models to be plotted per axis.

models⋱ PerAx⋱ is

Perform group – difference model location calculations.

In[ ]:=
```
modSet = 2;
groupNames = Union[Group];
numGroups = Length[groupNames];
numCV = numGroups - 1;
groupPosns = Table[Flatten[Position[Group, groupNames[[i]], 1]], {i, numGroups}];
cVariates = Take[eScores, All, numCV];

meanCV = Table[0.0, {numGroups}, {numCV}];
Do[
  ng = Length[groupPosns[[k]]];
  gp = Table[0.0, {ng}, {numCV}];
  Do[gp[[i, j]] = cVariates[[groupPosns[[k, i]], j]], {i, ng}, {j, numCV}];
  meanCV[[k]] = Mean[gp], {k, numGroups}];

meanCV2 = Append[meanCV, meanCV[[1]]];
models = Table[0.0, {numGroups}, {modelsPerAxis}, {numCV}];
modTable = Table[0.0, {numGroups * modelsPerAxis}, {m2}];
Do[
  dif = meanCV2[[k + 1]] - meanCV2[[k]];
  dif = dif / (modelsPerAxis - 1);
  models[[k, 1]] = meanCV[[k]];
  Do[models[[k, i + 1]] = models[[k, 1]] + (dif * i), {i, modelsPerAxis - 1}],
  {k, numGroups}];
models = Partition[Flatten[models], numGroups - 1];
Do[modTable[[i, j]] = models[[i, j]], {i, numGroups * modelsPerAxis}, {j, numCV}];
```

Calculate planar subspace models

Specify subspace modelling options.

In[•]:=
```
Panel[
 Labeled[Column[{Row[{Panel[Labeled[PopupMenu[Dynamic[xAxisName], cvNames],
        "Select canonical variate to be modeled on x-axis.", Top,
        LabelStyle → Directive[FontSize → 12, FontFamily → "Arial"]]], , ,
      Panel[Labeled[InputField[Dynamic[xModelNum], FieldSize → 5],
        "Enter no. of models along the x-axis.", Top,
        LabelStyle → Directive[FontSize → 12, FontFamily → "Arial"]]]}],
    Row[{Panel[Labeled[PopupMenu[Dynamic[yAxisName], cvNames],
        "Select canonical variate to be modeled on y-axis.", Top,
        LabelStyle → Directive[FontSize → 12, FontFamily → "Arial"]]], , ,
      Panel[Labeled[InputField[Dynamic[yModelNum], FieldSize → 5],
        "Enter no. of models along the y-axis.", Top,
        LabelStyle → Directive[FontSize → 12, FontFamily → "Arial"]]]}]}],
  "Subspace Modeling Parameters", Top, LabelStyle →
   Directive[FontSize → 18, Bold, FontFamily → "Arial"]]]
xAxisName = cvNames〚1〛;
yAxisName = cvNames〚2〛;
xModelNum = 5;
yModelNum = 4;
```

Out[•]=

Subspace Modeling Parameters

Select canonical variate to be modeled on x–axis.
PopupMenu[CV–1, Table[CV– <> ToString[i], {i, noAxes}]]

Enter no. of models along the x–axis.
xModel⋱ Num

Select canonical variate to be modeled on y–axis.
PopupMenu[CV–1, Table[CV– <> ToString[i], {i, noAxes}]]

Enter no. of models along the y–axis.
yModel⋱ Num

Perform subspace model location calculations

```
In[•]:= modSet = 3;
models = Table[0.0, {yModelNum}, {xModelNum}, {2}];
eScoresT = Transpose[eScores];
Do[If[xAxisName == cvNames[[j]], axis1 = j], {j, noAxes}]
Do[If[yAxisName == cvNames[[j]], axis2 = j], {j, noAxes}]
xMax = Max[eScoresT[[axis1]]];
xMin = Min[eScoresT[[axis1]]];
xRng = xMax - xMin;
xInt = xRng / (xModelNum - 1);
yMax = Max[eScoresT[[axis2]]];
yMin = Min[eScoresT[[axis2]]];
yRng = yMax - yMin;
yInt = yRng / (yModelNum - 1);

xBase = xMin - xInt;
yBase = yMin - yInt;
Do[
 yVal = yBase + (yInt * j);
 Do[
  models[[j, i, 1]] = xBase + (xInt * i);
  models[[j, i, 2]] = yVal, {i, xModelNum}], {j, yModelNum}]
models = Partition[Flatten[Reverse[models]], 2];

nrows = xModelNum * yModelNum;
modTable = Table[0.0, {nrows}, {m2}];
Do[
  modTable[[i, axis1]] = mTable[[i, 1]];
  modTable[[i, axis2]] = mTable[[i, 2]], {i, nrows}];
```

Import external set of model coordinates
(total must match along – axis model specifications [above] and contain the complete variable set) .

```
filenamein = SystemDialogInput["FileOpen"];
modTable = Import[filenamein, "CSV"];
```

Plot model coordinates in the CV space.

Specify 2 D plot options.
You must run this code after you read in the data so it can pick up the proper variable names.

```
In[•]:= cvNames = Table[StringJoin["CV-", ToString[i]], {i, noAxes}];
Panel[Labeled[Column[
   {Panel[Labeled[RadioButtonBar[Dynamic[modSet], {1 → "Along-axis Models",
         2 → "Group-Difference Models", 3 → "Subspace Models"}],
      "Enter type of model set.", Top, LabelStyle →
       Directive[FontSize → 12, Bold, FontFamily → "Arial"]]],
    Row[{Panel[Labeled[PopupMenu[Dynamic[xAxisName], cvNames],
         "Select variable to be plotted on x-axis.", Top, LabelStyle →
          Directive[FontSize → 12, Bold, FontFamily → "Arial"]]], "  ",
      Panel[Labeled[PopupMenu[Dynamic[yAxisName], cvNames],
        "Select variable to be plotted on y-axis.", Top,
        LabelStyle → Directive[FontSize → 12, Bold, FontFamily → "Arial"]]]}],
    Row[{
      Panel[Labeled[PopupMenu[Dynamic[pltAspect],
          {1 → "Golden Ratio Plot", 2 → "Square Plot (equi-length axes)",
           3 → "True-Scale Plot (actual axis scales)"}],
        "Enter plot aspect ratio type.", Top, LabelStyle →
         Directive[FontSize → 12, Bold, FontFamily → "Arial"]]], "  ",
      Panel[Labeled[PopupMenu[Dynamic[lch], {1 → "Simple scatterplot",
           2 → "Scatterplot w/ convex hulls"}],
        "Show group domians?", Top, LabelStyle →
         Directive[FontSize → 12, Bold, FontFamily → "Arial"]]], "  ",
      Panel[Labeled[PopupMenu[Dynamic[ptsJoin], {1 → "No", 2 → "Yes"}],
        "Join datapoints?", Top,
        LabelStyle → Directive[FontSize → 12, Bold, FontFamily → "Arial"]]]}],
    Row[{Panel[Labeled[InputField[Dynamic[pltSz], FieldSize → 5],
         "Enter plot size value.", Top, LabelStyle →
          Directive[FontSize → 12, Bold, FontFamily → "Arial"]]], "  ",
      Panel[Labeled[InputField[Dynamic[iconSizeMods], FieldSize → 5],
        "Enter plot icon size value.", Top, LabelStyle →
         Directive[FontSize → 12, Bold, FontFamily → "Arial"]]], "  ",
      Panel[Labeled[InputField[Dynamic[pltPad], FieldSize → 5],
        "Enter plot margin padding value.", Top,
        LabelStyle → Directive[FontSize → 12, Bold, FontFamily → "Arial"]]]}]},
   Center], "Model Coordinate Options", Top, LabelStyle →
   Directive[FontSize → 18, Bold, FontFamily → "Arial"]]]
pltSize = 500; iconSizeMods = 0.02; pltPad = 0.1;
xAxisName = cvNames⟦1⟧;
pltSz = 500; yAxisName = cvNames⟦2⟧; ptsJoin = 1;
dataTrans = 1; pltAspect = 1 ;
lch = 2;
```

Out[∘]=

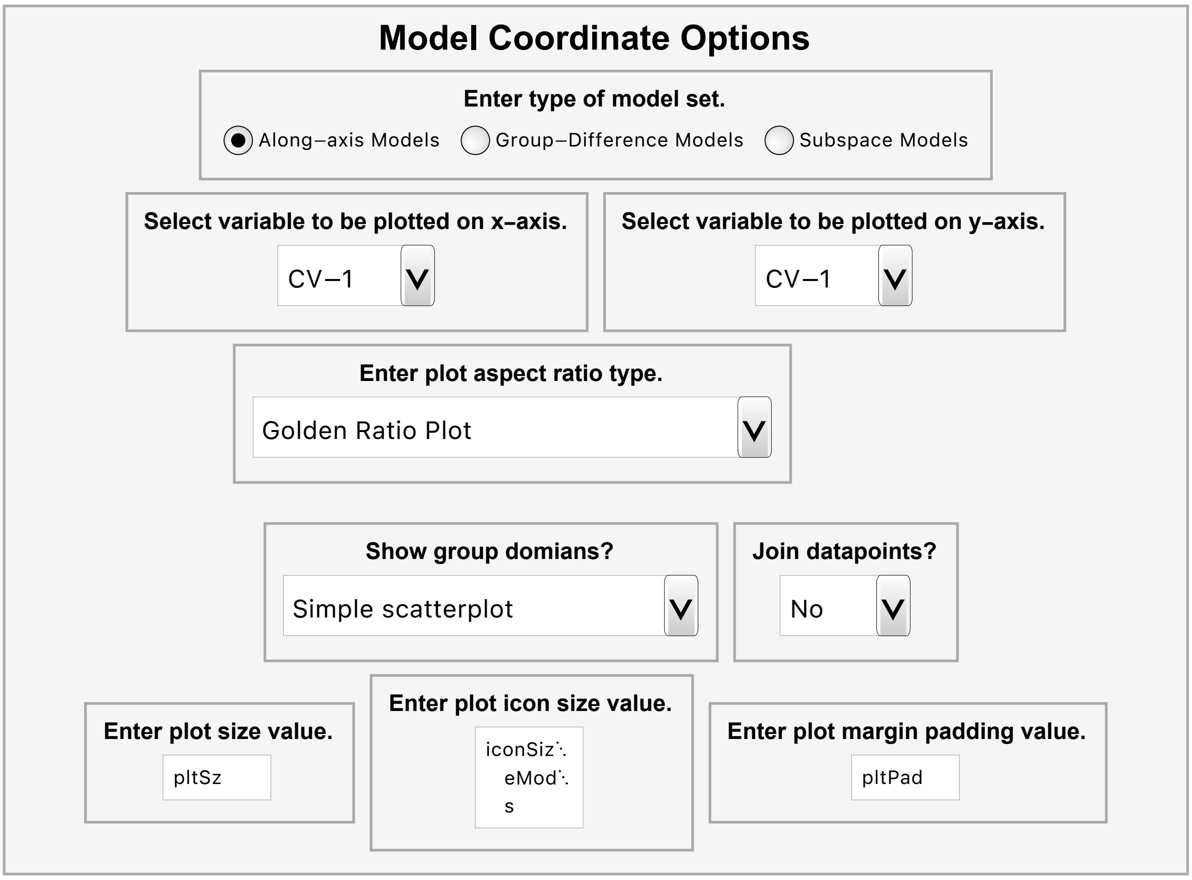

Plot model coordinates in space of a CVA plane (optional).

In[∘]:=

```
axisPlots = Table[" ", {2}];
Do[If[xAxisName == cvNames[[j]], axis1 = j], {j, noAxes}]
Do[If[yAxisName == cvNames[[j]], axis2 = j], {j, noAxes}]

If[modSet == 1,
  nrows = modelsPerAxis;
  modpts = Table[0.0, {2}, {nrows}, {2}];
  Do[modpts[[1, i, j]] = scrMeans[[axis2]], {i, nrows}, {j, 2}];
  Do[modpts[[2, i, j]] = scrMeans[[axis1]], {i, nrows}, {j, 2}];
  Do[modpts[[1, i, 1]] = models[[i, axis1]], {i, nrows}];
  Do[modpts[[2, i, 2]] = models[[i, axis2]], {i, nrows}];
 ];

If[modSet == 2,
  nrows = numGroups * modelsPerAxis;
  modpts = models];

If[modSet == 3,
  nrows = numGroups * modelsPerAxis;
  modpts = models];

groupNames = Union[Group];
```

```
numGroups = Length[groupNames];
numCV = numGroups - 1;
groupPosns = Table[Flatten[Position[Group, groupNames⟦i⟧, 1]], {i, numGroups}];
eScoresT = Transpose[eScores];

xAxis = eScoresT⟦axis1⟧; yAxis = eScoresT⟦axis2⟧;
lab1 = StringJoin[{"Canonical Variate ", ToString[axis1]},
   {" (Var. =", ToString[t3⟦axis1 + 1, 3⟧]}, {"%)"}];
lab2 = StringJoin[{"Canonical Variate ", ToString[axis2]},
   {" (Var. =", ToString[t3⟦axis2 + 1, 3⟧]}, {"%)"}];
maxx =
 Max[
  xAxis];
minx = Min[xAxis];
maxy = Max[yAxis];
miny = Min[yAxis];

If[pltAspect == 1 || pltAspect == 3,
  xPlotLow = minx; xPlotHi = maxx; yPlotLow = miny; yPlotHi = maxy];
If[pltAspect == 2,
  If[minx > miny,
   xPlotLow = miny; yPlotLow = miny,
   xPlotLow = minx; yPlotLow = minx ]];
If[pltAspect == 2,
  If[maxx < maxy,
   xPlotHi = maxy; yPlotHi = maxy,
   xPlotHi = maxx; yPlotHi = maxx]];
If[pltAspect == 1, aRatio = 1 / N[GoldenRatio]];
If[pltAspect == 2, aRatio = 1];
If[pltAspect == 3, aRatio = Automatic];

tmpPoints = Transpose[List[xAxis, yAxis]];
pltPoints = Table[tmpPoints⟦groupPosns⟦j⟧⟧, {j, numGroups}];
iconList = Flatten[Table[
    {Graphics[{EdgeForm[{Thickness[0.005], Black}],
       Hue[N[((numGroups + 1) - j) / numGroups]],
       Disk[{0, 0}, Scaled[iconSizeMods]]}]}, {j, numGroups}]];

If[lch == 1 || ptsJoin == 1,
  Do[
   pltTable⟦k, 1⟧ = pTmp =
     ListPlot[pltPoints⟦k⟧, AspectRatio → aRatio, Frame → True, Joined → False,
      Axes → False, PlotRange → {{xPlotLow, xPlotHi}, {yPlotLow, yPlotHi}},
      PlotRangePadding → Scaled[pltPad], Ticks → Automatic, FrameLabel →
       {lab1, lab2}, PlotMarkers → iconList⟦k⟧, ImageSize → pltSize, LabelStyle →
       Directive[FontSize → 14, Black, FontFamily → "Arial"]], {k, kg}],
  Do[
```

```
    pltTable⟦k, 1⟧ =
     ListPlot[pltPoints⟦k⟧, Frame → True, Axes → False, AspectRatio → aRatio,
      PlotRange → {{xPlotLow, xPlotHi}, {yPlotLow, yPlotHi}}, PlotRangePadding →
       Scaled[pltPad], Ticks → Automatic, FrameLabel → {lab1, lab2},
      LabelStyle → Directive[Black, FontSize → 14, FontFamily → "Arial"],
      ImageSize → pltSize, PlotStyle → Directive[Disk[],
        Hue[N[((+1) - k) / kg]], EdgeForm[{Thickness[1.0], Black}],
        PointSize[Scaled[iconSize - 0.009]]]], {k, kg}]];

If[ptsJoin == 2,
  Do[
   pltTable⟦k, 2⟧ =
    ListLinePlot[pltPoints⟦k⟧, AspectRatio → aRatio, Frame → True, Joined → True,
     Axes → False, PlotStyle → Directive[Hue[N[((+1) - k) / kg]], Thin],
     PlotRange → {{xPlotLow, xPlotHi}, {yPlotLow, yPlotHi}},
     PlotRangePadding → Scaled[pltPad], Ticks → Automatic,
     FrameLabel → {lab1, lab2}, ImageSize → pltSize, LabelStyle →
      Directive[FontSize → 14, Black, FontFamily → "Arial"]], {k, kg}]];

If[lch == 2,
  Do[
   hull = ConvexHullMesh[pltPoints⟦k⟧];
   pltTable⟦k, 3⟧ = HighlightMesh[hull,
     Style[2, Opacity[0.2], Hue[N[((+1) - k) / kg]]], Frame → True, Axes → False,
     AspectRatio → aRatio, PlotRange → {{xPlotLow, xPlotHi}, {yPlotLow, yPlotHi}},
     PlotRangePadding → Scaled[pltPad],
     Ticks → Automatic, FrameLabel → {lab1, lab2},
     LabelStyle → Directive[Black, FontSize → 14, FontFamily → "Arial"],
     ImageSize → pltSize], {k, kg}]];

If[ptsJoin == 1 && lch == 1, p0 = Show[pltTable⟦All, 1⟧]];
If[ptsJoin == 2 && lch == 1, p0 = Show[pltTable⟦All, 2⟧, pltTable⟦All, 1⟧]];
If[ptsJoin == 1 && lch == 2, p0 = Show[pltTable⟦All, 3⟧, pltTable⟦All, 1⟧]];
If[ptsJoin == 2 && lch == 2,
  p0 = Show[pltTable⟦All, 3⟧, pltTable⟦All, 2⟧, pltTable⟦All, 1⟧]];

iconList = Flatten[Table[
    {Graphics[{EdgeForm[{Thin, Black}],
       Black, Disk[{0, 0}, Scaled[iconSizeMods / 2]]}]}, {j, 1}]];

If[modSet == 1,
  Do[
   axisPlots⟦i⟧ = ListPlot[modpts⟦i⟧, AspectRatio → aRatio,
     Frame → True, Joined → True, Axes → False, PlotRange → All,
     PlotStyle → Directive[Black, AbsoluteThickness[0.8]],
     PlotRangePadding → Scaled[pltPad], Ticks → Automatic,
     FrameLabel → {lab1, lab2}, PlotMarkers → iconList], {i, 2}];
```

```
    p4 = Show[axisPlots]];
  If[modSet == 2,
    p4 = ListPlot[modpts, AspectRatio → aRatio,
      Frame → True, Joined → True, Axes → False, PlotRange → All,
      PlotStyle → Directive[Black, AbsoluteThickness[0.8]],
      PlotRangePadding → Scaled[pltPad], Ticks → Automatic,
      FrameLabel → {lab1, lab2}, PlotMarkers → iconList]];
  If[modSet == 3,
    subSpacePlot = ListPlot[modpts, AspectRatio → aRatio,
      Frame → True, Joined → False, Axes → False, PlotRange → All,
      PlotStyle → Directive[Black, AbsoluteThickness[0.8]],
      PlotRangePadding → Scaled[pltPad], Ticks → Automatic,
      FrameLabel → {lab1, lab2}, PlotMarkers → iconList];
    p4 = subSpacePlot];
  p1 = Labeled[Show[p0, p4], "          CV Score Plot", Top,
     LabelStyle → Directive[FontSize → 18, Bold, FontFamily → "Arial"]];
  g1 = Grid[Table[
      {Graphics[{EdgeForm[{Thin, Black}], Hue[N[((numGroups + 1) - j) / numGroups]],
         Disk[]}, ImageSize → 13]}, {j, numGroups}], Frame → False];
  g2 = Grid[Partition[groupNames, 1], Alignment → Left,
     BaseStyle → {FontFamily → "Arial", FontSize → 13, Italic}];
  p2 = Labeled[Text[Grid[{{g1, g2}}, Alignment → Bottom, Frame → True]], "Legend",
     Top, LabelStyle → Directive[Black, FontSize → 18, Bold, FontFamily → "Arial"]];

  plt2D = Grid[{{p1, p2}}, BaselinePosition → Top, Alignment → Top]
```

Export plot (optional).

```
In[•]:= filenameout = SystemDialogInput["FileSave"];
        Export[filenameout, plt2D, "TIFF", ImageResolution → 150]

Out[•]= /Users/n.macleod/Documents/Iris Results/CV-1 vs CV-2 (w: Model Coords).tif
```

Export model coordinates in the CV space.

```
In[•]:= If[modSet ≤ 2, partNum = noAxes, partNum = 2];
        modelsOut = Take[Partition[Flatten[modTable], m2], All, partNum];
        filenameout = SystemDialogInput["FileSave"];
        Export[filenameout, modelsOut, "CSV", "TextDelimiters" → ""]

Out[•]= /Users/n.macleod/Desktop/Dragonflies (Final)/Data & Results/Images
          (PCA-CVA)/Hindwings/CVA Results/Water Body Groups/CVA Model Coords.csv
```

Back – project the model coordinates into the space of the orginal variables.

In[•]:=
```
eVecsInv = Inverse[eVecsTotal];
modMatrix = modTable;
modCoords = modMatrix.eVecsInv;
```

Plot model coordinates in the space of the original variables (optional).

Specify 2 D plot options.
You must run this code after you read in the data so it can pick up the proper variable names.

```
In[•]:= Panel[Labeled[Column[
    {Panel[Labeled[RadioButtonBar[Dynamic[modSet], {1 → "Along-Axis Models",
         2 → "Group-Difference Models", 3 → "Subspace models"}],
       "Enter type of model set.", Top, LabelStyle →
        Directive[FontSize → 12, Bold, FontFamily → "Arial"]]],
     Row[{Panel[Labeled[PopupMenu[Dynamic[xAxisName], varNames],
         "Select variable to be plotted on x-axis.", Top, LabelStyle →
          Directive[FontSize → 12, Bold, FontFamily → "Arial"]]], "  ",
       Panel[Labeled[PopupMenu[Dynamic[yAxisName], varNames],
         "Select variable to be plotted on y-axis.", Top,
         LabelStyle → Directive[FontSize → 12, Bold, FontFamily → "Arial"]]]}],
     Row[{
       Panel[Labeled[PopupMenu[Dynamic[pltAspect],
          {1 → "Golden Ratio Plot", 2 → "Square Plot (equi-length axes)",
           3 → "True-Scale Plot (actual axis scales)"}],
         "Enter plot aspect ratio type.", Top, LabelStyle →
          Directive[FontSize → 12, Bold, FontFamily → "Arial"]]], "  ",
       Panel[Labeled[PopupMenu[Dynamic[lch], {1 → "Simple scatterplot",
           2 → "Scatterplot w/ convex hulls"}],
         "Show group domians?", Top, LabelStyle →
          Directive[FontSize → 12, Bold, FontFamily → "Arial"]]], "  ",
       Panel[Labeled[PopupMenu[Dynamic[ptsJoin], {1 → "No", 2 → "Yes"}],
         "Join datapoints?", Top,
         LabelStyle → Directive[FontSize → 12, Bold, FontFamily → "Arial"]]]}],
     Row[{Panel[Labeled[InputField[Dynamic[pltSz], FieldSize → 5],
         "Enter plot size value.", Top, LabelStyle →
          Directive[FontSize → 12, Bold, FontFamily → "Arial"]]], "  ",
       Panel[Labeled[InputField[Dynamic[iconSize], FieldSize → 5],
         "Enter plot icon size value.", Top, LabelStyle →
          Directive[FontSize → 12, Bold, FontFamily → "Arial"]]], "  ",
       Panel[Labeled[InputField[Dynamic[pltPad], FieldSize → 5],
         "Enter plot margin padding value.", Top,
         LabelStyle → Directive[FontSize → 12, Bold, FontFamily → "Arial"]]]}]},
    Center], "Model Coordinate Options", Top, LabelStyle →
    Directive[FontSize → 18, Bold, FontFamily → "Arial"]]]
pltSize = 500; iconSize = 0.02; pltPad = 0.1;
xAxisName = varNames⟦1⟧;
pltSz = 500; yAxisName = varNames⟦2⟧; ptsJoin = 1;
dataTrans = 1; pltAspect = 1 ;
lch = 2;
```

Out[•]=

Model Coordinate Options

Enter type of model set.

(●) Along–Axis Models ( ) Group–Difference Models ( ) Subspace models

Select variable to be plotted on x–axis.

Select variable to be plotted on y–axis.

Enter plot aspect ratio type.

Golden Ratio Plot

Show group domians?

Simple scatterplot

Join datapoints?

No

Enter plot size value.

pltSz

Enter plot icon size value.

iconSize

Enter plot margin padding value.

pltPad

Plot model coordinates in space of the original variables (optional).

In[•]:=

```
If[modSet == 1, modpts = Table[0.0, {numGroups * modelsPerAxis}, {2}]];
If[modSet == 2, modpts = Table[0.0, {numGroups * modelsPerAxis}, {2}]];
If[modSet == 3, modpts = Table[0.0, {xModelNum * yModelNum}, {2}]];
Do[If[xAxisName == varNames〚j〛, axis1 = j], {j, m2}];
Do[If[yAxisName == varNames〚j〛, axis2 = j], {j, m2}];

If[modSet == 1,
 axisPlots = Table[" ", {modelAxes}];
 modptsProj = Partition[modCoords, modelsPerAxis];
 modpts = Table[0.0, {modelAxes}, {modelsPerAxis}, {2}];
 Do[
  modpts〚k, i, 1〛 = modptsProj〚k, i, axis1〛;
  modpts〚k, i, 2〛 = modptsProj〚k, i, axis2〛,
  {i, modelsPerAxis}, {j, 2}, {k, modelAxes}]]

If[modSet == 2, modpts = Table[0.0, {numGroups * modelsPerAxis}, {2}]];
If[modSet == 3, modpts = Table[0.0, {xModelNum * yModelNum}, {2}]];
Do[If[xAxisName == varNames〚j〛, axis1 = j], {j, m2}];
Do[If[yAxisName == varNames〚j〛, axis2 = j], {j, m2}];

If [modSet == 2,
 Do[modpts〚i, 1〛 = modCoords〚i, axis1〛;
  modpts〚i, 2〛 = modCoords〚i, axis2〛, {i, numGroups * modelsPerAxis}]]
```

```
If[modSet == 3,
  Do[modpts[[i, 1]] = modCoords[[i, axis1]];
   modpts[[i, 2]] = modCoords[[i, axis2]], {i, nrows}]];

groupNames = Union[Group];
numGroups = Length[groupNames];
groupPosns = Table[Flatten[Position[Group, groupNames[[i]], 1]], {i, numGroups}];
eScoresT = Transpose[eScores];

x2T = Transpose[x2];
xAxis = x2T[[axis1]]; yAxis = x2T[[axis2]];
lab1 = varNames[[axis1]];
lab2 = varNames[[axis2]];
maxx = Max[xAxis];
minx = Min[xAxis];
maxy = Max[yAxis];
miny = Min[yAxis];

If[pltAspect == 1 || pltAspect == 3,
  xPlotLow = minx; xPlotHi = maxx; yPlotLow = miny; yPlotHi = maxy];
If[pltAspect == 2,
  If[minx > miny,
   xPlotLow = miny; yPlotLow = miny,
   xPlotLow = minx; yPlotLow = minx ]];
If[pltAspect == 2,
  If[maxx < maxy,
   xPlotHi = maxy; yPlotHi = maxy,
   xPlotHi = maxx; yPlotHi = maxx]];
If[pltAspect == 1, aRatio = 1 / N[GoldenRatio]];
If[pltAspect == 2, aRatio = 1];
If[pltAspect == 3, aRatio = Automatic];

tmpPoints = Transpose[List[xAxis, yAxis]];
pltPoints = Table[tmpPoints[[groupPosns[[j]]]], {j, numGroups}];
iconList = Flatten[Table[
    {Graphics[{EdgeForm[{Thickness[0.005], Black}],
       Hue[N[((numGroups + 1) - j) / numGroups]],
       Disk[{0, 0}, Scaled[iconSize]]}]}, {j, numGroups}]];

If[lch == 1 || ptsJoin == 1,
 Do[
  pltTable[[k, 1]] = pTmp =
    ListPlot[pltPoints[[k]], AspectRatio → aRatio, Frame → True, Joined → False,
     Axes → False, PlotRange → {{xPlotLow, xPlotHi}, {yPlotLow, yPlotHi}},
     PlotRangePadding → Scaled[pltPad], Ticks → Automatic, FrameLabel →
      {lab1, lab2}, PlotMarkers → iconList[[k]], ImageSize → pltSize, LabelStyle →
      Directive[FontSize → 14, Black, FontFamily → "Arial"]], {k, kg}],
```

```
 Do[
  pltTable⟦k, 1⟧ = ListPlot[pltPoints⟦k⟧, Frame → True, Axes → False,
    AspectRatio → aRatio, PlotRange → {{xPlotLow, xPlotHi}, {yPlotLow, yPlotHi}},
    PlotRangePadding → Scaled[pltPad], Ticks → Automatic,
    FrameLabel → {lab1, lab2}, LabelStyle →
     Directive[Black, FontSize → 14, FontFamily → "Arial"], ImageSize → pltSize,
    PlotStyle → Directive[Disk[], Hue[N[((kg + 1) - k) / kg]], EdgeForm[
       {Thickness[1.0], Black}], PointSize[Scaled[iconSize - 0.009]]]], {k, kg}]]

If[ptsJoin == 2,
  Do[
   pltTable⟦k, 2⟧ =
    ListLinePlot[pltPoints⟦k⟧, AspectRatio → aRatio, Frame → True, Joined → True,
     Axes → False, PlotStyle → Directive[Hue[N[((kg + 1) - k) / kg]], Thin],
     PlotRange → {{xPlotLow, xPlotHi}, {yPlotLow, yPlotHi}},
     PlotRangePadding → Scaled[pltPad], Ticks → Automatic,
     FrameLabel → {lab1, lab2}, ImageSize → pltSize, LabelStyle →
      Directive[FontSize → 14, Black, FontFamily → "Arial"]], {k, kg}]];

If[lch == 2,
  Do[
   hull = ConvexHullMesh[pltPoints⟦k⟧];
   pltTable⟦k, 3⟧ =
    HighlightMesh[hull, Style[2, Opacity[0.2], Hue[N[((kg + 1) - k) / kg]]],
     Frame → True, Axes → False, AspectRatio → aRatio,
     PlotRange → {{xPlotLow, xPlotHi}, {yPlotLow, yPlotHi}}, PlotRangePadding →
      Scaled[pltPad], Ticks → Automatic, FrameLabel → {lab1, lab2},
     LabelStyle → Directive[Black, FontSize → 14, FontFamily → "Arial"],
     ImageSize → pltSize], {k, kg}]];

If[ptsJoin == 1 && lch == 1, p0 = Show[pltTable⟦All, 1⟧]];
If[ptsJoin == 2 && lch == 1, p0 = Show[pltTable⟦All, 2⟧, pltTable⟦All, 1⟧]];
If[ptsJoin == 1 && lch == 2, p0 = Show[pltTable⟦All, 3⟧, pltTable⟦All, 1⟧]];
If[ptsJoin == 2 && lch == 2,
  p0 = Show[pltTable⟦All, 3⟧, pltTable⟦All, 2⟧, pltTable⟦All, 1⟧]];

iconList = Flatten[Table[
    {Graphics[{EdgeForm[{Thin, Black}],
       Black, Disk[{0, 0}, Scaled[iconSize / 2]]}]}, {j, 1}]];

If[modSet == 1,
  Do[
   axisPlots⟦i⟧ = ListPlot[modpts⟦i⟧, AspectRatio → 1 / GoldenRatio,
     Frame → True, Joined → True, Axes → False, PlotRange → Full,
     PlotStyle → Directive[Black, AbsoluteThickness[0.8]],
     PlotRangePadding → Scaled[pltPad], Ticks → Automatic,
     FrameLabel → {lab1, lab2}, PlotMarkers → iconList], {i, modelAxes}];
```

```
  p4 = Show[axisPlots]];

If[modSet == 2,
  p4 = ListPlot[modpts, AspectRatio → 1 / GoldenRatio,
    Frame → True, Joined → True, Axes → False, PlotRange → Full,
    PlotStyle → Directive[Black, AbsoluteThickness[0.8]],
    PlotRangePadding → Scaled[pltPad], Ticks → Automatic,
    FrameLabel → {lab1, lab2}, PlotMarkers → iconList]];

If[modSet == 3,
  p4 = ListPlot[modpts, AspectRatio → 1 / GoldenRatio,
    Frame → True, Joined → False, Axes → False, PlotRange → Full,
    PlotStyle → Directive[Black, AbsoluteThickness[0.8]],
    PlotRangePadding → Scaled[pltPad], Ticks → Automatic,
    FrameLabel → {lab1, lab2}, PlotMarkers → iconList]];

p1 = Labeled[Show[p0, p4], "           Original Data Plot", Top,
   LabelStyle → Directive[FontSize → 18, Bold, FontFamily → "Arial"]];
g1 = Grid[Table[
    {Graphics[{EdgeForm[{Thin, Black}], Hue[N[((numGroups + 1) - j) / numGroups]],
       Disk[]}, ImageSize → 13]}, {j, numGroups}], Frame → False];
g2 = Grid[Partition[groupNames, 1], Alignment → Left,
   BaseStyle → {FontFamily → "Arial", FontSize → 13, Italic}];
p2 = Labeled[Text[Grid[{{g1, g2}}, Alignment → Bottom, Frame → True]], "Legend",
   Top, LabelStyle → Directive[Black, FontSize → 18, Bold, FontFamily → "Arial"]];

plt2D = Grid[{{p1, p2}}, BaselinePosition → Top, Alignment → Top]
```

Export plot.

```
In[∘]:= filenameout = SystemDialogInput["FileSave"];
Export[filenameout, plt2D, "TIFF", ImageResolution → 150]

Out[∘]= /Users/n.macleod/Documents/Iris
  Results/Petal Length vs Petal Width (w: CVA Axes).tif
```

Export CVA model coordinates in space of original variables.

```
In[∘]:= filenameout = SystemDialogInput["FileSave"];
Export[filenameout, modCoords, "CSV", "TextDelimiters" → ""]

Out[∘]= /Users/n.macleod/Desktop/Dragonflies (Final)/Data & Results/Images
  (PCA-CVA)/Hindwings/CVA Results/Water Body Groups/PCA-CVA Model Coords.csv
```

Jackknife discrimination performance section

Calculate jackknifed estimate of discriminant function performance.

In[•]:=
```
eScoresJ = Table[0.0, {n2}, {m2}];
eScrsJ = Table[0.0, {m2}];
gps = Length[Union[Group]];
noVals = Min[{gps - 1, m2}];
gMeans = Table[0.0, {gps}, {noVals}];
mDist = Table[0.0, {n2}, {gps}];
distTable = Table[0.0, {n2 + 1}, {gps + 2}];
distTableOut = Table[0.0, {n2 + 1}, {gps + 2}];
n3 = n2 - 1;
m3 = m2;
smpSize2 = smpSize;

Panel[Labeled[ProgressIndicator[Dynamic[k], {1, n2}], "Calculation Progress",
  Top, LabelStyle → Directive[FontSize → 12, Bold, FontFamily → "Arial"]]]

Do[

 x3 = Drop[x2, {k}];
 xt = Take[x2, {k}];
 g3 = Drop[Group, {k}];
 gt = Take[Group, {k}];

 {gndMean, T, gNames, nGps, gpMeans, smpSize, W, B} = TWB[x3, n3, m3, Group];

 WI = PseudoInverse[W];
 sCovar = WI.B;
 eVals = Eigenvalues[sCovar];

 noVals = Min[{nGps - 1, m2}];
 eVals = Take[eVals, noVals];

 eVecs = Transpose[Eigenvectors[sCovar]];
 eScores = x3.eVecs;

 eScrs = Take[eScores, All, noVals];

 eScrsJ = xt.eVecs;
 eScoresJ[[k]] = eScrsJ;
 eScrsJ = Flatten[eScrsJ];
 eScrsJ = Take[eScrsJ, noVals];

 Do[
  Do[
```

```
    sum = 0.0;
    Do[
     If[Group⟦i⟧ == gNames⟦k3⟧,
      sum = sum + eScores⟦i, j⟧,
      sum = sum],
     {i, n3}];
    gMeans⟦k3, j⟧ = sum / smpSize2⟦k3⟧,
    {j, noVals}];,
  {k3, nGps}];
 gMeans;

 Do[
  mDist⟦k, k3⟧ = EuclideanDistance[gMeans⟦k3⟧, eScrsJ],
  {k3, nGps}];
 mDist, {k, 1, n2}]

eScoresJ = Partition[Flatten[eScoresJ], m2];
mDist;

pDist = mDist;
Do[
 n = 0;
 ref = Min[pDist⟦k⟧];
 Do[
  If[pDist⟦k, j⟧ == ref, pDist⟦k, j⟧ = Style[pDist⟦k, j⟧, Bold]],
  {j, nGps}], {k, n2}]

distTable⟦1, 1⟧ = "Object";
distTableOut⟦1, 1⟧ = "Object";
distTable⟦1, 2⟧ = "Group";
distTableOut⟦1, 2⟧ = "Group";
Do[distTable⟦1, j + 2⟧ = Text[Rotate[gNames⟦j⟧, 90 Degree]], {j, nGps}]
Do[distTableOut⟦1, j + 2⟧ = gNames⟦j⟧, {j, nGps}]
Do[
 distTable⟦i + 1, 1⟧ = objNames⟦i⟧;
 distTableOut⟦i + 1, 1⟧ = objNames⟦i⟧;
 distTable⟦i + 1, 2⟧ = Group⟦i⟧;
 distTableOut⟦i + 1, 2⟧ = Group⟦i⟧;
 Do[distTable⟦i + 1, j + 2⟧ = PaddedForm[pDist⟦i, j⟧, {4, 3}], {j, nGps}];
 Do[distTableOut⟦i + 1, j + 2⟧ = pDist⟦i, j⟧, {j, nGps}],
 {i, n2}]

distances = Labeled[Grid[distTable, BaseStyle → (FontFamily → "Arial"),
   Alignment → {{Left, Center, Center, Center, Center, Center, Center, Center},
     {Bottom, Baseline}}, Frame → True,
   Dividers → {{True, True, True}, {True, True}}], "Jackknifed Distance Table",
  Top, LabelStyle → Directive[FontSize → 14, Bold, FontFamily → "Arial"]]
```

```
Print[" "]

cMat = Table[0, {nGps}, {nGps}];
gPos = Table[Flatten[Position[Group, gNames⟦i⟧, 1]], {i, nGps}];
pDist = mDist;

Do[
 Do[
  Do[
   gPos⟦k, ig⟧;
   pDist⟦gPos⟦k, ig⟧⟧;
   ref = Min[pDist⟦gPos⟦k, ig⟧⟧];
   pDist⟦gPos⟦k, ig⟧, j⟧;
   If[pDist⟦gPos⟦k, ig⟧, j⟧ == ref,
    cMat⟦k, j⟧ = cMat⟦k, j⟧ + 1, cMat⟦k, j⟧ = cMat⟦k, j⟧],
   {j, nGps}],
  {ig, smpSize2⟦k⟧}],
 {k, nGps}]
cMat;

c = Tr[cMat];
s = Total[cMat, nGps];
num = c * s;
Do[
  num = num - (Total[Take[cMat⟦i, All⟧]] * Total[Take[cMat⟦All, i⟧]]), {i, nGps}];
d1 = s^2;
Do[d1 = d1 - Total[Take[cMat⟦i, All⟧]]^2, {i, nGps}];
d2 = s^2;
Do[d2 = d2 - Total[Take[cMat⟦All, i⟧]]^2, {i, nGps}];
denom = N[√(d1 * d2)];
mcc = num / denom;

cmTable = Table[" ", {nGps + 5}, {nGps + 4}];
cmOut = Table[" ", {nGps + 5}, {nGps + 4}];
cmTable⟦1, 1⟧ = "Groups";
cmOut⟦1, 1⟧ = "Groups";
Do[cmTable⟦1, j + 1⟧ = Text[Rotate[gNames⟦j⟧, 90 Degree]], {j, nGps}];
Do[cmOut⟦1, j + 1⟧ = gNames⟦j⟧, {j, nGps}];
Do[cmTable⟦j + 1, 1⟧ = gNames⟦j⟧, {j, nGps}];
Do[cmOut⟦j + 1, 1⟧ = gNames⟦j⟧, {j, nGps}];
cmTable⟦nGps + 2, 1⟧ = "Total Correct";
cmOut⟦nGps + 2, 1⟧ = "Total Correct";
cmTable⟦nGps + 3, 1⟧ = "Total Estimated";
cmOut⟦nGps + 3, 1⟧ = "Total Estimated";
cmTable⟦nGps + 4, 1⟧ = "Percent Estimated Correctly";
cmOut⟦nGps + 4, 1⟧ = "Percent Estimated Correctly";
```

```
cmTable⟦nGps + 5, 1⟧ = "Matthew's Correlation Coef.";
cmOut⟦nGps + 5, 1⟧ = "Matthew's Correlation Coef.";
cmTable⟦1, nGps + 3⟧ = Text[Rotate["Group Totals", 90 Degree]];
cmOut⟦1, nGps + 3⟧ = "Group Totals";
cmTable⟦1, nGps + 2⟧ = Text[Rotate["Total Correct", 90 Degree]];
cmOut⟦1, nGps + 2⟧ = "Total Correct";
cmTable⟦1, nGps + 4⟧ = Text[Rotate["Percent Correct", 90 Degree]];
cmOut⟦1, nGps + 4⟧ = "Percent Correct";
Do[cmTable⟦i + 1, j + 1⟧ = cMat⟦i, j⟧, {i, nGps}, {j, nGps}];
Do[cmOut⟦i + 1, j + 1⟧ = cMat⟦i, j⟧, {i, nGps}, {j, nGps}];
cTot = Total[cMat];
rTot = Diagonal[cMat];
gpTot = Total[cMat, {2}];
gTot = Total[cTot];

Do[cmTable⟦i + 1, nGps + 2⟧ = rTot⟦i⟧, {i, nGps}];
Do[cmOut⟦i + 1, nGps + 2⟧ = rTot⟦i⟧, {i, nGps}];
Do[cmTable⟦i + 1, nGps + 3⟧ = gpTot⟦i⟧, {i, nGps}];
Do[cmOut⟦i + 1, nGps + 3⟧ = gpTot⟦i⟧, {i, nGps}];
Do[cmTable⟦i + 1, nGps + 4⟧ =
   PaddedForm[N[(rTot⟦i⟧ / gpTot⟦i⟧) * 100.0], {4, 2}], {i, nGps}];
Do[cmOut⟦i + 1, nGps + 4⟧ = N[(rTot⟦i⟧ / gpTot⟦i⟧) * 100.0], {i, nGps}];
cmTable⟦nGps + 2, nGps + 3⟧ = gTot;
cmOut⟦nGps + 2, nGps + 3⟧ = gTot;
cmTable⟦nGps + 2, nGps + 2⟧ = Total[rTot];
cmOut⟦nGps + 2, nGps + 2⟧ = Total[rTot];
cmTable⟦nGps + 2, nGps + 4⟧ = PaddedForm[N[(Total[rTot] / gTot) * 100.0], {4, 2}];
cmOut⟦nGps + 2, nGps + 4⟧ = N[(Total[rTot] / gTot) * 100.0];

Do[cmTable⟦nGps + 2, i + 1⟧ = rTot⟦i⟧, {i, nGps}]
Do[cmOut⟦nGps + 2, i + 1⟧ = rTot⟦i⟧, {i, nGps}]
Do[cmTable⟦nGps + 3, i + 1⟧ = cTot⟦i⟧, {i, nGps}]
Do[cmOut⟦nGps + 3, i + 1⟧ = cTot⟦i⟧, {i, nGps}]
Do[cmTable⟦nGps + 4, i + 1⟧ =
   PaddedForm[N[(rTot⟦i⟧ / cTot⟦i⟧) * 100.0], {4, 2}], {i, nGps}];
Do[cmOut⟦nGps + 4, i + 1⟧ = N[(rTot⟦i⟧ / cTot⟦i⟧) * 100.0], {i, nGps}];
cmTable⟦nGps + 3, nGps + 2⟧ = Total[cTot];
cmOut⟦nGps + 3, nGps + 2⟧ = Total[cTot];
cmTable⟦nGps + 4, nGps + 2⟧ =
  PaddedForm[N[(Total[rTot] / Total[cTot]) * 100.0], {4, 2}];
cmOut⟦nGps + 4, nGps + 2⟧ = N[(Total[rTot] / Total[cTot]) * 100.0];
cmTable⟦nGps + 5, 2⟧ = PaddedForm[mcc, {4, 3}];
cmOut⟦nGps + 5, 2⟧ = mcc;

confusion = Labeled[Grid[cmTable, Frame → True,
   Dividers → {{2 → True, -4 → True}, {2 → True, -2 → True, -5 → True}},
   BaseStyle → (FontFamily → "Arial"),
```

```
  Alignment → {{Right, Center}, {Bottom, Baseline}}], "Raw Confusion Matrix",
 Top, LabelStyle → Directive[FontSize → 14, Bold, FontFamily → "Arial"]]
```

Out[•]=

Calculation Progress

Export jackknifed distance table.

In[•]:=

```
filenameout = SystemDialogInput["FileSave"];
Export[filenameout, distTableOut, "CSV", "TextDelimiters" → ""]
```

Out[•]= /Users/n.macleod/Desktop/Bumpus Sparrow Results/Distance Table (Jack).csv

Export jackknifed confusion matrix table.

In[•]:=

```
filenameout = SystemDialogInput["FileSave"];
Export[filenameout, cmOut, "CSV", "TextDelimiters" → ""]
```

Out[•]= /Users/n.macleod/Desktop/Bumpus Sparrow Results/Confusion Matrix (Jack).csv

Data Projection Module

This routine allows you to import data for objects that were not used to contruct the CVA space and project them into the CVA space with full support for data visualization graphics.

Read in projection datafile & partition into datasets.

In[•]:=
```
filenamein = SystemDialogInput["FileOpen"];
x3 = Import[filenamein, "CSV"];
filenamein

{nProj, mProj} = Dimensions[x3];
varNames = Flatten[Take[x3, 1]];
x4 = Drop[x3, 1];
varNames = Drop[varNames, 1];
varNames = Drop[varNames, 1];

projObjectNames = Flatten[Take[x4, nProj - 1, 1]];
x4 = Drop[x4, 0, 1];

projGroup = Flatten[Take[x4, nProj - 1, 1]];
x4 = Drop[x4, 0, 1];
numProjGroups = Length[Union[projGroup]];

{n4, m4} = Dimensions[x4];

Print["No. of groups: ", Length[Union[Group]]];
Print["No. of objects: ", n4];
Print["No. of variables: ", m4];
```

Out[•]=
```
/Users/n.macleod/Desktop/Data (5).csv
```

```
No. of groups: 4

No. of objects: 20

No. of variables: 4
```

Project into CVA space and combine with original data.

In[•]:=
```
If[meanTrans == 2, Do[x4⟦i⟧ = x4⟦i⟧ - mVec, {i, n4}]];
If[logTrans == 2, x2 = N[Log10[x4]]];
If[stdTrans == 2, x2 = Standardize[x4]];
If[shiftTrans == 2, x4 = x4 + knsnt];

projEScores = x4.eVecs;
combEScores = Join[eScores, projEScores];
combEScoresT = Transpose[combEScores];
combN = n2 + n4;

projGpNames = projGroup;
Do[projGpNames⟦i⟧ = StringJoin["proj-", projGroup⟦i⟧], {i, n4}]
Do[projObjectNames⟦i⟧ = StringJoin[ToString[projObjectNames⟦i⟧], "*"], {i, n4}]
combGroups = Join[Group, projGpNames];
combObjectNames = Join[objNames, projObjectNames];
combGpNames = Union[combGroups];
numProjGroups = Length[combGpNames];
combProjPosns =
  Table[Flatten[Position[combGroups, combGpNames⟦i⟧, 1]], {i, numProjGroups}];
```

Projection Plot section

Plot single – axis histogram.

Specify single – variable plot (histogram) options.

In[∘]:=
```
cvNames = Table[StringJoin["CV-", ToString[i]], {i, noAxes}];
Panel[Labeled[Column[{
    Row[{Panel[Labeled[PopupMenu[Dynamic[axisName], cvNames],
        "Select canonical variate to be plotted.", Top,
        LabelStyle → Directive[FontSize → 12, Bold, FontFamily → "Arial"]]],
      "  ", Panel[Labeled[PopupMenu[Dynamic[histType],
         {1 → "Stacked", 2 → "Overlapped"}], "Select histogram type.", Top,
        LabelStyle → Directive[FontSize → 12, Bold, FontFamily → "Arial"]]]}],
    Row[{Panel[Labeled[InputField[Dynamic[noBins], FieldSize → 5],
        "Enter no. of histogram bins.", Top, LabelStyle →
         Directive[FontSize → 12, Bold, FontFamily → "Arial"]]], "  ",
      Panel[Labeled[InputField[Dynamic[hSize], FieldSize → 5],
        "Enter histogram plot size.", Top,
        LabelStyle → Directive[FontSize → 12, Bold, FontFamily → "Arial"]]]}]},
   Center], "Single Axis (Histogram) Plot Options", Top,
  LabelStyle → Directive[FontSize → 16, Bold, FontFamily → "Arial"]]]
cvAxis = 1; noBins = 15; hSize = 500; histType = 1;
```

Out[∘]=

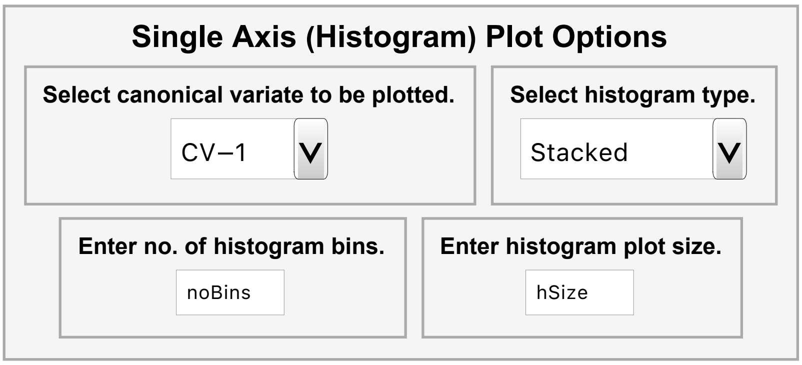

Constrruct and display histogram plot .

```
In[•]:= Do[If[axisName == cvNames[[j]], axis = j], {j, noAxes}];
Do[If[axisName == cvNames[[j]], axis = j], {j, noAxes}];
hScores = Flatten[Take[combEScores, All, {axis}]];
If[histType == 1, htype = "Stacked", htype = "Overlapped"];
projGpNames = Union[combGroups];
hPltPoints = Table[hScores[[combProjPosns[[j]]]], {j, numProjGroups}];
hueList =
  Table[Hue[N[((numProjGroups + 1) - j) / numProjGroups]], {j, numProjGroups}];
h2 = Labeled[Histogram[hPltPoints, noBins,
    ChartStyle → {hueList}, ChartLayout → htype,
    LabelStyle → Directive[FontSize → 12, Black, FontFamily → "Arial"], AxesLabel →
     {"PC Score", "Frequency"}, ImageSize → hSize, ChartLegends → projGpNames],
  StringJoin["Canonical Variate ", ToString[axis]], Top,
  LabelStyle → Directive[FontSize → 16, Bold, FontFamily → "Arial"]]
```

Export data projection histogram.

```
filenameout = SystemDialogInput["FileSave"];
Export[filenameout, projh1, "TIFF"]
```

```
/Users/nm/Projects/Storage/MacLeod/Manuscripts/01
  Developing/Wolf Dimorphism/Wolf Data/Mandibles/Data &
  Results 2/CVA Results/CV-1 Histogram (w: Unknowns).tif
```

Create 2D scatterplot (use only for datasets containing three groups or more).

Specify 2D plot options.
You must run this code after you read in the data so it can pick up the proper variable names.

```
In[•]:= cvNames = Table[StringJoin["CV-", ToString[i]], {i, noAxes}];
Panel[
 Labeled[Column[{Row[{Panel[Labeled[PopupMenu[Dynamic[xAxisName], cvNames],
        "Select canonical variate to be plotted on x-axis.", Top, LabelStyle →
         Directive[FontSize → 12, Bold, FontFamily → "Arial"]]], "  ",
      Panel[Labeled[PopupMenu[Dynamic[yAxisName], cvNames],
        "Select canonical variate to be plotted on y-axis.", Top,
        LabelStyle → Directive[FontSize → 12, Bold, FontFamily → "Arial"]]]}],
    Row[{
      Panel[Labeled[PopupMenu[Dynamic[pltAspect],
          {1 → "Golden Ratio Plot", 2 → "Square Plot (equi-length axes)",
           3 → "True-Scale Plot (actual axis scales)"}],
        "Enter plot aspect ratio type.", Top, LabelStyle →
         Directive[FontSize → 12, Bold, FontFamily → "Arial"]]], "  ",
      Panel[Labeled[PopupMenu[Dynamic[lch], {1 → "Simple scatterplot",
           2 → "Scatterplot w/ convex hulls"}],
        "Show group domians?", Top, LabelStyle →
         Directive[FontSize → 12, Bold, FontFamily → "Arial"]]], "  ",
      Panel[Labeled[PopupMenu[Dynamic[ptsJoin], {1 → "No", 2 → "Yes"}],
        "Join datapoints?", Top,
        LabelStyle → Directive[FontSize → 12, Bold, FontFamily → "Arial"]]]}],
    Row[{Panel[Labeled[InputField[Dynamic[pltSize], FieldSize → 10],
        "Enter plot size value.", Top,
        LabelStyle → Directive[FontSize → 12, Bold, FontFamily → "Arial"]]],
      "  ", Panel[Labeled[InputField[Dynamic[pltPad], FieldSize → 10],
        "Enter plot margin padding value.", Top, LabelStyle →
         Directive[FontSize → 12, Bold, FontFamily → "Arial"]]], "  ",
      Panel[Labeled[InputField[Dynamic[iconSize], FieldSize → 10],
        "Enter plot icon size value.", Top, LabelStyle →
         Directive[FontSize → 12, Bold, FontFamily → "Arial"]]]}]}, Center],
  "2D Plot Options", Top, LabelStyle → Directive[FontSize → 18,
    Bold, FontFamily → "Arial"]]]
pltSize = 500; iconSize = 0.03; pltPad = 0.1; xAxisName = cvNames⟦1⟧;
yAxisName = cvNames⟦2⟧;
 ptsJoin = 1; dataTrans = 1;  pltAspect = 1 ; lch = 2;
```

Out[∘]=

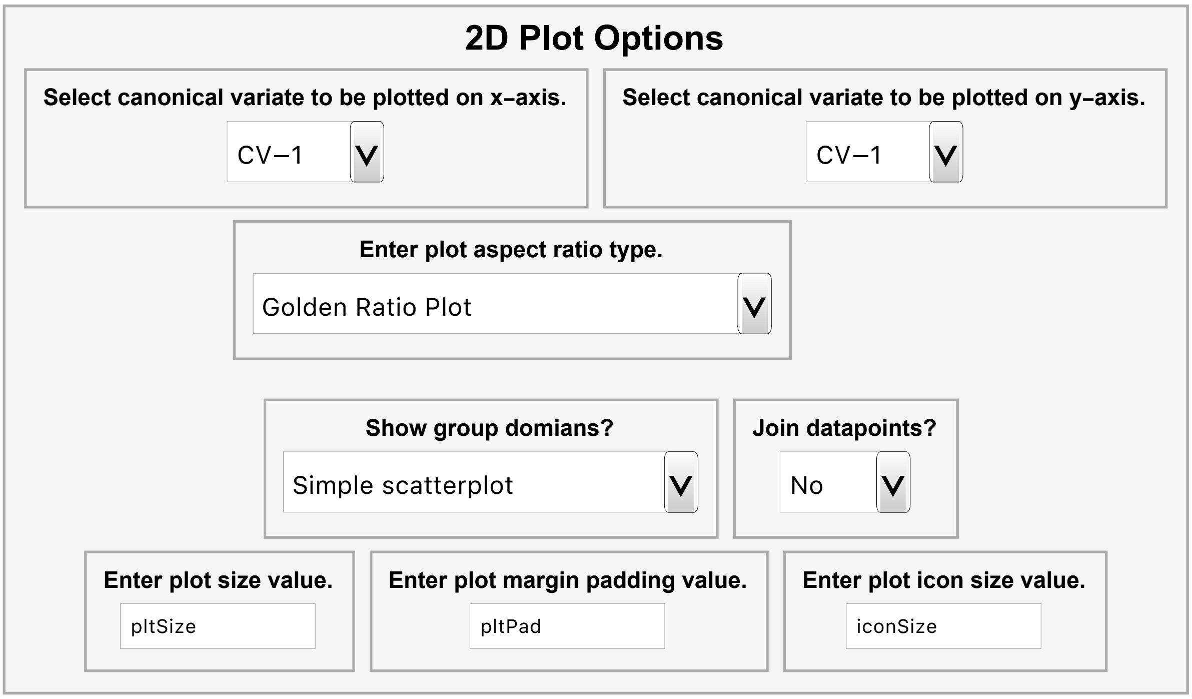

Plot script

In[∘]:=

```
projPltTable = Table[" ", {numProjGroups}, {3}];
Do[If[xAxisName == cvNames[[j]], axis1 = j], {j, noAxes}];
Do[If[yAxisName == cvNames[[j]], axis2 = j], {j, noAxes}];

xAxis = combEScoresT[[axis1]]; yAxis = combEScoresT[[axis2]];
lab1 = StringJoin[{"Canonical Variate ", ToString[axis1]},
   {" (Var. =", ToString[t3[[axis1 + 1, 3]]]}, {"%)"}];
lab2 = StringJoin[{"Canonical Variate ", ToString[axis2]},
   {" (Var. =", ToString[t3[[axis2 + 1, 3]]]}, {"%)"}];
maxx =
  Max[
   xAxis];
minx = Min[xAxis];
maxy = Max[yAxis];
miny = Min[yAxis];

If[pltAspect == 1 || pltAspect == 3,
   xPlotLow = minx; xPlotHi = maxx; yPlotLow = miny; yPlotHi = maxy];
If[pltAspect == 2,
   If[minx > miny,
    xPlotLow = miny; yPlotLow = miny,
    xPlotLow = minx; yPlotLow = minx ]];
If[pltAspect == 2,
   If[maxx < maxy,
    xPlotHi = maxy; yPlotHi = maxy,
    xPlotHi = maxx; yPlotHi = maxx]];
```

```
If[pltAspect == 1, aRatio = 1 / N[GoldenRatio]];
If[pltAspect == 2, aRatio = 1];
If[pltAspect == 3, aRatio = Automatic]

projTmpPoints = Transpose[List[xAxis, yAxis]];
projPltPoints = Table[projTmpPoints[[combProjPosns[[j]]]], {j, numProjGroups}];
iconList = Flatten[Table[
    {Graphics[
      {EdgeForm[{Thin, Black}], Hue[N[((numProjGroups + 1) - j) / numProjGroups]],
       Disk[{0, 0}, Scaled[iconSize]]}]}, {j, numProjGroups}]];

Do[
 projPltTable[[k, 1]] =
  ListPlot[projPltPoints[[k]], AspectRatio → aRatio, Frame → True, Joined → False,
   Axes → False, PlotRange → {{xPlotLow, xPlotHi}, {yPlotLow, yPlotHi}},
   PlotRangePadding → Scaled[pltPad], Ticks → Automatic, FrameLabel →
    {lab1, lab2}, PlotMarkers → iconList[[k]], ImageSize → pltSize, LabelStyle →
    Directive[FontSize → 14, Black, FontFamily → "Arial"]], {k, numProjGroups}]

If[ptsJoin == 2,
  Do[
   projPltTable[[k, 3]] = ListLinePlot[projPltPoints[[k]],
     AspectRatio → aRatio, Frame → True, Joined → True, Axes → False,
     PlotStyle → Directive[Hue[N[((numProjGroups + 1) - k) / numProjGroups]],
       Thickness[0.001]], PlotRange → {{xPlotLow, xPlotHi}, {yPlotLow, yPlotHi}},
     PlotRangePadding → Scaled[pltPad], Ticks → Automatic,
     FrameLabel → {lab1, lab2}, ImageSize → pltSize, LabelStyle → Directive[
       FontSize → 14, Black, FontFamily → "Arial"]], {k, numProjGroups}]];

If[lch == 2,
  Do[
   hull = ConvexHullMesh[projPltPoints[[k]]];
   projPltTable[[k, 2]] = HighlightMesh[hull,
     Style[2, Opacity[0.2], Hue[N[((numProjGroups + 1) - k) / numProjGroups]]],
     Frame → True, Axes → False, AspectRatio → aRatio,
     PlotRange → {{xPlotLow, xPlotHi}, {yPlotLow, yPlotHi}}, PlotRangePadding →
      Scaled[pltPad], Ticks → Automatic, FrameLabel → {lab1, lab2},
     LabelStyle → Directive[Black, FontSize → 14, FontFamily → "Arial"],
     ImageSize → pltSize], {k, numProjGroups}]];

If[ptsJoin == 1 && lch == 1, p0 = Show[projPltTable[[All, 1]]]];
If[ptsJoin == 2 && lch == 1,
  p0 = Show[projPltTable[[All, 3]], projPltTable[[All, 1]]]];
If[ptsJoin == 1 && lch == 2, p0 = Show[projPltTable[[All, 2]], projPltTable[[All, 1]]]];
If[ptsJoin == 2 && lch == 2,
  p0 = Show[projPltTable[[All, 3]], projPltTable[[All, 2]], projPltTable[[All, 1]]]];
```

```
p1 = Labeled[p0, "           CV Score Plot", Top,
    LabelStyle → Directive[FontSize → 18, Bold, FontFamily → "Arial"]];
g1 = Grid[Table[
     {Graphics[{EdgeForm[{Thin, Black}],
         Hue[N[((numProjGroups + 1) - j) / numProjGroups]], Disk[]},
        ImageSize → 13]}, {j, numProjGroups}], Frame → False];
g2 = Grid[Partition[combGpNames, 1], Alignment → Left,
    BaseStyle → {FontFamily → "Arial", FontSize → 14
      , Italic}];
p2 = Labeled[Text[Grid[{{g1, g2}}, Alignment → Bottom, Frame → True]], "Legend",
    Top, LabelStyle → Directive[Black, FontSize → 18, Bold, FontFamily → "Arial"]];

jPlt2D = Grid[{{p1, p2}}, BaselinePosition → Top, Alignment → Top]
```

Export current 2 D plot.

```
In[◦]:= filenameout = SystemDialogInput["FileSave"];
        Export[filenameout, Plt2D, "TIFF", ImageResolution → 150]
```

```
Out[◦]= /Users/n.macleod/Documents/Iris Results/Test Dataset Results/CV-1 vs CV-2.tif
```

Label plotted points.

```
pn1 = p0;
projNamePoints = projTmpPoints;
projTempPointsT = Transpose[projTmpPoints];
mxY = Max[projTempPointsT[[2]]];
mnY = Min[projTempPointsT[[2]]];
incY = N[(mxY - mnY) / 15];
Do[projNamePoints[[i, 2]] = projTmpPoints[[i, 2]] - incY, {i, combN}]
nPointsTable =
  Table[{Text[combObjectNames[[i]], projNamePoints[[i]], {-1, 0}]}, {i, combN}];
pn2 = Graphics[nPointsTable, Frame → True, AspectRatio → aRatio , Axes → False,
   FrameLabel → {lab1, lab2}, PlotRangePadding → Scaled[pltPad], BaseStyle →
    Directive[FontSize → 12, FontFamily → "Arial"], ImageSize → pltSize];
p1 = Labeled[Show[pn1, pn2, BaseStyle → {FontFamily → "Arial"}],
   "          CV Score Plot", Top,
   LabelStyle → Directive[FontSize → 18, Bold, FontFamily → "Arial"]];

Plt2D = Grid[{{p1, p2}}, BaselinePosition → Top, Alignment → Top]
pn1 = p0;
projNamePoints = projTmpPoints;
projTempPointsT = Transpose[projTmpPoints];
mxY = Max[projTempPointsT[[2]]];
mnY = Min[projTempPointsT[[2]]];
incY = N[(mxY - mnY) / 15];
Do[projNamePoints[[i, 2]] = projTmpPoints[[i, 2]] - incY, {i, combN}]
nPointsTable =
  Table[{Text[combObjectNames[[i]], projNamePoints[[i]], {-1, 0}]}, {i, combN}];
pn2 = Graphics[nPointsTable, Frame → True, AspectRatio → aRatio , Axes → False,
   FrameLabel → {lab1, lab2}, PlotRangePadding → Scaled[pltPad], BaseStyle →
    Directive[FontSize → 12, FontFamily → "Arial"], ImageSize → pltSize];
p1 = Labeled[Show[pn1, pn2, BaseStyle → {FontFamily → "Arial"}],
   "          CV Score Plot", Top,
   LabelStyle → Directive[FontSize → 18, Bold, FontFamily → "Arial"]];

Plt2D = Grid[{{p1, p2}}, BaselinePosition → Top, Alignment → Top]
Print["Projected point names marked with '*'."]
```

Export current 2 D plot.

```
In[◦]:= filenameout = SystemDialogInput["FileSave"];
Export[filenameout, Plt2D, "TIFF", ImageResolution → 150]
```

```
Out[◦]= /Users/n.macleod/Documents/Iris
  Results/Test Dataset Results/CV-1 vs CV-2 (w: Labels).tif
```

Create 3 D scatterplot (use only for datasets containing four groups or more).

Specify 3 D plot options.
You must run this code after you read in the data so it can pick up the proper variable names.

```
In[•]:= cvNames = Table[StringJoin["CV-", ToString[i]], {i, noAxes}];
Panel[
 Labeled[Column[{Row[{Panel[Labeled[PopupMenu[Dynamic[xAxisName], cvNames],
        "Select variable to be plotted on x-Axis.", Top, LabelStyle →
         Directive[FontSize → 12, Bold, FontFamily → "Arial"]]], "  ",
      Panel[Labeled[PopupMenu[Dynamic[yAxisName], cvNames],
        "Select variable to be plotted on y-Axis.", Top, LabelStyle →
         Directive[FontSize → 12, Bold, FontFamily → "Arial"]]], "  ",
      Panel[Labeled[PopupMenu[Dynamic[zAxisName], cvNames],
        "Select variable to be plotted on z-Axis.", Top,
        LabelStyle → Directive[FontSize → 12, Bold, FontFamily → "Arial"]]]}],
    Row[{
      Panel[Labeled[PopupMenu[Dynamic[pltAspect],
          {1 → "Golden Ratio Plot", 2 → "Square Plot (equi-length axes)",
           3 → "True-Scale Plot (actual axis scales)"}],
        "Enter plot aspect ratio type.", Top, LabelStyle →
         Directive[FontSize → 12, Bold, FontFamily → "Arial"]]], "  ",
      Panel[Labeled[PopupMenu[Dynamic[lch], {1 → "Simple scatterplot",
           2 → "Scatterplot w/ convex hulls"}],
        "Show group domians?", Top, LabelStyle →
         Directive[FontSize → 12, Bold, FontFamily → "Arial"]]], " ",
      Panel[Labeled[PopupMenu[Dynamic[ptsJoin], {1 → "No", 2 → "Yes"}],
        "Join datapoints?", Top,
        LabelStyle → Directive[FontSize → 12, Bold, FontFamily → "Arial"]]]}],
    Row[{Panel[Labeled[InputField[Dynamic[pltSize], FieldSize → 5],
        "Enter plot size value.", Top,
        LabelStyle → Directive[FontSize → 12, Bold, FontFamily → "Arial"]]],
      " ", Panel[Labeled[InputField[Dynamic[pltPad], FieldSize → 5],
        "Enter plot margin padding value.", Top, LabelStyle →
         Directive[FontSize → 12, Bold, FontFamily → "Arial"]]], " ",
      Panel[Labeled[InputField[Dynamic[iconSize3D], FieldSize → 5],
        "Enter plot icon size value.", Top, LabelStyle →
         Directive[FontSize → 12, Bold, FontFamily → "Arial"]]]}]}, Center],
  "3D Plot Options", Top, LabelStyle → Directive[FontSize → 18,
    Bold, FontFamily → "Arial"]]]
pltSize = 500; iconSize3D = 60; pltPad = 0.1; xAxisName = cvNames⟦1⟧;
 yAxisName = cvNames⟦2⟧;
zAxisName = cvNames⟦3⟧; ptsJoin = 1;
dataTrans = 1; pltAspect = 3 ;
lch = 1;
```

Out[•]=

3D Plot Options

Select variable to be plotted on x–Axis.
CV–1

Select variable to be plotted on y–Axis.
CV–1

Select variable to be plotted on z–Axis.
CV–1

Enter plot aspect ratio type.
Golden Ratio Plot

Show group domians?
Simple scatterplot

Join datapoints?
No

Enter plot size value.
pltSize

Enter plot margin padding value.
pltPad

Enter plot icon size value.
iconSize3D

Plot script

In[•]:=

```
Do[If[xAxisName == cvNames[[j]], axis1 = j], {j, noAxes}]
Do[If[yAxisName == cvNames[[j]], axis2 = j], {j, noAxes}]
Do[If[zAxisName == cvNames[[j]], axis3 = j], {j, noAxes}]

xAxis = combEScoresT[[axis1]];
yAxis = combEScoresT[[axis2]];
zAxis = combEScoresT[[axis3]];
lab1 = StringJoin["CV-", ToString[axis1]];
lab2 = StringJoin["CV-", ToString[axis2]];
lab3 = StringJoin["CV-", ToString[axis3]];
maxx = Max[xAxis];
minx = Min[xAxis];
maxy = Max[yAxis];
miny = Min[yAxis];
maxz = Max[zAxis]; minz = Min[zAxis];

mxax = Max[xAxis];
mnax = Min[xAxis];
If[pltAspect < 3, f = N[iconSize3D * 0.020]];
If[pltAspect == 3, f = N[iconSize3D * 0.0005]];
iSz3D = (mxax - mnax) * f;
```

```
If[pltAspect == 1 || pltAspect == 3,
  xPlotLow = minx; xPlotHi = maxx; yPlotLow = miny; yPlotHi = maxy];
If[pltAspect == 2,
  If[minx > miny,
   xPlotLow = miny; yPlotLow = miny,
   xPlotLow = minx; yPlotLow = minx ]];
If[pltAspect == 2,
  If[maxx < maxy,
   xPlotHi = maxy; yPlotHi = maxy,
   xPlotHi = maxx; yPlotHi = maxx]];

If[pltAspect == 1, bRatio = {1.61803, 1, 1}];
If[pltAspect == 2, bRatio = {1, 1, 1}];
If[pltAspect == 3, bRatio = Automatic];

projPoints3 = Transpose[List[xAxis, yAxis, zAxis]];
comGp1 = combProjPosns;
combProjH = Table[0, {combN}];
Do[
  tmp = comGp1[[i]];
  itr = Length[tmp];
  Do[combProjH[[tmp[[j]]]] = Hue[N[((numProjGroups + 1) - i) / numProjGroups]],
   {j, itr}], {i, numProjGroups}];

If[pltAspect == 3,
  pltPoints = Table[{combProjH[[i]], Sphere[projPoints3[[i]], iSz3D]}, {i, combN}];
  p0 = Graphics3D[pltPoints, Axes → True,
    Boxed → True, PlotRangePadding → Scaled[pltPad],
    LabelStyle → Directive[FontSize → 12, Black, FontFamily → "Arial"],
    AxesLabel → {lab1, lab2, lab3}, ImageSize → pltSize, BoxRatios → Automatic]];
If[pltAspect ≠ 3,
  pltPoints = Table[{combProjH[[i]],
     AbsolutePointSize[iSz3D], Point[projPoints3[[i]]]}, {i, combN}];
  p0 = Graphics3D[pltPoints, Axes → True, Boxed → True,
    PlotRangePadding → Scaled[pltPad],
    LabelStyle → Directive[FontSize → 12, Black, FontFamily → "Arial"],
    AxesLabel → {lab1, lab2, lab3}, ImageSize → pltSize, BoxRatios → bRatio]];
If[ptsJoin == 2,
  pltLineTable = Table[" ", {numProjGroups}];
  gpPoints = Table[projPoints3[[combProjPosns[[j]]]], {j, numProjGroups}];
  Do[
   pltLineTable[[k]] = Graphics3D[
     {Hue[N[((numProjGroups + 1) - k) / numProjGroups]], Line[gpPoints[[k]]]},
     Axes → True, Boxed → True, PlotRangePadding → Scaled[pltPad],
     LabelStyle → Directive[FontSize → 12, Black, FontFamily → "Arial"],
     AxesLabel → {lab1, lab2, lab3}, ImageSize → pltSize,
```

```
      BoxRatios → bRatio, ViewPoint → {xax, yax, zax}], {k, numProjGroups}];
  p0 = Show[{pltLineTable, p0}]];
If[lch == 2,
  pltMeshTable = Table[" ", {numProjGroups}];
  gpPoints = Table[projPoints3⟦combProjPosns⟦j⟧⟧, {j, numProjGroups}];
  Do[
   {n3, m3} = Dimensions[gpPoints⟦k⟧];
   cHull3D = ConvexHullMesh[gpPoints⟦k⟧, BaseStyle → {EdgeForm[]},
     Boxed → True, Axes → True, PlotRangePadding → Scaled[pltPad],
     LabelStyle → Directive[FontSize → 12, Black, FontFamily → "Arial"],
     AxesLabel → {lab1, lab2, lab3}, ImageSize → pltSize,
     BoxRatios → bRatio, ViewPoint → {xax, yax, zax}];
   pltMeshTable⟦k⟧ = Show[{HighlightMesh[cHull3D,
       Style[2, Opacity[0.2], Hue[N[((numProjGroups + 1) - k) / numProjGroups]]]],
      p0}], {k, numProjGroups}];
  p0 = Show[pltMeshTable]];


p1 = Labeled[p0, "           CV Score Plot", Top,
   LabelStyle → Directive[FontSize → 18, Bold, FontFamily → "Arial"]];
h2 = Table[0, {numProjGroups}];
l1 = Table[" ", {numProjGroups}, {2}];
horiz = Table["Left", numProjGroups];
vert = Table["Center", numProjGroups];
Do[h2⟦i⟧ = Hue[N[((numProjGroups + 1) - i) / numProjGroups]], {i, numProjGroups}];
If[pltAspect == 3,
  i1 = Table[{h2⟦i⟧, Sphere[{0, 0, 0}, 0.01]}, {i, numProjGroups}];
  Do[l1⟦i, 1⟧ = Graphics3D[i1⟦i⟧, Boxed → False, ImageSize → 16],
   {i, numProjGroups}]];
If[pltAspect ≠ 3,
 i1 = Table[{h2⟦i⟧, EdgeForm[{Directive[{Thickness[0.05]}]}],
     Disk[{0, 0}, 0.001]}, {i, numProjGroups}];
 Do[l1⟦i, 1⟧ = Graphics[i1⟦i⟧, ImageSize → 12], {i, numProjGroups}]]
Do[l1⟦i, 2⟧ = Style[combGpNames⟦i⟧, FontFamily → "Arial",
    FontSlant → Italic, FontSize → 14], {i, numProjGroups}];
g2 = Labeled[Grid[l1, Frame → True, Alignment → {horiz, vert},
    Spacings → {1, 0.5}], "Legend", Top,
   LabelStyle → Directive[Black, FontSize → 16, Bold, FontFamily → "Arial"]];

plt3D = Grid[{{p1, g2}}, BaselinePosition → Top, Alignment → Top]
```

Adjust orientation of 3 D plot (if necessary).
You must replot the data to activate the changes. These

changes will be able to be exported using the script below.

In[•]:=
```
Panel[
 Labeled[Row[{Labeled[Slider[Dynamic[xax], {-10, 10}, Appearance → "Labeled"],
      "x-Axis Viewpoint", Top,
      LabelStyle → Directive[FontSize → 10, Bold, FontFamily → "Arial"]] ×
    Labeled[Slider[Dynamic[yax], {-10, 10}, Appearance → "Labeled"],
      "y-Axis Viewpoint", Top,
      LabelStyle → Directive[FontSize → 10, Bold, FontFamily → "Arial"]] ×
    Labeled[Slider[Dynamic[zax], {-10, 10}, Appearance → "Labeled"],
      "z-Axis Viewpoint", Top,
      LabelStyle → Directive[FontSize → 10, Bold, FontFamily → "Arial"]]}],
  "3D Plot Orientation Controls", Top, LabelStyle →
   Directive[FontSize → 14, Bold, FontFamily → "Ariel"]]]
xax = 2.5; yax = -2.5; zax = 2.5;
```

Out[•]=

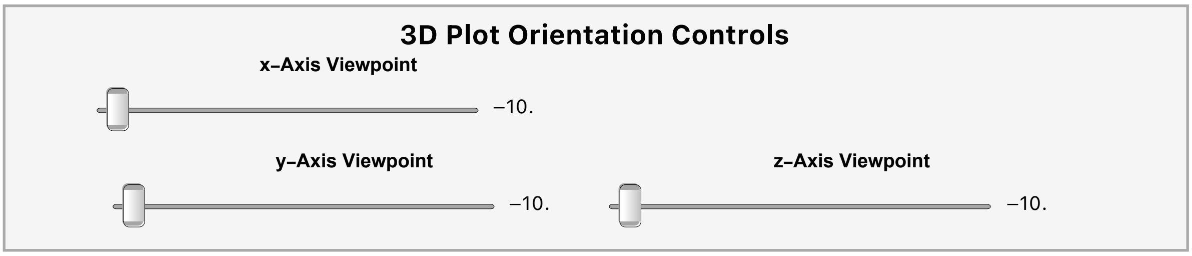

Export current 3 D plot.

```
filenameout = SystemDialogInput["FileSave"];
Export[filenameout, plt3D, "TIFF", ImageResolution → 150]
/Users/nm/Desktop/Fusulinids/PCA Results/PC-1 vs PC-2 vs PC-3.tif
```

Calculate automated identifications.

Calculate distance table and ID summary (optional).

In[•]:=
```
projMDist = Table[0.0, {n4}, {numGroups}];
projDistTable = Table[0.0, {n4 + 1}, {numGroups + 3}];
projEScores = Take[projEScores, All, noAxes];

Do[
  Do[
   projMDist[[i, k]] = EuclideanDistance[gpCVMeans[[k]], projEScores[[i]]],
   {i, n4}],
  {k, numGroups}];

pDist = projMDist;
Do[
```

```
 n = 0;
 ref = Min[pDist[[k]]];
 Do[
  If[pDist[[k, j]] == ref, pDist[[k, j]] = Style[pDist[[k, j]], Bold]],
  {j, numGroups}],
 {k, n4}]

projDistTable[[1, 1]] = "Object";
projDistTable[[1, 2]] = "Given Group";
projDistTable[[1, 3]] = "Predicted Group";
Do[projDistTable[[1, j + 3]] = groupNames[[j]], {j, numGroups}];
Do[
 projDistTable[[i + 1, 1]] = projObjectNames[[i]];
 projDistTable[[i + 1, 2]] = projGroup[[i]];
 Do[
  ptr = 0;
  idCol = Min[projMDist[[i]]];
  Do[If[projMDist[[i, j]] == idCol, ptr = j], {j, numGroups}];
  projDistTable[[i + 1, 3]] = groupNames[[ptr]], {i, n4}];
 Do[projDistTable[[i + 1, j + 3]] = PaddedForm[pDist[[i, j]], {4, 3}], {j, numGroups}],
 {i, n4}]

distances = Labeled[
  Grid[projDistTable, BaseStyle → (FontFamily → "Arial"), Alignment → {Center},
   Frame → True, Dividers → {{True, True, True, True}, {True, True}}],
  "Projected Objects Distance Table", Top,
  LabelStyle → Directive[FontSize → 16, Bold, FontFamily → "Arial"]]

sumTable = Table[0, {3}, {numGroups + 1}];
sumTable[[1, 1]] = " ";
sumTable[[2, 1]] = "Raw Count";
sumTable[[3, 1]] = "Percentage";
Do[sumTable[[1, j + 1]] = groupNames[[j]], {j, numGroups}]
Do[Do[If[Min[projMDist[[i]]] == projMDist[[i, j]],
   sumTable[[2, j + 1]] = sumTable[[2, j + 1]] + 1], {j, numGroups}], {i, n4}]
Do[sumTable[[3, j + 1]] = N[(sumTable[[2, j + 1]] / n4) * 100, 4], {j, numGroups}]

summary = Labeled[
  Grid[sumTable, BaseStyle → (FontFamily → "Arial"), Frame → True, Dividers → All,
   Alignment → {{Left, Center}, {Left, Center}}], "ID Summary Table",
  Top, LabelStyle → Directive[FontSize → 16, Bold, FontFamily → "Arial"]]
```

Export group distance table.

```
In[•]:= filenameout = SystemDialogInput["FileSave"];
Export[filenameout, projDistTable, "CSV", "TextDelimiters" → ""]

Out[•]= /Users/n.macleod/Documents/Iris Results/Test Dataset Results/Distance Table.csv
```

Export unknown ID summary table.

```
In[•]:= filenameout = SystemDialogInput["FileSave"];
Export[filenameout, sumTable, "CSV", "TextDelimiters" → ""]

Out[•]= /Users/n.macleod/Documents/Iris
  Results/Test Dataset Results/Group Assignment Summary.csv
```

Executable modules

TWB : Calculates the Total [T] , Within [W], and Between [B] SSQ matrices

```
In[•]:= TWB[x2_, n1_, m1_, Group_] :=
  Module[{gndMean, T, sum, gNames, nGps, gpMeans, smpSize, knt, wDiff, W, B},
   gndMean = Mean[x2];
   T = Table[0.0, {m1}, {m1}];
   Do[
    Do[
      sum = 0.0;
      Do[
       sum = sum + ((x2⟦i, j1⟧ - gndMean⟦j1⟧) * (x2⟦i, j2⟧ - gndMean⟦j2⟧)),
       {i, n1}];
      T⟦j1, j2⟧ = sum,
      {j2, m1}];,
    {j1, m1}];

   grp = Group;
   gNames = DeleteDuplicates[Group];
   nGps = Length[gNames];
   gpMeans = Table[0.0, {nGps}, {m1}];
   smpSize = Table[0.0, {nGps}];

   Do[
     knt = 0;
     Do[
      If[Group⟦i⟧ == gNames⟦k⟧, knt = knt + 1, knt = knt],
      {i, n1}];
     smpSize⟦k⟧ = knt,
     {k, nGps}] ×
```

```
  smpSize;

 Do[
   Do[
     sum = 0.0;
     Do[
      If[Group⟦i⟧ == gNames⟦k⟧,
       sum = sum + x2⟦i, j⟧,
       sum = sum],
      {i, n1}];
     gpMeans⟦k, j⟧ = sum / smpSize⟦k⟧,
     {j, m1}];,
   {k, nGps}] ×
  gpMeans;

 wDiff = x2;
 Do[
   Do[
     Do[
       If[Group⟦i⟧ == gNames⟦k⟧,
        wDiff⟦i, j⟧ = wDiff⟦i, j⟧ - gpMeans⟦k, j⟧,
        wDiff⟦i, j⟧ = wDiff⟦i, j⟧],
       {i, n1}];,
     {j, m1}];,
   {k, nGps}] ×
  wDiff;

 W = Table[0.0, {m1}, {m1}];
 Do[
   Do[
     sum = 0.0;
     Do[
      sum = sum + (wDiff⟦i, j1⟧ * wDiff⟦i, j2⟧),
      {i, n1}];
     W⟦j1, j2⟧ = sum,
     {j2, m1}];,
   {j1, m1}] ×
  W;

 B = T - W;

 Return[{gndMean, T, gNames, nGps, gpMeans, smpSize, W, B}]
];
```
