# Supplementary material for: What you sample is what you get: ecomorphological variation in Trithemis (Odonata, Libellulidae) dragonfly wings reconsidered
Source: BMC Ecol Evol. 2022 Apr 11;22:43. doi: 10.1186/s12862-022-01978-y (PMC8996507; doi:10.1186/s12862-022-01978-y)
Supplement: Supplementary file 3 — Additional file 3: Software Archive. [file 12862_2022_1978_MOESM3_ESM.zip › Additional Files 3/Software Archive/Dimensionality Reduction (vers. 1.0).pdf]

Dimensionality Reduction

XXX

Author: N. MacLeod
Version: 1.0
Date: 5 May 2020

Initialize libraries.

In[∘]:=
```
<< ComputationalGeometry`
```

Read in data file & partition into datasets.

In[∘]:=
```
filenamein = SystemDialogInput["FileOpen"];
x1 = Import[filenamein, "CSV"];
filenamein

{n, m} = Dimensions[x1];

varNames = Flatten[Take[x1, 1]];
x2 = Drop[x1, 1];
varNames = Drop[varNames, 1];
varNames = Drop[varNames, 1];

objNames = Flatten[Take[x2, n - 1, 1]];
x2 = Drop[x2, 0, 1];

Group = Flatten[Take[x2, n - 1, 1]];
x2 = Drop[x2, 0, 1];

{n1, m1} = Dimensions[x2];
Print["No. of groups: ", Length[Union[Group]]];
Print["No. of objects: ", n1];
Print["No. of variables: ", m1];


varNames;
objNames;
Group;
x2;
```

Out[∘]=
```
/Users/n.macleod/Desktop/Vases/Whole Dataset/Vases (Super).csv

No. of groups: 34

No. of objects: 325

No. of variables: 276
```

Reduce dimensionality.

Select user-specified dimensionality-reduction options.

In[∘]:=
```
Panel[Labeled[Column[{Panel[Labeled[
      InputField[Dynamic[dimNo], FieldSize → 5], "Enter no. of dimensions.",
      Top, LabelStyle → Directive[FontSize → 12, Bold, FontFamily → "Arial"]]],
    Panel[Labeled[PopupMenu[Dynamic[redMeth], {1 → "Automatic",
        2 → "Latent Semantic Analysis", 3 → "Best Linear Method",
        4 → "Low-Rank Matrix Factorization",
        5 → "Principal Components Analysis (PCA)",
        6 → "t-Distributed Stochastic Neighbor Embedding (t-SNE)",
        7 → "Autoencoder", 8 → "Locally Linear Embedding (LLE)",
        9 → "Isomapping"}], "Select Reduction Method", Top,
      LabelStyle → Directive[FontSize → 12, Bold, FontFamily → "Arial"]]]},
   Center], "Dimensionality-Reduction Options", Top,
  LabelStyle → Directive[FontSize → 16, Bold,
    FontFamily → "Arial"]]]
dimNo = 2; redMeth = 1;
```

Out[∘]=

Dimensionality–Reduction Options

Enter no. of dimensions.

dimNo

Select Reduction Method

Automatic

Perform calculations.

In[∘]:=
```
If[redMeth == 1, rMeth = Automatic; labMeth = "Variable "];
If[redMeth == 2, rMeth = "LatentSemanticAnalysis";
  labMeth = "Latent Semantic Axis "];
If[redMeth == 3, rMeth = "Linear"; labMeth = "Best Linear Axis "];
If[redMeth == 4, rMeth = "LowRankMatrixFactorization";
  labMeth = "LRMF Axis "];
If[redMeth == 5, rMeth = "PrincipalComponentsAnalysis";
  labMeth = "PC-"];
If[redMeth == 6, rMeth = "TSNE"; labMeth = "t-SNE Axis"];
If[redMeth == 7, rMeth = "AutoEncoder"; labMeth = "Autoencoder "];
If[redMeth == 8, rMeth = "LLE"; labMeth = "LLE Axis "];
If[redMeth == 9, rMeth = "Isomap"; labMeth = "Isomap Axis "];
redPts = DimensionReduction[x2, dimNo, Method → rMeth];
x3 = redPts[x2];
```

Export results file.

```
In[•]:= hdata = Table[0, {n1 + 1}, {dimNo + 2}];
hdata⟦1, 1⟧ = "Object";
hdata⟦1, 2⟧ = "Group";
Do[hdata⟦k + 1, 1⟧ = objNames⟦k⟧, {k, n1}];
Do[hdata⟦k + 1, 2⟧ = Group⟦k⟧, {k, n1}];
knt = 0;
Do[hdata⟦1, j + 2⟧ = StringJoin["Dim-", ToString[j]], {j, dimNo}];
Do[hdata⟦i + 1, j + 2⟧ = x3⟦i, j⟧, {i, n1}, {j, dimNo}];

filenameout = SystemDialogInput["FileSave"];
Export[filenameout, hdata, "CSV"];
```
